# Structure of a cereal purple acid phytase provides new insights to phytate degradation in plants

**DOI:** 10.1016/j.xplc.2022.100305

**Published:** 2022-02-19

**Authors:** Raquel Faba-Rodriguez, Yinghong Gu, Melissa Salmon, Giuseppe Dionisio, Henrik Brinch-Pedersen, Charles A. Brearley, Andrew M. Hemmings

**Affiliations:** 1 School of Chemistry, University of East Anglia, Norwich Research Park, Norwich NR4 7TJ, UK; 2 School of Biological Sciences, University of East Anglia, Norwich Research Park, Norwich NR4 7TJ, UK; 3 Department of Agroecology, Research Center Flakkebjerg, Aarhus University, 4200 Slagelse, Denmark; 4 College of Food Science and Technology, Shanghai Ocean University, Shanghai 201306, China

**Keywords:** wheat, purple acid phytase, X-ray crystallography, stereospecificity

## Abstract

Grain phytate, a mixed metal ion salt of inositol hexakisphosphate, accounts for 60%–80% of stored phosphorus in plants and is a potent antinutrient of non-ruminant animals including humans. Through neofunctionalization of purple acid phytases (PAPhy), some cereals such as wheat and rye have acquired particularly high mature grain phytase activity. As PAPhy activity supplies phosphate, liberates metal ions necessary for seedling emergence, and obviates antinutrient effects of phytate, its manipulation and control are targeted crop traits. Here we show the X-ray crystal structure of the b2 isoform of wheat PAPhy induced during germination. This high-resolution crystal structure suggests a model for phytate recognition that, validated by molecular dynamics simulations, implicates elements of two sequence inserts (termed PAPhy motifs) relative to a canonical metallophosphoesterase (MPE) domain in forming phytate-specific substrate specificity pockets. These motifs are well conserved in PAPhys from monocot cereals, enzymes which are characterized by high specificity for phytate. Tested by mutagenesis, residues His229 in PAPhy motif 4 and Lys410 in the MPE domain, both conserved in PAPhys, are found to strongly influence phytase activity. These results explain the observed phytase activity of cereal PAPhys and open the way to the rational engineering of phytase activity *in planta*.

## Teaser

Grain phytate accounts for 60%–80% of stored phosphorus in plants and is a potent antinutrient. The high-resolution X-ray crystal structure of a wheat purple acid phytase identifies amino acid sequence motifs forming phytate-specific specificity pockets necessary for substrate recognition and hydrolysis. This structural data explains the observed phytase activity of this class of enzymes and opens the way to the rational engineering of phytase activity *in planta*.

## Introduction

Among other nutrients, seeds must accumulate a large reservoir of phosphorus to sustain seedling growth. The principal form of phosphorus storage in seeds is in the form of phytate (*myo*-inositol hexakisphosphate; InsP_6_) (Ravindran et al., 1994). The bulk of mature grain phytase activity in cereals can be attributed to phytases belonging to the large family of purple acid phosphatases (PAPs). PAPs belong to the calcineurin-like metallophosphoesterase superfamily (Matange et al., 2015) and are known to require a heterovalent bimetal center (MI, MII) for their catalytic activity. MI is always a ferric ion (Fe^3+^) and the identity of MII has been reported to be either Fe^2+^, Zn^2+^ or Mn^2+^ depending on the protein (Schenk et al., 2013; Matange et al., 2015). PAPs form two distinct groups according to their molecular weights. The first category is referred to as high molecular weight (HMW) PAPs. They are mostly large 55–60 kDa plant and invertebrate enzymes with an N-terminal regulatory domain in addition to a metallophosphoesterase (MPE) domain. HMW PAPs are often homodimers linked by a disulfide bridge formed by a conserved cysteine and contain a heteronuclear metal center with Zn^2+^ or Mn^2+^ in the MII site (Olczak et al., 2003; Schenk et al., 2013; Matange et al., 2015). The second category is formed from mammalian, plant, and invertebrate enzymes that contain only the MPE domain. They are monomers of approximately 35 kDa usually referred to as low molecular weight (LMW) PAPs and present a Fe^3+^-Fe^2+^ homobinuclear metal center (Olczak et al., 2003; Schenk et al., 2013; Matange et al., 2015). HMW and LMW PAPs were first identified in plants (Schenk et al., 2000) and then verified in humans (Flanagan et al., 2006). Although the members of this superfamily are functionally diverse and have low overall sequence similarity, both the core MPE fold and the architecture of the active site are conserved (Matange et al., 2015). Not all PAPs can effectively utilize phytate as substrate. PAPs that can hydrolyze phytate are known as PAPhy (Dionisio et al., 2011). Activity toward phytate as substrate is an unexplained diversification/specialization of the PAPs that is unique to plants where they are essential germinative activities. In contrast to the situation with other phytase families (Lim et al., 2000; Chu et al., 2004; Zeng et al., 2011), the structural basis for PAPhy specificity toward phytate essential as a precursor to rational engineering of phytase activity is not well understood.

## Results

### The X-ray crystal structure of a wheat PAPhy

To shed further light on the structure-function relationships of enzymes responsible for the degradation of phytate in cereals, we set out to solve the X-ray crystal structure of a wheat purple acid phytase. We focused our attention on a b-isoform enzyme induced during germination, TaPAPhy_b2, selected based on its stability and yield following overexpression. To reduce the heterogeneity introduced by hyperglycosylation of recombinant proteins observed following expression in *Pichia pastoris*, a glycoengineered version of the KM71H *P. pastoris* strain was constructed. In this KM71H (OCH1:G418R) strain the OCH1 gene has been replaced with G418R, which reduces mannosylation and confers geneticin resistance. The enzyme was purified by immobilized metal ion chelate chromatography and gel filtration. Crystals grown using glycoengineered recombinant protein diffracted only to low resolution, so the protein was enzymatically partially deglycosylated and repurified before crystallization. The resulting crystals grew in space group *H*3 and diffracted to 1.42 Å resolution. The X-ray crystal structure was solved by molecular replacement using red kidney bean PAP (PDB: 2QFR) as a search model.

**Figure 1:**
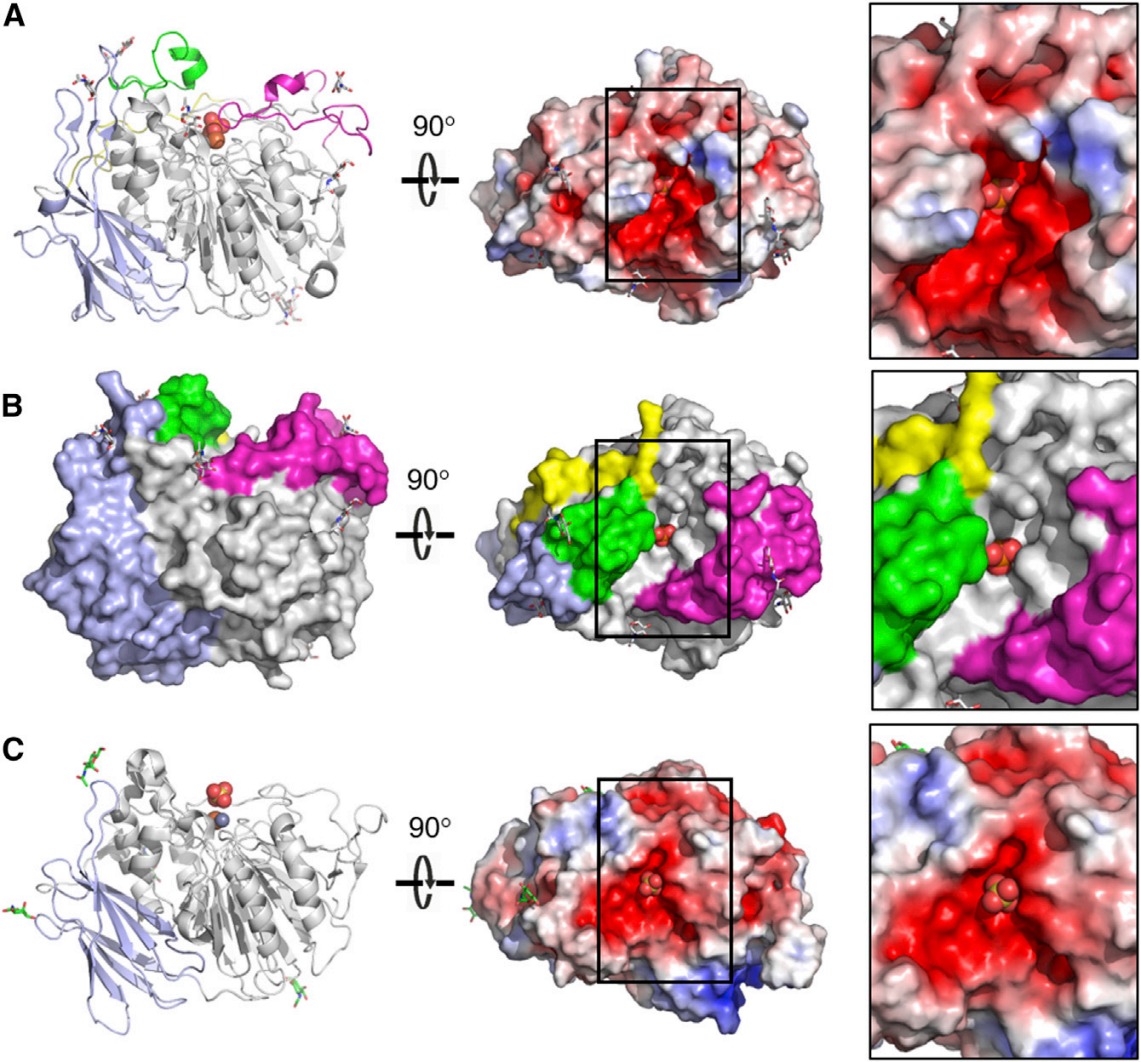
PAPhy motifs define the shape, volume, and charge distribution of the active site cavity. **(A)** Left panel, cartoon of the crystal structure of TaPAPhy_b2 in the product-bound state (this study, PDB entry 6GIT), side view. Polypeptide chain colored as follows: fibronectin-like FN3 domain, light blue; metallophosphoesterase (MPE) domain, gray; PAPhy 1 motif, yellow; PAPhy 4 motif, green; PAPhy 5 motif, light magenta. N-actylglucosamine (NAG) groups are shown as sticks, and the binuclear center and bound orthophosphate are shown in ball and stick format. Middle panel, top view of molecular surface colored by electrostatic potential (red, acidic; blue, basic). Black box indicates active site region. Right panel, expanded view of active site region. A collar of electropositive potential surrounds the bound phosphate group visible in the center. **(B)** Left panel, view of the molecular surface of TaPAPhy_b2 oriented and colored as in panel **(A)**. Middle panel, top view of surface oriented as in panel **(A)**. Right panel, expanded view of active site region. PAPhy motifs 4 (green) and 5 (light magenta) help define the shape and volume of the active site cavity. **(C)** Left panel, cartoon of the crystal structure of red kidney bean phosphatase (PDB: 2QFR), side view. Polypeptide chain colored as follows: fibronectin-like FN3 domain, light blue; metallophosphoesterase (MPE) domain, gray. NAG groups are shown as sticks and orthophosphate bound at binuclear center is shown in ball and stick format. Middle panel, top view of molecular surface colored by electrostatic potential. Black box indicates active site region. Right panel, expanded view of active site region with bound sulfate group visible in the center. The active site region is generally electronegative and lacks the features usually associated with recognition of a large, negatively charged substrate. This is consistent with low specificity toward phytate.

The refined crystal structure contains a monomer comprising residues Pro2-Leu508 of the 510-residue protein in the asymmetric unit (Figure 1). Four disulfide bonds and seven N-glycosylation sites are present, all according to previous predictions (Dionisio et al., 2011, 2012). In keeping with other HMW PAPs, TAPhy_b2 consists of a smaller fibronectin type III (FN3) non-catalytic N-terminal domain (Tsyguelnaia and Doolittle, 1998) (residues Pro43–Thr156) together with a larger C-terminal MPE domain (residues Arg168–Glu497). The structure of the core of the wheat enzyme closely resembles those of other plant HMW PAPs such as those from kidney bean (Schenk et al., 2008) (PDB: 2QFR; percentage amino acid sequence identity (PID) 34%; root-mean-square deviation (RMSD) 0.84 Å over 294 residues) and sweet potato (Schenk et al., 2005) (PDB: 1XZW; PID 35%; RMSD 0.75 Å over 291 residues). A preference for Fe^2+^ in the MII site of the MPE domain has been described for TaPAPhy_b2 (Dionisio et al., 2011), a feature arising perhaps as a route to cellular redox regulation (Rusnak and Reiter, 2000), and correspondingly two iron ions were modeled in the electron density present at the active site. Anomalous scattering peaks calculated using a dataset collected at the iron K-edge (Fe-SAD) supported this interpretation (Supplemental Figure 1). X-ray fluorescence spectra combined with absorption edge scans confirmed the absence of manganese. The iron in the MI site is tetrahedrally coordinated by residues Asp174, Tyr204, His379, and Asp201, the latter of which bridges the two metal ions. The iron in the MII site is octahedrally coordinated by Asn258, His340, His377, and the bridging residue Asp201. A phosphate ion is bound to the two metal ions and the side chains of His259, His350, and Glu409. The binding mode of the phosphate ion, together with the lack of electron density for a bridging solvent molecule, resembles that found in the structure of the red kidney bean PAP:orthophosphate complex in the product-bound state (Klabunde et al., 1996; Schenk et al., 2008) (PDB: 4KBP). We obtained a second structure of the TaPAPhy_b2:PO_4_ complex refined at 1.54 Å resolution from a further crystal in the same space group. The phosphate ion bound to the active site in this case resembled the structure of the pig PAP:orthophosphate complex in the substrate-bound state (Guddat et al., 1999; Schenk et al., 2008) (PDB: 1UTE) and included spherical electron density for a μ-hydroxide moiety bridging the two irons (Supplemental Table 1; Supplemental Figure 2). Despite screening numerous crystals, we failed to obtain a structure for TaPAPhy_b2 resembling the transition state of the reaction, a feature previously observed in the very high-resolution structure of pig PAP (Selleck et al., 2017) characterized by the phosphate ion binding only to MII.

### PAPhy motifs determine the shape and charge distribution of the active site

Previous bioinformatics analysis has identified four phytase-specific polypeptide insertions (termed PAPhy motifs 1–4) in the sequences of plant purple acid phytases relative to those of HMW PAPs, together with five PAP motifs (I–V) that identify metal-binding sequences in the MPE domain (Dionisio et al., 2011). With access to the crystal structure, we are now in a position to assign possible roles for the individual PAPhy motifs in the structure-function relationships of the enzyme. The wheat phytase motifs PAPhy 2 (Ser82–Gly87) and PAPhy 3 (Ala147–Pro158) are located within the N-terminal domain and, therefore, are unlikely to be involved in determining specificity toward phytate (although a regulatory role cannot be discounted). PAPhy 1 (Arg21–Arg37) is found near the N-terminus of the protein but does not form part of the N-terminal domain. Lying adjacent to the active site it may have a secondary role in contributing to specificity through a salt bridge interaction with PAPhy 4 involving His23 and Asp216. The PAPhy 4 motif (Leu209–His229) lies within the MPE domain and lines one side of the active site cavity. Analysis of the crystal structure revealed a further, previously unidentified polypeptide insertion lining the other side of the cavity (residues Asp418–Gln455). To investigate the conservation of this loop region among the wider PAP family, we conducted a detailed sequence analysis comprising PAPhys (both characterized and predicted) and PAPs from a variety of organisms. The resulting alignment (Supplemental Figures 3 and 4) demonstrated this loop to form part of a highly conserved sequence insertion in PAPhys that is absent in canonical PAP enzymes and which we name PAPhy 5. Together, motifs 4 and 5 create an electropositive horseshoe-shaped collar mounted on the strikingly electronegative active site landscape found in PAPs (Figure 1). By virtue of their roles in defining the shape, volume, and charge distribution of the active site cavity, the PAPhy 4 and 5 motif insertions provide ideal candidates by which the specificity of a PAP enzyme may be tuned to phytate.

### The presence of PAPhy motifs 4 and 5 correlate with high specificity toward phytate

**Figure 2:**
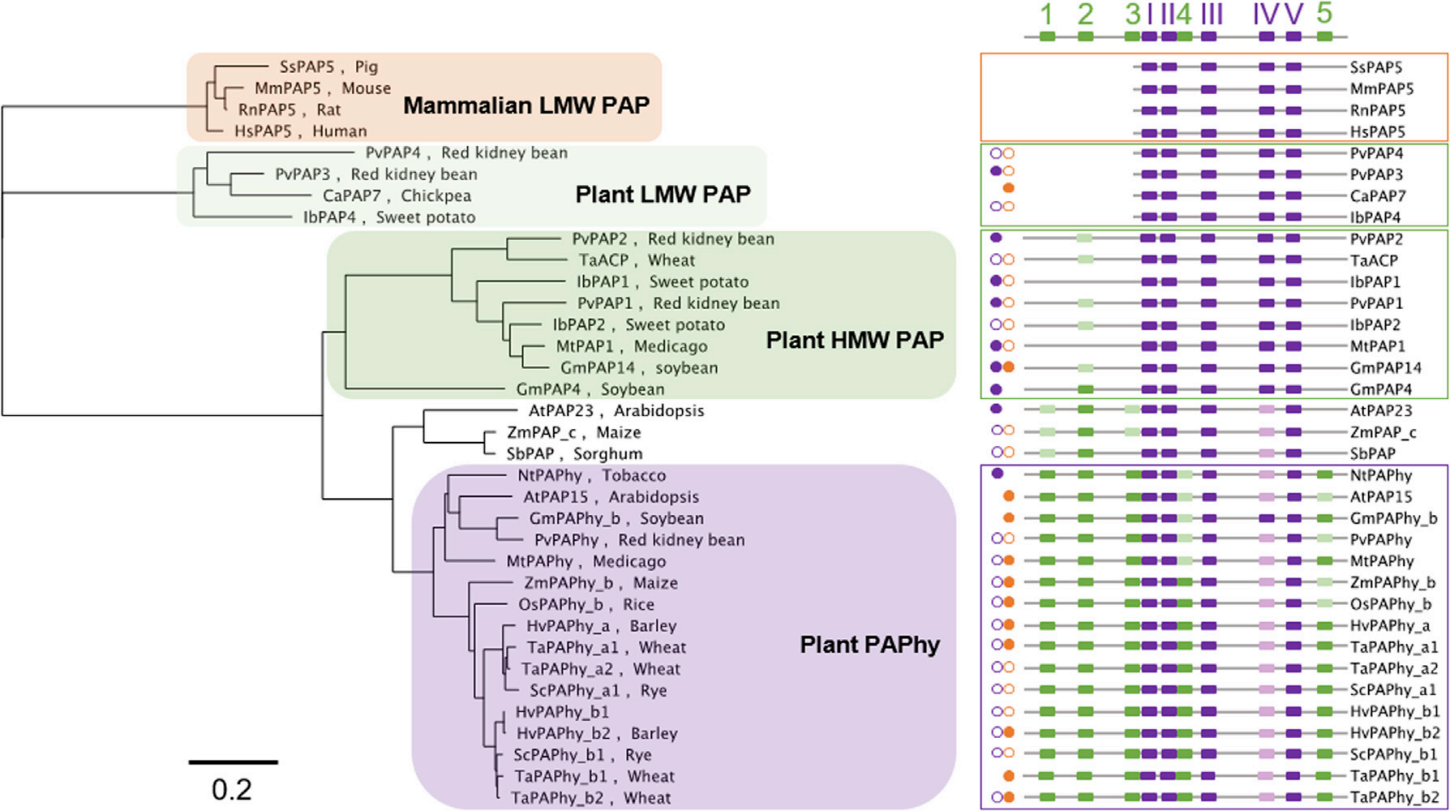
PAPhy motifs provide an insight into the emergence of specific phytase activity in plants. Left panel, phylogenetic tree constructed from a set of biochemically characterized members of PAP and PAPhy enzyme families. A key to enzyme identifiers can be found in Supplemental Table 2. Mammalian low molecular weight (LMW) PAPs are enclosed in an orange box, plant LMW PAPs in a light green box, plant HMW PAPs in a green box, and plant PAPhys in a mauve box. Right panel, schematic representation of the distribution of PAPhy motifs in sequences appearing in the phylogenetic analysis. Green boxes represent PAPhy motifs, numbered 1–5. Metal-binding PAP motifs (Dionisio et al., 2011; Schenk et al., 2013) are shown as dark purple and numbered I–V. The depth of color of both PAPhy and PAP motifs indicates their degree of similarity with darker coloring indicating higher sequence conservation. Small circles to the left of each sequence in the panel indicate documented phosphatase (closed purple) and phytase activities (closed orange), respectively. Open circles indicate predicted activities. Monocot clade enzymes possess well-conserved PAPhy motifs 4 and 5. All characterized members of this clade have high phytase activity and a low *K*_*m*_ for phytate.

To study the emergence of PAPhy motifs within the wider PAP family, a phylogenetic tree was constructed that, when combined with substrate specificity and activity data (Supplemental Table 2), provides an insight into the emergence of specificity toward phytate in PAPhys (Figure 2). LMW plant PAPs lack PAPhy motifs. Very few of these have been cloned and characterized but are predicted to have broad activity against phospho-substrates (Liang et al., 2010). A PAPhy 2-like motif appears in roughly 75% of HMW PAPs, but all lack PAPhy motifs 4 and 5. The substrate specificity of these PAPs is generally broad, although a quarter of the clade show some activity toward phytate. Between the HMW PAP clade and the PAPhy clade a further small clade of enzymes that possess an intermediate set of PAPhy motifs is found. These all contain PAPhy 1- and PAPhy 2-like motifs, with many also possessing a PAPhy 3-like motif. Only one member of this clade, AtPAP23, has been characterized, and while showing strongest activity toward other phospho-substrates, it also has a reasonable activity against phytate (Zhu et al., 2005). The plant PAPhys are split into two subclades containing either monocots or dicots. While all enzymes in both clades possess PAPhy motifs 1–5, the dicot clade contains only a partially conserved PAPhy 4 motif. Characterized dicot enzymes show a broad specificity, although we note that the tobacco and maize enzymes have a low *K*_m_ for phytate indicating a higher specificity for this substrate. On the other hand, the monocot clade enzymes possess well-conserved PAPhy motifs 4 and 5. All characterized members of this clade have high activity and a low *K*_*m*_ for phytate. The presence of PAPhy motifs 4 and 5 therefore correlates with high specificity toward phytate.

### Binding of a phytate analog inhibitor reveals a potential substrate standby binding mode

**Figure 3:**
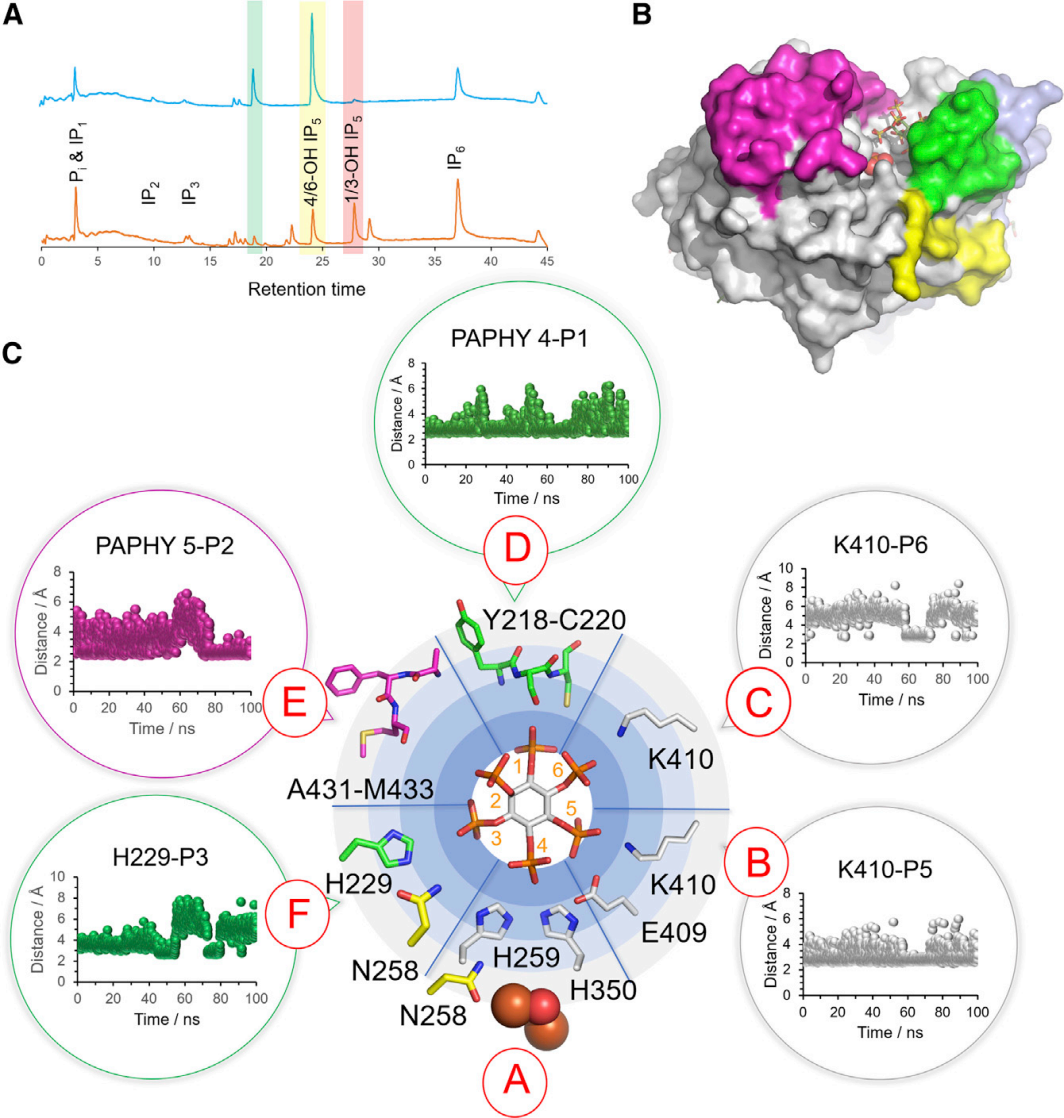
What is the structural basis for recognition of phytate? **(A)** HPLC chromatogram of InsP_6_ hydrolysis by recombinant TaPAPhy_b2 (blue trace). A chromatogram of an acid hydrolysate of the substrate (*myo-*inositol polyphosphate standards) is shown for reference (gold trace). The elution volume ranges for the various inositol polyphosphate product of phytate hydrolysis are highlighted by vertical colored backgrounds (note that the notation for the InsP_5_ products is based on the identity of the free hydroxyl group of the intermediate—red: 1/3-OH InsP_5_; yellow: 4/6-OH InsP_5_). The green vertical bar highlights 1234/1256-OH InsP_4_, a major InsP_4_ product of PAPhy activity. The potential of marginal D-1 and/or D-3 phytase activity was also noted, but a contaminant peak was also present in the undegraded substrate. **(B)** A view of the crystal structure of the complex of TaPAPhy_b2 with the substrate analog inhibitor, InsS_6_. The inhibitor is shown in stick format. The molecular surface of the enzyme is colored as in Figure 1 to reveal the PAPhy motifs. The phosphate group bound at the catalytic center is shown in sphere format. **(C)** A representation of the predicted specificity subsites (pockets) of TaPAPhy_b2 showing those active site residues that are predicted to form contacts with the bound substrate. Pockets are labeled A–F (red capital letters in red circles). Contact residues are labeled and shown in stick format, and colored according to their assignment within either the PAPhy motif 4 (green), the PAPhy motif 5 (light magenta), or the MPE domain (gray). Note that Asn258 is also a ligand to iron in the MII site and is colored yellow. In the center of the image sits a stick representation of InsP_6_ positioned so that the D-4 phosphate is located in specificity pocket A. This orientation places the axial 2-phosphate in specificity pocket E. Also shown for individual specificity pockets B–F are plots of the minimum contact distance from individual residues (H229, K410) or elements of PAPhy motifs 4 and 5 to the corresponding phosphate group of the substrate during the 100-ns MD simulation.

Wheat phytases and, in general, plant phytases are commonly classified as 6-phytases (EC 3.1.3.26), that is, they display a preference of hydrolysis for the L-6 (D-4) phosphate of phytate (Lim and Tate, 1973; Nakano et al., 1999, 2000; Brinch-Pedersen et al., 2002; Bohn et al., 2007; Bohn et al., 2008; Wu et al., 2015). To investigate the hydrolysis of phytate by the recombinant enzyme, inositol polyphosphate products of hydrolysis were separated by acid elution from a high-performance liquid chromatography (HPLC) anion exchange column with subsequent detection of inositol phosphate-ferric complexes (Phillippy and Bland, 1988; Blaabjerg et al., 2010) (Figure 3A). As expected, recombinant TaPAPhy_b2 showed a strong preference for initial hydrolysis of the phosphate in position D-4 and/or D-6 of the inositol ring (since these columns do not resolve enantiomers, it is not possible to conclude whether the peak corresponds to one or both intermediates). To investigate the structural basis for this observed positional hydrolytic specificity, we grew cocrystals of the complex of TaPhy_b2 with the non-hydrolyzable phytate analog, *myo*-inositol hexakissulfate (InsS_6_). While InsS_6_ inhibits the phytase activity of TaPAPhy_b2 *in vitro*, the crystal structure reveals it to fail to bind to the enzyme in such a way as to mimic phytate in a productive binding mode, contrary to what is typically seen for other classes of phytase (Lim et al., 2000; Chu et al., 2004; Zeng et al., 2011; Acquistapace et al., 2020). Instead, it binds at a site immediately adjacent to the active center (Figure 3B; Supplemental Figures 5 and 6). This may represent phytate (or a partially dephosphorylated inositol polyphosphate) in a standby mode between cycles of catalysis. A similar binding mode has been observed in a phytase of the protein tyrosine phosphatase class (Chu et al., 2004).

### A model for phytate recognition by wheat PAPhy

In an alternative approach to identify substrate specificity pockets, a molecular dynamics (MD) simulation of a modeled TaPAPhy_b2:phytate complex was performed at pH 5.5 and 298 K. Phytate was manually docked into the active site of the enzyme, superimposing the D-4 phosphate group of phytate onto the bound inorganic phosphate molecule of the TaPAPhy_b2 substrate complex. A 100-ns MD simulation was then performed to allow the substrate to sample conformational space within the active site. Geometric clustering was performed to identify similar structures sampled during the trajectory. The central member of the cluster with the highest population (representing 91% of the total) was taken to represent the productive enzyme-substrate complex that allowed a network of protein-substrate interactions to be defined (Figure 3C). To simplify the description of the interactions, we propose a nomenclature for the six specificity pockets. In this scheme the pocket responsible for binding the scissile phosphate is named A. With the D-4 phosphate group in pocket A and orienting the axial D-2 phosphate group toward the viewer, the remaining specificity pockets are sequentially named B–F in an anticlockwise fashion following the order of increasing phosphate number attached to the inositol ring. Using this nomenclature, the PAPhy 4 motif contributes to pockets D and F, while residues of the PAPhy 5 motif contribute to pocket E. Of the former, His229 is found in pocket F contacting phosphate D-3, while the D-1 phosphate contacts Ser219 and the PAPhy 4 α-helix macrodipole. The short helical turn consisting of PAPhy 5 residues Ala431–Met433 contacts the 2-phosphate, mainly through interactions with its main chain. Residue Lys410 contacts phosphates D-5 and D-6 in pockets B and C, respectively. While the amino acid pair Glu409-Lys410 is highly conserved in PAPhys, it is predominantly encountered as Glu–Gly in PAPs. The presence of PAPhy motifs 4 and 5 and incorporation of a lysine residue at position 410 may therefore constitute the major requirements for specific phytase activity. The remainder of the contacts to phytate in pockets A and F are provided by residues conserved across HMW PAPs and PAPhys and so presumably contribute toward the broader phospho-substrate specificity of the family and thus do not contribute explicitly to specificity toward phytate.

### Mutagenesis identifies active site elements with central roles in phytase activity

**Figure 4:**
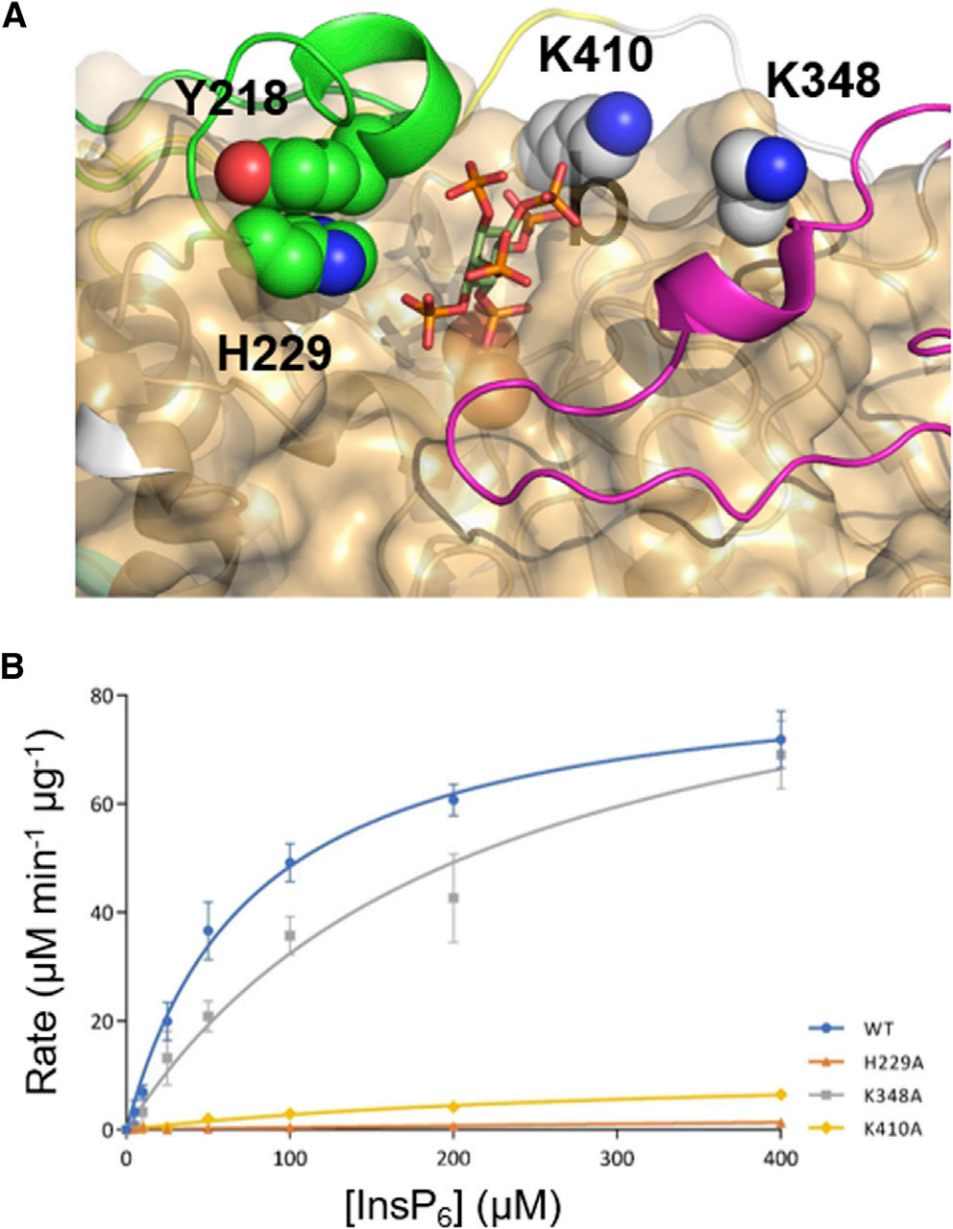
Site-directed mutagenesis of active site residues suggests central roles for PAPhy 4 and K410 in phytase activity. **(A)** A close-up view of the active site of TaPAPhy_b2. Polypeptide chain colored as follows: metallophosphoesterase (MPE) domain, gray; PAPhy 4 motif, green; PAPhy 5 motif, light magenta. Residues selected for mutagenesis are shown in sphere format and labeled (also shown is Y218, which forms a π-stacking interaction with H229). The coloration of the residues follows their assignment to either the MPE domain (K348 and K410) or the PAPhy 4 motif (H229 and Y218). The structure is overlaid with the molecular surface of red kidney bean PAP (gold color; PDB: 2QFR) and shows phytate in stick format in its predicted location from the 100-ns MD trajectory. The binuclear center is visible at the base of the TaPAPhy_b2 active site. **(B)** Michaelis-Menten kinetics of WT TaPAPhy_b2 and active site mutants H229A, K348A, and K410A. Error bars represent the standard deviations of triplicate measurements.

To validate our assignment of specificity pocket contents in TaPAPhy_b2, we turned to site-directed mutagenesis of predicted active site residues. Basic residues were chosen for alanine mutagenesis if they were found less than 6 Å from the predicted position of the bound substrate and were conserved in PAPhy but not in the non-phytase HMW PAPs. This process identified two residues highlighted from analysis of the MD trajectory, His229 (Pocket F) and Lys410 (pockets B and C), and one other, K348 (Figure 4A). The side chain of K348 forms direct hydrogen bonding contacts with residues of the PAPhy 5 motif and may have an indirect influence on phytase activity. The sequential degradation of phytate by the mutants as followed by HPLC and the pH-dependence of their phytase activities were indistinguishable from that observed for the wild-type enzyme (Supplemental Figures 7–9). The relative phytase activities of the mutants were <5% for H229A and 13% for K410A (Figure 4B). Kinetic parameters can be found in Supplemental Table 3. The rate constant for K348A was not significantly different from that of the wild-type enzyme. Mutation of K410 therefore significantly reduced phytase activity consistent with a central role of this residue as a major determinant of specificity toward phytate as previously predicted (Feder et al., 2020). That the phosphatase activity of this mutant toward an alternative substrate, *p*-nitrophenyl phosphate, is not significantly reduced from that of the wild-type enzyme is in keeping with the view that K410 is required to solvate the charges on the phosphates of bound phytic acid occupying pockets B and C (Supplemental Figure 10). The K348A mutant shows only minor change in phytase activity. On the other hand, the H229A mutant showed highly attenuated activity toward phytate. Crystallization of this mutant allowed its structure to be solved at 1.50 Å resolution, which proved it to be essentially identical to that of the wild-type enzyme. However, a region of discontinuous electron density was identified between residues Asp216 and Pro227, covering most of the PAPhy 4 motif. The lack of structural order in this region of the mutant enzyme can be explained by the deletion of a π-stacking interaction between His229 and Tyr218 following mutagenesis. Such interruption presumably results in instability of the PAPhy 4 motif, emphasizing the importance of this motif in phytate binding and recognition.

### Specificity pocket composition is conserved in other common cereal phytases

To explore variation in the amino acid composition of the predicted phytate specificity subsites among plant PAPhys and its relationship to the positional specificity of phytate hydrolysis, we turned our attention to the closely related enzymes from barley (HvPAPhy_a), rice (OsPAPhy_b), maize (ZmPAPhy_b), and soybean (GmPAPhy_b). Amino acid sequence identities relative to TaPAPhy_b2 range from 72% (GmPAPhy_b) to 91% (HvPAPhy_a). Comparative modeling was used to predict the structures of the four enzymes, and the sequence variabilities at 33 active site residue positions falling within 10 Å of the bound phosphate group in the crystal structure of TaPAPhy_b2 were assessed (Supplemental Figure 11, Supplemental Table 4). The active site residues of the cereal enzymes HvPAPhy_a, OsPAPhy_b, and ZmPAPhy_b vary from the TaPAPhy_b2 sequence at 42%, 27%, and 36% of residue positions, respectively. Notably, 67% of residues vary between TaPAPhy_b2 and GmPAPhy_b. In contrast, the conservation of residues in PAPhy motifs 4 and 5 that are predicted to be in contact with the substrate is high. Nevertheless, notable differences are seen in the E specificity pocket in the PAPhy 5 motif, involving residues Ala431 (proline in OsPAPhy_b, ZmPAPhy_b and GmPAPhy_b), Phe432 (tyrosine in GmPAPhy_b), and Met433 (isoleucine in HvPAPhy_a). Despite this, our modeling suggests the contribution of these residues to the binding of phytate is through their main chain amino groups rather than their side chains, and as such, these residue changes are not expected to influence the positional specificity of phytate hydrolysis.

The recombinant plant enzymes were prepared by heterologous expression using the KM71H::OCH1 glycoengineered strain of *P. pastoris* in the same manner as for TaPAPhy_b2. The purified enzymes were assayed for phytase activity under standard conditions at a range of enzyme concentrations except for GmPAPhy_b for which sufficient sample was obtained to permit assay at only a single unique concentration (Supplemental Figure 12). The resulting order of specific phosphate release activities was TaPAPhy_b2 > HvPAPhy_a > ZmPAPhy_b > OsPAPhy_b >> GmPAPhy_b. Insufficient soybean enzyme was available so HPLC profiles of inositol polyphosphates resulting from InsP_6_ degradation by only the recombinant cereal PAPhy enzymes were recorded (Supplemental Figures 13–16). Identical profiles of hydrolysis intermediates were obtained in reactions performed with all cereal enzymes tested, confirming a conserved D-4 and/or D-6 phytase activity. However, as the method cannot resolve the enantiomers D-Ins(1,2,3,5,6)P_5_ and D-Ins(1,2,3,4,5)P_5_, their absolute specificities remain unresolved. Hence, while the residue changes observed in the active sites of the cereal enzymes studied do not appear to alter the positional specificity of phytate hydrolysis, substitutions in the specificity pockets, particularly specificity pocket E, may serve to modulate specific phytase activity.

## Discussion

Cereals and legumes form a significant component of the food supply for humans and other animals, and constitute a major source of dietary carbohydrate, protein, lipids and minerals. Phytate is the major storage form of phosphorus in mature grains and legumes, contributing 60%–80% of the total (Viveros et al., 2000; Humer et al., 2015). Hydrolysis of phytate is catalyzed by phytases to yield bioavailable orthophosphate, and high native phytase activities are present in cereals and cereal by-products (Madsen et al., 2013; Brinch-Pedersen et al., 2014). Phytase activity usually increases on germination, and germination has historically been used to induce this activity in cereals. Crystal structures of plant PAPs, enzymes with broad specificity for phospho-substrates, have been available since 1995 (Sträter et al., 1995). However, these structures have failed to explain how specificity for phytate could be achieved by the closely related PAPhy. The high-resolution crystal structures of the wheat purple acid phytase isoform b2 reported herein provide a straightforward explanation by highlighting the roles of two sequence inserts (PAPhy motifs) relative to a canonical MPE domain in forming phytate-specific substrate specificity pockets. In this way, PAPhy motifs 4 and 5 serve to neofunctionalize a plant HMW PAP domain, endowing it with hydrolytic specificity toward phytate. Building on this, amino acid sequence analysis neatly explains the highly specific phytase activity observed in monocot cereals.

Our attempts to use a non-hydrolyzable analog to provide details of interactions with bound phytate allowed us instead to identify a possible standby mode of substrate binding. We therefore turned to MD simulation. MD simulations have a become primary tool used to investigate the action of biological macromolecules. Based on high-resolution structures of enzymes, MD simulations can be used to gain detailed insights to substrate binding and catalysis (Koch et al., 2013; Chen et al., 2015; Iqbal and Shah, 2018). Clustering of states from a 100-ns MD simulation of TaPAPhy_b2 have allowed the identification of a dominant bound conformation for phytate. This binding mode is stabilized by interaction of phytate with residues of PAPhy motif 4 in specificity subsites D and F, and with residues of PAPhy motif 5 in subsite E. Furthermore, residue K410 was identified as a central player in sensing phosphate groups of substrate in specificity pockets B and C. Despite substantial numbers of amino acid variations between the active sites of the plant PAPhys considered, all the cereal PAPhys tested generated the same phytate degradation profile, regardless of the plant species or the enzyme isoform. However, the phytase activities of the cereal enzymes (Viveros et al., 2000; Steiner et al., 2007; Dionisio et al., 2011) vary considerably, suggesting that mining of amino acid sequence data together with crystal structure and activity data may be a profitable route to identify substitutions leading to enhanced cereal mature grain phytase activity.

As phytase activity in food and feedstuffs is an important nutritional parameter, our structural data offers direction to manipulation of phytase activity *in planta* with implications for the development of crops with engineered inositol polyphosphate content or enhanced mature grain phytase activity.

## Methods

### Sequence analysis

The amino acid sequences of known PAPhy were collected and compared with those of PAPs demonstrated to lack phytase activity to determine key differences in addition to those described previously (Dionisio et al., 2011). A total of 124 PAP sequences were analyzed (Supplemental Table 5), of which 112 were collected from the UniProt database (The UniProt Consortium, 2017) and the remaining 12 were retrieved from Phytozome version 12.0 (Goodstein et al., 2012) or BLASTP (Altschul and Gish, 1996) searches following the methods described by Rivera-Solís et al. (Rivera-Solís et al., 2014). A multiple sequence alignment of the PAPhy and PAP sequences was performed using the MUSCLE algorithm (Edgar, 2004) with default parameters and analyzed with Jalview (Waterhouse et al., 2009). A phylogenetic analysis of the PAP sequences was performed with MEGA7 (Kumar et al., 2016), and a phylogenetic tree was constructed using the maximum likelihood method with default parameters.

### Production of the *OCH1::G418R* hyperglycosylation knockout of the *Pichia pastoris* KM71H strain

*P. pastoris* strain KM71H was chosen since it is Mut^S^, a phenotype of slow methanol utilization. The abolition of hyperglycosylation will render homogeneous the type of glycosylation to an average of Man_8-14_GlcNAc_2_ (Bretthauer and Castellino, 1999; Jacobs et al., 2009) better compatible with subsequent deglycosylation and crystallization. For this purpose, a knockout construct was generated of the ORF of the gene *OCH1*, encoding a mannosyltransferase of the *cis*-Golgi apparatus (XM_002489551, PAS_chr1-3_0251). PCR using the *OCH1* cloning primers was used to verify the correct gene substitution: 1299 bp was the PCR product for the escape transient expression and 2591 bp for the correct knockout integration product.

### Preparation of recombinant TaPAPhy_b2 samples

Recombinant TaPAPhy_b2 in fusion with an N-terminal peptide encoding the *Saccharomyces cerevisiae* α-factor secretion signal and a C-terminal 6xHis affinity tag was produced from a pGAPZαA (Invitrogen) construct (Dionisio et al., 2011). This construct uses the promoter of the glyceraldehyde-3-phosphate dehydrogenase enzyme to drive the constitutive production of extracellular TaPAPhy_b2 protein in *P. pastoris*. A 20-amino acid signal peptide and a C-terminal seven-amino acid ER-retention signal was excluded from the construct. TaPAPhy_b2 was obtained by growing a *P. pastoris* KM71H (*OCH1::G418R*) transformant with TaPAPhy_b2-pGAPZαA in 800 ml of buffered minimal glucose medium for 5 days under continuous shaking (200 rpm) at 26°C. The resulting supernatant was concentrated to 50 ml using a stirred cell (Amicon) with a regenerated cellulose ultrafiltration membrane (10 kDa NMWL; Merck).

Recombinant His-tagged protein was purified by metal affinity chromatography and deglycosylated at 4°C overnight in 1x GlycoBuffer 3 (50 mM sodium acetate, pH 6.0; NEB) with 100 000 U mg^−1^ recombinant GST-Endo F1 produced as described by Grueninger-Leitch et al. (Grueninger-Leitch et al., 1996) (Supplemental Figure 17). Deglycosylated protein (TaPAPhy_b2d) was purified by glutathione affinity chromatography followed by size exclusion chromatography. TaPAPhy_b2d was subsequently concentrated and dialyzed against 20 mM Tris–HCl (pH 8.0) for analysis. Single-site mutants H229A, K348A, and K410A were generated using a modified version of the QuickChange site-directed mutagenesis method (Liu and Naismith, 2008). The transformation, expression, and purification of the mutants were performed as for the wild-type enzyme.

### Preparation of recombinant plant PAPhys

Barley (HvPAPhy_a), rice (OsPAPhy_b), and maize (ZmPAPhy_b) PAPhy genes cloned in the vector pPICZαA (Dionisio et al., 2011) were used in this study. A synthetic gene for the soybean enzyme GmPAPhy_b was cloned into the Gateway entry vector pDONR207 and then transferred to the destination vector pPICZα-DEST (Sasagawa et al., 2011). The transformation and expression of the four cereal PAPhy-pPICZα constructs was performed essentially identically as for the TaPAPhy_b2 site mutants and utilized the KM71H (OCH1:G418R) *P. pastoris* glycoengineered strain. A preference for manganese in the MII site has been described for PAPhy_a isoforms (Dionisio et al., 2011), so for the expression of the barley a-isoform, HvPAPhy_a, 100 μM manganese(II) sulfate was also added to the buffered minimal methanol medium. The enzymes were purified by nickel-affinity chromatography and stored in 20 mM Tris–HCl (pH 8.0) buffer containing 30% (v/v) glycerol at –80°C.

### Phosphate release assays

Enzymatic characterization was performed with purified glycosylated proteins after nickel-affinity chromatography purification by means of standard phosphate release assays (Nagul et al., 2015) in 0.2 M acetate (pH 5.5) buffer with 5 mM potassium phytate (≥95% purity, Sigma). Absorbance at λ = 700 nm was subsequently measured in a microplate reader (Hidex Sense) after color development for 30 min.

### HPLC separation of products of enzymatic phytate hydrolysis

The product profiles of reaction of all wild-type and mutant cereal PAPhys with InsP_6_ were obtained by separating the inositol phosphate products on HPLC after the method of Blaabjerg et al. (Blaabjerg et al., 2010).

### X-ray crystal structure determination

Crystallization was performed using the sitting drop vapor diffusion method at 16°C with protein concentrated to 7–8 mg ml^−1^. Crystals in space group *H*3 grew in drops containing 0.2 M sodium thiocyanate and 20% (w/v) PEG 3350, and they were cryoprotected by addition of 25% (v/v) PEG 400. To obtain the crystal structure of the TaPAPhy_b2:InsS_6_ complex, crystals were soaked for 4 min in a solution of 5 mM *myo*-inositol hexakissulfate (InsS_6_) at pH 5.5 adjusted with acetate buffer. X-ray data was collected at Diamond Light Source (Didcot, UK) on beamlines I03 and I04 at wavelengths of 0.9763 Å (12.6994 keV) for native datasets and 1.7389 Å (7.1300 keV) for datasets collected at the iron K-absorption edge. The PHENIX suite (Adams et al., 2010) was used for structure solution and refinement. The crystal structure of TaPAPhr_b2d was solved by molecular replacement (MR) using as search model the structure of red kidney bean PAP (PDB: 2QFR; Schenk et al., 2008). MR solutions were subjected to several rounds of manual remodeling using COOT (Emsley et al., 2010) followed by refinement with PHENIX REFINE (Adams et al., 2010). Crystal parameters, data collection, and refinement statistics for the TaPAPhy_b2 structures are summarized in Supplemental Table 6.

### Molecular dynamics simulations

A dynamic model of the TaPAPhy_b2:InsP_6_ complex was obtained through molecular modeling and MD simulation. This approach utilized a modified version of the crystal structure of the TaPAPhy_b2:PO_4_ complex resembling substrate binding containing a μ-(hydr)oxo bridge in the active site. Simulations were performed using the GROMACS 2020.4 molecular dynamics package (Hess et al., 2008) with the amber99sb-ildn force field (Oostenbrink et al., 2004). InsP_6_ coordinates and topology were obtained from ATB version 3.0. InsP_6_ was modeled as C_6_H_12_O_24_P_6_^6-^ at pH 5.5 according to Veiga et al. (Veiga et al., 2014). To generate starting coordinates for the complex the D-4-phosphate of phytate was manually docked to superimpose the active site phosphate found in the crystal structure. MD simulations were carried out with weak restraints applied to the position of the two iron ions, the amino acid residues coordinating the irons, the μ-oxo bridge, and the phosphate molecule coordinated to the metals. An MD simulation of 100 ns duration of the TaPAPhy_b2:InsP_6_ complex in aqueous solution was then performed at a constant temperature of 298 K. Analysis of the MD trajectory was carried out using embedded tools in the GROMACS package.

### Other software

PyMOL (Schrodinger, 2015) was used for the visualization of protein models and preparation of Figures. The APBS (Baker et al., 2001) plug-in to PyMOL was used to calculate electrostatic potential contour maps.

Full details of all methods can be found in the supplemental information.

## Funding

This work was funded by the UK Biotechnology and Biological Sciences Research Council and AB Vista Ltd. through IPA award BB/M022978/1, and by the Danish Ministry of Food, Agriculture and Fisheries (grant no. 3304-FVFP-08-M-07-01).

## Author contributions

Conceptualization, A.M.H., C.A.B., G.D., and H.B.-P.; Methodology, A.M.H., C.A.B., G.D., and H.B.-P.; Investigation, R.F.R., Y.H.G., M.S., A.M.H., C.A.B.; Writing – Original Draft, R.F.R., A.M.H., C.A.B., Y.H.G., M.S.; Writing – Review & Editing, R.F.R., A.M.H., C.A.B., G.D., and H.B.-P.; Funding Acquisition, A.M.H., C.A.B.; Resources, A.M.H., C.A.B., G.D., and H.B.-P.; Supervision, A.M.H., C.A.B., G.D., and H.B.-P.

## Accession numbers

Atomic coordinates and crystallographic structure factors have been deposited in the Protein Data Bank under accession codes 6GIT (product-bound form), 6GIZ (substrate-bound form), 6GJA (H229A mutant), and 6GJ2 (complex with InsS_6_).

## Supplemental information

Document S1. Supplemental Figures 1–19 and Supplemental Tables 1–6

Document S2. Article plus supplemental information
