# Supplementary material for: Structure of a cereal purple acid phytase provides new insights to phytate degradation in plants
Source: Plant Commun. 2022 Feb 19;3(2):100305. doi: 10.1016/j.xplc.2022.100305 (PMC9073318; doi:10.1016/j.xplc.2022.100305)
Supplement: Document S1. Supplemental Figures 1–19 and Supplemental Tables 1–6 [file mmc1.pdf]

Plant Communications, Volume *3*

Supplemental information

Structure of a cereal purple acid phytase provides new insights to phytate degradation in plants

Raquel Faba-Rodriguez, Yinghong Gu, Melissa Salmon, Giuseppe Dionisio, Henrik Brinch-Pedersen, Charles A. Brearley, and Andrew M. Hemmings

SUPPLEMENTAL INFORMATION

Structure of a Cereal Purple Acid Phytase Provides New Insights to Phytate Degradation in Plants

Raquel Faba-Rodriguez[1,2], Yinghong Gu[2], Melissa Salmon[2], Giuseppe Dionisio[3], Henrik A. Brinch-Pedersen[3], Charles A. Brearley[2] and Andrew M. Hemmings[1,2,4*]

[1]School of Chemistry and [2]School of Biological Sciences, University of East Anglia, Norwich Research Park, Norwich NR4 7TJ, U.K. [3]Department of Agroecology, Research Center Flakkebjerg, Aarhus University, 4200-Slagelse, Denmark. [4]College of Food Science and Technology, Shanghai Ocean University, Shanghai 201306, China.

Running title: Crystal Structure of a Cereal Purple Acid Phytase

Contact information: a.hemmings@uea.ac.uk

CONTENTS

SUPPLEMENTAL METHODS (p.4)

LIST OF SUPPLEMENTAL TABLES (p.13)

1. Selected active site distances of the TaPAPhy_b2:PO4 complex structures.
2. Reported characteristics of plant PAPhy.
3. Estimation of kinetic parameters for $InsP_6$ hydrolysis by recombinant wheat phytase isoform b2 (TaPHY_b2) and active site mutants.
4. Purple acid phosphatase sequences used in bioinformatic analyses.
5. X-ray data collection and structure refinement statistics.
6. Comparison of active site residues of plant PAPhy.

LIST OF SUPPLEMENTAL FIGURES (p.28)

1. Anomalous difference electron density map calculated using a TaPAPhy_b2 Fe-SAD dataset collected at the iron K-edge.
2. The binuclear centre of the TaPAPhy_b2:PO4 complex in (a) product- and (b) substrate-bound states.
3. Multiple sequence alignment of PAPhys and HMW PAPs.
4. Molecular phylogenetic analysis of PAP sequences by the maximum likelihood method.
5. Inhibition of phytase activity by *myo*-inositol hexakissulfate ($InsS_6$).
6. Intermolecular interactions in the crystal structure of the TaPAPhy_b2:$InsS_6$ complex.
7. HPLC product profiles of recombinant wild type (WT) TaPAPhy_b2 and its active mutants after limited reaction against $InsP_6$.

8. HPLC product profiles of recombinant wild type (WT) TaPAPhy_b2 and its active site mutants after progressive reaction against $InsP_6$.

9. HPLC product profiles of recombinant wild type (WT) TaPAPhy_b2 and its K410A mutant after extended reaction against $InsP_6$.

10. Phytase and *p*-nitrophenyl phosphatase activities of WT TaPAPhy_b2 and its mutants.

11. Comparison of the active sites of cereal PAPhys.

12. Phytase activities of recombinant cereal PAPhy enzymes.

13. HPLC product profiles following hydrolysis of InsP6 by recombinant wheat PAPhy isoform b2 (TaPAPhy_b2).

14. HPLC product profiles following hydrolysis of InsP6 by recombinant barley PAPhy isoform a (HvPAPhy_a).

15. HPLC product profiles following hydrolysis of InsP6 by recombinant maize PAPhy isoform b (ZmPAPhy_b).

16. HPLC product profiles following hydrolysis of InsP6 by recombinant rice PAPhy isoform b (OsPAPhy_b).

17. Partial enzymatic deglycosylation of TaPAPhy_b2 using recombinant GST-Endo F1.

18. pH profile of phytase activities of recombinant wild type (WT) TaPAPhy_b2 and active site mutants.

19. Validation of metal-binding sites of TaPAPhy_b2:PO_4 structures.

SUPPLEMENTAL REFERENCES (p.67)

VALIDATION REPORTS FOR PDB ENTRIES (p. 74)

SUPPLEMENTAL METHODS

Sequence analysis. The amino acid sequences of known PAPhy were analysed and compared with those of PAPs demonstrated to lack phytase activity in order to determine key differences in addition to those described previously (Dionisio *et al.*, 2011). A total of 124 PAP sequences were analysed (Supplemental Table 4), of which 112 were collected from the UniProt database (Bateman *et al.*, 2017) and the remaining 12 were retrieved from Phytozome version 12.0 (Goodstein *et al.*, 2012) or BLASTP (Altschul and Gish, 1996) searches following the methods described by Rivera-Solís *et al.*(Rivera-Solís *et al.*, 2014). Sequence groups were created to facilitate the analysis, considering (1) reported phytase activity of the protein, (2) kingdom of life of the source organism, and (3) estimated molecular weight of the protein. In the PAPhy group, distinctions were made for characterised proteins, those predicted by sequence homology with characterised PAPhy, or sequence outliers compared to the rest of the PAPhy enzymes. A specific group was created for the microalgal PAPs, as these shared insufficient sequence conservation with the higher plant enzymes. The following numbers of sequences were contained in the resulting eight groups: (1) 29 PAPhy (23.4% of the total), of which 14 are characterised and 15 predicted; (2) 42 HMW plant PAPs (33.9%), with 2 being PAPhy outliers; (3) 13 LMW plant PAPs (10.5%); (4) 10 HMW animal PAPs (8.1%); (5) 10 LMW animal PAPs (8.1%); (6) 12 microalgal PAPs (9.7%); (7) 2 fungal PAPs (1.6%); and (8) 6 bacterial PAPs (4.8%). A multiple sequence alignment (MSA) of the PAPhy and PAP sequences was performed using the MUSCLE algorithm (Edgar, 2004) with default parameters and analysed with Jalview (Waterhouse *et al.*, 2009). A phylogenetic analysis of the PAP sequences was performed with MEGA7 (Kumar *et al.*, 2016), and a phylogenetic tree constructed using the Maximum Likelihood method with default parameters.

Production of a *OCH1::G418R* hyperglycosylation knockout of the *Pichia pastoris* KM71H strain. *Pichia pastoris* strain KM71H was chosen since it is Mut^s a phenotype of slow methanol utilization. To produce recombinant protein better compatible with subsequent deglycosylation and crystallization, a knockout construct was generated of the ORF of the gene *OCH1*, encoding a mannosyltransferase of the

cis-Golgi apparatus (XM_002489551, PAS_chr1-3_0251). The objective was that abolition of hyperglycosylation would render homogeneous the glycosylation of the recombinant product to an average of $Man_{8-14}GlcNAc_2$ (Bretthauer and Castellino, 1999; Jacobs *et al.*, 2009). The ORF was cloned using primers Pp_E12456 och1_fw 5´-TCGTCAACTATGGCGAAGGC-3´ and Pp_E12456 och1_rv 5´-TATGATGACGGACGATCGCTTA-3´. The PCR product (1299 bp) was cloned into pCR4-blunt-Topo vector (Life Technology). The resulting vector, called pCR4-OCH1, was restricted with *Bst*BI and *Sfi*I and a USER™ linker cassette (Pac_I_Bst_BI_overhang_up 5´-CGAAGCTGAGGCTTAATTAAACCTCAGCGGCCACTC-3´ and Pac_I_Sfi_I_overhang_dwn 5´-TGGCCGCTGAGGTTTAATTAAGCCTCAGCTT-3´) was introduced and ligated into it (pCR4-OCH1_Delta).

The USER™ insert was chosen to be the kanamycin resistance gene (*G418R*) obtained by PCR from the vector pKAN B alpha (Lin-Cereghino *et al.*, 2008) for which PCR was performed with the pfuCx DNA Polymerase (Stratagene) and primers G418_KAN_fw 5´-GGCTTAAUCAACTCCGAACGACCTGC-3´ G418_KAN_rv 5´-GGTTTAAUCGAGTTAGCCCTCCCACA-3´. The vector pCR4-OCH1_Delta was digested with *Pac*I and *Nt.BbvC*I enzymes and combined with the USER™ enzyme mix (New England Biolab) and treated *G418R* PCR product (1703 bp). The resulting vector pCR4-OCH1::G418R was used as template for the PCR reaction using M13 reverse and forward primers in order to get the knockout linear fragment to transform *Pichia*. *Pichia* transformation was performed according to Lin-Cereghino *et al*. (Lin-Cereghino *et al.*, 2005). Positive *OCH1::G418R* knockout colonies were checked over four rounds of plating out of single colonies with dilution in order to get rid of transient expression of the *G418R* gene. Furthermore, PCR using the *OCH1* cloning primers was used to verify the correct gene substitution: 1,299 bp was the PCR product for the escape transient expression and 2,591 bp for the correct knockout integration product.

Preparation of recombinant TaPAPhy_b2 protein samples. Recombinant TaPAPhy_b2 in fusion with an N-terminal peptide encoding the *Saccharomyces cerevisiae* α-factor secretion signal was produced from a pGAPZαA (Invitrogen) construct. This construct uses the promoter of the glyceraldehyde-3-phosphate dehydrogenase enzyme to drive the constitutive production of extracellular TaPAPhy_b2 protein in *P.pastoris*. A twenty-amino acid signal peptide and a

C-terminal seven amino acid ER-retention signal was excluded from the construct and a C-terminal 6xHis tag added. 10 μg of the construct was linearized with *Avr*II (NEB) and used for electroporation (1.8 kV, 25 μF, 200 Ω) of the engineered *P. pastoris* KM71H (*OCH1::G418R*) strain. Cells were left to recover at 28°C overnight before plating on yeast extract peptone dextrose (YPD) solid medium with 400 μg mL^{-1} Zeocin™. After four days of incubation at 28°C, the biggest colonies were transferred to fresh YPD solid medium with 400 μg mL^{-1} Zeocin™ and incubated for another two days at 28°C. Colonies were tested for the production of secreted protein in a small volume expression trial and the highest expressing colony selected.

Recombinant TaPAPhy_b2 for crystallization was obtained by growing the selected *P. pastoris* KM71H (*OCH1::G418R*) transformant with TaPAPhy_b2-pGAPZαA in 800 mL of buffered minimal glucose medium (1.34% w/v yeast nitrogen base, 2% w/v casamino acids, 2% w/v glucose, 100 mM phosphate buffer pH 5.0, 100 μg mL^{-1} kanamycin, 100 μM iron(II) sulfate, 100 μM iron(III) citrate) for five days under continuous shaking (200 rpm) at 26°C. 100 μM iron(II) sulfate and 100 μM iron(III) citrate were added to the cultures on the first two days of expression, while 200 μM iron(II) sulfate, 200 μM iron(III) citrate, 2% w/v glucose and 0.5% w/v casamino acids were added on the third day. The protein was purified from the culture media after centrifugation (11,900 x g, 20 min at 4°C) to separate the cells, followed by adjusting the pH to 8.0 with 10 M NaOH and further centrifugation to separate precipitated phosphate salts. The resulting supernatant was concentrated below 50 mL using a stirred cell (Amicon) with a regenerated cellulose ultrafiltration membrane (10 kDa NMWL; Merck) and dialysed against binding buffer for nickel-nitrilotriacetic acid (Ni-NTA) metal-affinity chromatography (50 mM Tris-HCl pH 8.0, 500 mM NaCl, 20 mM imidazole) using 3.5 kDa MWCO Spectra/Por dialysis tubing (Spectrum Labs). A 5 mL Ni-NTA Superflow cartridge (Qiagen) was used to perform metal-affinity chromatography in an ÄKTA pure chromatography system (GE Healthcare). The recombinant protein was eluted with a gradient of imidazole (20 mM-500 mM), concentrated and dialysed with a 10 kDa MWCO centrifugal filter (Merck) against 20 mM Tris-HCl pH 8.0 and deglycosylated at 4°C overnight in 1x GlycoBuffer 3 (50 mM sodium acetate pH 6.0; NEB) with 100,000 U mg^{-1} recombinant GST-Endo F1 (produced as described by Grueninger-Leitch *et al.* (Grueninger-Leitch *et al.*, 1996); Supplemental Figure 17). GST-Endo F1 was removed using a 1 mL GSTrap 4B

cartridge (GE Healthcare) and the resulting deglycosylated protein (TaPAPhy_b2d) was gel filtered through a HiLoad 16/600 Superdex 75 pg column (GE Healthcare) with a buffer containing 20 mM Tris-HCl pH 8.0 and 250 mM NaCl. TaPAPhy_b2d was concentrated and dialysed in 20 mM Tris-HCl pH 8.0 after purification. The concentration of recombinant TaPAPhy_b2 was calculated by absorbance measurement at a wavelength of 280 nm using a extinction coefficient of 113,680 $M^{-1}cm^{-1}$, calculated with the ExPaSy ProtParam online resource (Wilkins *et al.*, 1999) for the non-glycosylated protein (MW = 57.49 kDa).

Single site mutants H229A, K348A and K410A were generated using a modified version of the QuickChange™ site-directed mutagenesis method (Liu and Naismith, 2008). Mutants were verified by sequencing. The transformation, expression and purification of the mutants were performed as for the wild type enzyme.

Production of other recombinant plant PAPhys using *Pichia pastoris*. The transformation, expression and purification of HvPAPhy_a, OsPAPhy_b, ZmPAPhy_b and GmPAPhy_b was performed as for the TaPAPhy_b2 enzyme and its three mutants. The four PAPhy-pPICZα constructs linearized with DraI (NEB) were transformed into the KM71H (OCH1::G418R) *Pichia pastoris* glycoengineered strain through electroporation. Expression of the plant PAPhy enzymes was performed in 100 mL of buffered minimal glycerol/methanol medium, distributed in 250 mL conical flasks with 50 mL per flaks, for five days under continuous shaking (200 rpm) at 26°C, adding 1% (v/v) methanol and the appropriate metals daily. For the expression of the PAPhy_a isoform HvPAPhy_a, 100 µM manganese(II) sulfate and Complete Mini EDTA-free Protease inhibitor cocktail tablets (Roche) were also added to the buffered minimal methanol medium. The enzymes were harvested, purified by nickel-affinity chromatography and concentrated in the same way as the TaPAPhy_b2 using 1 mL HisTrap HP columns (GE Healthcare) at a flow rate of 1 mL min^{-1}. The nickel-affinity purified plant PAPhy enzymes were normalised to a working concentration of 20 µM and stored in 20 mM tris/HCl pH 8.0 buffer containing 30% (v/v) glycerol at -80°C.

Phosphate release assays. Enzymatic characterisation was performed with glycosylated proteins after nickel affinity chromatography purification by means of standard phosphate release assays (Nagul *et al.*, 2015) in 0.2 M acetate pH 5.5 buffer with 5 mM potassium phytate (≥ 95% purity, Sigma). The recombinant proteins were

assayed at concentrations ranging from 60 nM to 1 μM. Reactions (50 μL) were performed in 96-well plates for 15 min at room temperature with two to four replicates per condition, depending on the experiment layout. Standard curves for each assay were prepared with monopotassium phosphate. Enzyme-free buffer with $InsP_6$ and buffer only reactions were utilized to determine background absorbance of small levels of contaminant inorganic phosphate present in the $InsP_6$ substrate. The reactions were stopped with 50 μL of a colour reagent, containing four volumes of 1.5% w/v ammonium molybdate in a 5.5% v/v sulfuric acid solution and one volume of a 10.8% w/v iron(II) sulfate solution. Absorbance at λ = 700 nm was measured in a microplate reader (Hidex Sense) after colour development for 30 min.

For relative activity calculations of the mutant enzymes compared to the wild type, the enzymes were assayed at concentrations of 1 μM-100 nM and reactions were carried out with four replicates. For determination of pH optimum (Supplemental Figure 18), the following buffers were used: pH 2.0 to 3.5, 0.2 M glycine/HCL; pH 4.0 to 5.5, 0.2 M sodium acetate; pH 6.0 to 7.0, 0.2 M bis-Tris; and pH 7.5 to 8.5, 0.2 M Tris-HCl. Reactions were carried out in duplicate.

The effect of the non-hydrolysable $InsP_6$ analogue *myo*-inositol hexakissulfate ($InsS_6$, potassium salt; Alfa Chemistry) on the phytase activity of wild type TaPAPhy_b2 was tested with the standard phosphate release assay conditions described above, with 5 mM $InsP_6$ substrate and 1 μM enzyme. Reactions were performed in triplicate in the presence of increasing concentrations of $InsS_6$ (0 to 1 mM).

Kinetic parameters were estimated at the pH and temperature optima with sodium phytate (≥98% purity, Merck) as substrate and reactions in triplicate. A single timepoint (10 min for WT and K348A or 90 min for H229A and K410A) and enzyme concentration (60 nM) were chosen on the basis that, when less than 10-15% of the total substrate for each substrate concentration has been consumed during the reaction, the rate of reaction obtained can be assumed to be the initial rate. The substrate concentrations used to calculate the kinetic parameters for phytate were 0, 5, 10, 25, 50, 100, 200 and 400 μM.

Phosphate release was quantified by interpolation from linear least-squares regressions of plots of absorbance against monopotassium phosphate standards. Raw absorbance data were processed in Microsoft Excel after subtraction of

absorbances arising from $InsP_6$ and free phosphate in the $InsP_6$ substrate. In order to avoid negative values at low substrate concentrations, the data was transformed to increments of phosphate concentration released with respect to the points with 0 μM substrate. The results for each reaction were expressed as the rate of phosphate concentration released (μM) per time of the reaction (min) and amount of enzyme (0.173 μg). To estimate enzyme kinetic parameters, the data was fitted to the Michaelis-Menten equation (substrate *vs.* velocity) by performing non-linear regression with the least squares (ordinary) fit method using GraphPad Prism version 7.03 (GraphPad Software, La Jolla California USA).

HPLC separation of products of enzymatic phytate hydrolysis. Reactions were performed at room temperature in 0.2 M acetate buffer pH 5.5 with 1 μM enzyme and 1 mM sodium phytate (≥98% purity, Merck) as substrate. Reactions were stopped after 30 min by boiling at 100°C for 5 min. Reaction products were resolved by anion-exchange HPLC on a 250 x 3 mm CarboPac PA200 column (Dionex UK, Ltd) and a 50 x 3 mm guard column of the same material, injecting 20 μL of reaction per run. The elution was performed at a flow rate of 0.4 mL min^{-1} with a gradient of methanesulfonic acid delivered from solvent reservoirs containing (A) water and (B) 600 mM methane sulfonic acid according to the following programme: time (min), % B; 0, 0; 25, 100; 38, 100. The separated inositol phosphates were mixed post-column with a solution consisting of 0.1% w/v ferric nitrate in 2% w/v perchloric acid at a flow rate of 0.2 mL min^{-1} for their detection by UV absorbance at λ = 290 nm (Phillippy and Bland, 1988). Inositol phosphate standards were prepared by reflux in 1 M HCl for 24 h with subsequent rotary evaporation at 35°C to remove the HCl.

Crystallization. Crystal growth was performed at 16°C with TaPAPhy_b2d concentrated to 7-8 mg mL^{-1}. Sitting drops of total volume 0.5 μL containing the protein and reservoir solution in a 1:1 ratio in 96-well 2-drop MRC crystallisation plates (Molecular Dimensions) were equilibrated against 50 μL of reservoir solution and using a OryxNano protein crystallisation robot (Douglas Instruments). Crystals formed in drops containing 0.2 M sodium thiocyanate and 20% (w/v) PEG 3350. Single crystals were cryoprotected prior to storage in liquid nitrogen by brief soaking in a cryoprotectant solution containing 0.2 M sodium thiocyanate, 20% (w/v) PEG 3350 and 25% (v/v) PEG 400. In order to obtain the crystal structure of the

TaPAPhy_b2:$InsS_6$ complex, crystals were soaked for 4 minutes in the same cryoprotectant solution but also containing 5 mM *myo*-inositol hexakissulfate ($InsS_6$) at pH 5.5 adjusted with acetate buffer.

X-ray diffraction data collection and structure determination. X-ray data was collected at Diamond Light Source (DLS; Didcot, UK) on beamlines I03 and I04. Single-wavelength X-ray diffraction data collection was performed at a wavelength of 0.9763 Å (12.6994 keV) for native datasets and 1.7389 Å (7.1300 keV) for datasets collected at the iron edge. The 1.42 Å and 1.54 Å resolution TaPAPhy_b2:PO_4 complex datasets were collected on beamline I04 from crystals in the space group *H*3. The 1.68 Å resolution TaPAPhy_b2:$InsS_6$ complex and the 1.50 Å resolution TaPAPhy_b2-H229A datasets were collected from crystals in the same space group on beamline I03. The X-ray diffraction images collected from single crystals were scaled and integrated using the DLS automated software pipeline. Data reduction was performed with XIA2 (Winter, Lobley and Prince, 2013). The PHENIX suite (Adams *et al.*, 2010) was used for structure solution. A molecular replacement (MR) search model was generated with SCULPTOR (Bunkóczi and Read, 2011) based on the crystal structure of red kidney bean PAP (PDB ID: 2QFR (Schenk *et al.*, 2008)). The structures were solved by automated MR using PHASER (McCoy *et al.*, 2007). The MR solutions were subjected to several rounds of automatic refinement using PHENIX REFINE(Adams *et al.*, 2010) and manual refinement using COOT (Emsley *et al.*, 2010). All atoms except water were considered anisotropic in the final stages of refinement. Ligand restraints were generated with READYSET or REEL (Adams *et al.*, 2010). Metal coordination restraints were also generated with READYSET (Adams *et al.*, 2010) and included in the refinement for structures with a resolution lower than 1.60 Å. Crystal parameters, data collection and refinement statistics for the TaPAPhy_b2 structures are summarised in Supplemental Table 5. Validation of refined metal-binding sites was carried out using the CheckMyMetal web server (Zheng *et al.*, 2014) (Supplemental Figure 19). X-ray fluorescence spectra were routinely collected in order to confirm the identities of transition metals in the crystal. This was followed by X-ray absorption edge scans to test for the presence of specific metals (Fe, Mn) before collecting an Fe-SAD dataset. MR was carried out as described above, an anomalous difference electron density map was generated using tools from the PHENIX suite (Adams *et al.*, 2010) and inspected in COOT (Emsley *et al.*, 2010).

Molecular Dynamics simulations. A model of the TaPAPhy_b2:InsP$_6$ complex was obtained through molecular modelling and MD simulations. This approach utilized a modified version of the crystal structure of the TaPAPhy_b2:PO$_4$ complex resembling substrate binding containing a μ-(hydr)oxo bridge in the active site. Processing of the structure prior to the MD simulations was performed in COOT (Emsley *et al.*, 2010). Residues with side chains in alternate conformations were simplified to retain the conformation with the highest refined occupancy. The conformation of unresolved residues Asp20-Arg21-Gly22 was modelled using the MODLOOP (Fiser and Sali, 2003) and missing side chains of residues Arg11, Arg18, Glu19 and Lys224 were added as the most common rotamer for each amino acid. Solvent molecules were eliminated, retaining only the μ-(hydr)oxo bridge bound to the metals. Only one N-acetylglucosamine (NAG) molecule (i.e. the one directly bound to the protein through asparagine residues) was retained for each N-glycosylation site in order to simplify the simulation model. The simulations were performed using the GROMACS 2020.4 molecular dynamics package(Hess *et al.*, 2008) with the amber99sb-ildn force field (Oostenbrink *et al.*, 2004). The metal ions in the MI and MII sites were modelled as Fe^{3+} and Fe^{2+}, respectively, and the bridging solvent molecule modelled as a μ-oxo bridge. The metal ligand Tyr204 was modelled as a negatively charged tyrosinate residue. NAG coordinates and topology were obtained from the Automated Topology Builder (ATB) version 3.0 (Koziara *et al.*, 2014). The protonation states of histidine and aspartate residues were selected with reference to the H++ server (Gordon *et al.*, 2005). The protonation state of glutamate residues was assigned automatically. InsP$_6$ coordinates and topology were also obtained from ATB version 3.0. InsP$_6$ was modelled as $C_6H_{12}O_{24}P_6^{6-}$ at pH 5.5 according to Veiga *et al.* (Veiga *et al.*, 2014). MD simulations were carried out with restraints applied to the position of the two iron ions, the amino acid residues coordinating the irons, the μ-oxo bridge and the phosphate molecule coordinated to the metals. To generate starting coordinates for the complex the D-4-phosphate of phytate was manually docked to superimpose the active site phosphate found in the crystal structure and the remainder of the molecule rotated so as to avoid short van der Waals contacts with residues in the active site cavity. Only one orientation of the substrate was possible without violation of these non-bonded distance constraints. MD simulations in aqueous solution were then performed at a constant temperature of 298 K in a cubic box with

10 Å distance from the centre of the protein to the edge of the box. The box was solvated by the Simple Point Charge (SPC) water model, adding sodium counter ions to ensure neutral charge of the system. Prior to the unrestrained MD simulations, the systems were subjected to a maximum of 10,000 steps of energy minimisation using the steepest descent method followed by 20 ps of position-restrained MD in the NVT ensemble with force constants of 1,000 kJ mol^{-1} nm^{-2} on all protein atoms in order to equilibrate the water molecules in the solvation box. The equilibrated system was then subjected to a short production MD run of 100 ns duration. The position of the scissile D-4-phosphate group was weakly restrained during the simulation by imposing a restraining force constant 1,000 kJ mol^{-1} nm^{-2} on non-hydrogen atoms. Analysis of the MD trajectory was carried out using embedded tools in the GROMACS package. Root mean square deviation (RMSD) and root mean square fluctuations (RMSF) of the Cα atoms were calculated with the original model as a reference. A selection of atoms was made to represent the active site of the enzyme. This included all non-hydrogen atoms of the substrate and of all residues with an atom falling within 6 Å of any substrate atom. Clustering (Daura *et al.*, 1999) was performed in Gromacs using this atom selection with a cutoff of 1.0 Å revealing a total of 5 conformation clusters. The central member of the cluster with the highest population (representing 91% of the total) was taken to represent the productive enzyme-substrate complex. Minimum distances of key residues or regions of the protein to neighbouring phosphate groups of phytate were monitored during the production MD runs.

Other software. PyMOL (Schrodinger LLC, 2015) was used for the visualization of protein models and preparation of Figures. The APBS (Baker *et al.*, 2001) plug-in to PyMOL was used to calculate electrostatic potential contour maps.

SUPPLEMENTAL TABLES

Supplemental Table 1. Selected active site distances of the TaPAPhy_b2:PO_4 complex structures. Distances expressed in Å. Columns headed Product and Substrate refer to distances in the product (PDB entry 6GIT) and substrate (PDB entry 6GIZ) complexes, respectively.

From	To	Product	Substrate
Fe(III)	Fe(II)	3.57	3.45
"	Asp174 Oδ2	1.79	1.89
"	Asp201 Oδ2	2.35	2.35
"	Tyr204 O^-	1.86	1.88
"	His379 Nε2	2.75	2.42
"	μ-(hydr)oxo O	n/a	2.13
"	PO_4 O1	1.49	2.27
Fe(II)	Asp201 Oδ2	2.25	2.21
"	Asn258 Oδ1	2.18	2.13
"	His340 Nε2	2.00	2.12
"	His377 Nδ1	2.08	2.13
"	μ-(hydr)oxo O	n/a	2.24
"	PO_4 O2	2.00	2.45
PO_4 O3	His350 Nε2	2.83	2.59
PO_4 O4	His295 Nε2	2.72	3.03
"	Glu409 Oε1	2.56	2.63
μ-(hydr)oxo O	PO_4 P	n/a	2.63

Supplemental Table 2. Reported characteristics of plant PAPhy.

Length, number of amino acids in transcript; MW, molecular weight; pH_{opt}/T_{opt}, pH and temperature optima; ‘np’ data not provided)

Organism	Protein	Source	Length (aa) /MW (kDa)	pH_{opt}/ T_{opt} (°C)	Oligomer state	Phytase activity	References
Rice (*Oryza sativa*)	F1	Rice bran	np/66	4.4/40	Monomer	*Km* = 170 µM	(Hayakawa, Toma and Igaue)
Rice (*Oryza sativa*)	F2	Rice bran	np/68	4.6/40	Monomer	*Km* = 90 µM	(Hayakawa, Toma and Igaue)
Rye (*Secale cereale*)	np	Germinating seed	np/67	6.0/45	Monomer	*Km* = 300 µM *kcat* = 358 s^{-1}	(Greiner, Konietzny and Jany, 1998)
Wheat (*Triticum aestivum*)	PHYI	Mature grain	np/66	np	np	np	(Nakano *et al.*, 1999)
Wheat (*Triticum aestivum*)	PHYII	Mature grain	np/68	np	np	np	(Nakano *et al.*, 1999)
Barley (*Hordeum vulgare*)	P1	Germinating seed	np/66	5.0/45	Monomer	*Km* = 72 µM *kcat* = 136 s^{-1}	(Greiner, Jany and Larsson Alminger, 2000)
Barley (*Hordeum vulgare*)	P2	Mature seed	np/66	6.0/55	Monomer	*Km* = 190 µM *kcat* = 43 s^{-1}	(Greiner, Jany and Larsson Alminger, 2000)

Soybean (*Glycine max*)	GmPhy	Germinating seed	547/62.3	4.5-5/58	np	*Km* = 61 μM	(Hegeman and Grabau, 2001; Singh *et al.*, 2013)
Barrel medic (*Medicago truncatula*)	MtPHY1	Roots and leaves, recombinant	543/np	np	np	Effective phytate hydrolysis	(Xiao, Harrison and Wang, 2005; Xiao *et al.*, 2006)
Arabidopsis thaliana	AtPAP23	Recombinant	np/77.7	np	np	Weak activity	(Zhu *et al.*, 2005; Lung *et al.*, 2008)
Tobacco (*Nicotiana tabacum*)	NtPAP	Root	551/56	np	Monomer	*Km* = 14.7 μM *kcat* = 908 s-1	(Lung *et al.*, 2008)
Arabidopsis thaliana	AtPAP15	Recombinant	532/60	4.5/23-37	Monomer	Specific activity = 10 U mg^{-1} *Km* = 278 μM, *Vmax* = 13.44 U mg^{-1}	(Zhang *et al.*, 2008; Kuang *et al.*, 2009; Wang *et al.*, 2009)
Wheat (*Triticum aestivum*)	TaPAPhy_a1	Mature grain, recombinant	550/58	5.5/55	Monomer	*Km* = 35 μM, *Vmax* = 223 μmol min^{-1} mg^{-1}, *kcat* = 279 s^{-1}, *kcat/Km* = 796 x104 s^{-1} M^{-1}	(Dionisio *et al.*, 2011)
Wheat (*Triticum aestivum*)	TaPAPhy_a2	Mature grain, recombinant	549/58.6	np	Monomer	np	(Dionisio *et al.*, 2011)

Wheat (*Triticum aestivum*)	TaPAPhy_b1	Germinating seed, recombinant	538/57.4	5.0/50	Monomer	*Km* = 45 µM<br>*Vmax* = 216 µmol min^{-1} mg^{-1}<br>*kcat* = 270 s^{-1}<br>*kcat/Km* = 600 x104 s^{-1} M^{-1}	(Dionisio *et al.*, 2011)
Wheat (*Triticum aestivum*)	TaPAPhy_b2	Germinating seed, recombinant	537/57.4	np	Monomer	np	(Dionisio *et al.*, 2011, 2012)
Barley (*Hordeum vulgare*)	HvPAPhy_a	Mature grain, recombinant	544/57.8	np	Monomer	*Km* = 36 µM<br>*Vmax* = 208 µmol min^{-1} mg^{-1}<br>*kcat* = 260 s^{-1}<br>*kcat/Km* = 722 $x10^4$ s^{-1} M^{-1}	(Dionisio *et al.*, 2011)
Barley (*Hordeum vulgare*)	HvPAPhy_b1	Germinating seed, recombinant	536/57.2	np	Monomer	np	(Dionisio *et al.*, 2011, 2012)
Barley (*Hordeum vulgare*)	HvPAPhy_b2	Germinating seed, recombinant	537/57.2	np	Monomer	*Km* = 46 µM<br>*Vmax* = 202 µmol min^{-1} mg^{-1}<br>*kcat* = 253 s^{-1}<br>*kcat/Km* = 550 $x10^4$ s^{-1} M^{-1}	(Dionisio *et al.*, 2011)

Maize (*Zea mays*)	ZmPAPhy_b	Germinating seed, recombinant	544/57.4	np	Monomer	*Km* = 48 μM<br>*Vmax* = 198 μmol min^{-1} mg^{-1}<br>k*cat* = 248 s^{-1}<br>*kcat/Km* = 517 x10^4 s^{-1} M^{-1}	(Dionisio *et al.*, 2011)
Rice (*Oryza sativa*)	OsPAPhy_b	Germinating seed, recombinant	539/57.5	np	Monomer	*Km* = 54 μM<br>*Vmax* = 185 μmol min^{-1} mg^{-1}<br>*kcat* = 231 s^{-1}<br>*kcat/Km* = 428 x10^4 s^{-1} M^{-1}	(Dionisio *et al.*, 2011)
Mungbean (*Vigna radiata*)	VrPAP1	Germinating seed	547/62	np	np	Contains five PAP motifs and partial homology with four PAPhy motifs	(Wongkaew, Srinives and Nakasathien, 2013)
White lupin (*Lupinus albus*)	LASAP3	Germinating seed, recombinant	543/np	5.5/np	np	*Km* = 83.1 μM	(Maruyama *et al.*, 2012)
Wheat (*Triticum aestivum*)	TaPAPhy_a3	Mature grain	539/np	np	np	Gene isolated	(Madsen *et al.*, 2013)
Wheat	TaPAPhy_b3	Germinating seed	536/np	np	np	Gene isolated	(Madsen *et al.*, 2013)

(*Triticum aestivum*)							
Einkorn (*Triticum monococcum*)	TmPAPhy_a 1	Mature grain	np	np	np	Gene isolated	(Madsen *et al.*, 2013)
Einkorn (*Triticum monococcum*)	TmPAPhy_b 1	Germinating seed	np	np	np	Gene isolated	(Madsen *et al.*, 2013)
Goatgrass (*Aegilops tauschii*)	AtaPAPhy_a 1	Mature grain	np	np	np	Gene isolated	(Madsen *et al.*, 2013)
Goatgrass (*Aegilops tauschii*)	AtaPAPhy_b 1	Germinating seed	np	np	np	Gene isolated	(Madsen *et al.*, 2013)
Rye (*Secale cereale*)	ScPAPhy_a1	Mature grain	np	np	np	Gene isolated	(Madsen *et al.*, 2013)
Rye (*Secale cereale*)	ScPAPhy_a2	Mature grain	np	np	np	Gene isolated	(Madsen *et al.*, 2013)
Rye (*Secale cereale*)	ScPAPhy_b1	Germinating seed	np	np	np	Gene isolated	(Madsen *et al.*, 2013)
Red kidney bean (*Phaseolus vulgaris*)	np	Root nodules	np	np	np	Expression levels of transcript correlate with phytase activity	(Lazali *et al.*, 2013, 2014)

Soybean (*Glycine max*)	GmPAP4	Roots and recombinant	442/50.3	np	np	0.15 µM Pi h^{-1} U^{-1} (control = 0.06 µM Pi h^{-1} U^{-1})	(Kong *et al.*, 2014)
Chlamydomonas reinhardtii	CrPAP1	np	np	np	np	Gene expression induced by addition of phytate	(Rivera-Solís *et al.*, 2014)
Chlamydomonas reinhardtii	CrPAP5	np	np	np	np	Gene expression induced by addition of phytate	(Rivera-Solís *et al.*, 2014)
Trifoliate orange (Poncirus trifoliata)	PtPAP3	Germinating seed, recombinant	np/66	5.5/37	Monomer	*Km* = 46.2 µM *Vmax* = 214 µmol min^{-1} mg^{-1} *kcat* = 243 s^{-1} *kcat/Km* = 5.49 s^{-1} $µmol^{-1}$	(Shu, Wang and Xia, 2015)

Supplemental Table 3. Estimation of kinetic parameters for $InsP_6$ hydrolysis by recombinant wheat phytase isoform b2 (TaPHY_b2) and active site mutants.

Michaelis constants (K_m) are expressed as substrate concentration (µM) for the recombinant wild type (WT) enzyme and mutants (K348A and K410A). Note that the phytase activity of the H229A mutant was insufficient to allow reliable estimation of kinetic parameters. Maximal velocities (V_{max}) are expressed as phosphate concentration released (µM) per time of reaction (min) and amount of enzyme (µg). Catalytic rate constants (*kcat*) are expressed in s^{-1}. The calculated value of the standard error is shown for each parameter. The R^2 of the curve fit is also included. Significance (Student's *t*-test) relative to WT shown in brackets where $p > 0.05$ (n.s.); $p < 0.05$ (*); $p < 0.001$ (***).

Parameter	WT	K348A	K410A
K_m / µM	76.4 ± 7.7	214.6 ± 46.6 (*)	307.6 ± 56.7 (*)
V_{max} / µM min^{-1} µg^{-1}	85.5 ± 3.1	102.1 ± 10.8 (n.s.)	11.3 ± 1.2 (***)
kcat / s^{-1}	23.8 ± 0.9	28.4 ± 3.0 (n.s.)	3.1 ± 0.3 (***)
R^2	0.98	0.96	0.98

Supplementary Table 4. Comparison of active site residues of plant PAPhy. Amino acid positions within 10 Å of the phosphate ion in the TaPAPhy_b2 crystal structure are listed. Residues in each which differ to that in TaPAPhy_b2 are shaded in lilac (TaPAPhy_a1), green (HvPAPhy_a), orange (OsPAPhy_b), yellow (ZmPAPhy_b) or pink (GmPAPhy_b). 'Motif' indicates the motif (PAPhy or PAP) in which the corresponding residue is found. 'b→a' shows residue substitutions between b and a TaPAPhy isoforms. 'Cereal→Soybean' shows residue substitutions between the cereal enzymes considered and GmPAPhy_B.

TaPAPhy_b2	TaPAPhy_b1	TaPAPhy_a1	HvPAPhy_a	OsPAPhy_b	ZmPAPhy_b	GmPAPhy_b	Motif	b → a	Cereal → Soybean
His23	His23	His23	His23	His22	His22	Val14	PAPhy 1	n/a	His → Val
Leu199	Leu199	Val199	Val199	Leu198	Leu198	Ile189	n/a	n/a	n/a
Ser203	Ser203	Cys203	Ser203	Ser202	Cys202	Thr193	PAP 2	n/a	n/a
Leu207	Leu207	Met207	Met207	Leu206	Leu206	Leu197	n/a	Leu → Met	n/a
Thr215	Thr215	Ala215	Thr215	Thr214	Ala214	Ser205	n/a	n/a	n/a
Ser221	Ser221	Ala221	Ser221	Ser220	Ala220	Ser211	PAPhy 4	n/a	n/a
Ala223	Ala223	Gly223	Gly223	Ala222	Ala222	Pro213	PAPhy 4	n/a	Ala/Gly → Pro
Lys224	Lys224	Lys224	Lys224	Asn223	Lys223	Leu214	PAPhy 4	n/a	Lys/Asn → Leu
Ser225	Ser225	Ser225	Ser225	Ser224	Ser224	Deletion	PAPhy 4	n/a	Ser → Deletion
Gln263	Gln263	Glu263	Glu263	Glu262	Gln262	Lys252	n/a	n/a	Gln/Glu → Lys
Ala306	Ala306	Ala306	Ala306	Ala305	Ser305	Ala295	n/a	n/a	n/a
Ala308	Ala308	Ala308	Ala308	Ala307	Ala307	Ile297	n/a	n/a	n/a
Ala341	Ala341	Ala341	Ala341	Ala340	Ala340	Pro330	n/a	n/a	n/a
Ser345	Ser345	Thr345	Thr345	Ser344	Thr344	Ser334	n/a	n/a	n/a
Thr346	Thr346	Thr346	Thr346	Thr345	Thr345	Ser335	n/a	n/a	n/a
Tyr347	Tyr347	Tyr347	Tyr347	Phe346	Tyr346	Tyr336	n/a	n/a	n/a
Lys348	Lys348	Lys348	Lys348	Lys347	Lys347	Glu337	n/a	n/a	Lys → Glu
Ala354	Ala354	Val354	Val354	Ala353	Ala353	Ala343	n/a	Ala → Val	n/a
Ser401	Ser401	Ser401	Ser401	Ser400	Ser400	Thr390	n/a	n/a	n/a
Thr413	Thr413	Thr413	Thr413	Thr412	Thr412	Ile402	PAPhy 5	n/a	Thr → Ile
Thr414	Thr414	Thr414	Thr414	Ser413	Ala413	Lys403	PAPhy 5	n/a	Thr/Ser/Ala → Lys
His415	His415	His415	His415	Tyr414	His414	Phe404	PAPhy 5	n/a	n/a
Asp418	Asp418	Glu418	Glu418	Glu417	Glu417	Glu407	PAPhy 5	n/a	n/a
Pro419	Pro419	Pro419	Pro419	Pro418	Ala418	Pro408	PAPhy 5	n/a	n/a
Arg421	Arg421	His421	His421	Arg420	His420	His410	PAPhy 5	n/a	n/a
Glu424	Glu424	Asp424	Asp424	Asp423	Asp423	Asp413	PAPhy 5	n/a	n/a
Met426	Met426	Arg426	Arg426	Leu425	Ala425	Leu415	PAPhy 5	n/a	n/a
Ser427	Ser427	Pro427	Pro427	Ser426	Ser426	Ser416	PAPhy 5	Ser → Pro	n/a
Thr428	Thr428	Lys428	Lys428	Thr427	Thr427	Thr417	PAPhy 5	Thr → Lys	n/a
Asp430	Asp430	Asn430	Asn430	Asp429	Asp429	Asp419	PAPhy 5	Asp → Asn	n/a
Ala431	Ala431	Ala431	Ala431	Pro430	Pro430	Pro420	PAPhy 5	n/a	n/a
Phe432	Phe432	Phe432	Phe432	Phe431	Phe431	Tyr421	PAPhy 5	n/a	n/a
Met433	Met433	Ile433	Ile433	Met432	Met432	Met422	PAPhy 5	Met → Ile	n/a

Supplemental Table 5. Purple acid phosphatase sequences used in bioinformatic analyses. Collection of the purple acid phosphatase sequences, with and without phytase activity. PAPhy, pink shading. Plant PAPs, lilac shading. Animal PAPs, orange shading. Microalgal PAPs, green shading. Fungal PAPs, yellow shading. Bacterial PAPs, blue shading. Sequences excluded during the analysis, red shading. ‘n/a’, not applicable. PAPhy sequences are separated according to whether characterised (PAPhy), predicted by sequence homology (Predicted PAPhy) or sequence outliers (PAPhy outlier). Plant and animal PAP sequences are separated into HMW and LMW.

Name	Organism	Group	Alternative names	UniProt ID
AtPAP15	*Arabidopsis thaliana*	PAPhy	n/a	Q9SFU3
GmPAPhy_b	*Glycine max*	PAPhy	GmPhy	Q93XG4
HvPAPhy_a	*Hordeum vulgare*	PAPhy	(Hv)P2	C4PKL2
HvPAPhy_b1	*Hordeum vulgare*	PAPhy	(Hv)P1	C4PKL3
HvPAPhy_b2	*Hordeum vulgare*	PAPhy	(Hv)P1	C4PKL4
LaPAPhy	*Lupinus albus*	PAPhy	LASAP3	D2YZL4
MtPAPhy	*Medicago truncatula*	PAPhy	MtPHY1	Q3ZFI1
NtPAPhy	*Nicotiana tabacum*	PAPhy	NtPAP	A5YBN1
OsPAPhy_b	*Oryza sativa*	PAPhy	(Os)F1, (Os)F2, OsPAP5	D6QSX9
PtPAP3	*Poncirus trifoliata*	PAPhy	n/a	V9LXK5
TaPAPhy_a1	*Triticum aestivum*	PAPhy	(Ta)PHYI	C4PKK7
TaPAPhy_b1	*Triticum aestivum*	PAPhy	n/a	C4PKK9
TaPAPhy_b2	*Triticum aestivum*	PAPhy	n/a	C4PKL0
ZmPAPhy_b	*Zea mays*	PAPhy	n/a	C4PKL6
AtaPAPhy_a1	*Aegilops tauschii*	Predicted PAPhy	n/a	F6MIX0
AtaPAPhy_b1	*Aegilops tauschii*	Predicted PAPhy	n/a	F6MIX1
PvPAPhy	*Phaseolus vulgaris*	Predicted PAPhy	n/a	V7B3Z4
ScPAPhy_a1	*Secale cereale*	Predicted PAPhy	n/a	F6MIX2
ScPAPhy_a2	*Secale cereale*	Predicted PAPhy	n/a	F6MIX4
ScPAPhy_b1	*Secale cereale*	Predicted PAPhy	n/a	F6MIX5
TaPAPhy_a2	*Triticum aestivum*	Predicted PAPhy	(Ta)PHYII	C4PKK8
TaPAPhy_a3	*Triticum aestivum*	Predicted PAPhy	n/a	F6MIW2
TaPAPhy_b3	*Triticum aestivum*	Predicted PAPhy	n/a	F6MIW6
TmPAPhy_a1	*Triticum monococcum*	Predicted PAPhy	n/a	F6MIW8
TmPAPhy_b1	*Triticum monococcum*	Predicted PAPhy	n/a	F6MIW9
VrPAPhy	*Vigna radiata*	Predicted PAPhy	VrPAP1	B5ARZ7
AtPAP23	*Arabidopsis thaliana*	PAPhy outlier	AtPAP_c	Q6TPH1
GmPAP4	*Glycine max*	PAPhy outlier	n/a	V9HXG4
AcPAP	*Allium cepa*	HMW Plant PAP	ACPEPP	Q93WP4
AlPAP15	*Arabidopsis lyrata*	HMW Plant PAP	n/a	D7L636
AoPAP32	*Anchusa officinalis*	HMW Plant PAP	n/a	Q9XF09
AtPAP10	*Arabidopsis thaliana*	HMW Plant PAP	n/a	Q9SIV9
AtPAP11	*Arabidopsis thaliana*	HMW Plant PAP	n/a	Q9SI18
AtPAP12	*Arabidopsis thaliana*	HMW Plant PAP	n/a	Q38924
AtPAP13	*Arabidopsis thaliana*	HMW Plant PAP	n/a	O48840
AtPAP20	*Arabidopsis thaliana*	HMW Plant PAP	n/a	Q9LXI7
AtPAP21	*Arabidopsis thaliana*	HMW Plant PAP	n/a	Q9LXI4
AtPAP22	*Arabidopsis thaliana*	HMW Plant PAP	n/a	Q8S340
AtPAP25	*Arabidopsis thaliana*	HMW Plant PAP	n/a	O23244
AtPAP26	*Arabidopsis thaliana*	HMW Plant PAP	n/a	Q949Y3
AtPAP5	*Arabidopsis thaliana*	HMW Plant PAP	n/a	Q9C927
AtPAP6	*Arabidopsis thaliana*	HMW Plant PAP	n/a	Q9C510

Name	Organism	Group	Alternative names	UniProt ID
GmPAP1	*Glycine max*	HMW Plant PAP	n/a	Q09131
GmPAP3	*Glycine max*	HMW Plant PAP	n/a	Q6YGT9
HvPAP_c	*Hordeum vulgare*	HMW Plant PAP	n/a	C4PKL5
IbPAP1	*Ipomoea batatas*	HMW Plant PAP	SpPAP2	Q9SE00
IbPAP2	*Ipomoea batatas*	HMW Plant PAP	SpPAP3	Q9SDZ9
IbPAP3	*Ipomoea batatas*	HMW Plant PAP	SpPAP1	Q9ZP18
LaAP1	*Lupinus albus*	HMW Plant PAP	n/a	Q93VM7
LaAP2	*Lupinus albus*	HMW Plant PAP	n/a	Q9XJ24
LlAP1	*Lupinus luteus*	HMW Plant PAP	(Ll)AP1; acPase1	Q8L5E1
LlAP2	*Lupinus luteus*	HMW Plant PAP	(Ll)AP2; acpase2	Q8L6L1
LlPPD1	*Lupinus luteus*	HMW Plant PAP	PPD1	Q8VX11
LlPPD2	*Lupinus luteus*	HMW Plant PAP	PPD2	Q8VXF6
LlPPD4	*Lupinus luteus*	HMW Plant PAP	PPD4	Q8VXF4
LpPAP	*Landoltia punctata*	HMW Plant PAP	n/a	Q9MB07
MtPAP1	*Medicago truncatula*	HMW Plant PAP	n/a	Q4KU02
NtPAP	*Nicotiana tabacum*	HMW Plant PAP	n/a	Q84KZ3
OsPAP2	*Oryza sativa*	HMW Plant PAP	n/a	Q8S505
OsPAP3	*Oryza sativa*	HMW Plant PAP	Os08g0280100	Q6ZCX8
OsPAP4	*Oryza sativa*	HMW Plant PAP	OsI_28583	B8B909
PpPAP	*Physcomitrella patens*	HMW Plant PAP	n/a	A9SPI2
PvPAP1	*Phaseolus vulgaris*	HMW Plant PAP	PvPAP_tIII	P80366
PvPAP2	*Phaseolus vulgaris*	HMW Plant PAP	KeACP; PvPAP_tIV	Q764C1
RcPAP1	*Ricinus communis*	HMW Plant PAP	RCOM_1019210	B9RWG6
RcPAP2	*Ricinus communis*	HMW Plant PAP	RCOM_0003680	B9SXP8
RcPAP3	*Ricinus communis*	HMW Plant PAP	RCOM_0003560	B9SXP6
SbPAP	*Sorghum bicolor*	HMW Plant PAP	SORBI_3007G091100	A0A1Z5R9T8
StPAP3	*Solanum tuberosum*	HMW Plant PAP	n/a	Q6J5M8
TaACP	*Triticum aestivum*	HMW Plant PAP	n/a	C4PKL1
VvPAP	*Vitis vinifera*	HMW Plant PAP	VITISV_037278	A5BGI6
ZmPAP_c	*Zea mays*	HMW Plant PAP	n/a	C4PKL7
AtPAP17	*Arabidopsis thaliana*	LMW Plant PAP	AtACP5	Q9SCX8
AtPAP3	*Arabidopsis thaliana*	LMW Plant PAP	n/a	Q8H129
AtPAP7	*Arabidopsis thaliana*	LMW Plant PAP	n/a	Q8S341
AtPAP8	*Arabidopsis thaliana*	LMW Plant PAP	n/a	Q8VYZ2
BrPAP17_1	*Brassica rapa*	LMW Plant PAP	n/a	D6MW88
GmPAP2	*Glycine max*	LMW Plant PAP	n/a	Q9LL80
IbPAP4	*Ipomoea batatas*	LMW Plant PAP	n/a	Q9LL81
LlACP3	*Lupinus luteus*	LMW Plant PAP	n/a	Q707M7
LlPPD3	*Lupinus luteus*	LMW Plant PAP	PPD3	Q8VXF5
OsPAP1	*Oryza sativa*	LMW Plant PAP	OSJNBa0023I19.10	Q7XH73
PvPAP3	*Phaseolus vulgaris*	LMW Plant PAP	n/a	D2D4J4
PvPAP4	*Phaseolus vulgaris*	LMW Plant PAP	n/a	Q9LL79
PvPAP5	*Phaseolus vulgaris*	LMW Plant PAP	n/a	E2D740
StPAP1	*Solanum tuberosum*	LMW Plant PAP	n/a	Q6J5M7
ZmPAP	*Zea mays*	LMW Plant PAP	n/a	C4IZM1
AgPAP	*Anopheles gambiae*	HMW Animal PAP	Aga_PAPL1	Q7PUN5
AmPAP	*Apis mellifera*	HMW Animal PAP	Ame_PAPL1	A0A087ZWE4
CePAP1	*Caenorhabditis elegans*	HMW Animal PAP?	CELE_F02E9.7	O01320
CePAP3	*Caenorhabditis elegans*	HMW Animal PAP	Cel_PAPL3	Q9NAM9
DmPAP1	*Drosophila melanogaster*	HMW Animal PAP	Dme_PAPL1; DmPAP_b	Q9VZ56
DmPAP2	*Drosophila melanogaster*	HMW Animal PAP	Dme_PAPL2	Q9VZ58
DmPAP3	*Drosophila melanogaster*	HMW Animal PAP	Dme_PAPL3; DmPAP_a	Q9VZ57
HsPAP7	*Homo sapiens*	HMW Animal PAP	Hsa_PAPL1; HsACP7	Q6ZNF0

Name	Organism	Group	Alternative names	UniProt ID
MmPAP7	*Mus musculus*	HMW Animal PAP	Mmu_PAPL1; MmACP7	Q8BX37
TnPAP1	*Tetraodon nigroviridis*	HMW Animal PAP	Tni_PAPL1	Q4RLR4
DrPAP1	*Danio rerio*	LMW Animal PAP	Dre_PAP1; DrACP5a	Q6DHF5
DrPAP2	*Danio rerio*	LMW Animal PAP	Dre_PAP2; DrACP5a	Q7SXT1
HsPAP5	*Homo sapiens*	LMW Animal PAP	Hsa_ACP5	P13686
MmPAP5	*Mus musculus*	LMW Animal PAP	Mmu_ACP5	Q05117
RnPAP5	*Ratus novergicus*	LMW Animal PAP	Rn_ACP5	P29288
SsPAP5	*Sus scrofa*	LMW Animal PAP	Ss_ACP5	P09889
TnPAP2	*Tetraodon nigroviridis*	LMW Animal PAP	n/a	Q4S7S5
XlPAP1	*Xenopus laevis*	LMW Animal PAP	Xla_PAP1; XlACP5	Q6GNG2
XlPAP2	*Xenopus laevis*	LMW Animal PAP	Xla_PAP2; XlACP5	Q6IP56
XtPAP5	*Xenopus tropicalis*	LMW Animal PAP	XtACP5	Q66IG6
CrPAP1	*Chlamydomonas reinhardtii*	Microalgal PAP	Cre16.g672250.t1.3	n/a
CrPAP2	*Chlamydomonas reinhardtii*	Microalgal PAP	Cre13.g578350.t1.2	n/a
CrPAP3	*Chlamydomonas reinhardtii*	Microalgal PAP	Cre11.g476700.t1.2	n/a
CrPAP4	*Chlamydomonas reinhardtii*	Microalgal PAP	Cre11.g468500.t1.3	n/a
CrPAP5	*Chlamydomonas reinhardtii*	Microalgal PAP	Cre12.g500200.t1.3	n/a
CrPAP6	*Chlamydomonas reinhardtii*	Microalgal PAP	Cre06.g259650.t1.2	n/a
MpPAP1	*Micromonas pusilla*	Microalgal PAP	MpPAP(3567)	n/a
MpPAP2	*Micromonas pusilla*	Microalgal PAP	MpPAP(48357)	n/a
MpPAP3	*Micromonas pusilla*	Microalgal PAP	MpPAP(57207)	n/a
MpPAP4	*Micromonas pusilla*	Microalgal PAP	MpPAP(146371)	n/a
OlPAP1	*Ostreococcus lucimarinus*	Microalgal PAP	OlPAP(1604)	n/a
OlPAP2	*Ostreococcus lucimarinus*	Microalgal PAP	OlPAP(2983)	n/a
AfPAP	*Aspergillus ficuum*	Fungal PAP	AphA; APase6; AfPAPhyC	Q12546
AnidPAP	*Aspergillus nidulans*	Fungal PAP	suApacA	Q92200
BcPAP	*Burkholderia cenocepacia J2315*	Bacterial PAP	BCAM1663	B4EKR2
BmaPAP	*Burkholderia mallei ATCC 23344*	Bacterial PAP	BMA0259	A0A0H2WHP3
BpsPAP	*Burkholderia pseudomallei K96243*	Bacterial PAP	BPSL0702	Q63X35
LePAP	*Lysobacter enzymogenes*	Bacterial PAP	phoA	Q05205
MbPAP	*Mycobacterium bovis AF2122/97*	Bacterial PAP	BQ2027_MB2608	A0A1R3Y2F9
MtubPAP	*Mycobacterium tuberculosis H37Rv*	Bacterial PAP	Rv2577	P9WL81

Supplemental Table 6. X-ray data collection and structure refinement statistics. Figures in brackets refer to the high resolution data bin.

**Structure**	**Product**	**Substrate**	**InsS$_6$**	**H229A**
**PDB entry code**	**6GIT**	**6GIZ**	**6GJ2**	**6GJA**
**Crystal parameters**				
Space group	*H*3	*H*3	*H*3	*H*3
a, b, c (Å)	126.48, 126.48, 106.80	126.73, 126.73, 107.04	126.02, 126.02, 105.91	125.98, 125.98, 106.55
α, β, γ (°)	90.00, 90.00, 120.00	90.00, 90.00, 120.00	90.00, 90.00, 120.00	90.00, 90.00, 120.00
**Data collection**				
Wavelength (Å)	0.9763	0.9763	0.9763	0.9763
Resolution (Å)	63.24-1.42 (1.44-1.42)	48.11-1.54 (1.57-1.54)	48.51-1.68 (1.71-1.68)	38.46-1.50 (1.53-1.50)
R_{merge} (%)	4.7 (50.6)	5.6 (71.4)	6.4 (118.4)	5.6 (59.1)
Rpim	0.029 (0.608)	0.036 (0.876)	0.032 (0.569)	0.036 (0.515)
CC1/2	0.999 (0.69)	0.999 (0.554)	0.999 (0.537)	0.997 (0.469)
$< I/\sigma(I) >$	14.6 (2.4)	13.4 (1.5)	12.6 (1.3)	12.4 (1.7)
Completeness (%)	92.6 (99.4)	99.8 (99.7)	99.9 (100.0)	96.6 (75.0)
Multiplicity	3.5 (3.3)	3.4 (2.9)	5.1 (5.1)	3.2 (2.0)
$CC_{1/2}$	1.0 (0.7)	1.0 (0.5)	1.0 (0.5)	1.0 (0.4)
**Refinement**				
Total No. of atoms	5,093	4,915	4,748	4,940
Water molecules	489	433	286	443

No. of reflections	111,798	94,712	71,408	97,457
R_{work} (%)	13.2	13.6	13.4	12.8
R_{free} (%)	15.8	16.7	17.6	15.2
Wilson *B* factor (Å^2)	14.5	18.8	26.2	16.0
Anisotropy	0.135	0.131	0.24	0.062
RMS deviations				
Bonds (Å)	0.005	0.006	0.011	0.005
Angles (°)	0.838	0.896	0.838	0.833
Planes (Å)	0.006	0.005	0.006	0.006
Ramachandran plot				
Favoured (%)	97.23	96.80	96.20	96.91
Allowed (%)	2.77	3.20	3.60	3.09
Outliers (%)	0.00	0.00	0.20	0.00
Mean *B* factors (Å^2)	23.0	28.0	37.0	24.0

SUPPLEMENTAL FIGURES

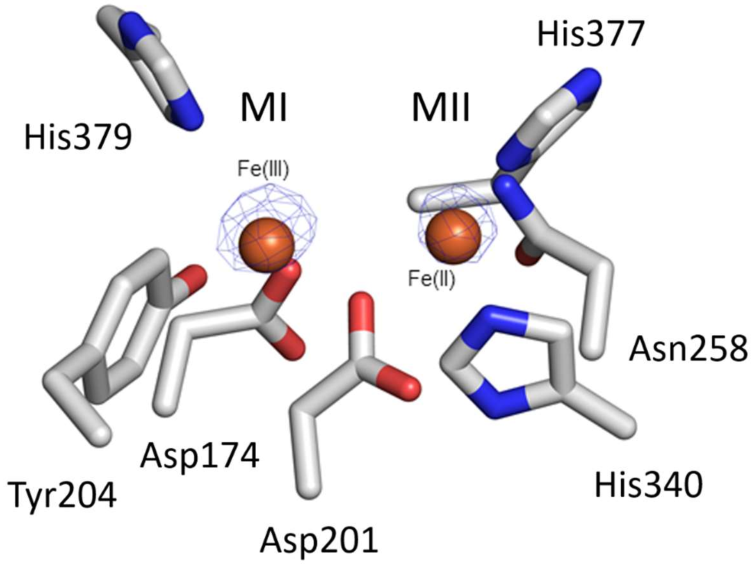

Supplemental Figure 1. Anomalous difference electron density map calculated using a TaPAPhy_b2 Fe-SAD dataset collected at the iron K-edge. Anomalous difference map calculated using data collected at a wavelengthof 1.74 Å is displayed as a blue mesh at a contour level of 10σ. Iron ions showed as brown spheres and labelled. The MI site is presumed to be in the ferric state and MII in the ferrous state. Sidechains of the residues acting as metal ligands in the active site are shown as sticks and coloured by element with carbons in grey.

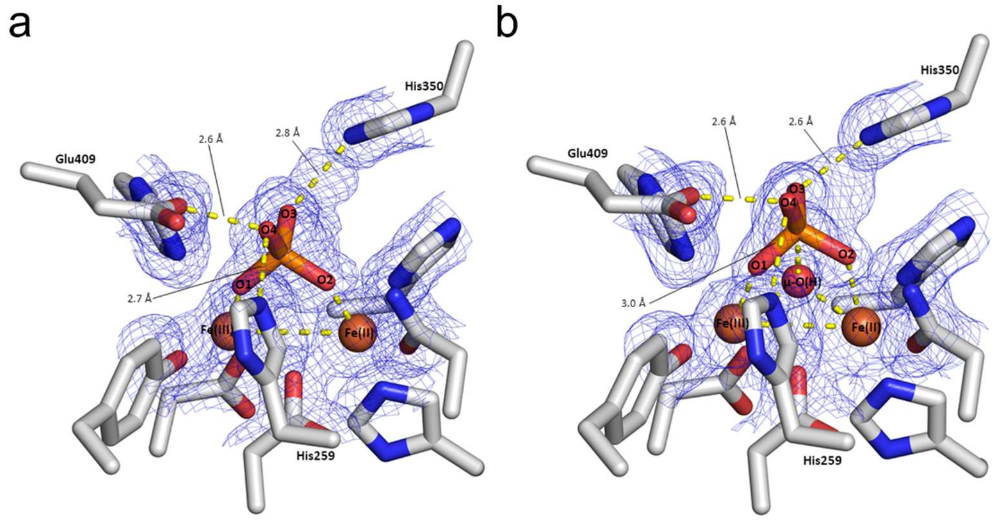

Supplemental Figure 2. The binuclear centre of the TaPAPhy_b2:PO_4 complex in (a) product- and (b) substrate-bound states. $2mF_o$-DF_c electron density maps are displayed as a blue mesh at a contour level of 1σ. Iron ions showed as brown spheres and labelled. The μ-oxo bridge is shown as a red sphere. Sidechains of the residues acting as metal ligands in the active site are shown as sticks and coloured by element with carbons in grey. Selected coordination interactions are indicated by yellow dashed lines with distances shown in Å.

Supplemental Figure 3. Multiple sequence alignment of PAPhys and HMW PAPs. PAPhy sequences are separated into groups depending on whether they have been biochemically characterised (PAPhy), predicted by sequence homology (Predicted PAPhy) or are sequence outliers (PAPhy outlier). Signal peptides are included when present in the corresponding entry from the UniProt database. Note that UniProt entry for TaPAPhy_b2 contains its signal peptide and so the residue numbering in this figure differs from that in the main paper. Specifically, subtract 20 from the residue number shown in this figure of the Supplemental Information to arrive at the corresponding number of the residue in the mature enzyme.

Horizontal groups	Vertical features
PAPhy	Signal peptide
Predicted PAPhy	PAP motifs I to V
PAPhy Outliers	PAPhy motifs 1 to 4
Plant PAPs	Proposed PAPhy motif 5

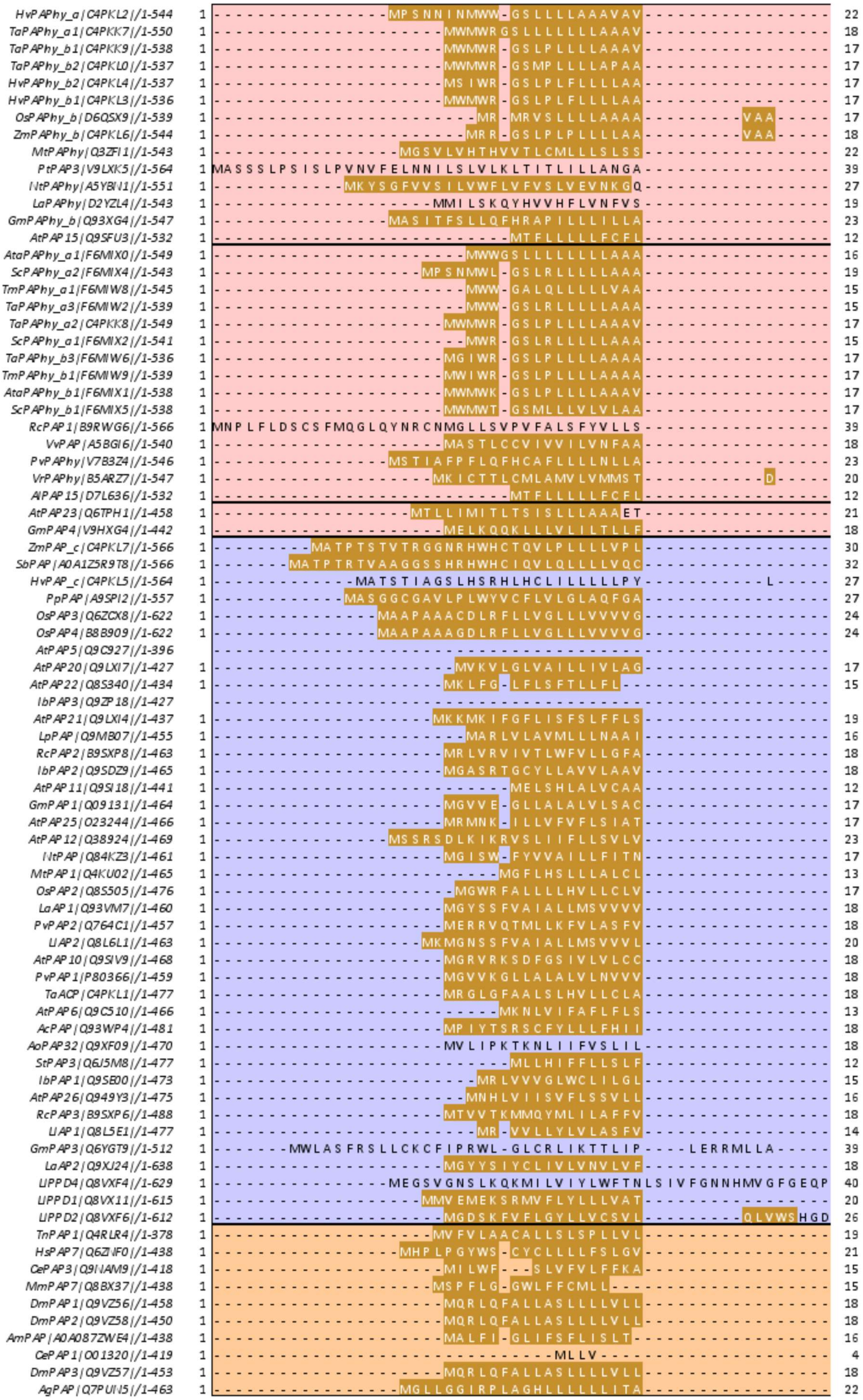

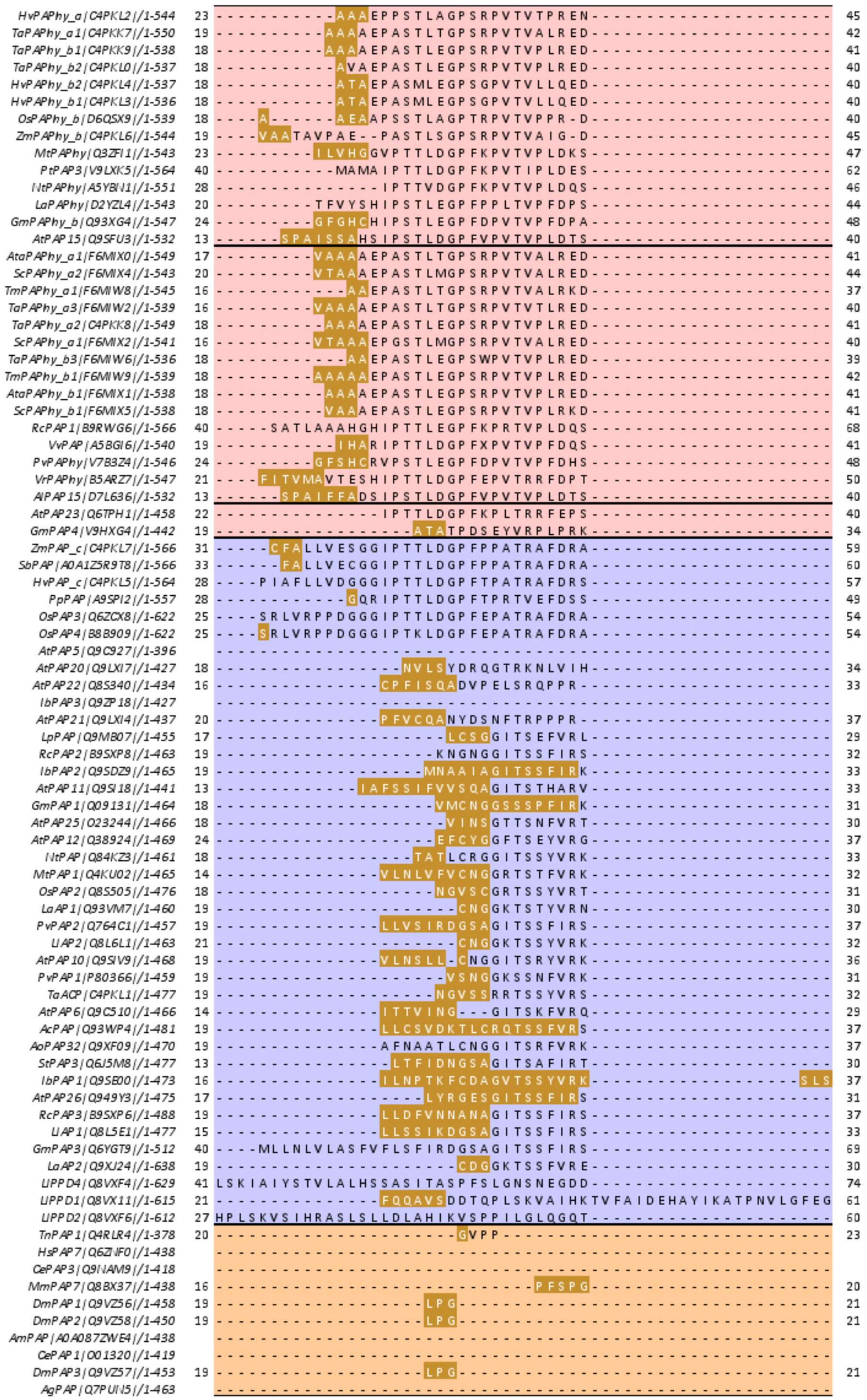

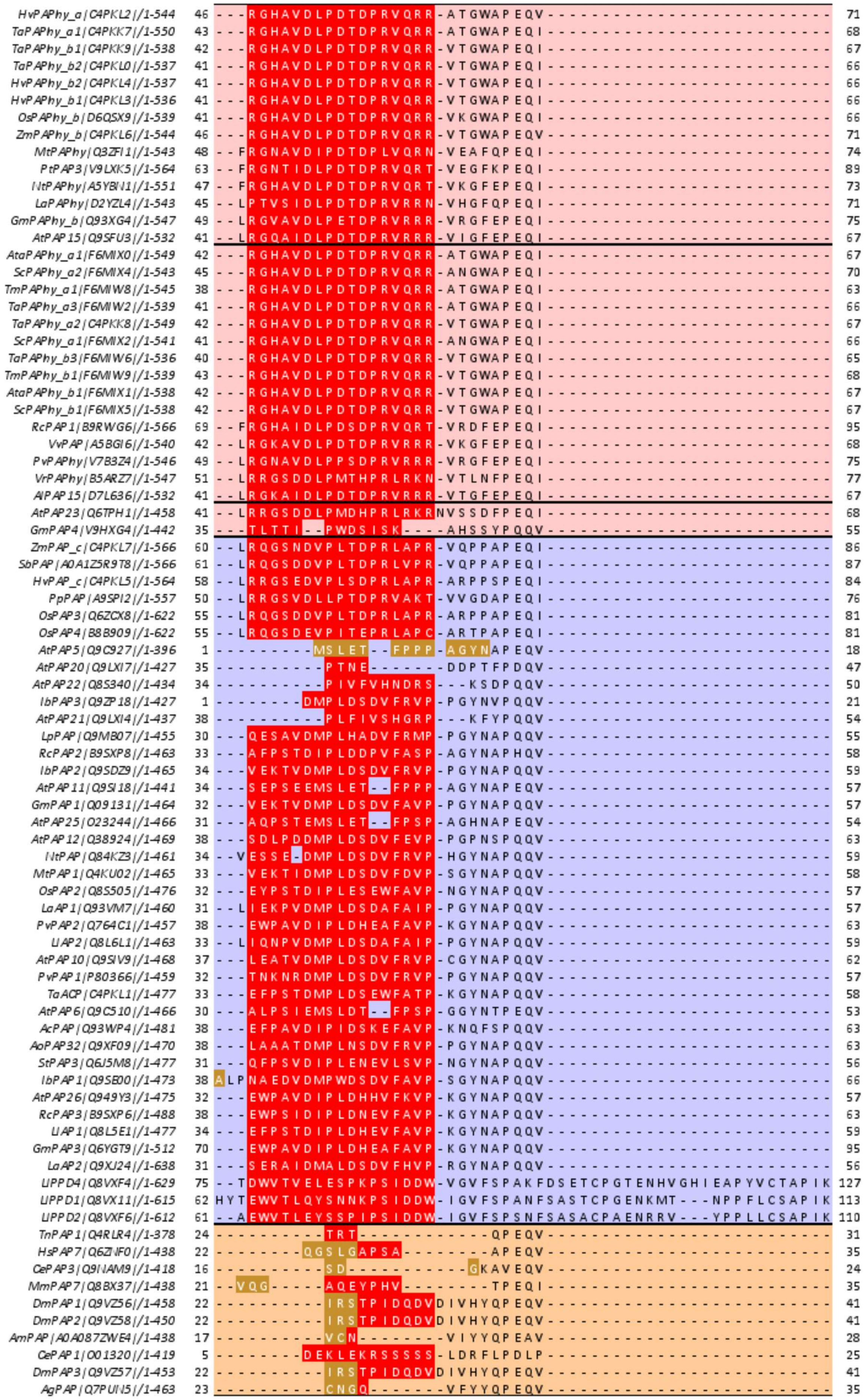
HvPAPhy_a|C4PKL2|/1-544 46 ---RGHAVDLPDTDPRVQRR-ATGWAPEQV--------------------------- 71
TaPAPhy_a1|C4PKK7|/1-550 43 ---RGHAVDLPDTDPRVQRR-ATGWAPEQI--------------------------- 68
TaPAPhy_b1|C4PKK9|/1-538 42 ---RGHAVDLPDTDPRVQRR-VTGWAPEQI--------------------------- 67
TaPAPhy_b2|C4PKL0|/1-537 41 ---RGHAVDLPDTDPRVQRR-VTGWAPEQI--------------------------- 66
HvPAPhy_b2|C4PKL4|/1-537 41 ---RGHAVDLPDTDPRVQRR-VTGWAPEQI--------------------------- 66
HvPAPhy_b1|C4PKL3|/1-536 41 ---RGHAVDLPDTDPRVQRR-VTGWAPEQI--------------------------- 66
OsPAPhy_b|D6QSX9|/1-539 41 ---RGHAVDLPDTDPRVQRR-VKGWAPEQI--------------------------- 66
ZmPAPhy_b|C4PKL6|/1-544 46 ---RGHAVDLPDTDPRVQRR-VTGWAPEQV--------------------------- 71
MtPAPhy|Q3ZFI1|/1-543 48 --FRGNAVDIPDTDPLVQRN-VEAFQPEQI--------------------------- 74
PtPAP3|V9LXK5|/1-564 63 --FRGNTIDLPDTDPRVQRT-VEGFKPEQI--------------------------- 89
NtPAPhy|A5YBN1|/1-551 47 --FRGHAVDLPDTDPRVQRT-VKGFEPEQI--------------------------- 73
LaPAPhy|D2YZL4|/1-543 45 --LPTVSIDLPDTDPRVRRN-VHGFQPEQI--------------------------- 71
GmPAPhy_b|Q93XG4|/1-547 49 --LRGVAVDLPETDPRVRRR-VRGFEPEQI--------------------------- 75
AtPAP15|Q9SFU3|/1-532 41 --LRGQAIDLPDTDPRVRRR-VIGFEPEQI--------------------------- 67
AtaPAPhy_a1|F6MIX0|/1-549 42 ---RGHAVDLPDTDPRVQRR-ATGWAPEQI--------------------------- 67
ScPAPhy_a2|F6MIX4|/1-543 45 ---RGHAVDLPDTDPRVQRR-ANGWAPEQI--------------------------- 70
TmPAPhy_a1|F6MIW8|/1-545 38 ---RGHAVDLPDTDPRVQRR-ATGWAPEQI--------------------------- 63
TaPAPhy_a3|F6MIW2|/1-539 41 ---RGHAVDLPDTDPRVQRR-ATGWAPEQI--------------------------- 66
TaPAPhy_a2|C4PKK8|/1-549 42 ---RGHAVDLPDTDPRVQRR-VTGWAPEQI--------------------------- 67
ScPAPhy_a1|F6MIX2|/1-541 41 ---RGHAVDLPDTDPRVQRR-ANGWAPEQI--------------------------- 66
TaPAPhy_b3|F6MIW6|/1-536 40 ---RGHAVDLPDTDPRVQRR-VTGWAPEQI--------------------------- 65
TmPAPhy_b1|F6MIW9|/1-539 43 ---RGHAVDLPDTDPRVQRR-VTGWAPEQI--------------------------- 68
AtaPAPhy_b1|F6MIX1|/1-538 42 ---RGHAVDLPDTDPRVQRR-VTGWAPEQI--------------------------- 67
ScPAPhy_b1|F6MIX5|/1-538 42 ---RGHAVDLPDTDPRVQRR-VTGWAPEQI--------------------------- 67
RcPAP1|B9RWG6|/1-566 69 --FRGHAIDLPDSDPRVQRT-VRDFEPEQI--------------------------- 95
VvPAP|A5BGI6|/1-540 42 --LRGKAVDLPDTDPRVRRR-VKGFEPEQI--------------------------- 68
PvPAPhy|V7B3Z4|/1-546 49 --LRGNAVDLPPSDPRVRRR-VRGFEPEQI--------------------------- 75
VrPAPhy|B5ARZ7|/1-547 51 --LRRGSDDLPMTHPRLRKN-VTLNFPEQI--------------------------- 77
AlPAP15|D7L636|/1-532 41 --LRGKAIDLPDTDPRVRRR-VTGFEPEQI--------------------------- 67
AtPAP23|Q6TPH1|/1-458 41 --LRRGSDDLPMDHPRLRKRNVSSDFPEQI--------------------------- 68
GmPAP4|V9HXG4|/1-442 35 ---TLTTI--PWDSISK----AHSSYPQQV--------------------------- 55
ZmPAP_c|C4PKL7|/1-566 60 --LRQGSNDVPLTDPRLAPR-VQPPAPEQI--------------------------- 86
SbPAP|A0A1Z5R9T8|/1-566 61 --LRQGSDDVPLTDPRLVPR-VQPPAPEQI--------------------------- 87
HvPAP_c|C4PKL5|/1-564 58 --LRRGSEDVPLSDPRLAPR-ARPPSPEQI--------------------------- 84
PpPAP|A9SPI2|/1-557 50 --LRRGSVDLLPTDPRVAKT-VVGDAPEQI--------------------------- 76
OsPAP3|Q6ZCX8|/1-622 55 --LRQGSDDVPLTDPRLAPR-ARPPAPEQI--------------------------- 81
OsPAP4|B8B909|/1-622 55 --LRQGSDEVPITEPRLAPC-ARTPAPEQI--------------------------- 81
AtPAP5|Q9C927|/1-396 1 ---------MSLET--FPPP-AGYNAPEQV--------------------------- 18
AtPAP20|Q9LXI7|/1-427 35 -----------PTNE------DDPTFPDQV--------------------------- 47
AtPAP22|Q8S340|/1-434 34 -----------PIVFVHNDRS---KSDPQQV--------------------------- 50
IbPAP3|Q9ZP18|/1-427 1 ---------DMPLDSDVFRVP-PGYNVPQQV--------------------------- 21
AtPAP21|Q9LXI4|/1-437 38 -----------PLFIVSHGRP---KFYPQQV--------------------------- 54
LpPAP|Q9MB07|/1-455 30 ---QESAVDMPLHADVFRMP-PGYNAPQQV--------------------------- 55
RcPAP2|B9SXP8|/1-463 33 ---AFPSTDIPLDDPVFASP-AGYNAPHQV--------------------------- 58
IbPAP2|Q9SDZ9|/1-465 34 ---VEKTVDMPLDSDVFRVP-PGYNAPQQV--------------------------- 59
AtPAP11|Q9SI18|/1-441 34 ---SEPSEEMSLET--FPPP-AGYNAPEQV--------------------------- 57
GmPAP1|Q09131|/1-464 32 ---VEKTVDMPLDSDVFAVP-PGYNAPQQV--------------------------- 57
AtPAP25|O23244|/1-466 31 ---AQPSTEMSLET--FPSP-AGHNAPEQV--------------------------- 54
AtPAP12|Q38924|/1-469 38 ---SDLPDDMPLDSDVFEVP-PGPNSPQQV--------------------------- 63
NtPAP|Q84KZ3|/1-461 34 --VESSE-DMPLDSDVFRVP-HGYNAPQQV--------------------------- 59
MtPAP1|Q4KU02|/1-465 33 ---VEKTIDMPLDSDVFDVP-SGYNAPQQV--------------------------- 58
OsPAP2|Q8S505|/1-476 32 ---EYPSTDIPLESEWFAVP-NGYNAPQQV--------------------------- 57
LaAP1|Q93VM7|/1-460 31 --LIEKPVDMPLDSDAFAIP-PGYNAPQQV--------------------------- 57
PvPAP2|Q764C1|/1-457 38 ---EWPAVDIPLDHEAFAVP-KGYNAPQQV--------------------------- 63
LlAP2|Q8L6L1|/1-463 33 --LIQNPVDMPLDSDAFAIP-PGYNAPQQV--------------------------- 59
AtPAP10|Q9SIV9|/1-468 37 ---LEATVDMPLDSDVFRVP-CGYNAPQQV--------------------------- 62
PvPAP1|P80366|/1-459 32 ---TNKNRDMPLDSDVFRVP-PGYNAPQQV--------------------------- 57
TaACP|C4PKL1|/1-477 33 ---EFPSTDMPLDSEWFATP-KGYNAPQQV--------------------------- 58
AtPAP6|Q9C510|/1-466 30 ---ALPSIEMSLDT--FPSP-GGYNTPEQV--------------------------- 53
AcPAP|Q93WP4|/1-481 38 ---EFPAVDIPIDSKEFAVP-KNQFSPQQV--------------------------- 63
AoPAP32|Q9XF09|/1-470 38 ---LAAATDMPLNSDVFRVP-PGYNAPQQV--------------------------- 63
StPAP3|Q6J5M8|/1-477 31 ---QFPSVDIPLENEVLSVP-NGYNAPQQV--------------------------- 56
IbPAP1|Q9SE00|/1-473 38 ALPNAEDVDMPWDSDVFAVP-SGYNAPQQV--------------------------- 66
AtPAP26|Q949Y3|/1-475 32 ---EWPAVDIPLDHHVFKVP-KGYNAPQQV--------------------------- 57
RcPAP3|B9SXP6|/1-488 38 ---EWPSIDIPLDNEVFAVP-KGYNAPQQV--------------------------- 63
LlAP1|Q8L5E1|/1-477 34 ---EFPSTDIPLDHEVFAVP-KGYNAPQQV--------------------------- 59
GmPAP3|Q6YGT9|/1-512 70 ---EWPAVDIPLDHEAFAVP-KGYNAPQQV--------------------------- 95
LaAP2|Q9XJ24|/1-638 31 ---SERAIDMALDSDVFHVP-RGYNAPQQV--------------------------- 56
LlPPD4|Q8VXF4|/1-629 75 --TDWVTVELESPKPSIDDW-VGVFSPAKFDSETCPGTENHVGHIEAPYVCTAPIK 127
LlPPD1|Q8VX11|/1-615 62 HYTEWVTLQYSNNKPSIDDW-IGVFSPANFSASTCPGENKMT---NPPFLCSAPIK 113
LlPPD2|Q8VXF6|/1-612 61 --AEWVTLEYSSPIPSIDDW-IGVFSPSNFSASACPAENRRV---YPPLLCSAPIK 110
TnPAP1|Q4RLR4|/1-378 24 -----------TRT-----------QPEQV--------------------------- 31
HsPAP7|Q6ZNF0|/1-438 22 ---------QGSLGAPSA----------APEQV--------------------------- 35
CePAP3|Q9NAM9|/1-418 16 -----------SD-----------GKAVEQV--------------------------- 24
MmPAP7|Q8BX37|/1-438 21 --VQG-----AQEYPHV--------TPEQI--------------------------- 35
DmPAP1|Q9VZ56|/1-458 22 -----------IRSTPIDQDVDIVHYQPEQV--------------------------- 41
DmPAP2|Q9VZ58|/1-450 22 -----------IRSTPIDQDVDIVHYQPEQV--------------------------- 41
AmPAP|A0A087ZWE4|/1-438 17 -----------VCN---------VIYYQPEAV--------------------------- 28
CePAP1|O01320|/1-419 5 ---------DEKLEKRSSSSS-LDRFLPDLP--------------------------- 25
DmPAP3|Q9VZ57|/1-453 22 -----------IRSTPIDQDVDIVHYQPEQV--------------------------- 41
AgPAP|Q7PUN5|/1-463 23 -----------CNGQ-------VFYYQPEQV--------------------------- 35

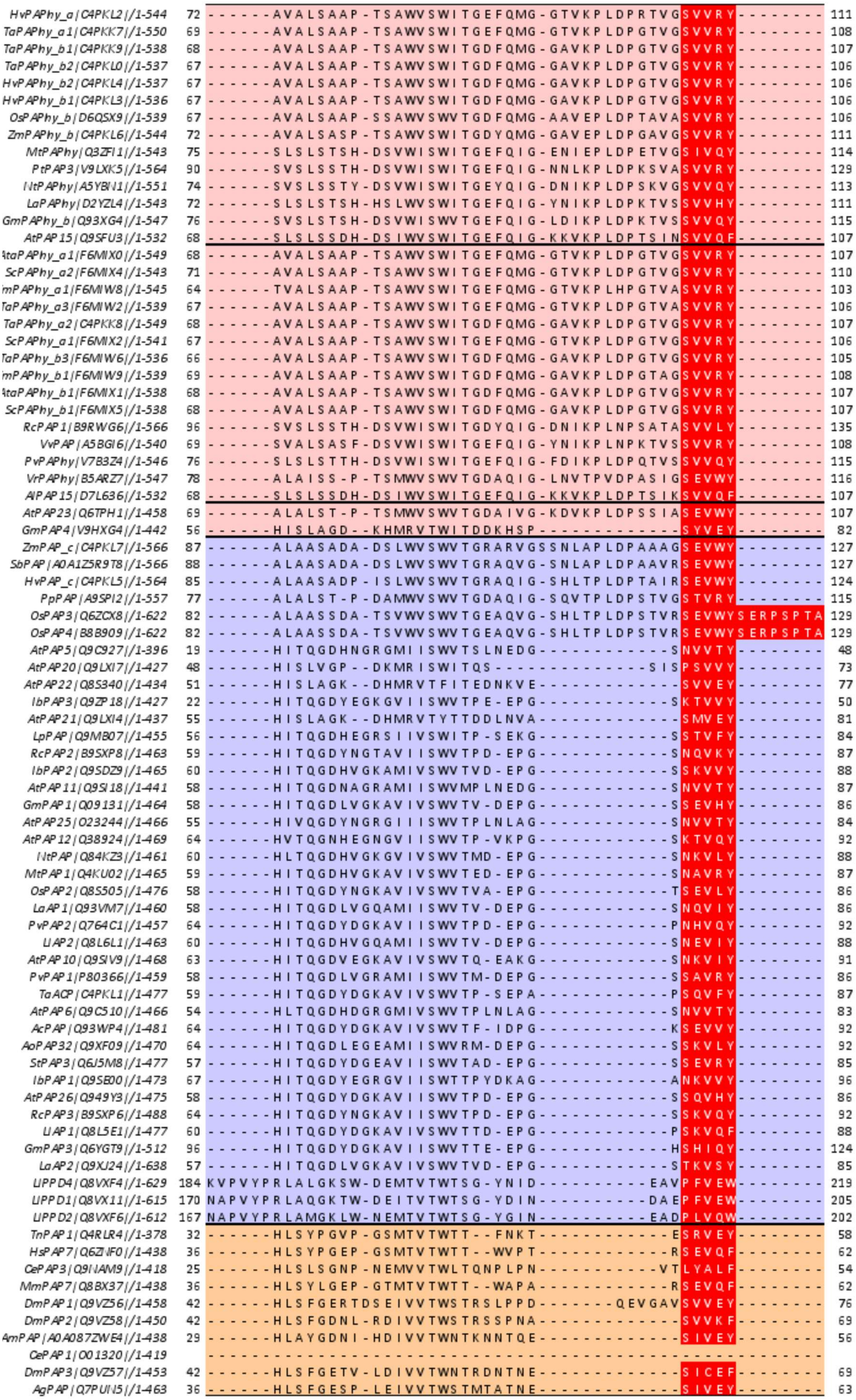

HvPAPhy_a|C4PKL2|/1-544	112	---GLAA-DS-----L-VREA-TGDALVYSQL---YPFEGLHNYTSGIIHHVRLQG	153
TaPAPhy_a1|C4PKK7|/1-550	109	---GLAA-DS-----L-VRQA-SGDALVYSQL---YPFEGLQNYTSGIIHHVRLQG	150
TaPAPhy_b1|C4PKK9|/1-538	108	---GLAA-DS-----L-AREA-TGEALVYSQL---YPFEGLQNYTSGIIHHVRILG	149
TaPAPhy_b2|C4PKL0|/1-537	107	---GLAA-DS-----L-VREA-TGDALVYSQL---YPFEGLQNYTSGIIHHVRLQG	148
HvPAPhy_b2|C4PKL4|/1-537	107	---GLAA-DS-----V-VREA-TGDALVYSQL---YPFEGLQNYTSGIIHHVRLQG	148
HvPAPhy_b1|C4PKL3|/1-536	107	---GLAA-DS-----V-VREA-TGDALVYSQL---YPFEGLQNYTSGIIHHVRLQG	148
OsPAPhy_b|D6QSX9|/1-539	107	---GLAA-DS-----L-VRRA-TGDALVYSQL---YPFDGLLNYTSAIIHHVRLQG	148
ZmPAPhy_b|C4PKL6|/1-544	112	---GLAA-DA-----L-DHEA-TGESLVYSQL---YPFEGLQNYTSGIIHHVRLQG	153
MtPAPhy|Q3ZFI1|/1-543	115	---GRFG-RS-----M-NGQA-VGYSLVYSQL---YPFEGLQNYTSGIIHHVRLTG	156
PtPAP3|V9LXK5|/1-564	130	---GTRR-SQ-----L-NRKA-TGRSLVYSQL---YPFLGLQNYTSGIIHHVRLTG	171
NtPAPhy|A5YBN1|/1-551	114	---GKDK-SS-----L-RHKA-IGESLIYNQL---YPFEGLQNYTSGIIHHVQLTG	155
LaPAPhy|D2YZL4|/1-543	112	---GTSR-TA-----L-VREA-RGQSLIYNQL---NPYEGLQNYTSGIIHHVQLRG	153
GmPAPhy_b|Q93XG4|/1-547	116	---GTSR-FE-----L-VHEA-RGQSLIYNQL---YPFEGLQNYTSGIIHHVQLKG	157
AtPAP15|Q9SFU3|/1-532	108	---GTLR-HS-----L-SHEA-KGHSLVYSQL---YPFDGLLNYTSGIIHHVRITG	149
AtaPAPhy_a1|F6MIX0|/1-549	108	---GLAA-DS-----L-VRQA-SGDALVYSQL---YPFEGLQNYTSGIIHHVRLQG	149
ScPAPhy_a2|F6MIX4|/1-543	111	---GLAA-DS-----L-VRVA-TGDALVYSQL---YPFEGLQNYTSGIIHHVRLQG	152
TmPAPhy_a1|F6MIW8|/1-545	104	---GLAA-DS-----L-VREA-TGDALVYSQL---YPFEGLQNYTSGIIHHVRLQG	145
TaPAPhy_a3|F6MIW2|/1-539	107	---GLAA-DS-----L-VRQA-TGDALVYSQL---YPFEGLQNYTSGIIHHVRLQG	148
TaPAPhy_a2|C4PKK8|/1-549	108	---GLAA-DS-----L-VREA-TGDALVYSQL---YPFEGLQNYTSGIIHHVRLQG	149
ScPAPhy_a1|F6MIX2|/1-541	107	---GLAA-DS-----L-VRVA-TGDALVYSQL---YPFEGLQNYTSGIIHHVRLQG	148
TaPAPhy_b3|F6MIW6|/1-536	106	---GLAA-DS-----L-VREA-TGDALVYSQL---YPFEGLQNYTSGIIHHVRLQG	147
TmPAPhy_b1|F6MIW9|/1-539	109	---GLAA-DS-----L-VREA-TGDALVYSQL---YPFEGLQNYTSGIIHHVRLQG	150
AtaPAPhy_b1|F6MIX1|/1-538	108	---GLAA-DS-----L-AREA-TGEALVYSQL---YPFEGLQNYTSGIIHHVRILG	149
ScPAPhy_b1|F6MIX5|/1-538	108	---GLAA-DS-----L-VREA-TGDVLVYSQL---YPFEGLQNYTSGIIHHVRLQG	149
RcPAP1|B9RWG6|/1-566	136	---GRSI-FP-----L-THQA-TGYSLVYNQL---YPFEGLKNYTSGVIHHVRLTG	177
VvPAP|A5BGI6|/1-540	109	---GTLR-YP-----L-RRKV-MGYSLVYNQL---YPFEGLQNYTSGIIHHVRLAG	150
PvPAPhy|V7B3Z4|/1-546	116	---GTSR-FD-----L-VHEA-RGQSLIYSQL---YPFDGLQNYTSGIIHHVRLIG	157
VrPAPhy|B5ARZ7|/1-547	117	---GKES-GK-----Y-TSVG-KGDSVVYSQL---YPFEGLWNYTSGIIHHVKLEG	158
AlPAP15|D7L636|/1-532	108	---GTLR-HS-----L-SHEA-KGHSLVYSQL---YPFDGLLNYTSGIIHHVRITG	149
AtPAP23|Q6TPH1|/1-458	108	---GKEK-GN-----Y-MLKK-KGNATVYSQL---YPSDGLLNYTSGIIHHVLIDG	149
GmPAP4|V9HXG4|/1-442	83	---GTLP-GR-----Y-DSIA-EGECTSYNYL----------LYSSGKIHHAVIGP	117
ZmPAP_c|C4PKL7|/1-566	128	---GERS-AADAAS-Y-PHVV-TGSAEVYSQL---YPYPGLLNYTSGAIHHVRLRG	173
SbPAP|A0A1Z5R9T8|/1-566	128	---GERS-AADAAS-Y-PHVA-TGSAEVYSQL---YPYPGLLNYTSGAIHHVRLRG	173
HvPAP_c|C4PKL5|/1-564	125	---GERP-ASADTVGH-PHVA-RGSAEVYSQL---YPYPGLLNYTSGVIHHVRLVG	171
PpPAP|A9SPI2|/1-557	116	---GLAP-GV-----Y-TFESPPGTSLVYSQL---YNFPGLRNYTSGIIHHVRLTG	158
OsPAP3|Q6ZCX8|/1-622	130	AAAGDVS-GH-----Y-PHVA-RGKAEVYSQL---YPYPGLLNYTSGAIHHVRLRG	174
OsPAP4|B8B909|/1-622	130	AAAGDVS-GH-----Y-PHVA-RGKAEVYSQL---YPYPGLLNYTSGAIHHVRLRG	174
AtPAP5|Q9C927|/1-396	49	---WIASSDG-----S-DNKSVIATTSSYRYF----------DYTSGYLHHAIIKE	85
AtPAP20|Q9LXI7|/1-427	74	---GTVS-GK-----Y-EGSA-NGTSSSYHYL---------LIYRSGQINDVVIGP	109
AtPAP22|Q8S340|/1-434	78	---GKQP-GK-----Y-DGKA-TGECTSYKYF----------FYKSGKIHHVKIGP	112
IbPAP3|Q9ZP18|/1-427	51	---WAEN-SS-----V-KRRA-DGVVVTYKYY----------NYTSGYIHHCTIKD	85
AtPAP21|Q9LXI4|/1-437	82	---GKHP-KK-----Y-DKKT-AGESTSYTYF----------FYNSGKIHHVKIGP	116
LpPAP|Q9MB07|/1-455	85	---GTSE-NK-----L-DQHA-EGTVTMYKFY----------TYTSGYIHHCVLTD	119
RcPAP2|B9SXP8|/1-463	88	---GKSE-KH-----Y-DSVA-EGT-------------------------------	101
IbPAP2|Q9SDZ9|/1-465	89	---WSEN-SQ-----H-KKVA-RGNIRTYTYF----------NYTSGYIHHCTIRN	123
AtPAP11|Q9SI18|/1-441	88	---WIASSDG-----SDNKNA-IATTSSYRYF----------NYTSGYLHHATIKK	124
GmPAP1|Q09131|/1-464	87	---WSEN-SD-----K-KKIA-EGKLVTYRFF----------NYSSGFIHHTTIRN	121
AtPAP25|O23244|/1-466	85	---WKAVDGD-----VKPKKK-RGHASTSSYR--------FYDYTSGFLHHATIKG	123
AtPAP12|Q38924|/1-469	93	---WCEN-EK-----S-RKQA-EATVNTYRFF----------NYTSGYIHHCLIDD	127
NtPAP|Q84KZ3|/1-461	89	---WEFN-SK-----I-KQIA-KGTVSTYKYH----------TYNSGYIHHCTIQN	123
MtPAP1|Q4KU02|/1-465	88	---WSKN-SK-----Q-KRLA-KGKIVTYRFF----------NYTSGFIHHTTIRN	122
OsPAP2|Q8S505|/1-476	87	---GKNE-HQ-----Y-DQRV-EGTVTNYTFY----------DYKSGYIHHCLVDG	121
LaAP1|Q93VM7|/1-460	87	---WSDS-SL-----Q-NFTA-EGEVFTYTYY----------NYTSGFIHHTTITN	121
PvPAP2|Q764C1|/1-457	93	---GTSE-SK-----F-QTSL-EGTVTNYTFY----------EYKSGYIHHCVIEG	127
LlAP2|Q8L6L1|/1-463	89	---WSNS-SL-----Q-NFTA-EGEVFTYTYY----------NYTSGFIHHTNITN	123
AtPAP10|Q9SIV9|/1-468	92	---WKEN-ST-----K-KHKA-HGKTNTYKFY----------NYTSGFIHHCPIRN	126
PvPAP1|P80366|/1-459	87	---WSEK-NG-----R-KRIA-KGKMSTYRFF----------NYSSGFIHHTTIRK	121
TaACP|C4PKL1|/1-477	88	---SKEE-NR-----Y-DQKA-EGTMTNYTFY----------DYKSGYIHHCLVDG	122
AtPAP6|Q9C510|/1-466	84	---WIATNGSDVKP-A-KKRA-HASTKSYRFY----------DYSSGFLHHATIKG	123
AcPAP|Q93WP4|/1-481	93	---GTSP-NS-----Y-DHSA-QGKTTNYTYY----------DYTSGYIHHCLLDK	127
AoPAP32|Q9XF09|/1-470	93	---WIDG-SN-----Q-KHSA-NGKITKYKYY----------NYTSGFIHHCTIRR	127
StPAP3|Q6J5M8|/1-477	86	---GLSE-GK-----Y-DVTV-EGTLNNYTFY----------KYESGYIHQCLVTG	120
IbPAP1|Q9SE00|/1-473	97	---WSEN-SK-----S-QKRA-MGTVVTYKYY----------NYTSAFIHHCTIKD	131
AtPAP26|Q949Y3|/1-475	87	---GAVQ-GK-----Y-EFVA-QGTYHNYTFY----------KYKSGFIHHCLVSD	121
RcPAP3|B9SXP6|/1-488	93	---GVSE-NK-----Y-DFIA-EGTARNYTFY----------QYKSGYIHQCLIDD	127
LlAP1|Q8L5E1|/1-477	89	---GTSE-NK-----F-QTSA-EGTVSNYTFY----------KYKSGYVHHCLIEG	123
GmPAP3|Q6YGT9|/1-512	125	---GTSE-NK-----F-QTSE-EGTVTNYTFH----------KYKSGYIHHCLIEG	159
LaAP2|Q9XJ24|/1-638	86	---WSDK-HS-----HDKKSA-HGKIVTYRFF----------NYTSGFIHH-TIKH	120
LlPPD4|Q8VXF4|/1-629	220	---GPTG-GR-----K-TRSP-AGTLTFDRNSLCGEPARTVGWRDPGFIHTSFLKE	264
LlPPD1|Q8VX11|/1-615	206	---GPKE-GN-----L-VKTP-AGTLTFDRNTMCGAPARTVGWRDPGYIHTSFLKE	250
LlPPD2|Q8VXF6|/1-612	203	---GPKG-GD-----H-IHSP-AGTLTFTKDSLCGAPARTVGWRDPGFIHTSYLKE	247
TnPAP1|Q4RLR4|/1-378	59	---GLLG-GR-----LFEKRA-KGESTLFVDS--------GVEKRKMFIHRVTLTG	96
HsPAP7|Q6ZNF0|/1-438	63	---GLQPSGP-----L-PLRA-QGTFVPFV--------DGGILRRKLYIHRVTLRK	100
CePAP3|Q9NAM9|/1-418	55	---GVSQ-DS-----L-RFTA-KGNTTGWADQ--------GKHKTMRYTHRATMQN	91
MmPAP7|Q8BX37|/1-438	63	---GSQLSGP-----L-PFRA-HGTARAFV--------DGGVLRRKLYIHRVTLRK	100
DmPAP1|Q9VZ56|/1-458	77	---GQLV-DGQVR--L-TQQA-RGKATKFV--------DGGHKQATQFIHRVTLRD	116
DmPAP2|Q9VZ58|/1-450	70	---SR---NY-----L-KDEP-IMVNGTWQRF-----VDGGKKARTQYIHNVELKD	107
AmPAP|A0A087ZWE4|/1-438	57	---GI---NG-----L-ILTA-TGNSTLFV--------DGGNEKQKQYIHRVWLKN	91
CePAP1|O01320|/1-419	26	-----QK-VK-------SRRK-MRKIMCFVGL-----------FGISTIFFIAVSN	56
DmPAP3|Q9VZ57|/1-453	70	---GI---DG-----L-HQRV-KATQMPTKFV------DGGAKKATQYIHRVTLSH	106
AgPAP|Q7PUN5|/1-463	64	---GI---GG-----L-ILSA-TGTETKFV--------DGGPAKRTQYIHRVVLRD	98

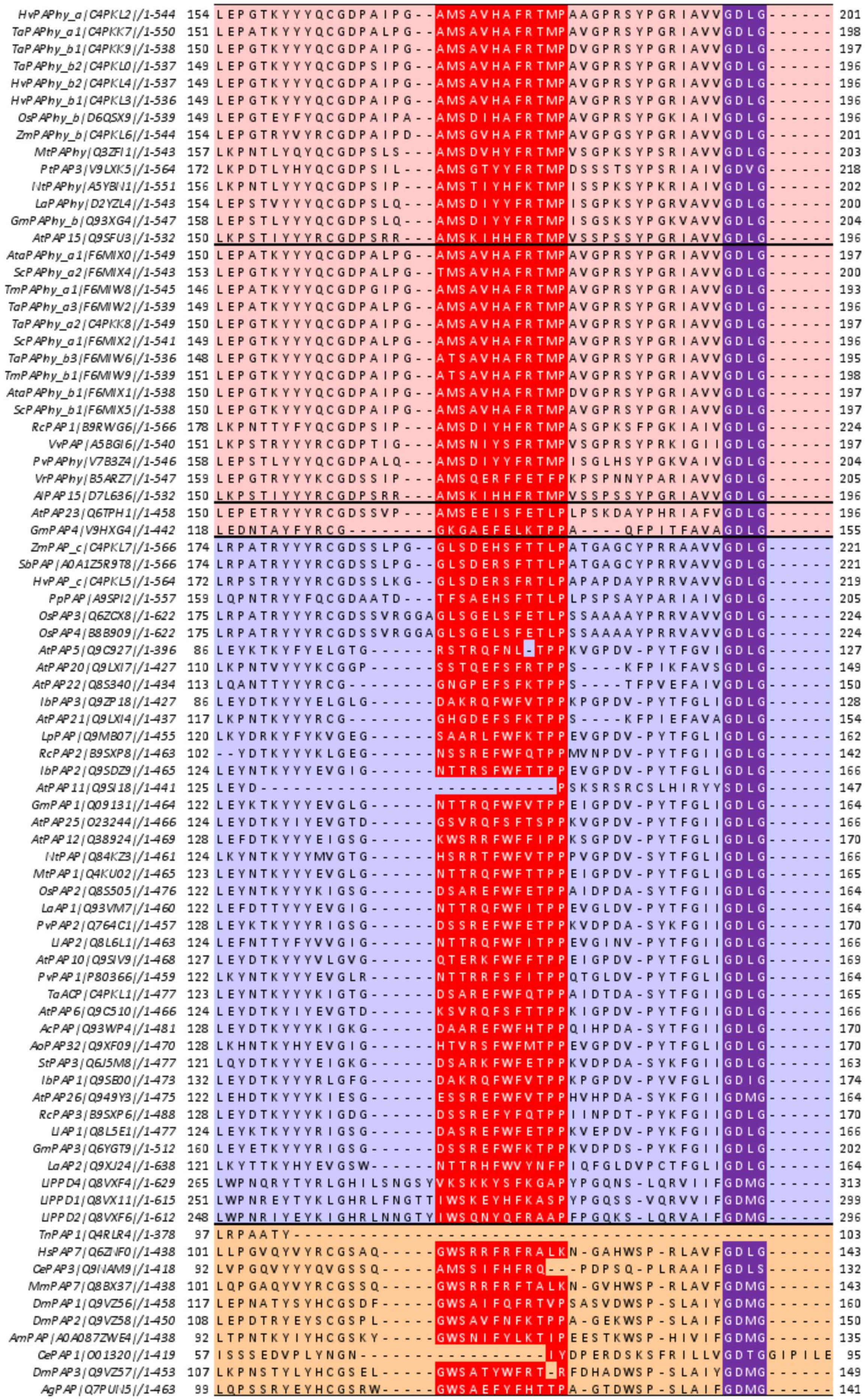

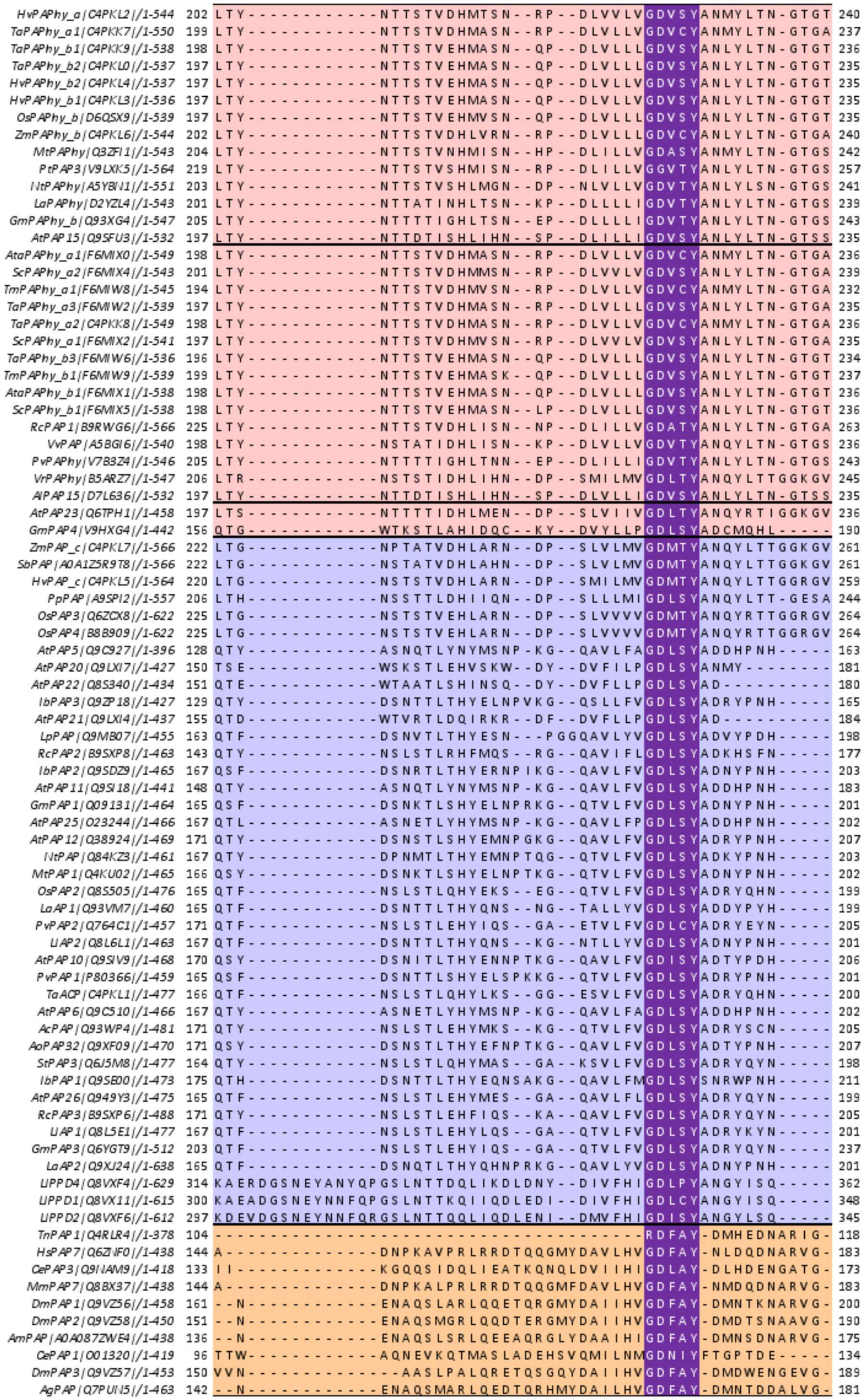
HvPAPhy_a|C4PKL2|/1-544 202 LTY------------NTTSTVDHMTSN--RP--DLVVLVGDVSYANMYLTN-GTGT 240
TaPAPhy_a1|C4PKK7|/1-550 199 LTY------------NTTSTVDHMASN--RP--DLVLVGDVCYANMYLTN-GTGA 237
TaPAPhy_b1|C4PKK9|/1-538 198 LTY------------NTTSTVEHMASN--QP--DLVLLLGDVSYANLYLTN-GTGT 236
TaPAPhy_b2|C4PKL0|/1-537 197 LTY------------NTTSTVEHMASN--QP--DLVLLLGDVSYANLYLTN-GTGT 235
HvPAPhy_b2|C4PKL4|/1-537 197 LTY------------NTTSTVEHMASN--QP--DLVLLVGDVSYANLYLTN-GTGT 235
HvPAPhy_b1|C4PKL3|/1-536 197 LTY------------NTTSTVEHMASN--QP--DLVLLVGDVSYANLYLTN-GTGT 235
OsPAPhy_b|D6QSX9|/1-539 197 LTY------------NTTSTVEHMVSN--QP--DLVLLLGDVSYANLYLTN-GTGT 235
ZmPAPhy_b|C4PKL6|/1-544 202 LTY------------NTTSTVDHLVRN--RP--DLVLLLGDVCYANLYLTN-GTGA 240
MtPAPhy|Q3ZFI1|/1-543 204 LTY------------NTTSTVNHMISN--HP--DLILLVGDASYANMYLTN-GTGS 242
PtPAP3|V9LXK5|/1-564 219 LTY------------NTTSTVSHMISN--RP--DLILLVGGVTYANLYLTN-GTGS 257
NtPAPhy|A5YBN1|/1-551 203 LTY------------NTTSTVSHLMGN--DP--NLVLLVGDVTYANLYLSN-GTGS 241
LaPAPhy|D2YZL4|/1-543 201 LTY------------NTTATINHLTSN--KP--DLLLLIGDVTYANLYLTN-GTGS 239
GmPAPhy_b|Q93XG4|/1-547 205 LTY------------NTTTTIGHLTSN--EP--DLLLLIGDVTYANLYLTN-GTGS 243
AtPAP15|Q9SFU3|/1-532 197 LTY------------NTTDTISHLIHN--SP--DLILLIGDVSYANLYLTN-GTSS 235
AtaPAPhy_a1|F6MIX0|/1-549 198 LTY------------NTTSTVDHMASN--RP--DLVLLVGDVCYANMYLTN-GTGA 236
ScPAPhy_a2|F6MIX4|/1-543 201 LTY------------NTTSTVDHMMSN--RP--DLVVLVGDVSYANLYLTN-GTGA 239
TmPAPhy_a1|F6MIW8|/1-545 194 LTY------------NTTSTVDHMVSN--RP--DLVLLVGDVCYANMYLTN-GTGA 232
TaPAPhy_a3|F6MIW2|/1-539 197 LTY------------NTTSTVDHMASN--RP--DLVLLLGDVSYANLYLTN-GTGA 235
TaPAPhy_a2|C4PKK8|/1-549 198 LTY------------NTTSTVDHMASN--RP--DLVLLVGDVCYANMYLTN-GTGA 236
ScPAPhy_a1|F6MIX2|/1-541 197 LTY------------NTTSTVDHMVSN--RP--DLVVLVGDVSYANLYLTN-GTGA 235
TaPAPhy_b3|F6MIW6|/1-536 196 LTY------------NTTSTVEHMASN--QP--DLVLLLGDVSYANLYLTN-GTGT 234
TmPAPhy_b1|F6MIW9|/1-539 199 LTY------------NTTSTVEHMASK--QP--DLVLLLGDVSYANLYLTN-GTGT 237
AtaPAPhy_b1|F6MIX1|/1-538 198 LTY------------NTTSTVEHMASN--QP--DLVLLLGDVSYANLYLTN-GTGT 236
ScPAPhy_b1|F6MIX5|/1-538 198 LTY------------NTTSTVEHMASN--LP--DLVLLLGDVSYANLYLTN-GTGT 236
RcPAP1|B9RWG6|/1-566 225 LTY------------NTTSTVDHLISN--NP--DLILLVGDATYANLYLTN-GTGA 263
VvPAP|A5BGI6|/1-540 198 LTY------------NSTATIDHLISN--KP--DLVLLVGDVTYANQYLTN-GTGS 236
PvPAPhy|V7B3Z4|/1-546 205 LTY------------NTTTTIGHLTNN--EP--DLILLIGDVTYANLYLTN-GTGS 243
VrPAPhy|B5ARZ7|/1-547 206 LTR------------NSTSTIDHLIHN--DP--SMILMVGDLTYANQYLTTGGKGV 245
AlPAP15|D7L636|/1-532 197 LTY------------NTTDTISHLIHN--SP--DLVLLIGDVSYANLYLTN-GTSS 235
AtPAP23|Q6TPH1|/1-458 197 LTS------------NTTTTIDHLMEN--DP--SLVIIVGDLTYANQYRTIGGKGV 236
GmPAP4|V9HXG4|/1-442 156 QTG------------WTKSTLAHIDQC--KY--DVYLLPGDLSYADCMQHL----- 190
ZmPAP_c|C4PKL7|/1-566 222 LTG------------NPTATVDHLARN--DP--SLVLMVGDMTYANQYLTTGGKGV 261
SbPAP|A0A1Z5R9T8|/1-566 222 LTG------------NSTATVDHLAHN--DP--SLVLMVGDMTYANQYLTTGGKGV 261
HvPAP_c|C4PKL5|/1-564 220 LTG------------NSTSTVDHLARN--DP--SMILMVGDMTYANQYLTTGGRGV 259
PpPAP|A9SPI2|/1-557 206 LTH------------NSSTTLDHIIQN--DP--SLLLMIGDLSYANQYLTT-GESA 244
OsPAP3|Q6ZCX8|/1-622 225 LTG------------NSTSTVEHLARN--DP--SLVVVVGDMTYANQYRTTGGRGV 264
OsPAP4|B8B909|/1-622 225 LTG------------NSTSTVEHLARN--DP--SLVVVVGDMTYANQYRTTGGRGV 264
AtPAP5|Q9C927|/1-396 128 QTY------------ASNQTLYNYMSNP-KG--QAVLFAGDLSYADDHPNH----- 163
AtPAP20|Q9LXI7|/1-427 150 TSE------------WSKSTLEHVSKW--DY--DVFILPGDLSYANMY-------- 181
AtPAP22|Q8S340|/1-434 151 QTE------------WTAATLSHINSQ--DY--DVFLLPGDLSYAD---------- 180
IbPAP3|Q9ZP18|/1-427 129 QTY------------DSNTTLTHYELNPVKG--QSLLFVGDLSYADRYPNH----- 165
AtPAP21|Q9LXI4|/1-437 155 QTD------------WTVRTLDQIRKR--DF--DVFLLPGDLSYAD---------- 184
LpPAP|Q9MB07|/1-455 163 QTF------------DSNVTLTHYESN---PGGQAVLYVGDLSYADVYPDH----- 198
RcPAP2|B9SXP8|/1-463 143 QTY------------NSLSTLRHFMQS--RG--QAVIFLGDLSYADKHSFN----- 177
IbPAP2|Q9SDZ9|/1-465 167 QSF------------DSNRTLTHYERNPIKG--QAVLFVGDLSYADNYPNH----- 203
AtPAP11|Q9SI18|/1-441 148 QTY------------ASNQTLYNYMSNP-KG--QAVLFVGDLSYADDHPNH----- 183
GmPAP1|Q09131|/1-464 165 QSF------------DSNKTLSHYELNPRKG--QTVLFVGDLSYADNYPNH----- 201
AtPAP25|O23244|/1-466 167 QTL------------ASNETLYHYMSNP-KG--QAVLFPGDLSYADDHPNH----- 202
AtPAP12|Q38924|/1-469 171 QTY------------DSNSTLSHYEMNPGKG--QAVLFVGDLSYADRYPNH----- 207
NtPAP|Q84KZ3|/1-461 167 QTY------------DPNMTLTHYEMNPTQG--QTVLFVGDLSYADKYPNH----- 203
MtPAP1|Q4KU02|/1-465 166 QSY------------DSNKTLSHYELNPTKG--QTVLFVGDLSYADNYPNH----- 202
OsPAP2|Q8S505|/1-476 165 QTF------------NSLSTLQHYEKS--EG--QTVLFVGDLSYADRYQHN----- 199
LaAP1|Q93VM7|/1-460 165 QTF------------DSNTTLTHYQNS--NG--TALLYVGDLSYADDYPYH----- 199
PvPAP2|Q764C1|/1-457 171 QTF------------NSLSTLEHYIQS--GA--ETVLFVGDLCYADRYEYN----- 205
LlAP2|Q8L6L1|/1-463 167 QTF------------DSNTTLTHYQNS--KG--NTLLYVGDLSYADNYPNH----- 201
AtPAP10|Q9SIV9|/1-468 170 QSY------------DSNITLTHYENNPTKG--QAVLFVGDISYADTYPDH----- 206
PvPAP1|P80366|/1-459 165 QSF------------DSNTTLSHYELSPKKG--QTVLFVGDLSYADRYPNH----- 201
TaACP|C4PKL1|/1-477 166 QTF------------NSLSTLQHYLKS--GG--ESVLFVGDLSYADRYQHN----- 200
AtPAP6|Q9C510|/1-466 167 QTY------------ASNETLYHYMSNP-KG--QAVLFAGDLSYADDHPNH----- 202
AcPAP|Q93WP4|/1-481 171 QTY------------NSLSTLEHYMKS--KG--QTVLFVGDLSYADRYSCN----- 205
AoPAP32|Q9XF09|/1-470 171 QSY------------DSNSTLTHYEFNPTKG--QAVLFVGDLSYADTYPNH----- 207
StPAP3|Q6J5M8|/1-477 164 QTY------------NSLSTLQHYMAS--GA--KSVLFVGDLSYADRYQYN----- 198
IbPAP1|Q9SE00|/1-473 175 QTH------------DSNTTLTHYEQNSAKG--QAVLFMGDLSYSNRWPNH----- 211
AtPAP26|Q949Y3|/1-475 165 QTF------------NSLSTLEHYMES--GA--QAVLFLGDLSYADRYQYN----- 199
RcPAP3|B9SXP6|/1-488 171 QTY------------NSLSTLEHFIQS--KA--QAVLFVGDLSYADRYQYN----- 205
LlAP1|Q8L5E1|/1-477 167 QTF------------NSLSTLEHYLQS--GA--QTVLFVGDLSYADRYKYN----- 201
GmPAP3|Q6YGT9|/1-512 203 QTF------------NSLSTLEHYIQS--GA--QTVLFVGDLSYADRYQYN----- 237
LaAP2|Q9XJ24|/1-638 165 QTF------------DSNQTLTHYQHNPRKG--QAVLYVGDLSYADNYPNH----- 201
LlPPD4|Q8VXF4|/1-629 314 KAERDGSNEYANYQPGSLNTTDQLIKDLDNY--DIVFHIGDLPYANGYISQ----- 362
LlPPD1|Q8VX11|/1-615 300 KAEADGSNEYNNFQPGSLNTTKQIIQDLEDI--DIVFHIGDLCYANGYISQ----- 348
LlPPD2|Q8VXF6|/1-612 297 KDEVDGSNEYNNFQRGSLNTTQQLIQDLENI--DMVFHIGDISYANGYLSQ----- 345
TnPAP1|Q4RLR4|/1-378 104 ------------------------------------RDFAY-DMHEDNARIG- 118
HsPAP7|Q6ZNF0|/1-438 144 A--------------DNPKAVPRLRRDTQQGMYDAVLHVGDFAY-NLDQDNARVG- 183
CePAP3|Q9NAM9|/1-418 133 II-------------KGQQSIDQLIEATKQNQLDVIIHIGDLAY-DLHDENGATG- 173
MmPAP7|Q8BX37|/1-438 144 A--------------DNPKALPRLRRDTQQGMFDAVLHVGDFAY-NMDQDNARVG- 183
DmPAP1|Q9VZ56|/1-458 161 --N------------ENAQSLARLQQETQRGMYDAIIHVGDFAY-DMNTKNARVG- 200
DmPAP2|Q9VZ58|/1-450 151 --N------------ENAQSMGRLQQDTERGMYDAIIHVGDFAY-DMDTSNAAVG- 190
AmPAP|A0A087ZWE4|/1-438 136 --N------------ENAQSLSRLQEEAQRGLYDAAIHIGDFAY-DMNSDNARVG- 175
CePAP1|O01320|/1-419 96 TTW------------AQNEVKQTMASLADEHSVQMILNMGDNIYFTGPTDE----- 134
DmPAP3|Q9VZ57|/1-453 150 VVN---------------AASLPALQRETQSGQYDAIIHVGDFAY-DMDWENGEVG- 189
AgPAP|Q7PUN5|/1-463 142 --N------------ENAQSMARLQEDTQRHMYDAILHVGDFAY-DMNTDDALVG- 181

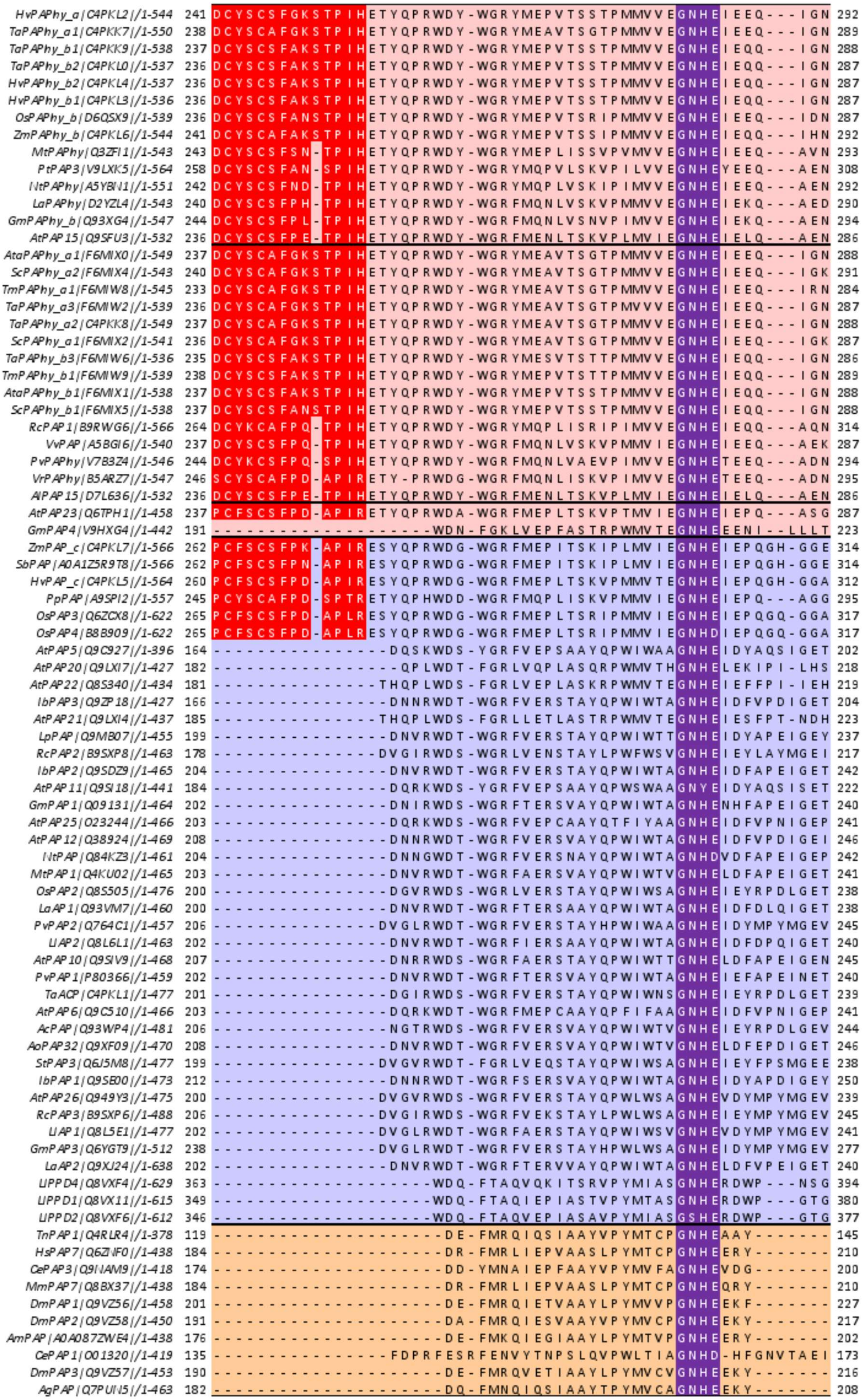

```
HvPAPhy_a|C4PKL2|/1-544      293 KTFAAYRS----------RFAFPSAESGSFSPFYY----SFDAGGIHFIMLGA--Y 332
TaPAPhy_a1|C4PKK7|/1-550     290 KTFAAYRS----------RFAFPSTESGSFSPFYY----SFDAGGIHFLMLGA--Y 329
TaPAPhy_b1|C4PKK9|/1-538     289 KTFAAYSA----------RFAFPSMESESFSPFYY----SFDAGGIHFIMLAA--Y 328
TaPAPhy_b2|C4PKL0|/1-537     288 KTFAAYSA----------RFAFPSMESESFSPFYY----SFDAGGIHFIMLAA--Y 327
HvPAPhy_b2|C4PKL4|/1-537     288 KTFAAYSA----------RFAFPSKESESFSPFYY----SFDVGGIHFIMLAA--Y 327
HvPAPhy_b1|C4PKL3|/1-536     288 KTFAAYSA----------RFAFPSKESESFSPFYY----SFDVGGIHFIMLAA--Y 327
OsPAPhy_b|D6QSX9|/1-539      288 KTFASYSS----------RFSFPSTESGSFSPFYY----SFDAGGIHFVMLAA--Y 327
ZmPAPhy_b|C4PKL6|/1-544      293 RTFAAYSS----------RFAFPSEESGSSSPFYY----SFDAGGIHFVMLAS--Y 332
MtPAPhy|Q3ZFI1|/1-543        294 KTFVAYSS----------RFAFPSEESGSSSTLYY----SFNAGGIHFIMLGS--Y 333
PtPAP3|V9LXK5|/1-564         309 RTFLAYTS----------RFAFPSKESGSLSKFYY----SFNAGGIHFIMLGA--Y 348
NtPAPhy|A5YBN1|/1-551        293 QTFAAYRS----------RFAFPSKESGSSSPFYY----SFNAGGIHFIMLGG--Y 332
LaPAPhy|D2YZL4|/1-543        291 KQFVAYSS----------RFAFPSEESGSSSTFYY----SFNAGGIHFIMLGA--Y 330
GmPAPhy_b|Q93XG4|/1-547      295 RTFVAYSS----------RFAFPSQESGSSSTFYY----SFNAGGIHFIMLGA--Y 334
AtPAP15|Q9SFU3|/1-532        287 KTFEAYSS----------RFAFPFNESGSSSTLYY----SFNAGGIHFVMLGA--Y 326
AtaPAPhy_a1|F6MIX0|/1-549    289 KTFAAYRS----------RFAFPSTESGSFSPFYY----SFDAGGIHFLMLGA--Y 328
ScPAPhy_a2|F6MIX4|/1-543     292 KTFEAYRS----------RFAFPSAENGSFSPFYY----SFDAGGIHFIMLAA--Y 331
TmPAPhy_a1|F6MIW8|/1-545     285 RTFAAYRS----------RFAFPSTESGSFSPFYY----SFDAGGIHFVMLAA--Y 324
TaPAPhy_a3|F6MIW2|/1-539     288 KTFAAYRS----------RFAFPSTESGSFSPFYY----SFDAGGIHFVMLGA--Y 327
TaPAPhy_a2|C4PKK8|/1-549     289 KTFAAYRS----------RFAFPSTESGSFSPFYY----SFDAGGIHFLMLGA--Y 328
ScPAPhy_a1|F6MIX2|/1-541     288 KTFEAYRS----------RFAFPSAESGSFSPFYY----SFDAGGIHFIMLAA--Y 327
TaPAPhy_b3|F6MIW6|/1-536     287 KTFAAYSA----------RFAFPSKESDSFSPFYY----SFDAGGIHFIMLAA--Y 326
TmPAPhy_b1|F6MIW9|/1-539     290 KTFAAYSA----------RFAFPSKESDSFSPFYY----SFDAGGIHFIMLAA--Y 329
AtaPAPhy_b1|F6MIX1|/1-538    289 KTFAAYSA----------RFAFPSMESESFSPFYY----SFDAGGIHFIMLAA--Y 328
ScPAPhy_b1|F6MIX5|/1-538     289 KTFAAYSA----------RFAFPSKESESFSPFYY----SFDAGGIHFIMLAA--Y 328
RcPAP1|B9RWG6|/1-566         315 QTFAAYSS----------RFAFPSKESGSPSTFYY----SFNAGGIHFVMLGA--Y 354
VvPAP|A5BGI6|/1-540          288 KNFVAYSS----------RFAFPSKESGSASTFYY----SFNAGGIHFIMLGA--Y 327
PvPAPhy|V7B3Z4|/1-546        295 RTFVAYSS----------RFAFPSEESGSSSTLYY----SFNAGGIHFIMLGA--Y 334
VrPAPhy|B5ARZ7|/1-547        296 KTFVAYSS----------RFAFPSEESGSLSTLYY----SFNAGGIHFIMLGA--Y 335
AlPAP15|D7L636|/1-532        287 KTFEAYSS----------RFAFPFKESGSSSTLYY----SFNAGGIHFVMLGA--Y 326
AtPAP23|Q6TPH1|/1-458        288 ITFKSYSE----------RFAVPASESGSNSNLYY----SFDAGGVHFVMLGA--Y 327
GmPAP4|V9HXG4|/1-442         224 DEFVSYNS----------RWKMPFEESGSTSNLYY----SFEVAGVHVIMLGS--Y 263
ZmPAP_c|C4PKL7|/1-566        315 VTFASYLA----------RVAVPSKESGSNTKFYY----SFNAGGIHFIMLGA--Y 354
SbPAP|A0A1Z5R9T8|/1-566      315 VTFASYLA----------RFAVPSNESGSNTKFYY----SFNAGGIHFIMLGA--Y 354
HvPAP_c|C4PKL5|/1-564        313 VTFASYLA----------RFAVPSEESGSNTKFYY----SFNAGGIHFIMLGA--Y 352
PpPAP|A9SPI2|/1-557          296 KSFVAYES----------RFSVPSQESGSNSKLYY----SFDAGGIHFVMLGG--Y 335
OsPAP3|Q6ZCX8|/1-622         318 VTFASYLA----------RFAVPSEESGSNTKFYY----SFNAGGIHFIMLGA--Y 357
OsPAP4|B8B909|/1-622         318 VTFASYLA----------RFAVPSEESGSNTKFYY----SFNAGGIHFIMLGA--Y 357
AtPAP5|Q9C927|/1-396         203 QPFKPYKN----------RYHVPYRASQN--------------------------- 221
AtPAP20|Q9LXI7|/1-427        219 NPFTAYNK----------RWRMPFEESGSSSNLYY----SFNVYGVHIIMLGS--Y 258
AtPAP22|Q8S340|/1-434        220 TTFKSYNA----------RWLMPHTESFSTSNLYY----SFDVAGVHTVMLGS--Y 259
IbPAP3|Q9ZP18|/1-427         205 VPFKPFTH----------RFFMPFESSGSTSPLWY----SIKRASAHIIVMSS--Y 244
AtPAP21|Q9LXI4|/1-437        224 ISFKSYNA----------RWLMPHAESLSHSNLYY----SFDVAGVHTVMLGS--Y 263
LpPAP|Q9MB07|/1-455          238 VPFKPFTH----------RYHVPHKSSGSGSPFWY----SIKRASAYIIVLAS--Y 277
RcPAP2|B9SXP8|/1-463         218 IPFKNYVY----------RYPTPYMASNSSSPLWY----AIRRASAHIIVLNS--Y 257
IbPAP2|Q9SDZ9|/1-465         243 KPFKPFTK----------RYHVPYKASGSTETFWY----PIKRASAYIIVLSS--Y 282
AtPAP11|Q9SI18|/1-441        223 QPFKPYKN----------RYHVPYKASQSTSPLWY----SIKRASTYIIVLSS--Y 262
GmPAP1|Q09131|/1-464         241 VPFKPYTH----------RYHVPYKASQSTSPFWY----SIKRASAHIIVLAS--Y 280
AtPAP25|O23244|/1-466        242 HAFKPYIH----------RYHNAYKASKSISPLWY----SIRRASAHIIVLSS--Y 281
AtPAP12|Q38924|/1-469        247 EPFKPFMN----------RYHTPHKASGSISPLWY----SIKRASAYIIVMSC--Y 286
NtPAP|Q84KZ3|/1-461          243 EPFRPYTN----------RYPVPYQASGSSSPLWY----SIKRASAYIIVLST--Y 282
MtPAP1|Q4KU02|/1-465         242 KPFKPYSH----------RYRTPYKASQSTSPFWY----SIKRASAHIIVLAS--Y 281
OsPAP2|Q8S505|/1-476         239 STFKPYLH----------RCHTPYLASKSSSPMWY----AVRRASAHIIVLSS--Y 278
LaAP1|Q93VM7|/1-460          239 QPFKPFST----------RYHTPYEASQSTEPFYY----SIKRGPAHVIVLAT--Y 278
PvPAP2|Q764C1|/1-457         246 VPFKNFLY----------RYTTPYLASNSSNPLWY----AVRRASAHIIVLSS--Y 285
LlAP2|Q8L6L1|/1-463          241 QPFKPFSN----------RYHTPYVASQSTEPYYY----SIKRGPAHIIVLAS--Y 280
AtPAP10|Q9SIV9|/1-468        246 RPFKPFTH----------RYRTPYRSSGSTEPFWY----SIKRGPAYIIVLAS--Y 285
PvPAP1|P80366|/1-459         241 EPFKPFSY----------RYHVPYEASQSTSPFWY----SIKRASAHIIVLSS--Y 280
TaACP|C4PKL1|/1-477          240 STFKPYLH----------RYSTPYLASKSSSPMWY----AVRRASAHIIVLSS--Y 279
AtPAP6|Q9C510|/1-466         242 HAFKPYTH----------RYPNAYKASQSTSPLWY----SVRRASAHIIVLSS--Y 281
AcPAP|Q93WP4|/1-481          245 FPFRAYLN----------RYPTPHLASASSSPLWY----SIRRASAHIIVLSS--Y 284
AoPAP32|Q9XF09|/1-470        247 KPFKPFSN----------RYRTPYKASNSTSPFFY----SIKRGPAHIIVLAS--Y 286
StPAP3|Q6J5M8|/1-477         239 VPFRSFLS----------RYPTPYRASKSSNPLWY----AIRRASAHIIVLSS--Y 278
IbPAP1|Q9SE00|/1-473         251 QPFVPFTN----------RYPTPHEASGSGDPLWY----AIKRASAHIIVLSS--Y 290
AtPAP26|Q949Y3|/1-475        240 TPFRNYLQ----------RYTTPYLASKSSSPLWY----AVRRASAHIIVLSS--Y 279
RcPAP3|B9SXP6|/1-488         246 TPFKSYLH----------RYPTPHLASKSSSPLWY----AIRCASAHIIVLSS--Y 285
LlAP1|Q8L5E1|/1-477          242 TPFKNFLN----------RYTTPYLASQSSSPLWY----AIRRASAHIIVLSS--Y 281
GmPAP3|Q6YGT9|/1-512         278 VPFKNYLY----------RYTTPYLASNSSSPLWY----AVRRASAHIIVLSS--Y 317
LaAP2|Q9XJ24|/1-638          241 KPFKPFTH----------RYPVPFKPSESTEPFWY----SIKRGPAHVIVLAS--Y 280
LlPPD4|Q8VXF4|/1-629         395 SFFDTPDSGGECGVLAETMYYFP---AENRAKFWY----KADYGMFRFCIADS--E 441
LlPPD1|Q8VX11|/1-615         381 SFYGNLDSGGECGVPAQTMFFVP---AENREKFWY----STDYGMFRFCIAHT--E 427
LlPPD2|Q8VXF6|/1-612         378 SFYENMDSGGECGVLAQIMFYVP---ASNRAKFWY----PIDYGMFRFRIADT--E 424
TnPAP1|Q4RLR4|/1-378         146 -NFSNYRN----------RFSMP----GQTESLWY----SWNLGPVHIISLSTEVY 182
HsPAP7|Q6ZNF0|/1-438         211 -NFSNYKA----------RFSMP----GDNEGLWY----SWDLGPAHIISFSTEVY 247
CePAP3|Q9NAM9|/1-418         201 -DFNHIKN----------RFTMPRNGVYDNNLFW-----SFTYGFVHIIAINSEYY 240
MmPAP7|Q8BX37|/1-438         211 -NFSNYKA----------RFSMP----GDNEGLWY----SWDLGPAHIISFSTEVY 247
DmPAP1|Q9VZ56|/1-458         228 -NFSNYRA----------RFSMP----GGTENMFY----SFDLGPVHFVGISTEVY 264
DmPAP2|Q9VZ58|/1-450         218 -NFSNYRA----------RFNMP----GETDSLWY----SFNLGPVHFVSFSTEVY 254
AmPAP|A0A087ZWE4|/1-438      203 -NFSNYRF----------RFTMP----GDSEGLWY----SFNIGPVHFIGIETEAY 239
CePAP1|O01320|/1-419         174 EYTKHSK-----------KWYFP--------SLYYKKSVEFNGTSIDFLMIDT--I 208
DmPAP3|Q9VZ57|/1-453         217 -NFSHYIN----------RFSMP----GGSDNMFY----SFDLGPVHFIGFSTEVY 253
AgPAP|Q7PUN5|/1-463          209 -NFSNYRA----------RFSMP----GGTENIMY----SFNLGPVHFIGFSTEVY 245
```

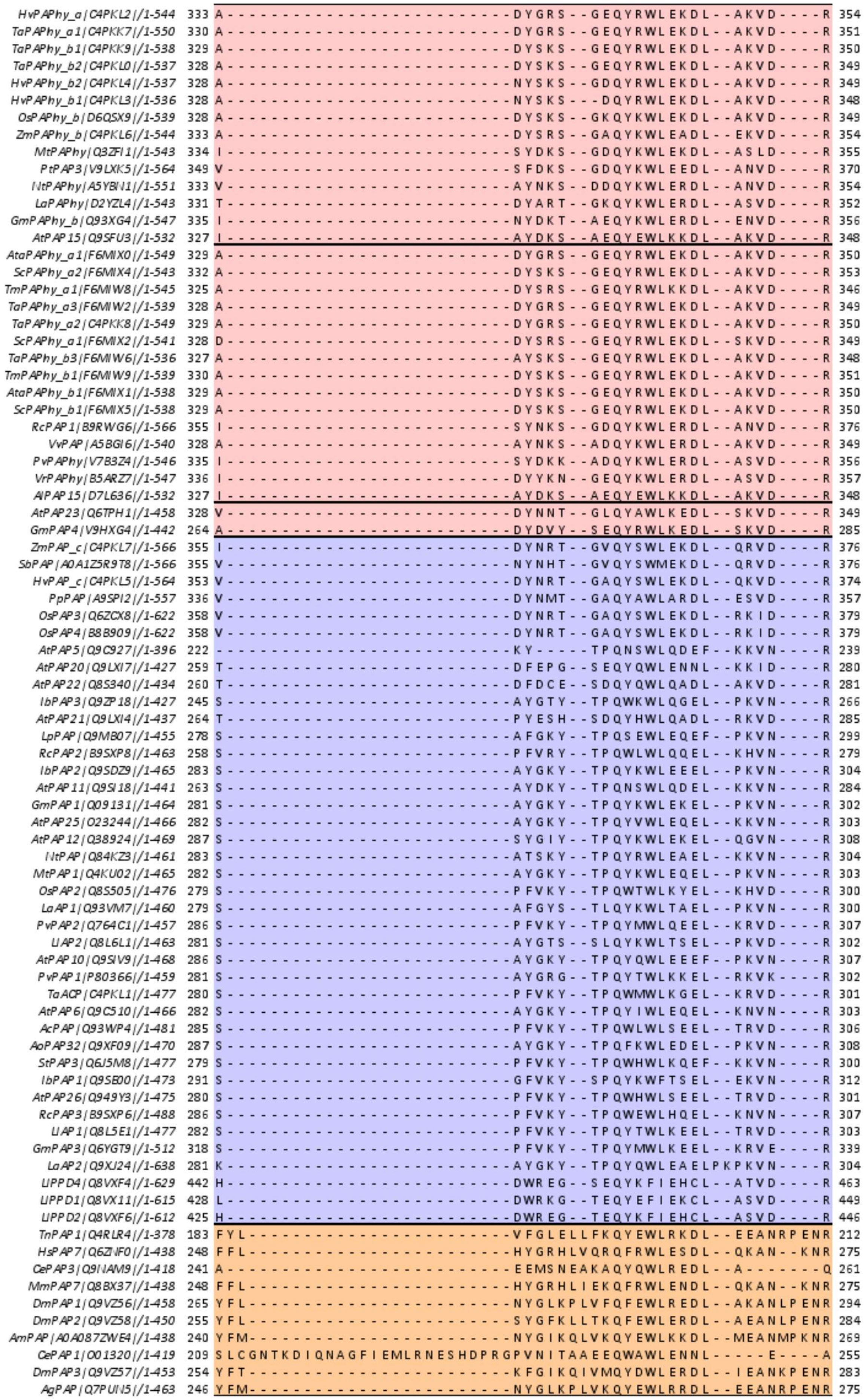

HvPAPhy_a|C4PKL2|/1-544 333 A------------------------DYGRS--GEQYRWLEKDL--AKVD----R 354
TaPAPhy_a1|C4PKK7|/1-550 330 A------------------------DYGRS--GEQYRWLEKDL--AKVD----R 351
TaPAPhy_b1|C4PKK9|/1-538 329 A------------------------DYSKS--GEQYRWLEKDL--AKVD----R 350
TaPAPhy_b2|C4PKL0|/1-537 328 A------------------------DYSKS--GEQYRWLEKDL--AKVD----R 349
HvPAPhy_b2|C4PKL4|/1-537 328 A------------------------NYSKS--GDQYRWLEKDL--AKVD----R 349
HvPAPhy_b1|C4PKL3|/1-536 328 A------------------------NYSKS---DQYRWLEKDL--AKVD----R 348
OsPAPhy_b|D6QSX9|/1-539 328 A------------------------DYSKS--GKQYKWLEKDL--AKVD----R 349
ZmPAPhy_b|C4PKL6|/1-544 333 A------------------------DYSRS--GAQYKWLEADL--EKVD----R 354
MtPAPhy|Q3ZFI1|/1-543 334 I------------------------SYDKS--GDQYKWLEKDL--ASLD----R 355
PtPAP3|V9LXK5|/1-564 349 V------------------------SFDKS--GDQYKWLEEDL--ANVD----R 370
NtPAPhy|A5YBN1|/1-551 333 V------------------------AYNKS--DDQYKWLERDL--ANVD----R 354
LaPAPhy|D2YZL4|/1-543 331 T------------------------DYART--GKQYKWLERDL--ASVD----R 352
GmPAPhy_b|Q93XG4|/1-547 335 I------------------------NYDKT--AEQYKWLERDL--ENVD----R 356
AtPAP15|Q9SFU3|/1-532 327 I------------------------AYDKS--AEQYEWLKKDL--AKVD----R 348
AtaPAPhy_a1|F6MIX0|/1-549 329 A------------------------DYGRS--GEQYRWLEKDL--AKVD----R 350
ScPAPhy_a2|F6MIX4|/1-543 332 A------------------------DYSKS--GEQYRWLEKDL--AKVD----R 353
TmPAPhy_a1|F6MIW8|/1-545 325 A------------------------DYSRS--GEQYRWLKKDL--AKVD----R 346
TaPAPhy_a3|F6MIW2|/1-539 328 A------------------------DYGRS--GEQYRWLEKDL--AKVD----R 349
TaPAPhy_a2|C4PKK8|/1-549 329 A------------------------DYGRS--GEQYRWLEKDL--AKVD----R 350
ScPAPhy_a1|F6MIX2|/1-541 328 D------------------------DYSRS--GEQYRWLEKDL--SKVD----R 349
TaPAPhy_b3|F6MIW6|/1-536 327 A------------------------AYSKS--GEQYRWLEKDL--AKVD----R 348
TmPAPhy_b1|F6MIW9|/1-539 330 A------------------------DYSKS--GEQYRWLEKDL--AKVD----R 351
AtaPAPhy_b1|F6MIX1|/1-538 329 A------------------------DYSKS--GEQYRWLEKDL--AKVD----R 350
ScPAPhy_b1|F6MIX5|/1-538 329 A------------------------DYSKS--GEQYRWLEKDL--AKVD----R 350
RcPAP1|B9RWG6|/1-566 355 I------------------------SYNKS--GDQYKWLERDL--ANVD----R 376
VvPAP|A5BGI6|/1-540 328 A------------------------AYNKS--ADQYKWLERDL--AKVD----R 349
PvPAPhy|V7B3Z4|/1-546 335 I------------------------SYDKK--ADQYKWLERDL--ASVD----R 356
VrPAPhy|B5ARZ7|/1-547 336 I------------------------DYYKN--GEQYKWLERDL--ASVD----R 357
AlPAP15|D7L636|/1-532 327 I------------------------AYDKS--AEQYEWLKKDL--AKVD----R 348
AtPAP23|Q6TPH1|/1-458 328 V------------------------DYNNT--GLQYAWLKEDL--SKVD----R 349
GmPAP4|V9HXG4|/1-442 264 A------------------------DYDVY--SEQYRWLKEDL--SKVD----R 285
ZmPAP_c|C4PKL7|/1-566 355 I------------------------DYNRT--GVQYSWLEKDL--QRVD----R 376
SbPAP|A0A1Z5R9T8|/1-566 355 V------------------------NYNHT--GVQYSWMEKDL--QRVD----R 376
HvPAP_c|C4PKL5|/1-564 353 V------------------------DYNRT--GAQYSWLEKDL--QKVD----R 374
PpPAP|A9SPI2|/1-557 336 V------------------------DYNMT--GAQYAWLARDL--ESVD----R 357
OsPAP3|Q6ZCX8|/1-622 358 V------------------------DYNRT--GAQYSWLEKDL--RKID----R 379
OsPAP4|B8B909|/1-622 358 V------------------------DYNRT--GAQYSWLEKDL--RKID----R 379
AtPAP5|Q9C927|/1-396 222 -------------------------KY-----TPQNSWLQDEF--KKVN----R 239
AtPAP20|Q9LXI7|/1-427 259 T------------------------DFEPG--SEQYQWLENNL--KKID----R 280
AtPAP22|Q8S340|/1-434 260 T------------------------DFDCE--SDQYQWLQADL--AKVD----R 281
IbPAP3|Q9ZP18|/1-427 245 S------------------------AYGTY--TPQWKWLQGEL--PKVN----R 266
AtPAP21|Q9LXI4|/1-437 264 T------------------------PYESH--SDQYHWLQADL--RKVD----R 285
LpPAP|Q9MB07|/1-455 278 S------------------------AFGKY--TPQSEWLEQEF--PKVN----R 299
RcPAP2|B9SXP8|/1-463 258 S------------------------PFVRY--TPQWLWLQQEL--KHVN----R 279
IbPAP2|Q9SDZ9|/1-465 283 S------------------------AYGKY--TPQYKWLEEEL--PKVN----R 304
AtPAP11|Q9SI18|/1-441 263 S------------------------AYDKY--TPQNSWLQDEL--KKVN----R 284
GmPAP1|Q09131|/1-464 281 S------------------------AYGKY--TPQYKWLEKEL--PKVN----R 302
AtPAP25|O23244|/1-466 282 S------------------------AYGKY--TPQYVWLEQEL--KKVN----R 303
AtPAP12|Q38924|/1-469 287 S------------------------SYGIY--TPQYKWLEKEL--QGVN----R 308
NtPAP|Q84KZ3|/1-461 283 S------------------------ATSKY--TPQYRWLEAEL--KKVN----R 304
MtPAP1|Q4KU02|/1-465 282 S------------------------AYGKY--TPQYKWLEQEL--PKVN----R 303
OsPAP2|Q8S505|/1-476 279 S------------------------PFVKY--TPQWTWLKYEL--KHVD----R 300
LaAP1|Q93VM7|/1-460 279 S------------------------AFGYS--TLQYKWLTAEL--PKVN----R 300
PvPAP2|Q764C1|/1-457 286 S------------------------PFVKY--TPQYMWLQEEL--KRVD----R 307
LlAP2|Q8L6L1|/1-463 281 S------------------------AYGTS--SLQYKWLTSEL--PKVD----R 302
AtPAP10|Q9SIV9|/1-468 286 S------------------------AYGKY--TPQYQWLEEEF--PKVN----R 307
PvPAP1|P80366|/1-459 281 S------------------------AYGRG--TPQYTWLKKEL--RKVK----R 302
TaACP|C4PKL1|/1-477 280 S------------------------PFVKY--TPQWMWLKGEL--KRVD----R 301
AtPAP6|Q9C510|/1-466 282 S------------------------AYGKY--TPQYIWLEQEL--KNVN----R 303
AcPAP|Q93WP4|/1-481 285 S------------------------PFVKY--TPQWLWLSEEL--TRVD----R 306
AoPAP32|Q9XF09|/1-470 287 S------------------------AYGKY--TPQFKWLEDEL--PKVN----R 308
StPAP3|Q6J5M8|/1-477 279 S------------------------PFVKY--TPQWHWLKQEF--KKVN----R 300
IbPAP1|Q9SE00|/1-473 291 S------------------------GFVKY--SPQYKWFTSEL--EKVN----R 312
AtPAP26|Q949Y3|/1-475 280 S------------------------PFVKY--TPQWHWLSEEL--TRVD----R 301
RcPAP3|B9SXP6|/1-488 286 S------------------------PFVKY--TPQWEWLHQEL--KNVN----R 307
LlAP1|Q8L5E1|/1-477 282 S------------------------PFVKY--TPQYTWLKEEL--TRVD----R 303
GmPAP3|Q6YGT9|/1-512 318 S------------------------PFVKY--TPQYMWLKEEL--KRVE----R 339
LaAP2|Q9XJ24|/1-638 281 K------------------------AYGKY--TPQYQWLEAELPKPKVN----R 304
LlPPD4|Q8VXF4|/1-629 442 H------------------------DWREG--SEQYKFIEHCL--ATVD----R 463
LlPPD1|Q8VX11|/1-615 428 L------------------------DWRKG--TEQYEFIEKCL--ASVD----R 449
LlPPD2|Q8VXF6|/1-612 425 H------------------------DWREG--TEQYKFIEHCL--ASVD----R 446
TnPAP1|Q4RLR4|/1-378 183 FYL----------------------VFGLELLFKQYEWLRKDL--EEANRPENR 212
HsPAP7|Q6ZNF0|/1-438 248 FFL----------------------HYGRHLVQRQFRWLESDL--QKAN--KNR 275
CePAP3|Q9NAM9|/1-418 241 A------------------------EEMSNEAKAQYQWLREDL--A-------Q 261
MmPAP7|Q8BX37|/1-438 248 FFL----------------------HYGRHLIEKQFRWLENDL--QKAN--KNR 275
DmPAP1|Q9VZ56|/1-458 265 YFL----------------------NYGLKPLVFQFEWLREDL--AKANLPENR 294
DmPAP2|Q9VZ58|/1-450 255 YFL----------------------SYGFKLLTKQFEWLERDL--AEANLPENR 284
AmPAP|A0A087ZWE4|/1-438 240 YFM----------------------NYGIKQLVKQYEWLKKDL--MEANMPKNR 269
CePAP1|O01320|/1-419 209 SLCGNTKDIQNAGFIEMLRNESHDPRGPVNITAAEEQWAWLENNL-----E----A 255
DmPAP3|Q9VZ57|/1-453 254 YFT----------------------KFGIKQIVMQYDWLERDL--IEANKPENR 283
AgPAP|Q7PUN5|/1-463 246 YFM----------------------NYGLKPLVKQYEWLRRDL--EEANRPENR 275

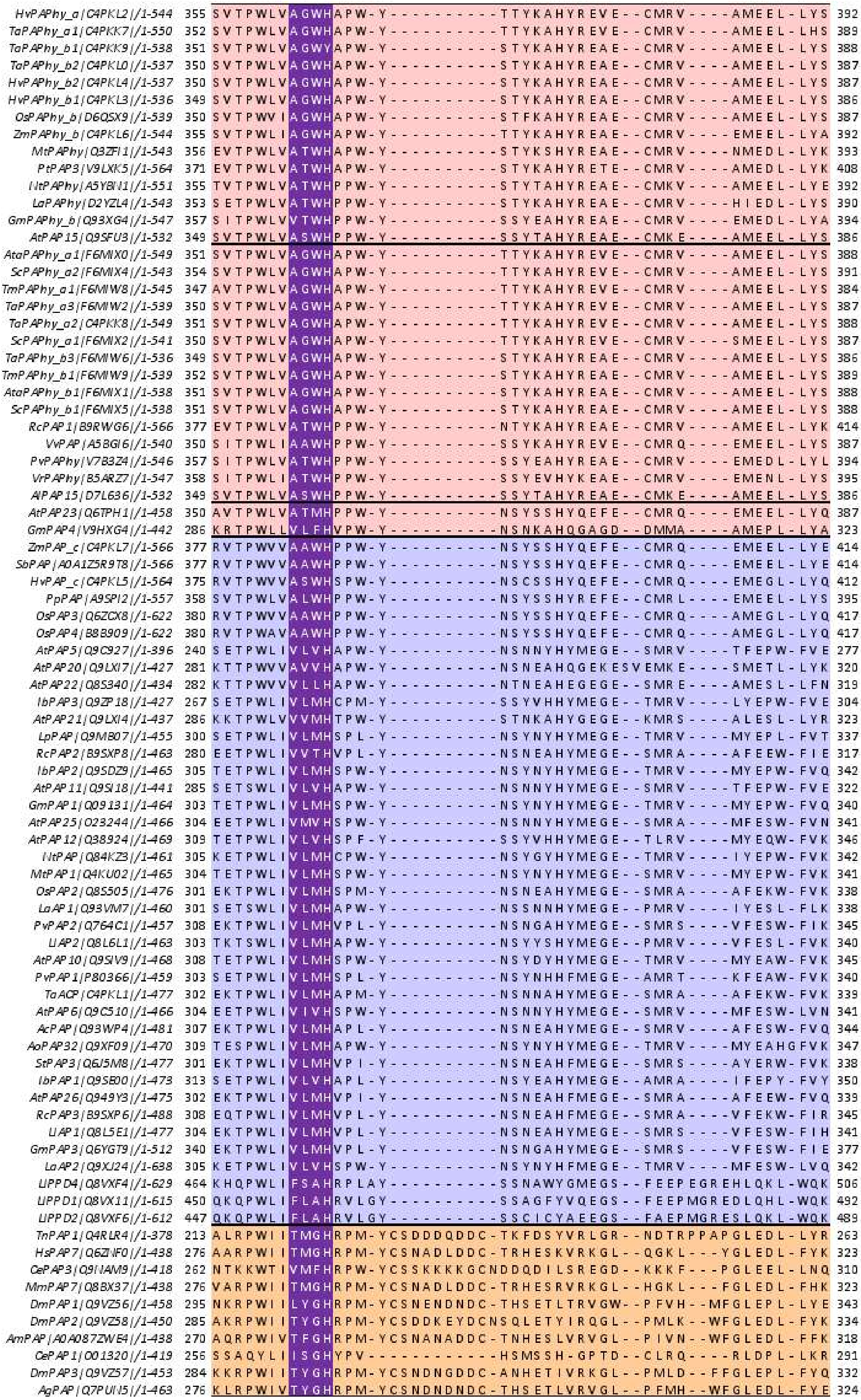

HvPAPhy_a/C4PKL2/1-544	393	HGLDIAFTGHVHAYER------SNRVFNYTL-----------DPCGAVYISVGDGG	431
TaPAPhy_a1/C4PKK7/1-550	390	HGLDIAFTGHVHAYER------SNRVFNYTL-----------DPCGAVHISVGDGG	428
TaPAPhy_b1/C4PKK9/1-538	389	YGLDIVFTGHVHAYER------SNRVFNYTL-----------DPCGAVHISVGDGG	427
TaPAPhy_b2/C4PKL0/1-537	388	YGLDIVFTGHVHAYER------SNRVFNYTL-----------DPCGAVHISVGDGG	426
HvPAPhy_b2/C4PKL4/1-537	388	YGIDIVFTGHVHAYER------SNRVFNYTL-----------DPCGAVHISVGDGG	426
HvPAPhy_b1/C4PKL3/1-536	387	YGIDIVFTGHVHAYER------SNRVFNYTL-----------DPCGAVHISVGDGG	425
OsPAPhy_b/D6QSX9/1-539	388	YAVDVVFTGHVHAYER------SNRVFNYTL-----------DPCGPVHISVGDGG	426
ZmPAPhy_b/C4PKL6/1-544	393	YGVDVVFTGHVHAYER------SNRVFNYTL-----------DACGPVHISVGDGG	431
MtPAPhy/Q3ZFI1/1-543	394	YGVDIVFNGHVHAYER------SNRVYNYTL-----------DPCGPVYITVGDGG	432
PtPAP3/V9LXK5/1-564	409	YGVDVVFSGHVHAYER------SNRVYNYTL-----------DPCGPVHITVGDGG	447
NtPAPhy/A5YBN1/1-551	393	CGVDLVFNGHVHAYER------SNRVYNYTL-----------DPCGPVYITVGDGG	431
LaPAPhy/D2YZL4/1-543	391	YGVDIVLNGHIHAYER------SNRVYNYNL-----------DPCGPVHITIGDGG	429
GmPAPhy_b/Q93XG4/1-547	395	YGVDIIFNGHVHAYER------SNRVYNYNL-----------DPCGPVYITVGDGG	433
AtPAP15/Q9SFU3/1-532	387	YGTDIVFNGHVHAYER------SNRVYNYEL-----------DPCGPVYIVIGDGG	425
AtaPAPhy_a1/F6MIX0/1-549	389	HGLDIAFTGHVHAYER------SNRVFNYTL-----------DPCGAVHISVGDGG	427
ScPAPhy_a2/F6MIX4/1-543	392	HGLDIAFTGHVHAYER------SNRVFNYTL-----------DPCGAVHISVGDGG	430
TmPAPhy_a1/F6MIW8/1-545	385	HGLDIAFTGHVHAYER------SNRVFNYTL-----------DPCGAVHISVGDGG	423
TaPAPhy_a3/F6MIW2/1-539	388	HGLDIAFTGHVHAYER------SNRVFNYTL-----------DPCGAVHISVGDGG	426
TaPAPhy_a2/C4PKK8/1-549	389	HGLDIAFTGHVHAYER------SNRVFNYTL-----------DPCGAVHISVGDGG	427
ScPAPhy_a1/F6MIX2/1-541	388	HGLDIAFTGHVHAYER------SNRVFNYTL-----------DPCGAVHISVGDGG	426
TaPAPhy_b3/F6MIW6/1-536	387	YGLDIVFTGHVHAYER------SNRVFNYTL-----------DPCGAVHISVGDGG	425
TmPAPhy_b1/F6MIW9/1-539	390	YGLDIVFTGHVHAYER------SNRVFNYTL-----------DPCGAVHISVGDGG	428
AtaPAPhy_b1/F6MIX1/1-538	389	YGLDIVFTGHVHAYER------SNRVFNYTL-----------DPCGAVHISVGDGG	427
ScPAPhy_b1/F6MIX5/1-538	389	YGLDIVFTGHVHAYER------SYRVFNYTL-----------DPCGAVHISVGDGG	427
RcPAP1/B9RWG6/1-566	415	YGVDMVFNGHVHAYER------SNRVYNYTL-----------DPCGPVHITVGDGG	453
VvPAP/A5BGI6/1-540	388	YGVDIVFNGHVHAYER------SNRVYNYTL-----------DPCGPVHIMVGDGG	426
PvPAPhy/V7B3Z4/1-546	395	YGVDIVFNGHVHAYER------SNRVYNYSL-----------DPCGPVHIAVGDGG	433
VrPAPhy/B5ARZ7/1-547	396	YGVDIVFNGHVHAYER------SNRVYNYSL-----------DPCGPVHIAVGDGG	434
AlPAP15/D7L636/1-532	387	YGIDIVFNGHVHAYER------SNRVYNYEL-----------DPCGPVYIVVGDGG	425
AtPAP23/Q6TPH1/1-458	388	YRVDIVFAGHVHAYER------MNRIYNYTL-----------DPCGPVYITIGDGG	426
GmPAP4/V9HXG4/1-442	324	ASVDLVIAGHVHAYER------SKRLYNGRL-----------DPCGAVHITIGDGG	362
ZmPAP_c/C4PKL7/1-566	415	YQVDIVFSGHVHAYER------MNRVFNYTL-----------DPCGPIYIGIGDGG	453
SbPAP/A0A1Z5R9T8/1-566	415	YQVDIVFTGHVHAYER------MNRVFNYTL-----------DPCGPVYIGIGDGG	453
HvPAP_c/C4PKL5/1-564	413	HGVDIVFSGHVHAYER------MNRVFNYTL-----------DSCGPVYITIGDGG	451
PpPAP/A9SPI2/1-557	396	YKVNIVFSGHVHAYER------TNQVYNYTL-----------NPCGPVYVTVGDGG	434
OsPAP3/Q6ZCX8/1-622	418	HGVDIVFSGHVHAYER------MNRVFNYTL-----------DPCGPVYITIGDGG	456
OsPAP4/B8B909/1-622	418	HGVDIVFSGHVHAYER------MNRVFNYTL-----------DPCGPVYITIGDGG	456
AtPAP5/Q9C927/1-396	278	NKVDIVFAGHVHAYER------SERVSNIQYNITDGMSTPVKDQNAPVYITIGDGG	327
AtPAP20/Q9LXI7/1-427	321	ARVDLVFAGHVHAYER------FSRVYQDKF-----------DKCGPVYINIGDGG	359
AtPAP22/Q8S340/1-434	320	ARVDVVFSGHVHAYER------FKRVYNNKA-----------DPCGPIHITIGDGG	358
IbPAP3/Q9ZP18/1-427	305	YKVDVVFAGHVHSYER------TERVSNVAYNIVNGLCSPKNDSSAPVYITIGDGG	354
AtPAP21/Q9LXI4/1-437	324	AQVDVVFAGHVHTYER------FKPIYNKKA-----------DPCGPMYITIGDGG	362
LpPAP/Q9MB07/1-455	338	YKVDVIFAGHVHAYER------SYRISNVAYNITDGKCTPTSDLSAPVYITVGDGG	387
RcPAP2/B9SXP8/1-463	318	YKVDVIFSGHVHAYER------SYRFSNVRSSVSSPNCYPVANESAPMYITVGDGG	367
IbPAP2/Q9SDZ9/1-465	343	HKVDLVFAGHVHAYER------SERVSNVAYDIVNGKCTPVRDQSAPVYITIGDGG	392
AtPAP11/Q9SI18/1-441	323	NKVDIVFAGHVHAYER------SKRISNIHYNITDGMSTPVKDQNAPIYITIGDGG	372
GmPAP1/Q09131/1-464	341	YKVDVVFAGHVHAYER------SERVSNVAYNIVNGLCAPVNDKSAPVYITIGDGG	390
AtPAP25/O23244/1-466	342	SKVDLVLSGHVHSYER------SERVSNIKYNITNGLSYPVKDPSAPIYITIGDGG	391
AtPAP12/Q38924/1-469	347	YKVDVVFAGHVHAYER------SERVSNIAYNIVNGLCEPISDESAPIYITIGDGG	396
NtPAP/Q84KZ3/1-461	343	YKVDMVFAGHVHAYER------SKRISNIDYKIVSGECTPASNPSAPVYITVGDGG	392
MtPAP1/Q4KU02/1-465	342	YKVDVVYAGHVHAYER------SERVSNVAYNVVNGICTPIKDQSAPVYITIGDGG	391
OsPAP2/Q8S505/1-476	339	YKVDLVFAGHVHAYER------SYRISNINYNITSGNRYPVPDKSAPVYITVGDGG	388
LaAP1/Q93VM7/1-460	339	YKVDVVFAGHVHAYER------SERVSNNKYNITNGICTPVEDITAPIYITNGDGG	388
PvPAP2/Q764C1/1-457	346	YKVDVIFAGHVHAYER------SYRFSNIDYNITNGNRYPLPDKSAPVYITVGDGG	395
LlAP2/Q8L6L1/1-463	341	YKGDVVFAGHVHAYER------PERVSNDKYNITNGICTPVKDISAPVYITNGDGG	390
AtPAP10/Q9SIV9/1-468	346	YKVDVVFAGHVHAYER------SERVSNIAYNVVNGICTPVKDQSAPVYITIGDGG	395
PvPAP1/P80366/1-459	341	YKVDVVFAGHVHAYER------SERVSNIAYKITNGLCTPVKDQSAPVYITIGDAG	390
TaACP/C4PKL1/1-477	340	YKVDLVFAGHVHAYER------SYRISNINYNVTSGNRYPVPDKSAPVYITVGDGG	389
AtPAP6/Q9C510/1-466	342	SKVDLVLSGHVHAYER------SERISNIKYNITNGLSSPVKDPNAPIYITIGDGG	391
AcPAP/Q93WP4/1-481	345	YKVDLVFAGHVHAYER------SYRISNIVYNITSGNRYPIPDKSAPVYITVGDGG	394
AoPAP32/Q9XF09/1-470	348	YKVDLVFAGHVHAYER------TERISNIVYNVVNGICTPVNDSSAPIYITIGDGG	397
StPAP3/Q6J5M8/1-477	339	YKVDVIFAGHVHAYER------SYRISNIHYNVSGGDAYPVPDKAAPIYITVGDGG	388
IbPAP1/Q9SE00/1-473	351	YKVDIVFSGHVHSYER------SERVSNVAYNIVNAKCTPVSDESAPVYITIGDGG	400
AtPAP26/Q949Y3/1-475	340	HKVDVIFAGHVHAYER------SYRISNVRYNVSSGDRYPVPDKSAPVYITVGDGG	389
RcPAP3/B9SXP6/1-488	346	YKVDIIFAGHVHAYER------SYRISNIQYNVSSGERYPIADKSAPVYITVGDGG	395
LlAP1/Q8L5E1/1-477	342	YEVDVIFAGHVHAYER------SYRFSNTDYNITSGHRFPIADKSAPVYITVGDGG	391
GmPAP3/Q6YGT9/1-512	378	YKVDVIFAGHVHAYER------SYRYSNVDYNITGGNRYPLPNKSAPVYITVGDGG	427
LaAP2/Q9XJ24/1-638	343	YKVDVVFAGHVHAYER------SECVSNVEVRHCKWQVYPCKDQSAPVYITIGDGG	392
LlPPD4/Q8VXF4/1-629	507	YKVDIAFYGHVHNYER------ICPIYQNQCVNSEKTHYSG-TVNGTIHVVVGGGG	555
LlPPD1/Q8VX11/1-615	493	YKVDIAMYGHVHNYER------TCPIYQNVCTNKEKHNYKG-NLNGTIHVVVGGGG	541
LlPPD2/Q8VXF6/1-612	490	YKVDIAIYGHVHNYER------TCPIYQNICTSEEKHHYKG-TLNGTIHIVAGGAG	538
TnPAP1/Q4RLR4/1-378	264	YGVDLELWAHEHTYER------LWPVYGDKVWNGS-TEQPYVKPRAPVHIITGSAG	312
HsPAP7/Q6ZNF0/1-438	324	YGVDLQLWAHEHSYER------LWPIYNYQVFNGS-REMPYTNPRGPVHIITGSAG	372
CePAP3/Q9NAM9/1-418	311	YKVDMVLYGHKHTYER------MWPIYNKNPFKSA-NPGHIKNAPAPVYILTGGAG	359
MmPAP7/Q8BX37/1-438	324	YGVDLEFWAHEHSYER------LWPIYNYQVFNGS-LESPYTNPRGPVHIITGSAG	372
DmPAP1/Q9VZ56/1-458	344	FGVDVAIWAHEHSYER------LWPIYDYKVRNGTLKDSPYNDPSAPVHIVTGSAG	393
DmPAP2/Q9VZ58/1-450	335	HGVDVEIFAHEHFYTR------LWPIYDYKVYNGS-AEAPYTNPKAPIQIITGSAG	383
AmPAP/A0A087ZWE4/1-438	319	YKVDLLLWAHEHSYER------LWPMYNFKVQNGS-YEKPYKNYKAPVHIVTGSAG	367
CePAP1/O01320/1-419	292	FNVNAYFSGHDHSLQHFTFPGYGEHIINYVVSGAASRA----DASTK-HI---KEF	339
DmPAP3/Q9VZ57/1-453	333	YGVDIELWAHEHCYER------MWPMYNYTVFNGS-LAEPYVNPGAPIHIISGAAG	381
AgPAP/Q7PUN5/1-463	325	HGVDVEIWAHEHSYER------LFPIYDYKVYNGS-YEEPYRNPRAPVHLVTGSAG	373

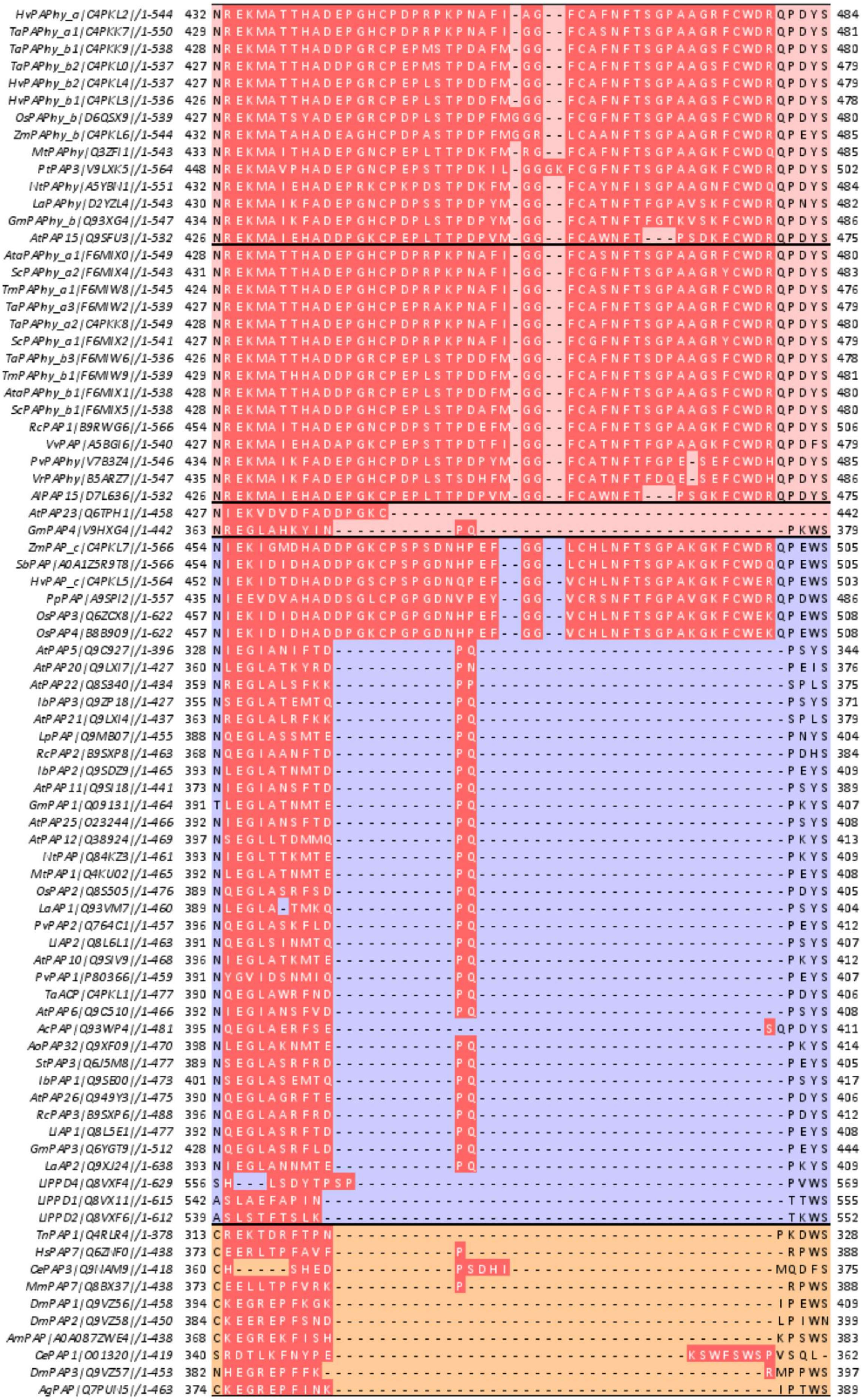
HvPAPhy_a|C4PKL2|/1-544 432 NREKMATTHADEPGHCPDPRPKPNAFI-AG--FCAFNFTSGPAAGRFCWDRQPDYS 484
TaPAPhy_a1|C4PKK7|/1-550 429 NREKMATTHADEPGHCPDPRPKPNAFI-GG--FCASNFTSGPAAGRFCWDRQPDYS 481
TaPAPhy_b1|C4PKK9|/1-538 428 NREKMATTHADDPGRCPEPMSTPDAFM-GG--FCAFNFTSGPAAGSFCWDRQPDYS 480
TaPAPhy_b2|C4PKL0|/1-537 427 NREKMATTHADDPGRCPEPMSTPDAFM-GG--FCAFNFTSGPAAGSFCWDRQPDYS 479
HvPAPhy_b2|C4PKL4|/1-537 427 NREKMATTHADEPGRCPEPLSTPDDFM-GG--FCAFNFTSGPAAGSFCWDRQPDYS 479
HvPAPhy_b1|C4PKL3|/1-536 426 NREKMATTHADEPGRCPEPLSTPDDFM-GG--FCAFNFTSGPAAGSFCWDRQPDYS 478
OsPAPhy_b|D6QSX9|/1-539 427 NREKMATSYADEPGRCPDPLSTPDPFMGGG--FCGFNFTSGPAAGSFCWDRQPDYS 480
ZmPAPhy_b|C4PKL6|/1-544 432 NREKMATAHADEAGHCPDPASTPDPFMGGR--LCAANFTSGPAAGRFCWDRQPEYS 485
MtPAPhy|Q3ZFI1|/1-543 433 NREKMAITHADEPGNCPEPLTTPDKFM-RG--FCAFNFTSGPAAGKFCWDQQPDYS 485
PtPAP3|V9LXK5|/1-564 448 NREKMAVPHADEPGNCPEPSTTPDKIL-GGGKFCGFNFTSGPAAGKFCWDRQPDYS 502
NtPAPhy|A5YBN1|/1-551 432 NREKMAIEHADEPRKCPKPDSTPDKFM-GG--FCAYNFISGPAAGNFCWDQQPDYS 484
LaPAPhy|D2YZL4|/1-543 430 NREKMAIKFADEPGNCPDPSSTPDPYM-GG--FCATNFTFGPAVSKFCWDRQPNYS 482
GmPAPhy_b|Q93XG4|/1-547 434 NREKMAIKFADEPGHCPDPLSTPDPYM-GG--FCATNFTFGTKVSKFCWDRQPDYS 486
AtPAP15|Q9SFU3|/1-532 426 NREKMAIEHADDPGKCPEPLTTPDPVM-GG--FCAWNFT---PSDKFCWDRQPDYS 475
AtaPAPhy_a1|F6MIX0|/1-549 428 NREKMATTHADEPGHCPDPRPKPNAFI-GG--FCASNFTSGPAAGRFCWDRQPDYS 480
ScPAPhy_a2|F6MIX4|/1-543 431 NREKMATTHADEPGHCPDPRPKPNAFI-GG--FCGFNFTSGPAAGRYCWDRQPDYS 483
TmPAPhy_a1|F6MIW8|/1-545 424 NREKMATTHADEPGHCPDPRPKPNAFI-GG--FCASNFTSGPAAGRFCWDRQPDYS 476
TaPAPhy_a3|F6MIW2|/1-539 427 NREKMATTHADEPGHCPEPRAKPNAFI-GG--FCAFNFTSGPAAGRFCWDRQPDYS 479
TaPAPhy_a2|C4PKK8|/1-549 428 NREKMATTHADEPGHCPDPRPKPNAFI-GG--FCAFNFTSGPAAGRFCWDRQPDYS 480
ScPAPhy_a1|F6MIX2|/1-541 427 NREKMATTHADEPGHCPDPRPKPNAFI-GG--FCGFNFTSGPAAGRYCWDRQPDYS 479
TaPAPhy_b3|F6MIW6|/1-536 426 NREKMATTHADDPGRCPEPLSTPDDFM-GG--FCAFNFTSDPAAGSFCWDRQPDYS 478
TmPAPhy_b1|F6MIW9|/1-539 429 NREKMATHHADDPGRCPEPLSTPDDFM-GG--FCAFNFTSGPAAGSFCWDRQPDYS 481
AtaPAPhy_b1|F6MIX1|/1-538 428 NREKMATTHADDPGRCPEPLSTPDDFM-GG--FCAFNFTSGPAAGSFCWDRQPDYS 480
ScPAPhy_b1|F6MIX5|/1-538 428 NREKMATTHADDPGHCPEPLSTPDAFM-GG--FCAFNFTSGPAAGSFCWDRQPDYS 480
RcPAP1|B9RWG6|/1-566 454 NREKMAITHADEPGNCPDPSTTPDEFM-GG--FCAFNFTSGPAAGKFCWDRQPDYS 506
VvPAP|A5BGI6|/1-540 427 NREKMAIEHADAPGKCPEPSTTPDTFI-GG--FCATNFTFGPAAGKFCWDRQPDFS 479
PvPAPhy|V7B3Z4|/1-546 434 NREKMAIKFADEPGHCPDPLSTPDPYM-GG--FCATNFTFGPE-SEFCWDHQPDYS 485
VrPAPhy|B5ARZ7|/1-547 435 NREKMAIKFADEPGHCPDPLSTSDHFM-GG--FCATNFTFDQE-SEFCWDHQPDYS 486
AlPAP15|D7L636|/1-532 426 NREKMAIEHADDPGKCPEPLTTPDPVM-GG--FCAWNFT---PSGKFCWDRQPDYS 475
AtPAP23|Q6TPH1|/1-458 427 NIEKVDVDFADDPGKC-- 442
GmPAP4|V9HXG4|/1-442 363 NREGLAHKYIN----------PQ-----------------------------PKWS 379
ZmPAP_c|C4PKL7|/1-566 454 NIEKIGMDHADDPGKCPSPSDNHPEF--GG--LCHLNFTSGPAKGKFCWDRQPEWS 505
SbPAP|A0A1Z5R9T8|/1-566 454 NIEKIDIDHADDPGKCPSPGDNHPEF--GG--LCHLNFTSGPAKGKFCWDQQPEWS 505
HvPAP_c|C4PKL5|/1-564 452 NIEKIDTDHADDPGSCPSPGDNQPEF--GG--VCHLNFTSGPAKGKFCWERQPEWS 503
PpPAP|A9SPI2|/1-557 435 NIEEVDVAHADDSGLCPGPGDNVPEY--GG--VCRSNFTFGPAVGKFCWDRQPDWS 486
OsPAP3|Q6ZCX8|/1-622 457 NIEKIDIDHADDPGKCPGPGDNHPEF--GG--VCHLNFTSGPAKGKFCWEKQPEWS 508
OsPAP4|B8B909|/1-622 457 NIEKIDIDHADDPGKCPGPGDNHPEF--GG--VCHLNFTSGPAKGKFCWEKQPEWS 508
AtPAP5|Q9C927|/1-396 328 NIEGIANIFTD----------PQ-----------------------------PSYS 344
AtPAP20|Q9LXI7|/1-427 360 NLEGLATKYRD----------PN-----------------------------PEIS 376
AtPAP22|Q8S340|/1-434 359 NREGLALSFKK----------PP-----------------------------SPLS 375
IbPAP3|Q9ZP18|/1-427 355 NSEGLATEMTQ----------PQ-----------------------------PSYS 371
AtPAP21|Q9LXI4|/1-437 363 NREGLALRFKK----------PQ-----------------------------SPLS 379
LpPAP|Q9MB07|/1-455 388 NQEGLASSMTE----------PQ-----------------------------PNYS 404
RcPAP2|B9SXP8|/1-463 368 NQEGIAANFTD----------PQ-----------------------------PDHS 384
IbPAP2|Q9SDZ9|/1-465 393 NLEGLATNMTD----------PQ-----------------------------PEYS 409
AtPAP11|Q9SI18|/1-441 373 NIEGIANSFTD----------PQ-----------------------------PSYS 389
GmPAP1|Q09131|/1-464 391 TLEGLATNMTE----------PQ-----------------------------PKYS 407
AtPAP25|O23244|/1-466 392 NIEGIANSFTD----------PQ-----------------------------PSYS 408
AtPAP12|Q38924|/1-469 397 NSEGLLTDMMQ----------PQ-----------------------------PKYS 413
NtPAP|Q84KZ3|/1-461 393 NIEGLTTKMTE----------PQ-----------------------------PKYS 409
MtPAP1|Q4KU02|/1-465 392 NLEGLATNMTE----------PQ-----------------------------PEYS 408
OsPAP2|Q8S505|/1-476 389 NQEGLASRFSD----------PQ-----------------------------PDYS 405
LaAP1|Q93VM7|/1-460 389 NLEGLA-TMKQ----------PQ-----------------------------PSYS 404
PvPAP2|Q764C1|/1-457 396 NQEGLASKFLD----------PQ-----------------------------PEYS 412
LlAP2|Q8L6L1|/1-463 391 NQEGLSINMTQ----------PQ-----------------------------PSYS 407
AtPAP10|Q9SIV9|/1-468 396 NIEGLATKMTE----------PQ-----------------------------PKYS 412
PvPAP1|P80366|/1-459 391 NYGVIDSNMIQ----------PQ-----------------------------PEYS 407
TaACP|C4PKL1|/1-477 390 NQEGLAWRFND----------PQ-----------------------------PDYS 406
AtPAP6|Q9C510|/1-466 392 NIEGIANSFVD----------PQ-----------------------------PSYS 408
AcPAP|Q93WP4|/1-481 395 NQEGLAERFSE---------------------------------------SQPDYS 411
AoPAP32|Q9XF09|/1-470 398 NLEGLAKNMTE----------PQ-----------------------------PKYS 414
StPAP3|Q6J5M8|/1-477 389 NSEGLASRFRD----------PQ-----------------------------PEYS 405
IbPAP1|Q9SE00|/1-473 401 NSEGLASEMTQ----------PQ-----------------------------PSYS 417
AtPAP26|Q949Y3|/1-475 390 NQEGLAGRFTE----------PQ-----------------------------PDYS 406
RcPAP3|B9SXP6|/1-488 396 NQEGLAARFRD----------PQ-----------------------------PDYS 412
LlAP1|Q8L5E1|/1-477 392 NQEGLASRFTD----------PQ-----------------------------PEYS 408
GmPAP3|Q6YGT9|/1-512 428 NQEGLASRFLD----------PQ-----------------------------PEYS 444
LaAP2|Q9XJ24|/1-638 393 NIEGLANNMTE----------PQ-----------------------------PKYS 409
LlPPD4|Q8VXF4|/1-629 556 SH---LSDYTPSP---------------------------------------PVWS 569
LlPPD1|Q8VX11|/1-615 542 ASLAEFAPIN--TTWS 555
LlPPD2|Q8VXF6|/1-612 539 ASLSTFTSLK--TKWS 552
TnPAP1|Q4RLR4|/1-378 313 CREKTDRFTPN--PKDWS 328
HsPAP7|Q6ZNF0|/1-438 373 CEERLTPFAVF----------P------------------------------RPWS 388
CePAP3|Q9NAM9|/1-418 360 CH-----SHED----------PSDHI-------------------------MQDFS 375
MmPAP7|Q8BX37|/1-438 373 CEELLTPFVRK----------P------------------------------RPWS 388
DmPAP1|Q9VZ56|/1-458 394 CKEGREPFKGK--IPEWS 409
DmPAP2|Q9VZ58|/1-450 384 CKEEREPFSND--LPIWN 399
AmPAP|A0A087ZWE4|/1-438 368 CKEGREKFISH--KPSWS 383
CePAP1|O01320|/1-419 340 SRDTLKFNYPE--------------------------------KSWFSWSPVSQL- 362
DmPAP3|Q9VZ57|/1-453 382 NHEGREPFFK--RMPPWS 397
AgPAP|Q7PUN5|/1-463 374 CKEGREPFINK--IPTWS 389

HvPAPhy_a|C4PKL2|/1-544	485	AYRESSFGHGILEVKNETHAL-----WRWHRNQDL---------------------	514
TaPAPhy_a1|C4PKK7|/1-550	482	AYRESSFGHGILEVKNETHAL-----WRWHRNQDH---------------------	511
TaPAPhy_b1|C4PKK9|/1-538	481	AYRESSFGHGILEVKNETYAL-----WKWHRNQDL---------------------	510
TaPAPhy_b2|C4PKL0|/1-537	480	AYRESSFGHGILEVKNETHAL-----WKWHRNQDL---------------------	509
HvPAPhy_b2|C4PKL4|/1-537	480	AYRESSFGHGILEVKNETHAL-----WKWHRNQDL---------------------	509
HvPAPhy_b1|C4PKL3|/1-536	479	AYRESSFGHGILEVKNETHAL-----WKWHRNQDL---------------------	508
OsPAPhy_b|D6QSX9|/1-539	481	AYRESSFGHGILEVKNETHAL-----WRWHRNQDL---------------------	510
ZmPAPhy_b|C4PKL6|/1-544	486	AYRESSFGHGVLEVRNDTHAL-----WRWHRNQDL---------------------	515
MtPAPhy|Q3ZFI1|/1-543	486	AFRESSFGHGILEVKNETHAL-----WSWNRNQDY---------------------	515
PtPAP3|V9LXK5|/1-564	503	AFRESSFGHGILEVKNETHAL-----WTWHRNQDF---------------------	532
NtPAPhy|A5YBN1|/1-551	485	AYRESSFGHGILEVKSETHAL-----WTWHRNQDM---------------------	514
LaPAPhy|D2YZL4|/1-543	483	AFRESSFGYGILEVKNETWAL-----WSWYRNQDS---------------------	512
GmPAPhy_b|Q93XG4|/1-547	487	AFRESSFGYGILEVKNETWAL-----WSWYRNQDS---------------------	516
AtPAP15|Q9SFU3|/1-532	476	ALRESSFGHGILEMKNETWAL-----WTWYRNQDS---------------------	505
AtaPAPhy_a1|F6MIX0|/1-549	481	AYRESSFGHGILEVKNETHAL-----WRWHRNQDH---------------------	510
ScPAPhy_a2|F6MIX4|/1-543	484	AYRESSFGHGILEVKNETHAL-----WRWHRNQDM---------------------	513
TmPAPhy_a1|F6MIW8|/1-545	477	AYRESSFGHGILEVKNETHAL-----WRWHRNQDH---------------------	506
TaPAPhy_a3|F6MIW2|/1-539	480	AYRESSFGHGILEVKNETHAL-----WRWHRNQDM---------------------	509
TaPAPhy_a2|C4PKK8|/1-549	481	AYRESSFGHGILEVKNETHAL-----WRWHRNQDM---------------------	510
ScPAPhy_a1|F6MIX2|/1-541	480	AYRESSFGHGILEVKNETHAL-----WRWHRNQDM---------------------	509
TaPAPhy_b3|F6MIW6|/1-536	479	AYRESSFGHGILEVKNETHAL-----WKWHRNQDL---------------------	508
TmPAPhy_b1|F6MIW9|/1-539	482	AYRESSFGHGILEVKNETHAL-----WKWHRNQDL---------------------	511
AtaPAPhy_b1|F6MIX1|/1-538	481	AYRESSFGHGILEVKNETHAL-----WKWHRNQDL---------------------	510
ScPAPhy_b1|F6MIX5|/1-538	481	AYRESSFGHGILEVKNETHAL-----WKWHRNQDL---------------------	510
RcPAP1|B9RWG6|/1-566	507	AYRESSFGHGILEVKNETHAL-----WTWHRNQDL---------------------	536
VvPAP|A5BGI6|/1-540	480	AFRESSFGHGILEVKNDTWAL-----WTWYRNQDS---------------------	509
PvPAPhy|V7B3Z4|/1-546	486	AFRETSFGYGILEVKNETWAL-----WSWYRNQDS---------------------	515
VrPAPhy|B5ARZ7|/1-547	487	AFRETSFGYGILEVKNETWAL-----WSWYRNQDS---------------------	516
AlPAP15|D7L636|/1-532	476	AMRESSFGHGILEMKNETWAL-----WTWYRNQDS---------------------	505
AtPAP23|Q6TPH1|/1-458	443	---------------HSSYDL-----FFF---------------------------	451
GmPAP4|V9HXG4|/1-442	380	EFREASFGHGELKIVNSTHAF-----WSWHRNDDD---------------------	409
ZmPAP_c|C4PKL7|/1-566	506	AYRESSFGHGILEVLNSTYAL-----WTWHRNQDA---------------------	535
SbPAP|A0A1Z5R9T8|/1-566	506	AYRESSFGHGILEVLNSTYAL-----WTWHRNQDA---------------------	535
HvPAP_c|C4PKL5|/1-564	504	AFRESSFGHGILEVVNSTYAL-----WTWHRNQDT---------------------	533
PpPAP|A9SPI2|/1-557	487	AFRESSFGHGVLEVVNSSHAL-----WTWHRNQDM---------------------	516
OsPAP3|Q6ZCX8|/1-622	509	AFRESSFGHGILEVVNSTYAL-----WTWHRNQDA---------------------	538
OsPAP4|B8B909|/1-622	509	AFRESSFGHGILEVVNSTYAL-----WTWHRNQDA---------------------	538
AtPAP5|Q9C927|/1-396	345	AFREASFGHALLEIKNRTHAH-----YTWHRNKED---------------------	374
AtPAP20|Q9LXI7|/1-427	377	LFREASFGHGQLVVENATHAR-----WEWHRNDDD---------------------	406
AtPAP22|Q8S340|/1-434	376	EFRESSFGHGRLKVMDGKRAH-----WSWHRNNDS---------------------	405
IbPAP3|Q9ZP18|/1-427	372	AYREASFGHGIFDIKNRTHAH-----FGWHRNQDG---------------------	401
AtPAP21|Q9LXI4|/1-437	380	EFRESSFGHGRLRIIDHKRAH-----WSWHRNNDE---------------------	409
LpPAP|Q9MB07|/1-455	405	AYREASFGHAIFGIKNRTHAY-----YNWYRNQDG---------------------	434
RcPAP2|B9SXP8|/1-463	385	AFREASYGHSTLEIMNKTHAF-----YYWHRNDDG---------------------	414
IbPAP2|Q9SDZ9|/1-465	410	AFREASFGHATLDIKNRTHAY-----YSWHRNQDG---------------------	439
AtPAP11|Q9SI18|/1-441	390	AFREASFGHALLEIKNRTHAH-----YTWHRNKED---------------------	419
GmPAP1|Q09131|/1-464	408	AFREASFGHAIFDITNRTHAH-----YSWHRNQDG---------------------	437
AtPAP25|O23244|/1-466	409	AYREASFGHAVLEIYNRTHAY-----YTWHRNQDN---------------------	438
AtPAP12|Q38924|/1-469	414	AFREASFGHGLLEIKNRTHAY-----FSWNRNQDG---------------------	443
NtPAP|Q84KZ3|/1-461	410	AYRESSFGHAILEIKNRTHAY-----YSWHRNQDG---------------------	439
MtPAP1|Q4KU02|/1-465	409	AYREASFGHAIFDIKNRTHAH-----YSWHRNQDG---------------------	438
OsPAP2|Q8S505|/1-476	406	AFREASYGHSILQLKNRTHAI-----YQWNRNDDG---------------------	435
LaAP1|Q93VM7|/1-460	405	AYRKASFGHGIFAIKNRTHAH-----YSWNRNQDG---------------------	434
PvPAP2|Q764C1|/1-457	413	AFREASYGHSTLEIKNRTHAI-----YHWNRNDDG---------------------	442
LlAP2|Q8L6L1|/1-463	408	AYREASFGHGTLEIKNRTHAH-----YSWNRNQDG---------------------	437
AtPAP10|Q9SIV9|/1-468	413	AFREASFGHAIFSIKNRTHAH-----YGWHRNHDG---------------------	442
PvPAP1|P80366|/1-459	408	AFREASFGHGMFDIKNRTHAH-----FSWNRNQDG---------------------	437
TaACP|C4PKL1|/1-477	407	AFREASFGHSTLQLVNRTHAV-----YQWNRNDDG---------------------	436
AtPAP6|Q9C510|/1-466	409	AYREASFGHAVLEIMNRTHAQ-----YTWHRNQDN---------------------	438
AcPAP|Q93WP4|/1-481	412	AFRESSYGHSTLELRNRTHAF-----YQWNRNDDG---------------------	441
AoPAP32|Q9XF09|/1-470	415	AFREASFGHATLDIKNRTHAY-----YAWHRNQDG---------------------	444
StPAP3|Q6J5M8|/1-477	406	AFREASYGHSTLDIKNRTHAI-----YHWNRNDDG---------------------	435
IbPAP1|Q9SE00|/1-473	418	AFREASFGHGIFDIKNRTHAH-----FSWHRNQDG---------------------	447
AtPAP26|Q949Y3|/1-475	407	AFREASYGHSTLDIKNRTHAI-----YHWNRNDDG---------------------	436
RcPAP3|B9SXP6|/1-488	413	AFREASFGHSTLEIKNRTHAF-----YQWNRNDDG---------------------	442
LlAP1|Q8L5E1|/1-477	409	AFREASYGHSTLEIKNRTHAI-----YHWNRNDDG---------------------	438
GmPAP3|Q6YGT9|/1-512	445	AFREASYGHSTLEIKNRTHAI-----YHWNRNDDG---------------------	474
LaAP2|Q9XJ24|/1-638	410	AYREASFGHAIFDIKNRTVLGLFSENYRLHTKQEEDEKNLASKGAMVKGVILQQVV	465
LlPPD4|Q8VXF4|/1-629	570	VFRDRDFGFGKLTAFNHSYLL-----FEYKRSSD----------------------	598
LlPPD1|Q8VX11|/1-615	556	IFKDHDFGFVKLTAFDHSNLL-----LEYRKSSD----------------------	584
LlPPD2|Q8VXF6|/1-612	553	IFKDYDHGFVKLTAFDHSNLL-----FEYKKSRD----------------------	581
TnPAP1|Q4RLR4|/1-378	329	AFRSRDYGYTRMQVVNATHLY-----LEQVSDDQY---------------------	358
HsPAP7|Q6ZNF0|/1-438	389	AVRVKEYGYTRLHILNGTHIH-----IQQVSDDQD---------------------	418
CePAP3|Q9NAM9|/1-418	376	VKALGEYGYTYLTVYNSTHIS-----TDYVDTSST---------------------	405
MmPAP7|Q8BX37|/1-438	389	AVRVKEYGYTRMHILNGTHMH-----IQQVSDDQD---------------------	418
DmPAP1|Q9VZ56|/1-458	410	AFHSQDYGYTRLKAHNRTHIH-----FEQV-SDDK---------------------	438
DmPAP2|Q9VZ58|/1-450	400	AYHSNDYGYTRLKAHNGTHLH-----FEQVSDDQN---------------------	429
AmPAP|A0A087ZWE4|/1-438	384	AYRSSDYGYTRMKVYNQTHLY-----LEQV-SDDK---------------------	412
CePAP1|O01320|/1-419	363	GFRKGGLIYAEFGHYNARLDF-----FDKR--------------------------	387
DmPAP3|Q9VZ57|/1-453	398	AFHSQDFGYLRLKAHNGTHLH-----FEQV-SDDK---------------------	426
AgPAP|Q7PUN5|/1-463	390	AIHSRDYGYTRMKAINGSHLY-----FEQI-SVDK---------------------	418

HvPAPhy_a|C4PKL2|/1-544	515	--------Y-GSA-GDE---------------------------IYI--VREPERC	531
TaPAPhy_a1|C4PKK7|/1-550	512	--------Y-GSA-GDE---------------------------IYI--VREPHRC	528
TaPAPhy_b1|C4PKK9|/1-538	511	--------YQGAV-GDE---------------------------IYI--VREPERC	528
TaPAPhy_b2|C4PKL0|/1-537	510	--------YQGAV-GDE---------------------------IYI--VREPERC	527
HvPAPhy_b2|C4PKL4|/1-537	510	--------YQGAV-GDE---------------------------IYI--VREPGRC	527
HvPAPhy_b1|C4PKL3|/1-536	509	--------YQGAV-GDE---------------------------IYI--VREPERC	526
OsPAPhy_b|D6QSX9|/1-539	511	--------Y-GSV-GDE---------------------------IYI--VREPDKC	527
ZmPAPhy_b|C4PKL6|/1-544	516	--------HAANVAADE---------------------------VYI--VREPDKC	534
MtPAPhy|Q3ZFI1|/1-543	516	--------Y-GTA-GDE---------------------------IYI--VRQPDKC	532
PtPAP3|V9LXK5|/1-564	533	--------Y-EAA-GDQ---------------------------IYI--VRQPDLC	549
NtPAPhy|A5YBN1|/1-551	515	--------Y-NKA-GDI---------------------------IYI--VRQPEKC	531
LaPAPhy|D2YZL4|/1-543	513	--------Y-NEV-GDQ---------------------------IYI--VRQPHLC	529
GmPAPhy_b|Q93XG4|/1-547	517	--------Y-KEV-GDQ---------------------------IYI--VRQPDIC	533
AtPAP15|Q9SFU3|/1-532	506	--------S-SEV-GDQ---------------------------IYI--VRQPDRC	522
AtaPAPhy_a1|F6MIX0|/1-549	511	--------Y-GSA-GDE---------------------------IYI--VREPHRC	527
ScPAPhy_a2|F6MIX4|/1-543	514	--------Y-GSA-GDE---------------------------IYI--VREPERC	530
TmPAPhy_a1|F6MIW8|/1-545	507	--------Y-GSA-GDE---------------------------IYI--VREPHRC	523
TaPAPhy_a3|F6MIW2|/1-539	510	--------Y-GSA-GDE---------------------------IYI--VREPHRC	526
TaPAPhy_a2|C4PKK8|/1-549	511	--------Y-GSA-GDE---------------------------IYI--VREPHRC	527
ScPAPhy_a1|F6MIX2|/1-541	510	--------Y-GSA-GDE---------------------------IYI--VREPERC	526
TaPAPhy_b3|F6MIW6|/1-536	509	--------YQGGV-GDE---------------------------IYI--VREPERC	526
TmPAPhy_b1|F6MIW9|/1-539	512	--------YQGVV-ADE---------------------------IYI--VREPERC	529
AtaPAPhy_b1|F6MIX1|/1-538	511	--------YQGAV-GDE---------------------------IYI--VREPERC	528
ScPAPhy_b1|F6MIX5|/1-538	511	--------YQGAV-GDE---------------------------IFI--VREPERC	528
RcPAP1|B9RWG6|/1-566	537	--------Y-SSA-GDQ---------------------------IYI--VRQQERC	553
VvPAP|A5BGI6|/1-540	510	--------R-DNA-GDQ---------------------------IYI--VRTPDMC	526
PvPAPhy|V7B3Z4|/1-546	516	--------Y-KEV-GDQ---------------------------IYI--VRQPDIC	532
VrPAPhy|B5ARZ7|/1-547	517	--------Y-KEV-GDQ---------------------------IYI--VRQPDIC	533
AlPAP15|D7L636|/1-532	506	--------S-SQV-GDQ---------------------------IYI--VRQPDRC	522
AtPAP23|Q6TPH1|/1-458		--	
GmPAP4|V9HXG4|/1-442	410	--------E-PVK-ADD---------------------------IWITSL-VSSRC	427
ZmPAP_c|C4PKL7|/1-566	536	--------YAENSVGDQ---------------------------IYI--VRQPDKC	554
SbPAP|A0A1Z5R9T8|/1-566	536	--------YGENSVGDQ---------------------------IYI--VRQPDKC	554
HvPAP_c|C4PKL5|/1-564	534	--------YGEHSVGDE---------------------------IYI--VREPDKC	552
PpPAP|A9SPI2|/1-557	517	--------YKEAV-GDQ---------------------------IYI--VRQPDGC	534
OsPAP3|Q6ZCX8|/1-622	539	--------YGEDSVGDQ---------------------------IYI--VRQPDKC	557
OsPAP4|B8B909|/1-622	539	--------YGEDSVGDQ---------------------------IYI--VRQPDKC	557
AtPAP5|Q9C927|/1-396	375	--------E-AVI-ADS---------------------------IWL---------	384
AtPAP20|Q9LXI7|/1-427	407	--------V-SVE-KDS---------------------------VWLTSLLADSSC	425
AtPAP22|Q8S340|/1-434	406	--------N-SLL-ADE---------------------------VWLDSLSTSSSC	424
IbPAP3|Q9ZP18|/1-427	402	--------L-AVE-GDS---------------------------LWF---------	411
AtPAP21|Q9LXI4|/1-437	410	--------M-SSI-ADE---------------------------VSFESPRTSSHC	428
LpPAP|Q9MB07|/1-455	435	--------N-AVE-ADS---------------------------LWF---------	444
RcPAP2|B9SXP8|/1-463	415	----------KKVVADK---------------------------LVL---------	424
IbPAP2|Q9SDZ9|/1-465	440	--------Y-AVE-ADS---------------------------MWV---------	449
AtPAP11|Q9SI18|/1-441	420	--------E-AVI-ADS---------------------------IWL---------	429
GmPAP1|Q09131|/1-464	438	--------V-AVE-ADS---------------------------LWS---------	447
AtPAP25|O23244|/1-466	439	--------E-PVA-ADS---------------------------IML---------	448
AtPAP12|Q38924|/1-469	444	--------N-AVA-ADS---------------------------VWL---------	453
NtPAP|Q84KZ3|/1-461	440	--------F-SAK-ADS---------------------------FLF---------	449
MtPAP1|Q4KU02|/1-465	439	--------Y-SVE-ADS---------------------------HWF---------	448
OsPAP2|Q8S505|/1-476	436	--------K-HVP-ADN---------------------------VVF---------	445
LaAP1|Q93VM7|/1-460	435	--------Y-AVE-ADK---------------------------LWL---------	444
PvPAP2|Q764C1|/1-457	443	--------K-KVP-TDS---------------------------FVL---------	452
LlAP2|Q8L6L1|/1-463	438	--------Y-AVE-ADK---------------------------LWL---------	447
AtPAP10|Q9SIV9|/1-468	443	--------Y-AVE-GDR---------------------------MWF---------	452
PvPAP1|P80366|/1-459	438	--------V-AVE-ADS---------------------------VWF---------	447
TaACP|C4PKL1|/1-477	437	--------K-HVP-TDN---------------------------VVF---------	446
AtPAP6|Q9C510|/1-466	439	--------E-PVA-ADS---------------------------IML---------	448
AcPAP|Q93WP4|/1-481	442	----------KHIPVDR---------------------------IIF---------	451
AoPAP32|Q9XF09|/1-470	445	--------Y-AVE-ADT---------------------------LWI---------	454
StPAP3|Q6J5M8|/1-477	436	----------NNITTDS---------------------------FTL---------	445
IbPAP1|Q9SE00|/1-473	448	--------A-SVE-ADS---------------------------LWL---------	457
AtPAP26|Q949Y3|/1-475	437	--------K-KVA-TDE---------------------------FVL---------	446
RcPAP3|B9SXP6|/1-488	443	--------N-KVA-TDA---------------------------FVL---------	452
LlAP1|Q8L5E1|/1-477	439	--------K-KVP-IDS---------------------------FIL---------	448
GmPAP3|Q6YGT9|/1-512	475	--------K-KVP-TDS---------------------------FVL---------	484
LaAP2|Q9XJ24|/1-638	466	QAVVATLLF-AVT-GNDSQDTNQNASLLVSARQFVIAMLVIDTWQYF---------	510
LlPPD4|Q8VXF4|/1-629	599	----------GNV-YDF---------------------------FTI--SRDYRDV	614
LlPPD1|Q8VX11|/1-615	585	----------GQV-YDS---------------------------FTI--SRDYRDI	600
LlPPD2|Q8VXF6|/1-612	582	----------GKV-YDS---------------------------FKI--SRDYRDI	597
TnPAP1|Q4RLR4|/1-378	359	----------GKV-IDS---------------------------IWV--VKEKHG-	373
HsPAP7|Q6ZNF0|/1-438	419	----------GKI-VDD---------------------------VWV--VRPLFG-	433
CePAP3|Q9NAM9|/1-418	406	--------T-GKF-LDP---------------------------FVL---------	415
MmPAP7|Q8BX37|/1-438	419	----------GKI-VDD---------------------------VWV--VRPL---	431
DmPAP1|Q9VZ56|/1-458	439	--------N-GAI-IDD---------------------------FWL--VKSKHGS	455
DmPAP2|Q9VZ58|/1-450	430	----------GAI-VDS---------------------------FWV--IKDKHGA	445
AmPAP|A0A087ZWE4|/1-438	413	--------E-GAV-LDH---------------------------VWL---------	422
CePAP1|O01320|/1-419	388	--------------GKQ---------------------------LYS--TIIPTRV	400
DmPAP3|Q9VZ57|/1-453	427	--------K-GEV-IDS---------------------------FWV--VKDKHGP	443
AgPAP|Q7PUN5|/1-463	419	--------E-GAV-IDS---------------------------FTI--IKDEHLP	435

HvPAPhy_a|C4PKL2|/1-544	532	-L--HK---HNST---	538
TaPAPhy_a1|C4PKK7|/1-550	529	-L--HK---HNSS---	535
TaPAPhy_b1|C4PKK9|/1-538	529	-L-------LKSS---	533
TaPAPhy_b2|C4PKL0|/1-537	528	-L-------LKSS---	532
HvPAPhy_b2|C4PKL4|/1-537	528	-L-------LSSS---	532
HvPAPhy_b1|C4PKL3|/1-536	527	-L-------LKSS---	531
OsPAPhy_b|D6QSX9|/1-539	528	-L--IK---SSRN---	534
ZmPAPhy_b|C4PKL6|/1-544	535	-L-------AKTA---	539
MtPAPhy|Q3ZFI1|/1-543	533	PPVMPEE-AHNT--	543
PtPAP3|V9LXK5|/1-564	550	-PVQPEAYRLNKP---	561
NtPAPhy|A5YBN1|/1-551	532	-PVKPK---VIKP---	540
LaPAPhy|D2YZL4|/1-543	530	-PINQK---VCRE---	538
GmPAPhy_b|Q93XG4|/1-547	534	-PIHQR---VNID---	542
AtPAP15|Q9SFU3|/1-532	523	-PLHHR---LVNH---	531
AtaPAPhy_a1|F6MIX0|/1-549	528	-L--HK---HNSS---	534
ScPAPhy_a2|F6MIX4|/1-543	531	-L--HK---HNST---	537
TmPAPhy_a1|F6MIW8|/1-545	524	-L--HK---HNST---	530
TaPAPhy_a3|F6MIW2|/1-539	527	-L--HK---HNST---	533
TaPAPhy_a2|C4PKK8|/1-549	528	-L--HK---HNST---	534
ScPAPhy_a1|F6MIX2|/1-541	527	-LHKHK---HNST---	535
TaPAPhy_b3|F6MIW6|/1-536	527	-L-------LKSS---	531
TmPAPhy_b1|F6MIW9|/1-539	530	-L-------LKSS---	534
AtaPAPhy_b1|F6MIX1|/1-538	529	-L-------LKSS---	533
ScPAPhy_b1|F6MIX5|/1-538	529	-L-------LKSS---	533
RcPAP1|B9RWG6|/1-566	554	-PVKPK-GAINVL---	564
VvPAP|A5BGI6|/1-540	527	-PTLSA---VTKL---	535
PvPAPhy|V7B3Z4|/1-546	533	-PVPQR---VSGD---	541
VrPAPhy|B5ARZ7|/1-547	534	DVPRK----VCRD---	542
AlPAP15|D7L636|/1-532	523	-PLHHR---LVNH---	531
AtPAP23|Q6TPH1|/1-458	452	---------NSLN---	455
GmPAP4|V9HXG4|/1-442	428	---VDQ---KTHE---	434
ZmPAP_c|C4PKL7|/1-566	555	LLQPASA--SSLN---	565
SbPAP|A0A1Z5R9T8|/1-566	555	LLQPTNA--SSLN---	565
HvPAP_c|C4PKL5|/1-564	553	LL-------QPRG---	558
PpPAP|A9SPI2|/1-557	535	-PYSSMKNYRDRK---	546
OsPAP3|Q6ZCX8|/1-622	558	LLQTTS---ASSE---	567
OsPAP4|B8B909|/1-622	558	LLQTTS---ASSE---	567
AtPAP5|Q9C927|/1-396	385	---------KNRY---	388
AtPAP20|Q9LXI7|/1-427	426	------------K---	426
AtPAP22|Q8S340|/1-434		--	
IbPAP3|Q9ZP18|/1-427	412	---------INRY---	415
AtPAP21|Q9LXI4|/1-437	429	---------HSNR---	432
LpPAP|Q9MB07|/1-455	445	---------FNRV---	448
RcPAP2|B9SXP8|/1-463	425	---------HNQY---	428
IbPAP2|Q9SDZ9|/1-465	450	---------SNRF---	453
AtPAP11|Q9SI18|/1-441	430	---------KKRY---	433
GmPAP1|Q09131|/1-464	448	---------FNRY---	451
AtPAP25|O23244|/1-466	449	---------HNRY---	452
AtPAP12|Q38924|/1-469	454	---------LNRF---	457
NtPAP|Q84KZ3|/1-461	450	---------FNRY---	453
MtPAP1|Q4KU02|/1-465	449	---------FNRF---	452
OsPAP2|Q8S505|/1-476	446	---------HNQY---	449
LaAP1|Q93VM7|/1-460	445	---------FNRY---	448
PvPAP2|Q764C1|/1-457	453	---------HNQY---	456
LlAP2|Q8L6L1|/1-463	448	---------FNRY---	451
AtPAP10|Q9SIV9|/1-468	453	---------YNRF---	456
PvPAP1|P80366|/1-459	448	---------FNRH---	451
TaACP|C4PKL1|/1-477	447	---------HNQY---	450
AtPAP6|Q9C510|/1-466	449	---------HNRH---	452
AcPAP|Q93WP4|/1-481	452	---------RNQY---	455
AoPAP32|Q9XF09|/1-470	455	---------FNRY---	458
StPAP3|Q6J5M8|/1-477	446	---------HNQY---	449
IbPAP1|Q9SE00|/1-473	458	---------LNRY---	461
AtPAP26|Q949Y3|/1-475	447	---------HNQY---	450
RcPAP3|B9SXP6|/1-488	453	---------HNQY---	456
LlAP1|Q8L5E1|/1-477	449	---------YNQY---	452
GmPAP3|Q6YGT9|/1-512	485	---------HNQY---	488
LaAP2|Q9XJ24|/1-638	511	---------MHRYMHHNKFLYKHIHSQHHRLIVPYSFGALYNHPLVGLILDTIGGA	557
LlPPD4|Q8VXF4|/1-629	615	---------LARV---	618
LlPPD1|Q8VX11|/1-615	601	---------LACS---	604
LlPPD2|Q8VXF6|/1-612	598	---------LACT---	601
TnPAP1|Q4RLR4|/1-378		--	
HsPAP7|Q6ZNF0|/1-438		--	
CePAP3|Q9NAM9|/1-418	416	---------EKL--	418
MmPAP7|Q8BX37|/1-438	432	---------LGRM---	435
DmPAP1|Q9VZ56|/1-458	456	---------YRN--	458
DmPAP2|Q9VZ58|/1-450		--	
AmPAP|A0A087ZWE4|/1-438	423	---------IKDD---	426
CePAP1|O01320|/1-419	401	IPTDTS---TRST---	410
DmPAP3|Q9VZ57|/1-453	444	---------YQSD---	447
AgPAP|Q7PUN5|/1-463	436	---------YKQL---	439

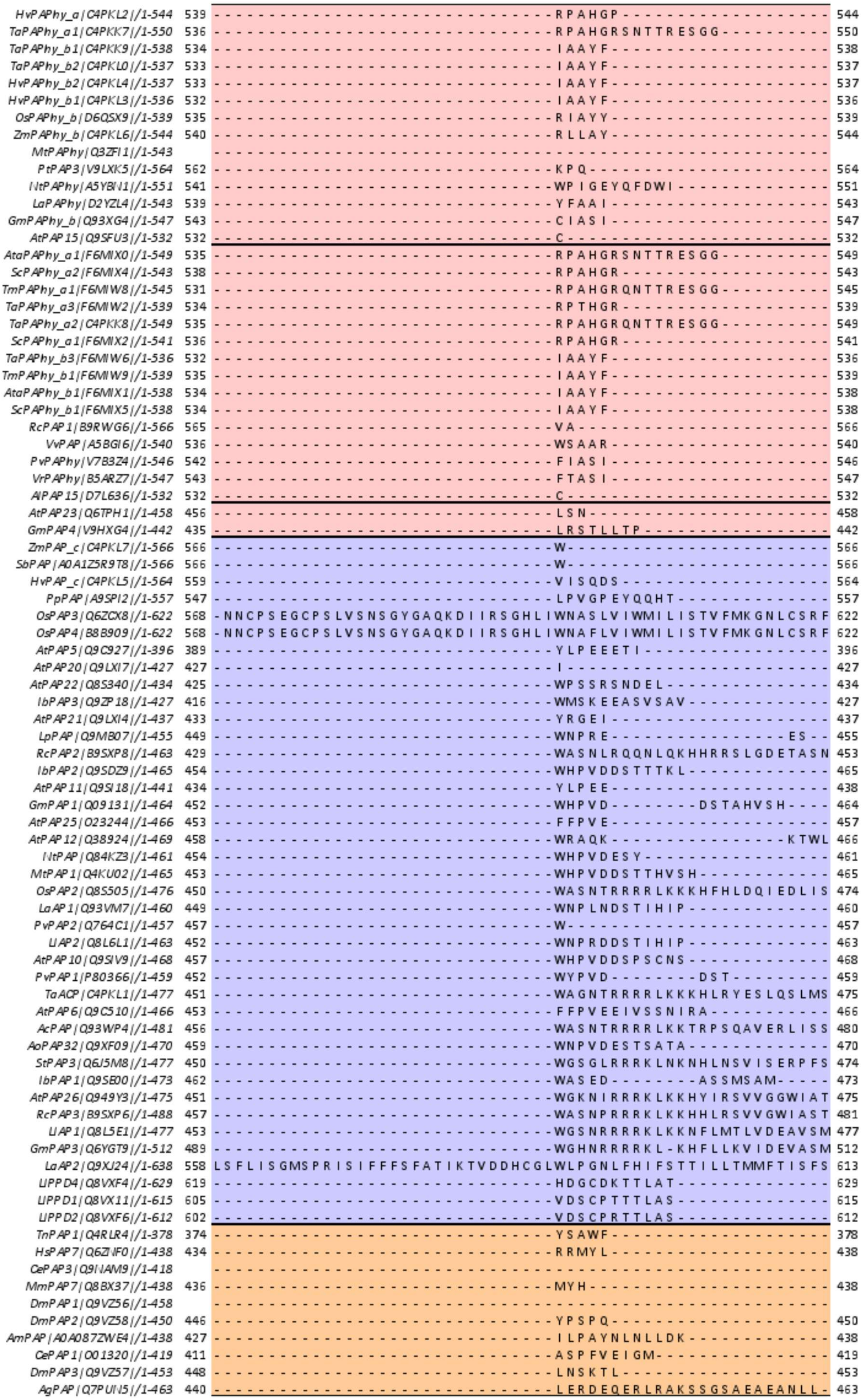
HvPAPhy_a|C4PKL2|/1-544 539 -------------------------------RPAHGP------------------- 544
TaPAPhy_a1|C4PKK7|/1-550 536 -------------------------------RPAHGRSNTTRESGG---------- 550
TaPAPhy_b1|C4PKK9|/1-538 534 -------------------------------IAAYF-------------------- 538
TaPAPhy_b2|C4PKL0|/1-537 533 -------------------------------IAAYF-------------------- 537
HvPAPhy_b2|C4PKL4|/1-537 533 -------------------------------IAAYF-------------------- 537
HvPAPhy_b1|C4PKL3|/1-536 532 -------------------------------IAAYF-------------------- 536
OsPAPhy_b|D6QSX9|/1-539 535 -------------------------------RIAYY-------------------- 539
ZmPAPhy_b|C4PKL6|/1-544 540 -------------------------------RLLAY-------------------- 544
MtPAPhy|Q3ZFI1|/1-543 --
PtPAP3|V9LXK5|/1-564 562 -------------------------------KPQ---------------------- 564
NtPAPhy|A5YBN1|/1-551 541 -------------------------------WPIGEYQFDWI-------------- 551
LaPAPhy|D2YZL4|/1-543 539 -------------------------------YFAAI-------------------- 543
GmPAPhy_b|Q93XG4|/1-547 543 -------------------------------CIASI-------------------- 547
AtPAP15|Q9SFU3|/1-532 532 -------------------------------C------------------------ 532
AtaPAPhy_a1|F6MIX0|/1-549 535 -------------------------------RPAHGRSNTTRESGG---------- 549
ScPAPhy_a2|F6MIX4|/1-543 538 -------------------------------RPAHGR------------------- 543
TmPAPhy_a1|F6MIW8|/1-545 531 -------------------------------RPAHGRQNTTRESGG---------- 545
TaPAPhy_a3|F6MIW2|/1-539 534 -------------------------------RPTHGR------------------- 539
TaPAPhy_a2|C4PKK8|/1-549 535 -------------------------------RPAHGRQNTTRESGG---------- 549
ScPAPhy_a1|F6MIX2|/1-541 536 -------------------------------RPAHGR------------------- 541
TaPAPhy_b3|F6MIW6|/1-536 532 -------------------------------IAAYF-------------------- 536
TmPAPhy_b1|F6MIW9|/1-539 535 -------------------------------IAAYF-------------------- 539
AtaPAPhy_b1|F6MIX1|/1-538 534 -------------------------------IAAYF-------------------- 538
ScPAPhy_b1|F6MIX5|/1-538 534 -------------------------------IAAYF-------------------- 538
RcPAP1|B9RWG6|/1-566 565 -------------------------------VA----------------------- 566
VvPAP|A5BGI6|/1-540 536 -------------------------------WSAAR-------------------- 540
PvPAPhy|V7B3Z4|/1-546 542 -------------------------------FIASI-------------------- 546
VrPAPhy|B5ARZ7|/1-547 543 -------------------------------FTASI-------------------- 547
AlPAP15|D7L636|/1-532 532 -------------------------------C------------------------ 532
AtPAP23|Q6TPH1|/1-458 456 -------------------------------LSN---------------------- 458
GmPAP4|V9HXG4|/1-442 435 -------------------------------LRSTLLTP----------------- 442
ZmPAP_c|C4PKL7|/1-566 566 -------------------------------W------------------------ 566
SbPAP|A0A1Z5R9T8|/1-566 566 -------------------------------W------------------------ 566
HvPAP_c|C4PKL5|/1-564 559 -------------------------------VISQDS------------------- 564
PpPAP|A9SPI2|/1-557 547 -------------------------------LPVGPEYQQHT-------------- 557
OsPAP3|Q6ZCX8|/1-622 568 -NNCPSEGCPSLVSNSGYGAQKDIIRSGHLIWNASLVIWMILISTVFMKGNLCSRF 622
OsPAP4|B8B909|/1-622 568 -NNCPSEGCPSLVSNSGYGAQKDIIRSGHLIWNAFLVIWMILISTVFMKGNLCSRF 622
AtPAP5|Q9C927|/1-396 389 -------------------------------YLPEEETI----------------- 396
AtPAP20|Q9LXI7|/1-427 427 -------------------------------I------------------------ 427
AtPAP22|Q8S340|/1-434 425 -------------------------------WPSSRSNDEL--------------- 434
IbPAP3|Q9ZP18|/1-427 416 -------------------------------WMSKEEASVSAV------------- 427
AtPAP21|Q9LXI4|/1-437 433 -------------------------------YRGEI-------------------- 437
LpPAP|Q9MB07|/1-455 449 -------------------------------WNPRE----------------ES-- 455
RcPAP2|B9SXP8|/1-463 429 -------------------------------WASNLRQQNLQKHHRRSLGDETASN 453
IbPAP2|Q9SDZ9|/1-465 454 -------------------------------WHPVDDSTTTKL------------- 465
AtPAP11|Q9SI18|/1-441 434 -------------------------------YLPEE-------------------- 438
GmPAP1|Q09131|/1-464 452 -------------------------------WHPVD--------DSTAHVSH---- 464
AtPAP25|O23244|/1-466 453 -------------------------------FFPVE-------------------- 457
AtPAP12|Q38924|/1-469 458 -------------------------------WRAQK----------------KTWL 466
NtPAP|Q84KZ3|/1-461 454 -------------------------------WHPVDESY----------------- 461
MtPAP1|Q4KU02|/1-465 453 -------------------------------WHPVDDSTTHVSH------------ 465
OsPAP2|Q8S505|/1-476 450 -------------------------------WASNTRRRRLKKKHFHLDQIEDLIS 474
LaAP1|Q93VM7|/1-460 449 -------------------------------WNPLNDSTIHIP------------- 460
PvPAP2|Q764C1|/1-457 457 -------------------------------W------------------------ 457
LlAP2|Q8L6L1|/1-463 452 -------------------------------WNPRDDSTIHIP------------- 463
AtPAP10|Q9SIV9|/1-468 457 -------------------------------WHPVDDSPSCNS------------- 468
PvPAP1|P80366|/1-459 452 -------------------------------WYPVD--------DST--------- 459
TaACP|C4PKL1|/1-477 451 -------------------------------WAGNTRRRRLKKKHLRYESLQSLMS 475
AtPAP6|Q9C510|/1-466 453 -------------------------------FFPVEEIVSSNIRA----------- 466
AcPAP|Q93WP4|/1-481 456 -------------------------------WASNTRRRRLKKTRPSQAVERLISS 480
AoPAP32|Q9XF09|/1-470 459 -------------------------------WNPVDESTSATA------------- 470
StPAP3|Q6J5M8|/1-477 450 -------------------------------WGSGLRRRKLNKNHLNSVISERPFS 474
IbPAP1|Q9SE00|/1-473 462 -------------------------------WASED--------ASSMSAM----- 473
AtPAP26|Q949Y3|/1-475 451 -------------------------------WGKNIRRRKLKKHYIRSVVGGWIAT 475
RcPAP3|B9SXP6|/1-488 457 -------------------------------WASNPRRRKLKKHHLRSVVGWIAST 481
LlAP1|Q8L5E1|/1-477 453 -------------------------------WGSNRRRRKLKKNFLMTLVDEAVSM 477
GmPAP3|Q6YGT9|/1-512 489 -------------------------------WGHNRRRRKL-KHFLLKVIDEVASM 512
LaAP2|Q9XJ24|/1-638 558 LSFLISGMSPRISIFFFSFATIKTVDDHCGLWLPGNLFHIFSTTILLTMMFTISFS 613
LlPPD4|Q8VXF4|/1-629 619 -------------------------------HDGCDKTTLAT-------------- 629
LlPPD1|Q8VX11|/1-615 605 -------------------------------VDSCPTTTLAS-------------- 615
LlPPD2|Q8VXF6|/1-612 602 -------------------------------VDSCPRTTLAS-------------- 612
TnPAP1|Q4RLR4|/1-378 374 -------------------------------YSAWF-------------------- 378
HsPAP7|Q6ZNF0|/1-438 434 -------------------------------RRMYL-------------------- 438
CePAP3|Q9NAM9|/1-418 --
MmPAP7|Q8BX37|/1-438 436 -------------------------------MYH---------------------- 438
DmPAP1|Q9VZ56|/1-458 --
DmPAP2|Q9VZ58|/1-450 446 -------------------------------YPSPQ-------------------- 450
AmPAP|A0A087ZWE4|/1-438 427 -------------------------------ILPAYNLNLLDK------------- 438
CePAP1|O01320|/1-419 411 -------------------------------ASPFVEIGM---------------- 419
DmPAP3|Q9VZ57|/1-453 448 -------------------------------LNSKTL------------------- 453
AgPAP|Q7PUN5|/1-463 440 -------------------------------LERDEQERLRAKSSGSAEAEANLL- 463

HvPAPhy_a|C4PKL2|/1-544		-------------------------	
TaPAPhy_a1|C4PKK7|/1-550		-------------------------	
TaPAPhy_b1|C4PKK9|/1-538		-------------------------	
TaPAPhy_b2|C4PKL0|/1-537		-------------------------	
HvPAPhy_b2|C4PKL4|/1-537		-------------------------	
HvPAPhy_b1|C4PKL3|/1-536		-------------------------	
OsPAPhy_b|D6QSX9|/1-539		-------------------------	
ZmPAPhy_b|C4PKL6|/1-544		-------------------------	
MtPAPhy|Q3ZFI1|/1-543		-------------------------	
PtPAP3|V9LXK5|/1-564		-------------------------	
NtPAPhy|A5YBN1|/1-551		-------------------------	
LaPAPhy|D2YZL4|/1-543		-------------------------	
GmPAPhy_b|Q93XG4|/1-547		-------------------------	
AtPAP15|Q9SFU3|/1-532		-------------------------	
AtaPAPhy_a1|F6MIX0|/1-549		-------------------------	
ScPAPhy_a2|F6MIX4|/1-543		-------------------------	
TmPAPhy_a1|F6MIW8|/1-545		-------------------------	
TaPAPhy_a3|F6MIW2|/1-539		-------------------------	
TaPAPhy_a2|C4PKK8|/1-549		-------------------------	
ScPAPhy_a1|F6MIX2|/1-541		-------------------------	
TaPAPhy_b3|F6MIW6|/1-536		-------------------------	
TmPAPhy_b1|F6MIW9|/1-539		-------------------------	
AtaPAPhy_b1|F6MIX1|/1-538		-------------------------	
ScPAPhy_b1|F6MIX5|/1-538		-------------------------	
RcPAP1|B9RWG6|/1-566		-------------------------	
VvPAP|A5BGI6|/1-540		-------------------------	
PvPAPhy|V7B3Z4|/1-546		-------------------------	
VrPAPhy|B5ARZ7|/1-547		-------------------------	
AlPAP15|D7L636|/1-532		-------------------------	
AtPAP23|Q6TPH1|/1-458		-------------------------	
GmPAP4|V9HXG4|/1-442		-------------------------	
ZmPAP_c|C4PKL7|/1-566		-------------------------	
SbPAP|A0A1Z5R9T8|/1-566		-------------------------	
HvPAP_c|C4PKL5|/1-564		-------------------------	
PpPAP|A9SPI2|/1-557		-------------------------	
OsPAP3|Q6ZCX8|/1-622		-------------------------	
OsPAP4|B8B909|/1-622		-------------------------	
AtPAP5|Q9C927|/1-396		-------------------------	
AtPAP20|Q9LXI7|/1-427		-------------------------	
AtPAP22|Q8S340|/1-434		-------------------------	
IbPAP3|Q9ZP18|/1-427		-------------------------	
AtPAP21|Q9LXI4|/1-437		-------------------------	
LpPAP|Q9MB07|/1-455		-------------------------	
RcPAP2|B9SXP8|/1-463	454	SENDLPHHTK---------------	463
IbPAP2|Q9SDZ9|/1-465		-------------------------	
AtPAP11|Q9SI18|/1-441	439	----------ETA------------	441
GmPAP1|Q09131|/1-464		-------------------------	
AtPAP25|O23244|/1-466	458	----------ELESGNTRA------	466
AtPAP12|Q38924|/1-469	467	DAF----------------------	469
NtPAP|Q84KZ3|/1-461		-------------------------	
MtPAP1|Q4KU02|/1-465		-------------------------	
OsPAP2|Q8S505|/1-476	475	VF-----------------------	476
LaAP1|Q93VM7|/1-460		-------------------------	
PvPAP2|Q764C1|/1-457		-------------------------	
LlAP2|Q8L6L1|/1-463		-------------------------	
AtPAP10|Q9SIV9|/1-468		-------------------------	
PvPAP1|P80366|/1-459		-------------------------	
TaACP|C4PKL1|/1-477	476	ML-----------------------	477
AtPAP6|Q9C510|/1-466		-------------------------	
AcPAP|Q93WP4|/1-481	481	Y------------------------	481
AoPAP32|Q9XF09|/1-470		-------------------------	
StPAP3|Q6J5M8|/1-477	475	ARL----------------------	477
IbPAP1|Q9SE00|/1-473		-------------------------	
AtPAP26|Q949Y3|/1-475		-------------------------	
RcPAP3|B9SXP6|/1-488	482	DKECDNL------------------	488
LlAP1|Q8L5E1|/1-477		-------------------------	
GmPAP3|Q6YGT9|/1-512		-------------------------	
LaAP2|Q9XJ24|/1-638	614	ATSTTTHSHSLLCGIKSWVPTCLTH	638
LlPPD4|Q8VXF4|/1-629		-------------------------	
LlPPD1|Q8VX11|/1-615		-------------------------	
LlPPD2|Q8VXF6|/1-612		-------------------------	
TnPAP1|Q4RLR4|/1-378		-------------------------	
HsPAP7|Q6ZNF0|/1-438		-------------------------	
CePAP3|Q9NAM9|/1-418		-------------------------	
MmPAP7|Q8BX37|/1-438		-------------------------	
DmPAP1|Q9VZ56|/1-458		-------------------------	
DmPAP2|Q9VZ58|/1-450		-------------------------	
AmPAP|A0A087ZWE4|/1-438		-------------------------	
CePAP1|O01320|/1-419		-------------------------	
DmPAP3|Q9VZ57|/1-453		-------------------------	
AgPAP|Q7PUN5|/1-463		-------------------------	

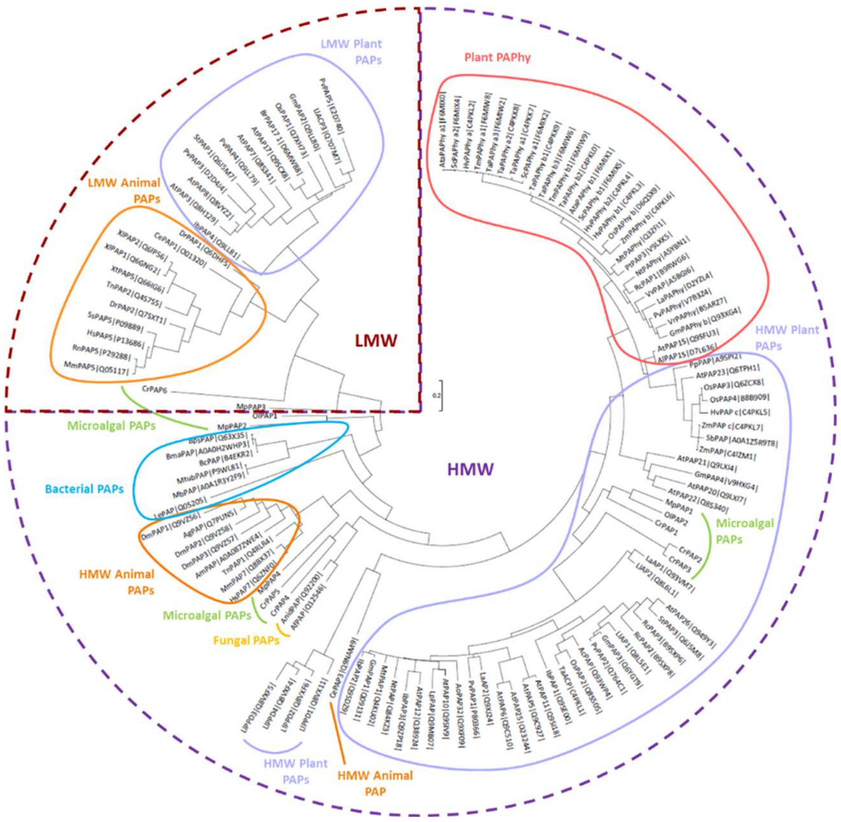

Supplemental Figure 4. Molecular phylogenetic analysis of PAP sequences by the maximum likelihood method. The evolutionary history was inferred by using the Maximum Likelihood method based on the JTT matrix-based model(Jones *et al.*, 1992). The tree with the highest log likelihood (-5950.08) is shown. Initial tree(s) for the heuristic search were obtained automatically by applying Neighbour-Join and BioNJ algorithms to a matrix of pairwise distances estimated using a JTT model, and then selecting the topology with superior log likelihood value. The tree is drawn to scale, with branch lengths measured in the number of substitutions per site. The analysis involved 126 amino acid sequences. All positions containing gaps and missing data were eliminated. There was a total of 59 positions in the final dataset. Evolutionary analyses were conducted in MEGA7 (Kumar *et al.*, 2016).

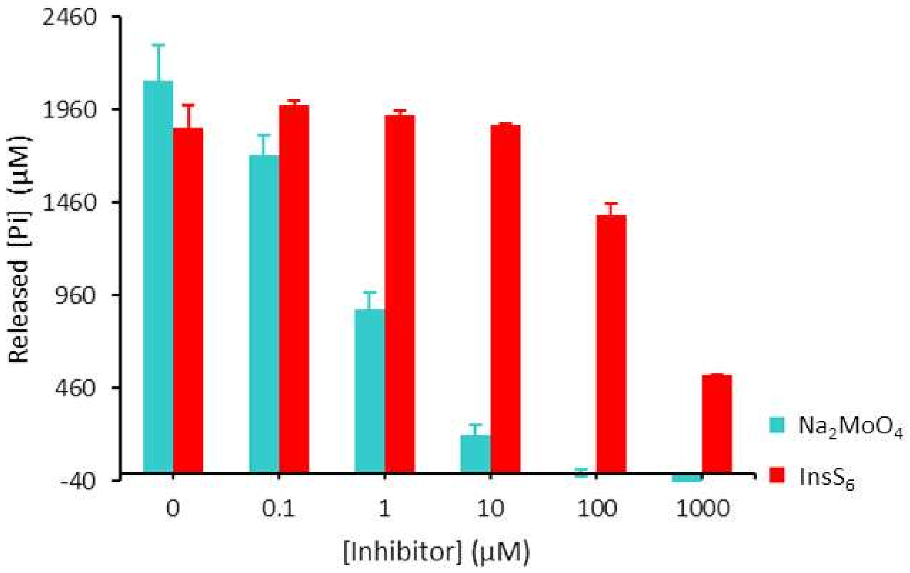

Supplemental Figure 5. Inhibition of phytase activity by *myo*-inositol hexakissulfate ($InsS_6$). Inhibition by sodium molybdate, a known competitive plant PAP inhibitor, is included for reference. Error bars show standard deviations of triplicate measurements.

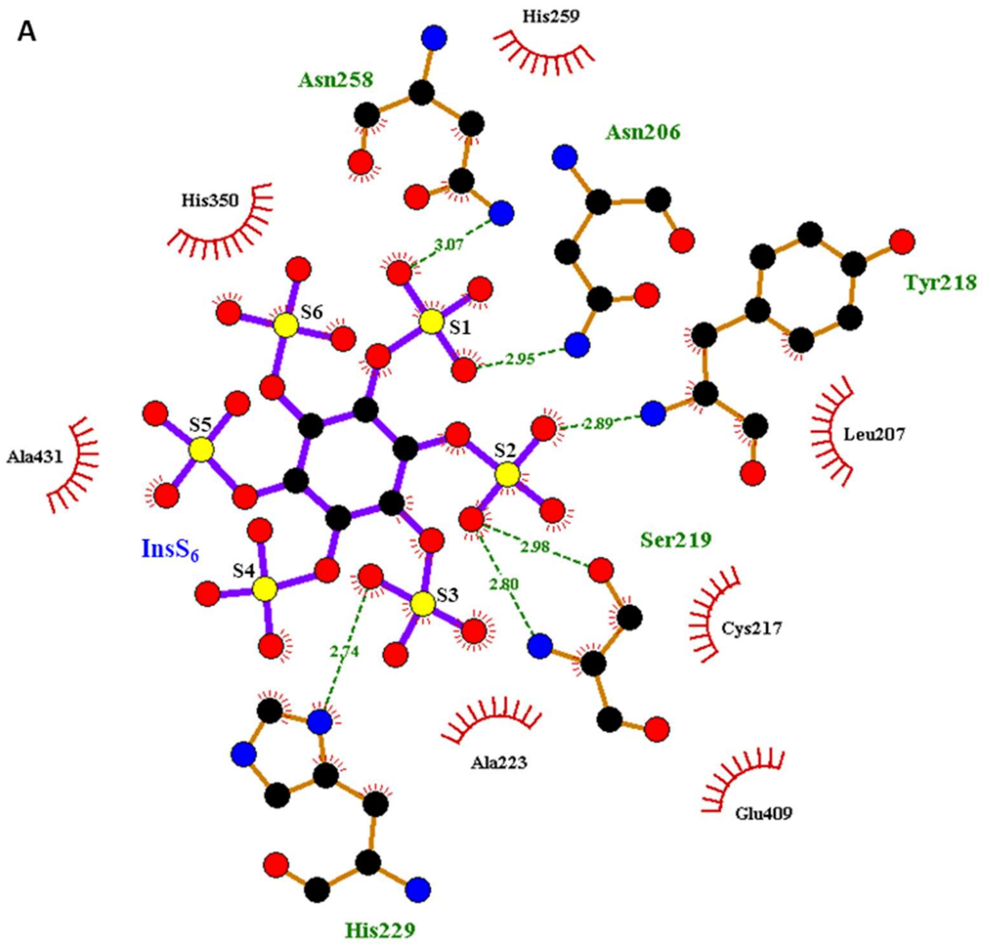

Supplemental Figure 6. Intermolecular interactions in the crystal structure of the TaPAPhy_b2:InsS$_6$ complex. Rendering of interactions in the crystal structure of the complex (PDB entry 6GJ2). Produced using Ligplot+ (Laskowski and Swindells, 2011).

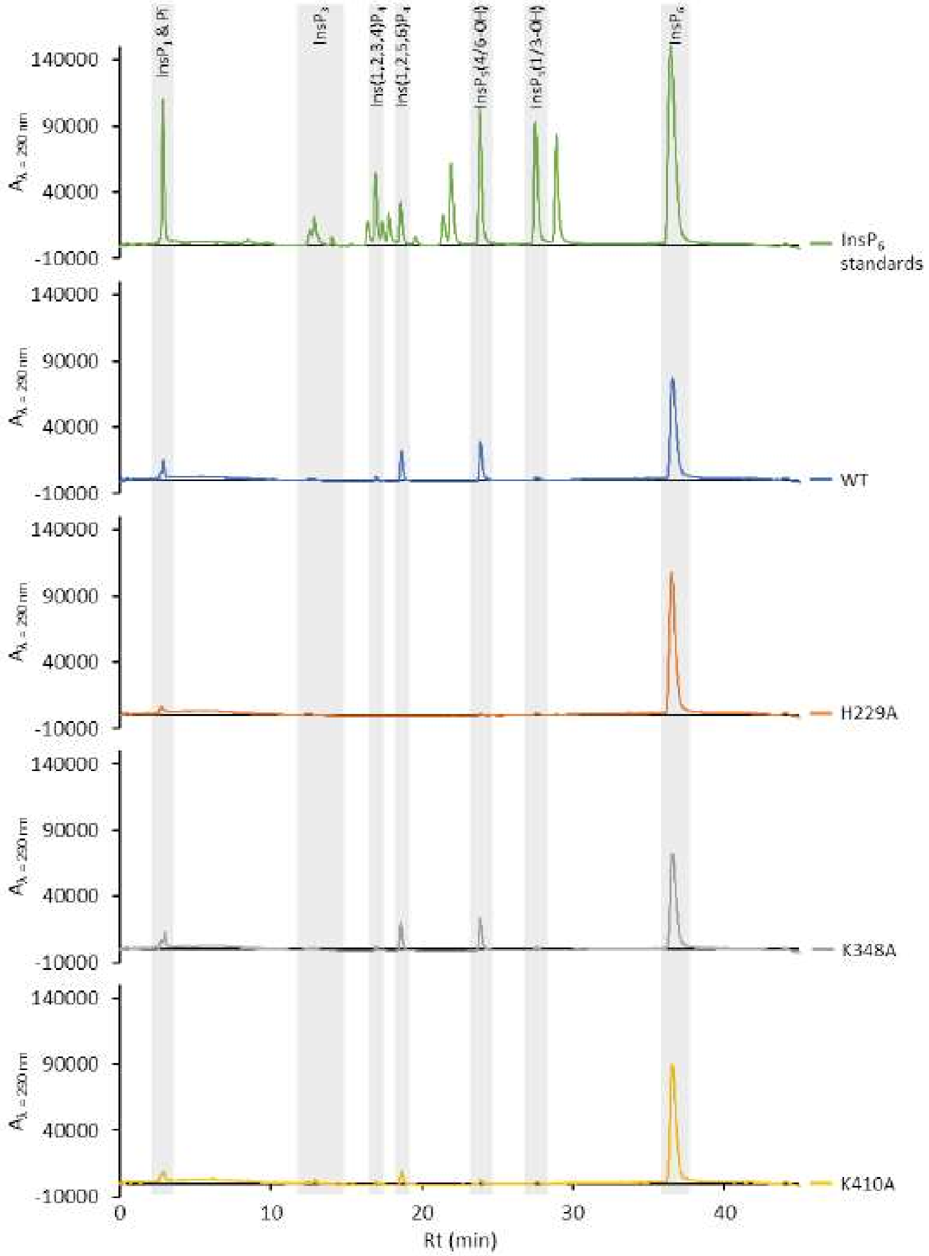

Supplemental Figure 7. HPLC product profiles of recombinant wild type (WT) TaPAPhy_b2 and its active mutants after limited reaction against $InsP_6$. Reactions were performed for 15 min at room temperature with 1 mM $InsP_6$ substrate and 1 µM enzymes in 0.2 M acetate buffer pH 5.5. An acid hydrolysate of $InsP_6$ with relevant peaks labelled for reference is shown ($InsP_5$s are identified by the residual hydroxyl). 'Rt', retention time.

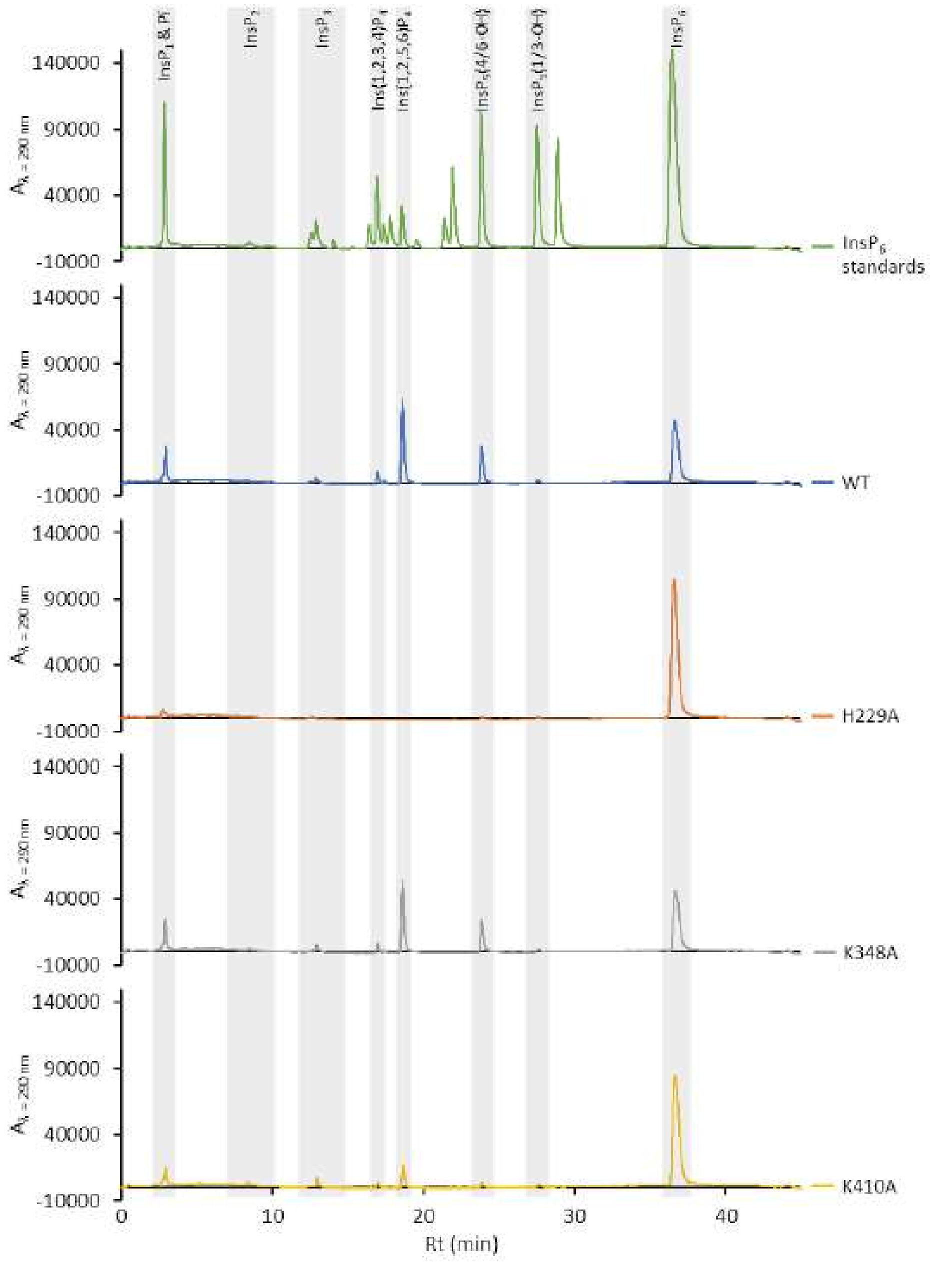

Supplemental Figure 8. HPLC product profiles of recombinant wild type (WT) TaPAPhy_b2 and its active site mutants after progressive reaction against $InsP_6$. Reactions were performed for 30 min at room temperature with 1 mM $InsP_6$ substrate and 1 µM enzymes in 0.2 M acetate buffer pH 5.5. An acid hydrolysate of $InsP_6$ with relevant peaks labelled for reference is shown ($InsP_5$s are identified by the residual hydroxyl). 'Rt', retention time.

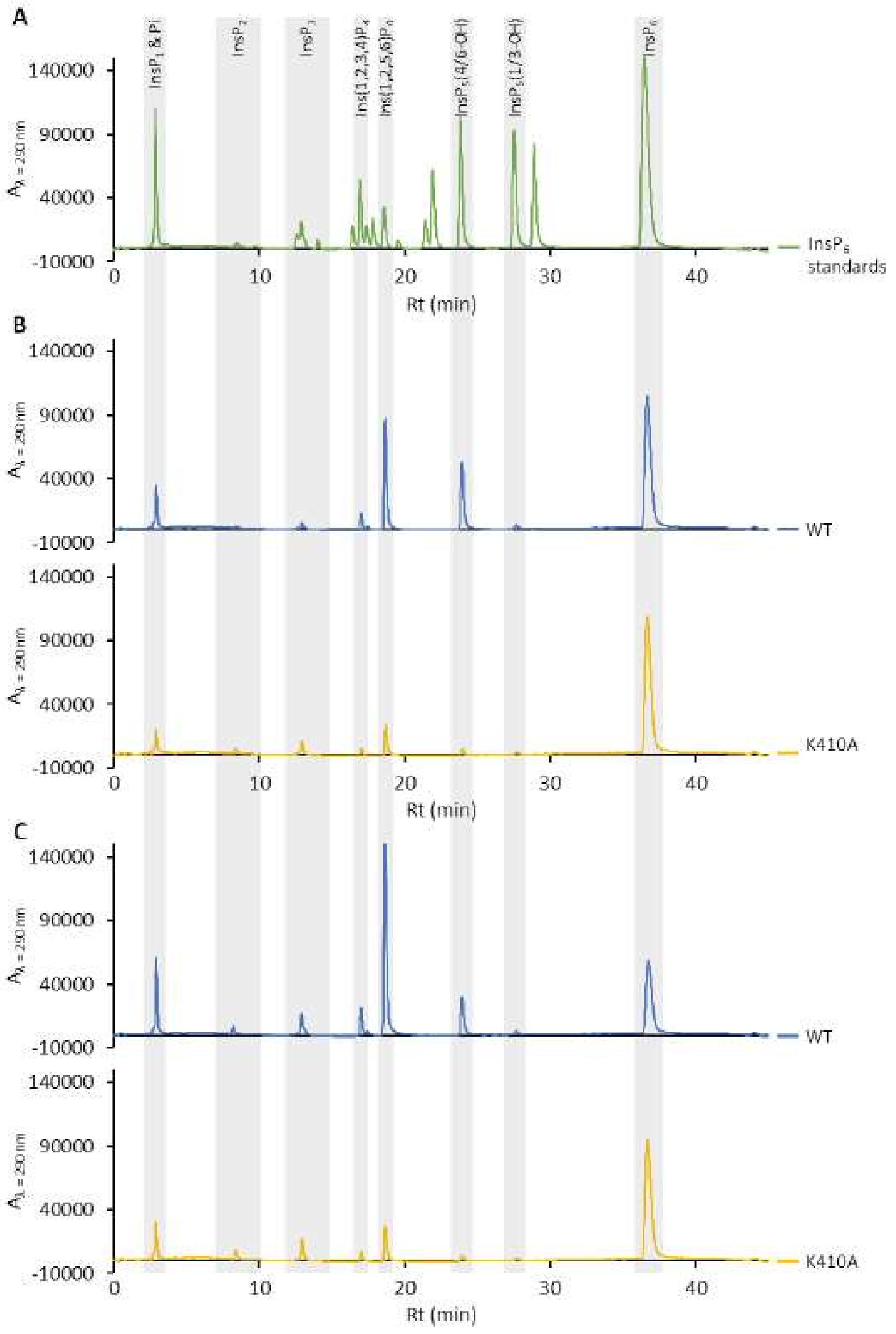

Supplemental Figure 9. HPLC product profiles of recombinant wild type (WT) TaPAPhy_b2 and its K410A mutant after extended reaction against InsP6. Reactions were performed at room temperature with 1 mM $InsP_6$ substrate and 1 µM enzymes in 0.2 M acetate buffer pH 5.5. ‘Rt’, retention time. (**A**) An acid hydrolysate of $InsP_6$ with relevant peaks labelled for reference is shown ($InsP_5$s are identified by the residual hydroxyl). (**B**) 1 h reaction. (**C**) 2 h reaction.

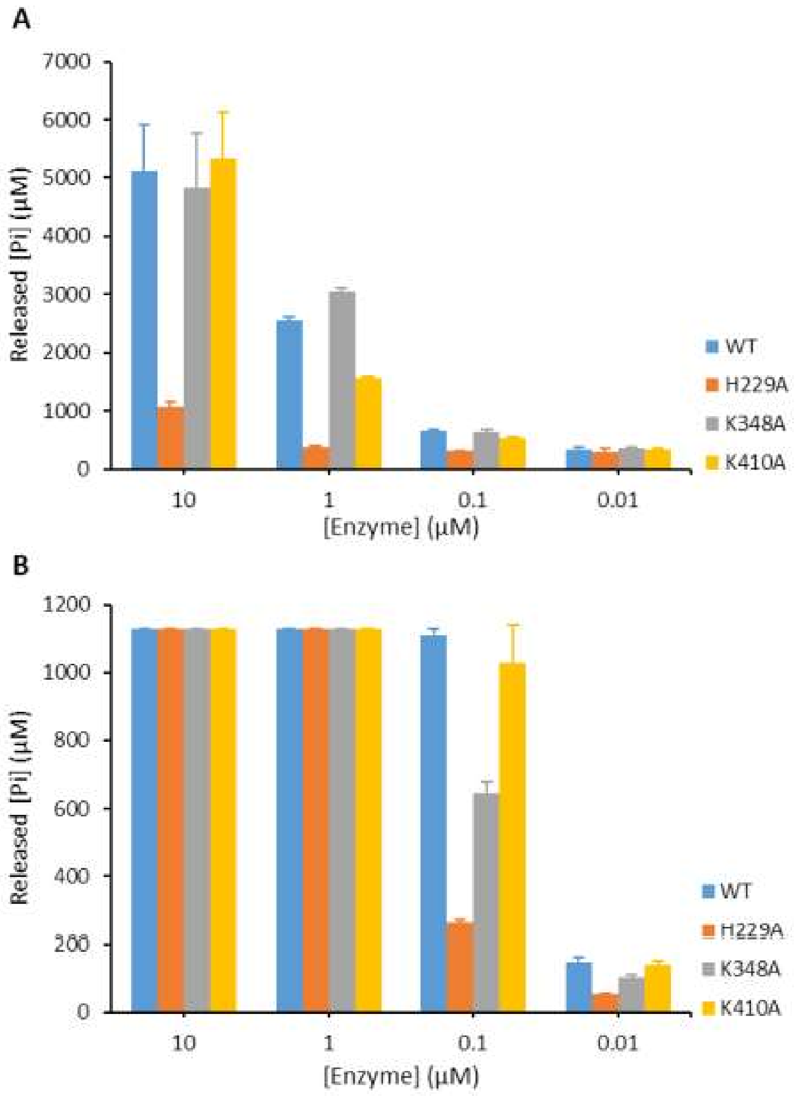

Supplemental Figure 10. Phytase and *p*-nitrophenyl phosphatase activity of WT TaPAPhy_b2 and its mutants. Phosphate release assay with 5 mM $InsP_6$ as substrate in 0.2 M acetate buffer pH 5.5 for 15 min at room temperature. The average phosphate concentration released as a measure of phytase activity of four replicate reactions with decreasing enzyme concentrations is displayed. Error bars represent the standard deviation of the four replicates. (B) Phosphate release assay with 5 mM *p*-nitrophenyl phosphatase (pNPP) as substrate in 0.2M acetate buffer pH 5.5 for 15 min at room temperature. The average phosphate concentration released as a measure of phosphatase activity of four replicate reactions with decreasing enzyme concentrations is displayed. Error bars represent the standard deviation of the four replicates. pNPP background absorbance was subtracted from the measurements. 'Pi', inorganic phosphate.

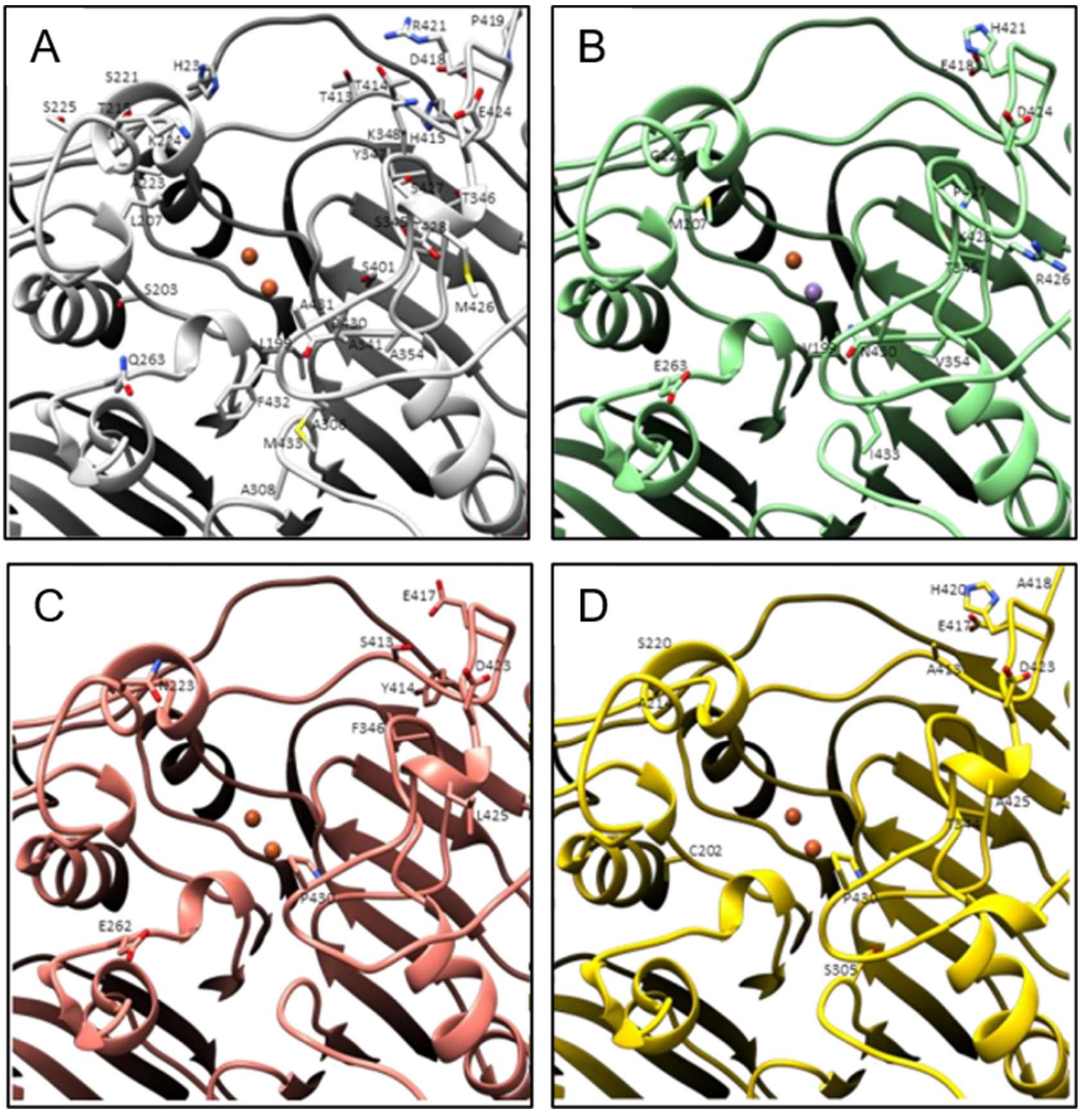

Supplemental Figure 11. Comparison of the active sites of cereal PAPhys. The active sites of the TaPAPhy_b2 crystal structure and plant PAPhy homology models are displayed in cartoon representation with metal ions shown as spheres and coloured by element (i.e. Fe, brown; Mn, lilac). Residues that are not conserved in one or more of the enzymes analysed with respect to TaPAPhy_b2 are shown as sticks, coloured by element (oxygen-red; nitrogen-blue) and labelled. Images created with the UCSF Chimera package (Pettersen *et al.*, 2004). (A) TaPAPhy_b2; (B) HvPAPhy_a; (C) OsPAPhy_b; (D) ZmPAPhy_b.

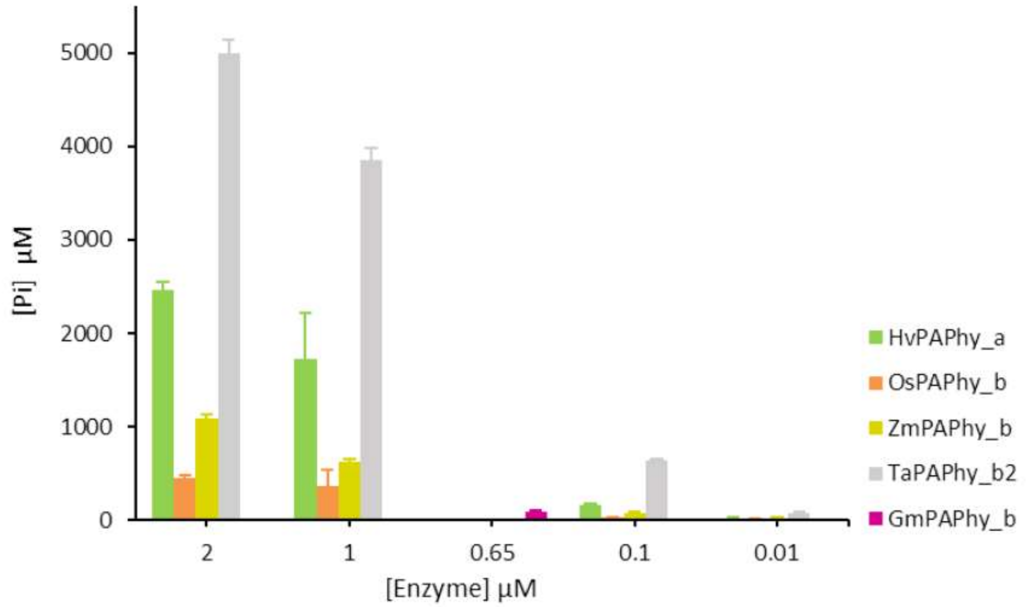

Supplemental Figure 12. Phytase activities of recombinant cereal PAPhy enzymes. Phosphate release assay with 5 mM InsP6 as substrate in 0.2 M acetate buffer pH 5.5 for 15 min at room temperature. The average phosphate concentration released as a measure of phytase activity of four replicate reactions with decreasing enzyme concentrations is displayed. Error bars represent the standard deviation of the four replicates. A single unique concentration with two replicate reactions was assayed for GmPAPhy_b. $InsP_6$ background absorbance was subtracted from the measurements. 'P_i', inorganic phosphate.

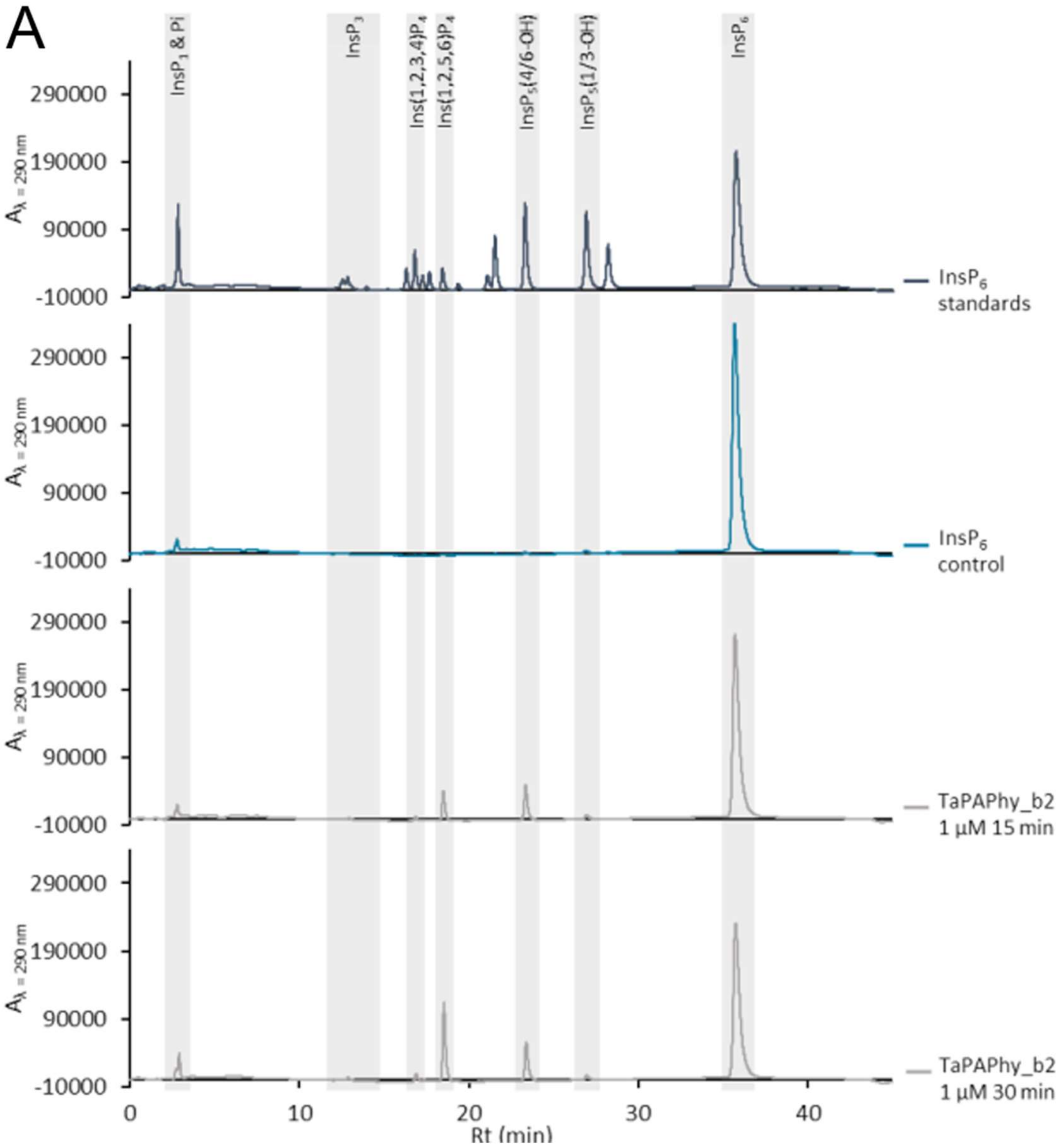

Supplemental Figure 13. Inositol polyphosphate product profiles following hydrolysis of $InsP_6$ by recombinant wheat PAPhy isoform b2 (TaPAPhy_b2). Reactions were performed for varying times (15, 30 mins) at room temperature with 1 mM $InsP_6$ substrate and 1 µM enzyme concentration in 0.2 M acetate buffer pH 5.5. A control reaction in the absence of enzyme was included. An acid hydrolysate of $InsP_6$ with relevant peaks labelled for reference is shown ($InsP_5$ are identified by the residual hydroxyl group). 'Rt', retention time.

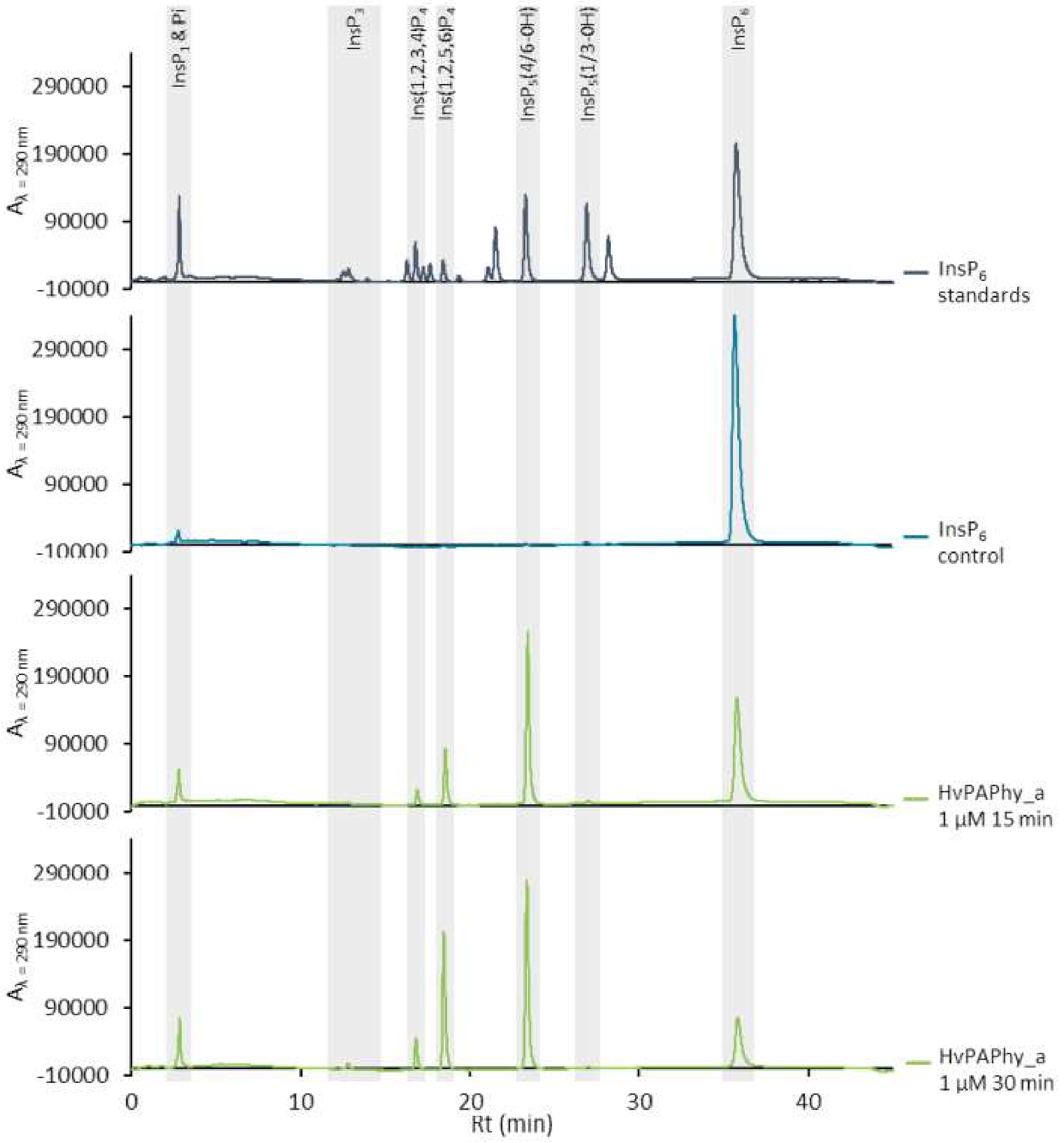

Supplemental Figure 14. Inositol polyphosphate product profiles following hydrolysis of $InsP_6$ by recombinant barley PAPhy isoform a (HvPAPhy_a). Reactions were performed for varying times (15, 30 mins) at room temperature with 1 mM $InsP_6$ substrate and 1 μM enzyme concentration in 0.2 M acetate buffer pH 5.5. A control reaction in the absence of enzyme was included. An acid hydrolysate of $InsP_6$ with relevant peaks labelled for reference is shown ($InsP_5$ are identified by the residual hydroxyl group). 'Rt', retention time.

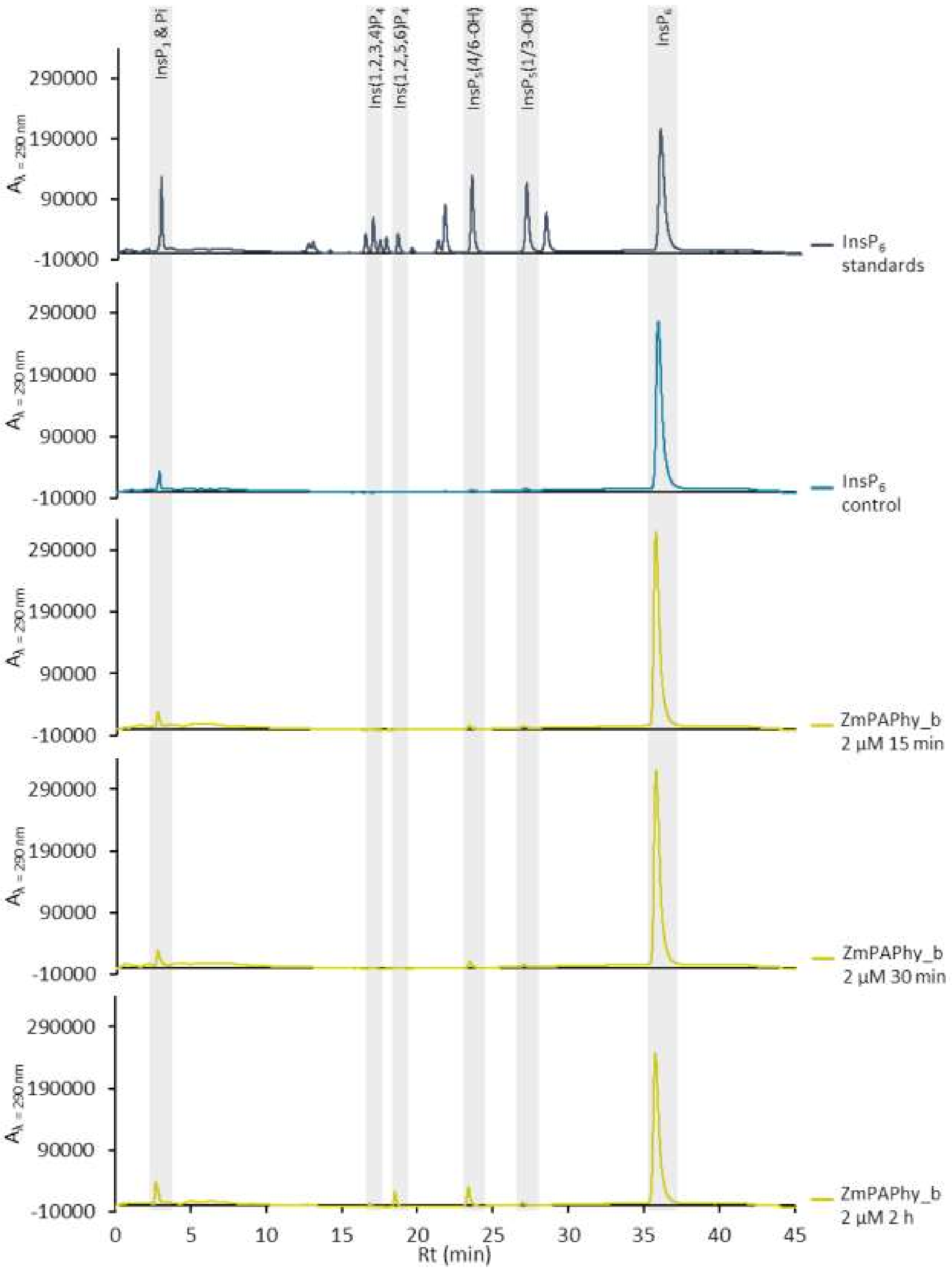

Supplemental Figure 15. Inositol polyphosphate product profiles following hydrolysis of $InsP_6$ by recombinant maize PAPhy isoform b (ZmPAPhy_b). Reactions were performed for varying times (15, 30, 120 mins) at room temperature with 1 mM $InsP_6$ substrate and 2 μM enzyme concentration in 0.2 M acetate buffer pH

5.5. A control reaction in the absence of enzyme was included. An acid hydrolysate of $InsP_6$ with relevant peaks labelled for reference is shown ($InsP_5$ are identified by the residual hydroxyl group). ‘Rt’, retention time.

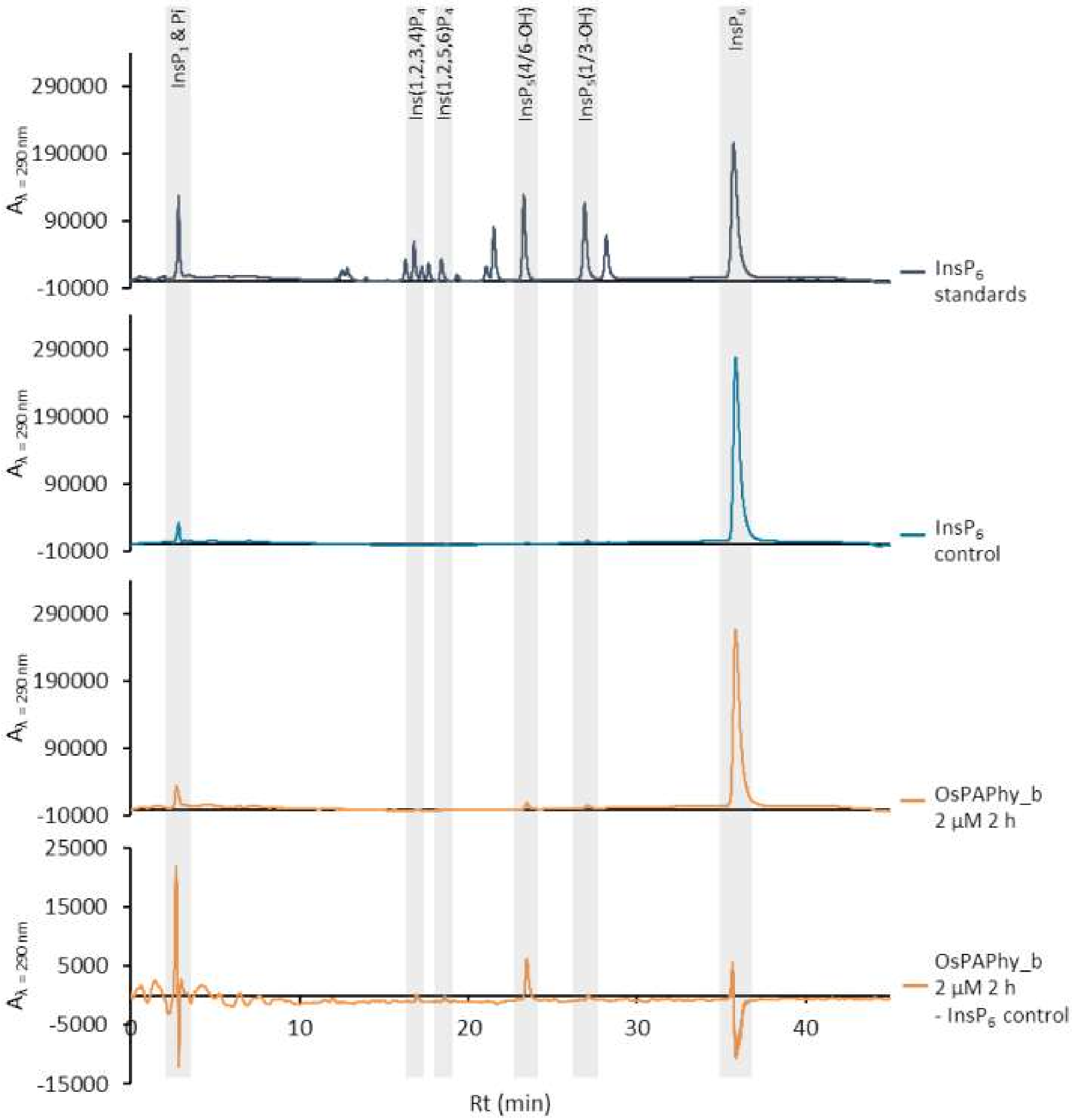

Supplemental Figure 16. Inositol polyphosphate product profiles following hydrolysis of $InsP_6$ by recombinant rice PAPhy isoform b (OsPAPhy_b). Reactions were performed for 120 mins at room temperature with 1 mM $InsP_6$ substrate and 2 µM enzyme concentration in 0.2 M acetate buffer pH 5.5. A control reaction in the absence of enzyme was included. An acid hydrolysate of $InsP_6$ with relevant peaks labelled for reference is shown ($InsP_5$ are identified by the residual

hydroxyl group). ‘Rt’, retention time. Note that for OsPAPhy_b the lower trace shows the profile after subtraction of the substrate-only profile.

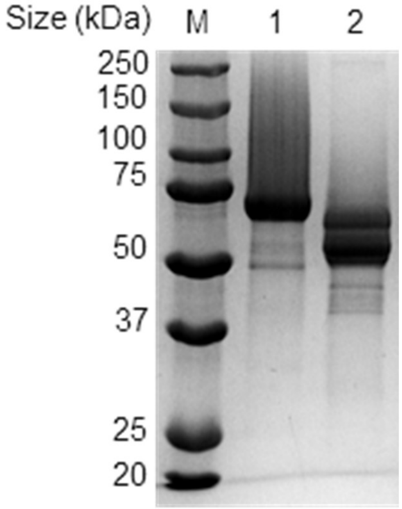

Supplemental Figure 17. Partial enzymatic deglycosylation of TaPAPhy_b2 using recombinant GST-Endo F1. SDS-PAGE (10% v/v acrylamide) gel showing: Lane M, dual colour protein standards (Biorad); Lane 1, glycoengineered TaPAPhy_b2 untreated control; lane 2, TaPAPhy_b2 with GST-Endo F1 (58.7 kDa band).

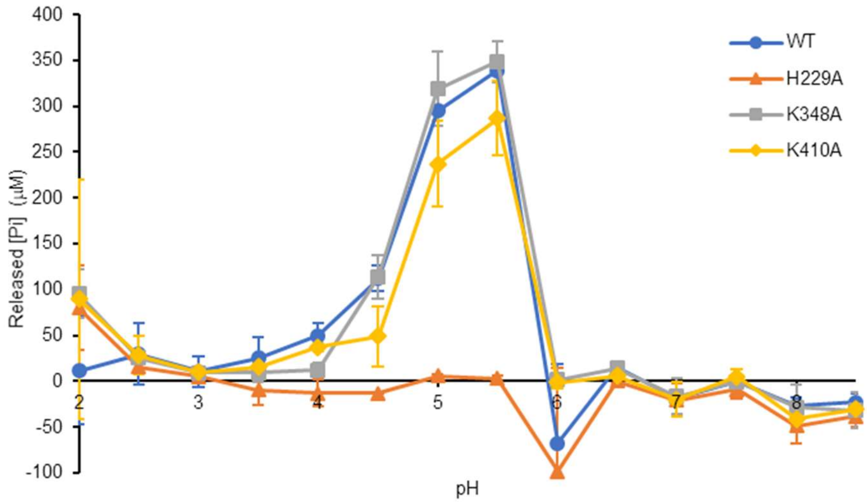

Supplemental Figure 18. pH profile of phytase activities of recombinant wild type (WT) TaPAPhy_b2 and active site mutants.

a

ID	Res.	Metal	Occupancy	*B factor (env.)*[1]	Ligands	*Valence*[2]	*nVECSUM*[3]	*Geometry*[1,4]	*gRMSD(°)*[1]	*Vacancy*[1]	Bidentate	Alt. metal
B:1	FE	Fe	0.47	44.8 (35.3)	O_4	4.2	0.32	Tetrahedral	16.4°	0	0	Cu, Fe
B:2	FE	Fe	0.89	12.8 (18.2)	O_3N_2	2	0.11	Octahedral	9.8°	16%	0	Co, Cu, Zn

Legend: Not applicable | Outlier | *Borderline* | Acceptable

b

ID	Res.	Metal	Occupancy	*B factor (env.)*[1]	Ligands	*Valence*[2]	*nVECSUM*[3]	*Geometry*[1,4]	*gRMSD(°)*[1]	*Vacancy*[1]	Bidentate	Alt. metal
B:1	FE	Fe	0.62	20.1 (24.8)	O_5N_1	2.4	0.11	Octahedral	15.7°	0	0	Co, Cu, Zn
B:2	FE	Fe	0.9	16.7 (23.1)	O_4N_2	1.8	0.058	Octahedral	14.6°	0	0	Co, Cu

c

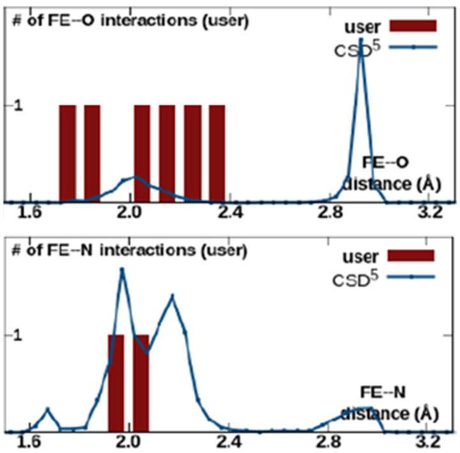

d

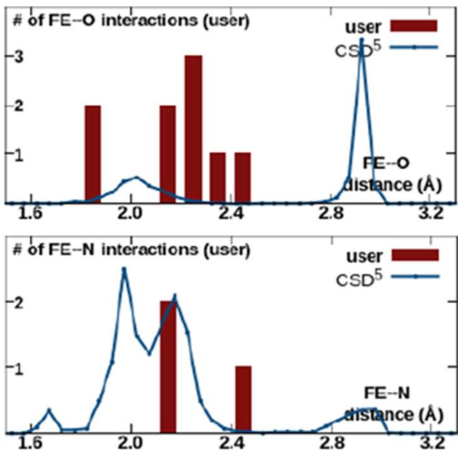

Supplemental Figure 19. Validation of metal-binding sites of TaPAPhy_b2:PO_4 structures. Analysis performed using CheckMyMetal (Zheng *et al.*, 2014). Evaluation of the metal-binding sites in **a**, product-bound and **b**, substrate-bound structures. Metal-ligand distance distributions for **c**, the product-bound and **d**, the substrate-bound structures in comparison with data from the Cambridge Structural Database (CSD).

SUPPLEMENTAL REFERENCES

Adams, P. D. *et al.* (2010) 'PHENIX: A comprehensive Python-based system for macromolecular structure solution', *Acta Crystallographica Section D: Biological Crystallography*, 66(2), pp. 213–221. doi: 10.1107/S0907444909052925.

Altschul, S. F. and Gish, W. (1996) 'Local alignment statistics', *Methods in enzymology*, 266, pp. 460–480. doi: 10.1016/S0076-6879(96)66029-7.

Baker, N. A. *et al.* (2001) 'Electrostatics of nanosystems: Application to microtubules and the ribosome', 98, pp. 10037–10041. Available at: www.pnas.orgcgidoi10.1073pnas.181342398 (Accessed: 28 July 2021).

Bateman, A. *et al.* (2017) 'UniProt: The universal protein knowledgebase', *Nucleic Acids Research*. Oxford University Press, 45(D1), pp. D158–D169. doi: 10.1093/nar/gkw1099.

Bretthauer, R. K. and Castellino, F. J. (1999) 'Glycosylation of Pichia pastoris-derived proteins.', *Biotechnology and applied biochemistry*, 30(3), pp. 193–200. doi: 10.1111/j.1470-8744.1999.tb00770.x.

Bunkóczi, G. and Read, R. J. (2011) 'Improvement of molecular-replacement models with Sculptor', *Acta Crystallographica Section D: Biological Crystallography*. International Union of Crystallography, 67(4), pp. 303–312. doi: 10.1107/S0907444910051218.

Daura, X. *et al.* (1999) 'Peptide folding: When simulation meets experiment', *Angewandte Chemie - International Edition*, 38(1/2), pp. 236–240. doi: 10.1002/(sici)1521-3773(19990115)38:1/2<236::aid-anie236>3.0.co;2-m.

Dionisio, G. *et al.* (2011) 'Cloning and characterization of purple acid phosphatase phytases from wheat, barley, maize, and rice', *Plant Physiology*. American Society of Plant Biologists, 156(3), pp. 1087–1100. doi: 10.1104/pp.110.164756.

Dionisio, G. *et al.* (2012) ‘Glycosylations and truncations of functional cereal phytases expressed and secreted by Pichia pastoris documented by mass spectrometry.’, *Protein expression and purification*. Protein Expr Purif, 82(1), pp. 179–85. doi: 10.1016/j.pep.2011.12.003.

Edgar, R. C. (2004) ‘MUSCLE: Multiple sequence alignment with high accuracy and high throughput’, *Nucleic Acids Research*. Oxford University Press, 32(5), pp. 1792–1797. doi: 10.1093/nar/gkh340.

Emsley, P. *et al.* (2010) ‘Features and development of Coot’, *Acta Crystallographica Section D: Biological Crystallography*. International Union of Crystallography, 66(4), pp. 486–501. doi: 10.1107/S0907444910007493.

Fiser, A. and Sali, A. (2003) ‘ModLoop: automated modeling of loops in protein structures’, *BIOINFORMATICS APPLICATIONS NOTE*, 19(18), pp. 2500–2501. doi: 10.1093/bioinformatics/btg362.

Goodstein, D. M. *et al.* (2012) ‘Phytozome: a comparative platform for green plant genomics’, *Nucleic Acids Research*. Oxford University Press, 40(D1), pp. D1178-86. doi: 10.1093/nar/gkr944.

Gordon, J. C. *et al.* (2005) ‘H++: A server for estimating pKas and adding missing hydrogens to macromolecules’, *Nucleic Acids Research*, 33(SUPPL. 2), pp. 368–371. doi: 10.1093/nar/gki464.

Greiner, R., Jany, K. D. and Larsson Alminger, M. (2000) ‘Identification and Properties of myo -Inositol Hexakisphosphate Phosphohydrolases (Phytases) from Barley (Hordeum vulgare)’, *Journal of Cereal Science*. Academic Press, 31(2), pp. 127–139. doi: 10.1006/JCRS.1999.0254.

Greiner, R., Konietzny, U. and Jany, K. D. (1998) ‘Purification and properties of a phytase from rye’, *Journal of Food Biochemistry*. Blackwell Publishing Ltd, 22(2), pp. 143–161. doi: 10.1111/J.1745-4514.1998.TB00236.X.

Grueninger-Leitch, F. *et al.* (1996) ‘Deglycosylation of proteins for crystallization using recombinant fusion protein glycosidases’, *Protein Science*, 5(12), pp. 2617–2622. doi: 10.1002/pro.5560051224.

Hayakawa, T., Toma, Y. and Igaue, I. ‘Purification and Characterization of Acid Phosphatases with or without Phytase Activity from Rice Bran’, *Agr. Biol. Chem.*, 53(6), pp. 1475–1483. doi: 10.1080/00021369.1989.10869506.

Hegeman, C. E. and Grabau, E. A. (2001) ‘A novel phytase with sequence similarity to purple acid phosphatases is expressed in cotyledons of germinating soybean seedlings’, *Plant Physiology*. Plant Physiol, 126(4), pp. 1598–1608. doi: 10.1104/pp.126.4.1598.

Hess, B. *et al.* (2008) ‘GROMACS 4: Algorithms for highly efficient, load-balanced, and scalable molecular simulation’, *Journal of Chemical Theory and Computation*, 4(3), pp. 435–447. doi: 10.1021/ct700301q.

Jacobs, P. P. *et al.* (2009) ‘Engineering complex-type N-glycosylation in Pichia pastoris using GlycoSwitch technology’, *Nature Protocols*, 4(1), pp. 58–70. doi: 10.1038/nprot.2008.213.

Jones, D. T. *et al.* (1992) ‘The rapid generation of mutation data matrices from protein sequences’, *CABIOS*, 8(3), pp. 275–282. Available at: https://pubmed.ncbi.nlm.nih.gov/1633570/ (Accessed: 1 August 2021).

Kong, Y. *et al.* (2014) ‘GmPAP4, a novel purple acid phosphatase gene isolated from soybean (Glycine max), enhanced extracellular phytate utilization in Arabidopsis thaliana’, *Plant Cell Reports*. Springer Verlag, 33(4), pp. 655–667. doi: 10.1007/s00299-014-1588-5.

Koziara, K. B. *et al.* (2014) ‘Testing and validation of the Automated Topology Builder (ATB) version 2.0: Prediction of hydration free enthalpies’, *Journal of Computer-Aided Molecular Design*, 28(3), pp. 221–233. doi: 10.1007/s10822-014-9713-7.

Kuang, R. *et al.* (2009) 'Molecular and biochemical characterization of AtPAP15, a purple acid phosphatase with phytase activity, in Arabidopsis', *Plant Physiology*. American Society of Plant Biologists, 151(1), pp. 199–209. doi: 10.1104/pp.109.143180.

Kumar, S. *et al.* (2016) 'MEGA7: Molecular Evolutionary Genetics Analysis Version 7.0 for Bigger Datasets', *Mol. Biol. Evol*, 33(7), pp. 1870–1874. doi: 10.1093/molbev/msw054.

Laskowski, R. A. and Swindells, M. B. (2011) 'LigPlot+: Multiple ligand-protein interaction diagrams for drug discovery', *Journal of Chemical Information and Modeling*. J Chem Inf Model, 51(10), pp. 2778–2786. doi: 10.1021/ci200227u.

Lazali, M. *et al.* (2013) 'A phytase gene is overexpressed in root nodules cortex of Phaseolus vulgaris-rhizobia symbiosis under phosphorus deficiency', *Planta*. Planta, 238(2), pp. 317–324. doi: 10.1007/s00425-013-1893-1.

Lazali, M. *et al.* (2014) 'Localization of phytase transcripts in germinating seeds of the common bean (Phaseolus vulgaris L.)', *Planta*. Springer Verlag, 240(3), pp. 471–478. doi: 10.1007/s00425-014-2101-7.

Lin-Cereghino, J. *et al.* (2005) 'Condensed protocol for competent cell preparation and transformation of the methylotrophic yeast Pichia pastoris', *Biotechniques*, 38(1), pp. 44–48.

Lin-Cereghino, J. *et al.* (2008) 'Direct selection of Pichia pastoris expression strains using new G418 resistance vectors', *Yeast*, 25(4), pp. 293–299.

Liu, H. and Naismith, J. H. (2008) 'An efficient one-step site-directed deletion, insertion, single and multiple-site plasmid mutagenesis protocol', *BMC biotechnology*. BMC Biotechnol, 8, p. 91. doi: 10.1186/1472-6750-8-91.

Lung, S. C. *et al.* (2008) 'Phytase activity in tobacco (Nicotiana tabacum) root exudates is exhibited by a purple acid phosphatase', *Phytochemistry*.

Phytochemistry, 69(2), pp. 365–373. doi: 10.1016/j.phytochem.2007.06.036.

Madsen, C. K. *et al.* (2013) ‘High mature grain phytase activity in the Triticeae has evolved by duplication followed by neofunctionalization of the purple acid phosphatase phytase (PAPhy) gene’, *Journal of Experimental Botany*. Oxford Academic, 64(11), pp. 3111–3123. doi: 10.1093/jxb/ert116.

Maruyama, H. *et al.* (2012) ‘Effect of exogenous phosphatase and phytase activities on organic phosphate mobilization in soils with different phosphate adsorption capacities’, *J. Soil Sci. Plant Nutr.* Taylor & Francis Group, 58(1), pp. 41–51. doi: 10.1080/00380768.2012.656298.

McCoy, A. J. *et al.* (2007) ‘Phaser crystallographic software’, *Journal of Applied Crystallography*, 40(4), pp. 658–674. doi: 10.1107/S0021889807021206.

Nagul, E. A. *et al.* (2015) ‘The molybdenum blue reaction for the determination of orthophosphate revisited: Opening the black box’, *Analytica Chimica Acta*. Elsevier B.V., pp. 60–82. doi: 10.1016/j.aca.2015.07.030.

Nakano, T. *et al.* (1999) ‘Purification and Characterization of Phytase from Bran of Triticum aestivum L.cv. Nourin #61.’, *Food Science and Technology Research*. S. Karger AG, 5(1), pp. 18–23. doi: 10.3136/fstr.5.18.

Oostenbrink, C. *et al.* (2004) ‘A biomolecular force field based on the free enthalpy of hydration and solvation: The GROMOS force-field parameter sets 53A5 and 53A6’, *Journal of Computational Chemistry*, 25(13), pp. 1656–1676. doi: 10.1002/jcc.20090.

Pettersen, E. F. *et al.* (2004) ‘UCSF Chimera - A visualization system for exploratory research and analysis’, *Journal of Computational Chemistry*, 25(13), pp. 1605–1612. doi: 10.1002/jcc.20084.

Phillippy, B. Q. and Bland, J. M. (1988) ‘Gradient ion chromatography of inositol phosphates’, *Analytical Biochemistry*, 175(1), pp. 162–166. doi: 10.1016/0003-2697(88)90374-0.

Rivera-Solís, R. A. *et al.* (2014) ‘Chlamydomonas reinhardtii has a small family of purple acid phosphatase homologue genes that are differentially expressed in response to phytate’, *Annals of Microbiology*, 64(2), pp. 551–559. doi: 10.1007/s13213-013-0688-8.

Schenk, G. *et al.* (2008) ‘Crystal structures of a purple acid phosphatase, representing different steps of this enzyme’s catalytic cycle’, *BMC Struct Biol*, 8, p. 6. doi: 10.1186/1472-6807-8-6.

Schrodinger LLC (2015) ‘The PyMOL Molecular Graphics System, Version 1.3’.

Shu, B., Wang, P. and Xia, R. X. (2015) ‘Characterisation of the phytase gene in trifoliate orange (Poncirus trifoliata (L.) Raf.) seedlings’, *Scientia Horticulturae*. Elsevier, 194, pp. 222–229. doi: 10.1016/J.SCIENTA.2015.08.028.

Singh, P. *et al.* (2013) ‘Characterization and expression of codon optimized soybean phytase gene in E. coli’, *Indian Journal of Biochemistry and Biophysics*, 50(6), pp. 537–547. Available at: http://www.ncbi.nlm.nih.gov/pubmed/24772979 (Accessed: 31 March 2020).

Veiga, N. *et al.* (2014) ‘Coordination, microprotonation equilibria and conformational changes of *myo*-inositol hexakisphosphate with pertinence to its biological function’, *Dalton Trans.* The Royal Society of Chemistry, 43(43), pp. 16238–16251. doi: 10.1039/C4DT01350F.

Wang, X. *et al.* (2009) ‘Overexpressing AtPAP15 enhances phosphorus efficiency in soybean’, *Plant Physiology*. American Society of Plant Biologists, 151(1), pp. 233–240. doi: 10.1104/pp.109.138891.

Waterhouse, A. M. *et al.* (2009) ‘Sequence analysis Jalview Version 2-a multiple sequence alignment editor and analysis workbench’, *BIOINFORMATICS APPLICATIONS NOTE*, 25(9), pp. 1189–1191. doi: 10.1093/bioinformatics/btp033.

Wilkins, M. R. *et al.* (1999) ‘Protein identification and analysis tools in the ExPASy

server.’, *Methods in molecular biology (Clifton, N.J.)*. Humana Press, pp. 531–552. doi: 10.1385/1-59259-584-7:531.

Winter, G., Lobley, C. M. C. and Prince, S. M. (2013) ‘Decision making in xia2’, *Acta Crystallogr D Biol Crystallogr.*, 69(7), pp. 1260–1273. doi: 10.1107/S0907444913015308.

Wongkaew, A., Srinives, P. and Nakasathien, S. (2013) ‘Isolation and characterization of purple acid phosphatase gene during seedling development in mungbean’, *Biologia Plantarum*, 57(2), pp. 267–273. doi: 10.1007/s10535-012-0292-y.

Xiao, K. *et al.* (2006) ‘Ectopic Expression of a Phytase Gene from Medicago truncatula Barrel Medic Enhances Phosphorus Absorption in Plants’, *Journal of Integrative Plant Biology*. John Wiley & Sons, Ltd, 48(1), pp. 35–43. doi: 10.1111/J.1744-7909.2006.00189.X.

Xiao, K., Harrison, M. J. and Wang, Z. Y. (2005) ‘Transgenic expression of a novel M. truncatula phytase gene results in improved acquisition of organic phosphorus by Arabidopsis’, *Planta*. Planta, 222(1), pp. 27–36. doi: 10.1007/s00425-005-1511-y.

Zhang, W. *et al.* (2008) ‘An Arabidopsis Purple Acid Phosphatase with Phytase Activity Increases Foliar Ascorbate’, *Plant Physiology*, 146(2), pp. 431–440. doi: 10.1104/pp.107.109934.

Zheng, H. *et al.* (2014) ‘Validation of metal-binding sites in macromolecular structures with the CheckMyMetal web server’, *Nat. Protoc.*, 9(1), pp. 156–170. doi: 10.1038/nprot.2013.172.

Zhu, H. *et al.* (2005) ‘Expression patterns of purple acid phosphatase genes in Arabidopsis organs and functional analysis of AtPAP23 predominantly transcribed in flower’, *Plant Molecular Biology*. Springer, 59(4), pp. 581–594. doi: 10.1007/s11103-005-0183-0.

VALIDATION REPORTS FOR PDB ENTRIES

1. 6GIT. PURPLE ACID PHYTASE FROM WHEAT ISOFORM B2 - PRODUCT COMPLEX
2. 6GIZ. PURPLE ACID PHYTASE FROM WHEAT ISOFORM B2 - SUBSTRATE COMPLEX
3. 6GJ2. PURPLE ACID PHYTASE FROM WHEAT ISOFORM B2 - COMPLEX WITH INOSITOL HEXASULPHATE
4. 6GJA. PURPLE ACID PHYTASE FROM WHEAT ISOFORM B2 - H229A MUTANT

Full wwPDB X-ray Structure Validation Report (i)

Aug 10, 2020 – 11:15 AM BST

PDB ID	:	6GIT
Title	:	PURPLE ACID PHYTASE FROM WHEAT ISOFORM B2 - PRODUCT COMPLEX
Authors	:	Faba-Rodriguez, R.; Brearley, C.A.; Hemmings, A.M.
Deposited on	:	2018-05-15
Resolution	:	1.42 Å(reported)

This is a Full wwPDB X-ray Structure Validation Report for a publicly released PDB entry.

We welcome your comments at *validation@mail.wwpdb.org*
A user guide is available at
https://www.wwpdb.org/validation/2017/XrayValidationReportHelp
with specific help available everywhere you see the (i) symbol.

The following versions of software and data (see references (i)) were used in the production of this report:

MolProbity	:	4.02b-467
Mogul	:	1.8.5 (274361), CSD as541be (2020)
Xtriage (Phenix)	:	1.13
EDS	:	2.13.1
Percentile statistics	:	20191225.v01 (using entries in the PDB archive December 25th 2019)
Refmac	:	5.8.0158
CCP4	:	7.0.044 (Gargrove)
Ideal geometry (proteins)	:	Engh & Huber (2001)
Ideal geometry (DNA, RNA)	:	Parkinson et al. (1996)
Validation Pipeline (wwPDB-VP)	:	2.13.1

1 Overall quality at a glance

The following experimental techniques were used to determine the structure:
X-RAY DIFFRACTION

The reported resolution of this entry is 1.42 Å.

Percentile scores (ranging between 0-100) for global validation metrics of the entry are shown in the following graphic. The table shows the number of entries on which the scores are based.

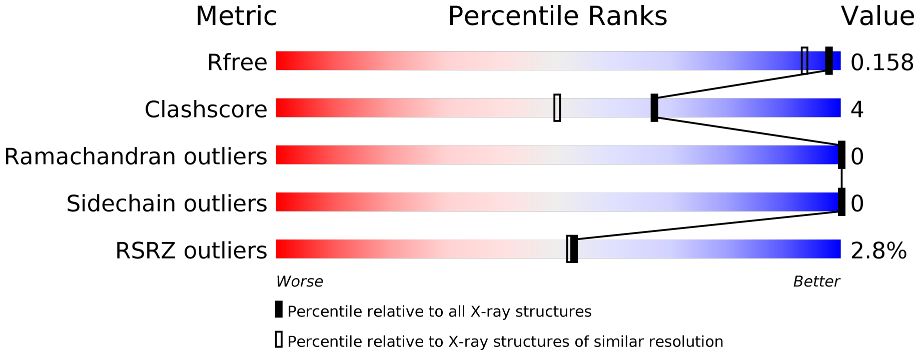

Metric	Whole archive (#Entries)	Similar resolution (#Entries, resolution range(Å))
R_{free}	130704	2579 (1.44-1.40)
Clashscore	141614	2696 (1.44-1.40)
Ramachandran outliers	138981	2632 (1.44-1.40)
Sidechain outliers	138945	2631 (1.44-1.40)
RSRZ outliers	127900	2528 (1.44-1.40)

The table below summarises the geometric issues observed across the polymeric chains and their fit to the electron density. The red, orange, yellow and green segments on the lower bar indicate the fraction of residues that contain outliers for >=3, 2, 1 and 0 types of geometric quality criteria respectively. A grey segment represents the fraction of residues that are not modelled. The numeric value for each fraction is indicated below the corresponding segment, with a dot representing fractions <=5% The upper red bar (where present) indicates the fraction of residues that have poor fit to the electron density. The numeric value is given above the bar.

Mol	Chain	Length	Quality of chain
1	A	516	3% 92% 6% •
2	B	2	100%

2 Entry composition (i)

There are 10 unique types of molecules in this entry. The entry contains 5093 atoms, of which 283 are hydrogens and 0 are deuteriums.

In the tables below, the ZeroOcc column contains the number of atoms modelled with zero occupancy, the AltConf column contains the number of residues with at least one atom in alternate conformation and the Trace column contains the number of residues modelled with at most 2 atoms.

- Molecule 1 is a protein called Purple acid phosphatase.

Mol	Chain	Residues	Atoms					ZeroOcc	AltConf	Trace
			Total	C	N	O	S			
1	A	507	4078	2597	684	772	25	0	24	0

There are 6 discrepancies between the modelled and reference sequences:

Chain	Residue	Modelled	Actual	Comment	Reference
A	511	HIS	-	expression tag	UNP C4PKL0
A	512	HIS	-	expression tag	UNP C4PKL0
A	513	HIS	-	expression tag	UNP C4PKL0
A	514	HIS	-	expression tag	UNP C4PKL0
A	515	HIS	-	expression tag	UNP C4PKL0
A	516	HIS	-	expression tag	UNP C4PKL0

- Molecule 2 is an oligosaccharide called 2-acetamido-2-deoxy-beta-D-glucopyranose-(1-4)-2-acetamido-2-deoxy-beta-D-glucopyranose.

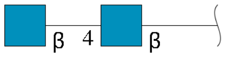

Mol	Chain	Residues	Atoms					ZeroOcc	AltConf	Trace
			Total	C	H	N	O			
2	B	2	55	16	27	2	10	0	0	0

- Molecule 3 is FE (III) ION (three-letter code: FE) (formula: Fe).

Mol	Chain	Residues	Atoms		ZeroOcc	AltConf
			Total	Fe		
3	A	2	2	2	0	0

- Molecule 4 is 2-acetamido-2-deoxy-beta-D-glucopyranose (three-letter code: NAG) (formula: $C_8H_{15}NO_6$).

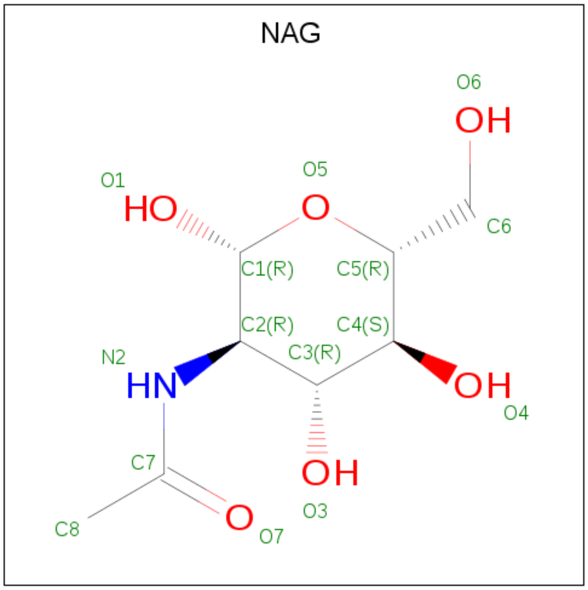

Mol	Chain	Residues	Atoms	ZeroOcc	AltConf
4	A	1	Total C H N O<br>28 8 14 1 5	0	0
4	A	1	Total C H N O<br>28 8 14 1 5	0	0
4	A	1	Total C H N O<br>28 8 14 1 5	0	0
4	A	1	Total C H N O<br>28 8 14 1 5	0	0
4	A	1	Total C H N O<br>28 8 14 1 5	0	0
4	A	1	Total C H N O<br>28 8 14 1 5	0	0

- Molecule 5 is PHOSPHATE ION (three-letter code: PO4) (formula: O_4P).

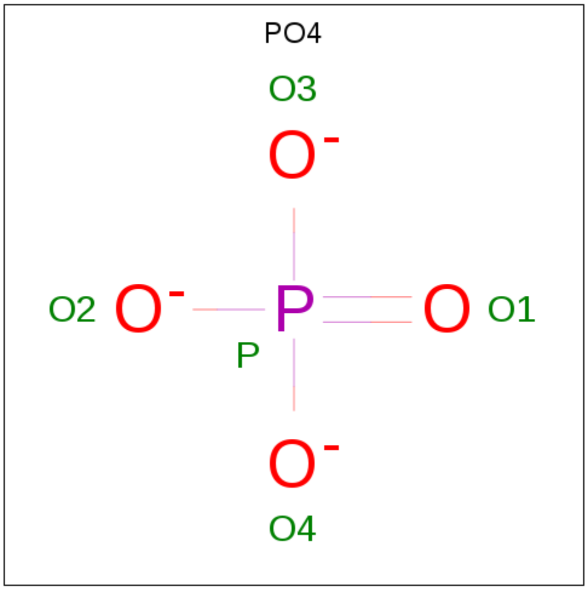

Mol	Chain	Residues	Atoms	ZeroOcc	AltConf
5	A	1	Total O P<br>5 4 1	0	0
5	A	1	Total O P<br>5 4 1	0	0

- Molecule 6 is DI(HYDROXYETHYL)ETHER (three-letter code: PEG) (formula: $C_4H_{10}O_3$).

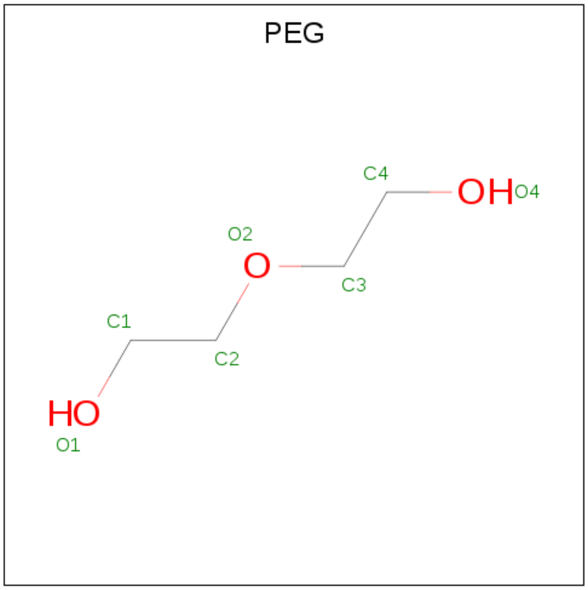

Mol	Chain	Residues	Atoms	ZeroOcc	AltConf
6	A	1	Total C H O<br>17 4 10 3	0	0
6	A	1	Total C H O<br>17 4 10 3	0	0

Continued on next page...

Continued from previous page...

Mol	Chain	Residues	Atoms	ZeroOcc	AltConf
6	A	1	Total C H O<br>17 4 10 3	0	0
6	A	1	Total C H O<br>17 4 10 3	0	0
6	A	1	Total C H O<br>17 4 10 3	0	0

- Molecule 7 is TRIETHYLENE GLYCOL (three-letter code: PGE) (formula: $C_6H_{14}O_4$).

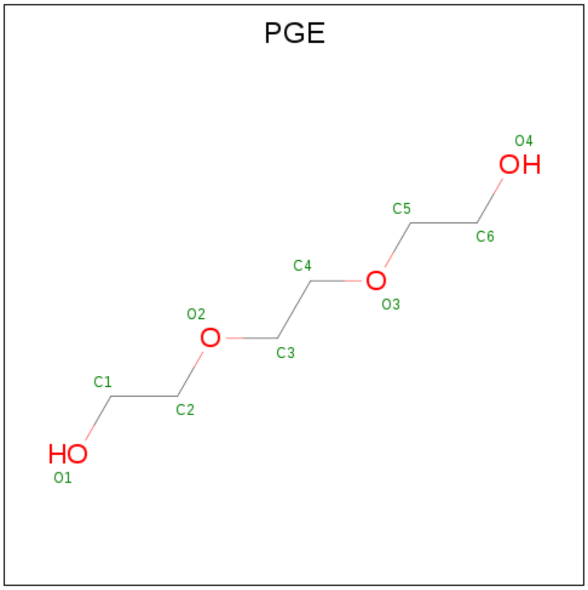

Mol	Chain	Residues	Atoms	ZeroOcc	AltConf
7	A	1	Total C H O<br>24 6 14 4	0	0
7	A	1	Total C H O<br>24 6 14 4	0	0

- Molecule 8 is PENTAETHYLENE GLYCOL (three-letter code: 1PE) (formula: $C_{10}H_{22}O_6$).

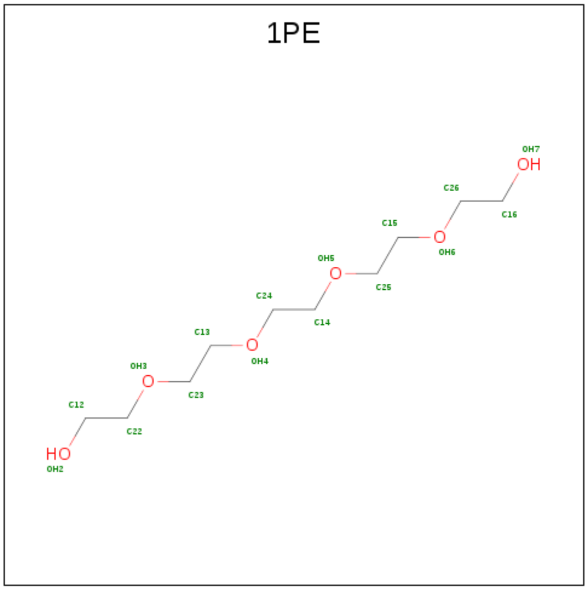

Mol	Chain	Residues	Atoms				ZeroOcc	AltConf
			Total	C	H	O		
8	A	1	38	10	22	6	0	0

- Molecule 9 is 1,2-ETHANEDIOL (three-letter code: EDO) (formula: $C_2H_6O_2$).

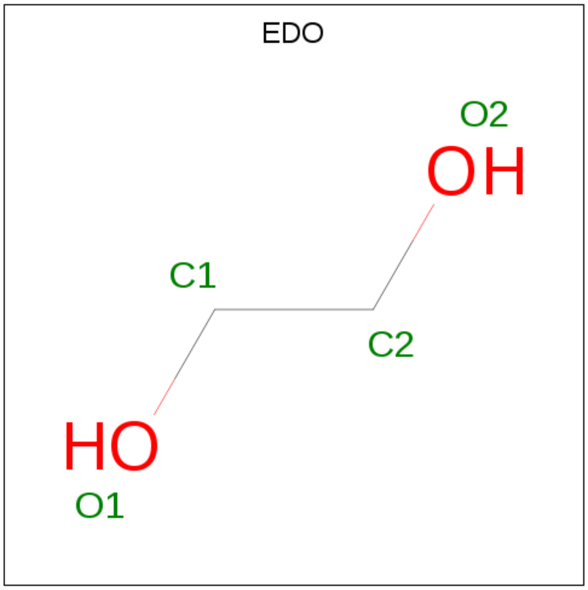

Mol	Chain	Residues	Atoms				ZeroOcc	AltConf
			Total	C	H	O		
9	A	1	10	2	6	2	0	0
9	A	1	10	2	6	2	0	0
9	A	1	10	2	6	2	0	0

Continued on next page...

Continued from previous page...

Mol	Chain	Residues	Atoms	ZeroOcc	AltConf
9	A	1	Total C H O<br>10 2 6 2	0	0
9	A	1	Total C H O<br>10 2 6 2	0	0
9	A	1	Total C H O<br>10 2 6 2	0	0
9	A	1	Total C H O<br>10 2 6 2	0	0
9	A	1	Total C H O<br>10 2 6 2	0	0
9	A	1	Total C H O<br>10 2 6 2	0	0
9	A	1	Total C H O<br>10 2 6 2	0	0
9	A	1	Total C H O<br>10 2 6 2	0	0
9	A	1	Total C H O<br>10 2 6 2	0	0

- Molecule 10 is water.

Mol	Chain	Residues	Atoms	ZeroOcc	AltConf
10	A	489	Total O<br>489 489	0	0

3 Residue-property plots (i)

These plots are drawn for all protein, RNA, DNA and oligosaccharide chains in the entry. The first graphic for a chain summarises the proportions of the various outlier classes displayed in the second graphic. The second graphic shows the sequence view annotated by issues in geometry and electron density. Residues are color-coded according to the number of geometric quality criteria for which they contain at least one outlier: green = 0, yellow = 1, orange = 2 and red = 3 or more. A red dot above a residue indicates a poor fit to the electron density (RSRZ > 2). Stretches of 2 or more consecutive residues without any outlier are shown as a green connector. Residues present in the sample, but not in the model, are shown in grey.

- Molecule 1: Purple acid phosphatase

Chain A:

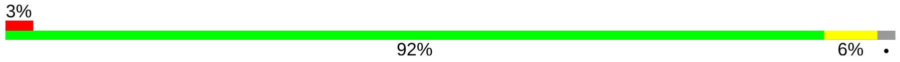

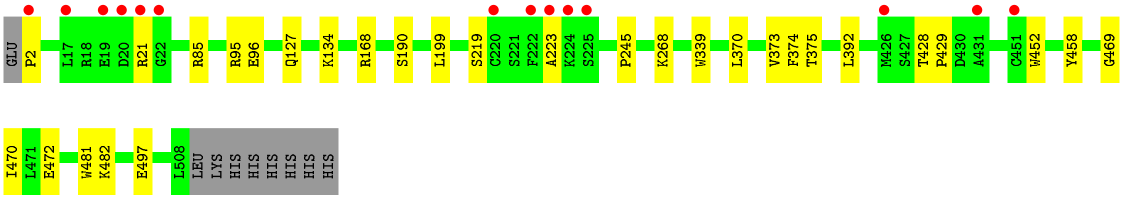

- Molecule 2: 2-acetamido-2-deoxy-beta-D-glucopyranose-(1-4)-2-acetamido-2-deoxy-beta-D-glucopyranose

Chain B:

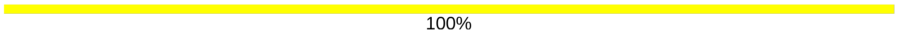

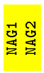

4 Data and refinement statistics (i)

Property	Value	Source
Space group	H 3	Depositor
Cell constants<br>a, b, c, α, β, γ	126.48Å 126.48Å 106.80Å<br>90.00° 90.00° 120.00°	Depositor
Resolution (Å)	63.24 – 1.42<br>63.24 – 1.42	Depositor<br>EDS
% Data completeness<br>(in resolution range)	92.6 (63.24-1.42)<br>92.6 (63.24-1.42)	Depositor<br>EDS
R_{merge}	0.05	Depositor
R_{sym}	(Not available)	Depositor
$< I/\sigma(I) >$ [1]	2.11 (at 1.42Å)	Xtriage
Refinement program	PHENIX (1.11.1_2575: ???)	Depositor
R, R_{free}	0.132 , 0.158<br>0.132 , 0.158	Depositor<br>DCC
R_{free} test set	5746 reflections (5.14%)	wwPDB-VP
Wilson B-factor (Å^2)	14.5	Xtriage
Anisotropy	0.135	Xtriage
Bulk solvent k_{sol}(e/Å^3), B_{sol}(Å^2)	0.38 , 47.0	EDS
L-test for twinning[2]	$< \|L\| > = 0.49$, $< L^2 > = 0.33$	Xtriage
Estimated twinning fraction	0.018 for h,-h-k,-l	Xtriage
F_o,F_c correlation	0.98	EDS
Total number of atoms	5093	wwPDB-VP
Average B, all atoms (Å^2)	23.0	wwPDB-VP

Xtriage's analysis on translational NCS is as follows: *The largest off-origin peak in the Patterson function is 3.87% of the height of the origin peak. No significant pseudotranslation is detected.*

[1] Intensities estimated from amplitudes.

[2] Theoretical values of $< |L| >$, $< L^2 >$ for acentric reflections are 0.5, 0.333 respectively for untwinned datasets, and 0.375, 0.2 for perfectly twinned datasets.

5 Model quality (i)

5.1 Standard geometry (i)

Bond lengths and bond angles in the following residue types are not validated in this section: PGE, NAG, PO4, EDO, 1PE, FE, PEG

The Z score for a bond length (or angle) is the number of standard deviations the observed value is removed from the expected value. A bond length (or angle) with $|Z| > 5$ is considered an outlier worth inspection. RMSZ is the root-mean-square of all Z scores of the bond lengths (or angles).

Mol	Chain	Bond lengths		Bond angles	
		RMSZ	#$|Z|$ >5	RMSZ	#$|Z|$ >5
1	A	0.32	0/4282	0.54	0/5836

There are no bond length outliers.

There are no bond angle outliers.

There are no chirality outliers.

There are no planarity outliers.

5.2 Too-close contacts (i)

In the following table, the Non-H and H(model) columns list the number of non-hydrogen atoms and hydrogen atoms in the chain respectively. The H(added) column lists the number of hydrogen atoms added and optimized by MolProbity. The Clashes column lists the number of clashes within the asymmetric unit, whereas Symm-Clashes lists symmetry related clashes.

Mol	Chain	Non-H	H(model)	H(added)	Clashes	Symm-Clashes
1	A	4078	0	3879	32	0
2	B	28	27	25	0	0
3	A	2	0	0	1	0
4	A	84	84	78	0	0
5	A	10	0	0	1	0
6	A	35	50	50	3	0
7	A	20	28	28	1	0
8	A	16	22	22	0	0
9	A	48	72	72	11	0
10	A	489	0	0	5	0
All	All	4810	283	4154	33	0

The all-atom clashscore is defined as the number of clashes found per 1000 atoms (including hydrogen atoms). The all-atom clashscore for this structure is 4.

All (33) close contacts within the same asymmetric unit are listed below, sorted by their clash magnitude.

Atom-1	Atom-2	Interatomic distance (Å)	Clash overlap (Å)
1:A:190[B]:SER:OG	10:A:701:HOH:O	2.09	0.70
1:A:134:LYS:NZ	7:A:619:PGE:O4	2.15	0.70
1:A:219:SER:HA	1:A:223:ALA:HB2	1.75	0.67
1:A:2:PRO:HG3	1:A:497[A]:GLU:OE2	1.96	0.65
3:A:601:FE:FE	5:A:611:PO4:O1	1.49	0.65
1:A:85:ARG:NH1	10:A:702:HOH:O	2.21	0.65
1:A:470:ILE:HG13	9:A:623:EDO:H12	1.78	0.64
1:A:21:ARG:HE	9:A:624:EDO:C2	2.10	0.64
1:A:21:ARG:HE	9:A:624:EDO:H22	1.61	0.64
1:A:469:GLY:HA3	1:A:481:TRP:CH2	2.40	0.57
1:A:95:ARG:HE	9:A:621:EDO:H22	1.69	0.56
1:A:245:PRO:HB2	6:A:614:PEG:H12	1.88	0.56
1:A:2:PRO:HB3	1:A:497[A]:GLU:HG3	1.88	0.55
1:A:127[A]:GLN:NE2	10:A:714:HOH:O	2.39	0.55
1:A:472:GLU:OE1	9:A:623:EDO:O2	2.26	0.53
1:A:168[A]:ARG:HH12	9:A:627:EDO:C1	2.21	0.53
1:A:96:GLU:O	9:A:631:EDO:H22	2.08	0.53
1:A:127[A]:GLN:HG2	10:A:709:HOH:O	2.09	0.52
1:A:482:LYS:HE3	9:A:623:EDO:O1	2.09	0.52
1:A:168[A]:ARG:HH12	9:A:627:EDO:H12	1.75	0.51
1:A:452:TRP:HA	6:A:617:PEG:H21	1.92	0.50
1:A:199:LEU:HD11	1:A:374:PHE:HB3	1.94	0.50
1:A:452:TRP:HA	6:A:617:PEG:H41	1.93	0.49
1:A:428:THR:N	1:A:429:PRO:CD	2.77	0.48
1:A:428:THR:N	1:A:429:PRO:HD2	2.29	0.48
1:A:370:LEU:HD21	1:A:373:VAL:HG22	1.99	0.45
1:A:268[B]:LYS:HE2	1:A:268[B]:LYS:HB3	1.75	0.43
1:A:482:LYS:HG2	1:A:497[B]:GLU:HG3	2.00	0.43
1:A:21:ARG:HE	9:A:624:EDO:H21	1.82	0.42
1:A:339:TRP:O	1:A:375:THR:HA	2.20	0.42
1:A:392:LEU:HD21	1:A:458:TYR:HA	2.02	0.42
1:A:127[A]:GLN:NE2	10:A:725:HOH:O	2.50	0.41
1:A:96:GLU:O	9:A:631:EDO:C2	2.69	0.41

There are no symmetry-related clashes.

5.3 Torsion angles ⓘ

5.3.1 Protein backbone ⓘ

In the following table, the Percentiles column shows the percent Ramachandran outliers of the chain as a percentile score with respect to all X-ray entries followed by that with respect to entries of similar resolution.

The Analysed column shows the number of residues for which the backbone conformation was analysed, and the total number of residues.

Mol	Chain	Analysed	Favoured	Allowed	Outliers	Percentiles	
1	A	531/516 (103%)	516 (97%)	15 (3%)	0	100	100

There are no Ramachandran outliers to report.

5.3.2 Protein sidechains ⓘ

In the following table, the Percentiles column shows the percent sidechain outliers of the chain as a percentile score with respect to all X-ray entries followed by that with respect to entries of similar resolution.

The Analysed column shows the number of residues for which the sidechain conformation was analysed, and the total number of residues.

Mol	Chain	Analysed	Rotameric	Outliers	Percentiles	
1	A	439/425 (103%)	439 (100%)	0	100	100

There are no protein residues with a non-rotameric sidechain to report.

Some sidechains can be flipped to improve hydrogen bonding and reduce clashes. There are no such sidechains identified.

5.3.3 RNA ⓘ

There are no RNA molecules in this entry.

5.4 Non-standard residues in protein, DNA, RNA chains ⓘ

There are no non-standard protein/DNA/RNA residues in this entry.

5.5 Carbohydrates (i)

2 monosaccharides are modelled in this entry.

In the following table, the Counts columns list the number of bonds (or angles) for which Mogul statistics could be retrieved, the number of bonds (or angles) that are observed in the model and the number of bonds (or angles) that are defined in the Chemical Component Dictionary. The Link column lists molecule types, if any, to which the group is linked. The Z score for a bond length (or angle) is the number of standard deviations the observed value is removed from the expected value. A bond length (or angle) with $|Z| > 2$ is considered an outlier worth inspection. RMSZ is the root-mean-square of all Z scores of the bond lengths (or angles).

Mol	Type	Chain	Res	Link	Bond lengths			Bond angles		
					Counts	RMSZ	$\#\|Z\| > 2$	Counts	RMSZ	$\#\|Z\| > 2$
2	NAG	B	1	1,2	14,14,15	1.89	3 (21%)	17,19,21	1.15	1 (5%)
2	NAG	B	2	2	14,14,15	1.98	4 (28%)	17,19,21	1.23	2 (11%)

In the following table, the Chirals column lists the number of chiral outliers, the number of chiral centers analysed, the number of these observed in the model and the number defined in the Chemical Component Dictionary. Similar counts are reported in the Torsion and Rings columns. '-' means no outliers of that kind were identified.

Mol	Type	Chain	Res	Link	Chirals	Torsions	Rings
2	NAG	B	1	1,2	-	2/6/23/26	0/1/1/1
2	NAG	B	2	2	-	0/6/23/26	0/1/1/1

All (7) bond length outliers are listed below:

Mol	Chain	Res	Type	Atoms	Z	Observed(Å)	Ideal(Å)
2	B	2	NAG	O5-C1	4.47	1.50	1.43
2	B	1	NAG	O5-C1	4.28	1.50	1.43
2	B	2	NAG	C7-N2	3.68	1.47	1.34
2	B	1	NAG	C7-N2	3.60	1.46	1.34
2	B	2	NAG	C2-N2	2.42	1.50	1.46
2	B	1	NAG	C2-N2	2.41	1.50	1.46
2	B	2	NAG	O5-C5	2.09	1.47	1.43

All (3) bond angle outliers are listed below:

Mol	Chain	Res	Type	Atoms	Z	Observed(^{o})	Ideal(^{o})
2	B	2	NAG	C8-C7-N2	2.85	120.93	116.10
2	B	1	NAG	C8-C7-N2	2.51	120.35	116.10
2	B	2	NAG	C2-N2-C7	-2.39	119.50	122.90

There are no chirality outliers.

All (2) torsion outliers are listed below:

Mol	Chain	Res	Type	Atoms
2	B	1	NAG	C8-C7-N2-C2
2	B	1	NAG	O7-C7-N2-C2

There are no ring outliers.

No monomer is involved in short contacts.

The following is a two-dimensional graphical depiction of Mogul quality analysis of bond lengths, bond angles, torsion angles, and ring geometry for oligosaccharide.

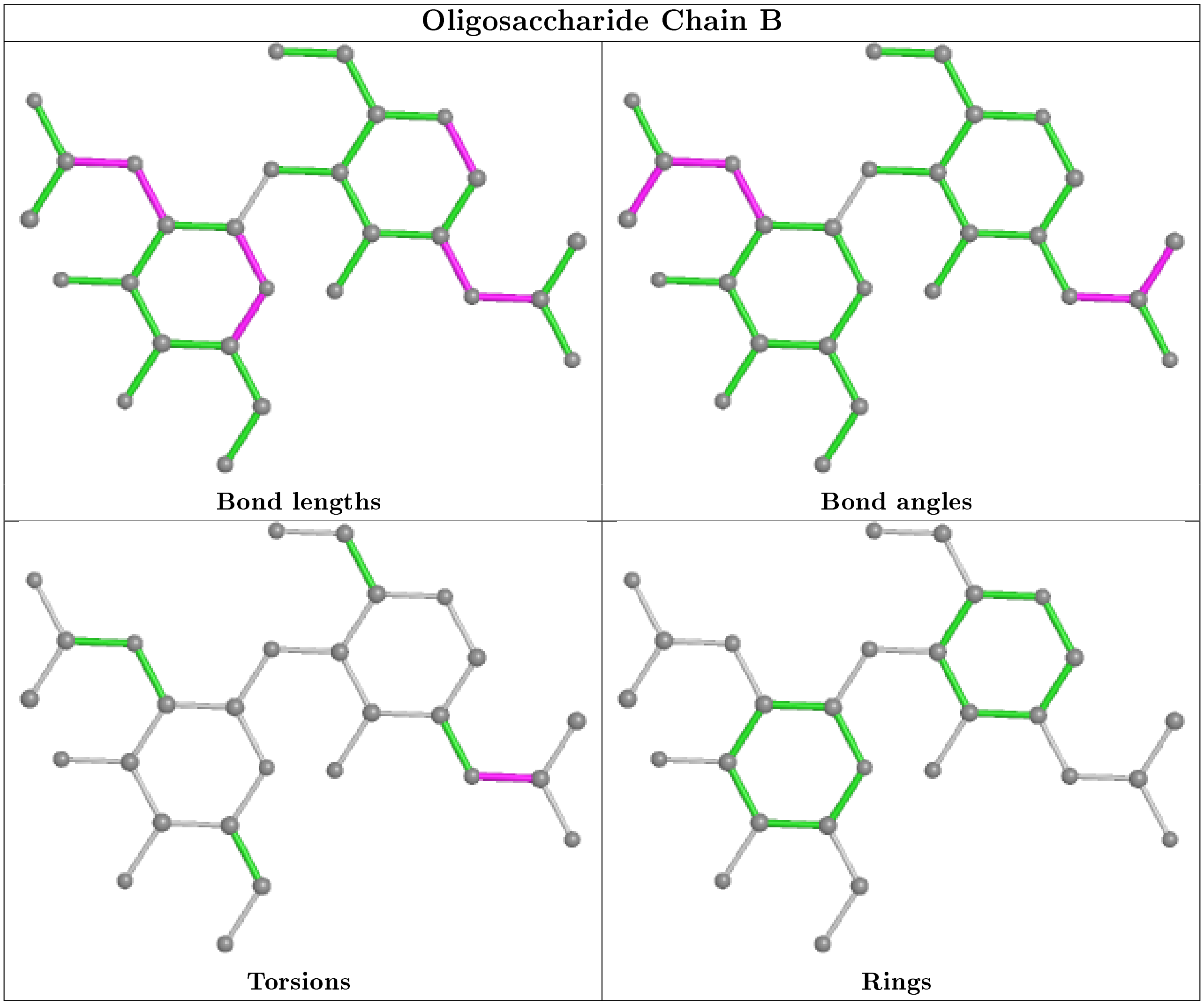

5.6 Ligand geometry (i)

Of 30 ligands modelled in this entry, 2 are monoatomic - leaving 28 for Mogul analysis.

In the following table, the Counts columns list the number of bonds (or angles) for which Mogul statistics could be retrieved, the number of bonds (or angles) that are observed in the model and the number of bonds (or angles) that are defined in the Chemical Component Dictionary. The Link column lists molecule types, if any, to which the group is linked. The Z score for a bond length (or angle) is the number of standard deviations the observed value is removed from the expected value. A bond length (or angle) with $|Z| > 2$ is considered an outlier worth inspection. RMSZ is the root-mean-square of all Z scores of the bond lengths (or angles).

Mol	Type	Chain	Res	Link	Bond lengths			Bond angles		
					Counts	RMSZ	$\#\|Z\| > 2$	Counts	RMSZ	$\#\|Z\| > 2$
9	EDO	A	632	-	3,3,3	0.42	0	2,2,2	0.15	0
7	PGE	A	618	-	9,9,9	0.51	0	8,8,8	0.34	0
5	PO4	A	611	3	4,4,4	0.87	0	6,6,6	0.44	0
8	1PE	A	620	-	15,15,15	0.53	0	14,14,14	0.40	0
9	EDO	A	628	-	3,3,3	0.47	0	2,2,2	0.26	0
5	PO4	A	612	-	4,4,4	0.93	0	6,6,6	0.50	0
6	PEG	A	614	-	6,6,6	0.47	0	5,5,5	0.88	0
6	PEG	A	616	-	6,6,6	0.47	0	5,5,5	0.32	0
6	PEG	A	617	-	6,6,6	0.50	0	5,5,5	0.56	0
4	NAG	A	604	1	14,14,15	1.56	2 (14%)	17,19,21	0.97	1 (5%)
4	NAG	A	605	1	14,14,15	1.99	4 (28%)	17,19,21	1.25	2 (11%)
9	EDO	A	631	-	3,3,3	0.45	0	2,2,2	0.22	0
9	EDO	A	630	-	3,3,3	0.47	0	2,2,2	0.29	0
4	NAG	A	603	1	14,14,15	1.76	3 (21%)	17,19,21	1.02	2 (11%)
4	NAG	A	607	1	14,14,15	1.92	3 (21%)	17,19,21	1.05	2 (11%)
7	PGE	A	619	-	9,9,9	0.51	0	8,8,8	0.24	0
9	EDO	A	621	-	3,3,3	0.47	0	2,2,2	0.39	0
9	EDO	A	625	-	3,3,3	0.48	0	2,2,2	0.28	0
4	NAG	A	608	1	14,14,15	1.79	3 (21%)	17,19,21	1.10	2 (11%)
9	EDO	A	624	-	3,3,3	0.45	0	2,2,2	0.27	0
9	EDO	A	629	-	3,3,3	0.47	0	2,2,2	0.33	0
9	EDO	A	626	-	3,3,3	0.45	0	2,2,2	0.26	0
9	EDO	A	627	-	3,3,3	0.47	0	2,2,2	0.31	0
9	EDO	A	622	-	3,3,3	0.47	0	2,2,2	0.34	0
9	EDO	A	623	-	3,3,3	0.49	0	2,2,2	0.36	0
4	NAG	A	606	1	14,14,15	1.98	5 (35%)	17,19,21	1.67	3 (17%)
6	PEG	A	615	-	6,6,6	0.49	0	5,5,5	0.21	0
6	PEG	A	613	-	6,6,6	0.48	0	5,5,5	0.28	0

In the following table, the Chirals column lists the number of chiral outliers, the number of chiral centers analysed, the number of these observed in the model and the number defined in the Chemical Component Dictionary. Similar counts are reported in the Torsion and Rings columns. '-' means no outliers of that kind were identified.

Mol	Type	Chain	Res	Link	Chirals	Torsions	Rings
9	EDO	A	632	-	-	1/1/1/1	-
7	PGE	A	618	-	-	1/7/7/7	-
8	1PE	A	620	-	-	1/13/13/13	-
9	EDO	A	628	-	-	0/1/1/1	-
6	PEG	A	615	-	-	0/4/4/4	-
6	PEG	A	614	-	-	3/4/4/4	-
6	PEG	A	616	-	-	1/4/4/4	-
6	PEG	A	617	-	-	1/4/4/4	-
4	NAG	A	604	1	-	0/6/23/26	0/1/1/1
4	NAG	A	605	1	-	1/6/23/26	0/1/1/1
9	EDO	A	631	-	-	1/1/1/1	-
9	EDO	A	630	-	-	0/1/1/1	-
4	NAG	A	603	1	-	0/6/23/26	0/1/1/1
4	NAG	A	607	1	-	0/6/23/26	0/1/1/1
7	PGE	A	619	-	-	0/7/7/7	-
9	EDO	A	621	-	-	0/1/1/1	-
9	EDO	A	625	-	-	0/1/1/1	-
4	NAG	A	608	1	-	0/6/23/26	0/1/1/1
9	EDO	A	624	-	-	1/1/1/1	-
9	EDO	A	629	-	-	0/1/1/1	-
9	EDO	A	626	-	-	0/1/1/1	-
9	EDO	A	627	-	-	0/1/1/1	-
9	EDO	A	622	-	-	1/1/1/1	-
9	EDO	A	623	-	-	1/1/1/1	-
4	NAG	A	606	1	-	0/6/23/26	0/1/1/1
6	PEG	A	613	-	-	0/4/4/4	-

All (20) bond length outliers are listed below:

Mol	Chain	Res	Type	Atoms	Z	Observed(Å)	Ideal(Å)
4	A	605	NAG	O5-C1	4.67	1.51	1.43
4	A	606	NAG	O5-C1	4.39	1.50	1.43
4	A	607	NAG	O5-C1	4.37	1.50	1.43
4	A	603	NAG	O5-C1	3.89	1.49	1.43
4	A	608	NAG	O5-C1	3.86	1.49	1.43
4	A	604	NAG	O5-C1	3.56	1.49	1.43
4	A	606	NAG	C7-N2	3.51	1.46	1.34
4	A	603	NAG	C7-N2	3.47	1.46	1.34
4	A	607	NAG	C7-N2	3.47	1.46	1.34
4	A	605	NAG	C7-N2	3.46	1.46	1.34
4	A	608	NAG	C7-N2	3.45	1.46	1.34
4	A	604	NAG	C7-N2	3.00	1.44	1.34
4	A	606	NAG	O5-C5	2.35	1.48	1.43

Continued on next page...

Continued from previous page...

Mol	Chain	Res	Type	Atoms	Z	Observed(Å)	Ideal(Å)
4	A	605	NAG	O5-C5	2.26	1.48	1.43
4	A	607	NAG	C2-N2	2.24	1.50	1.46
4	A	605	NAG	C2-N2	2.17	1.50	1.46
4	A	606	NAG	C2-N2	2.14	1.50	1.46
4	A	603	NAG	C2-N2	2.11	1.49	1.46
4	A	608	NAG	C2-N2	2.10	1.49	1.46
4	A	606	NAG	C3-C2	-2.07	1.48	1.52

All (12) bond angle outliers are listed below:

Mol	Chain	Res	Type	Atoms	Z	Observed(^{o})	Ideal(^{o})
4	A	606	NAG	C1-C2-N2	-3.87	103.89	110.49
4	A	606	NAG	O5-C1-C2	3.48	116.79	111.29
4	A	608	NAG	C2-N2-C7	-2.88	118.81	122.90
4	A	605	NAG	C2-N2-C7	-2.55	119.28	122.90
4	A	605	NAG	C8-C7-N2	2.52	120.37	116.10
4	A	608	NAG	C8-C7-N2	2.43	120.21	116.10
4	A	606	NAG	C8-C7-N2	2.31	120.01	116.10
4	A	607	NAG	C8-C7-N2	2.20	119.83	116.10
4	A	607	NAG	C2-N2-C7	-2.19	119.79	122.90
4	A	603	NAG	C2-N2-C7	-2.19	119.79	122.90
4	A	603	NAG	C8-C7-N2	2.14	119.72	116.10
4	A	604	NAG	O5-C1-C2	-2.04	108.07	111.29

There are no chirality outliers.

All (13) torsion outliers are listed below:

Mol	Chain	Res	Type	Atoms
6	A	614	PEG	C4-C3-O2-C2
6	A	614	PEG	O1-C1-C2-O2
9	A	632	EDO	O1-C1-C2-O2
9	A	631	EDO	O1-C1-C2-O2
7	A	618	PGE	C1-C2-O2-C3
6	A	614	PEG	C1-C2-O2-C3
9	A	624	EDO	O1-C1-C2-O2
8	A	620	1PE	C23-C13-OH4-C24
4	A	605	NAG	O5-C5-C6-O6
6	A	617	PEG	C4-C3-O2-C2
9	A	622	EDO	O1-C1-C2-O2
6	A	616	PEG	C4-C3-O2-C2
9	A	623	EDO	O1-C1-C2-O2

There are no ring outliers.

9 monomers are involved in 16 short contacts:

Mol	Chain	Res	Type	Clashes	Symm-Clashes
5	A	611	PO4	1	0
6	A	614	PEG	1	0
6	A	617	PEG	2	0
9	A	631	EDO	2	0
7	A	619	PGE	1	0
9	A	621	EDO	1	0
9	A	624	EDO	3	0
9	A	627	EDO	2	0
9	A	623	EDO	3	0

5.7 Other polymers (i)

There are no such residues in this entry.

5.8 Polymer linkage issues (i)

There are no chain breaks in this entry.

6 Fit of model and data (i)

6.1 Protein, DNA and RNA chains (i)

In the following table, the column labelled '#RSRZ> 2' contains the number (and percentage) of RSRZ outliers, followed by percent RSRZ outliers for the chain as percentile scores relative to all X-ray entries and entries of similar resolution. The OWAB column contains the minimum, median, 95th percentile and maximum values of the occupancy-weighted average B-factor per residue. The column labelled 'Q< 0.9' lists the number of (and percentage) of residues with an average occupancy less than 0.9.

Mol	Chain	Analysed	<RSRZ>	#RSRZ>2			OWAB(Å^2)	Q<0.9
1	A	507/516 (98%)	-0.17	14 (2%)	53	52	11, 16, 33, 70	0

All (14) RSRZ outliers are listed below:

Mol	Chain	Res	Type	RSRZ
1	A	223	ALA	5.2
1	A	220	CYS	4.9
1	A	20	ASP	3.7
1	A	21	ARG	3.3
1	A	224	LYS	3.1
1	A	222	PHE	2.8
1	A	426	MET	2.6
1	A	22	GLY	2.6
1	A	2	PRO	2.5
1	A	451	CYS	2.4
1	A	225	SER	2.3
1	A	431	ALA	2.3
1	A	17	LEU	2.2
1	A	19	GLU	2.2

6.2 Non-standard residues in protein, DNA, RNA chains (i)

There are no non-standard protein/DNA/RNA residues in this entry.

6.3 Carbohydrates (i)

In the following table, the Atoms column lists the number of modelled atoms in the group and the number defined in the chemical component dictionary. The B-factors column lists the minimum, median, 95th percentile and maximum values of B factors of atoms in the group. The column labelled 'Q< 0.9' lists the number of atoms with occupancy less than 0.9.

Mol	Type	Chain	Res	Atoms	RSCC	RSR	B-factors($Å^2$)	Q<0.9
2	NAG	B	2	14/15	0.81	0.18	43,50,60,61	28
2	NAG	B	1	14/15	0.92	0.13	27,40,51,51	0

The following is a graphical depiction of the model fit to experimental electron density for oligosaccharide. Each fit is shown from different orientation to approximate a three-dimensional view.

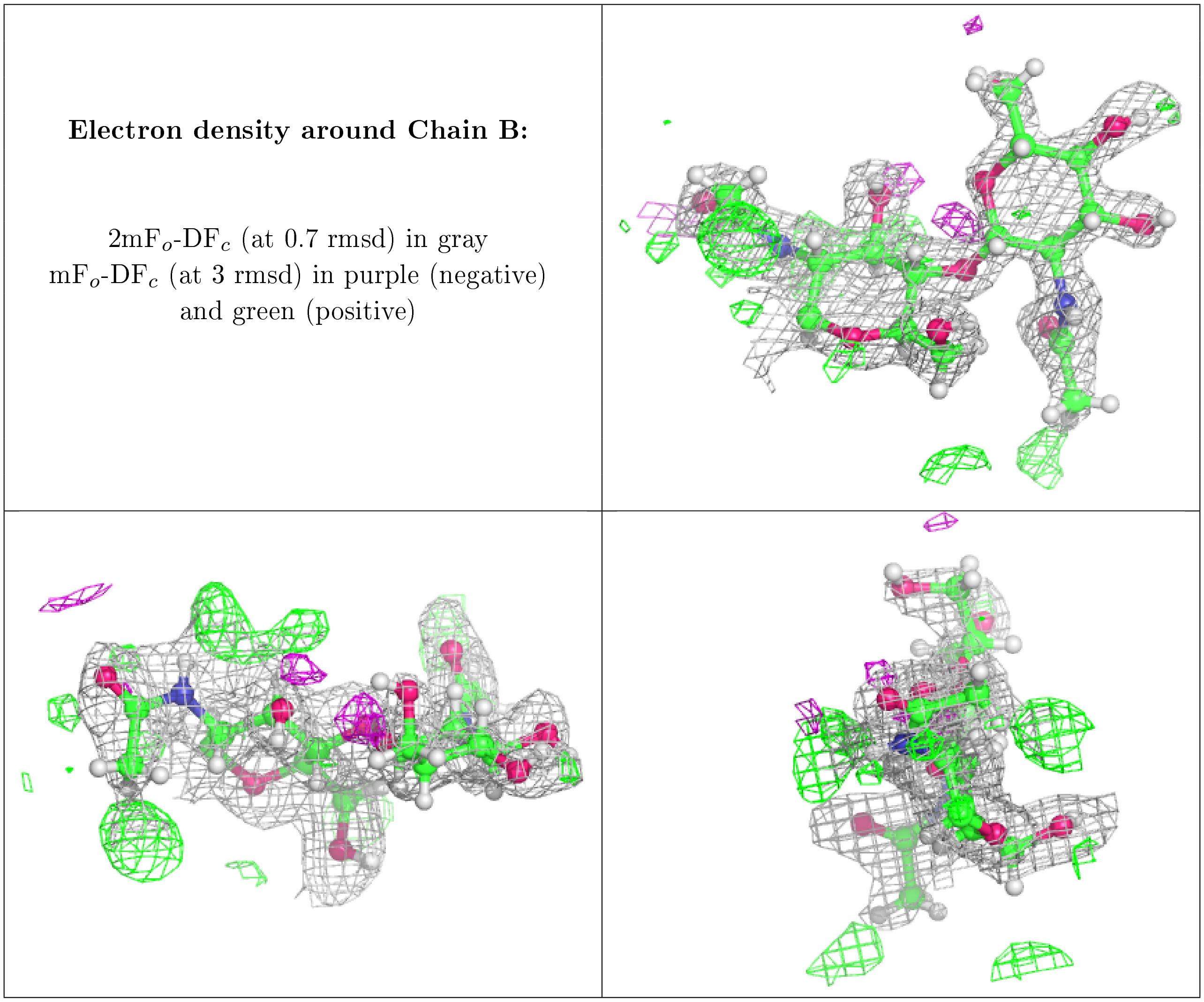

6.4 Ligands ⓘ

In the following table, the Atoms column lists the number of modelled atoms in the group and the number defined in the chemical component dictionary. The B-factors column lists the minimum, median, 95th percentile and maximum values of B factors of atoms in the group. The column labelled 'Q< 0.9' lists the number of atoms with occupancy less than 0.9.

Mol	Type	Chain	Res	Atoms	RSCC	RSR	B-factors($Å^2$)	Q<0.9
4	NAG	A	606	14/15	0.75	0.23	49,61,73,73	28

Continued on next page...

Continued from previous page...

Mol	Type	Chain	Res	Atoms	RSCC	RSR	B-factors(Å^2)	Q<0.9
9	EDO	A	624	4/4	0.76	0.19	47,57,59,60	0
6	PEG	A	617	7/7	0.77	0.28	47,58,69,70	0
9	EDO	A	627	4/4	0.78	0.14	56,67,67,68	0
6	PEG	A	613	7/7	0.78	0.16	53,64,67,68	0
9	EDO	A	625	4/4	0.80	0.14	60,72,74,75	0
4	NAG	A	607	14/15	0.82	0.20	35,44,53,55	28
7	PGE	A	619	10/10	0.83	0.15	55,67,73,74	0
6	PEG	A	615	7/7	0.83	0.12	56,67,70,70	0
6	PEG	A	614	7/7	0.83	0.16	45,54,63,63	0
9	EDO	A	628	4/4	0.84	0.15	57,68,69,71	0
9	EDO	A	626	4/4	0.84	0.14	44,53,56,58	0
9	EDO	A	630	4/4	0.85	0.12	52,62,63,63	0
6	PEG	A	616	7/7	0.85	0.13	58,69,70,70	0
9	EDO	A	632	4/4	0.86	0.20	30,37,42,44	0
9	EDO	A	629	4/4	0.87	0.23	47,57,59,60	0
9	EDO	A	621	4/4	0.89	0.09	40,48,49,50	0
9	EDO	A	623	4/4	0.91	0.15	40,48,48,51	0
4	NAG	A	605	14/15	0.91	0.14	36,45,56,58	0
7	PGE	A	618	10/10	0.92	0.11	40,48,58,60	0
8	1PE	A	620	16/16	0.92	0.16	33,43,63,65	0
9	EDO	A	631	4/4	0.94	0.20	31,37,41,41	0
4	NAG	A	603	14/15	0.95	0.12	20,28,34,36	0
4	NAG	A	608	14/15	0.95	0.17	26,33,47,47	0
5	PO4	A	612	5/5	0.95	0.21	56,57,58,59	0
9	EDO	A	622	4/4	0.95	0.06	51,62,63,64	0
3	FE	A	601	1/1	0.96	0.09	44,44,44,44	1
4	NAG	A	604	14/15	0.98	0.08	14,18,23,26	0
5	PO4	A	611	5/5	0.98	0.07	22,27,29,40	0
3	FE	A	602	1/1	1.00	0.06	12,12,12,12	1

6.5 Other polymers ⓘ

There are no such residues in this entry.

Full wwPDB X-ray Structure Validation Report (i)

Aug 7, 2020 – 12:14 PM BST

PDB ID	:	6GIZ
Title	:	PURPLE ACID PHYTASE FROM WHEAT ISOFORM B2 - SUBSTRATE COMPLEX
Authors	:	Faba-Rodriguez, R.; Brearley, C.A.; Hemmings, A.M.
Deposited on	:	2018-05-15
Resolution	:	1.54 Å(reported)

This is a Full wwPDB X-ray Structure Validation Report for a publicly released PDB entry.

We welcome your comments at *validation@mail.wwpdb.org*
A user guide is available at
https://www.wwpdb.org/validation/2017/XrayValidationReportHelp
with specific help available everywhere you see the (i) symbol.

The following versions of software and data (see references (i)) were used in the production of this report:

MolProbity	:	4.02b-467
Mogul	:	1.8.5 (274361), CSD as541be (2020)
Xtriage (Phenix)	:	1.13
EDS	:	2.13.1
Percentile statistics	:	20191225.v01 (using entries in the PDB archive December 25th 2019)
Refmac	:	5.8.0158
CCP4	:	7.0.044 (Gargrove)
Ideal geometry (proteins)	:	Engh & Huber (2001)
Ideal geometry (DNA, RNA)	:	Parkinson et al. (1996)
Validation Pipeline (wwPDB-VP)	:	2.13.1

1 Overall quality at a glance (i)

The following experimental techniques were used to determine the structure:
X-RAY DIFFRACTION

The reported resolution of this entry is 1.54 Å.

Percentile scores (ranging between 0-100) for global validation metrics of the entry are shown in the following graphic. The table shows the number of entries on which the scores are based.

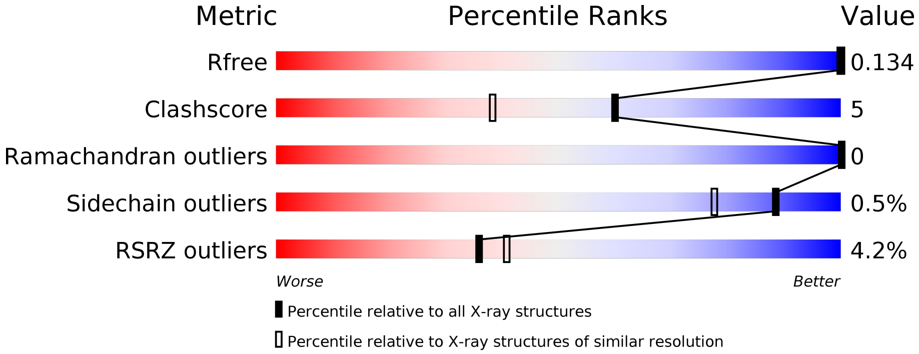

Metric	Whole archive (#Entries)	Similar resolution (#Entries, resolution range(Å))
R_{free}	130704	2556 (1.56-1.52)
Clashscore	141614	2634 (1.56-1.52)
Ramachandran outliers	138981	2580 (1.56-1.52)
Sidechain outliers	138945	2577 (1.56-1.52)
RSRZ outliers	127900	2524 (1.56-1.52)

The table below summarises the geometric issues observed across the polymeric chains and their fit to the electron density. The red, orange, yellow and green segments on the lower bar indicate the fraction of residues that contain outliers for >=3, 2, 1 and 0 types of geometric quality criteria respectively. A grey segment represents the fraction of residues that are not modelled. The numeric value for each fraction is indicated below the corresponding segment, with a dot representing fractions <=5% The upper red bar (where present) indicates the fraction of residues that have poor fit to the electron density. The numeric value is given above the bar.

Mol	Chain	Length	Quality of chain
1	A	516	4% / 91% 7% •
2	B	2	100%
2	C	2	100%

The following table lists non-polymeric compounds, carbohydrate monomers and non-standard residues in protein, DNA, RNA chains that are outliers for geometric or electron-density-fit criteria:

Mol	Type	Chain	Res	Chirality	Geometry	Clashes	Electron density
8	EDO	A	624	-	-	X	-

2 Entry composition (i)

There are 9 unique types of molecules in this entry. The entry contains 4915 atoms, of which 254 are hydrogens and 0 are deuteriums.

In the tables below, the ZeroOcc column contains the number of atoms modelled with zero occupancy, the AltConf column contains the number of residues with at least one atom in alternate conformation and the Trace column contains the number of residues modelled with at most 2 atoms.

- Molecule 1 is a protein called Purple acid phosphatase.

Mol	Chain	Residues	Atoms					ZeroOcc	AltConf	Trace
			Total	C	N	O	S			
1	A	504	3995	2541	668	762	24	5	14	0

There are 6 discrepancies between the modelled and reference sequences:

Chain	Residue	Modelled	Actual	Comment	Reference
A	511	HIS	-	expression tag	UNP C4PKL0
A	512	HIS	-	expression tag	UNP C4PKL0
A	513	HIS	-	expression tag	UNP C4PKL0
A	514	HIS	-	expression tag	UNP C4PKL0
A	515	HIS	-	expression tag	UNP C4PKL0
A	516	HIS	-	expression tag	UNP C4PKL0

- Molecule 2 is an oligosaccharide called 2-acetamido-2-deoxy-beta-D-glucopyranose-(1-4)-2-acetamido-2-deoxy-beta-D-glucopyranose.

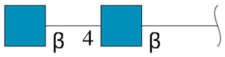

Mol	Chain	Residues	Atoms					ZeroOcc	AltConf	Trace
			Total	C	H	N	O			
2	B	2	55	16	27	2	10	0	0	0
2	C	2	55	16	27	2	10	0	0	0

- Molecule 3 is FE (III) ION (three-letter code: FE) (formula: Fe).

Mol	Chain	Residues	Atoms		ZeroOcc	AltConf
			Total	Fe		
3	A	2	2	2	0	0

- Molecule 4 is 2-acetamido-2-deoxy-beta-D-glucopyranose (three-letter code: NAG) (formula: $C_8H_{15}NO_6$).

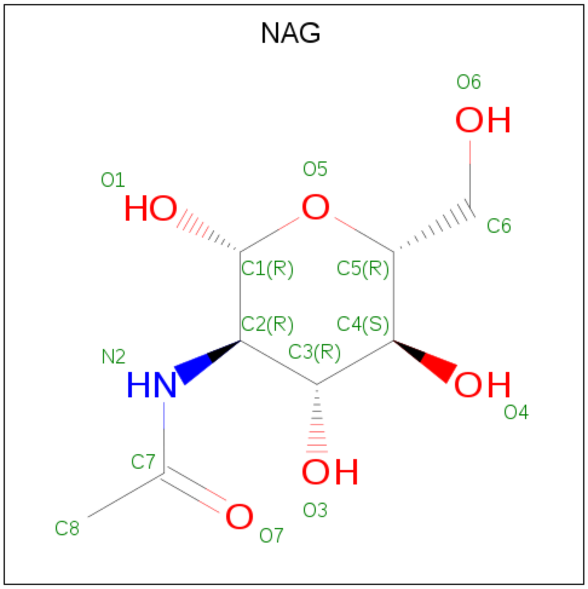

Mol	Chain	Residues	Atoms					ZeroOcc	AltConf
4	A	1	Total 28	C 8	H 14	N 1	O 5	0	0
4	A	1	Total 28	C 8	H 14	N 1	O 5	0	0
4	A	1	Total 28	C 8	H 14	N 1	O 5	0	0
4	A	1	Total 28	C 8	H 14	N 1	O 5	0	0
4	A	1	Total 28	C 8	H 14	N 1	O 5	0	0

- Molecule 5 is PHOSPHATE ION (three-letter code: PO4) (formula: O_4P).

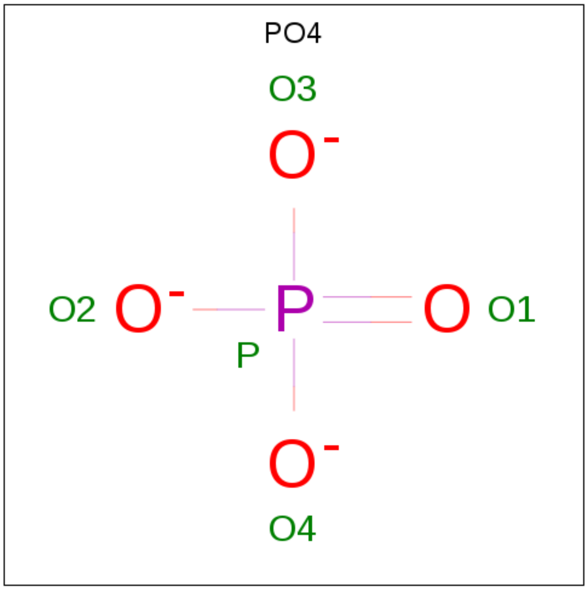

Mol	Chain	Residues	Atoms	ZeroOcc	AltConf
5	A	1	Total O P<br>5 4 1	0	0
5	A	1	Total O P<br>5 4 1	0	0
5	A	1	Total O P<br>5 4 1	0	0

- Molecule 6 is TRIETHYLENE GLYCOL (three-letter code: PGE) (formula: $C_6H_{14}O_4$).

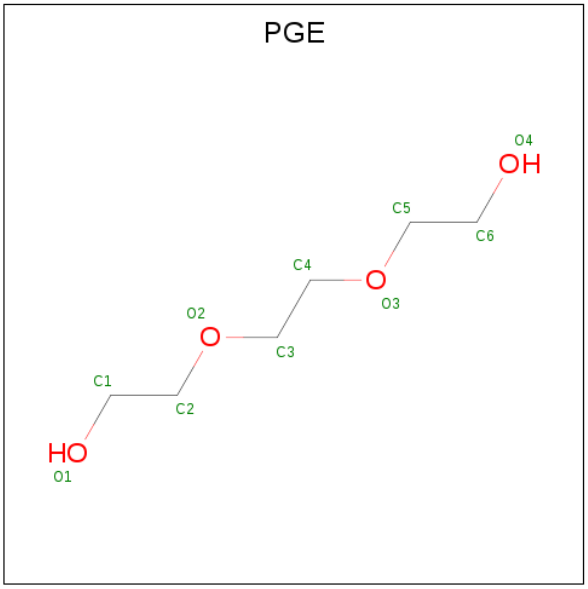

Mol	Chain	Residues	Atoms	ZeroOcc	AltConf
6	A	1	Total C H O<br>24 6 14 4	0	0

Continued on next page...

Continued from previous page...

Mol	Chain	Residues	Atoms				ZeroOcc	AltConf
6	A	1	Total 24	C 6	H 14	O 4	0	0
6	A	1	Total 24	C 6	H 14	O 4	0	0

- Molecule 7 is DI(HYDROXYETHYL)ETHER (three-letter code: PEG) (formula: $C_4H_{10}O_3$).

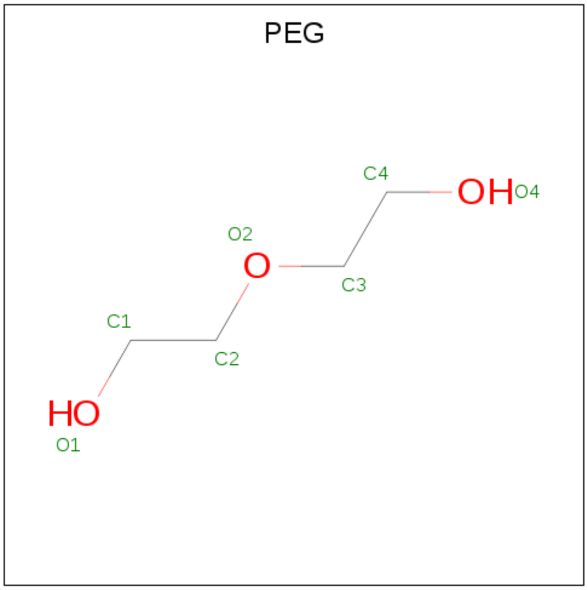

Mol	Chain	Residues	Atoms				ZeroOcc	AltConf
7	A	1	Total 17	C 4	H 10	O 3	0	0
7	A	1	Total 17	C 4	H 10	O 3	0	0
7	A	1	Total 17	C 4	H 10	O 3	0	0
7	A	1	Total 17	C 4	H 10	O 3	0	0

- Molecule 8 is 1,2-ETHANEDIOL (three-letter code: EDO) (formula: $C_2H_6O_2$).

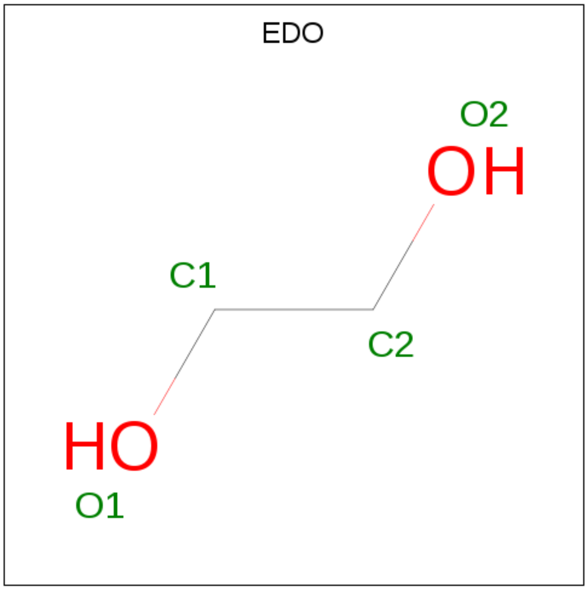

Mol	Chain	Residues	Atoms	ZeroOcc	AltConf
8	A	1	Total C H O<br>10 2 6 2	0	0
8	A	1	Total C H O<br>10 2 6 2	0	0
8	A	1	Total C H O<br>10 2 6 2	0	0
8	A	1	Total C H O<br>10 2 6 2	0	0
8	A	1	Total C H O<br>10 2 6 2	0	0
8	A	1	Total C H O<br>10 2 6 2	0	0
8	A	1	Total C H O<br>10 2 6 2	0	0
8	A	1	Total C H O<br>10 2 6 2	0	0

- Molecule 9 is water.

Mol	Chain	Residues	Atoms	ZeroOcc	AltConf
9	A	433	Total O<br>433 433	0	0

3 Residue-property plots (i)

These plots are drawn for all protein, RNA, DNA and oligosaccharide chains in the entry. The first graphic for a chain summarises the proportions of the various outlier classes displayed in the second graphic. The second graphic shows the sequence view annotated by issues in geometry and electron density. Residues are color-coded according to the number of geometric quality criteria for which they contain at least one outlier: green = 0, yellow = 1, orange = 2 and red = 3 or more. A red dot above a residue indicates a poor fit to the electron density (RSRZ > 2). Stretches of 2 or more consecutive residues without any outlier are shown as a green connector. Residues present in the sample, but not in the model, are shown in grey.

• Molecule 1: Purple acid phosphatase

Chain A:

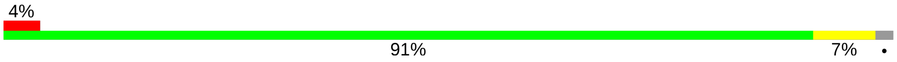

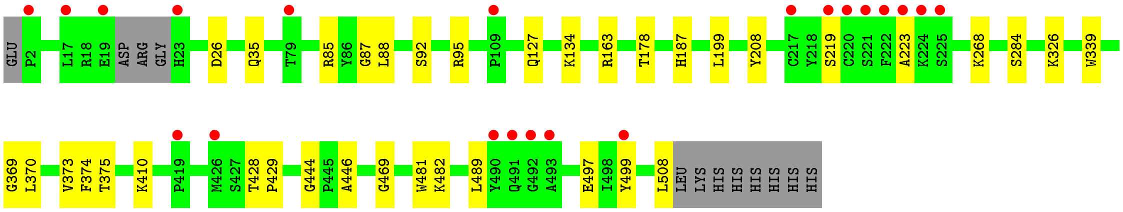

• Molecule 2: 2-acetamido-2-deoxy-beta-D-glucopyranose-(1-4)-2-acetamido-2-deoxy-beta-D-glucopyranose

Chain B:

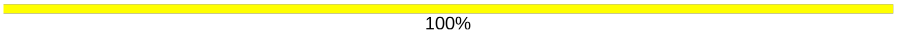

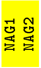

• Molecule 2: 2-acetamido-2-deoxy-beta-D-glucopyranose-(1-4)-2-acetamido-2-deoxy-beta-D-glucopyranose

Chain C:

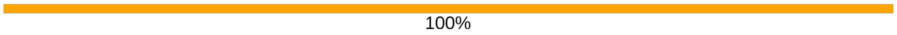

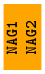

4 Data and refinement statistics (i)

Property	Value	Source
Space group	H 3	Depositor
Cell constants<br>a, b, c, α, β, γ	126.73Å 126.73Å 107.04Å<br>90.00° 90.00° 120.00°	Depositor
Resolution (Å)	48.10 – 1.54<br>48.11 – 1.54	Depositor<br>EDS
% Data completeness<br>(in resolution range)	99.8 (48.10-1.54)<br>99.8 (48.11-1.54)	Depositor<br>EDS
R_{merge}	0.06	Depositor
R_{sym}	(Not available)	Depositor
$< I/\sigma(I) >$ [1]	1.66 (at 1.54Å)	Xtriage
Refinement program	PHENIX	Depositor
R, R_{free}	0.136 , 0.167<br>0.138 , 0.134	Depositor<br>DCC
R_{free} test set	4709 reflections (4.97%)	wwPDB-VP
Wilson B-factor (Å^2)	18.8	Xtriage
Anisotropy	0.131	Xtriage
Bulk solvent k_{sol}(e/Å^3), B_{sol}(Å^2)	0.38 , 53.1	EDS
L-test for twinning[2]	$< \|L\| >$ = 0.48, $< L^2 >$ = 0.31	Xtriage
Estimated twinning fraction	0.024 for h,-h-k,-l	Xtriage
F_o,F_c correlation	0.98	EDS
Total number of atoms	4915	wwPDB-VP
Average B, all atoms (Å^2)	28.0	wwPDB-VP

Xtriage's analysis on translational NCS is as follows: *The largest off-origin peak in the Patterson function is 3.82% of the height of the origin peak. No significant pseudotranslation is detected.*

[1]Intensities estimated from amplitudes.

[2]Theoretical values of $< |L| >$, $< L^2 >$ for acentric reflections are 0.5, 0.333 respectively for untwinned datasets, and 0.375, 0.2 for perfectly twinned datasets.

5 Model quality (i)

5.1 Standard geometry (i)

Bond lengths and bond angles in the following residue types are not validated in this section: PGE, NAG, PO4, EDO, FE, PEG

The Z score for a bond length (or angle) is the number of standard deviations the observed value is removed from the expected value. A bond length (or angle) with $|Z| > 5$ is considered an outlier worth inspection. RMSZ is the root-mean-square of all Z scores of the bond lengths (or angles).

Mol	Chain	Bond lengths		Bond angles	
		RMSZ	#|Z| >5	RMSZ	#|Z| >5
1	A	0.34	0/4161	0.52	0/5678

There are no bond length outliers.

There are no bond angle outliers.

There are no chirality outliers.

There are no planarity outliers.

5.2 Too-close contacts (i)

In the following table, the Non-H and H(model) columns list the number of non-hydrogen atoms and hydrogen atoms in the chain respectively. The H(added) column lists the number of hydrogen atoms added and optimized by MolProbity. The Clashes column lists the number of clashes within the asymmetric unit, whereas Symm-Clashes lists symmetry related clashes.

Mol	Chain	Non-H	H(model)	H(added)	Clashes	Symm-Clashes
1	A	3995	0	3749	34	0
2	B	28	27	25	0	0
2	C	28	27	25	2	0
3	A	2	0	0	0	0
4	A	70	70	65	1	0
5	A	15	0	0	2	0
6	A	30	42	42	4	0
7	A	28	40	40	3	0
8	A	32	48	47	8	0
9	A	433	0	0	7	0
All	All	4661	254	3993	38	0

The all-atom clashscore is defined as the number of clashes found per 1000 atoms (including hydrogen atoms). The all-atom clashscore for this structure is 5.

All (38) close contacts within the same asymmetric unit are listed below, sorted by their clash magnitude.

Atom-1	Atom-2	Interatomic distance (Å)	Clash overlap (Å)
1:A:219:SER:HA	1:A:223:ALA:HB2	1.56	0.87
1:A:88:LEU:HG	8:A:624:EDO:H11	1.61	0.82
1:A:444:GLY:HA3	6:A:617:PGE:H12	1.73	0.71
1:A:134:LYS:NZ	7:A:620:PEG:O1	2.16	0.71
1:A:127[B]:GLN:NE2	9:A:707:HOH:O	2.26	0.69
1:A:482:LYS:HG2	1:A:497:GLU:HG3	1.75	0.68
1:A:26[B]:ASP:OD2	1:A:208:TYR:OH	2.11	0.67
1:A:95:ARG:HD2	8:A:624:EDO:O1	1.97	0.63
1:A:26[A]:ASP:OD2	9:A:702:HOH:O	2.15	0.62
1:A:369:GLY:HA3	1:A:508:LEU:HD21	1.83	0.60
1:A:127[B]:GLN:HG2	9:A:704:HOH:O	2.02	0.59
1:A:326:LYS:HZ2	6:A:616:PGE:H3	1.67	0.59
1:A:469:GLY:HA3	1:A:481:TRP:CH2	2.37	0.58
1:A:87:GLY:HA2	8:A:624:EDO:H12	1.86	0.56
5:A:612:PO4:P	9:A:709:HOH:O	2.63	0.56
1:A:163:ARG:NH1	8:A:627:EDO:O2	2.36	0.51
1:A:428:THR:N	1:A:429:PRO:HD2	2.27	0.49
1:A:88:LEU:CG	8:A:624:EDO:H11	2.39	0.49
1:A:410:LYS:HE3	5:A:614:PO4:O1	2.13	0.48
1:A:428:THR:N	1:A:429:PRO:CD	2.79	0.46
2:C:1:NAG:O3	2:C:2:NAG:O5	2.31	0.46
1:A:26[B]:ASP:OD1	1:A:178:THR:HG22	2.16	0.46
1:A:199:LEU:HD11	1:A:374:PHE:HB3	1.99	0.45
1:A:187:HIS:NE2	1:A:489:LEU:HD23	2.31	0.45
1:A:85:ARG:NH1	9:A:701:HOH:O	2.09	0.44
1:A:284:SER:O	6:A:616:PGE:H4	2.16	0.44
1:A:446:ALA:HA	8:A:625:EDO:H21	2.00	0.44
1:A:163:ARG:H	8:A:627:EDO:H12	1.84	0.43
1:A:92:SER:HB3	9:A:711:HOH:O	2.18	0.43
4:A:611:NAG:C8	4:A:611:NAG:H3	2.48	0.43
1:A:339:TRP:O	1:A:375:THR:HA	2.18	0.42
1:A:370:LEU:HD21	1:A:373:VAL:CG2	2.50	0.42
1:A:268:LYS:HE2	9:A:1017:HOH:O	2.20	0.41
1:A:326:LYS:HZ2	6:A:616:PGE:H6	1.85	0.41
1:A:370:LEU:HD21	1:A:373:VAL:HG22	2.03	0.41
7:A:621:PEG:C1	2:C:2:NAG:O4	2.69	0.41
1:A:95:ARG:HE	7:A:618:PEG:H12	1.86	0.40
1:A:446:ALA:HA	8:A:625:EDO:C2	2.50	0.40

There are no symmetry-related clashes.

5.3 Torsion angles (i)

5.3.1 Protein backbone (i)

In the following table, the Percentiles column shows the percent Ramachandran outliers of the chain as a percentile score with respect to all X-ray entries followed by that with respect to entries of similar resolution.

The Analysed column shows the number of residues for which the backbone conformation was analysed, and the total number of residues.

Mol	Chain	Analysed	Favoured	Allowed	Outliers	Percentiles	
1	A	514/516 (100%)	498 (97%)	16 (3%)	0	100	100

There are no Ramachandran outliers to report.

5.3.2 Protein sidechains (i)

In the following table, the Percentiles column shows the percent sidechain outliers of the chain as a percentile score with respect to all X-ray entries followed by that with respect to entries of similar resolution.

The Analysed column shows the number of residues for which the sidechain conformation was analysed, and the total number of residues.

Mol	Chain	Analysed	Rotameric	Outliers	Percentiles	
1	A	424/425 (100%)	422 (100%)	2 (0%)	88	77

All (2) residues with a non-rotameric sidechain are listed below:

Mol	Chain	Res	Type
1	A	35	GLN
1	A	499	TYR

Some sidechains can be flipped to improve hydrogen bonding and reduce clashes. All (1) such sidechains are listed below:

Mol	Chain	Res	Type
1	A	191	ASN

5.3.3 RNA (i)

There are no RNA molecules in this entry.

5.4 Non-standard residues in protein, DNA, RNA chains (i)

There are no non-standard protein/DNA/RNA residues in this entry.

5.5 Carbohydrates (i)

4 monosaccharides are modelled in this entry.

In the following table, the Counts columns list the number of bonds (or angles) for which Mogul statistics could be retrieved, the number of bonds (or angles) that are observed in the model and the number of bonds (or angles) that are defined in the Chemical Component Dictionary. The Link column lists molecule types, if any, to which the group is linked. The Z score for a bond length (or angle) is the number of standard deviations the observed value is removed from the expected value. A bond length (or angle) with $|Z| > 2$ is considered an outlier worth inspection. RMSZ is the root-mean-square of all Z scores of the bond lengths (or angles).

Mol	Type	Chain	Res	Link	Bond lengths			Bond angles		
					Counts	RMSZ	#|Z| > 2	Counts	RMSZ	#|Z| > 2
2	NAG	B	1	1,2	14,14,15	1.66	2 (14%)	17,19,21	1.59	4 (23%)
2	NAG	B	2	2	14,14,15	1.94	4 (28%)	17,19,21	1.25	2 (11%)
2	NAG	C	1	1,2	14,14,15	1.62	2 (14%)	17,19,21	1.03	0
2	NAG	C	2	2	14,14,15	1.94	4 (28%)	17,19,21	1.32	2 (11%)

In the following table, the Chirals column lists the number of chiral outliers, the number of chiral centers analysed, the number of these observed in the model and the number defined in the Chemical Component Dictionary. Similar counts are reported in the Torsion and Rings columns. '-' means no outliers of that kind were identified.

Mol	Type	Chain	Res	Link	Chirals	Torsions	Rings
2	NAG	B	1	1,2	-	2/6/23/26	0/1/1/1
2	NAG	B	2	2	-	0/6/23/26	0/1/1/1
2	NAG	C	1	1,2	-	2/6/23/26	0/1/1/1
2	NAG	C	2	2	-	0/6/23/26	0/1/1/1

All (12) bond length outliers are listed below:

Mol	Chain	Res	Type	Atoms	Z	Observed(Å)	Ideal(Å)
2	C	2	NAG	O5-C1	4.48	1.50	1.43
2	B	2	NAG	O5-C1	4.39	1.50	1.43
2	B	2	NAG	C7-N2	3.51	1.46	1.34
2	C	2	NAG	C7-N2	3.47	1.46	1.34
2	B	1	NAG	O5-C1	3.42	1.49	1.43
2	C	1	NAG	O5-C1	3.34	1.49	1.43

Continued on next page...

Continued from previous page...

Mol	Chain	Res	Type	Atoms	Z	Observed(Å)	Ideal(Å)
2	B	1	NAG	C7-N2	3.28	1.45	1.34
2	C	1	NAG	C7-N2	3.12	1.45	1.34
2	C	2	NAG	C2-N2	2.33	1.50	1.46
2	B	2	NAG	C2-N2	2.30	1.50	1.46
2	B	2	NAG	O5-C5	2.11	1.47	1.43
2	C	2	NAG	O5-C5	2.03	1.47	1.43

All (8) bond angle outliers are listed below:

Mol	Chain	Res	Type	Atoms	Z	Observed(^{o})	Ideal(^{o})
2	B	2	NAG	C1-C2-N2	-3.59	104.35	110.49
2	B	1	NAG	C4-C3-C2	2.91	115.29	111.02
2	B	1	NAG	O4-C4-C5	-2.88	102.15	109.30
2	B	1	NAG	O5-C1-C2	-2.41	107.48	111.29
2	C	2	NAG	O5-C5-C6	2.36	110.90	107.20
2	B	2	NAG	C8-C7-N2	2.23	119.88	116.10
2	B	1	NAG	C2-N2-C7	-2.16	119.83	122.90
2	C	2	NAG	C8-C7-N2	2.03	119.53	116.10

There are no chirality outliers.

All (4) torsion outliers are listed below:

Mol	Chain	Res	Type	Atoms
2	B	1	NAG	C4-C5-C6-O6
2	C	1	NAG	C4-C5-C6-O6
2	C	1	NAG	O5-C5-C6-O6
2	B	1	NAG	O5-C5-C6-O6

There are no ring outliers.

2 monomers are involved in 2 short contacts:

Mol	Chain	Res	Type	Clashes	Symm-Clashes
2	C	1	NAG	1	0
2	C	2	NAG	2	0

The following is a two-dimensional graphical depiction of Mogul quality analysis of bond lengths, bond angles, torsion angles, and ring geometry for oligosaccharide.

Oligosaccharide Chain B

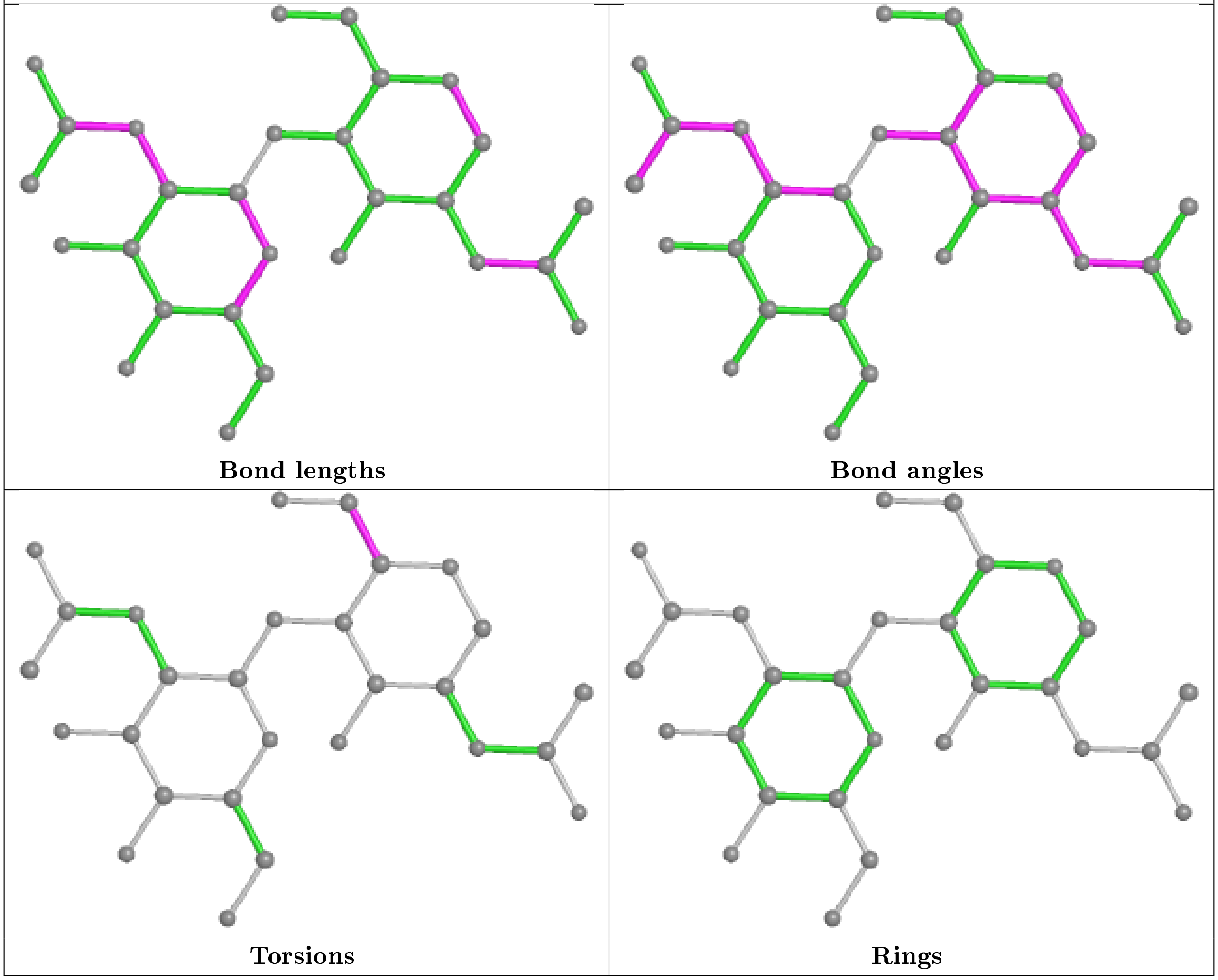

Oligosaccharide Chain C

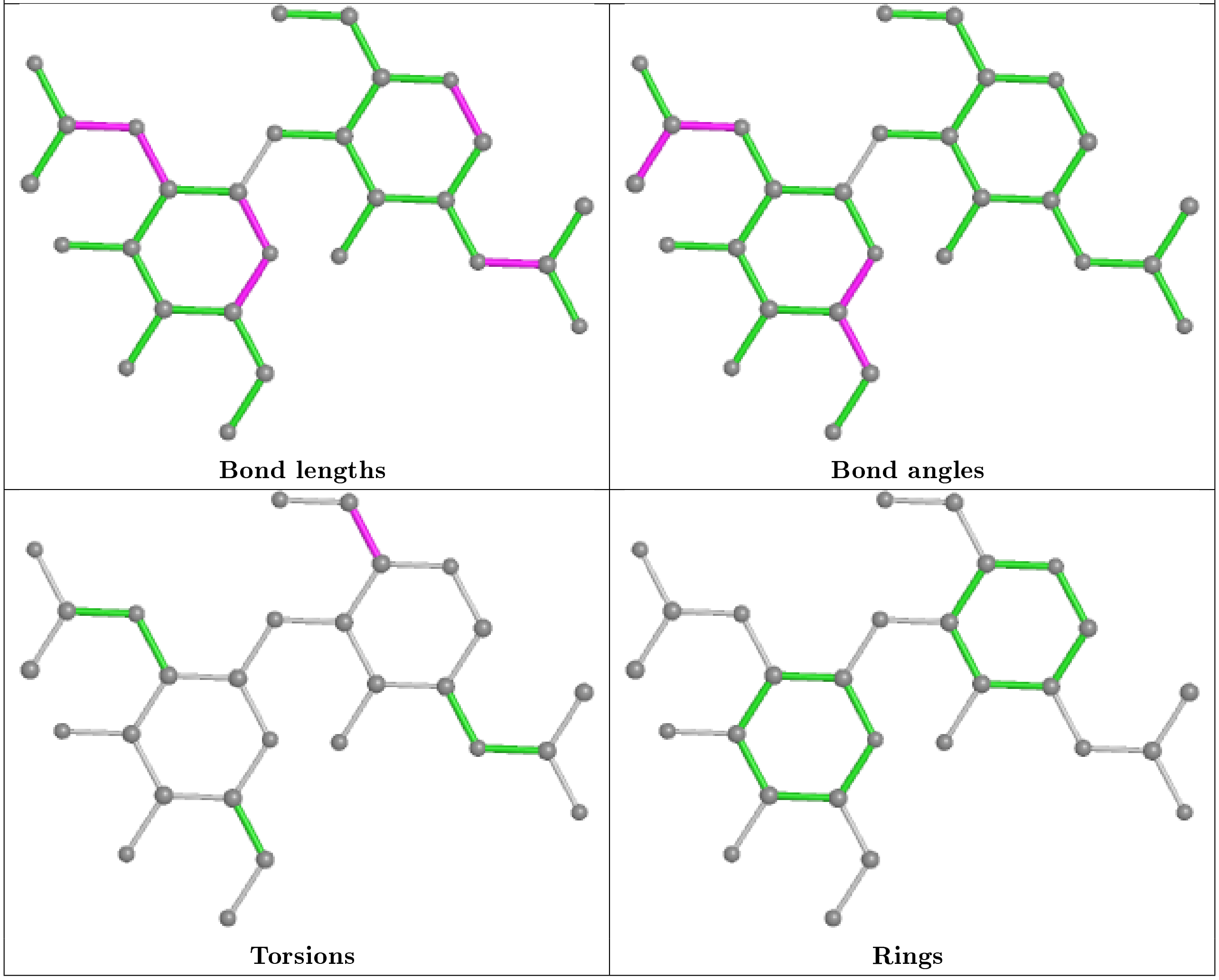

5.6 Ligand geometry ⓘ

Of 25 ligands modelled in this entry, 2 are monoatomic - leaving 23 for Mogul analysis.

In the following table, the Counts columns list the number of bonds (or angles) for which Mogul statistics could be retrieved, the number of bonds (or angles) that are observed in the model and the number of bonds (or angles) that are defined in the Chemical Component Dictionary. The Link column lists molecule types, if any, to which the group is linked. The Z score for a bond length (or angle) is the number of standard deviations the observed value is removed from the expected value. A bond length (or angle) with $|Z| > 2$ is considered an outlier worth inspection. RMSZ is the root-mean-square of all Z scores of the bond lengths (or angles).

Mol	Type	Chain	Res	Link	Bond lengths			Bond angles		
					Counts	RMSZ	$\#\|Z\| > 2$	Counts	RMSZ	$\#\|Z\| > 2$
4	NAG	A	608	1	14,14,15	2.10	5 (35%)	17,19,21	1.70	3 (17%)
8	EDO	A	628	-	3,3,3	0.47	0	2,2,2	0.26	0
4	NAG	A	611	1	14,14,15	1.90	3 (21%)	17,19,21	2.12	5 (29%)

Mol	Type	Chain	Res	Link	Bond lengths			Bond angles		
					Counts	RMSZ	#|Z| > 2	Counts	RMSZ	#|Z| > 2
7	PEG	A	620	-	6,6,6	0.50	0	5,5,5	0.36	0
4	NAG	A	607	1	14,14,15	1.86	2 (14%)	17,19,21	1.35	4 (23%)
8	EDO	A	622	-	3,3,3	0.49	0	2,2,2	0.34	0
8	EDO	A	623	-	3,3,3	0.47	0	2,2,2	0.33	0
5	PO4	A	612	3	4,4,4	0.86	0	6,6,6	0.76	0
4	NAG	A	609	1	14,14,15	1.91	3 (21%)	17,19,21	1.07	1 (5%)
7	PEG	A	621	-	6,6,6	0.48	0	5,5,5	0.39	0
8	EDO	A	625	-	3,3,3	0.47	0	2,2,2	0.24	0
4	NAG	A	610	1	14,14,15	1.86	3 (21%)	17,19,21	1.08	2 (11%)
8	EDO	A	627	-	3,3,3	0.87	0	2,2,2	0.68	0
7	PEG	A	619	-	6,6,6	0.47	0	5,5,5	0.43	0
5	PO4	A	613	-	4,4,4	0.87	0	6,6,6	0.45	0
5	PO4	A	614	-	4,4,4	0.97	0	6,6,6	0.62	0
6	PGE	A	615	-	9,9,9	0.51	0	8,8,8	0.31	0
8	EDO	A	626	-	3,3,3	0.47	0	2,2,2	0.28	0
8	EDO	A	629	-	3,3,3	0.45	0	2,2,2	0.34	0
6	PGE	A	617	-	9,9,9	0.50	0	8,8,8	0.56	0
7	PEG	A	618	-	6,6,6	0.46	0	5,5,5	0.53	0
8	EDO	A	624	-	3,3,3	0.47	0	2,2,2	0.40	0
6	PGE	A	616	-	9,9,9	0.50	0	8,8,8	0.70	0

In the following table, the Chirals column lists the number of chiral outliers, the number of chiral centers analysed, the number of these observed in the model and the number defined in the Chemical Component Dictionary. Similar counts are reported in the Torsion and Rings columns. '-' means no outliers of that kind were identified.

Mol	Type	Chain	Res	Link	Chirals	Torsions	Rings
4	NAG	A	607	1	-	2/6/23/26	0/1/1/1
6	PGE	A	615	-	-	0/7/7/7	-
8	EDO	A	622	-	-	1/1/1/1	-
4	NAG	A	608	1	-	0/6/23/26	0/1/1/1
4	NAG	A	610	1	-	0/6/23/26	0/1/1/1
7	PEG	A	619	-	-	1/4/4/4	-
8	EDO	A	628	-	-	0/1/1/1	-
8	EDO	A	623	-	-	0/1/1/1	-
7	PEG	A	620	-	-	0/4/4/4	-
8	EDO	A	629	-	-	1/1/1/1	-
4	NAG	A	609	1	-	0/6/23/26	0/1/1/1
7	PEG	A	621	-	-	1/4/4/4	-
6	PGE	A	617	-	-	7/7/7/7	-
7	PEG	A	618	-	-	4/4/4/4	-

Continued on next page...

Continued from previous page...

Mol	Type	Chain	Res	Link	Chirals	Torsions	Rings
4	NAG	A	611	1	-	3/6/23/26	0/1/1/1
8	EDO	A	625	-	-	1/1/1/1	-
8	EDO	A	624	-	-	0/1/1/1	-
8	EDO	A	626	-	-	0/1/1/1	-
8	EDO	A	627	-	-	0/1/1/1	-
6	PGE	A	616	-	-	2/7/7/7	-

All (16) bond length outliers are listed below:

Mol	Chain	Res	Type	Atoms	Z	Observed(Å)	Ideal(Å)
4	A	608	NAG	O5-C1	4.89	1.51	1.43
4	A	607	NAG	O5-C1	4.36	1.50	1.43
4	A	609	NAG	O5-C1	4.25	1.50	1.43
4	A	610	NAG	O5-C1	4.12	1.50	1.43
4	A	611	NAG	O5-C1	4.02	1.50	1.43
4	A	609	NAG	C7-N2	3.57	1.46	1.34
4	A	611	NAG	C7-N2	3.52	1.46	1.34
4	A	610	NAG	C7-N2	3.48	1.46	1.34
4	A	608	NAG	C7-N2	3.45	1.46	1.34
4	A	607	NAG	C7-N2	3.32	1.45	1.34
4	A	608	NAG	O5-C5	2.78	1.49	1.43
4	A	611	NAG	C2-N2	2.59	1.50	1.46
4	A	608	NAG	C3-C2	-2.30	1.47	1.52
4	A	609	NAG	C2-N2	2.26	1.50	1.46
4	A	608	NAG	C2-N2	2.06	1.49	1.46
4	A	610	NAG	C2-N2	2.05	1.49	1.46

All (15) bond angle outliers are listed below:

Mol	Chain	Res	Type	Atoms	Z	Observed(°)	Ideal(°)
4	A	611	NAG	C8-C7-N2	5.17	124.85	116.10
4	A	611	NAG	C1-C2-N2	-4.94	102.06	110.49
4	A	608	NAG	C1-C2-N2	-4.16	103.38	110.49
4	A	608	NAG	O5-C1-C2	3.62	117.00	111.29
4	A	611	NAG	C2-N2-C7	3.27	127.56	122.90
4	A	611	NAG	O7-C7-N2	-2.77	116.86	121.95
4	A	608	NAG	O5-C5-C6	2.77	111.54	107.20
4	A	610	NAG	C2-N2-C7	-2.66	119.11	122.90
4	A	607	NAG	C1-O5-C5	-2.59	108.68	112.19
4	A	609	NAG	C8-C7-N2	2.48	120.29	116.10
4	A	607	NAG	C8-C7-N2	2.40	120.16	116.10
4	A	607	NAG	C1-C2-N2	-2.39	106.40	110.49

Continued on next page...

Continued from previous page...

Mol	Chain	Res	Type	Atoms	Z	Observed(°)	Ideal(°)
4	A	610	NAG	C8-C7-N2	2.35	120.08	116.10
4	A	607	NAG	C2-N2-C7	-2.29	119.64	122.90
4	A	611	NAG	O7-C7-C8	-2.04	118.28	122.06

There are no chirality outliers.

All (23) torsion outliers are listed below:

Mol	Chain	Res	Type	Atoms
4	A	611	NAG	C3-C2-N2-C7
6	A	616	PGE	C1-C2-O2-C3
4	A	607	NAG	O5-C5-C6-O6
4	A	607	NAG	C4-C5-C6-O6
4	A	611	NAG	C8-C7-N2-C2
4	A	611	NAG	O7-C7-N2-C2
6	A	617	PGE	O2-C3-C4-O3
7	A	621	PEG	O1-C1-C2-O2
6	A	617	PGE	O1-C1-C2-O2
6	A	616	PGE	O2-C3-C4-O3
6	A	617	PGE	O3-C5-C6-O4
7	A	618	PEG	O1-C1-C2-O2
7	A	618	PEG	O2-C3-C4-O4
8	A	625	EDO	O1-C1-C2-O2
6	A	617	PGE	C3-C4-O3-C5
6	A	617	PGE	C4-C3-O2-C2
7	A	618	PEG	C1-C2-O2-C3
6	A	617	PGE	C1-C2-O2-C3
6	A	617	PGE	C6-C5-O3-C4
8	A	622	EDO	O1-C1-C2-O2
7	A	619	PEG	C1-C2-O2-C3
8	A	629	EDO	O1-C1-C2-O2
7	A	618	PEG	C4-C3-O2-C2

There are no ring outliers.

11 monomers are involved in 18 short contacts:

Mol	Chain	Res	Type	Clashes	Symm-Clashes
4	A	611	NAG	1	0
7	A	620	PEG	1	0
5	A	612	PO4	1	0
7	A	621	PEG	1	0
8	A	625	EDO	2	0

Continued on next page...

Continued from previous page...

Mol	Chain	Res	Type	Clashes	Symm-Clashes
8	A	627	EDO	2	0
5	A	614	PO4	1	0
6	A	617	PGE	1	0
7	A	618	PEG	1	0
8	A	624	EDO	4	0
6	A	616	PGE	3	0

5.7 Other polymers (i)

There are no such residues in this entry.

5.8 Polymer linkage issues (i)

There are no chain breaks in this entry.

6 Fit of model and data (i)

6.1 Protein, DNA and RNA chains (i)

In the following table, the column labelled '#RSRZ> 2' contains the number (and percentage) of RSRZ outliers, followed by percent RSRZ outliers for the chain as percentile scores relative to all X-ray entries and entries of similar resolution. The OWAB column contains the minimum, median, 95th percentile and maximum values of the occupancy-weighted average B-factor per residue. The column labelled 'Q< 0.9' lists the number of (and percentage) of residues with an average occupancy less than 0.9.

Mol	Chain	Analysed	<RSRZ>	#RSRZ>2			OWAB(Å^2)	Q<0.9
1	A	504/516 (97%)	0.07	21 (4%)	36	41	13, 21, 43, 68	0

All (21) RSRZ outliers are listed below:

Mol	Chain	Res	Type	RSRZ
1	A	223	ALA	7.1
1	A	220	CYS	6.5
1	A	224	LYS	5.8
1	A	222	PHE	5.4
1	A	493	ALA	5.3
1	A	2	PRO	5.2
1	A	490	TYR	4.9
1	A	17	LEU	4.2
1	A	492	GLY	4.0
1	A	225	SER	3.6
1	A	19	GLU	3.5
1	A	217	CYS	3.4
1	A	491	GLN	3.3
1	A	221	SER	3.1
1	A	79	THR	2.5
1	A	426	MET	2.5
1	A	23	HIS	2.2
1	A	219	SER	2.1
1	A	109	PRO	2.1
1	A	499	TYR	2.0
1	A	419	PRO	2.0

6.2 Non-standard residues in protein, DNA, RNA chains (i)

There are no non-standard protein/DNA/RNA residues in this entry.

6.3 Carbohydrates (i)

In the following table, the Atoms column lists the number of modelled atoms in the group and the number defined in the chemical component dictionary. The B-factors column lists the minimum, median, 95th percentile and maximum values of B factors of atoms in the group. The column labelled 'Q< 0.9' lists the number of atoms with occupancy less than 0.9.

Mol	Type	Chain	Res	Atoms	RSCC	RSR	B-factors(Å^2)	Q<0.9
2	NAG	B	2	14/15	0.71	0.36	53,63,72,75	28
2	NAG	C	2	14/15	0.76	0.17	42,51,62,65	28
2	NAG	B	1	14/15	0.93	0.16	26,36,45,46	0
2	NAG	C	1	14/15	0.96	0.09	22,28,36,39	0

The following is a graphical depiction of the model fit to experimental electron density for oligosaccharide. Each fit is shown from different orientation to approximate a three-dimensional view.

Electron density around Chain B:

2mF$_o$-DF$_c$ (at 0.7 rmsd) in gray
mF$_o$-DF$_c$ (at 3 rmsd) in purple (negative)
and green (positive)

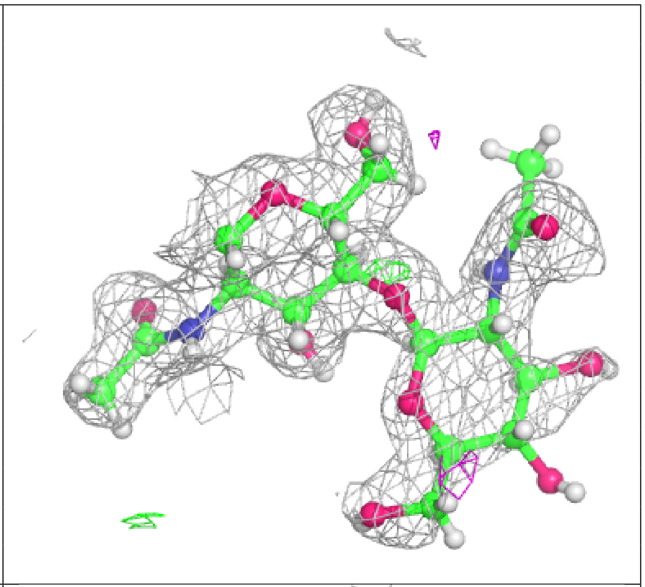

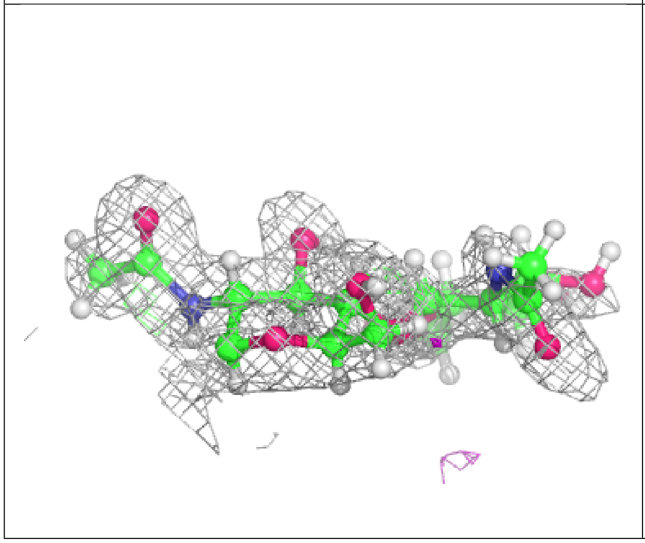

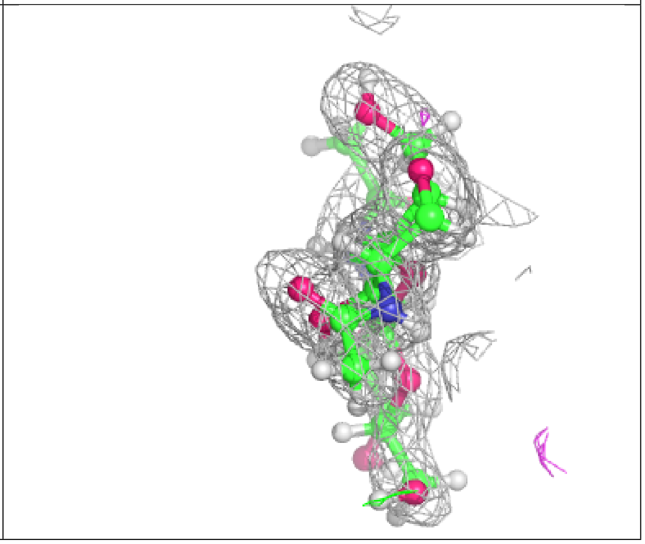

Electron density around Chain C:

2mF$_o$-DF$_c$ (at 0.7 rmsd) in gray
mF$_o$-DF$_c$ (at 3 rmsd) in purple (negative)
and green (positive)

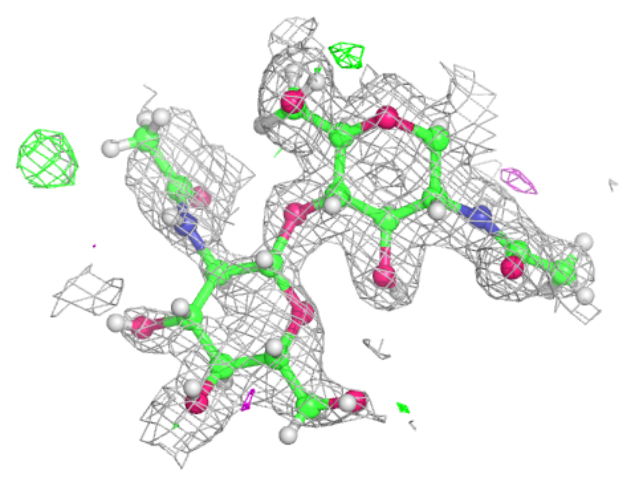

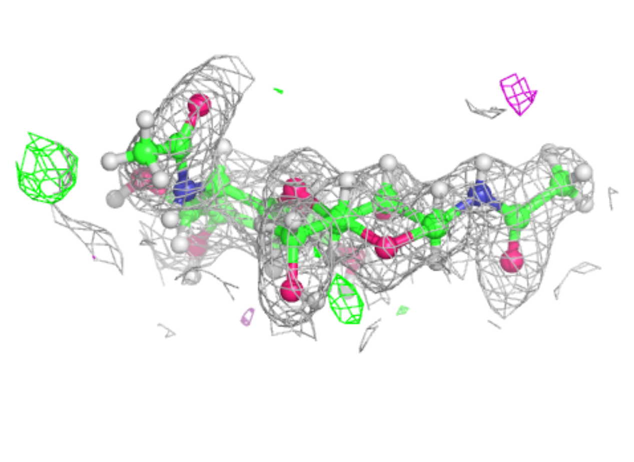

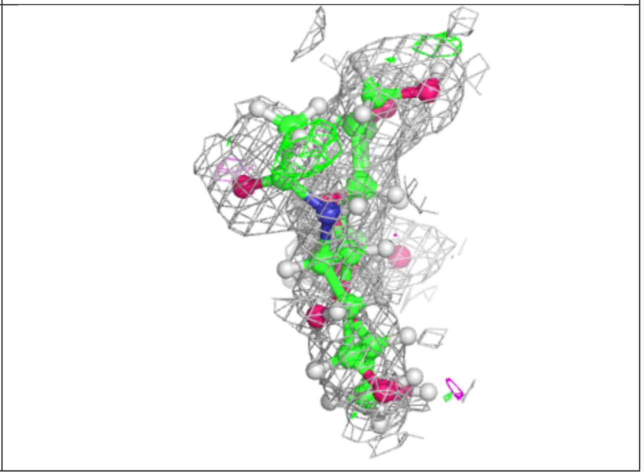

6.4 Ligands (i)

In the following table, the Atoms column lists the number of modelled atoms in the group and the number defined in the chemical component dictionary. The B-factors column lists the minimum, median, 95th percentile and maximum values of B factors of atoms in the group. The column labelled 'Q< 0.9' lists the number of atoms with occupancy less than 0.9.

Mol	Type	Chain	Res	Atoms	RSCC	RSR	B-factors(Å^2)	Q<0.9
8	EDO	A	625	4/4	0.51	0.22	52,63,70,72	0
4	NAG	A	609	14/15	0.60	0.34	48,63,75,77	28
7	PEG	A	619	7/7	0.61	0.23	55,66,74,75	0
4	NAG	A	608	14/15	0.65	0.24	53,66,80,80	28
7	PEG	A	621	7/7	0.65	0.28	60,72,75,76	0
8	EDO	A	626	4/4	0.67	0.18	66,79,83,83	0
6	PGE	A	616	10/10	0.71	0.14	61,73,76,77	0
7	PEG	A	620	7/7	0.74	0.15	66,79,79,80	0
8	EDO	A	623	4/4	0.77	0.14	65,78,79,80	0
6	PGE	A	617	10/10	0.81	0.20	57,70,72,74	0
4	NAG	A	611	14/15	0.82	0.21	35,51,62,62	28

Continued on next page...

Continued from previous page...

Mol	Type	Chain	Res	Atoms	RSCC	RSR	B-factors(Å^2)	Q<0.9
4	NAG	A	607	14/15	0.85	0.14	41,51,62,65	28
7	PEG	A	618	7/7	0.87	0.14	54,65,75,77	0
8	EDO	A	628	4/4	0.87	0.09	35,42,46,51	0
8	EDO	A	629	4/4	0.88	0.28	53,64,64,65	0
8	EDO	A	622	4/4	0.89	0.15	59,71,72,73	0
4	NAG	A	610	14/15	0.89	0.25	39,48,63,63	0
6	PGE	A	615	10/10	0.91	0.10	40,50,63,65	0
5	PO4	A	613	5/5	0.93	0.14	66,66,67,67	5
8	EDO	A	624	4/4	0.95	0.16	48,58,61,66	0
5	PO4	A	612	5/5	0.95	0.13	31,32,35,36	5
8	EDO	A	627	4/4	0.97	0.12	14,32,39,46	0
5	PO4	A	614	5/5	0.98	0.10	36,36,40,41	0
3	FE	A	601	1/1	1.00	0.05	20,20,20,20	1
3	FE	A	602	1/1	1.00	0.07	16,16,16,16	0

6.5 Other polymers (i)

There are no such residues in this entry.

Full wwPDB X-ray Structure Validation Report (i)

Aug 8, 2020 – 06:40 AM BST

PDB ID	:	6GJ2
Title	:	PURPLE ACID PHYTASE FROM WHEAT ISOFORM B2 - COMPLEX WITH INOSITOL HEXASULPHATE
Authors	:	Faba-Rodriguez, R.; Brearley, C.A.; Hemmings, A.M.
Deposited on	:	2018-05-15
Resolution	:	1.68 Å(reported)

This is a Full wwPDB X-ray Structure Validation Report for a publicly released PDB entry.

We welcome your comments at *validation@mail.wwpdb.org*
A user guide is available at
https://www.wwpdb.org/validation/2017/XrayValidationReportHelp
with specific help available everywhere you see the (i) symbol.

The following versions of software and data (see references (i)) were used in the production of this report:

MolProbity	:	4.02b-467
Mogul	:	1.8.5 (274361), CSD as541be (2020)
Xtriage (Phenix)	:	1.13
EDS	:	2.13.1
buster-report	:	1.1.7 (2018)
Percentile statistics	:	20191225.v01 (using entries in the PDB archive December 25th 2019)
Refmac	:	5.8.0158
CCP4	:	7.0.044 (Gargrove)
Ideal geometry (proteins)	:	Engh & Huber (2001)
Ideal geometry (DNA, RNA)	:	Parkinson et al. (1996)
Validation Pipeline (wwPDB-VP)	:	2.13.1

1 Overall quality at a glance (i)

The following experimental techniques were used to determine the structure:
X-RAY DIFFRACTION

The reported resolution of this entry is 1.68 Å.

Percentile scores (ranging between 0-100) for global validation metrics of the entry are shown in the following graphic. The table shows the number of entries on which the scores are based.

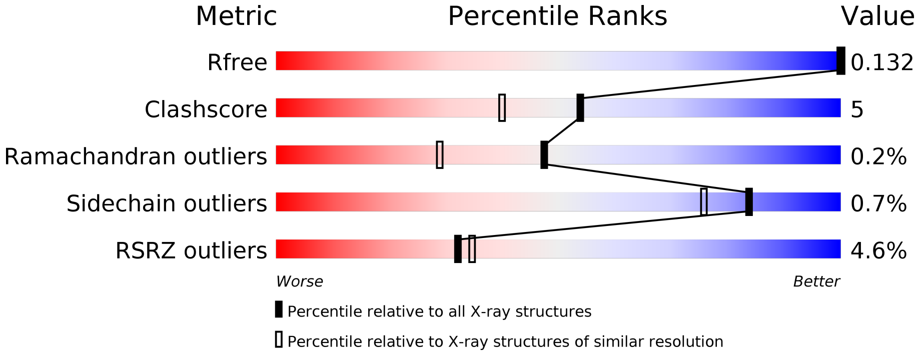

Metric	Whole archive (#Entries)	Similar resolution (#Entries, resolution range(Å))
R_{free}	130704	6780 (1.70-1.66)
Clashscore	141614	7310 (1.70-1.66)
Ramachandran outliers	138981	7173 (1.70-1.66)
Sidechain outliers	138945	7172 (1.70-1.66)
RSRZ outliers	127900	6661 (1.70-1.66)

The table below summarises the geometric issues observed across the polymeric chains and their fit to the electron density. The red, orange, yellow and green segments on the lower bar indicate the fraction of residues that contain outliers for >=3, 2, 1 and 0 types of geometric quality criteria respectively. A grey segment represents the fraction of residues that are not modelled. The numeric value for each fraction is indicated below the corresponding segment, with a dot representing fractions <=5% The upper red bar (where present) indicates the fraction of residues that have poor fit to the electron density. The numeric value is given above the bar.

Mol	Chain	Length	Quality of chain
1	A	516	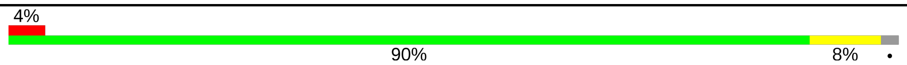
2	B	2	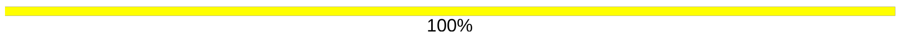

The following table lists non-polymeric compounds, carbohydrate monomers and non-standard residues in protein, DNA, RNA chains that are outliers for geometric or electron-density-fit criteria:

Mol	Type	Chain	Res	Chirality	Geometry	Clashes	Electron density
9	EDO	A	622	-	-	X	-

2 Entry composition (i)

There are 11 unique types of molecules in this entry. The entry contains 4748 atoms, of which 251 are hydrogens and 0 are deuteriums.

In the tables below, the ZeroOcc column contains the number of atoms modelled with zero occupancy, the AltConf column contains the number of residues with at least one atom in alternate conformation and the Trace column contains the number of residues modelled with at most 2 atoms.

- Molecule 1 is a protein called Purple acid phosphatase.

Mol	Chain	Residues	Atoms					ZeroOcc	AltConf	Trace
1	A	504	Total<br>3966	C<br>2522	N<br>670	O<br>750	S<br>24	0	7	0

There are 6 discrepancies between the modelled and reference sequences:

Chain	Residue	Modelled	Actual	Comment	Reference
A	511	HIS	-	expression tag	UNP C4PKL0
A	512	HIS	-	expression tag	UNP C4PKL0
A	513	HIS	-	expression tag	UNP C4PKL0
A	514	HIS	-	expression tag	UNP C4PKL0
A	515	HIS	-	expression tag	UNP C4PKL0
A	516	HIS	-	expression tag	UNP C4PKL0

- Molecule 2 is an oligosaccharide called 2-acetamido-2-deoxy-beta-D-glucopyranose-(1-4)-2-acetamido-2-deoxy-beta-D-glucopyranose.

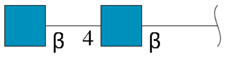

Mol	Chain	Residues	Atoms					ZeroOcc	AltConf	Trace
2	B	2	Total<br>55	C<br>16	H<br>27	N<br>2	O<br>10	0	0	0

- Molecule 3 is FE (III) ION (three-letter code: FE) (formula: Fe).

Mol	Chain	Residues	Atoms		ZeroOcc	AltConf
3	A	2	Total<br>2	Fe<br>2	0	0

- Molecule 4 is 2-acetamido-2-deoxy-beta-D-glucopyranose (three-letter code: NAG) (formula: $C_8H_{15}NO_6$).

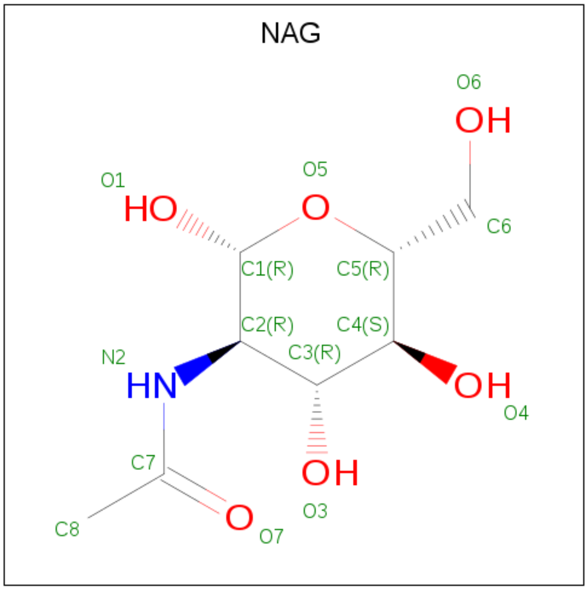

Mol	Chain	Residues	Atoms					ZeroOcc	AltConf
			Total	C	H	N	O		
4	A	1	28	8	14	1	5	0	0
4	A	1	28	8	14	1	5	0	0
4	A	1	28	8	14	1	5	0	0
4	A	1	28	8	14	1	5	0	0
4	A	1	28	8	14	1	5	0	0

- Molecule 5 is DI(HYDROXYETHYL)ETHER (three-letter code: PEG) (formula: $C_4H_{10}O_3$).

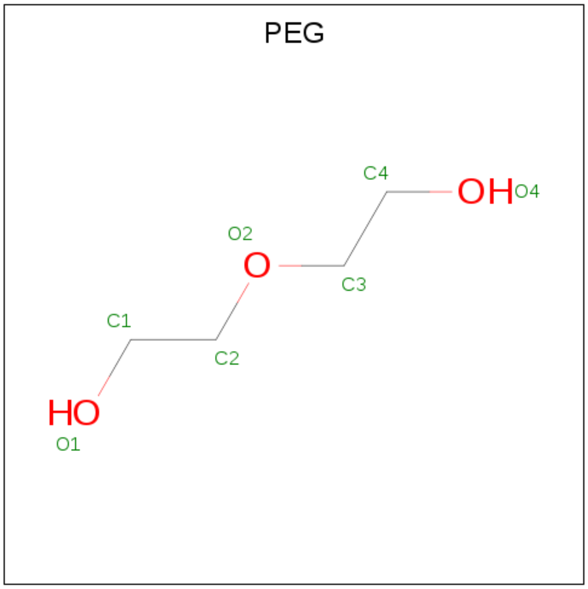

Mol	Chain	Residues	Atoms				ZeroOcc	AltConf
5	A	1	Total 17	C 4	H 10	O 3	0	0
5	A	1	Total 17	C 4	H 10	O 3	0	0
5	A	1	Total 17	C 4	H 10	O 3	0	0
5	A	1	Total 17	C 4	H 10	O 3	0	0
5	A	1	Total 17	C 4	H 10	O 3	0	0

- Molecule 6 is TRIETHYLENE GLYCOL (three-letter code: PGE) (formula: $C_6H_{14}O_4$).

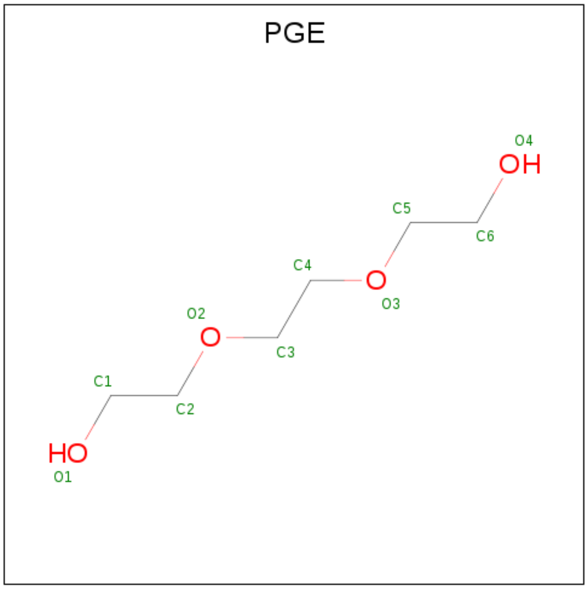

Mol	Chain	Residues	Atoms				ZeroOcc	AltConf
6	A	1	Total 24	C 6	H 14	O 4	0	0
6	A	1	Total 24	C 6	H 14	O 4	0	0
6	A	1	Total 24	C 6	H 14	O 4	0	0
6	A	1	Total 24	C 6	H 14	O 4	0	0

- Molecule 7 is PHOSPHATE ION (three-letter code: PO4) (formula: O_4P).

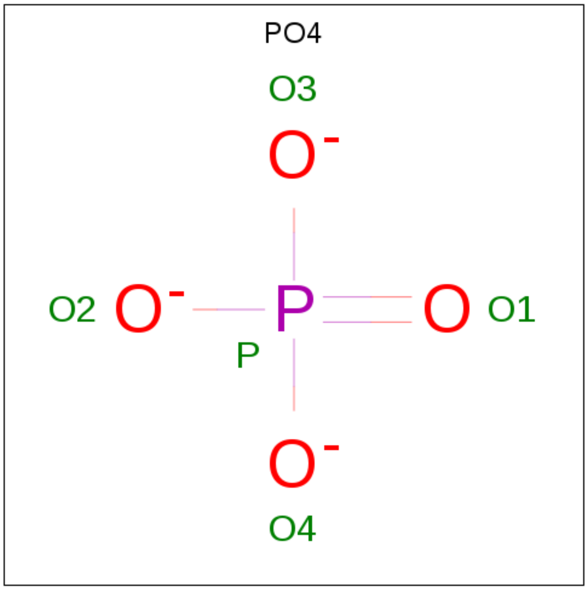

Mol	Chain	Residues	Atoms	ZeroOcc	AltConf
7	A	1	Total O P<br>5 4 1	0	0

- Molecule 8 is TETRAETHYLENE GLYCOL (three-letter code: PG4) (formula: $C_8H_{18}O_5$).

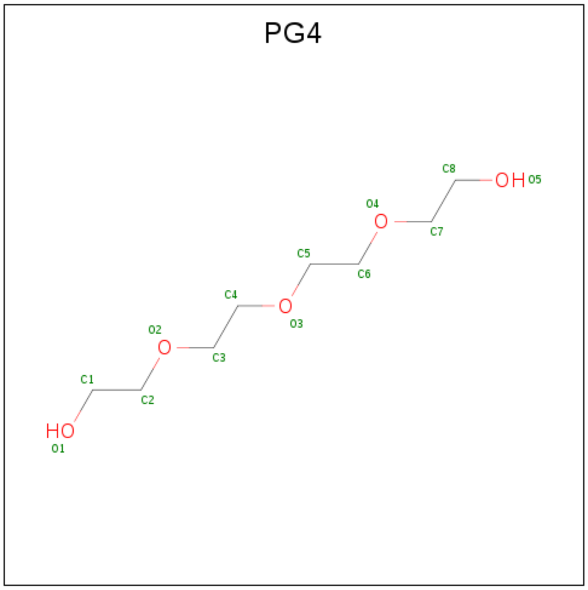

Mol	Chain	Residues	Atoms	ZeroOcc	AltConf
8	A	1	Total C H O<br>31 8 18 5	0	0

- Molecule 9 is 1,2-ETHANEDIOL (three-letter code: EDO) (formula: $C_2H_6O_2$).

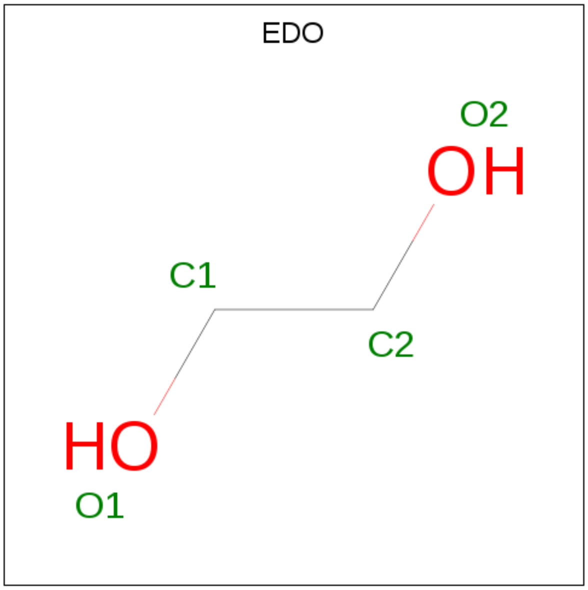

Mol	Chain	Residues	Atoms				ZeroOcc	AltConf
9	A	1	Total 10	C 2	H 6	O 2	0	0
9	A	1	Total 10	C 2	H 6	O 2	0	0
9	A	1	Total 10	C 2	H 6	O 2	0	0
9	A	1	Total 10	C 2	H 6	O 2	0	0

- Molecule 10 is D-MYO-INOSITOL-HEXASULPHATE (three-letter code: IHS) (formula: $C_6H_{12}O_{24}S_6$).

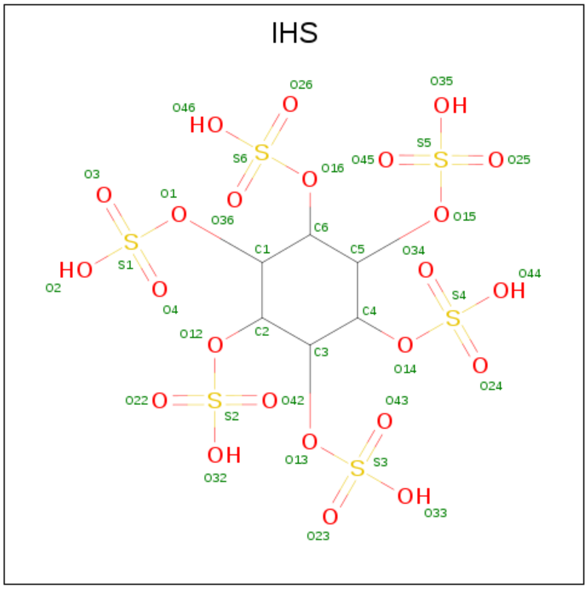

Mol	Chain	Residues	Atoms					ZeroOcc	AltConf
			Total	C	H	O	S		
10	A	1	42	6	6	24	6	0	0

- Molecule 11 is water.

Mol	Chain	Residues	Atoms		ZeroOcc	AltConf
			Total	O		
11	A	286	286	286	0	0

3 Residue-property plots (i)

These plots are drawn for all protein, RNA, DNA and oligosaccharide chains in the entry. The first graphic for a chain summarises the proportions of the various outlier classes displayed in the second graphic. The second graphic shows the sequence view annotated by issues in geometry and electron density. Residues are color-coded according to the number of geometric quality criteria for which they contain at least one outlier: green = 0, yellow = 1, orange = 2 and red = 3 or more. A red dot above a residue indicates a poor fit to the electron density (RSRZ > 2). Stretches of 2 or more consecutive residues without any outlier are shown as a green connector. Residues present in the sample, but not in the model, are shown in grey.

- Molecule 1: Purple acid phosphatase

Chain A:
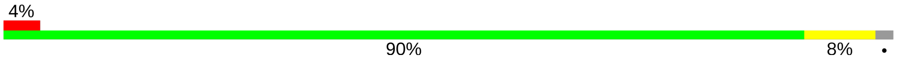

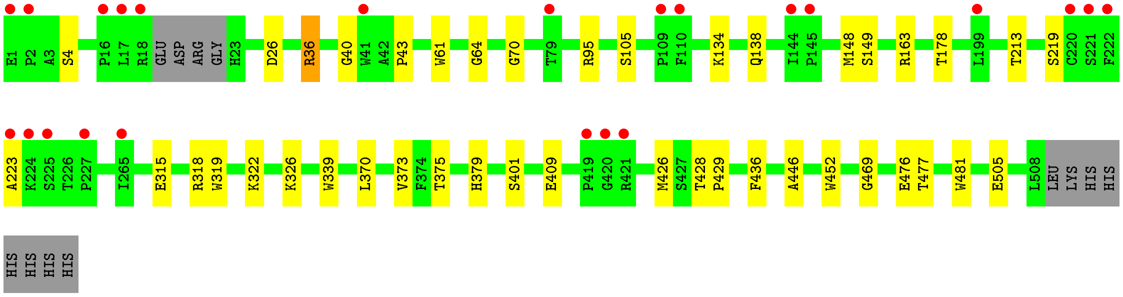

- Molecule 2: 2-acetamido-2-deoxy-beta-D-glucopyranose-(1-4)-2-acetamido-2-deoxy-beta-D-gluc opyranose

Chain B:
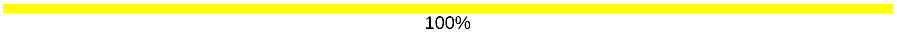

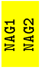

4 Data and refinement statistics (i)

Property	Value	Source
Space group	H 3	Depositor
Cell constants<br>a, b, c, α, β, γ	126.02Å 126.02Å 105.91Å<br>90.00° 90.00° 120.00°	Depositor
Resolution (Å)	38.44 – 1.68<br>38.44 – 1.68	Depositor<br>EDS
% Data completeness<br>(in resolution range)	99.9 (38.44-1.68)<br>99.9 (38.44-1.68)	Depositor<br>EDS
R_{merge}	0.06	Depositor
R_{sym}	(Not available)	Depositor
$< I/\sigma(I) >$ [1]	1.56 (at 1.68Å)	Xtriage
Refinement program	PHENIX	Depositor
R, R_{free}	0.134 , 0.176<br>0.136 , 0.132	Depositor<br>DCC
R_{free} test set	3791 reflections (5.31%)	wwPDB-VP
Wilson B-factor (Å^2)	26.2	Xtriage
Anisotropy	0.240	Xtriage
Bulk solvent k_{sol}(e/Å^3), B_{sol}(Å^2)	0.41 , 54.7	EDS
L-test for twinning[2]	$< \|L\| >$ = 0.50, $< L^2 >$ = 0.34	Xtriage
Estimated twinning fraction	0.020 for h,-h-k,-l	Xtriage
F_o,F_c correlation	0.98	EDS
Total number of atoms	4748	wwPDB-VP
Average B, all atoms (Å^2)	37.0	wwPDB-VP

Xtriage's analysis on translational NCS is as follows: *The largest off-origin peak in the Patterson function is 4.15% of the height of the origin peak. No significant pseudotranslation is detected.*

[1] Intensities estimated from amplitudes.

[2] Theoretical values of $< |L| >$, $< L^2 >$ for acentric reflections are 0.5, 0.333 respectively for untwinned datasets, and 0.375, 0.2 for perfectly twinned datasets.

5 Model quality (i)

5.1 Standard geometry (i)

Bond lengths and bond angles in the following residue types are not validated in this section: PGE, NAG, IHS, PO4, EDO, PG4, FE, PEG

The Z score for a bond length (or angle) is the number of standard deviations the observed value is removed from the expected value. A bond length (or angle) with $|Z| > 5$ is considered an outlier worth inspection. RMSZ is the root-mean-square of all Z scores of the bond lengths (or angles).

Mol	Chain	Bond lengths		Bond angles	
		RMSZ	$\#\|Z\| > 5$	RMSZ	$\#\|Z\| > 5$
1	A	0.34	0/4111	0.52	0/5609

There are no bond length outliers.

There are no bond angle outliers.

There are no chirality outliers.

There are no planarity outliers.

5.2 Too-close contacts (i)

In the following table, the Non-H and H(model) columns list the number of non-hydrogen atoms and hydrogen atoms in the chain respectively. The H(added) column lists the number of hydrogen atoms added and optimized by MolProbity. The Clashes column lists the number of clashes within the asymmetric unit, whereas Symm-Clashes lists symmetry related clashes.

Mol	Chain	Non-H	H(model)	H(added)	Clashes	Symm-Clashes
1	A	3966	0	3722	42	0
2	B	28	27	25	0	0
3	A	2	0	0	0	0
4	A	70	70	65	2	0
5	A	35	50	50	8	0
6	A	40	56	56	7	0
7	A	5	0	0	0	0
8	A	13	18	18	0	0
9	A	16	24	24	4	0
10	A	36	6	12	1	0
11	A	286	0	0	1	0
All	All	4497	251	3972	43	0

The all-atom clashscore is defined as the number of clashes found per 1000 atoms (including

hydrogen atoms). The all-atom clashscore for this structure is 5.

All (43) close contacts within the same asymmetric unit are listed below, sorted by their clash magnitude.

Atom-1	Atom-2	Interatomic distance (Å)	Clash overlap (Å)
1:A:213:THR:OG1	4:A:605:NAG:N2	2.18	0.74
1:A:134:LYS:HE2	6:A:616:PGE:H62	1.73	0.70
1:A:446:ALA:HA	5:A:613:PEG:H22	1.76	0.68
1:A:315:GLU:HG3	9:A:622:EDO:H11	1.78	0.65
1:A:64:GLY:HA2	6:A:617:PGE:H3	1.78	0.64
1:A:219:SER:HB3	10:A:625:IHS:H3	1.81	0.62
1:A:319:TRP:HB2	9:A:622:EDO:H12	1.80	0.62
1:A:315:GLU:HG3	9:A:622:EDO:C1	2.31	0.60
1:A:95:ARG:HH21	5:A:614:PEG:H22	1.66	0.60
1:A:213:THR:HG1	4:A:605:NAG:HN2	1.46	0.59
1:A:429:PRO:CG	1:A:436:PHE:HD1	2.16	0.58
1:A:163:ARG:NH2	5:A:612:PEG:H31	2.20	0.56
1:A:219:SER:HA	1:A:223:ALA:HB2	1.88	0.56
1:A:105:SER:H	5:A:611:PEG:H11	1.71	0.54
1:A:138[B]:GLN:HG3	1:A:148:MET:CE	2.37	0.54
1:A:134:LYS:HE2	6:A:616:PGE:C6	2.37	0.53
1:A:476[A]:GLU:HG3	1:A:477:THR:HG23	1.91	0.53
1:A:429:PRO:HG3	1:A:436:PHE:HD1	1.72	0.52
1:A:138[A]:GLN:HG2	1:A:149:SER:O	2.10	0.51
1:A:95:ARG:HB3	5:A:614:PEG:H32	1.92	0.51
1:A:318:ARG:HG2	9:A:622:EDO:H22	1.92	0.51
1:A:370:LEU:HD21	1:A:373:VAL:HG22	1.93	0.51
1:A:469:GLY:HA3	1:A:481:TRP:CH2	2.45	0.51
1:A:318:ARG:HE	1:A:322:LYS:HZ1	1.60	0.49
1:A:452:TRP:HA	5:A:613:PEG:H21	1.94	0.48
5:A:610:PEG:H12	6:A:616:PGE:H12	1.96	0.46
1:A:43:PRO:HB3	1:A:61:TRP:CD1	2.51	0.46
1:A:326:LYS:HD2	6:A:615:PGE:H52	1.99	0.45
1:A:138[B]:GLN:HG3	1:A:148:MET:HE2	1.98	0.45
1:A:379:HIS:HE1	1:A:409:GLU:OE1	2.00	0.45
1:A:36[B]:ARG:NH2	1:A:40:GLY:O	2.49	0.45
1:A:105:SER:H	5:A:611:PEG:C1	2.31	0.44
1:A:134:LYS:NZ	6:A:616:PGE:H6	2.32	0.44
1:A:429:PRO:HG3	1:A:436:PHE:CD1	2.53	0.43
1:A:339:TRP:O	1:A:375:THR:HA	2.19	0.43
1:A:138[B]:GLN:HG3	1:A:148:MET:HE3	2.01	0.42
1:A:326:LYS:NZ	6:A:615:PGE:H52	2.35	0.41
1:A:428:THR:N	1:A:429:PRO:CD	2.83	0.41

Continued on next page...

Continued from previous page...

Atom-1	Atom-2	Interatomic distance (Å)	Clash overlap (Å)
1:A:426:MET:CE	1:A:426:MET:HA	2.50	0.41
1:A:505:GLU:HG3	11:A:797:HOH:O	2.20	0.41
1:A:318:ARG:NE	1:A:322:LYS:HZ1	2.19	0.41
1:A:429:PRO:HG2	1:A:436:PHE:HD1	1.85	0.40
1:A:26:ASP:OD1	1:A:178:THR:HG22	2.21	0.40

There are no symmetry-related clashes.

5.3 Torsion angles (i)

5.3.1 Protein backbone (i)

In the following table, the Percentiles column shows the percent Ramachandran outliers of the chain as a percentile score with respect to all X-ray entries followed by that with respect to entries of similar resolution.

The Analysed column shows the number of residues for which the backbone conformation was analysed, and the total number of residues.

Mol	Chain	Analysed	Favoured	Allowed	Outliers	Percentiles	
1	A	507/516 (98%)	488 (96%)	18 (4%)	1 (0%)	47	29

All (1) Ramachandran outliers are listed below:

Mol	Chain	Res	Type
1	A	70	GLY

5.3.2 Protein sidechains (i)

In the following table, the Percentiles column shows the percent sidechain outliers of the chain as a percentile score with respect to all X-ray entries followed by that with respect to entries of similar resolution.

The Analysed column shows the number of residues for which the sidechain conformation was analysed, and the total number of residues.

Mol	Chain	Analysed	Rotameric	Outliers	Percentiles	
1	A	417/425 (98%)	413 (99%)	4 (1%)	76	65

All (4) residues with a non-rotameric sidechain are listed below:

Mol	Chain	Res	Type
1	A	4	SER
1	A	36[A]	ARG
1	A	36[B]	ARG
1	A	401	SER

Some sidechains can be flipped to improve hydrogen bonding and reduce clashes. There are no such sidechains identified.

5.3.3 RNA (i)

There are no RNA molecules in this entry.

5.4 Non-standard residues in protein, DNA, RNA chains (i)

There are no non-standard protein/DNA/RNA residues in this entry.

5.5 Carbohydrates (i)

2 monosaccharides are modelled in this entry.

In the following table, the Counts columns list the number of bonds (or angles) for which Mogul statistics could be retrieved, the number of bonds (or angles) that are observed in the model and the number of bonds (or angles) that are defined in the Chemical Component Dictionary. The Link column lists molecule types, if any, to which the group is linked. The Z score for a bond length (or angle) is the number of standard deviations the observed value is removed from the expected value. A bond length (or angle) with $|Z| > 2$ is considered an outlier worth inspection. RMSZ is the root-mean-square of all Z scores of the bond lengths (or angles).

Mol	Type	Chain	Res	Link	Bond lengths			Bond angles		
					Counts	RMSZ	$\#\|Z\| > 2$	Counts	RMSZ	$\#\|Z\| > 2$
2	NAG	B	1	1,2	14,14,15	1.77	2 (14%)	17,19,21	1.02	1 (5%)
2	NAG	B	2	2	14,14,15	1.97	4 (28%)	17,19,21	1.12	2 (11%)

In the following table, the Chirals column lists the number of chiral outliers, the number of chiral centers analysed, the number of these observed in the model and the number defined in the Chemical Component Dictionary. Similar counts are reported in the Torsion and Rings columns. '-' means no outliers of that kind were identified.

Mol	Type	Chain	Res	Link	Chirals	Torsions	Rings
2	NAG	B	1	1,2	-	0/6/23/26	0/1/1/1
2	NAG	B	2	2	-	0/6/23/26	0/1/1/1

All (6) bond length outliers are listed below:

Mol	Chain	Res	Type	Atoms	Z	Observed(Å)	Ideal(Å)
2	B	2	NAG	O5-C1	4.29	1.50	1.43
2	B	1	NAG	O5-C1	4.15	1.50	1.43
2	B	2	NAG	C7-N2	3.64	1.46	1.34
2	B	1	NAG	C7-N2	3.38	1.46	1.34
2	B	2	NAG	C2-N2	2.57	1.50	1.46
2	B	2	NAG	O5-C5	2.00	1.47	1.43

All (3) bond angle outliers are listed below:

Mol	Chain	Res	Type	Atoms	Z	Observed(^{o})	Ideal(^{o})
2	B	2	NAG	C8-C7-N2	3.01	121.19	116.10
2	B	1	NAG	C1-C2-N2	-2.66	105.95	110.49
2	B	2	NAG	C2-N2-C7	-2.00	120.05	122.90

There are no chirality outliers.

There are no torsion outliers.

There are no ring outliers.

No monomer is involved in short contacts.

The following is a two-dimensional graphical depiction of Mogul quality analysis of bond lengths, bond angles, torsion angles, and ring geometry for oligosaccharide.

Oligosaccharide Chain B

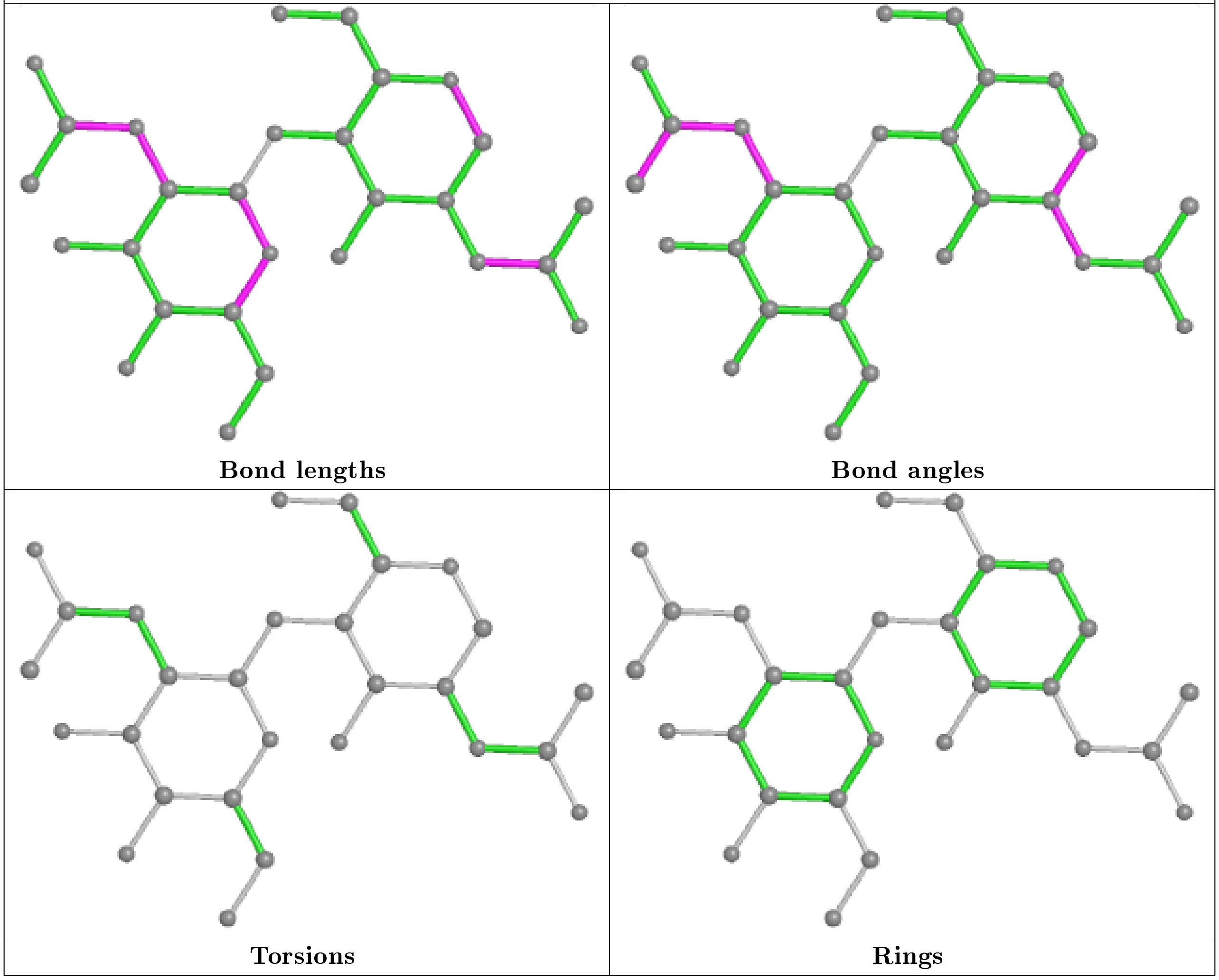

5.6 Ligand geometry (i)

Of 23 ligands modelled in this entry, 2 are monoatomic - leaving 21 for Mogul analysis.

In the following table, the Counts columns list the number of bonds (or angles) for which Mogul statistics could be retrieved, the number of bonds (or angles) that are observed in the model and the number of bonds (or angles) that are defined in the Chemical Component Dictionary. The Link column lists molecule types, if any, to which the group is linked. The Z score for a bond length (or angle) is the number of standard deviations the observed value is removed from the expected value. A bond length (or angle) with $|Z| > 2$ is considered an outlier worth inspection. RMSZ is the root-mean-square of all Z scores of the bond lengths (or angles).

Mol	Type	Chain	Res	Link	Bond lengths			Bond angles		
					Counts	RMSZ	$\#\|Z\| > 2$	Counts	RMSZ	$\#\|Z\| > 2$
6	PGE	A	616	-	9,9,9	0.53	0	8,8,8	0.88	0
10	IHS	A	625	-	36,36,36	1.84	12 (33%)	42,60,60	1.31	6 (14%)
5	PEG	A	613	-	6,6,6	0.46	0	5,5,5	0.34	0

Mol	Type	Chain	Res	Link	Bond lengths			Bond angles		
					Counts	RMSZ	#|Z| > 2	Counts	RMSZ	#|Z| > 2
9	EDO	A	621	-	3,3,3	0.47	0	2,2,2	0.31	0
5	PEG	A	614	-	6,6,6	0.49	0	5,5,5	0.24	0
4	NAG	A	604	1	14,14,15	1.66	2 (14%)	17,19,21	0.93	1 (5%)
4	NAG	A	607	1	14,14,15	1.84	2 (14%)	17,19,21	1.18	2 (11%)
5	PEG	A	611	-	6,6,6	0.47	0	5,5,5	0.76	0
5	PEG	A	610	-	6,6,6	0.50	0	5,5,5	0.33	0
9	EDO	A	624	-	3,3,3	0.47	0	2,2,2	0.28	0
4	NAG	A	603	1	14,14,15	1.77	2 (14%)	17,19,21	1.09	1 (5%)
9	EDO	A	623	-	3,3,3	0.48	0	2,2,2	0.29	0
6	PGE	A	615	-	9,9,9	0.52	0	8,8,8	0.35	0
8	PG4	A	620	-	12,12,12	0.52	0	11,11,11	0.35	0
7	PO4	A	619	3	4,4,4	0.90	0	6,6,6	0.59	0
4	NAG	A	605	1	14,14,15	1.88	3 (21%)	17,19,21	1.27	3 (17%)
9	EDO	A	622	-	3,3,3	0.45	0	2,2,2	0.27	0
6	PGE	A	617	-	9,9,9	0.51	0	8,8,8	0.87	0
4	NAG	A	606	1	14,14,15	1.94	3 (21%)	17,19,21	1.07	1 (5%)
5	PEG	A	612	-	6,6,6	0.51	0	5,5,5	0.48	0
6	PGE	A	618	-	9,9,9	0.51	0	8,8,8	0.50	0

In the following table, the Chirals column lists the number of chiral outliers, the number of chiral centers analysed, the number of these observed in the model and the number defined in the Chemical Component Dictionary. Similar counts are reported in the Torsion and Rings columns. '-' means no outliers of that kind were identified.

Mol	Type	Chain	Res	Link	Chirals	Torsions	Rings
6	PGE	A	616	-	-	0/7/7/7	-
10	IHS	A	625	-	-	2/30/54/54	0/1/1/1
5	PEG	A	613	-	-	0/4/4/4	-
9	EDO	A	621	-	-	1/1/1/1	-
5	PEG	A	614	-	-	1/4/4/4	-
4	NAG	A	604	1	-	0/6/23/26	0/1/1/1
4	NAG	A	607	1	-	0/6/23/26	0/1/1/1
5	PEG	A	611	-	-	1/4/4/4	-
5	PEG	A	610	-	-	0/4/4/4	-
9	EDO	A	624	-	-	1/1/1/1	-
4	NAG	A	603	1	-	0/6/23/26	0/1/1/1
9	EDO	A	623	-	-	0/1/1/1	-
6	PGE	A	615	-	-	3/7/7/7	-
8	PG4	A	620	-	-	1/10/10/10	-
4	NAG	A	605	1	-	0/6/23/26	0/1/1/1
9	EDO	A	622	-	-	0/1/1/1	-

Continued on next page...

Continued from previous page...

Mol	Type	Chain	Res	Link	Chirals	Torsions	Rings
6	PGE	A	617	-	-	4/7/7/7	-
4	NAG	A	606	1	-	0/6/23/26	0/1/1/1
5	PEG	A	612	-	-	0/4/4/4	-
6	PGE	A	618	-	-	4/7/7/7	-

All (24) bond length outliers are listed below:

Mol	Chain	Res	Type	Atoms	Z	Observed(Å)	Ideal(Å)
4	A	606	NAG	O5-C1	4.62	1.51	1.43
4	A	607	NAG	O5-C1	4.24	1.50	1.43
4	A	603	NAG	O5-C1	4.11	1.50	1.43
4	A	605	NAG	O5-C1	3.99	1.50	1.43
4	A	604	NAG	O5-C1	3.75	1.49	1.43
4	A	605	NAG	C7-N2	3.64	1.46	1.34
4	A	606	NAG	C7-N2	3.43	1.46	1.34
4	A	607	NAG	C7-N2	3.43	1.46	1.34
4	A	603	NAG	C7-N2	3.34	1.45	1.34
4	A	604	NAG	C7-N2	3.26	1.45	1.34
10	A	625	IHS	O1-S1	3.16	1.66	1.57
10	A	625	IHS	O15-S5	3.11	1.66	1.57
10	A	625	IHS	O12-S2	2.98	1.66	1.57
10	A	625	IHS	O16-S6	2.91	1.65	1.57
10	A	625	IHS	O13-S3	2.87	1.65	1.57
10	A	625	IHS	O14-S4	2.78	1.65	1.57
4	A	605	NAG	C2-N2	2.67	1.50	1.46
10	A	625	IHS	O14-C4	-2.63	1.41	1.46
10	A	625	IHS	O13-C3	-2.62	1.41	1.46
10	A	625	IHS	O12-C2	-2.58	1.41	1.46
10	A	625	IHS	O16-C6	-2.48	1.41	1.46
10	A	625	IHS	O15-C5	-2.46	1.41	1.46
10	A	625	IHS	O1-C1	-2.30	1.41	1.46
4	A	606	NAG	C2-N2	2.12	1.49	1.46

All (14) bond angle outliers are listed below:

Mol	Chain	Res	Type	Atoms	Z	Observed(^{o})	Ideal(^{o})
4	A	607	NAG	C2-N2-C7	-2.95	118.70	122.90
10	A	625	IHS	O4-S1-O3	-2.91	100.55	112.22
10	A	625	IHS	O36-S6-O26	-2.86	100.76	112.22
10	A	625	IHS	O42-S2-O22	-2.84	100.81	112.22
10	A	625	IHS	O43-S3-O23	-2.77	101.08	112.22
10	A	625	IHS	O34-S4-O24	-2.71	101.36	112.22

Continued on next page...

Continued from previous page...

Mol	Chain	Res	Type	Atoms	Z	Observed(°)	Ideal(°)
4	A	605	NAG	C8-C7-N2	2.65	120.58	116.10
4	A	603	NAG	C2-N2-C7	-2.55	119.28	122.90
4	A	607	NAG	C8-C7-N2	2.44	120.23	116.10
4	A	605	NAG	C1-C2-N2	-2.17	106.78	110.49
4	A	604	NAG	C2-N2-C7	-2.16	119.83	122.90
10	A	625	IHS	O35-S5-O25	-2.15	101.02	108.49
4	A	606	NAG	C1-O5-C5	-2.14	109.30	112.19
4	A	605	NAG	O5-C1-C2	2.04	114.50	111.29

There are no chirality outliers.

All (18) torsion outliers are listed below:

Mol	Chain	Res	Type	Atoms
6	A	617	PGE	C4-C3-O2-C2
6	A	615	PGE	O2-C3-C4-O3
6	A	617	PGE	O2-C3-C4-O3
6	A	618	PGE	C4-C3-O2-C2
6	A	615	PGE	O3-C5-C6-O4
5	A	611	PEG	C1-C2-O2-C3
5	A	614	PEG	C4-C3-O2-C2
6	A	617	PGE	C6-C5-O3-C4
6	A	615	PGE	C3-C4-O3-C5
6	A	618	PGE	C3-C4-O3-C5
10	A	625	IHS	C1-C6-O16-S6
6	A	617	PGE	C3-C4-O3-C5
9	A	624	EDO	O1-C1-C2-O2
9	A	621	EDO	O1-C1-C2-O2
10	A	625	IHS	C6-O16-S6-O46
8	A	620	PG4	O3-C5-C6-O4
6	A	618	PGE	C6-C5-O3-C4
6	A	618	PGE	O2-C3-C4-O3

There are no ring outliers.

11 monomers are involved in 21 short contacts:

Mol	Chain	Res	Type	Clashes	Symm-Clashes
6	A	616	PGE	4	0
10	A	625	IHS	1	0
5	A	613	PEG	2	0
5	A	614	PEG	2	0
5	A	611	PEG	2	0

Continued on next page...

Continued from previous page...

Mol	Chain	Res	Type	Clashes	Symm-Clashes
5	A	610	PEG	1	0
6	A	615	PGE	2	0
4	A	605	NAG	2	0
9	A	622	EDO	4	0
6	A	617	PGE	1	0
5	A	612	PEG	1	0

The following is a two-dimensional graphical depiction of Mogul quality analysis of bond lengths, bond angles, torsion angles, and ring geometry for all instances of the Ligand of Interest. In addition, ligands with molecular weight > 250 and outliers as shown on the validation Tables will also be included. For torsion angles, if less then 5% of the Mogul distribution of torsion angles is within 10 degrees of the torsion angle in question, then that torsion angle is considered an outlier. Any bond that is central to one or more torsion angles identified as an outlier by Mogul will be highlighted in the graph. For rings, the root-mean-square deviation (RMSD) between the ring in question and similar rings identified by Mogul is calculated over all ring torsion angles. If the average RMSD is greater than 60 degrees and the minimal RMSD between the ring in question and any Mogul-identified rings is also greater than 60 degrees, then that ring is considered an outlier. The outliers are highlighted in purple. The color gray indicates Mogul did not find sufficient equivalents in the CSD to analyse the geometry.

Ligand IHS A 625

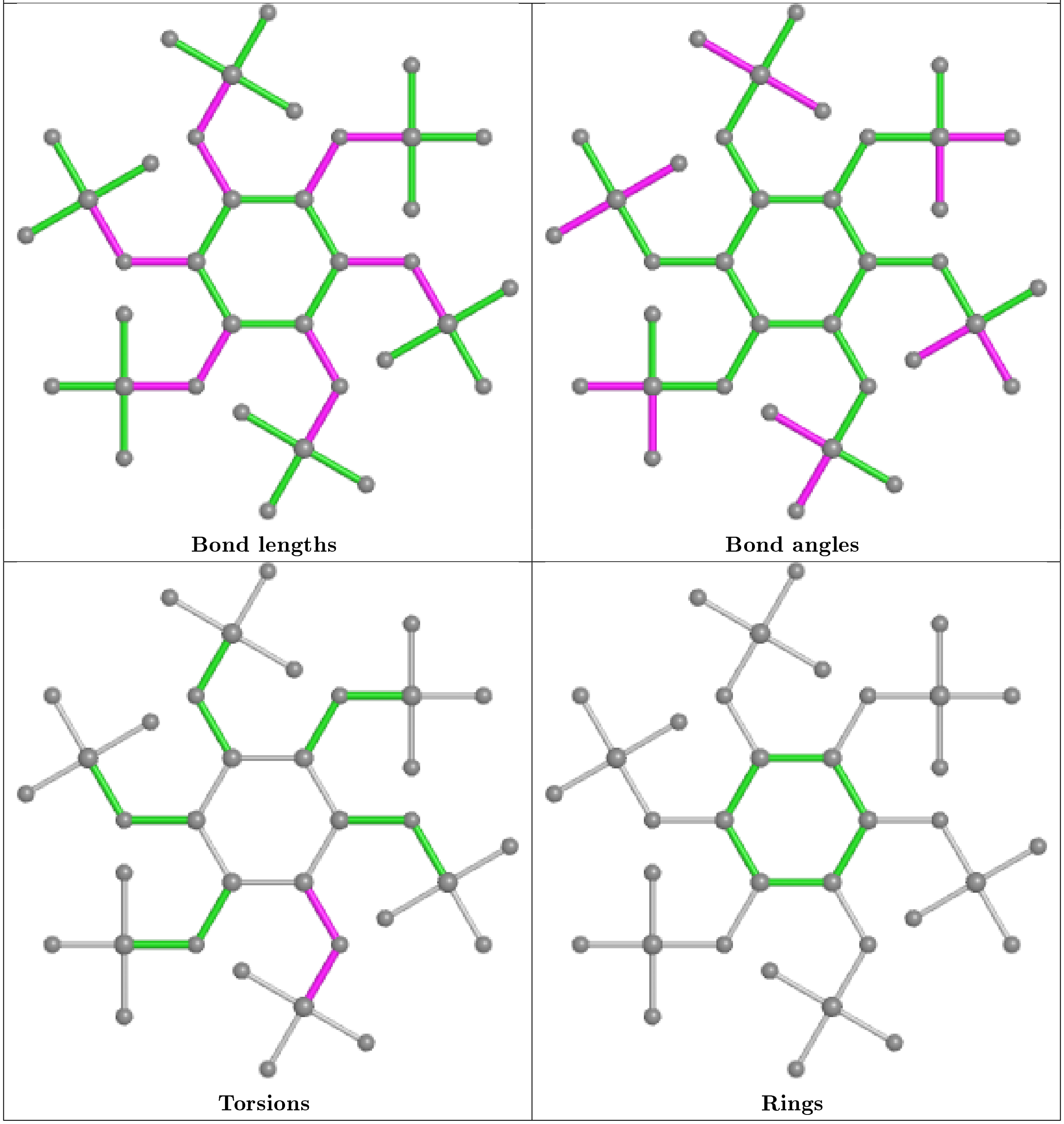

5.7 Other polymers (i)

There are no such residues in this entry.

5.8 Polymer linkage issues (i)

There are no chain breaks in this entry.

6 Fit of model and data

6.1 Protein, DNA and RNA chains

In the following table, the column labelled '#RSRZ> 2' contains the number (and percentage) of RSRZ outliers, followed by percent RSRZ outliers for the chain as percentile scores relative to all X-ray entries and entries of similar resolution. The OWAB column contains the minimum, median, 95th percentile and maximum values of the occupancy-weighted average B-factor per residue. The column labelled 'Q< 0.9' lists the number of (and percentage) of residues with an average occupancy less than 0.9.

Mol	Chain	Analysed	<RSRZ>	#RSRZ>2			OWAB(Å^2)	Q<0.9
1	A	504/516 (97%)	-0.12	23 (4%)	32	35	20, 29, 52, 76	0

All (23) RSRZ outliers are listed below:

Mol	Chain	Res	Type	RSRZ
1	A	17	LEU	7.2
1	A	220	CYS	6.0
1	A	223	ALA	4.9
1	A	222	PHE	4.8
1	A	1	GLU	4.4
1	A	2	PRO	3.6
1	A	419	PRO	3.3
1	A	18	ARG	3.1
1	A	145	PRO	3.1
1	A	221	SER	3.0
1	A	225	SER	3.0
1	A	109	PRO	2.9
1	A	110	PHE	2.8
1	A	224	LYS	2.6
1	A	421	ARG	2.6
1	A	144	ILE	2.4
1	A	16	PRO	2.4
1	A	265	ILE	2.3
1	A	41	TRP	2.2
1	A	420	GLY	2.2
1	A	79	THR	2.1
1	A	199	LEU	2.1
1	A	227	PRO	2.0

6.2 Non-standard residues in protein, DNA, RNA chains ⓘ

There are no non-standard protein/DNA/RNA residues in this entry.

6.3 Carbohydrates ⓘ

In the following table, the Atoms column lists the number of modelled atoms in the group and the number defined in the chemical component dictionary. The B-factors column lists the minimum, median, 95th percentile and maximum values of B factors of atoms in the group. The column labelled 'Q< 0.9' lists the number of atoms with occupancy less than 0.9.

Mol	Type	Chain	Res	Atoms	RSCC	RSR	B-factors(Å^2)	Q<0.9
2	NAG	B	2	14/15	0.77	0.27	57,69,87,90	0
2	NAG	B	1	14/15	0.95	0.13	37,53,65,65	0

The following is a graphical depiction of the model fit to experimental electron density for oligosaccharide. Each fit is shown from different orientation to approximate a three-dimensional view.

Electron density around Chain B:

2mF$_o$-DF$_c$ (at 0.7 rmsd) in gray
mF$_o$-DF$_c$ (at 3 rmsd) in purple (negative)
and green (positive)

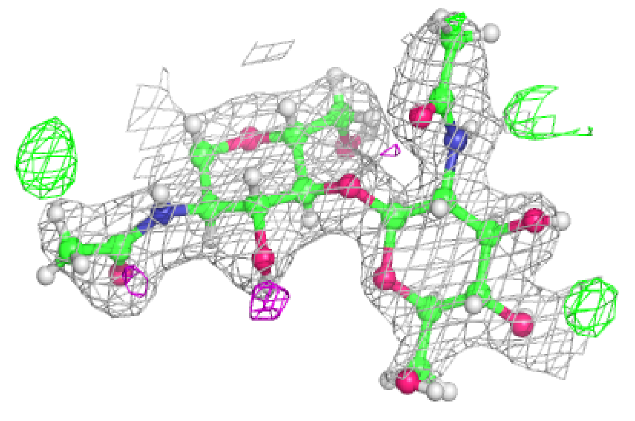

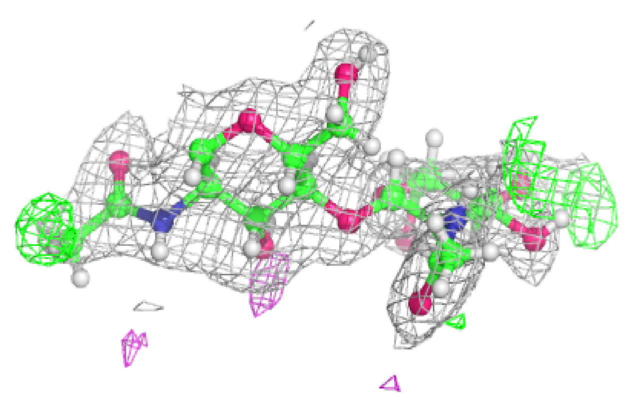

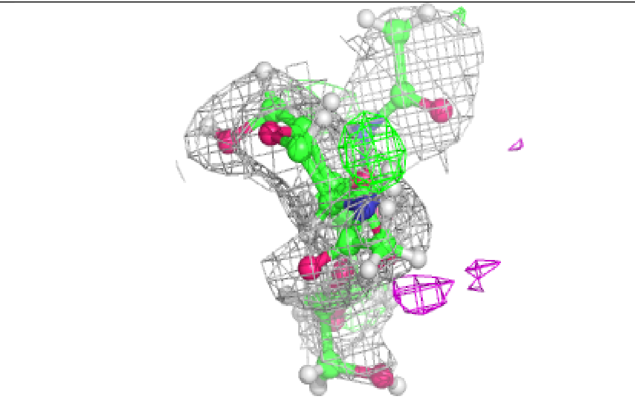

6.4 Ligands ⓘ

In the following table, the Atoms column lists the number of modelled atoms in the group and the number defined in the chemical component dictionary. The B-factors column lists the minimum,

median, 95th percentile and maximum values of B factors of atoms in the group. The column labelled 'Q< 0.9' lists the number of atoms with occupancy less than 0.9.

Mol	Type	Chain	Res	Atoms	RSCC	RSR	B-factors($Å^2$)	Q<0.9
9	EDO	A	623	4/4	0.73	0.16	53,65,73,78	0
5	PEG	A	613	7/7	0.78	0.26	70,84,89,90	0
4	NAG	A	605	14/15	0.78	0.18	66,79,92,94	0
5	PEG	A	614	7/7	0.79	0.13	59,71,76,78	0
5	PEG	A	612	7/7	0.81	0.20	59,70,84,86	0
4	NAG	A	606	14/15	0.81	0.23	54,67,80,83	28
10	IHS	A	625	36/36	0.82	0.15	95,116,139,146	0
6	PGE	A	617	10/10	0.83	0.27	60,75,90,92	0
9	EDO	A	624	4/4	0.83	0.12	71,85,90,91	0
6	PGE	A	618	10/10	0.84	0.13	66,81,92,92	0
6	PGE	A	616	10/10	0.86	0.14	67,80,88,90	0
8	PG4	A	620	13/13	0.86	0.11	58,72,87,88	0
5	PEG	A	610	7/7	0.87	0.15	62,74,86,89	0
9	EDO	A	621	4/4	0.88	0.18	69,83,84,85	0
5	PEG	A	611	7/7	0.89	0.21	66,79,80,80	0
9	EDO	A	622	4/4	0.89	0.16	66,80,80,81	0
6	PGE	A	615	10/10	0.90	0.12	51,64,78,78	0
3	FE	A	601	1/1	0.95	0.09	56,56,56,56	1
4	NAG	A	603	14/15	0.96	0.18	38,48,57,61	0
4	NAG	A	607	14/15	0.96	0.20	46,58,72,72	0
7	PO4	A	619	5/5	0.98	0.10	38,46,52,63	0
4	NAG	A	604	14/15	0.98	0.06	29,37,46,48	0
3	FE	A	602	1/1	1.00	0.13	20,20,20,20	1

The following is a graphical depiction of the model fit to experimental electron density of all instances of the Ligand of Interest. In addition, ligands with molecular weight > 250 and outliers as shown on the geometry validation Tables will also be included. Each fit is shown from different orientation to approximate a three-dimensional view.

Electron density around IHS A 625:

$2mF_o$-DF_c (at 0.7 rmsd) in gray
mF_o-DF_c (at 3 rmsd) in purple (negative)
and green (positive)

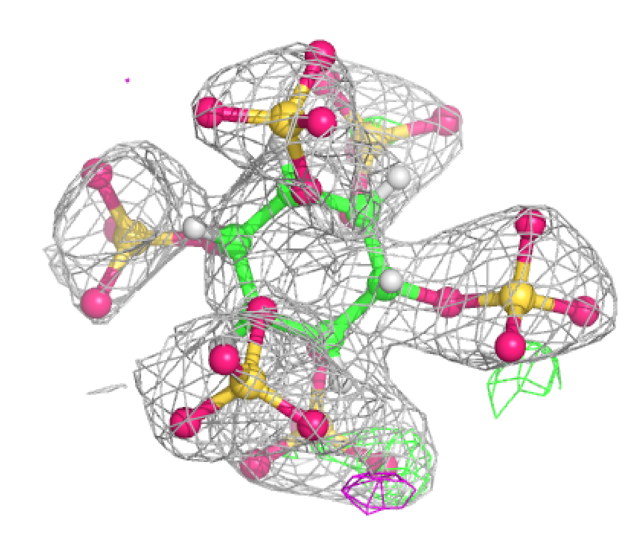

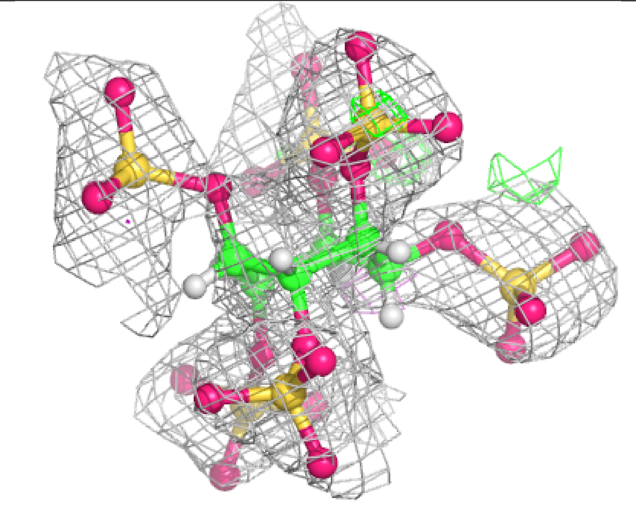

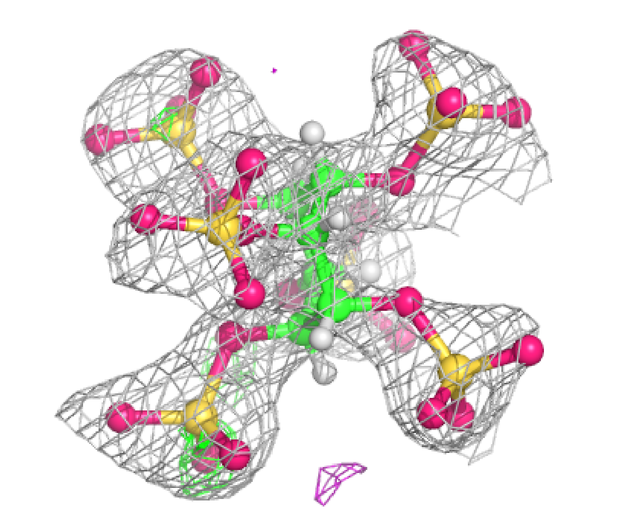

6.5 Other polymers ⓘ

There are no such residues in this entry.

WORLDWIDE PDB PROTEIN DATA BANK

Full wwPDB X-ray Structure Validation Report ⓘ

Aug 7, 2020 – 11:09 PM BST

PDB ID	:	6GJA
Title	:	PURPLE ACID PHYTASE FROM WHEAT ISOFORM B2 - H229A MUTANT
Authors	:	Faba-Rodriguez, R.; Brearley, C.A.; Hemmings, A.M.
Deposited on	:	2018-05-16
Resolution	:	1.50 Å(reported)

This is a Full wwPDB X-ray Structure Validation Report for a publicly released PDB entry.

We welcome your comments at *validation@mail.wwpdb.org*
A user guide is available at
https://www.wwpdb.org/validation/2017/XrayValidationReportHelp
with specific help available everywhere you see the ⓘ symbol.

The following versions of software and data (see references ⓘ) were used in the production of this report:

MolProbity	:	4.02b-467
Mogul	:	1.8.5 (274361), CSD as541be (2020)
Xtriage (Phenix)	:	1.13
EDS	:	2.13.1
buster-report	:	1.1.7 (2018)
Percentile statistics	:	20191225.v01 (using entries in the PDB archive December 25th 2019)
Refmac	:	5.8.0158
CCP4	:	7.0.044 (Gargrove)
Ideal geometry (proteins)	:	Engh & Huber (2001)
Ideal geometry (DNA, RNA)	:	Parkinson et al. (1996)
Validation Pipeline (wwPDB-VP)	:	2.13.1

1 Overall quality at a glance (i)

The following experimental techniques were used to determine the structure:
X-RAY DIFFRACTION

The reported resolution of this entry is 1.50 Å.

Percentile scores (ranging between 0-100) for global validation metrics of the entry are shown in the following graphic. The table shows the number of entries on which the scores are based.

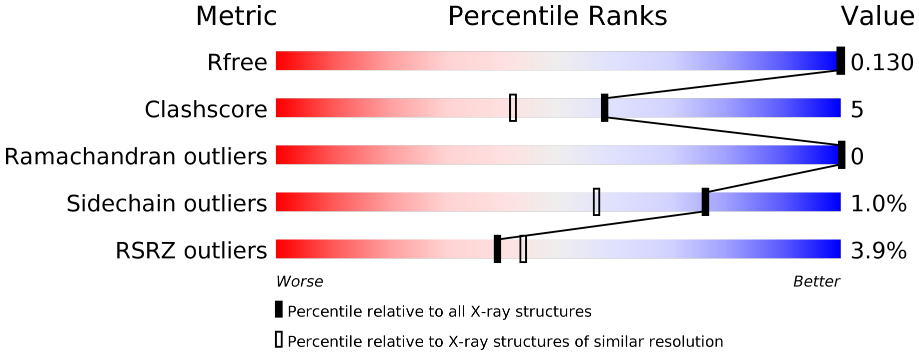

Metric	Whole archive (#Entries)	Similar resolution (#Entries, resolution range(Å))
R_{free}	130704	2936 (1.50-1.50)
Clashscore	141614	3144 (1.50-1.50)
Ramachandran outliers	138981	3066 (1.50-1.50)
Sidechain outliers	138945	3064 (1.50-1.50)
RSRZ outliers	127900	2884 (1.50-1.50)

The table below summarises the geometric issues observed across the polymeric chains and their fit to the electron density. The red, orange, yellow and green segments on the lower bar indicate the fraction of residues that contain outliers for >=3, 2, 1 and 0 types of geometric quality criteria respectively. A grey segment represents the fraction of residues that are not modelled. The numeric value for each fraction is indicated below the corresponding segment, with a dot representing fractions <=5% The upper red bar (where present) indicates the fraction of residues that have poor fit to the electron density. The numeric value is given above the bar.

Mol	Chain	Length	Quality of chain
1	A	516	4% 89% 6% • 5%
2	B	3	67% 33%

The following table lists non-polymeric compounds, carbohydrate monomers and non-standard residues in protein, DNA, RNA chains that are outliers for geometric or electron-density-fit criteria:

Mol	Type	Chain	Res	Chirality	Geometry	Clashes	Electron density
7	EDO	A	627	-	-	X	-

2 Entry composition

There are 10 unique types of molecules in this entry. The entry contains 4940 atoms, of which 285 are hydrogens and 0 are deuteriums.

In the tables below, the ZeroOcc column contains the number of atoms modelled with zero occupancy, the AltConf column contains the number of residues with at least one atom in alternate conformation and the Trace column contains the number of residues modelled with at most 2 atoms.

- Molecule 1 is a protein called Purple acid phosphatase.

Mol	Chain	Residues	Atoms					ZeroOcc	AltConf	Trace
			Total	C	N	O	S			
1	A	492	3957	2528	659	746	24	0	26	0

There are 7 discrepancies between the modelled and reference sequences:

Chain	Residue	Modelled	Actual	Comment	Reference
A	229	ALA	HIS	conflict	UNP C4PKL0
A	511	HIS	-	expression tag	UNP C4PKL0
A	512	HIS	-	expression tag	UNP C4PKL0
A	513	HIS	-	expression tag	UNP C4PKL0
A	514	HIS	-	expression tag	UNP C4PKL0
A	515	HIS	-	expression tag	UNP C4PKL0
A	516	HIS	-	expression tag	UNP C4PKL0

- Molecule 2 is an oligosaccharide called beta-D-mannopyranose-(1-4)-2-acetamido-2-deoxy-beta-D-glucopyranose-(1-4)-2-acetamido-2-deoxy-beta-D-glucopyranose.

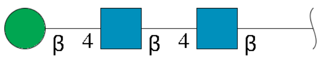

Mol	Chain	Residues	Atoms					ZeroOcc	AltConf	Trace
			Total	C	H	N	O			
2	B	3	74	22	35	2	15	0	0	0

- Molecule 3 is FE (III) ION (three-letter code: FE) (formula: Fe).

Mol	Chain	Residues	Atoms		ZeroOcc	AltConf
			Total	Fe		
3	A	2	2	2	0	0

- Molecule 4 is 2-acetamido-2-deoxy-beta-D-glucopyranose (three-letter code: NAG) (formula:

$C_8H_{15}NO_6$).

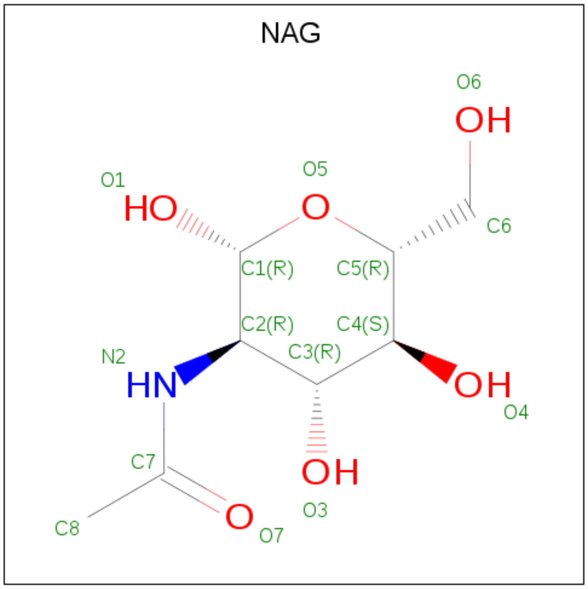

Mol	Chain	Residues	Atoms	ZeroOcc	AltConf
4	A	1	Total C H N O<br>28 8 14 1 5	0	0
4	A	1	Total C H N O<br>28 8 14 1 5	0	0
4	A	1	Total C H N O<br>28 8 14 1 5	0	0
4	A	1	Total C H N O<br>28 8 14 1 5	0	0
4	A	1	Total C H N O<br>28 8 14 1 5	0	0
4	A	1	Total C H N O<br>28 8 14 1 5	0	0

- Molecule 5 is PHOSPHATE ION (three-letter code: PO4) (formula: O_4P).

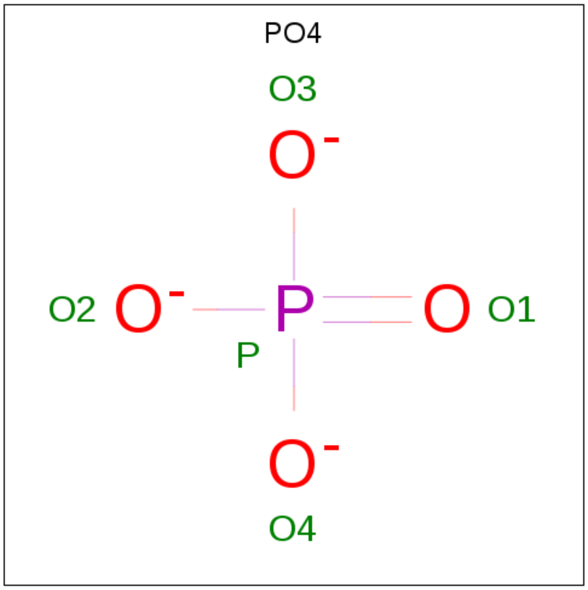

Mol	Chain	Residues	Atoms	ZeroOcc	AltConf
5	A	1	Total O P<br>5 4 1	0	0
5	A	1	Total O P<br>5 4 1	0	0
5	A	1	Total O P<br>5 4 1	0	0

- Molecule 6 is DI(HYDROXYETHYL)ETHER (three-letter code: PEG) (formula: $C_4H_{10}O_3$).

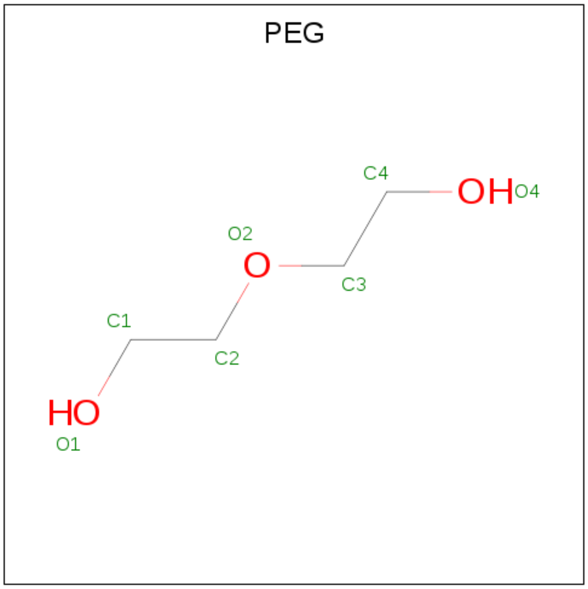

Mol	Chain	Residues	Atoms	ZeroOcc	AltConf
6	A	1	Total C H O<br>17 4 10 3	0	0

Continued on next page...

Continued from previous page...

Mol	Chain	Residues	Atoms	ZeroOcc	AltConf
6	A	1	Total C H O<br>17 4 10 3	0	0
6	A	1	Total C H O<br>17 4 10 3	0	0
6	A	1	Total C H O<br>17 4 10 3	0	0
6	A	1	Total C H O<br>17 4 10 3	0	0

- Molecule 7 is 1,2-ETHANEDIOL (three-letter code: EDO) (formula: $C_2H_6O_2$).

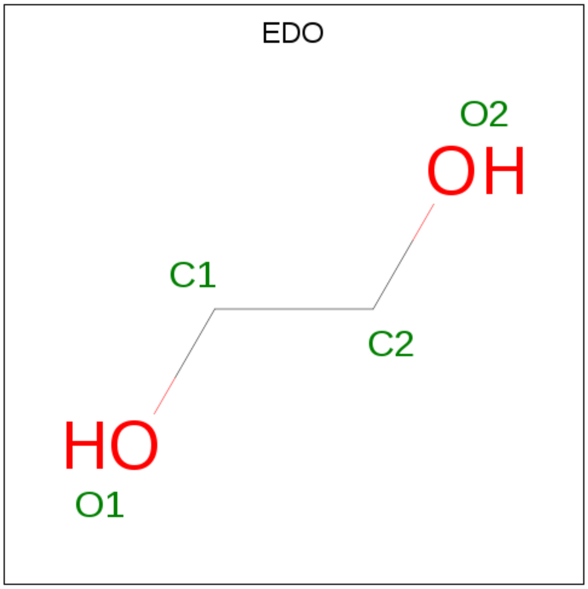

Mol	Chain	Residues	Atoms	ZeroOcc	AltConf
7	A	1	Total C H O<br>10 2 6 2	0	0
7	A	1	Total C H O<br>10 2 6 2	0	0
7	A	1	Total C H O<br>10 2 6 2	0	0
7	A	1	Total C H O<br>10 2 6 2	0	0
7	A	1	Total C H O<br>10 2 6 2	0	0
7	A	1	Total C H O<br>10 2 6 2	0	0
7	A	1	Total C H O<br>10 2 6 2	0	0

Continued on next page...

Continued from previous page...

Mol	Chain	Residues	Atoms				ZeroOcc	AltConf
			Total	C	H	O		
7	A	1	10	2	6	2	0	0

- Molecule 8 is TRIETHYLENE GLYCOL (three-letter code: PGE) (formula: $C_6H_{14}O_4$).

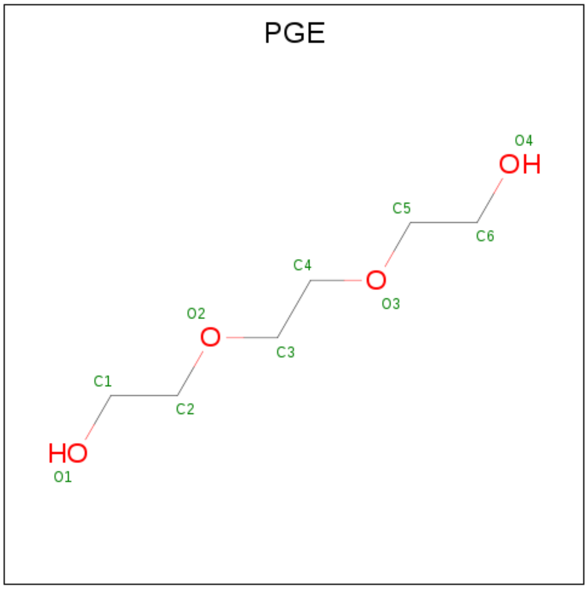

Mol	Chain	Residues	Atoms				ZeroOcc	AltConf
			Total	C	H	O		
8	A	1	24	6	14	4	0	0
8	A	1	24	6	14	4	0	0
8	A	1	24	6	14	4	0	0

- Molecule 9 is 1-(2-METHOXY-ETHOXY)-2-{2-[2-(2-METHOXY-ETHOXY]-ETHOXY}-ETHANE (three-letter code: PG6) (formula: $C_{12}H_{26}O_6$).

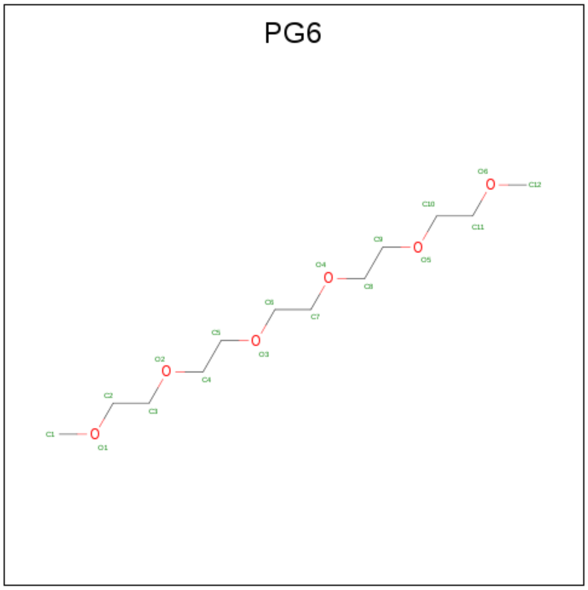

Mol	Chain	Residues	Atoms				ZeroOcc	AltConf
			Total	C	H	O		
9	A	1	44	12	26	6	0	0

- Molecule 10 is water.

Mol	Chain	Residues	Atoms		ZeroOcc	AltConf
			Total	O		
10	A	443	443	443	0	0

3 Residue-property plots (i)

These plots are drawn for all protein, RNA, DNA and oligosaccharide chains in the entry. The first graphic for a chain summarises the proportions of the various outlier classes displayed in the second graphic. The second graphic shows the sequence view annotated by issues in geometry and electron density. Residues are color-coded according to the number of geometric quality criteria for which they contain at least one outlier: green = 0, yellow = 1, orange = 2 and red = 3 or more. A red dot above a residue indicates a poor fit to the electron density (RSRZ > 2). Stretches of 2 or more consecutive residues without any outlier are shown as a green connector. Residues present in the sample, but not in the model, are shown in grey.

- Molecule 1: Purple acid phosphatase

Chain A:

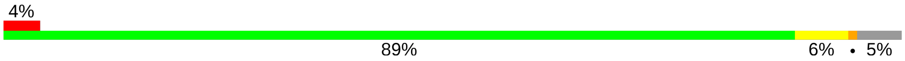

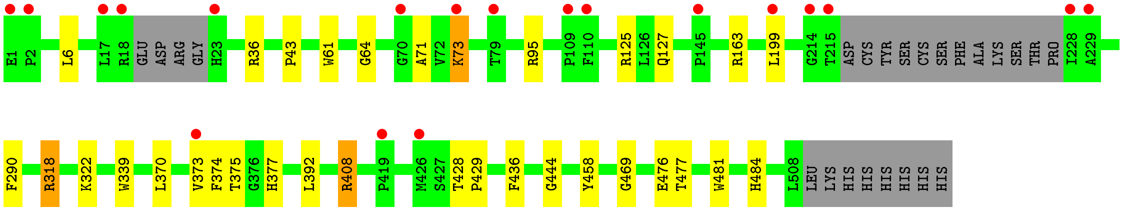

- Molecule 2: beta-D-mannopyranose-(1-4)-2-acetamido-2-deoxy-beta-D-glucopyranose-(1-4)-2-acetamido-2-deoxy-beta-D-glucopyranose

Chain B:

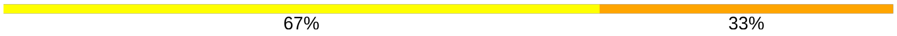

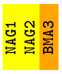

4 Data and refinement statistics (i)

Property	Value	Source
Space group	H 3	Depositor
Cell constants<br>a, b, c, α, β, γ	125.98Å 125.98Å 106.55Å<br>90.00° 90.00° 120.00°	Depositor
Resolution (Å)	36.37 – 1.50<br>36.37 – 1.50	Depositor<br>EDS
% Data completeness<br>(in resolution range)	96.5 (36.37-1.50)<br>96.5 (36.37-1.50)	Depositor<br>EDS
R_{merge}	0.06	Depositor
R_{sym}	(Not available)	Depositor
$< I/\sigma(I) >$ [1]	1.78 (at 1.50Å)	Xtriage
Refinement program	PHENIX	Depositor
R, R_{free}	0.128 , 0.152<br>0.130 , 0.130	Depositor<br>DCC
R_{free} test set	4921 reflections (5.05%)	wwPDB-VP
Wilson B-factor (Å^2)	16.0	Xtriage
Anisotropy	0.062	Xtriage
Bulk solvent k_{sol}(e/Å^3), B_{sol}(Å^2)	0.41 , 58.2	EDS
L-test for twinning[2]	$< \|L\| > = 0.51$, $< L^2 > = 0.34$	Xtriage
Estimated twinning fraction	0.015 for h,-h-k,-l	Xtriage
F_o,F_c correlation	0.98	EDS
Total number of atoms	4940	wwPDB-VP
Average B, all atoms (Å^2)	24.0	wwPDB-VP

Xtriage's analysis on translational NCS is as follows: *The largest off-origin peak in the Patterson function is 4.30% of the height of the origin peak. No significant pseudotranslation is detected.*

[1]Intensities estimated from amplitudes.

[2]Theoretical values of $< |L| >$, $< L^2 >$ for acentric reflections are 0.5, 0.333 respectively for untwinned datasets, and 0.375, 0.2 for perfectly twinned datasets.

5 Model quality (i)

5.1 Standard geometry (i)

Bond lengths and bond angles in the following residue types are not validated in this section: PGE, NAG, PO4, EDO, PG6, BMA, FE, PEG

The Z score for a bond length (or angle) is the number of standard deviations the observed value is removed from the expected value. A bond length (or angle) with $|Z| > 5$ is considered an outlier worth inspection. RMSZ is the root-mean-square of all Z scores of the bond lengths (or angles).

Mol	Chain	Bond lengths		Bond angles	
		RMSZ	#$|Z|$ >5	RMSZ	#$|Z|$ >5
1	A	0.33	0/4157	0.53	0/5668

There are no bond length outliers.

There are no bond angle outliers.

There are no chirality outliers.

There are no planarity outliers.

5.2 Too-close contacts (i)

In the following table, the Non-H and H(model) columns list the number of non-hydrogen atoms and hydrogen atoms in the chain respectively. The H(added) column lists the number of hydrogen atoms added and optimized by MolProbity. The Clashes column lists the number of clashes within the asymmetric unit, whereas Symm-Clashes lists symmetry related clashes.

Mol	Chain	Non-H	H(model)	H(added)	Clashes	Symm-Clashes
1	A	3957	0	3780	39	0
2	B	39	35	34	1	0
3	A	2	0	0	0	0
4	A	84	84	78	0	0
5	A	15	0	0	0	0
6	A	35	50	50	2	0
7	A	32	48	48	8	0
8	A	30	42	42	6	0
9	A	18	26	26	2	0
10	A	443	0	0	6	0
All	All	4655	285	4058	40	0

The all-atom clashscore is defined as the number of clashes found per 1000 atoms (including hydrogen atoms). The all-atom clashscore for this structure is 5.

All (40) close contacts within the same asymmetric unit are listed below, sorted by their clash magnitude.

Atom-1	Atom-2	Interatomic distance (Å)	Clash overlap (Å)
1:A:163:ARG:HH22	8:A:629:PGE:H32	1.47	0.79
1:A:444:GLY:H	9:A:631:PG6:H13	1.50	0.75
1:A:318:ARG:HD2	7:A:621:EDO:H22	1.70	0.73
1:A:322:LYS:HE3	7:A:627:EDO:O2	1.89	0.71
1:A:163:ARG:HH12	8:A:629:PGE:H3	1.58	0.69
1:A:6:LEU:O	10:A:702:HOH:O	2.13	0.67
1:A:64:GLY:HA2	6:A:617:PEG:H31	1.78	0.66
1:A:408[A]:ARG:HD3	10:A:741:HOH:O	1.98	0.63
1:A:322:LYS:HG2	7:A:627:EDO:H21	1.80	0.62
1:A:444:GLY:N	9:A:631:PG6:H32	2.13	0.62
1:A:199[B]:LEU:HD23	1:A:375:THR:CA	2.30	0.61
1:A:95:ARG:HE	7:A:622:EDO:H22	1.65	0.61
1:A:322:LYS:HE3	7:A:627:EDO:C2	2.32	0.60
1:A:199[B]:LEU:HD21	1:A:374:PHE:HB3	1.82	0.60
1:A:429:PRO:HG3	1:A:436:PHE:HD1	1.66	0.59
1:A:199[B]:LEU:HD23	1:A:375:THR:C	2.23	0.59
1:A:322:LYS:HG2	7:A:627:EDO:C2	2.36	0.55
1:A:125[A]:ARG:HH22	8:A:630:PGE:C1	2.18	0.54
1:A:163:ARG:HH22	8:A:629:PGE:C3	2.21	0.53
1:A:484:HIS:HE1	7:A:625:EDO:H12	1.76	0.51
1:A:429:PRO:CG	1:A:436:PHE:HD1	2.23	0.51
1:A:377:HIS:HA	10:A:704:HOH:O	2.11	0.50
1:A:71:ALA:O	1:A:73:LYS:HD2	2.12	0.50
1:A:127[A]:GLN:HG2	10:A:815:HOH:O	2.14	0.48
1:A:125[A]:ARG:HH22	8:A:630:PGE:H1	1.78	0.47
1:A:469:GLY:HA3	1:A:481:TRP:CH2	2.49	0.47
1:A:290:PHE:HZ	6:A:618:PEG:H32	1.79	0.47
1:A:428:THR:N	1:A:429:PRO:CD	2.79	0.46
1:A:476:GLU:HG2	1:A:477:THR:HG23	1.98	0.46
1:A:370:LEU:HD21	1:A:373:VAL:HG22	1.99	0.45
1:A:429:PRO:HG3	1:A:436:PHE:CD1	2.50	0.44
1:A:125[A]:ARG:HH22	8:A:630:PGE:H12	1.80	0.44
1:A:318:ARG:HD3	10:A:707:HOH:O	2.17	0.44
1:A:339:TRP:O	1:A:375:THR:HA	2.18	0.43
10:A:703:HOH:O	2:B:3:BMA:O3	2.20	0.43
1:A:322:LYS:HE3	7:A:627:EDO:H22	2.01	0.42
1:A:199[A]:LEU:HD11	1:A:374:PHE:HB3	2.00	0.42
1:A:392:LEU:HD21	1:A:458:TYR:HA	2.02	0.42
1:A:428:THR:N	1:A:429:PRO:HD3	2.35	0.41
1:A:43:PRO:HB3	1:A:61:TRP:CD1	2.57	0.40

There are no symmetry-related clashes.

5.3 Torsion angles (i)

5.3.1 Protein backbone (i)

In the following table, the Percentiles column shows the percent Ramachandran outliers of the chain as a percentile score with respect to all X-ray entries followed by that with respect to entries of similar resolution.

The Analysed column shows the number of residues for which the backbone conformation was analysed, and the total number of residues.

Mol	Chain	Analysed	Favoured	Allowed	Outliers	Percentiles	
1	A	513/516 (99%)	497 (97%)	16 (3%)	0	100	100

There are no Ramachandran outliers to report.

5.3.2 Protein sidechains (i)

In the following table, the Percentiles column shows the percent sidechain outliers of the chain as a percentile score with respect to all X-ray entries followed by that with respect to entries of similar resolution.

The Analysed column shows the number of residues for which the sidechain conformation was analysed, and the total number of residues.

Mol	Chain	Analysed	Rotameric	Outliers	Percentiles	
1	A	424/424 (100%)	419 (99%)	5 (1%)	71	48

All (5) residues with a non-rotameric sidechain are listed below:

Mol	Chain	Res	Type
1	A	36	ARG
1	A	73	LYS
1	A	318	ARG
1	A	408[A]	ARG
1	A	408[B]	ARG

Some sidechains can be flipped to improve hydrogen bonding and reduce clashes. There are no such sidechains identified.

5.3.3 RNA (i)

There are no RNA molecules in this entry.

5.4 Non-standard residues in protein, DNA, RNA chains (i)

There are no non-standard protein/DNA/RNA residues in this entry.

5.5 Carbohydrates (i)

3 monosaccharides are modelled in this entry.

In the following table, the Counts columns list the number of bonds (or angles) for which Mogul statistics could be retrieved, the number of bonds (or angles) that are observed in the model and the number of bonds (or angles) that are defined in the Chemical Component Dictionary. The Link column lists molecule types, if any, to which the group is linked. The Z score for a bond length (or angle) is the number of standard deviations the observed value is removed from the expected value. A bond length (or angle) with $|Z| > 2$ is considered an outlier worth inspection. RMSZ is the root-mean-square of all Z scores of the bond lengths (or angles).

Mol	Type	Chain	Res	Link	Bond lengths			Bond angles		
					Counts	RMSZ	$\#\|Z\| > 2$	Counts	RMSZ	$\#\|Z\| > 2$
2	NAG	B	1	1,2	14,14,15	1.78	3 (21%)	17,19,21	0.98	1 (5%)
2	NAG	B	2	2	14,14,15	1.88	3 (21%)	17,19,21	1.02	1 (5%)
2	BMA	B	3	2	11,11,12	1.75	2 (18%)	15,15,17	0.77	1 (6%)

In the following table, the Chirals column lists the number of chiral outliers, the number of chiral centers analysed, the number of these observed in the model and the number defined in the Chemical Component Dictionary. Similar counts are reported in the Torsion and Rings columns. '-' means no outliers of that kind were identified.

Mol	Type	Chain	Res	Link	Chirals	Torsions	Rings
2	NAG	B	1	1,2	-	0/6/23/26	0/1/1/1
2	NAG	B	2	2	-	0/6/23/26	0/1/1/1
2	BMA	B	3	2	-	0/2/19/22	0/1/1/1

All (8) bond length outliers are listed below:

Mol	Chain	Res	Type	Atoms	Z	Observed(Å)	Ideal(Å)
2	B	3	BMA	O5-C1	4.61	1.51	1.43
2	B	2	NAG	O5-C1	4.31	1.50	1.43
2	B	1	NAG	O5-C1	3.93	1.50	1.43
2	B	2	NAG	C7-N2	3.48	1.46	1.34

Continued on next page...

Continued from previous page...

Mol	Chain	Res	Type	Atoms	Z	Observed(Å)	Ideal(Å)
2	B	1	NAG	C7-N2	3.45	1.46	1.34
2	B	3	BMA	C2-C3	-2.37	1.49	1.52
2	B	2	NAG	C2-N2	2.28	1.50	1.46
2	B	1	NAG	C2-N2	2.26	1.50	1.46

All (3) bond angle outliers are listed below:

Mol	Chain	Res	Type	Atoms	Z	Observed(^{o})	Ideal(^{o})
2	B	1	NAG	C8-C7-N2	2.22	119.86	116.10
2	B	2	NAG	C8-C7-N2	2.15	119.74	116.10
2	B	3	BMA	C1-C2-C3	2.09	112.24	109.67

There are no chirality outliers.

There are no torsion outliers.

There are no ring outliers.

1 monomer is involved in 1 short contact:

Mol	Chain	Res	Type	Clashes	Symm-Clashes
2	B	3	BMA	1	0

The following is a two-dimensional graphical depiction of Mogul quality analysis of bond lengths, bond angles, torsion angles, and ring geometry for oligosaccharide.

Oligosaccharide Chain B

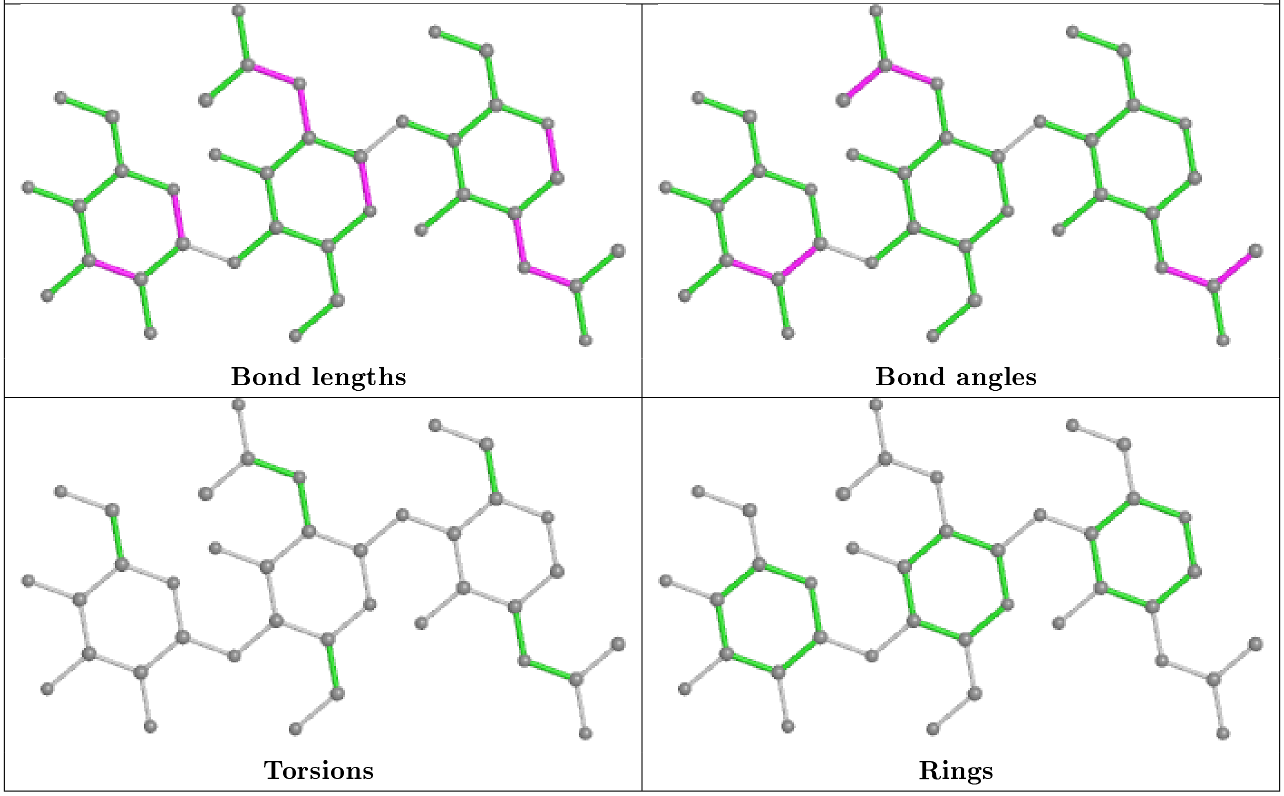

5.6 Ligand geometry (i)

Of 28 ligands modelled in this entry, 2 are monoatomic - leaving 26 for Mogul analysis.

In the following table, the Counts columns list the number of bonds (or angles) for which Mogul statistics could be retrieved, the number of bonds (or angles) that are observed in the model and the number of bonds (or angles) that are defined in the Chemical Component Dictionary. The Link column lists molecule types, if any, to which the group is linked. The Z score for a bond length (or angle) is the number of standard deviations the observed value is removed from the expected value. A bond length (or angle) with $|Z| > 2$ is considered an outlier worth inspection. RMSZ is the root-mean-square of all Z scores of the bond lengths (or angles).

Mol	Type	Chain	Res	Link	Bond lengths			Bond angles		
					Counts	RMSZ	$\#\|Z\| > 2$	Counts	RMSZ	$\#\|Z\| > 2$
6	PEG	A	619	-	6,6,6	0.48	0	5,5,5	0.61	0
4	NAG	A	606	-	14,14,15	1.87	3 (21%)	17,19,21	1.05	1 (5%)
8	PGE	A	629	-	9,9,9	0.50	0	8,8,8	0.58	0
5	PO4	A	612	3	4,4,4	0.98	0	6,6,6	0.29	0
7	EDO	A	626	-	3,3,3	0.46	0	2,2,2	0.30	0
4	NAG	A	603	1	14,14,15	1.76	2 (14%)	17,19,21	1.12	1 (5%)
7	EDO	A	622	-	3,3,3	0.47	0	2,2,2	0.34	0
8	PGE	A	630	-	9,9,9	0.52	0	8,8,8	0.35	0
4	NAG	A	604	1	14,14,15	1.59	2 (14%)	17,19,21	1.00	1 (5%)

Mol	Type	Chain	Res	Link	Bond lengths			Bond angles		
					Counts	RMSZ	#|Z| > 2	Counts	RMSZ	#|Z| > 2
4	NAG	A	605	1	14,14,15	1.91	3 (21%)	17,19,21	1.17	2 (11%)
7	EDO	A	620	-	3,3,3	0.47	0	2,2,2	0.35	0
7	EDO	A	621	-	3,3,3	0.49	0	2,2,2	0.37	0
4	NAG	A	608	1	14,14,15	1.77	3 (21%)	17,19,21	1.09	2 (11%)
9	PG6	A	631	-	17,17,17	0.52	0	16,16,16	0.47	0
4	NAG	A	607	1	14,14,15	1.91	3 (21%)	17,19,21	1.13	1 (5%)
7	EDO	A	623	-	3,3,3	0.47	0	2,2,2	0.26	0
6	PEG	A	617	-	6,6,6	0.51	0	5,5,5	0.53	0
8	PGE	A	628	-	9,9,9	0.51	0	8,8,8	0.24	0
6	PEG	A	618	-	6,6,6	0.47	0	5,5,5	0.60	0
7	EDO	A	625	-	3,3,3	0.47	0	2,2,2	0.37	0
6	PEG	A	615	-	6,6,6	0.49	0	5,5,5	0.40	0
5	PO4	A	613	-	4,4,4	0.95	0	6,6,6	0.41	0
7	EDO	A	627	-	3,3,3	0.46	0	2,2,2	0.23	0
6	PEG	A	616	-	6,6,6	0.50	0	5,5,5	0.25	0
5	PO4	A	614	-	4,4,4	0.91	0	6,6,6	0.41	0
7	EDO	A	624	-	3,3,3	0.48	0	2,2,2	0.24	0

In the following table, the Chirals column lists the number of chiral outliers, the number of chiral centers analysed, the number of these observed in the model and the number defined in the Chemical Component Dictionary. Similar counts are reported in the Torsion and Rings columns. '-' means no outliers of that kind were identified.

Mol	Type	Chain	Res	Link	Chirals	Torsions	Rings
6	PEG	A	619	-	-	1/4/4/4	-
4	NAG	A	606	-	-	0/6/23/26	0/1/1/1
8	PGE	A	629	-	-	1/7/7/7	-
7	EDO	A	626	-	-	0/1/1/1	-
4	NAG	A	603	1	-	0/6/23/26	0/1/1/1
7	EDO	A	622	-	-	0/1/1/1	-
8	PGE	A	630	-	-	1/7/7/7	-
4	NAG	A	604	1	-	0/6/23/26	0/1/1/1
4	NAG	A	605	1	-	0/6/23/26	0/1/1/1
7	EDO	A	620	-	-	0/1/1/1	-
7	EDO	A	621	-	-	0/1/1/1	-
4	NAG	A	608	1	-	0/6/23/26	0/1/1/1
9	PG6	A	631	-	-	8/15/15/15	-
4	NAG	A	607	1	-	4/6/23/26	0/1/1/1
7	EDO	A	623	-	-	0/1/1/1	-
6	PEG	A	617	-	-	2/4/4/4	-
8	PGE	A	628	-	-	0/7/7/7	-

Continued on next page...

Continued from previous page...

Mol	Type	Chain	Res	Link	Chirals	Torsions	Rings
6	PEG	A	618	-	-	2/4/4/4	-
7	EDO	A	625	-	-	1/1/1/1	-
6	PEG	A	615	-	-	1/4/4/4	-
7	EDO	A	627	-	-	1/1/1/1	-
6	PEG	A	616	-	-	0/4/4/4	-
7	EDO	A	624	-	-	0/1/1/1	-

All (16) bond length outliers are listed below:

Mol	Chain	Res	Type	Atoms	Z	Observed(Å)	Ideal(Å)
4	A	605	NAG	O5-C1	4.44	1.50	1.43
4	A	607	NAG	O5-C1	4.27	1.50	1.43
4	A	606	NAG	O5-C1	4.21	1.50	1.43
4	A	603	NAG	O5-C1	3.89	1.49	1.43
4	A	608	NAG	O5-C1	3.79	1.49	1.43
4	A	607	NAG	C7-N2	3.60	1.46	1.34
4	A	606	NAG	C7-N2	3.52	1.46	1.34
4	A	605	NAG	C7-N2	3.46	1.46	1.34
4	A	604	NAG	O5-C1	3.45	1.49	1.43
4	A	608	NAG	C7-N2	3.45	1.46	1.34
4	A	603	NAG	C7-N2	3.37	1.46	1.34
4	A	604	NAG	C7-N2	3.12	1.45	1.34
4	A	607	NAG	C2-N2	2.50	1.50	1.46
4	A	606	NAG	C2-N2	2.17	1.50	1.46
4	A	605	NAG	C2-N2	2.17	1.50	1.46
4	A	608	NAG	C2-N2	2.09	1.49	1.46

All (8) bond angle outliers are listed below:

Mol	Chain	Res	Type	Atoms	Z	Observed(°)	Ideal(°)
4	A	607	NAG	C8-C7-N2	2.92	121.05	116.10
4	A	608	NAG	C2-N2-C7	-2.66	119.12	122.90
4	A	604	NAG	O5-C1-C2	-2.63	107.14	111.29
4	A	605	NAG	C2-N2-C7	-2.57	119.24	122.90
4	A	606	NAG	C1-C2-N2	-2.42	106.36	110.49
4	A	608	NAG	C8-C7-N2	2.39	120.14	116.10
4	A	605	NAG	C8-C7-N2	2.12	119.69	116.10
4	A	603	NAG	C2-N2-C7	-2.08	119.94	122.90

There are no chirality outliers.

All (22) torsion outliers are listed below:

Mol	Chain	Res	Type	Atoms
4	A	607	NAG	C8-C7-N2-C2
4	A	607	NAG	O7-C7-N2-C2
9	A	631	PG6	C9-C8-O4-C7
6	A	617	PEG	O1-C1-C2-O2
6	A	617	PEG	C4-C3-O2-C2
8	A	629	PGE	O1-C1-C2-O2
4	A	607	NAG	C4-C5-C6-O6
4	A	607	NAG	O5-C5-C6-O6
6	A	615	PEG	O1-C1-C2-O2
7	A	625	EDO	O1-C1-C2-O2
9	A	631	PG6	C5-C4-O2-C3
6	A	618	PEG	C4-C3-O2-C2
6	A	618	PEG	C1-C2-O2-C3
9	A	631	PG6	C10-C11-O6-C12
6	A	619	PEG	C4-C3-O2-C2
7	A	627	EDO	O1-C1-C2-O2
8	A	630	PGE	C4-C3-O2-C2
9	A	631	PG6	C11-C10-O5-C9
9	A	631	PG6	O1-C2-C3-O2
9	A	631	PG6	C3-C2-O1-C1
9	A	631	PG6	O5-C10-C11-O6
9	A	631	PG6	O2-C4-C5-O3

There are no ring outliers.

9 monomers are involved in 18 short contacts:

Mol	Chain	Res	Type	Clashes	Symm-Clashes
8	A	629	PGE	3	0
7	A	622	EDO	1	0
8	A	630	PGE	3	0
7	A	621	EDO	1	0
9	A	631	PG6	2	0
6	A	617	PEG	1	0
6	A	618	PEG	1	0
7	A	625	EDO	1	0
7	A	627	EDO	5	0

The following is a two-dimensional graphical depiction of Mogul quality analysis of bond lengths, bond angles, torsion angles, and ring geometry for all instances of the Ligand of Interest. In addition, ligands with molecular weight > 250 and outliers as shown on the validation Tables will also be included. For torsion angles, if less then 5% of the Mogul distribution of torsion angles is within 10 degrees of the torsion angle in question, then that torsion angle is considered an outlier. Any bond that is central to one or more torsion angles identified as an outlier by Mogul will be

highlighted in the graph. For rings, the root-mean-square deviation (RMSD) between the ring in question and similar rings identified by Mogul is calculated over all ring torsion angles. If the average RMSD is greater than 60 degrees and the minimal RMSD between the ring in question and any Mogul-identified rings is also greater than 60 degrees, then that ring is considered an outlier. The outliers are highlighted in purple. The color gray indicates Mogul did not find sufficient equivalents in the CSD to analyse the geometry.

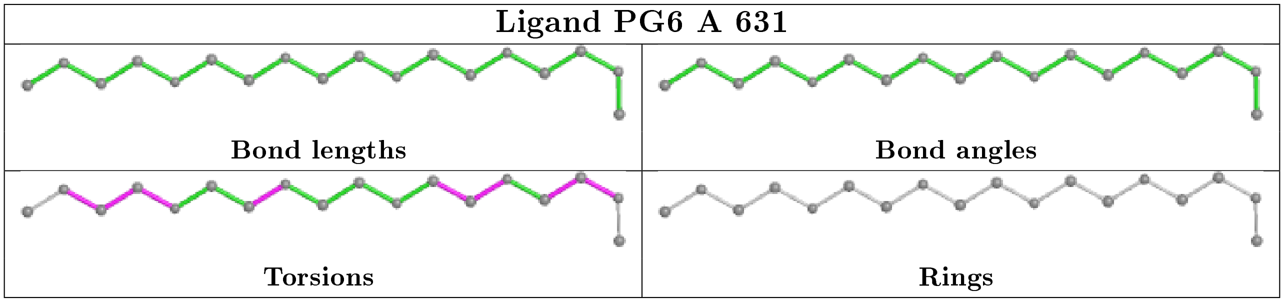

5.7 Other polymers (i)

There are no such residues in this entry.

5.8 Polymer linkage issues (i)

There are no chain breaks in this entry.

6 Fit of model and data (i)

6.1 Protein, DNA and RNA chains (i)

In the following table, the column labelled '#RSRZ> 2' contains the number (and percentage) of RSRZ outliers, followed by percent RSRZ outliers for the chain as percentile scores relative to all X-ray entries and entries of similar resolution. The OWAB column contains the minimum, median, 95th percentile and maximum values of the occupancy-weighted average B-factor per residue. The column labelled 'Q< 0.9' lists the number of (and percentage) of residues with an average occupancy less than 0.9.

Mol	Chain	Analysed	<RSRZ>	#RSRZ>2			OWAB(Å^2)	Q<0.9
1	A	492/516 (95%)	0.09	19 (3%)	39	44	12, 17, 34, 59	0

All (19) RSRZ outliers are listed below:

Mol	Chain	Res	Type	RSRZ
1	A	17	LEU	6.3
1	A	215	THR	5.0
1	A	2	PRO	5.0
1	A	228	ILE	4.8
1	A	110	PHE	3.9
1	A	229	ALA	3.5
1	A	109	PRO	3.3
1	A	214	GLY	2.7
1	A	1	GLU	2.7
1	A	70	GLY	2.5
1	A	79	THR	2.5
1	A	23	HIS	2.4
1	A	145	PRO	2.4
1	A	18	ARG	2.3
1	A	199[A]	LEU	2.3
1	A	73	LYS	2.3
1	A	419	PRO	2.2
1	A	373	VAL	2.1
1	A	426	MET	2.1

6.2 Non-standard residues in protein, DNA, RNA chains (i)

There are no non-standard protein/DNA/RNA residues in this entry.

6.3 Carbohydrates (i)

In the following table, the Atoms column lists the number of modelled atoms in the group and the number defined in the chemical component dictionary. The B-factors column lists the minimum, median, 95th percentile and maximum values of B factors of atoms in the group. The column labelled 'Q< 0.9' lists the number of atoms with occupancy less than 0.9.

Mol	Type	Chain	Res	Atoms	RSCC	RSR	B-factors($Å^2$)	Q<0.9
2	BMA	B	3	11/12	0.91	0.30	43,49,58,59	0
2	NAG	B	2	14/15	0.92	0.22	27,34,45,48	0
2	NAG	B	1	14/15	0.97	0.11	19,28,39,39	0

The following is a graphical depiction of the model fit to experimental electron density for oligosaccharide. Each fit is shown from different orientation to approximate a three-dimensional view.

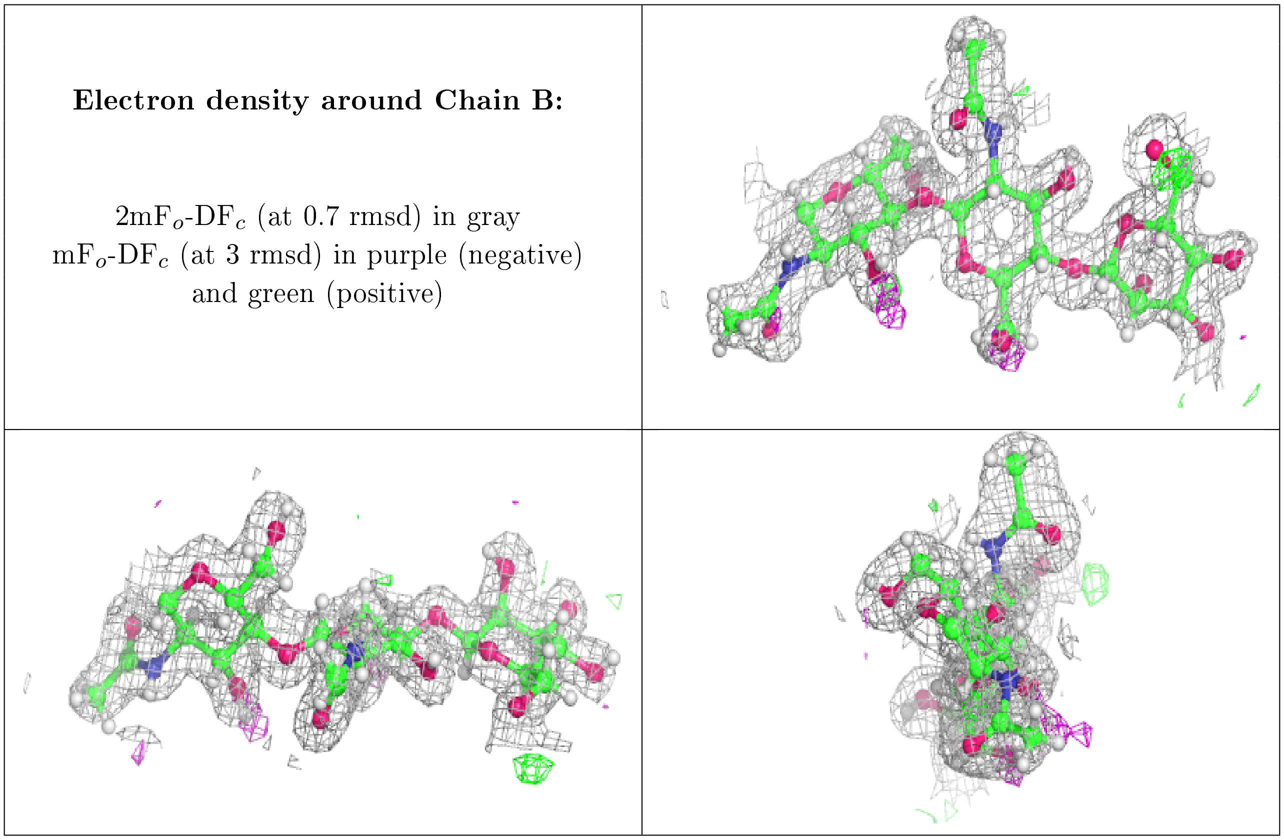

6.4 Ligands (i)

In the following table, the Atoms column lists the number of modelled atoms in the group and the number defined in the chemical component dictionary. The B-factors column lists the minimum, median, 95th percentile and maximum values of B factors of atoms in the group. The column labelled 'Q< 0.9' lists the number of atoms with occupancy less than 0.9.

Mol	Type	Chain	Res	Atoms	RSCC	RSR	B-factors(Å^2)	Q<0.9
4	NAG	A	606	14/15	0.72	0.21	50,56,66,67	28
7	EDO	A	624	4/4	0.75	0.27	62,75,75,76	0
5	PO4	A	614	5/5	0.77	0.25	77,77,77,78	5
8	PGE	A	629	10/10	0.78	0.19	51,62,73,73	0
6	PEG	A	617	7/7	0.81	0.15	49,59,64,64	0
6	PEG	A	619	7/7	0.82	0.22	47,57,63,63	0
7	EDO	A	627	4/4	0.82	0.27	39,49,58,58	0
4	NAG	A	607	14/15	0.83	0.17	30,38,44,46	28
6	PEG	A	618	7/7	0.85	0.17	53,64,69,70	0
9	PG6	A	631	18/18	0.85	0.17	36,48,60,60	0
7	EDO	A	622	4/4	0.86	0.14	55,66,67,68	0
7	EDO	A	625	4/4	0.86	0.20	58,70,70,71	0
5	PO4	A	613	5/5	0.86	0.23	63,65,66,67	5
8	PGE	A	630	10/10	0.87	0.15	53,64,69,71	0
8	PGE	A	628	10/10	0.89	0.21	56,68,74,75	0
6	PEG	A	615	7/7	0.89	0.12	49,59,60,61	0
4	NAG	A	605	14/15	0.89	0.24	45,56,67,70	0
7	EDO	A	623	4/4	0.92	0.22	54,65,66,68	0
6	PEG	A	616	7/7	0.92	0.19	40,51,64,66	0
7	EDO	A	621	4/4	0.94	0.22	43,51,55,57	0
7	EDO	A	620	4/4	0.94	0.10	33,47,54,58	0
7	EDO	A	626	4/4	0.95	0.13	42,51,52,53	0
4	NAG	A	603	14/15	0.96	0.20	26,35,41,42	0
4	NAG	A	608	14/15	0.97	0.17	27,35,50,50	0
5	PO4	A	612	5/5	0.97	0.08	22,22,25,25	5
4	NAG	A	604	14/15	0.98	0.08	16,21,26,30	0
3	FE	A	601	1/1	1.00	0.05	20,20,20,20	1
3	FE	A	602	1/1	1.00	0.07	14,14,14,14	0

The following is a graphical depiction of the model fit to experimental electron density of all instances of the Ligand of Interest. In addition, ligands with molecular weight > 250 and outliers as shown on the geometry validation Tables will also be included. Each fit is shown from different orientation to approximate a three-dimensional view.

Electron density around PG6 A 631:

2mF$_o$-DF$_c$ (at 0.7 rmsd) in gray
mF$_o$-DF$_c$ (at 3 rmsd) in purple (negative)
and green (positive)

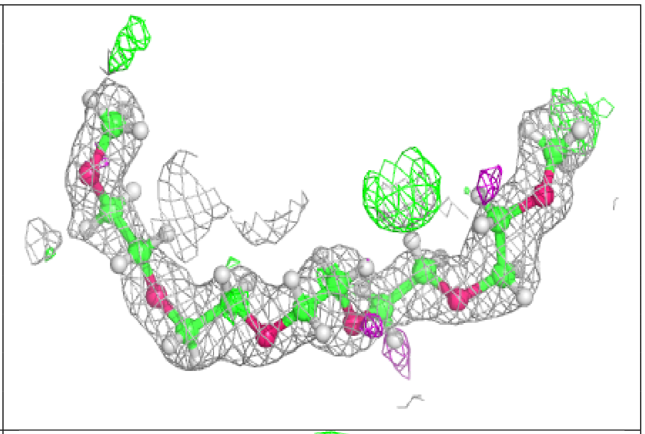

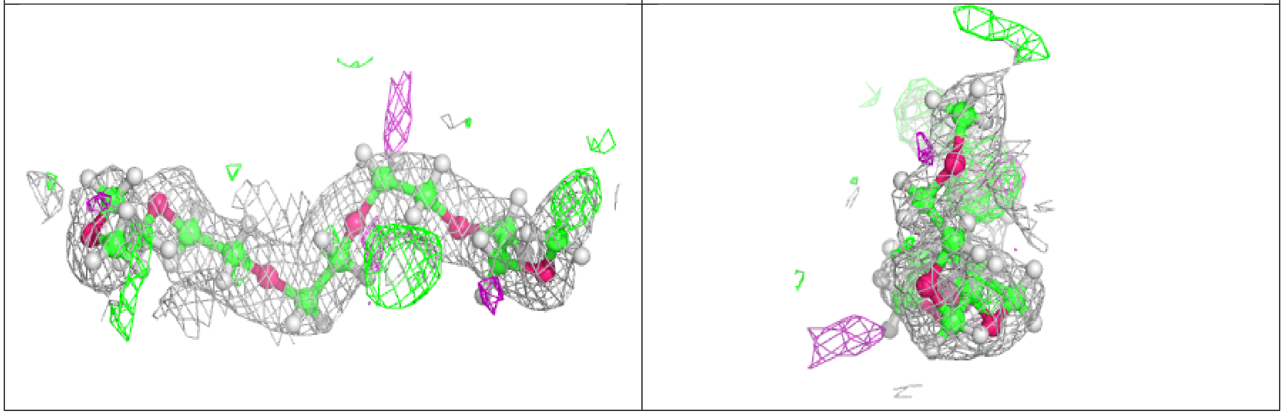

6.5 Other polymers ⓘ

There are no such residues in this entry.
