# Supplementary material for: Structure of a cereal purple acid phytase provides new insights to phytate degradation in plants
Source: Plant Commun. 2022 Feb 19;3(2):100305. doi: 10.1016/j.xplc.2022.100305 (PMC9073318; doi:10.1016/j.xplc.2022.100305)
Supplement: Document S2. Article plus supplemental information [file mmc2.pdf]

Plant Communications
Research Article

CellPress
Partner Journal

Structure of a cereal purple acid phytase provides new insights to phytate degradation in plants

Raquel Faba-Rodriguez[1,2], Yinghong Gu[2], Melissa Salmon[2], Giuseppe Dionisio[3], Henrik Brinch-Pedersen[3], Charles A. Brearley[2] and Andrew M. Hemmings[1,2,4,*]

[1]School of Chemistry, University of East Anglia, Norwich Research Park, Norwich NR4 7TJ, UK

[2]School of Biological Sciences, University of East Anglia, Norwich Research Park, Norwich NR4 7TJ, UK

[3]Department of Agroecology, Research Center Flakkebjerg, Aarhus University, 4200 Slagelse, Denmark

[4]College of Food Science and Technology, Shanghai Ocean University, Shanghai 201306, China

***Correspondence: Andrew M. Hemmings (a.hemmings@uea.ac.uk)**

https://doi.org/10.1016/j.xplc.2022.100305

ABSTRACT

Grain phytate, a mixed metal ion salt of inositol hexakisphosphate, accounts for 60%–80% of stored phosphorus in plants and is a potent antinutrient of non-ruminant animals including humans. Through neo-functionalization of purple acid phytases (PAPhy), some cereals such as wheat and rye have acquired particularly high mature grain phytase activity. As PAPhy activity supplies phosphate, liberates metal ions necessary for seedling emergence, and obviates antinutrient effects of phytate, its manipulation and control are targeted crop traits. Here we show the X-ray crystal structure of the b2 isoform of wheat PAPhy induced during germination. This high-resolution crystal structure suggests a model for phytate recognition that, validated by molecular dynamics simulations, implicates elements of two sequence inserts (termed PAPhy motifs) relative to a canonical metallophosphoesterase (MPE) domain in forming phytate-specific substrate specificity pockets. These motifs are well conserved in PAPhys from monocot cereals, enzymes which are characterized by high specificity for phytate. Tested by mutagenesis, residues His229 in PAPhy motif 4 and Lys410 in the MPE domain, both conserved in PAPhys, are found to strongly influence phytase activity. These results explain the observed phytase activity of cereal PAPhys and open the way to the rational engineering of phytase activity *in planta*.

Key words: wheat, purple acid phytase, X-ray crystallography, stereospecificity

Faba-Rodriguez R., Gu Y., Salmon M., Dionisio G., Brinch-Pedersen H., Brearley C.A., and Hemmings A.M. (2022). Structure of a cereal purple acid phytase provides new insights to phytate degradation in plants. Plant Comm. **3**, 100305.

INTRODUCTION

Among other nutrients, seeds must accumulate a large reservoir of phosphorus to sustain seedling growth. The principal form of phosphorus storage in seeds is in the form of phytate (*myo*-inositol hexakisphosphate; $InsP_6$) (Ravindran et al., 1994). The bulk of mature grain phytase activity in cereals can be attributed to phytases belonging to the large family of purple acid phosphatases (PAPs). PAPs belong to the calcineurin-like metallo-phosphoesterase superfamily (Matange et al., 2015) and are known to require a heterovalent bimetal center (MI, MII) for their catalytic activity. MI is always a ferric ion (Fe^{3+}) and the identity of MII has been reported to be either Fe^{2+}, Zn^{2+} or Mn^{2+} depending on the protein (Schenk et al., 2013; Matange et al., 2015). PAPs form two distinct groups according to their molecular weights. The first category is referred to as high molecular weight (HMW) PAPs. They are mostly large 55–60 kDa plant and invertebrate enzymes with an N-terminal regulatory domain in addition to a metallophosphoesterase (MPE) domain. HMW PAPs are often homodimers linked by a disulfide bridge formed by a conserved cysteine and contain a heteronuclear metal center with Zn^{2+} or Mn^{2+} in the MII site (Olczak et al., 2003; Schenk et al., 2013; Matange et al., 2015). The second category is formed from mammalian, plant, and invertebrate enzymes that contain only the MPE domain. They are monomers of approximately 35 kDa usually referred to as low molecular weight (LMW) PAPs and present a Fe^{3+}-Fe^{2+} homobinuclear metal center (Olczak et al., 2003; Schenk et al., 2013; Matange et al., 2015). HMW and LMW PAPs were first identified in plants (Schenk et al., 2000) and then verified in humans (Flanagan et al., 2006). Although the members of this superfamily are functionally

Published by the Plant Communications Shanghai Editorial Office in association with Cell Press, an imprint of Elsevier Inc., on behalf of CSPB and CEMPS, CAS.

© 2022 University of East Anglia.
This is an open access article under the CC BY license (http://creativecommons.org/licenses/by/4.0/).

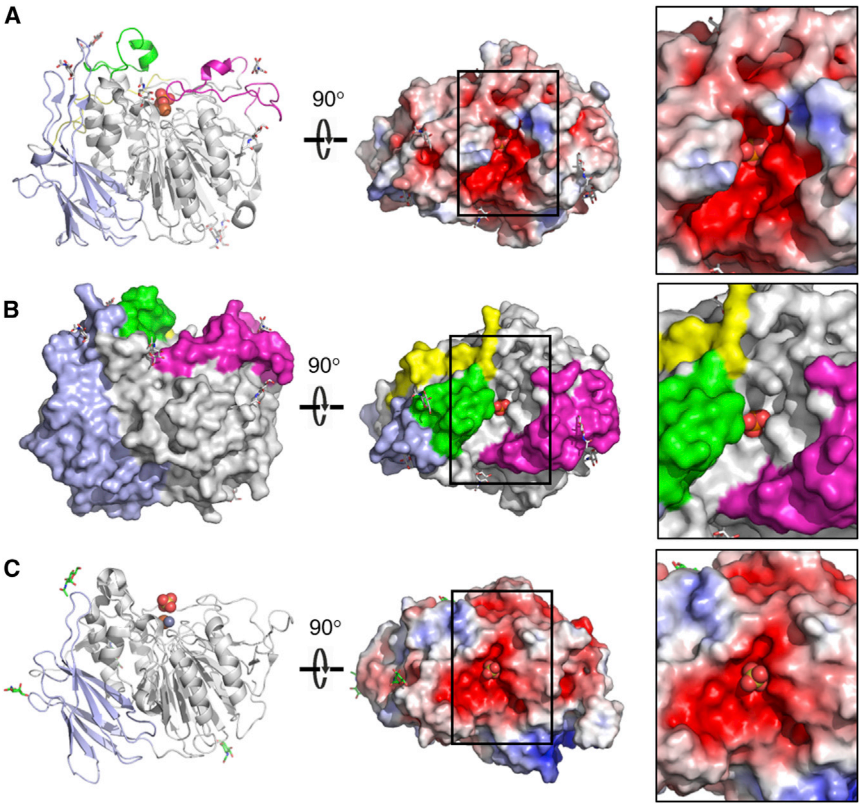

Figure 1. PAPhy motifs define the shape, volume, and charge distribution of the active site cavity.

(A) Left panel, cartoon of the crystal structure of TaPAPhy_b2 in the product-bound state (this study, PDB entry 6GIT), side view. Polypeptide chain colored as follows: fibronectin-like FN3 domain, light blue; metallophosphoesterase (MPE) domain, gray; PAPhy 1 motif, yellow; PAPhy 4 motif, green; PAPhy 5 motif, light magenta. N-actylglucosamine (NAG) groups are shown as sticks, and the binuclear center and bound orthophosphate are shown in ball and stick format. Middle panel, top view of molecular surface colored by electrostatic potential (red, acidic; blue, basic). Black box indicates active site region. Right panel, expanded view of active site region. A collar of electropositive potential surrounds the bound phosphate group visible in the center.

(B) Left panel, view of the molecular surface of TaPAPhy_b2 oriented and colored as in panel **(A)**. Middle panel, top view of surface oriented as in panel **(A)**. Right panel, expanded view of active site region. PAPhy motifs 4 (green) and 5 (light magenta) help define the shape and volume of the active site cavity.

(C) Left panel, cartoon of the crystal structure of red kidney bean phosphatase (PDB: 2QFR), side view. Polypeptide chain colored as follows: fibronectin-like FN3 domain, light blue; metallophosphoesterase (MPE) domain, gray. NAG groups are shown as sticks and orthophosphate bound at binuclear center is shown in ball and stick format. Middle panel, top view of molecular surface colored by electrostatic potential. Black box indicates active site region. Right panel, expanded view of active site region with bound sulfate group visible in the center. The active site region is generally electronegative and lacks the features usually associated with recognition of a large, negatively charged substrate. This is consistent with low specificity toward phytate.

diverse and have low overall sequence similarity, both the core MPE fold and the architecture of the active site are conserved (Matange et al., 2015). Not all PAPs can effectively utilize phytate as substrate. PAPs that can hydrolyze phytate are known as PAPhy (Dionisio et al., 2011). Activity toward phytate as substrate is an unexplained diversification/specialization of the PAPs that is unique to plants where they are essential germinative activities. In contrast to the situation with other phytase families (Lim et al., 2000; Chu et al., 2004; Zeng et al., 2011), the structural basis for PAPhy specificity toward phytate essential as a precursor to rational engineering of phytase activity is not well understood.

RESULTS

The X-ray crystal structure of a wheat PAPhy

To shed further light on the structure-function relationships of enzymes responsible for the degradation of phytate in cereals, we set out to solve the X-ray crystal structure of a wheat purple acid phytase. We focused our attention on a b-isoform enzyme induced during germination, TaPAPhy_b2, selected based on its stability and yield following overexpression. To reduce the heterogeneity introduced by hyperglycosylation of recombinant proteins observed following expression in *Pichia pastoris*, a glycoengineered version of the KM71H *P. pastoris* strain was constructed. In this KM71H (OCH1:G418R) strain the OCH1 gene has been replaced with G418R, which reduces mannosylation and confers geneticin resistance. The enzyme was purified by immobilized metal ion chelate chromatography and gel filtration. Crystals grown using glycoengineered recombinant protein diffracted only to low resolution, so the protein was enzymatically partially deglycosylated and repurified before crystallization. The resulting crystals grew in space group *H*3 and diffracted to 1.42 Å resolution. The X-ray crystal structure was solved by molecular replacement using red kidney bean PAP (PDB: 2QFR) as a search model.

The refined crystal structure contains a monomer comprising residues Pro2-Leu508 of the 510-residue protein in the asymmetric unit (Figure 1). Four disulfide bonds and seven N-glycosylation sites are present, all according to previous predictions (Dionisio et al., 2011, 2012). In keeping with other HMW PAPs, TAPhy_b2 consists of a smaller fibronectin type III (FN3) non-catalytic N-terminal domain (Tsyguelnaia and Doolittle, 1998) (residues Pro43–Thr156) together with a larger C-terminal MPE domain (residues Arg168–Glu497). The structure of the core of the wheat enzyme closely resembles those of other plant HMW PAPs such as those from kidney bean (Schenk et al., 2008) (PDB: 2QFR; percentage amino acid sequence identity (PID) 34%; root-mean-square deviation (RMSD) 0.84 Å over 294 residues) and sweet potato (Schenk et al., 2005) (PDB: 1XZW; PID 35%; RMSD 0.75 Å over 291 residues). A preference for Fe^{2+} in the MII site of the MPE domain has been described for TaPAPhy_b2 (Dionisio et al., 2011), a feature arising perhaps as a route to cellular redox

regulation (Rusnak and Reiter, 2000), and correspondingly two iron ions were modeled in the electron density present at the active site. Anomalous scattering peaks calculated using a dataset collected at the iron K-edge (Fe-SAD) supported this interpretation (Supplemental Figure 1). X-ray fluorescence spectra combined with absorption edge scans confirmed the absence of manganese. The iron in the MI site is tetrahedrally coordinated by residues Asp174, Tyr204, His379, and Asp201, the latter of which bridges the two metal ions. The iron in the MII site is octahedrally coordinated by Asn258, His340, His377, and the bridging residue Asp201. A phosphate ion is bound to the two metal ions and the side chains of His259, His350, and Glu409. The binding mode of the phosphate ion, together with the lack of electron density for a bridging solvent molecule, resembles that found in the structure of the red kidney bean PAP:orthophosphate complex in the product-bound state (Klabunde et al., 1996; Schenk et al., 2008) (PDB: 4KBP). We obtained a second structure of the TaPAPhy_b2:PO_4 complex refined at 1.54 Å resolution from a further crystal in the same space group. The phosphate ion bound to the active site in this case resembled the structure of the pig PAP:orthophosphate complex in the substrate-bound state (Guddat et al., 1999; Schenk et al., 2008) (PDB: 1UTE) and included spherical electron density for a μ-hydroxide moiety bridging the two irons (Supplemental Table 1; Supplemental Figure 2). Despite screening numerous crystals, we failed to obtain a structure for TaPAPhy_b2 resembling the transition state of the reaction, a feature previously observed in the very high-resolution structure of pig PAP (Selleck et al., 2017) characterized by the phosphate ion binding only to MII.

PAPhy motifs determine the shape and charge distribution of the active site

Previous bioinformatics analysis has identified four phytase-specific polypeptide insertions (termed PAPhy motifs 1–4) in the sequences of plant purple acid phytases relative to those of HMW PAPs, together with five PAP motifs (I–V) that identify metal-binding sequences in the MPE domain (Dionisio et al., 2011). With access to the crystal structure, we are now in a position to assign possible roles for the individual PAPhy motifs in the structure-function relationships of the enzyme. The wheat phytase motifs PAPhy 2 (Ser82–Gly87) and PAPhy 3 (Ala147–Pro158) are located within the N-terminal domain and, therefore, are unlikely to be involved in determining specificity toward phytate (although a regulatory role cannot be discounted). PAPhy 1 (Arg21–Arg37) is found near the N-terminus of the protein but does not form part of the N-terminal domain. Lying adjacent to the active site it may have a secondary role in contributing to specificity through a salt bridge interaction with PAPhy 4 involving His23 and Asp216. The PAPhy 4 motif (Leu209–His229) lies within the MPE domain and lines one side of the active site cavity. Analysis of the crystal structure revealed a further, previously unidentified polypeptide insertion lining the other side of the cavity (residues Asp418–Gln455). To investigate the conservation of this loop region among the wider PAP family, we conducted a detailed sequence analysis comprising PAPhys (both characterized and predicted) and PAPs from a variety of organisms. The resulting alignment (Supplemental Figures 3 and 4) demonstrated this loop to form part of a highly conserved sequence insertion in PAPhys that is absent in canonical PAP enzymes and which we name PAPhy 5. Together, motifs 4 and 5 create an electropositive horseshoe-shaped collar mounted on the strikingly electronegative active site landscape found in PAPs (Figure 1). By virtue of their roles in defining the shape, volume, and charge distribution of the active site cavity, the PAPhy 4 and 5 motif insertions provide ideal candidates by which the specificity of a PAP enzyme may be tuned to phytate.

The presence of PAPhy motifs 4 and 5 correlate with high specificity toward phytate

To study the emergence of PAPhy motifs within the wider PAP family, a phylogenetic tree was constructed that, when combined with substrate specificity and activity data (Supplemental Table 2), provides an insight into the emergence of specificity toward phytate in PAPhys (Figure 2). LMW plant PAPs lack PAPhy motifs. Very few of these have been cloned and characterized but are predicted to have broad activity against phospho-substrates (Liang et al., 2010). A PAPhy 2-like motif appears in roughly 75% of HMW PAPs, but all lack PAPhy motifs 4 and 5. The substrate specificity of these PAPs is generally broad, although a quarter of the clade show some activity toward phytate. Between the HMW PAP clade and the PAPhy clade a further small clade of enzymes that possess an intermediate set of PAPhy motifs is found. These all contain PAPhy 1- and PAPhy 2-like motifs, with many also possessing a PAPhy 3-like motif. Only one member of this clade, AtPAP23, has been characterized, and while showing strongest activity toward other phospho-substrates, it also has a reasonable activity against phytate (Zhu et al., 2005). The plant PAPhys are split into two subclades containing either monocots or dicots. While all enzymes in both clades possess PAPhy motifs 1–5, the dicot clade contains only a partially conserved PAPhy 4 motif. Characterized dicot enzymes show a broad specificity, although we note that the tobacco and maize enzymes have a low K_m for phytate indicating a higher specificity for this substrate. On the other hand, the monocot clade enzymes possess well-conserved PAPhy motifs 4 and 5. All characterized members of this clade have high activity and a low K_m for phytate. The presence of PAPhy motifs 4 and 5 therefore correlates with high specificity toward phytate.

Binding of a phytate analog inhibitor reveals a potential substrate standby binding mode

Wheat phytases and, in general, plant phytases are commonly classified as 6-phytases (EC 3.1.3.26), that is, they display a preference of hydrolysis for the L-6 (D-4) phosphate of phytate (Lim and Tate, 1973; Nakano et al., 1999, 2000; Brinch-Pedersen et al., 2002; Bohn et al., 2007; Bohn et al., 2008; Wu et al., 2015). To investigate the hydrolysis of phytate by the recombinant enzyme, inositol polyphosphate products of hydrolysis were separated by acid elution from a high-performance liquid chromatography (HPLC) anion exchange column with subsequent detection of inositol phosphate-ferric complexes (Phillippy and Bland, 1988; Blaabjerg et al., 2010) (Figure 3A). As expected, recombinant TaPAPhy_b2 showed a strong preference for initial hydrolysis of the phosphate in position D-4 and/or D-6 of the inositol ring (since these columns do not resolve enantiomers, it is not possible to conclude whether the peak corresponds to one or both intermediates). To investigate the structural basis for this

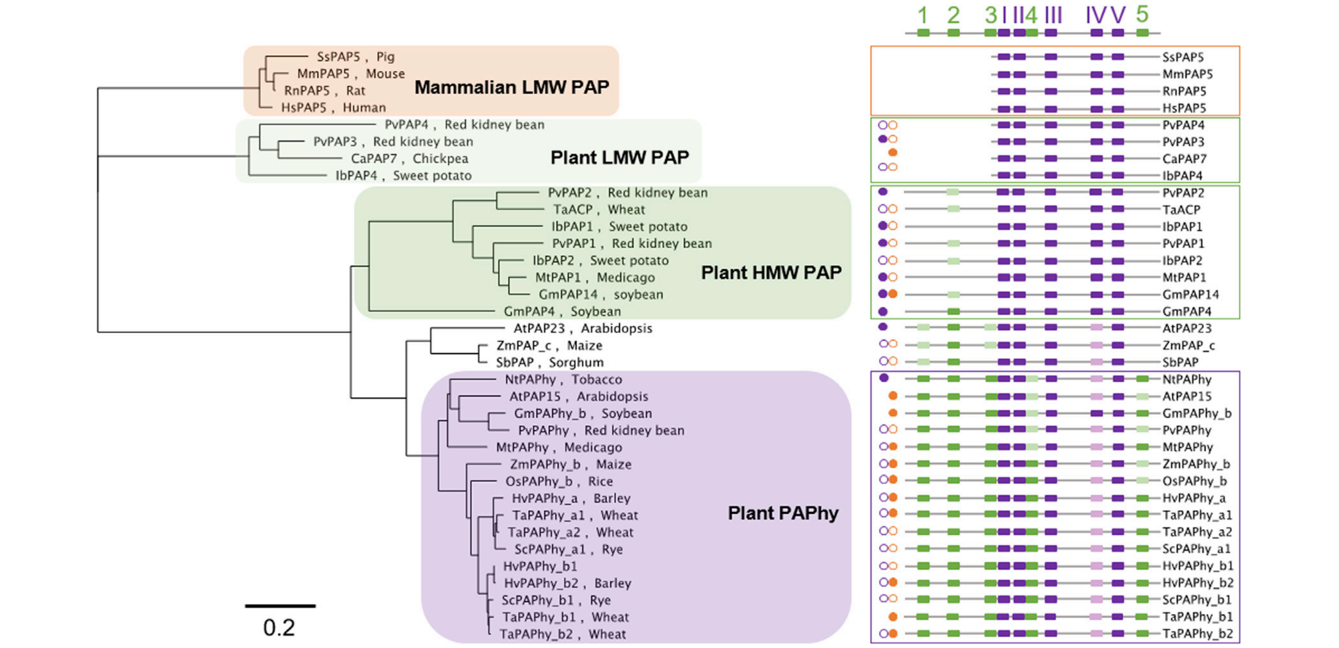

Figure 2. PAPhy motifs provide an insight into the emergence of specific phytase activity in plants.

Left panel, phylogenetic tree constructed from a set of biochemically characterized members of PAP and PAPhy enzyme families. A key to enzyme identifiers can be found in Supplemental Table 2. Mammalian low molecular weight (LMW) PAPs are enclosed in an orange box, plant LMW PAPs in a light green box, plant HMW PAPs in a green box, and plant PAPhys in a mauve box. Right panel, schematic representation of the distribution of PAPhy motifs in sequences appearing in the phylogenetic analysis. Green boxes represent PAPhy motifs, numbered 1–5. Metal-binding PAP motifs (Dionisio et al., 2011; Schenk et al., 2013) are shown as dark purple and numbered I–V. The depth of color of both PAPhy and PAP motifs indicates their degree of similarity with darker coloring indicating higher sequence conservation. Small circles to the left of each sequence in the panel indicate documented phosphatase (closed purple) and phytase activities (closed orange), respectively. Open circles indicate predicted activities. Monocot clade enzymes possess well-conserved PAPhy motifs 4 and 5. All characterized members of this clade have high phytase activity and a low K_m for phytate.

observed positional hydrolytic specificity, we grew cocrystals of the complex of TaPhy_b2 with the non-hydrolyzable phytate analog, *myo*-inositol hexakissulfate ($InsS_6$). While $InsS_6$ inhibits the phytase activity of TaPAPhy_b2 *in vitro*, the crystal structure reveals it to fail to bind to the enzyme in such a way as to mimic phytate in a productive binding mode, contrary to what is typically seen for other classes of phytase (Lim et al., 2000; Chu et al., 2004; Zeng et al., 2011; Acquistapace et al., 2020). Instead, it binds at a site immediately adjacent to the active center (Figure 3B; Supplemental Figures 5 and 6). This may represent phytate (or a partially dephosphorylated inositol polyphosphate) in a standby mode between cycles of catalysis. A similar binding mode has been observed in a phytase of the protein tyrosine phosphatase class (Chu et al., 2004).

A model for phytate recognition by wheat PAPhy

In an alternative approach to identify substrate specificity pockets, a molecular dynamics (MD) simulation of a modeled TaPAPhy_b2:phytate complex was performed at pH 5.5 and 298 K. Phytate was manually docked into the active site of the enzyme, superimposing the D-4 phosphate group of phytate onto the bound inorganic phosphate molecule of the TaPAPhy_b2 substrate complex. A 100-ns MD simulation was then performed to allow the substrate to sample conformational space within the active site. Geometric clustering was performed to identify similar structures sampled during the trajectory. The central member of the cluster with the highest population (representing 91% of the total) was taken to represent the productive enzyme-substrate complex that allowed a network of protein-substrate interactions to be defined (Figure 3C). To simplify the description of the interactions, we propose a nomenclature for the six specificity pockets. In this scheme the pocket responsible for binding the scissile phosphate is named A. With the D-4 phosphate group in pocket A and orienting the axial D-2 phosphate group toward the viewer, the remaining specificity pockets are sequentially named B–F in an anticlockwise fashion following the order of increasing phosphate number attached to the inositol ring. Using this nomenclature, the PAPhy 4 motif contributes to pockets D and F, while residues of the PAPhy 5 motif contribute to pocket E. Of the former, His229 is found in pocket F contacting phosphate D-3, while the D-1 phosphate contacts Ser219 and the PAPhy 4 α-helix macrodipole. The short helical turn consisting of PAPhy 5 residues Ala431–Met433 contacts the 2-phosphate, mainly through interactions with its main chain. Residue Lys410 contacts phosphates D-5 and D-6 in pockets B and C, respectively. While the amino acid pair Glu409-Lys410 is highly conserved in PAPhys, it is predominantly encountered as Glu–Gly in PAPs. The presence of PAPhy motifs 4 and 5 and incorporation of a lysine residue at position 410 may therefore constitute the major requirements for specific phytase activity. The remainder of the contacts to phytate in pockets A and F are provided by residues conserved across HMW PAPs and PAPhys and so presumably contribute toward the broader phospho-substrate specificity of the family and thus do not contribute explicitly to specificity toward phytate.

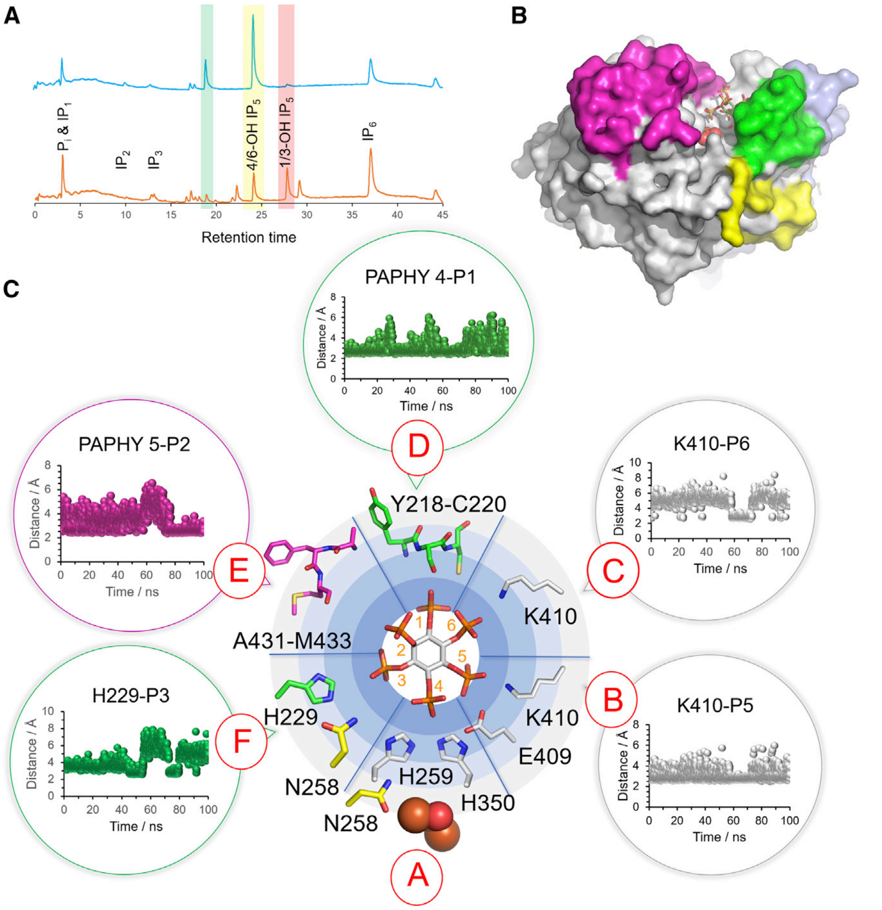

Figure 3. What is the structural basis for recognition of phytate?

(A) HPLC chromatogram of $InsP_6$ hydrolysis by recombinant TaPAPhy_b2 (blue trace). A chromatogram of an acid hydrolysate of the substrate (*myo*-inositol polyphosphate standards) is shown for reference (gold trace). The elution volume ranges for the various inositol polyphosphate product of phytate hydrolysis are highlighted by vertical colored backgrounds (note that the notation for the $InsP_5$ products is based on the identity of the free hydroxyl group of the intermediate—red: 1/3-OH $InsP_5$; yellow: 4/6-OH $InsP_5$). The green vertical bar highlights 1234/1256-OH $InsP_4$, a major $InsP_4$ product of PAPhy activity. The potential of marginal D-1 and/or D-3 phytase activity was also noted, but a contaminant peak was also present in the undegraded substrate.

(B) A view of the crystal structure of the complex of TaPAPhy_b2 with the substrate analog inhibitor, $InsS_6$. The inhibitor is shown in stick format. The molecular surface of the enzyme is colored as in Figure 1 to reveal the PAPhy motifs. The phosphate group bound at the catalytic center is shown in sphere format.

(C) A representation of the predicted specificity subsites (pockets) of TaPAPhy_b2 showing those active site residues that are predicted to form contacts with the bound substrate. Pockets are labeled A–F (red capital letters in red circles). Contact residues are labeled and shown in stick format, and colored according to their assignment within either the PAPhy motif 4 (green), the PAPhy motif 5 (light magenta), or the MPE domain (gray). Note that Asn258 is also a ligand to iron in the MII site and is colored yellow. In the center of the image sits a stick representation of $InsP_6$ positioned so that the D-4 phosphate is located in specificity pocket A. This orientation places the axial 2-phosphate in specificity pocket E. Also shown for individual specificity pockets B–F are plots of the minimum contact distance from individual residues (H229, K410) or elements of PAPhy motifs 4 and 5 to the corresponding phosphate group of the substrate during the 100-ns MD simulation.

Mutagenesis identifies active site elements with central roles in phytase activity

To validate our assignment of specificity pocket contents in TaPAPhy_b2, we turned to site-directed mutagenesis of predicted active site residues. Basic residues were chosen for alanine mutagenesis if they were found less than 6 Å from the predicted position of the bound substrate and were conserved in PAPhy but not in the non-phytase HMW PAPs. This process identified two residues highlighted from analysis of the MD trajectory, His229 (Pocket F) and Lys410 (pockets B and C), and one other, K348 (Figure 4A). The side chain of K348 forms direct hydrogen bonding contacts with residues of the PAPhy 5 motif and may have an indirect influence on phytase activity. The sequential degradation of phytate by the mutants as followed by HPLC and the pH-dependence of their phytase activities were indistinguishable from that observed for the wild-type enzyme (Supplemental Figures 7–9). The relative phytase activities of the mutants were <5% for H229A and 13% for K410A (Figure 4B). Kinetic parameters can be found in Supplemental Table 3. The rate constant for K348A was not significantly different from that of the wild-type enzyme. Mutation of K410 therefore significantly reduced phytase activity consistent with a central role of this residue as a major determinant of specificity toward phytate as previously predicted (Feder et al., 2020). That the phosphatase activity of this mutant toward an alternative substrate, *p*-nitrophenyl phosphate, is not significantly reduced from that of the wild-type enzyme is in keeping with the view that K410 is required to solvate the charges on the phosphates of bound phytic acid occupying pockets B and C (Supplemental Figure 10). The K348A mutant shows only minor change in phytase activity. On the other hand, the H229A mutant showed highly attenuated activity toward phytate. Crystallization of this mutant allowed its structure to be solved at 1.50 Å resolution, which proved it to be essentially identical to that of the wild-type enzyme. However, a region of discontinuous electron density was identified between residues Asp216 and Pro227, covering most of the PAPhy 4 motif. The lack of structural order in this region of the mutant enzyme can be explained by the deletion of a π-stacking interaction between His229 and Tyr218 following mutagenesis. Such interruption presumably results in instability of the PAPhy 4 motif, emphasizing the importance of this motif in phytate binding and recognition.

Specificity pocket composition is conserved in other common cereal phytases

To explore variation in the amino acid composition of the predicted phytate specificity subsites among plant PAPhys and its relationship to the positional specificity of phytate hydrolysis, we turned our attention to the closely related enzymes from barley

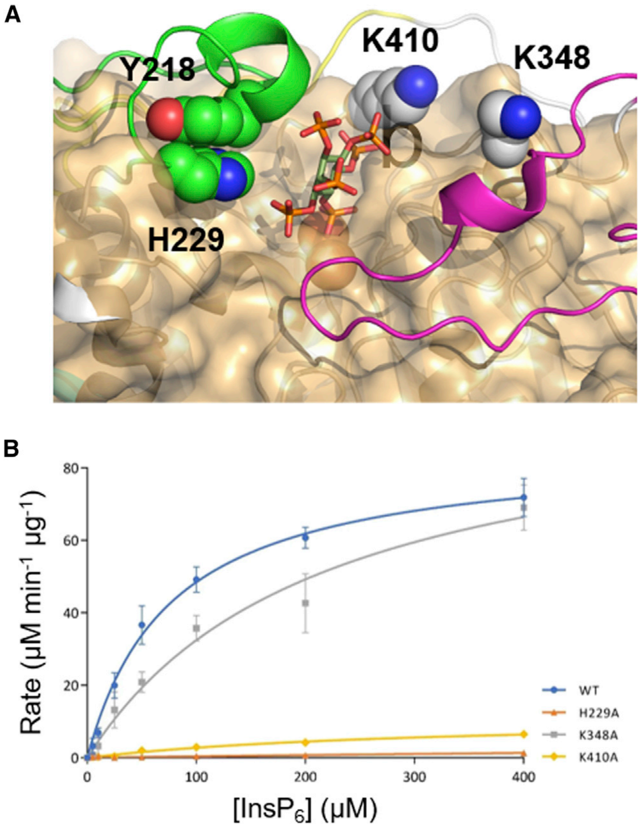

Figure 4. Site-directed mutagenesis of active site residues suggests central roles for PAPhy 4 and K410 in phytase activity.
(A) A close-up view of the active site of TaPAPhy_b2. Polypeptide chain colored as follows: metallophosphoesterase (MPE) domain, gray; PAPhy 4 motif, green; PAPhy 5 motif, light magenta. Residues selected for mutagenesis are shown in sphere format and labeled (also shown is Y218, which forms a π-stacking interaction with H229). The coloration of the residues follows their assignment to either the MPE domain (K348 and K410) or the PAPhy 4 motif (H229 and Y218). The structure is overlaid with the molecular surface of red kidney bean PAP (gold color; PDB: 2QFR) and shows phytate in stick format in its predicted location from the 100-ns MD trajectory. The binuclear center is visible at the base of the TaPAPhy_b2 active site.
(B) Michaelis-Menten kinetics of WT TaPAPhy_b2 and active site mutants H229A, K348A, and K410A. Error bars represent the standard deviations of triplicate measurements.

(HvPAPhy_a), rice (OsPAPhy_b), maize (ZmPAPhy_b), and soybean (GmPAPhy_b). Amino acid sequence identities relative to TaPAPhy_b2 range from 72% (GmPAPhy_b) to 91% (HvPAPhy_a). Comparative modeling was used to predict the structures of the four enzymes, and the sequence variabilities at 33 active site residue positions falling within 10 Å of the bound phosphate group in the crystal structure of TaPAPhy_b2 were assessed (Supplemental Figure 11, Supplemental Table 4). The active site residues of the cereal enzymes HvPAPhy_a, OsPAPhy_b, and ZmPAPhy_b vary from the TaPAPhy_b2 sequence at 42%, 27%, and 36% of residue positions, respectively. Notably, 67% of residues vary between TaPAPhy_b2 and GmPAPhy_b. In contrast, the conservation of residues in PAPhy motifs 4 and 5 that are predicted to be in contact with the substrate is high. Nevertheless, notable differences are seen in the E specificity pocket in the PAPhy 5 motif, involving residues Ala431 (proline in OsPAPhy_b, ZmPAPhy_b and GmPAPhy_b), Phe432 (tyrosine in GmPAPhy_b), and Met433 (isoleucine in HvPAPhy_a). Despite this, our modeling suggests the contribution of these residues to the binding of phytate is through their main chain amino groups rather than their side chains, and as such, these residue changes are not expected to influence the positional specificity of phytate hydrolysis.

The recombinant plant enzymes were prepared by heterologous expression using the KM71H::OCH1 glycoengineered strain of *P. pastoris* in the same manner as for TaPAPhy_b2. The purified enzymes were assayed for phytase activity under standard conditions at a range of enzyme concentrations except for GmPAPhy_b for which sufficient sample was obtained to permit assay at only a single unique concentration (Supplemental Figure 12). The resulting order of specific phosphate release activities was TaPAPhy_b2 > HvPAPhy_a > ZmPAPhy_b > OsPAPhy_b >> GmPAPhy_b. Insufficient soybean enzyme was available so HPLC profiles of inositol polyphosphates resulting from $InsP_6$ degradation by only the recombinant cereal PAPhy enzymes were recorded (Supplemental Figures 13–16). Identical profiles of hydrolysis intermediates were obtained in reactions performed with all cereal enzymes tested, confirming a conserved D-4 and/or D-6 phytase activity. However, as the method cannot resolve the enantiomers D-Ins(1,2,3,5,6)P_5 and D-Ins(1,2,3,4,5)P_5, their absolute specificities remain unresolved. Hence, while the residue changes observed in the active sites of the cereal enzymes studied do not appear to alter the positional specificity of phytate hydrolysis, substitutions in the specificity pockets, particularly specificity pocket E, may serve to modulate specific phytase activity.

DISCUSSION

Cereals and legumes form a significant component of the food supply for humans and other animals, and constitute a major source of dietary carbohydrate, protein, lipids and minerals. Phytate is the major storage form of phosphorus in mature grains and legumes, contributing 60%–80% of the total (Viveros et al., 2000; Humer et al., 2015). Hydrolysis of phytate is catalyzed by phytases to yield bioavailable orthophosphate, and high native phytase activities are present in cereals and cereal by-products (Madsen et al., 2013; Brinch-Pedersen et al., 2014). Phytase activity usually increases on germination, and germination has historically been used to induce this activity in cereals. Crystal structures of plant PAPs, enzymes with broad specificity for phospho-substrates, have been available since 1995 (Sträter et al., 1995). However, these structures have failed to explain how specificity for phytate could be achieved by the closely related PAPhy. The high-resolution crystal structures of the wheat purple acid phytase isoform b2 reported herein provide a straightforward explanation by highlighting the roles of two sequence inserts (PAPhy motifs) relative to a canonical MPE domain in forming phytate-specific substrate specificity pockets. In this way, PAPhy motifs 4 and 5 serve to neofunctionalize a plant HMW PAP domain, endowing it with hydrolytic specificity toward phytate. Building on this, amino acid sequence analysis neatly explains the highly specific phytase activity observed in monocot cereals.

Our attempts to use a non-hydrolyzable analog to provide details of interactions with bound phytate allowed us instead to identify a

possible standby mode of substrate binding. We therefore turned to MD simulation. MD simulations have a become primary tool used to investigate the action of biological macromolecules. Based on high-resolution structures of enzymes, MD simulations can be used to gain detailed insights to substrate binding and catalysis (Koch et al., 2013; Chen et al., 2015; Iqbal and Shah, 2018). Clustering of states from a 100-ns MD simulation of TaPAPhy_b2 have allowed the identification of a dominant bound conformation for phytate. This binding mode is stabilized by interaction of phytate with residues of PAPhy motif 4 in specificity subsites D and F, and with residues of PAPhy motif 5 in subsite E. Furthermore, residue K410 was identified as a central player in sensing phosphate groups of substrate in specificity pockets B and C. Despite substantial numbers of amino acid variations between the active sites of the plant PAPhys considered, all the cereal PAPhys tested generated the same phytate degradation profile, regardless of the plant species or the enzyme isoform. However, the phytase activities of the cereal enzymes (Viveros et al., 2000; Steiner et al., 2007; Dionisio et al., 2011) vary considerably, suggesting that mining of amino acid sequence data together with crystal structure and activity data may be a profitable route to identify substitutions leading to enhanced cereal mature grain phytase activity.

As phytase activity in food and feedstuffs is an important nutritional parameter, our structural data offers direction to manipulation of phytase activity *in planta* with implications for the development of crops with engineered inositol polyphosphate content or enhanced mature grain phytase activity.

METHODS

Sequence analysis

The amino acid sequences of known PAPhy were collected and compared with those of PAPs demonstrated to lack phytase activity to determine key differences in addition to those described previously (Dionisio et al., 2011). A total of 124 PAP sequences were analyzed (Supplemental Table 5), of which 112 were collected from the UniProt database (The UniProt Consortium, 2017) and the remaining 12 were retrieved from Phytozome version 12.0 (Goodstein et al., 2012) or BLASTP (Altschul and Gish, 1996) searches following the methods described by Rivera-Solís et al. (Rivera-Solís et al., 2014). A multiple sequence alignment of the PAPhy and PAP sequences was performed using the MUSCLE algorithm (Edgar, 2004) with default parameters and analyzed with Jalview (Waterhouse et al., 2009). A phylogenetic analysis of the PAP sequences was performed with MEGA7 (Kumar et al., 2016), and a phylogenetic tree was constructed using the maximum likelihood method with default parameters.

Production of the *OCH1::G418R* hyperglycosylation knockout of the *Pichia pastoris* KM71H strain

P. pastoris strain KM71H was chosen since it is Mut^S, a phenotype of slow methanol utilization. The abolition of hyperglycosylation will render homogeneous the type of glycosylation to an average of $Man_{8-14}GlcNAc_2$ (Bretthauer and Castellino, 1999; Jacobs et al., 2009) better compatible with subsequent deglycosylation and crystallization. For this purpose, a knockout construct was generated of the ORF of the gene *OCH1*, encoding a mannosyltransferase of the *cis*-Golgi apparatus (XM_002489551, PAS_chr1-3_0251). PCR using the *OCH1* cloning primers was used to verify the correct gene substitution: 1299 bp was the PCR product for the escape transient expression and 2591 bp for the correct knockout integration product.

Preparation of recombinant TaPAPhy_b2 samples

Recombinant TaPAPhy_b2 in fusion with an N-terminal peptide encoding the *Saccharomyces cerevisiae* α-factor secretion signal and a C-terminal 6xHis affinity tag was produced from a pGAPZαA (Invitrogen) construct (Dionisio et al., 2011). This construct uses the promoter of the glyceraldehyde-3-phosphate dehydrogenase enzyme to drive the constitutive production of extracellular TaPAPhy_b2 protein in *P. pastoris*. A 20-amino acid signal peptide and a C-terminal seven-amino acid ER-retention signal was excluded from the construct. TaPAPhy_b2 was obtained by growing a *P. pastoris* KM71H (*OCH1::G418R*) transformant with TaPAPhy_b2-pGAPZαA in 800 ml of buffered minimal glucose medium for 5 days under continuous shaking (200 rpm) at 26°C. The resulting supernatant was concentrated to 50 ml using a stirred cell (Amicon) with a regenerated cellulose ultrafiltration membrane (10 kDa NMWL; Merck).

Recombinant His-tagged protein was purified by metal affinity chromatography and deglycosylated at 4°C overnight in 1x GlycoBuffer 3 (50 mM sodium acetate, pH 6.0; NEB) with 100 000 U mg^{-1} recombinant GST-Endo F1 produced as described by Grueninger-Leitch et al. (Grueninger-Leitch et al., 1996) (Supplemental Figure 17). Deglycosylated protein (TaPAPhy_b2d) was purified by glutathione affinity chromatography followed by size exclusion chromatography. TaPAPhy_b2d was subsequently concentrated and dialyzed against 20 mM Tris–HCl (pH 8.0) for analysis. Single-site mutants H229A, K348A, and K410A were generated using a modified version of the QuickChange site-directed mutagenesis method (Liu and Naismith, 2008). The transformation, expression, and purification of the mutants were performed as for the wild-type enzyme.

Preparation of recombinant plant PAPhys

Barley (HvPAPhy_a), rice (OsPAPhy_b), and maize (ZmPAPhy_b) PAPhy genes cloned in the vector pPICZαA (Dionisio et al., 2011) were used in this study. A synthetic gene for the soybean enzyme GmPAPhy_b was cloned into the Gateway entry vector pDONR207 and then transferred to the destination vector pPICZα-DEST (Sasagawa et al., 2011). The transformation and expression of the four cereal PAPhy-pPICZα constructs was performed essentially identically as for the TaPAPhy_b2 site mutants and utilized the KM71H (OCH1:G418R) *P. pastoris* glycoengineered strain. A preference for manganese in the MII site has been described for PAPhy_a isoforms (Dionisio et al., 2011), so for the expression of the barley a-isoform, HvPAPhy_a, 100 μM manganese(II) sulfate was also added to the buffered minimal methanol medium. The enzymes were purified by nickel-affinity chromatography and stored in 20 mM Tris–HCl (pH 8.0) buffer containing 30% (v/v) glycerol at –80°C.

Phosphate release assays

Enzymatic characterization was performed with purified glycosylated proteins after nickel-affinity chromatography purification by means of standard phosphate release assays (Nagul et al., 2015) in 0.2 M acetate (pH 5.5) buffer with 5 mM potassium phytate (≥95% purity, Sigma). Absorbance at λ = 700 nm was subsequently measured in a microplate reader (Hidex Sense) after color development for 30 min.

HPLC separation of products of enzymatic phytate hydrolysis

The product profiles of reaction of all wild-type and mutant cereal PAPhys with $InsP_6$ were obtained by separating the inositol phosphate products on HPLC after the method of Blaabjerg et al. (Blaabjerg et al., 2010).

X-ray crystal structure determination

Crystallization was performed using the sitting drop vapor diffusion method at 16°C with protein concentrated to 7–8 mg ml^{-1}. Crystals in space group *H*3 grew in drops containing 0.2 M sodium thiocyanate and 20% (w/v) PEG 3350, and they were cryoprotected by addition of 25% (v/v) PEG 400. To obtain the crystal structure of the TaPAPhy_b2:$InsS_6$ complex, crystals were soaked for 4 min in a solution of 5 mM *myo*-inositol hexakissulfate ($InsS_6$) at pH 5.5 adjusted with

acetate buffer. X-ray data was collected at Diamond Light Source (Didcot, UK) on beamlines I03 and I04 at wavelengths of 0.9763 Å (12.6994 keV) for native datasets and 1.7389 Å (7.1300 keV) for datasets collected at the iron K-absorption edge. The PHENIX suite (Adams et al., 2010) was used for structure solution and refinement. The crystal structure of TaPAPhr_b2d was solved by molecular replacement (MR) using as search model the structure of red kidney bean PAP (PDB: 2QFR; Schenk et al., 2008). MR solutions were subjected to several rounds of manual remodeling using COOT (Emsley et al., 2010) followed by refinement with PHENIX REFINE (Adams et al., 2010). Crystal parameters, data collection, and refinement statistics for the TaPAPhy_b2 structures are summarized in Supplemental Table 6.

Molecular dynamics simulations

A dynamic model of the TaPAPhy_b2:$InsP_6$ complex was obtained through molecular modeling and MD simulation. This approach utilized a modified version of the crystal structure of the TaPAPhy_b2:PO_4 complex resembling substrate binding containing a μ-(hydr)oxo bridge in the active site. Simulations were performed using the GROMACS 2020.4 molecular dynamics package (Hess et al., 2008) with the amber99sb-ildn force field (Oostenbrink et al., 2004). $InsP_6$ coordinates and topology were obtained from ATB version 3.0. $InsP_6$ was modeled as $C_6H_{12}O_{24}P_6^{6-}$ at pH 5.5 according to Veiga et al. (Veiga et al., 2014). To generate starting coordinates for the complex the D-4-phosphate of phytate was manually docked to superimpose the active site phosphate found in the crystal structure. MD simulations were carried out with weak restraints applied to the position of the two iron ions, the amino acid residues coordinating the irons, the μ-oxo bridge, and the phosphate molecule coordinated to the metals. An MD simulation of 100 ns duration of the TaPAPhy_b2:$InsP_6$ complex in aqueous solution was then performed at a constant temperature of 298 K. Analysis of the MD trajectory was carried out using embedded tools in the GROMACS package.

Other software

PyMOL (Schrodinger, 2015) was used for the visualization of protein models and preparation of Figures. The APBS (Baker et al., 2001) plug-in to PyMOL was used to calculate electrostatic potential contour maps.

Full details of all methods can be found in the supplemental information.

SUPPLEMENTAL INFORMATION

Supplemental information can be found online at *Plant Communications Online*.

ACCESSION NUMBERS

Atomic coordinates and crystallographic structure factors have been deposited in the Protein Data Bank under accession codes 6GIT (product-bound form), 6GIZ (substrate-bound form), 6GJA (H229A mutant), and 6GJ2 (complex with $InsS_6$).

FUNDING

This work was funded by the UK Biotechnology and Biological Sciences Research Council and AB Vista Ltd. through IPA award BB/M022978/1, and by the Danish Ministry of Food, Agriculture and Fisheries (grant no. 3304-FVFP-08-M-07-01).

AUTHOR CONTRIBUTIONS

Conceptualization, A.M.H., C.A.B., G.D., and H.B.-P.; Methodology, A.M.H., C.A.B., G.D., and H.B.-P.; Investigation, R.F.R., Y.H.G., M.S., A.M.H., C.A.B.; Writing – Original Draft, R.F.R., A.M.H., C.A.B., Y.H.G., M.S.; Writing – Review & Editing, R.F.R., A.M.H., C.A.B., G.D., and H.B.-P.; Funding Acquisition, A.M.H., C.A.B.; Resources, A.M.H., C.A.B., G.D., and H.B.-P.; Supervision, A.M.H., C.A.B., G.D., and H.B.-P.

ACKNOWLEDGMENTS

The authors would like to thank Diamond Light Source for access to beamtime under proposal MX13467, and the staff of beamlines I03 and I04 for assistance with X-ray data collection. The molecular dynamics simulations presented in this paper were carried out on the High Performance Computing Cluster supported by the Research and Specialist Computing Support service at the University of East Anglia, Norwich, U.K. No conflict of interest is declared.

Received: November 4, 2021
Revised: February 2, 2022
Accepted: February 17, 2022
Published: February 19, 2022

REFERENCES

Acquistapace, I.M., Zi Etek, M.A., Li, A.W.H., Salmon, M., Kühn, I., Bedford, M.R., Brearley, C.A., and Hemmings, A.M. (2020). Snapshots during the catalytic cycle of a histidine acid phytase reveal an induced-fit structural mechanism. J. Biol. Chem. **295**:17724. https://doi.org/10.1074/JBC.RA120.015925.

Adams, P.D., Afonine, P.V., Bunkóczi, G., Chen, V.B., Davis, I.W., Echols, N., Headd, J.J., Hung, L.W., Kapral, G.J., Grosse-Kunstleve, R.W., et al. (2010). PHENIX: a comprehensive Python-based system for macromolecular structure solution. Acta Crystallogr. Section D: Biol. Crystallogr. **66**:213–221. https://doi.org/10.1107/S0907444909052925.

Altschul, S.F., and Gish, W. (1996). Local alignment statistics. Methods Enzymol. **266**:460–480. https://doi.org/10.1016/S0076-6879(96)66029-7.

Baker, N.A., Sept, D., Joseph, S., Holst, M.J., and McCammon, J.A. (2001). Electrostatics of nanosystems: application to microtubules and the ribosome. Proc. Natl. Acad. Sci. U S A. **98**:10037–10041. www.pnas.orgcgidoi10.1073pnas.181342398.

Blaabjerg, K., Hansen-Møller, J., and Poulsen, H.D. (2010). High-performance ion chromatography method for separation and quantification of inositol phosphates in diets and digesta. J. Chromatogr. B: Anal. Tech. Biomed. Life Sci. **878**:347–354. https://doi.org/10.1016/j.jchromb.2009.11.046.

Bohn, L., Josefsen, L., Meyer, A.S., and Rasmussen, S.K. (2007). Quantitative analysis of phytate globoids isolated from wheat bran and characterization of their sequential dephosphorylation by wheat phytase. J. Agric. Food Chem. **55**:7547–7552. https://doi.org/10.1021/jf071191t.

Bohn, L., Meyer, A.S., and Rasmussen, S.K. (2008). Phytate: impact on environment and human nutrition. A challenge for molecular breeding. J. Zhejiang Univ. Sci. 165–191. https://doi.org/10.1631/jzus.B0710640.

Bretthauer, R.K., and Castellino, F.J. (1999). Glycosylation of Pichia pastoris-derived proteins. Biotechnol. Appl. Biochem. **30**:193–200. https://doi.org/10.1111/j.1470-8744.1999.tb00770.x.

Brinch-Pedersen, H., Madsen, C.K., Inger Holme, I.B., and Dionisio, G. (2014). Increased understanding of the cereal phytase complement for better mineral bio-availability and resource management. J. Cereal Sci. 373–381. https://doi.org/10.1016/j.jcs.2013.10.003.

Brinch-Pedersen, H., Sørensen, L.D., and Holm, P.B. (2002). Engineering crop plants: getting a handle on phosphate. Trends Plant Sci. **7**:118–125. https://doi.org/10.1016/S1360-1385(01)02222-1.

Chen, Q., Luan, Z.J., Cheng, X., and Xu, J.H. (2015). Molecular dynamics investigation of the substrate binding mechanism in carboxylesterase. Biochemistry **54**:1841–1848. https://doi.org/10.1021/BI5015612.

Chu, H.M., Guo, R.T., Lin, T.W., Chou, C.C., Shr, H.L., Lai, H.L., Tang, T.Y., Cheng, K.J., Selinger, B.L., and Wang, A.H. (2004). Structures of Selenomonas ruminantium phytase in complex with persulfated

phytate: DSP phytase fold and mechanism for sequential substrate hydrolysis. Structure **12**:2015–2024. https://doi.org/10.1016/j.str.2004.08.010.

Dionisio, G., Madsen, C.K., Holm, P.B., Welinder, K.G., Jørgensen, M., Stoger, E., Arcalis, E., and Brinch-Pedersen, H. (2011). Cloning and characterization of purple acid phosphatase phytases from wheat, barley, maize, and rice. Plant Physiol. **156**:1087–1100. https://doi.org/10.1104/pp.110.164756.

Dionisio, G., Jørgensen, M., Welinder, K.G., and Brinch-Pedersen, H. (2012). Glycosylations and truncations of functional cereal phytases expressed and secreted by Pichia pastoris documented by mass spectrometry. Protein Expr. Purif. **82**:179–185. https://doi.org/10.1016/j.pep.2011.12.003.

Edgar, R.C. (2004). MUSCLE: multiple sequence alignment with high accuracy and high throughput. Nucleic Acids Res. **32**:1792–1797. https://doi.org/10.1093/nar/gkh340.

Emsley, P., Lohkamp, B., Scott, W.G., and Cowtan, K. (2010). Features and development of coot. Acta Crystallogr. D: Biol. Crystallogr. **66**:486–501. https://doi.org/10.1107/S0907444910007493.

Feder, D., McGeary, R.P., Mitić, N., Lonhienne, T., Furtado, A., Schulz, B.L., Henry, R.J., Schmidt, S., Guddat, L.W., and Schenk, G. (2020). Structural elements that modulate the substrate specificity of plant purple acid phosphatases: avenues for improved phosphorus acquisition in crops. Plant Sci. **294**:110445. https://doi.org/10.1016/j.plantsci.2020.110445.

Flanagan, J.U., Cassady, A.I., Schenk, G., Guddat, L.W., and Hume, D.A. (2006). Identification and molecular modeling of a novel, plant-like, human purple acid phosphatase. Gene **377**:12–20. https://doi.org/10.1016/J.GENE.2006.02.031.

Goodstein, D.M., Shu, S., Howson, R., Neupane, R., Hayes, R.D., Fazo, J., Mitros, T., Dirks, W., Hellsten, U., Putnam, N., et al. (2012). Phytozome: a comparative platform for green plant genomics. Nucleic Acids Res. **40**:D1178–D1186. https://doi.org/10.1093/nar/gkr944.

Grueninger-Leitch, F., D'Arcy, A., D'Arcy, B., and Chène, C. (1996). Deglycosylation of proteins for crystallization using recombinant fusion protein glycosidases. Protein Sci. **5**:2617–2622. https://doi.org/10.1002/pro.5560051224.

Guddat, L.W., McAlpine, A.S., Hume, D., Hamilton, S., de Jersey, J., and Martin, J.L. (1999). Crystal structure of mammalian purple acid phosphatase. Structure **7**:757–767. https://doi.org/10.1016/S0969-2126(99)80100-2.

Hess, B., Kutzner, C., van der Spoel, D., and Lindahl, E. (2008). GROMACS 4: algorithms for highly efficient, load-balanced, and scalable molecular simulation. J. Chem. Theor. Comput. **4**:435–447. https://doi.org/10.1021/ct700301q.

Humer, E., Schwarz, C., and Schedle, K. (2015). Phytate in pig and poultry nutrition. J. Anim. Physiol. Anim. Nutr. **99**:605–625. https://doi.org/10.1111/jpn.12258.

Iqbal, J., and Shah, S.J.A. (2018). Molecular dynamic simulations reveal structural insights into substrate and inhibitor binding modes and functionality of Ecto-Nucleoside Triphosphate Diphosphohydrolases. Scientific Rep. **8**:2581. https://doi.org/10.1038/s41598-018-20971-4.

Jacobs, P.P., Geysens, S., Vervecken, W., Contreras, R., and Callewaert, N. (2009). Engineering complex-type N-glycosylation in Pichia pastoris using GlycoSwitch technology. Nat. Protoc. **4**:58–70. https://doi.org/10.1038/nprot.2008.213.

Klabunde, T., Sträter, N., Fröhlich, R., Witzel, H., and Krebs, B. (1996). Mechanism of Fe(III)-Zn(II) purple acid phosphatase based on crystal structures. J. Mol. Biol. **259**:737–748. https://doi.org/10.1006/jmbi.1996.0354.

Koch, O., Cappel, D., Nocker, M., Jäger, T., Flohé, L., Sotriffer, C.A., and Selzer, P.M. (2013). Molecular dynamics reveal binding mode of glutathionylspermidine by trypanothione synthetase. PLoS One **8**:e56788. https://doi.org/10.1371/JOURNAL.PONE.0056788.

Kumar, S., Stecher, G., and Tamura, K. (2016). MEGA7: molecular evolutionary genetics analysis version 7.0 for bigger datasets. Mol. Biol. Evol. **33**:1870–1874. https://doi.org/10.1093/molbev/msw054.

Liang, C., Tian, J., Lam, H.M., Lim, B.L., Yan, X., and Liao, H. (2010). Biochemical and molecular characterization of PvPAP3, a novel purple acid phosphatase isolated from common bean enhancing extracellular ATP utilization. Plant Physiol. **152**:854–865. https://doi.org/10.1104/pp.109.147918.

Lim, D., Golovan, S., Forsberg, C.W., and Jia, Z. (2000). 'Crystal structures of *Escherichia coli* phytase and its complex with phytate. Nat. Struct. Biol. **7**:108–113. https://doi.org/10.1038/72371.

Lim, P.E., and Tate, M.E. (1973). The phytases. II. Properties of phytase fractions F1 and F2 from wheat bran and the myo-inositol phosphates produced by fraction F2. Biochim. Biophys. Acta **302**:316–328. https://doi.org/10.1016/0005-2744(73)90160-5.

Liu, H., and Naismith, J.H. (2008). An efficient one-step site-directed deletion, insertion, single and multiple-site plasmid mutagenesis protocol. BMC Biotechnol. **8**:91. https://doi.org/10.1186/1472-6750-8-91.

Madsen, C.K., Dionisio, G., Holme, I.B., Holm, P.B., and Brinch-Pedersen, H. (2013). High mature grain phytase activity in the Triticeae has evolved by duplication followed by neofunctionalization of the purple acid phosphatase phytase (PAPhy) gene. J. Exp. Bot. **64**:3111–3123. https://doi.org/10.1093/jxb/ert116.

Matange, N., Podobnik, M., and Visweswariah, S.S. (2015). Metallophosphoesterases: structural fidelity with functional promiscuity. Biochem. J. **467**:201–216. https://doi.org/10.1042/BJ20150028.

Nagul, E.A., McKelvie, I.D., Worsfold, P., and Kolev, S.D. (2015). The molybdenum blue reaction for the determination of orthophosphate revisited: opening the black box. Analytica Chim. Acta, 60–82. https://doi.org/10.1016/j.aca.2015.07.030.

Nakano, T., joh, T., tokumoto, E., and hayakawa, T. (1999). Purification and characterization of phytase from bran of Triticum aestivum L.cv. Nourin #61. *Food Sci. Technology Res*. S. Karger AG **5**:18–23. https://doi.org/10.3136/fstr.5.18.

Nakano, T., Joh, T., Narita, K., and Hayakawa, T. (2000). The pathway of dephosphorylation of myo-inositol hexakisphosphate by phytases from wheat bran of triticum aestivum L. cv. nourin #61. Biosci. Biotechnol. Biochem. **64**:995–1003. https://doi.org/10.1271/bbb.64.995.

Olczak, M., Morawiecka, B., and Watorek, W. (2003). Plant purple acid phosphatases - genes, structures and biological function. Acta Biochim. Pol. 1245–1256. https://doi.org/10.18388/abp.2003_3648.

Oostenbrink, C., Villa, A., Mark, A.E., and van Gunsteren, W.F. (2004). A biomolecular force field based on the free enthalpy of hydration and solvation: the GROMOS force-field parameter sets 53A5 and 53A6. J. Comput. Chem. **25**:1656–1676. https://doi.org/10.1002/jcc.20090.

Phillippy, B.Q., and Bland, J.M. (1988). Gradient ion chromatography of inositol phosphates. Anal. Biochem. **175**:162–166. https://doi.org/10.1016/0003-2697(88)90374-0.

Ravindran, V., Ravindran, G., and Sivalogan, S. (1994). Total and phytate phosphorus contents of various foods and feedstuffs of plant origin. Food Chem. **50**:133–136. https://doi.org/10.1016/0308-8146(94)90109-0.

Rivera-Solís, R.A., Peraza-Echeverria, S., Echevarría-Machado, I., and Herrera-Valencia, V.A. (2014). Chlamydomonas reinhardtii has a small family of purple acid phosphatase homologue genes that are

differentially expressed in response to phytate. Ann. Microbiol. **64**:551–559. https://doi.org/10.1007/s13213-013-0688-8.

Rusnak, F., and Reiter, T. (2000). Sensing electrons: protein phosphatase redox regulation. Trends Biochem. Sci. **25**:527–529. https://doi.org/10.1016/S0968-0004(00)01659-5.

Sasagawa, T., Matsui, M., Kobayashi, Y., Otagiri, M., Moriya, S., Sakamoto, Y., Ito, Y., Lee, C.C., Kitamoto, K., and Arioka, M. (2011). High-throughput recombinant gene expression systems in Pichia pastoris using newly developed plasmid vectors. Plasmid **65**:65–69. https://doi.org/10.1016/J.PLASMID.2010.08.004.

Schenk, G., Guddat, L.W., Ge, Y., Carrington, L.E., Hume, D.A., Hamilton, S., and de Jersey, J. (2000). Identification of mammalian-like purple acid phosphatases in a wide range of plants. Gene **250**:117–125. https://doi.org/10.1016/S0378-1119(00)00186-4.

Schenk, G., Gahan, L.R., Carrington, L.E., Mitic, N., Valizadeh, M., Hamilton, S.E., de Jersey, J., and Guddat, L.W. (2005). Phosphate forms an unusual tripodal complex with the Fe-Mn center of sweet potato purple acid phosphatase. Proc. Natl. Acad. Sci. U S A. **102**:273–278. https://doi.org/10.1073/pnas.0407239102.

Schenk, G., Elliott, T.W., Leung, E., Carrington, L.E., Mitić, N., Gahan, L.R., and Guddat, L.W. (2008). Crystal structures of a purple acid phosphatase, representing different steps of this enzyme's catalytic cycle. BMC Struct. Biol. **8**:6. https://doi.org/10.1186/1472-6807-8-6.

Schenk, G., Mitić, N., Hansonc, G.R., and Combad, P. (2013). Purple acid phosphatase: a journey into the function and mechanism of a colorful enzyme. Coord. Chem. Rev. **257**:473–482. https://doi.org/10.1016/j.ccr.2012.03.020.

Schrodinger, L.L.C. (2015). The PyMOL Molecular Graphics System (Version 1.3). https://pymol.org/2/support.html.

Selleck, C., Clayton, D., Gahan, L.R., Mitić, N., McGeary, R.P., Pedroso, M.M., Guddat, L.W., and Schenk, G. (2017). Visualization of the reaction trajectory and transition state in a hydrolytic reaction catalyzed by a metalloenzyme. Chemistry **23**:4778–4781. https://doi.org/10.1002/CHEM.201700866.

Steiner, T., Mosenthin, R., Zimmermann, B., Greinerc, R., and Rotha, S. (2007). Distribution of phytase activity, total phosphorus and phytate phosphorus in legume seeds, cereals and cereal by-products as influenced by harvest year and cultivar. Anim. Feed Sci. Technology **133**:320–334. https://doi.org/10.1016/J.ANIFEEDSCI.2006.04.007.

Sträter, N., Klabunde, T., Tucker, P., Witzel, H., and Krebs, B. (1995). Crystal structure of a purple acid phosphatase containing a dinuclear Fe(III)-Zn(II) active site. Science **268**:1489–1492. https://doi.org/10.1126/science.7770774.

The UniProt Consortium, 2017. (2017). UniProt: the universal protein knowledgebase. Nucleic Acids Res. **45**:D158–D169. https://doi.org/10.1093/nar/gkw1099.

Tsyguelnaia, I., and Doolittle, R.F. (1998). Presence of a fibronectin type III domain in a plant protein. J. Mol. Evol. **46**:612–614. https://doi.org/10.1007/PL00013148.

Veiga, N., Torres, J., Macho, I., Gómez, K., González, G., and Kremer, C. (2014). Coordination, microprotonation equilibria and conformational changes of *myo*-inositol hexakisphosphate with pertinence to its biological function. Dalton Trans. **43**:16238–16251. https://doi.org/10.1039/C4DT01350F.

Viveros, A., Centeno, C., Brenes, A., Canales, R., and Lozano, A. (2000). Phytase and acid phosphatase activities in plant feedstuffs. J. Agric. Food Chem. **48**:4009–4013. https://doi.org/10.1021/JF991126M.

Waterhouse, A.M., Procter, J.B., Martin, D.M., Clamp, M., and Barton, G.J. (2009). Sequence analysis Jalview Version 2-a multiple sequence alignment editor and analysis workbench. Bioinformatics **25**:1189–1191. https://doi.org/10.1093/bioinformatics/btp033.

Wu, J., Paudel, P., Sun, M., Joshi, S.R., Stout, L.M., Greiner, R., and Jaisi, D.P. (2015). Mechanisms and pathways of phytate degradation: evidence from oxygen isotope ratios of phosphate, HPLC, and phosphorus-31 NMR spectroscopy. Soil Sci. Soc. America J. **79**:1615–1628. https://doi.org/10.2136/sssaj2015.01.0002.

Zeng, Y.F., Ko, T.P., Lai, H.L., Cheng, Y.S., Wu, T.H., Ma, Y., Chen, C.C., Yang, C.S., Cheng, K.J., Huang, C.H., et al. (2011). Crystal structures of Bacillus alkaline phytase in complex with divalent metal ions and inositol hexasulfate. J. Mol. Biol. **409**:214–224. https://doi.org/10.1016/j.jmb.2011.03.063.

Zhu, H., Qian, W., Lu, X., Li, D., Liu, X., Liu, K., and Wang, D. (2005). Expression patterns of purple acid phosphatase genes in Arabidopsis organs and functional analysis of AtPAP23 predominantly transcribed in flower. Plant Mol. Biol. **59**:581–594. https://doi.org/10.1007/s11103-005-0183-0.

Plant Communications, Volume *3*

Supplemental information

Structure of a cereal purple acid phytase provides new insights to phytate degradation in plants

Raquel Faba-Rodriguez, Yinghong Gu, Melissa Salmon, Giuseppe Dionisio, Henrik Brinch-Pedersen, Charles A. Brearley, and Andrew M. Hemmings

SUPPLEMENTAL INFORMATION

Structure of a Cereal Purple Acid Phytase Provides New Insights to Phytate Degradation in Plants

Raquel Faba-Rodriguez[1,2], Yinghong Gu[2], Melissa Salmon[2], Giuseppe Dionisio[3], Henrik A. Brinch-Pedersen[3], Charles A. Brearley[2] and Andrew M. Hemmings[1,2,4*]

[1]School of Chemistry and [2]School of Biological Sciences, University of East Anglia, Norwich Research Park, Norwich NR4 7TJ, U.K. [3]Department of Agroecology, Research Center Flakkebjerg, Aarhus University, 4200-Slagelse, Denmark. [4]College of Food Science and Technology, Shanghai Ocean University, Shanghai 201306, China.

Running title: Crystal Structure of a Cereal Purple Acid Phytase

Contact information: a.hemmings@uea.ac.uk

CONTENTS

SUPPLEMENTAL METHODS (p.4)

LIST OF SUPPLEMENTAL TABLES (p.13)

1. Selected active site distances of the TaPAPhy_b2:PO4 complex structures.
2. Reported characteristics of plant PAPhy.
3. Estimation of kinetic parameters for $InsP_6$ hydrolysis by recombinant wheat phytase isoform b2 (TaPHY_b2) and active site mutants.
4. Purple acid phosphatase sequences used in bioinformatic analyses.
5. X-ray data collection and structure refinement statistics.
6. Comparison of active site residues of plant PAPhy.

LIST OF SUPPLEMENTAL FIGURES (p.28)

1. Anomalous difference electron density map calculated using a TaPAPhy_b2 Fe-SAD dataset collected at the iron K-edge.
2. The binuclear centre of the TaPAPhy_b2:PO4 complex in (a) product- and (b) substrate-bound states.
3. Multiple sequence alignment of PAPhys and HMW PAPs.
4. Molecular phylogenetic analysis of PAP sequences by the maximum likelihood method.
5. Inhibition of phytase activity by *myo*-inositol hexakissulfate ($InsS_6$).
6. Intermolecular interactions in the crystal structure of the TaPAPhy_b2:$InsS_6$ complex.
7. HPLC product profiles of recombinant wild type (WT) TaPAPhy_b2 and its active mutants after limited reaction against $InsP_6$.

8. HPLC product profiles of recombinant wild type (WT) TaPAPhy_b2 and its active site mutants after progressive reaction against $InsP_6$.

9. HPLC product profiles of recombinant wild type (WT) TaPAPhy_b2 and its K410A mutant after extended reaction against $InsP_6$.

10. Phytase and *p*-nitrophenyl phosphatase activities of WT TaPAPhy_b2 and its mutants.

11. Comparison of the active sites of cereal PAPhys.

12. Phytase activities of recombinant cereal PAPhy enzymes.

13. HPLC product profiles following hydrolysis of InsP6 by recombinant wheat PAPhy isoform b2 (TaPAPhy_b2).

14. HPLC product profiles following hydrolysis of InsP6 by recombinant barley PAPhy isoform a (HvPAPhy_a).

15. HPLC product profiles following hydrolysis of InsP6 by recombinant maize PAPhy isoform b (ZmPAPhy_b).

16. HPLC product profiles following hydrolysis of InsP6 by recombinant rice PAPhy isoform b (OsPAPhy_b).

17. Partial enzymatic deglycosylation of TaPAPhy_b2 using recombinant GST-Endo F1.

18. pH profile of phytase activities of recombinant wild type (WT) TaPAPhy_b2 and active site mutants.

19. Validation of metal-binding sites of TaPAPhy_b2:PO_4 structures.

SUPPLEMENTAL REFERENCES (p.67)

VALIDATION REPORTS FOR PDB ENTRIES (p. 74)

SUPPLEMENTAL METHODS

Sequence analysis. The amino acid sequences of known PAPhy were analysed and compared with those of PAPs demonstrated to lack phytase activity in order to determine key differences in addition to those described previously (Dionisio *et al.*, 2011). A total of 124 PAP sequences were analysed (Supplemental Table 4), of which 112 were collected from the UniProt database (Bateman *et al.*, 2017) and the remaining 12 were retrieved from Phytozome version 12.0 (Goodstein *et al.*, 2012) or BLASTP (Altschul and Gish, 1996) searches following the methods described by Rivera-Solís *et al.*(Rivera-Solís *et al.*, 2014). Sequence groups were created to facilitate the analysis, considering (1) reported phytase activity of the protein, (2) kingdom of life of the source organism, and (3) estimated molecular weight of the protein. In the PAPhy group, distinctions were made for characterised proteins, those predicted by sequence homology with characterised PAPhy, or sequence outliers compared to the rest of the PAPhy enzymes. A specific group was created for the microalgal PAPs, as these shared insufficient sequence conservation with the higher plant enzymes. The following numbers of sequences were contained in the resulting eight groups: (1) 29 PAPhy (23.4% of the total), of which 14 are characterised and 15 predicted; (2) 42 HMW plant PAPs (33.9%), with 2 being PAPhy outliers; (3) 13 LMW plant PAPs (10.5%); (4) 10 HMW animal PAPs (8.1%); (5) 10 LMW animal PAPs (8.1%); (6) 12 microalgal PAPs (9.7%); (7) 2 fungal PAPs (1.6%); and (8) 6 bacterial PAPs (4.8%). A multiple sequence alignment (MSA) of the PAPhy and PAP sequences was performed using the MUSCLE algorithm (Edgar, 2004) with default parameters and analysed with Jalview (Waterhouse *et al.*, 2009). A phylogenetic analysis of the PAP sequences was performed with MEGA7 (Kumar *et al.*, 2016), and a phylogenetic tree constructed using the Maximum Likelihood method with default parameters.

Production of a *OCH1::G418R* hyperglycosylation knockout of the *Pichia pastoris* KM71H strain. *Pichia pastoris* strain KM71H was chosen since it is Mut^s a phenotype of slow methanol utilization. To produce recombinant protein better compatible with subsequent deglycosylation and crystallization, a knockout construct was generated of the ORF of the gene *OCH1*, encoding a mannosyltransferase of the

cis-Golgi apparatus (XM_002489551, PAS_chr1-3_0251). The objective was that abolition of hyperglycosylation would render homogeneous the glycosylation of the recombinant product to an average of $Man_{8-14}GlcNAc_2$ (Bretthauer and Castellino, 1999; Jacobs *et al.*, 2009). The ORF was cloned using primers Pp_E12456 och1_fw 5´-TCGTCAACTATGGCGAAGGC-3´ and Pp_E12456 och1_rv 5´-TATGATGACGGACGATCGCTTA-3´. The PCR product (1299 bp) was cloned into pCR4-blunt-Topo vector (Life Technology). The resulting vector, called pCR4-OCH1, was restricted with *Bst*BI and *Sfi*I and a USER™ linker cassette (Pac_I_Bst_BI_overhang_up 5´-CGAAGCTGAGGCTTAATTAAACCTCAGCGGCCACTC-3´ and Pac_I_Sfi_I_overhang_dwn 5´-TGGCCGCTGAGGTTTAATTAAGCCTCAGCTT-3´) was introduced and ligated into it (pCR4-OCH1_Delta).

The USER™ insert was chosen to be the kanamycin resistance gene (*G418R*) obtained by PCR from the vector pKAN B alpha (Lin-Cereghino *et al.*, 2008) for which PCR was performed with the pfuCx DNA Polymerase (Stratagene) and primers G418_KAN_fw 5´-GGCTTAAUCAACTCCGAACGACCTGC-3´ G418_KAN_rv 5´-GGTTTAAUCGAGTTAGCCCTCCCACA-3´. The vector pCR4-OCH1_Delta was digested with *Pac*I and *Nt.BbvC*I enzymes and combined with the USER™ enzyme mix (New England Biolab) and treated *G418R* PCR product (1703 bp). The resulting vector pCR4-OCH1::G418R was used as template for the PCR reaction using M13 reverse and forward primers in order to get the knockout linear fragment to transform *Pichia*. *Pichia* transformation was performed according to Lin-Cereghino *et al*. (Lin-Cereghino *et al.*, 2005). Positive *OCH1::G418R* knockout colonies were checked over four rounds of plating out of single colonies with dilution in order to get rid of transient expression of the *G418R* gene. Furthermore, PCR using the *OCH1* cloning primers was used to verify the correct gene substitution: 1,299 bp was the PCR product for the escape transient expression and 2,591 bp for the correct knockout integration product.

Preparation of recombinant TaPAPhy_b2 protein samples. Recombinant TaPAPhy_b2 in fusion with an N-terminal peptide encoding the *Saccharomyces cerevisiae* α-factor secretion signal was produced from a pGAPZαA (Invitrogen) construct. This construct uses the promoter of the glyceraldehyde-3-phosphate dehydrogenase enzyme to drive the constitutive production of extracellular TaPAPhy_b2 protein in *P.pastoris*. A twenty-amino acid signal peptide and a

C-terminal seven amino acid ER-retention signal was excluded from the construct and a C-terminal 6xHis tag added. 10 μg of the construct was linearized with *Avr*II (NEB) and used for electroporation (1.8 kV, 25 μF, 200 Ω) of the engineered *P. pastoris* KM71H (*OCH1::G418R*) strain. Cells were left to recover at 28°C overnight before plating on yeast extract peptone dextrose (YPD) solid medium with 400 μg mL^{-1} Zeocin™. After four days of incubation at 28°C, the biggest colonies were transferred to fresh YPD solid medium with 400 μg mL^{-1} Zeocin™ and incubated for another two days at 28°C. Colonies were tested for the production of secreted protein in a small volume expression trial and the highest expressing colony selected.

Recombinant TaPAPhy_b2 for crystallization was obtained by growing the selected *P. pastoris* KM71H (*OCH1::G418R*) transformant with TaPAPhy_b2-pGAPZαA in 800 mL of buffered minimal glucose medium (1.34% w/v yeast nitrogen base, 2% w/v casamino acids, 2% w/v glucose, 100 mM phosphate buffer pH 5.0, 100 μg mL^{-1} kanamycin, 100 μM iron(II) sulfate, 100 μM iron(III) citrate) for five days under continuous shaking (200 rpm) at 26°C. 100 μM iron(II) sulfate and 100 μM iron(III) citrate were added to the cultures on the first two days of expression, while 200 μM iron(II) sulfate, 200 μM iron(III) citrate, 2% w/v glucose and 0.5% w/v casamino acids were added on the third day. The protein was purified from the culture media after centrifugation (11,900 x g, 20 min at 4°C) to separate the cells, followed by adjusting the pH to 8.0 with 10 M NaOH and further centrifugation to separate precipitated phosphate salts. The resulting supernatant was concentrated below 50 mL using a stirred cell (Amicon) with a regenerated cellulose ultrafiltration membrane (10 kDa NMWL; Merck) and dialysed against binding buffer for nickel-nitrilotriacetic acid (Ni-NTA) metal-affinity chromatography (50 mM Tris-HCl pH 8.0, 500 mM NaCl, 20 mM imidazole) using 3.5 kDa MWCO Spectra/Por dialysis tubing (Spectrum Labs). A 5 mL Ni-NTA Superflow cartridge (Qiagen) was used to perform metal-affinity chromatography in an ÄKTA pure chromatography system (GE Healthcare). The recombinant protein was eluted with a gradient of imidazole (20 mM-500 mM), concentrated and dialysed with a 10 kDa MWCO centrifugal filter (Merck) against 20 mM Tris-HCl pH 8.0 and deglycosylated at 4°C overnight in 1x GlycoBuffer 3 (50 mM sodium acetate pH 6.0; NEB) with 100,000 U mg^{-1} recombinant GST-Endo F1 (produced as described by Grueninger-Leitch *et al.* (Grueninger-Leitch *et al.*, 1996); Supplemental Figure 17). GST-Endo F1 was removed using a 1 mL GSTrap 4B

cartridge (GE Healthcare) and the resulting deglycosylated protein (TaPAPhy_b2d) was gel filtered through a HiLoad 16/600 Superdex 75 pg column (GE Healthcare) with a buffer containing 20 mM Tris-HCl pH 8.0 and 250 mM NaCl. TaPAPhy_b2d was concentrated and dialysed in 20 mM Tris-HCl pH 8.0 after purification. The concentration of recombinant TaPAPhy_b2 was calculated by absorbance measurement at a wavelength of 280 nm using a extinction coefficient of 113,680 $M^{-1}cm^{-1}$, calculated with the ExPaSy ProtParam online resource (Wilkins *et al.*, 1999) for the non-glycosylated protein (MW = 57.49 kDa).

Single site mutants H229A, K348A and K410A were generated using a modified version of the QuickChange™ site-directed mutagenesis method (Liu and Naismith, 2008). Mutants were verified by sequencing. The transformation, expression and purification of the mutants were performed as for the wild type enzyme.

Production of other recombinant plant PAPhys using *Pichia pastoris*. The transformation, expression and purification of HvPAPhy_a, OsPAPhy_b, ZmPAPhy_b and GmPAPhy_b was performed as for the TaPAPhy_b2 enzyme and its three mutants. The four PAPhy-pPICZα constructs linearized with DraI (NEB) were transformed into the KM71H (OCH1::G418R) *Pichia pastoris* glycoengineered strain through electroporation. Expression of the plant PAPhy enzymes was performed in 100 mL of buffered minimal glycerol/methanol medium, distributed in 250 mL conical flasks with 50 mL per flaks, for five days under continuous shaking (200 rpm) at 26°C, adding 1% (v/v) methanol and the appropriate metals daily. For the expression of the PAPhy_a isoform HvPAPhy_a, 100 µM manganese(II) sulfate and Complete Mini EDTA-free Protease inhibitor cocktail tablets (Roche) were also added to the buffered minimal methanol medium. The enzymes were harvested, purified by nickel-affinity chromatography and concentrated in the same way as the TaPAPhy_b2 using 1 mL HisTrap HP columns (GE Healthcare) at a flow rate of 1 mL min^{-1}. The nickel-affinity purified plant PAPhy enzymes were normalised to a working concentration of 20 µM and stored in 20 mM tris/HCl pH 8.0 buffer containing 30% (v/v) glycerol at -80°C.

Phosphate release assays. Enzymatic characterisation was performed with glycosylated proteins after nickel affinity chromatography purification by means of standard phosphate release assays (Nagul *et al.*, 2015) in 0.2 M acetate pH 5.5 buffer with 5 mM potassium phytate (≥ 95% purity, Sigma). The recombinant proteins were

assayed at concentrations ranging from 60 nM to 1 μM. Reactions (50 μL) were performed in 96-well plates for 15 min at room temperature with two to four replicates per condition, depending on the experiment layout. Standard curves for each assay were prepared with monopotassium phosphate. Enzyme-free buffer with $InsP_6$ and buffer only reactions were utilized to determine background absorbance of small levels of contaminant inorganic phosphate present in the $InsP_6$ substrate. The reactions were stopped with 50 μL of a colour reagent, containing four volumes of 1.5% w/v ammonium molybdate in a 5.5% v/v sulfuric acid solution and one volume of a 10.8% w/v iron(II) sulfate solution. Absorbance at λ = 700 nm was measured in a microplate reader (Hidex Sense) after colour development for 30 min.

For relative activity calculations of the mutant enzymes compared to the wild type, the enzymes were assayed at concentrations of 1 μM-100 nM and reactions were carried out with four replicates. For determination of pH optimum (Supplemental Figure 18), the following buffers were used: pH 2.0 to 3.5, 0.2 M glycine/HCL; pH 4.0 to 5.5, 0.2 M sodium acetate; pH 6.0 to 7.0, 0.2 M bis-Tris; and pH 7.5 to 8.5, 0.2 M Tris-HCl. Reactions were carried out in duplicate.

The effect of the non-hydrolysable $InsP_6$ analogue *myo*-inositol hexakissulfate ($InsS_6$, potassium salt; Alfa Chemistry) on the phytase activity of wild type TaPAPhy_b2 was tested with the standard phosphate release assay conditions described above, with 5 mM $InsP_6$ substrate and 1 μM enzyme. Reactions were performed in triplicate in the presence of increasing concentrations of $InsS_6$ (0 to 1 mM).

Kinetic parameters were estimated at the pH and temperature optima with sodium phytate (≥98% purity, Merck) as substrate and reactions in triplicate. A single timepoint (10 min for WT and K348A or 90 min for H229A and K410A) and enzyme concentration (60 nM) were chosen on the basis that, when less than 10-15% of the total substrate for each substrate concentration has been consumed during the reaction, the rate of reaction obtained can be assumed to be the initial rate. The substrate concentrations used to calculate the kinetic parameters for phytate were 0, 5, 10, 25, 50, 100, 200 and 400 μM.

Phosphate release was quantified by interpolation from linear least-squares regressions of plots of absorbance against monopotassium phosphate standards. Raw absorbance data were processed in Microsoft Excel after subtraction of

absorbances arising from $InsP_6$ and free phosphate in the $InsP_6$ substrate. In order to avoid negative values at low substrate concentrations, the data was transformed to increments of phosphate concentration released with respect to the points with 0 μM substrate. The results for each reaction were expressed as the rate of phosphate concentration released (μM) per time of the reaction (min) and amount of enzyme (0.173 μg). To estimate enzyme kinetic parameters, the data was fitted to the Michaelis-Menten equation (substrate *vs.* velocity) by performing non-linear regression with the least squares (ordinary) fit method using GraphPad Prism version 7.03 (GraphPad Software, La Jolla California USA).

HPLC separation of products of enzymatic phytate hydrolysis. Reactions were performed at room temperature in 0.2 M acetate buffer pH 5.5 with 1 μM enzyme and 1 mM sodium phytate (≥98% purity, Merck) as substrate. Reactions were stopped after 30 min by boiling at 100°C for 5 min. Reaction products were resolved by anion-exchange HPLC on a 250 x 3 mm CarboPac PA200 column (Dionex UK, Ltd) and a 50 x 3 mm guard column of the same material, injecting 20 μL of reaction per run. The elution was performed at a flow rate of 0.4 mL min^{-1} with a gradient of methanesulfonic acid delivered from solvent reservoirs containing (A) water and (B) 600 mM methane sulfonic acid according to the following programme: time (min), % B; 0, 0; 25, 100; 38, 100. The separated inositol phosphates were mixed post-column with a solution consisting of 0.1% w/v ferric nitrate in 2% w/v perchloric acid at a flow rate of 0.2 mL min^{-1} for their detection by UV absorbance at λ = 290 nm (Phillippy and Bland, 1988). Inositol phosphate standards were prepared by reflux in 1 M HCl for 24 h with subsequent rotary evaporation at 35°C to remove the HCl.

Crystallization. Crystal growth was performed at 16°C with TaPAPhy_b2d concentrated to 7-8 mg mL^{-1}. Sitting drops of total volume 0.5 μL containing the protein and reservoir solution in a 1:1 ratio in 96-well 2-drop MRC crystallisation plates (Molecular Dimensions) were equilibrated against 50 μL of reservoir solution and using a OryxNano protein crystallisation robot (Douglas Instruments). Crystals formed in drops containing 0.2 M sodium thiocyanate and 20% (w/v) PEG 3350. Single crystals were cryoprotected prior to storage in liquid nitrogen by brief soaking in a cryoprotectant solution containing 0.2 M sodium thiocyanate, 20% (w/v) PEG 3350 and 25% (v/v) PEG 400. In order to obtain the crystal structure of the

TaPAPhy_b2:$InsS_6$ complex, crystals were soaked for 4 minutes in the same cryoprotectant solution but also containing 5 mM *myo*-inositol hexakissulfate ($InsS_6$) at pH 5.5 adjusted with acetate buffer.

X-ray diffraction data collection and structure determination. X-ray data was collected at Diamond Light Source (DLS; Didcot, UK) on beamlines I03 and I04. Single-wavelength X-ray diffraction data collection was performed at a wavelength of 0.9763 Å (12.6994 keV) for native datasets and 1.7389 Å (7.1300 keV) for datasets collected at the iron edge. The 1.42 Å and 1.54 Å resolution TaPAPhy_b2:PO_4 complex datasets were collected on beamline I04 from crystals in the space group *H*3. The 1.68 Å resolution TaPAPhy_b2:$InsS_6$ complex and the 1.50 Å resolution TaPAPhy_b2-H229A datasets were collected from crystals in the same space group on beamline I03. The X-ray diffraction images collected from single crystals were scaled and integrated using the DLS automated software pipeline. Data reduction was performed with XIA2 (Winter, Lobley and Prince, 2013). The PHENIX suite (Adams *et al.*, 2010) was used for structure solution. A molecular replacement (MR) search model was generated with SCULPTOR (Bunkóczi and Read, 2011) based on the crystal structure of red kidney bean PAP (PDB ID: 2QFR (Schenk *et al.*, 2008)). The structures were solved by automated MR using PHASER (McCoy *et al.*, 2007). The MR solutions were subjected to several rounds of automatic refinement using PHENIX REFINE(Adams *et al.*, 2010) and manual refinement using COOT (Emsley *et al.*, 2010). All atoms except water were considered anisotropic in the final stages of refinement. Ligand restraints were generated with READYSET or REEL (Adams *et al.*, 2010). Metal coordination restraints were also generated with READYSET (Adams *et al.*, 2010) and included in the refinement for structures with a resolution lower than 1.60 Å. Crystal parameters, data collection and refinement statistics for the TaPAPhy_b2 structures are summarised in Supplemental Table 5. Validation of refined metal-binding sites was carried out using the CheckMyMetal web server (Zheng *et al.*, 2014) (Supplemental Figure 19). X-ray fluorescence spectra were routinely collected in order to confirm the identities of transition metals in the crystal. This was followed by X-ray absorption edge scans to test for the presence of specific metals (Fe, Mn) before collecting an Fe-SAD dataset. MR was carried out as described above, an anomalous difference electron density map was generated using tools from the PHENIX suite (Adams *et al.*, 2010) and inspected in COOT (Emsley *et al.*, 2010).

Molecular Dynamics simulations. A model of the TaPAPhy_b2:InsP$_6$ complex was obtained through molecular modelling and MD simulations. This approach utilized a modified version of the crystal structure of the TaPAPhy_b2:PO$_4$ complex resembling substrate binding containing a μ-(hydr)oxo bridge in the active site. Processing of the structure prior to the MD simulations was performed in COOT (Emsley *et al.*, 2010). Residues with side chains in alternate conformations were simplified to retain the conformation with the highest refined occupancy. The conformation of unresolved residues Asp20-Arg21-Gly22 was modelled using the MODLOOP (Fiser and Sali, 2003) and missing side chains of residues Arg11, Arg18, Glu19 and Lys224 were added as the most common rotamer for each amino acid. Solvent molecules were eliminated, retaining only the μ-(hydr)oxo bridge bound to the metals. Only one N-acetylglucosamine (NAG) molecule (i.e. the one directly bound to the protein through asparagine residues) was retained for each N-glycosylation site in order to simplify the simulation model. The simulations were performed using the GROMACS 2020.4 molecular dynamics package(Hess *et al.*, 2008) with the amber99sb-ildn force field (Oostenbrink *et al.*, 2004). The metal ions in the MI and MII sites were modelled as Fe^{3+} and Fe^{2+}, respectively, and the bridging solvent molecule modelled as a μ-oxo bridge. The metal ligand Tyr204 was modelled as a negatively charged tyrosinate residue. NAG coordinates and topology were obtained from the Automated Topology Builder (ATB) version 3.0 (Koziara *et al.*, 2014). The protonation states of histidine and aspartate residues were selected with reference to the H++ server (Gordon *et al.*, 2005). The protonation state of glutamate residues was assigned automatically. InsP$_6$ coordinates and topology were also obtained from ATB version 3.0. InsP$_6$ was modelled as $C_6H_{12}O_{24}P_6^{6-}$ at pH 5.5 according to Veiga *et al.* (Veiga *et al.*, 2014). MD simulations were carried out with restraints applied to the position of the two iron ions, the amino acid residues coordinating the irons, the μ-oxo bridge and the phosphate molecule coordinated to the metals. To generate starting coordinates for the complex the D-4-phosphate of phytate was manually docked to superimpose the active site phosphate found in the crystal structure and the remainder of the molecule rotated so as to avoid short van der Waals contacts with residues in the active site cavity. Only one orientation of the substrate was possible without violation of these non-bonded distance constraints. MD simulations in aqueous solution were then performed at a constant temperature of 298 K in a cubic box with

10 Å distance from the centre of the protein to the edge of the box. The box was solvated by the Simple Point Charge (SPC) water model, adding sodium counter ions to ensure neutral charge of the system. Prior to the unrestrained MD simulations, the systems were subjected to a maximum of 10,000 steps of energy minimisation using the steepest descent method followed by 20 ps of position-restrained MD in the NVT ensemble with force constants of 1,000 kJ mol^{-1} nm^{-2} on all protein atoms in order to equilibrate the water molecules in the solvation box. The equilibrated system was then subjected to a short production MD run of 100 ns duration. The position of the scissile D-4-phosphate group was weakly restrained during the simulation by imposing a restraining force constant 1,000 kJ mol^{-1} nm^{-2} on non-hydrogen atoms. Analysis of the MD trajectory was carried out using embedded tools in the GROMACS package. Root mean square deviation (RMSD) and root mean square fluctuations (RMSF) of the Cα atoms were calculated with the original model as a reference. A selection of atoms was made to represent the active site of the enzyme. This included all non-hydrogen atoms of the substrate and of all residues with an atom falling within 6 Å of any substrate atom. Clustering (Daura *et al.*, 1999) was performed in Gromacs using this atom selection with a cutoff of 1.0 Å revealing a total of 5 conformation clusters. The central member of the cluster with the highest population (representing 91% of the total) was taken to represent the productive enzyme-substrate complex. Minimum distances of key residues or regions of the protein to neighbouring phosphate groups of phytate were monitored during the production MD runs.

Other software. PyMOL (Schrodinger LLC, 2015) was used for the visualization of protein models and preparation of Figures. The APBS (Baker *et al.*, 2001) plug-in to PyMOL was used to calculate electrostatic potential contour maps.

SUPPLEMENTAL TABLES

Supplemental Table 1. Selected active site distances of the TaPAPhy_b2:PO_4 complex structures. Distances expressed in Å. Columns headed Product and Substrate refer to distances in the product (PDB entry 6GIT) and substrate (PDB entry 6GIZ) complexes, respectively.

From	To	Product	Substrate
Fe(III)	Fe(II)	3.57	3.45
"	Asp174 Oδ2	1.79	1.89
"	Asp201 Oδ2	2.35	2.35
"	Tyr204 O^-	1.86	1.88
"	His379 Nε2	2.75	2.42
"	μ-(hydr)oxo O	n/a	2.13
"	PO_4 O1	1.49	2.27
Fe(II)	Asp201 Oδ2	2.25	2.21
"	Asn258 Oδ1	2.18	2.13
"	His340 Nε2	2.00	2.12
"	His377 Nδ1	2.08	2.13
"	μ-(hydr)oxo O	n/a	2.24
"	PO_4 O2	2.00	2.45
PO_4 O3	His350 Nε2	2.83	2.59
PO_4 O4	His295 Nε2	2.72	3.03
"	Glu409 Oε1	2.56	2.63
μ-(hydr)oxo O	PO_4 P	n/a	2.63

Supplemental Table 2. Reported characteristics of plant PAPhy.

Length, number of amino acids in transcript; MW, molecular weight; pH_{opt}/T_{opt}, pH and temperature optima; 'np' data not provided)

Organism	Protein	Source	Length (aa) /MW (kDa)	pH_{opt}/ T_{opt} (°C)	Oligomer state	Phytase activity	References
Rice (*Oryza sativa*)	F1	Rice bran	np/66	4.4/40	Monomer	*Km* = 170 µM	(Hayakawa, Toma and Igaue)
Rice (*Oryza sativa*)	F2	Rice bran	np/68	4.6/40	Monomer	*Km* = 90 µM	(Hayakawa, Toma and Igaue)
Rye (*Secale cereale*)	np	Germinating seed	np/67	6.0/45	Monomer	*Km* = 300 µM *kcat* = 358 s^{-1}	(Greiner, Konietzny and Jany, 1998)
Wheat (*Triticum aestivum*)	PHYI	Mature grain	np/66	np	np	np	(Nakano *et al.*, 1999)
Wheat (*Triticum aestivum*)	PHYII	Mature grain	np/68	np	np	np	(Nakano *et al.*, 1999)
Barley (*Hordeum vulgare*)	P1	Germinating seed	np/66	5.0/45	Monomer	*Km* = 72 µM *kcat* = 136 s^{-1}	(Greiner, Jany and Larsson Alminger, 2000)
Barley (*Hordeum vulgare*)	P2	Mature seed	np/66	6.0/55	Monomer	*Km* = 190 µM *kcat* = 43 s^{-1}	(Greiner, Jany and Larsson Alminger, 2000)

Soybean (*Glycine max*)	GmPhy	Germinating seed	547/62.3	4.5-5/58	np	*Km* = 61 µM	(Hegeman and Grabau, 2001; Singh *et al.*, 2013)
Barrel medic (*Medicago truncatula*)	MtPHY1	Roots and leaves, recombinant	543/np	np	np	Effective phytate hydrolysis	(Xiao, Harrison and Wang, 2005; Xiao *et al.*, 2006)
Arabidopsis thaliana	AtPAP23	Recombinant	np/77.7	np	np	Weak activity	(Zhu *et al.*, 2005; Lung *et al.*, 2008)
Tobacco (*Nicotiana tabacum*)	NtPAP	Root	551/56	np	Monomer	*Km* = 14.7 µM *kcat* = 908 s-1	(Lung *et al.*, 2008)
Arabidopsis thaliana	AtPAP15	Recombinant	532/60	4.5/23-37	Monomer	Specific activity = 10 U mg^{-1} *Km* = 278 µM, *Vmax* = 13.44 U mg^{-1}	(Zhang *et al.*, 2008; Kuang *et al.*, 2009; Wang *et al.*, 2009)
Wheat (*Triticum aestivum*)	TaPAPhy_a1	Mature grain, recombinant	550/58	5.5/55	Monomer	*Km* = 35 µM, *Vmax* = 223 µmol min^{-1} mg^{-1}, *kcat* = 279 s^{-1}, *kcat/Km* = 796 x104 s^{-1} M^{-1}	(Dionisio *et al.*, 2011)
Wheat (*Triticum aestivum*)	TaPAPhy_a2	Mature grain, recombinant	549/58.6	np	Monomer	np	(Dionisio *et al.*, 2011)

Wheat (*Triticum aestivum*)	TaPAPhy_b1	Germinating seed, recombinant	538/57.4	5.0/50	Monomer	*Km* = 45 µM<br>*Vmax* = 216 µmol min^{-1} mg^{-1}<br>*kcat* = 270 s^{-1}<br>*kcat/Km* = 600 x104 s^{-1} M^{-1}	(Dionisio *et al.*, 2011)
Wheat (*Triticum aestivum*)	TaPAPhy_b2	Germinating seed, recombinant	537/57.4	np	Monomer	np	(Dionisio *et al.*, 2011, 2012)
Barley (*Hordeum vulgare*)	HvPAPhy_a	Mature grain, recombinant	544/57.8	np	Monomer	*Km* = 36 µM<br>*Vmax* = 208 µmol min^{-1} mg^{-1}<br>*kcat* = 260 s^{-1}<br>*kcat/Km* = 722 $x10^{4}$ s^{-1} M^{-1}	(Dionisio *et al.*, 2011)
Barley (*Hordeum vulgare*)	HvPAPhy_b1	Germinating seed, recombinant	536/57.2	np	Monomer	np	(Dionisio *et al.*, 2011, 2012)
Barley (*Hordeum vulgare*)	HvPAPhy_b2	Germinating seed, recombinant	537/57.2	np	Monomer	*Km* = 46 µM<br>*Vmax* = 202 µmol min^{-1} mg^{-1}<br>*kcat* = 253 s^{-1}<br>*kcat/Km* = 550 $x10^{4}$ s^{-1} M^{-1}	(Dionisio *et al.*, 2011)

Maize (*Zea mays*)	ZmPAPhy_b	Germinating seed, recombinant	544/57.4	np	Monomer	*Km* = 48 µM *Vmax* = 198 µmol min^{-1} mg^{-1} k*cat* = 248 s^{-1} *kcat/Km* = 517 x10^4 s^{-1} M^{-1}	(Dionisio *et al.*, 2011)
Rice (*Oryza sativa*)	OsPAPhy_b	Germinating seed, recombinant	539/57.5	np	Monomer	*Km* = 54 µM *Vmax* = 185 µmol min^{-1} mg^{-1} *kcat* = 231 s^{-1} *kcat/Km* = 428 x10^4 s^{-1} M^{-1}	(Dionisio *et al.*, 2011)
Mungbean (*Vigna radiata*)	VrPAP1	Germinating seed	547/62	np	np	Contains five PAP motifs and partial homology with four PAPhy motifs	(Wongkaew, Srinives and Nakasathien, 2013)
White lupin (*Lupinus albus*)	LASAP3	Germinating seed, recombinant	543/np	5.5/np	np	*Km* = 83.1 µM	(Maruyama *et al.*, 2012)
Wheat (*Triticum aestivum*)	TaPAPhy_a3	Mature grain	539/np	np	np	Gene isolated	(Madsen *et al.*, 2013)
Wheat	TaPAPhy_b3	Germinating seed	536/np	np	np	Gene isolated	(Madsen *et al.*, 2013)

(*Triticum aestivum*)							
Einkorn (*Triticum monococcum*)	TmPAPhy_a 1	Mature grain	np	np	np	Gene isolated	(Madsen *et al.*, 2013)
Einkorn (*Triticum monococcum*)	TmPAPhy_b 1	Germinating seed	np	np	np	Gene isolated	(Madsen *et al.*, 2013)
Goatgrass (*Aegilops tauschii*)	AtaPAPhy_a 1	Mature grain	np	np	np	Gene isolated	(Madsen *et al.*, 2013)
Goatgrass (*Aegilops tauschii*)	AtaPAPhy_b 1	Germinating seed	np	np	np	Gene isolated	(Madsen *et al.*, 2013)
Rye (*Secale cereale*)	ScPAPhy_a1	Mature grain	np	np	np	Gene isolated	(Madsen *et al.*, 2013)
Rye (*Secale cereale*)	ScPAPhy_a2	Mature grain	np	np	np	Gene isolated	(Madsen *et al.*, 2013)
Rye (*Secale cereale*)	ScPAPhy_b1	Germinating seed	np	np	np	Gene isolated	(Madsen *et al.*, 2013)
Red kidney bean (*Phaseolus vulgaris*)	np	Root nodules	np	np	np	Expression levels of transcript correlate with phytase activity	(Lazali *et al.*, 2013, 2014)

Soybean (*Glycine max*)	GmPAP4	Roots and recombinant	442/50.3	np	np	0.15 µM Pi h^{-1} U^{-1} (control = 0.06 µM Pi h^{-1} U^{-1})	(Kong *et al.*, 2014)
Chlamydomonas reinhardtii	CrPAP1	np	np	np	np	Gene expression induced by addition of phytate	(Rivera-Solís *et al.*, 2014)
Chlamydomonas reinhardtii	CrPAP5	np	np	np	np	Gene expression induced by addition of phytate	(Rivera-Solís *et al.*, 2014)
Trifoliate orange (Poncirus trifoliata)	PtPAP3	Germinating seed, recombinant	np/66	5.5/37	Monomer	Km = 46.2 µM $Vmax$ = 214 µmol min^{-1} mg^{-1} $kcat$ = 243 s^{-1} $kcat/Km$ = 5.49 s^{-1} $µmol^{-1}$	(Shu, Wang and Xia, 2015)

Supplemental Table 3. Estimation of kinetic parameters for $InsP_6$ hydrolysis by recombinant wheat phytase isoform b2 (TaPHY_b2) and active site mutants.

Michaelis constants (K_m) are expressed as substrate concentration (µM) for the recombinant wild type (WT) enzyme and mutants (K348A and K410A). Note that the phytase activity of the H229A mutant was insufficient to allow reliable estimation of kinetic parameters. Maximal velocities (V_{max}) are expressed as phosphate concentration released (µM) per time of reaction (min) and amount of enzyme (µg). Catalytic rate constants (*kcat*) are expressed in s^{-1}. The calculated value of the standard error is shown for each parameter. The R^2 of the curve fit is also included. Significance (Student's *t*-test) relative to WT shown in brackets where $p > 0.05$ (n.s.); $p < 0.05$ (*); $p < 0.001$ (***).

Parameter	WT	K348A	K410A
K_m / µM	76.4 ± 7.7	214.6 ± 46.6 (*)	307.6 ± 56.7 (*)
V_{max} / µM min^{-1} µg^{-1}	85.5 ± 3.1	102.1 ± 10.8 (n.s.)	11.3 ± 1.2 (***)
kcat / s^{-1}	23.8 ± 0.9	28.4 ± 3.0 (n.s.)	3.1 ± 0.3 (***)
R^2	0.98	0.96	0.98

Supplementary Table 4. Comparison of active site residues of plant PAPhy. Amino acid positions within 10 Å of the phosphate ion in the TaPAPhy_b2 crystal structure are listed. Residues in each which differ to that in TaPAPhy_b2 are shaded in lilac (TaPAPhy_a1), green (HvPAPhy_a), orange (OsPAPhy_b), yellow (ZmPAPhy_b) or pink (GmPAPhy_b). 'Motif' indicates the motif (PAPhy or PAP) in which the corresponding residue is found. 'b→a' shows residue substitutions between b and a TaPAPhy isoforms. 'Cereal→Soybean' shows residue substitutions between the cereal enzymes considered and GmPAPhy_B.

TaPAPhy_b2	TaPAPhy_b1	TaPAPhy_a1	HvPAPhy_a	OsPAPhy_b	ZmPAPhy_b	GmPAPhy_b	Motif	b → a	Cereal → Soybean
His23	His23	His23	His23	His22	His22	Val14	PAPhy 1	n/a	His → Val
Leu199	Leu199	Val199	Val199	Leu198	Leu198	Ile189	n/a	n/a	n/a
Ser203	Ser203	Cys203	Ser203	Ser202	Cys202	Thr193	PAP 2	n/a	n/a
Leu207	Leu207	Met207	Met207	Leu206	Leu206	Leu197	n/a	Leu → Met	n/a
Thr215	Thr215	Ala215	Thr215	Thr214	Ala214	Ser205	n/a	n/a	n/a
Ser221	Ser221	Ala221	Ser221	Ser220	Ala220	Ser211	PAPhy 4	n/a	n/a
Ala223	Ala223	Gly223	Gly223	Ala222	Ala222	Pro213	PAPhy 4	n/a	Ala/Gly → Pro
Lys224	Lys224	Lys224	Lys224	Asn223	Lys223	Leu214	PAPhy 4	n/a	Lys/Asn → Leu
Ser225	Ser225	Ser225	Ser225	Ser224	Ser224	Deletion	PAPhy 4	n/a	Ser → Deletion
Gln263	Gln263	Glu263	Glu263	Glu262	Gln262	Lys252	n/a	n/a	Gln/Glu → Lys
Ala306	Ala306	Ala306	Ala306	Ala305	Ser305	Ala295	n/a	n/a	n/a
Ala308	Ala308	Ala308	Ala308	Ala307	Ala307	Ile297	n/a	n/a	n/a
Ala341	Ala341	Ala341	Ala341	Ala340	Ala340	Pro330	n/a	n/a	n/a
Ser345	Ser345	Thr345	Thr345	Ser344	Thr344	Ser334	n/a	n/a	n/a
Thr346	Thr346	Thr346	Thr346	Thr345	Thr345	Ser335	n/a	n/a	n/a
Tyr347	Tyr347	Tyr347	Tyr347	Phe346	Tyr346	Tyr336	n/a	n/a	n/a
Lys348	Lys348	Lys348	Lys348	Lys347	Lys347	Glu337	n/a	n/a	Lys → Glu
Ala354	Ala354	Val354	Val354	Ala353	Ala353	Ala343	n/a	Ala → Val	n/a
Ser401	Ser401	Ser401	Ser401	Ser400	Ser400	Thr390	n/a	n/a	n/a
Thr413	Thr413	Thr413	Thr413	Thr412	Thr412	Ile402	PAPhy 5	n/a	Thr → Ile
Thr414	Thr414	Thr414	Thr414	Ser413	Ala413	Lys403	PAPhy 5	n/a	Thr/Ser/Ala → Lys
His415	His415	His415	His415	Tyr414	His414	Phe404	PAPhy 5	n/a	n/a
Asp418	Asp418	Glu418	Glu418	Glu417	Glu417	Glu407	PAPhy 5	n/a	n/a
Pro419	Pro419	Pro419	Pro419	Pro418	Ala418	Pro408	PAPhy 5	n/a	n/a
Arg421	Arg421	His421	His421	Arg420	His420	His410	PAPhy 5	n/a	n/a
Glu424	Glu424	Asp424	Asp424	Asp423	Asp423	Asp413	PAPhy 5	n/a	n/a
Met426	Met426	Arg426	Arg426	Leu425	Ala425	Leu415	PAPhy 5	n/a	n/a
Ser427	Ser427	Pro427	Pro427	Ser426	Ser426	Ser416	PAPhy 5	Ser → Pro	n/a
Thr428	Thr428	Lys428	Lys428	Thr427	Thr427	Thr417	PAPhy 5	Thr → Lys	n/a
Asp430	Asp430	Asn430	Asn430	Asp429	Asp429	Asp419	PAPhy 5	Asp → Asn	n/a
Ala431	Ala431	Ala431	Ala431	Pro430	Pro430	Pro420	PAPhy 5	n/a	n/a
Phe432	Phe432	Phe432	Phe432	Phe431	Phe431	Tyr421	PAPhy 5	n/a	n/a
Met433	Met433	Ile433	Ile433	Met432	Met432	Met422	PAPhy 5	Met → Ile	n/a

Supplemental Table 5. Purple acid phosphatase sequences used in bioinformatic analyses. Collection of the purple acid phosphatase sequences, with and without phytase activity. PAPhy, pink shading. Plant PAPs, lilac shading. Animal PAPs, orange shading. Microalgal PAPs, green shading. Fungal PAPs, yellow shading. Bacterial PAPs, blue shading. Sequences excluded during the analysis, red shading. 'n/a', not applicable. PAPhy sequences are separated according to whether characterised (PAPhy), predicted by sequence homology (Predicted PAPhy) or sequence outliers (PAPhy outlier). Plant and animal PAP sequences are separated into HMW and LMW.

Name	Organism	Group	Alternative names	UniProt ID
AtPAP15	*Arabidopsis thaliana*	PAPhy	n/a	Q9SFU3
GmPAPhy_b	*Glycine max*	PAPhy	GmPhy	Q93XG4
HvPAPhy_a	*Hordeum vulgare*	PAPhy	(Hv)P2	C4PKL2
HvPAPhy_b1	*Hordeum vulgare*	PAPhy	(Hv)P1	C4PKL3
HvPAPhy_b2	*Hordeum vulgare*	PAPhy	(Hv)P1	C4PKL4
LaPAPhy	*Lupinus albus*	PAPhy	LASAP3	D2YZL4
MtPAPhy	*Medicago truncatula*	PAPhy	MtPHY1	Q3ZFI1
NtPAPhy	*Nicotiana tabacum*	PAPhy	NtPAP	A5YBN1
OsPAPhy_b	*Oryza sativa*	PAPhy	(Os)F1, (Os)F2, OsPAP5	D6QSX9
PtPAP3	*Poncirus trifoliata*	PAPhy	n/a	V9LXK5
TaPAPhy_a1	*Triticum aestivum*	PAPhy	(Ta)PHYI	C4PKK7
TaPAPhy_b1	*Triticum aestivum*	PAPhy	n/a	C4PKK9
TaPAPhy_b2	*Triticum aestivum*	PAPhy	n/a	C4PKL0
ZmPAPhy_b	*Zea mays*	PAPhy	n/a	C4PKL6
AtaPAPhy_a1	*Aegilops tauschii*	Predicted PAPhy	n/a	F6MIX0
AtaPAPhy_b1	*Aegilops tauschii*	Predicted PAPhy	n/a	F6MIX1
PvPAPhy	*Phaseolus vulgaris*	Predicted PAPhy	n/a	V7B3Z4
ScPAPhy_a1	*Secale cereale*	Predicted PAPhy	n/a	F6MIX2
ScPAPhy_a2	*Secale cereale*	Predicted PAPhy	n/a	F6MIX4
ScPAPhy_b1	*Secale cereale*	Predicted PAPhy	n/a	F6MIX5
TaPAPhy_a2	*Triticum aestivum*	Predicted PAPhy	(Ta)PHYII	C4PKK8
TaPAPhy_a3	*Triticum aestivum*	Predicted PAPhy	n/a	F6MIW2
TaPAPhy_b3	*Triticum aestivum*	Predicted PAPhy	n/a	F6MIW6
TmPAPhy_a1	*Triticum monococcum*	Predicted PAPhy	n/a	F6MIW8
TmPAPhy_b1	*Triticum monococcum*	Predicted PAPhy	n/a	F6MIW9
VrPAPhy	*Vigna radiata*	Predicted PAPhy	VrPAP1	B5ARZ7
AtPAP23	*Arabidopsis thaliana*	PAPhy outlier	AtPAP_c	Q6TPH1
GmPAP4	*Glycine max*	PAPhy outlier	n/a	V9HXG4
AcPAP	*Allium cepa*	HMW Plant PAP	ACPEPP	Q93WP4
AlPAP15	*Arabidopsis lyrata*	HMW Plant PAP	n/a	D7L636
AoPAP32	*Anchusa officinalis*	HMW Plant PAP	n/a	Q9XF09
AtPAP10	*Arabidopsis thaliana*	HMW Plant PAP	n/a	Q9SIV9
AtPAP11	*Arabidopsis thaliana*	HMW Plant PAP	n/a	Q9SI18
AtPAP12	*Arabidopsis thaliana*	HMW Plant PAP	n/a	Q38924
AtPAP13	*Arabidopsis thaliana*	HMW Plant PAP	n/a	O48840
AtPAP20	*Arabidopsis thaliana*	HMW Plant PAP	n/a	Q9LXI7
AtPAP21	*Arabidopsis thaliana*	HMW Plant PAP	n/a	Q9LXI4
AtPAP22	*Arabidopsis thaliana*	HMW Plant PAP	n/a	Q8S340
AtPAP25	*Arabidopsis thaliana*	HMW Plant PAP	n/a	O23244
AtPAP26	*Arabidopsis thaliana*	HMW Plant PAP	n/a	Q949Y3
AtPAP5	*Arabidopsis thaliana*	HMW Plant PAP	n/a	Q9C927
AtPAP6	*Arabidopsis thaliana*	HMW Plant PAP	n/a	Q9C510

Name	Organism	Group	Alternative names	UniProt ID
GmPAP1	*Glycine max*	HMW Plant PAP	n/a	Q09131
GmPAP3	*Glycine max*	HMW Plant PAP	n/a	Q6YGT9
HvPAP_c	*Hordeum vulgare*	HMW Plant PAP	n/a	C4PKL5
IbPAP1	*Ipomoea batatas*	HMW Plant PAP	SpPAP2	Q9SE00
IbPAP2	*Ipomoea batatas*	HMW Plant PAP	SpPAP3	Q9SDZ9
IbPAP3	*Ipomoea batatas*	HMW Plant PAP	SpPAP1	Q9ZP18
LaAP1	*Lupinus albus*	HMW Plant PAP	n/a	Q93VM7
LaAP2	*Lupinus albus*	HMW Plant PAP	n/a	Q9XJ24
LlAP1	*Lupinus luteus*	HMW Plant PAP	(Ll)AP1; acPase1	Q8L5E1
LlAP2	*Lupinus luteus*	HMW Plant PAP	(Ll)AP2; acpase2	Q8L6L1
LlPPD1	*Lupinus luteus*	HMW Plant PAP	PPD1	Q8VX11
LlPPD2	*Lupinus luteus*	HMW Plant PAP	PPD2	Q8VXF6
LlPPD4	*Lupinus luteus*	HMW Plant PAP	PPD4	Q8VXF4
LpPAP	*Landoltia punctata*	HMW Plant PAP	n/a	Q9MB07
MtPAP1	*Medicago truncatula*	HMW Plant PAP	n/a	Q4KU02
NtPAP	*Nicotiana tabacum*	HMW Plant PAP	n/a	Q84KZ3
OsPAP2	*Oryza sativa*	HMW Plant PAP	n/a	Q8S505
OsPAP3	*Oryza sativa*	HMW Plant PAP	Os08g0280100	Q6ZCX8
OsPAP4	*Oryza sativa*	HMW Plant PAP	OsI_28583	B8B909
PpPAP	*Physcomitrella patens*	HMW Plant PAP	n/a	A9SPI2
PvPAP1	*Phaseolus vulgaris*	HMW Plant PAP	PvPAP_tIII	P80366
PvPAP2	*Phaseolus vulgaris*	HMW Plant PAP	KeACP; PvPAP_tIV	Q764C1
RcPAP1	*Ricinus communis*	HMW Plant PAP	RCOM_1019210	B9RWG6
RcPAP2	*Ricinus communis*	HMW Plant PAP	RCOM_0003680	B9SXP8
RcPAP3	*Ricinus communis*	HMW Plant PAP	RCOM_0003560	B9SXP6
SbPAP	*Sorghum bicolor*	HMW Plant PAP	SORBI_3007G091100	A0A1Z5R9T8
StPAP3	*Solanum tuberosum*	HMW Plant PAP	n/a	Q6J5M8
TaACP	*Triticum aestivum*	HMW Plant PAP	n/a	C4PKL1
VvPAP	*Vitis vinifera*	HMW Plant PAP	VITISV_037278	A5BGI6
ZmPAP_c	*Zea mays*	HMW Plant PAP	n/a	C4PKL7
AtPAP17	*Arabidopsis thaliana*	LMW Plant PAP	AtACP5	Q9SCX8
AtPAP3	*Arabidopsis thaliana*	LMW Plant PAP	n/a	Q8H129
AtPAP7	*Arabidopsis thaliana*	LMW Plant PAP	n/a	Q8S341
AtPAP8	*Arabidopsis thaliana*	LMW Plant PAP	n/a	Q8VYZ2
BrPAP17_1	*Brassica rapa*	LMW Plant PAP	n/a	D6MW88
GmPAP2	*Glycine max*	LMW Plant PAP	n/a	Q9LL80
IbPAP4	*Ipomoea batatas*	LMW Plant PAP	n/a	Q9LL81
LlACP3	*Lupinus luteus*	LMW Plant PAP	n/a	Q707M7
**LlPPD3**	***Lupinus luteus***	**LMW Plant PAP**	**PPD3**	**Q8VXF5**
OsPAP1	*Oryza sativa*	LMW Plant PAP	OSJNBa0023I19.10	Q7XH73
PvPAP3	*Phaseolus vulgaris*	LMW Plant PAP	n/a	D2D4J4
PvPAP4	*Phaseolus vulgaris*	LMW Plant PAP	n/a	Q9LL79
PvPAP5	*Phaseolus vulgaris*	LMW Plant PAP	n/a	E2D740
StPAP1	*Solanum tuberosum*	LMW Plant PAP	n/a	Q6J5M7
**ZmPAP**	***Zea mays***	**LMW Plant PAP**	**n/a**	**C4IZM1**
AgPAP	*Anopheles gambiae*	HMW Animal PAP	Aga_PAPL1	Q7PUN5
AmPAP	*Apis mellifera*	HMW Animal PAP	Ame_PAPL1	A0A087ZWE4
CePAP1	*Caenorhabditis elegans*	HMW Animal PAP?	CELE_F02E9.7	O01320
CePAP3	*Caenorhabditis elegans*	HMW Animal PAP	Cel_PAPL3	Q9NAM9
DmPAP1	*Drosophila melanogaster*	HMW Animal PAP	Dme_PAPL1; DmPAP_b	Q9VZ56
DmPAP2	*Drosophila melanogaster*	HMW Animal PAP	Dme_PAPL2	Q9VZ58
DmPAP3	*Drosophila melanogaster*	HMW Animal PAP	Dme_PAPL3; DmPAP_a	Q9VZ57
HsPAP7	*Homo sapiens*	HMW Animal PAP	Hsa_PAPL1; HsACP7	Q6ZNF0

Name	Organism	Group	Alternative names	UniProt ID
MmPAP7	*Mus musculus*	HMW Animal PAP	Mmu_PAPL1; MmACP7	Q8BX37
TnPAP1	*Tetraodon nigroviridis*	HMW Animal PAP	Tni_PAPL1	Q4RLR4
DrPAP1	*Danio rerio*	LMW Animal PAP	Dre_PAP1; DrACP5a	Q6DHF5
DrPAP2	*Danio rerio*	LMW Animal PAP	Dre_PAP2; DrACP5a	Q7SXT1
HsPAP5	*Homo sapiens*	LMW Animal PAP	Hsa_ACP5	P13686
MmPAP5	*Mus musculus*	LMW Animal PAP	Mmu_ACP5	Q05117
RnPAP5	*Ratus novergicus*	LMW Animal PAP	Rn_ACP5	P29288
SsPAP5	*Sus scrofa*	LMW Animal PAP	Ss_ACP5	P09889
TnPAP2	*Tetraodon nigroviridis*	LMW Animal PAP	n/a	Q4S7S5
XlPAP1	*Xenopus laevis*	LMW Animal PAP	Xla_PAP1; XlACP5	Q6GNG2
XlPAP2	*Xenopus laevis*	LMW Animal PAP	Xla_PAP2; XlACP5	Q6IP56
XtPAP5	*Xenopus tropicalis*	LMW Animal PAP	XtACP5	Q66IG6
CrPAP1	*Chlamydomonas reinhardtii*	Microalgal PAP	Cre16.g672250.t1.3	n/a
CrPAP2	*Chlamydomonas reinhardtii*	Microalgal PAP	Cre13.g578350.t1.2	n/a
CrPAP3	*Chlamydomonas reinhardtii*	Microalgal PAP	Cre11.g476700.t1.2	n/a
CrPAP4	*Chlamydomonas reinhardtii*	Microalgal PAP	Cre11.g468500.t1.3	n/a
CrPAP5	*Chlamydomonas reinhardtii*	Microalgal PAP	Cre12.g500200.t1.3	n/a
CrPAP6	*Chlamydomonas reinhardtii*	Microalgal PAP	Cre06.g259650.t1.2	n/a
MpPAP1	*Micromonas pusilla*	Microalgal PAP	MpPAP(3567)	n/a
MpPAP2	*Micromonas pusilla*	Microalgal PAP	MpPAP(48357)	n/a
MpPAP3	*Micromonas pusilla*	Microalgal PAP	MpPAP(57207)	n/a
MpPAP4	*Micromonas pusilla*	Microalgal PAP	MpPAP(146371)	n/a
OlPAP1	*Ostreococcus lucimarinus*	Microalgal PAP	OlPAP(1604)	n/a
OlPAP2	*Ostreococcus lucimarinus*	Microalgal PAP	OlPAP(2983)	n/a
AfPAP	*Aspergillus ficuum*	Fungal PAP	AphA; APase6; AfPAPhyC	Q12546
AnidPAP	*Aspergillus nidulans*	Fungal PAP	suApacA	Q92200
BcPAP	*Burkholderia cenocepacia J2315*	Bacterial PAP	BCAM1663	B4EKR2
BmaPAP	*Burkholderia mallei ATCC 23344*	Bacterial PAP	BMA0259	A0A0H2WHP3
BpsPAP	*Burkholderia pseudomallei K96243*	Bacterial PAP	BPSL0702	Q63X35
LePAP	*Lysobacter enzymogenes*	Bacterial PAP	phoA	Q05205
MbPAP	*Mycobacterium bovis AF2122/97*	Bacterial PAP	BQ2027_MB2608	A0A1R3Y2F9
MtubPAP	*Mycobacterium tuberculosis H37Rv*	Bacterial PAP	Rv2577	P9WL81

Supplemental Table 6. X-ray data collection and structure refinement statistics. Figures in brackets refer to the high resolution data bin.

Structure	Product	Substrate	$InsS_6$	H229A
**PDB entry code**	**6GIT**	**6GIZ**	**6GJ2**	**6GJA**
**Crystal parameters**				
Space group	*H*3	*H*3	*H*3	*H*3
a, b, c (Å)	126.48, 126.48, 106.80	126.73, 126.73, 107.04	126.02, 126.02, 105.91	125.98, 125.98, 106.55
α, β, γ (°)	90.00, 90.00, 120.00	90.00, 90.00, 120.00	90.00, 90.00, 120.00	90.00, 90.00, 120.00
**Data collection**				
Wavelength (Å)	0.9763	0.9763	0.9763	0.9763
Resolution (Å)	63.24-1.42 (1.44-1.42)	48.11-1.54 (1.57-1.54)	48.51-1.68 (1.71-1.68)	38.46-1.50 (1.53-1.50)
R_{merge} (%)	4.7 (50.6)	5.6 (71.4)	6.4 (118.4)	5.6 (59.1)
Rpim	0.029 (0.608)	0.036 (0.876)	0.032 (0.569)	0.036 (0.515)
CC1/2	0.999 (0.69)	0.999 (0.554)	0.999 (0.537)	0.997 (0.469)
< I/$\sigma(I)$ >	14.6 (2.4)	13.4 (1.5)	12.6 (1.3)	12.4 (1.7)
Completeness (%)	92.6 (99.4)	99.8 (99.7)	99.9 (100.0)	96.6 (75.0)
Multiplicity	3.5 (3.3)	3.4 (2.9)	5.1 (5.1)	3.2 (2.0)
$CC_{1/2}$	1.0 (0.7)	1.0 (0.5)	1.0 (0.5)	1.0 (0.4)
**Refinement**				
Total No. of atoms	5,093	4,915	4,748	4,940
Water molecules	489	433	286	443

No. of reflections	111,798	94,712	71,408	97,457
R_{work} (%)	13.2	13.6	13.4	12.8
R_{free} (%)	15.8	16.7	17.6	15.2
Wilson *B* factor (Å^2)	14.5	18.8	26.2	16.0
Anisotropy	0.135	0.131	0.24	0.062
RMS deviations				
Bonds (Å)	0.005	0.006	0.011	0.005
Angles (°)	0.838	0.896	0.838	0.833
Planes (Å)	0.006	0.005	0.006	0.006
Ramachandran plot				
Favoured (%)	97.23	96.80	96.20	96.91
Allowed (%)	2.77	3.20	3.60	3.09
Outliers (%)	0.00	0.00	0.20	0.00
Mean *B* factors (Å^2)	23.0	28.0	37.0	24.0

SUPPLEMENTAL FIGURES

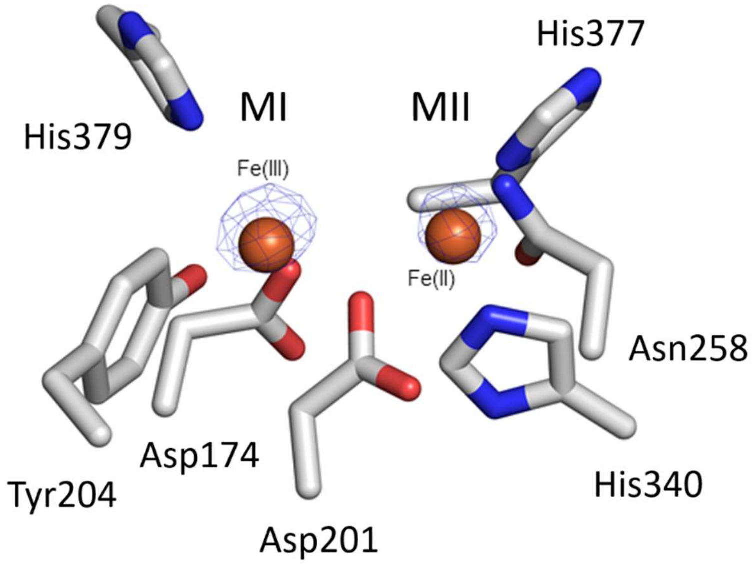

Supplemental Figure 1. Anomalous difference electron density map calculated using a TaPAPhy_b2 Fe-SAD dataset collected at the iron K-edge. Anomalous difference map calculated using data collected at a wavelengthof 1.74 Å is displayed as a blue mesh at a contour level of 10σ. Iron ions showed as brown spheres and labelled. The MI site is presumed to be in the ferric state and MII in the ferrous state. Sidechains of the residues acting as metal ligands in the active site are shown as sticks and coloured by element with carbons in grey.

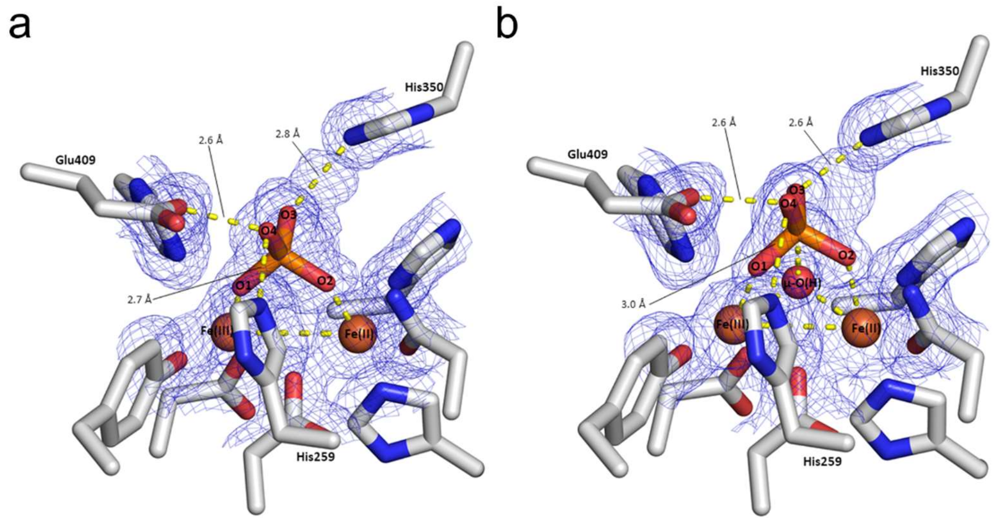

Supplemental Figure 2. The binuclear centre of the TaPAPhy_b2:PO_4 complex in (a) product- and (b) substrate-bound states. $2mF_o$-DF_c electron density maps are displayed as a blue mesh at a contour level of 1σ. Iron ions showed as brown spheres and labelled. The μ-oxo bridge is shown as a red sphere. Sidechains of the residues acting as metal ligands in the active site are shown as sticks and coloured by element with carbons in grey. Selected coordination interactions are indicated by yellow dashed lines with distances shown in Å.

Supplemental Figure 3. Multiple sequence alignment of PAPhys and HMW PAPs. PAPhy sequences are separated into groups depending on whether they have been biochemically characterised (PAPhy), predicted by sequence homology (Predicted PAPhy) or are sequence outliers (PAPhy outlier). Signal peptides are included when present in the corresponding entry from the UniProt database. Note that UniProt entry for TaPAPhy_b2 contains its signal peptide and so the residue numbering in this figure differs from that in the main paper. Specifically, subtract 20 from the residue number shown in this figure of the Supplemental Information to arrive at the corresponding number of the residue in the mature enzyme.

Horizontal groups	Vertical features
PAPhy	Signal peptide
Predicted PAPhy	PAP motifs I to V
PAPhy Outliers	PAPhy motifs 1 to 4
Plant PAPs	Proposed PAPhy motif 5

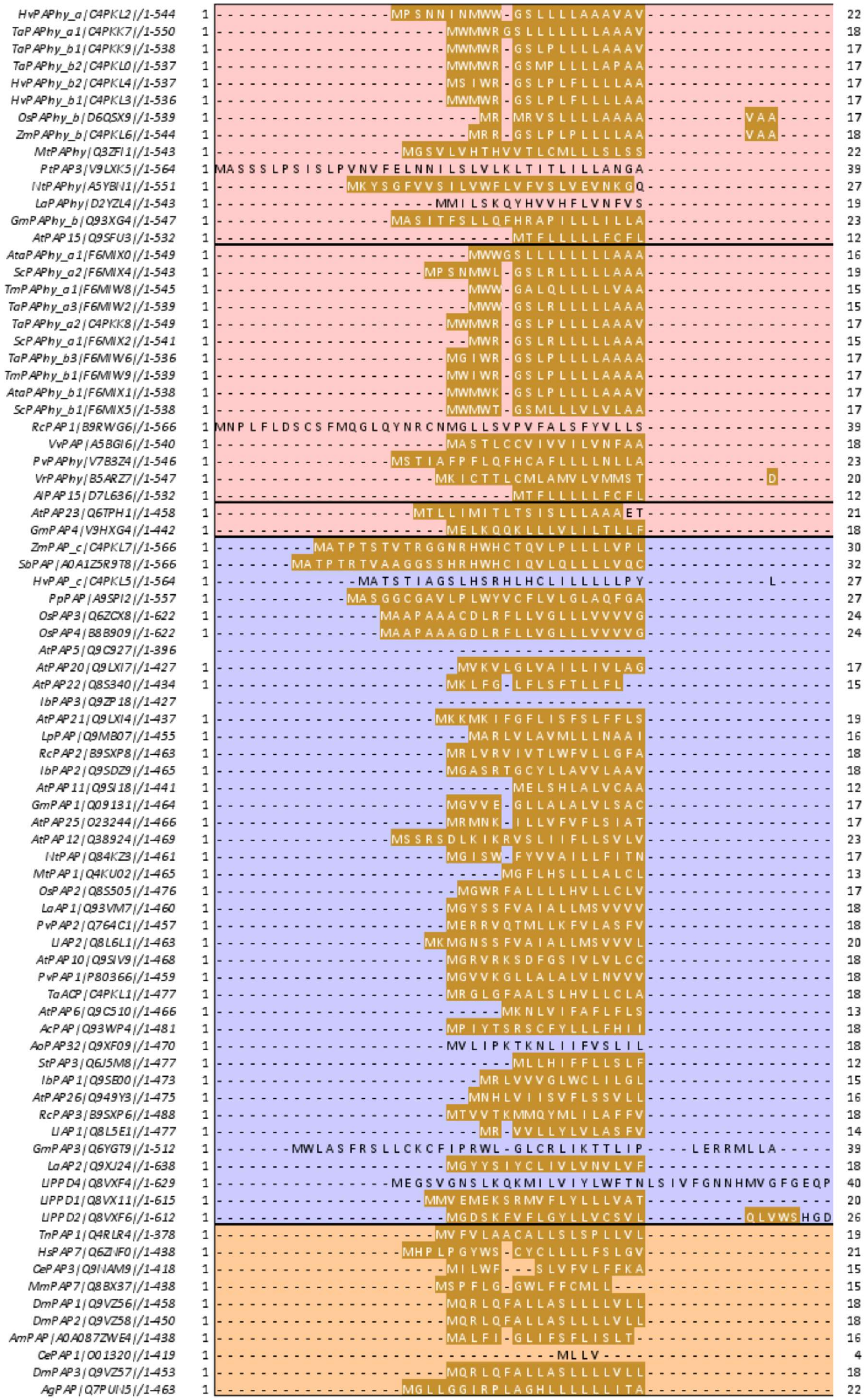

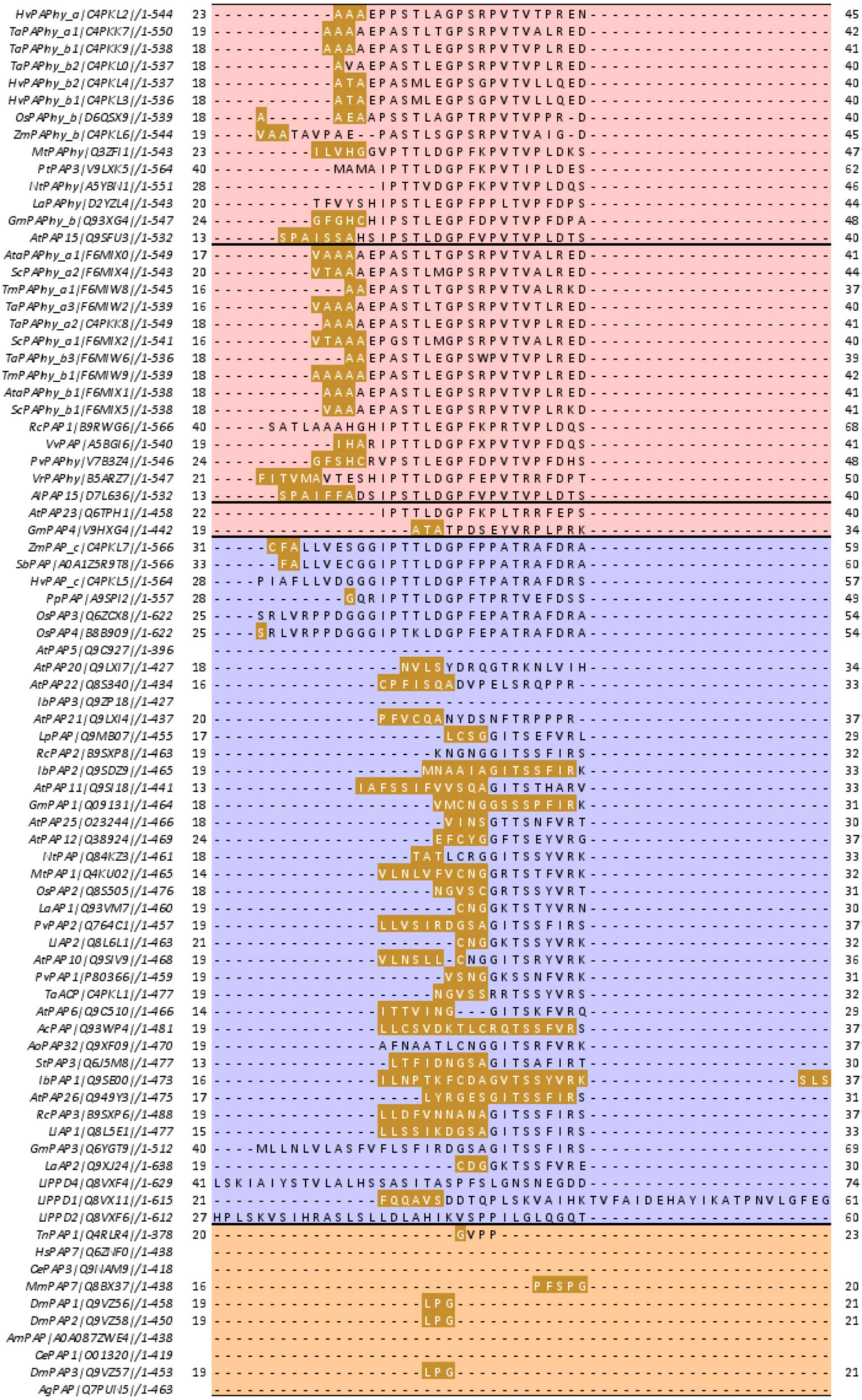
HvPAPhy_a|C4PKL2|/1-544
TaPAPhy_a1|C4PKK7|/1-550
TaPAPhy_b1|C4PKK9|/1-538
TaPAPhy_b2|C4PKL0|/1-537
HvPAPhy_b2|C4PKL4|/1-537
HvPAPhy_b1|C4PKL3|/1-536
OsPAPhy_b|D6QSX9|/1-539
ZmPAPhy_b|C4PKL6|/1-544
MtPAPhy|Q3ZFI1|/1-543
PtPAP3|V9LXK5|/1-564
NtPAPhy|A5YBN1|/1-551
LaPAPhy|D2YZL4|/1-543
GmPAPhy_b|Q93XG4|/1-547
AtPAP15|Q9SFU3|/1-532
AtaPAPhy_a1|F6MIX0|/1-549
ScPAPhy_a2|F6MIX4|/1-543
TmPAPhy_a1|F6MIW8|/1-545
TaPAPhy_a3|F6MIW2|/1-539
TaPAPhy_a2|C4PKK8|/1-549
ScPAPhy_a1|F6MIX2|/1-541
TaPAPhy_b3|F6MIW6|/1-536
TmPAPhy_b1|F6MIW9|/1-539
AtaPAPhy_b1|F6MIX1|/1-538
ScPAPhy_b1|F6MIX5|/1-538
RcPAP1|B9RWG6|/1-566
VvPAP|A5BGI6|/1-540
PvPAPhy|V7B3Z4|/1-546
VrPAPhy|B5ARZ7|/1-547
AlPAP15|D7L636|/1-532
AtPAP23|Q6TPH1|/1-458
GmPAP4|V9HXG4|/1-442
ZmPAP_c|C4PKL7|/1-566
SbPAP|A0A1Z5R9T8|/1-566
HvPAP_c|C4PKL5|/1-564
PpPAP|A9SPI2|/1-557
OsPAP3|Q6ZCX8|/1-622
OsPAP4|B8B909|/1-622
AtPAP5|Q9C927|/1-396
AtPAP20|Q9LXI7|/1-427
AtPAP22|Q8S340|/1-434
IbPAP3|Q9ZP18|/1-427
AtPAP21|Q9LXI4|/1-437
LpPAP|Q9MB07|/1-455
RcPAP2|B9SXP8|/1-463
IbPAP2|Q9SDZ9|/1-465
AtPAP11|Q9SI18|/1-441
GmPAP1|Q09131|/1-464
AtPAP25|O23244|/1-466
AtPAP12|Q38924|/1-469
NtPAP|Q84KZ3|/1-461
MtPAP1|Q4KU02|/1-465
OsPAP2|Q8S505|/1-476
LaAP1|Q93VM7|/1-460
PvPAP2|Q764C1|/1-457
LlAP2|Q8L6L1|/1-463
AtPAP10|Q9SIV9|/1-468
PvPAP1|P80366|/1-459
TaACP|C4PKL1|/1-477
AtPAP6|Q9C510|/1-466
AcPAP|Q93WP4|/1-481
AoPAP32|Q9XF09|/1-470
StPAP3|Q6J5M8|/1-477
IbPAP1|Q9SE00|/1-473
AtPAP26|Q949Y3|/1-475
RcPAP3|B9SXP6|/1-488
LlAP1|Q8L5E1|/1-477
GmPAP3|Q6YGT9|/1-512
LaAP2|Q9XJ24|/1-638
LlPPD4|Q8VXF4|/1-629
LlPPD1|Q8VX11|/1-615
LlPPD2|Q8VXF6|/1-612
TnPAP1|Q4RLR4|/1-378
HsPAP7|Q6ZNF0|/1-438
CePAP3|Q9NAM9|/1-418
MmPAP7|Q8BX37|/1-438
DmPAP1|Q9VZ56|/1-458
DmPAP2|Q9VZ58|/1-450
AmPAP|A0A087ZWE4|/1-438
CePAP1|O01320|/1-419
DmPAP3|Q9VZ57|/1-453
AgPAP|Q7PUN5|/1-463

Sequence	Start	Alignment	End
HvPAPhy_a|C4PKL2|/1-544	46	---RGHAVDLPDTDPRVQRR-ATGWAPEQV--------------------------	71
TaPAPhy_a1|C4PKK7|/1-550	43	---RGHAVDLPDTDPRVQRR-ATGWAPEQI--------------------------	68
TaPAPhy_b1|C4PKK9|/1-538	42	---RGHAVDLPDTDPRVQRR-VTGWAPEQI--------------------------	67
TaPAPhy_b2|C4PKL0|/1-537	41	---RGHAVDLPDTDPRVQRR-VTGWAPEQI--------------------------	66
HvPAPhy_b2|C4PKL4|/1-537	41	---RGHAVDLPDTDPRVQRR-VTGWAPEQI--------------------------	66
HvPAPhy_b1|C4PKL3|/1-536	41	---RGHAVDLPDTDPRVQRR-VTGWAPEQI--------------------------	66
OsPAPhy_b|D6QSX9|/1-539	41	---RGHAVDLPDTDPRVQRR-VKGWAPEQI--------------------------	66
ZmPAPhy_b|C4PKL6|/1-544	46	---RGHAVDLPDTDPRVQRR-VTGWAPEQV--------------------------	71
MtPAPhy|Q3ZFI1|/1-543	48	--FRGNAVDIPDTDPLVQRN-VEAFQPEQI--------------------------	74
PtPAP3|V9LXK5|/1-564	63	--FRGNTIDLPDTDPRVQRT-VEGFKPEQI--------------------------	89
NtPAPhy|A5YBN1|/1-551	47	--FRGHAVDLPDTDPRVQRT-VKGFEPEQI--------------------------	73
LaPAPhy|D2YZL4|/1-543	45	--LPTVSIDLPDTDPRVRRN-VHGFQPEQI--------------------------	71
GmPAPhy_b|Q93XG4|/1-547	49	--LRGVAVDLPETDPRVRRR-VRGFEPEQI--------------------------	75
AtPAP15|Q9SFU3|/1-532	41	--LRGQAIDLPDTDPRVRRR-VIGFEPEQI--------------------------	67
AtaPAPhy_a1|F6MIX0|/1-549	42	---RGHAVDLPDTDPRVQRR-ATGWAPEQI--------------------------	67
ScPAPhy_a2|F6MIX4|/1-543	45	---RGHAVDLPDTDPRVQRR-ANGWAPEQI--------------------------	70
TmPAPhy_a1|F6MIW8|/1-545	38	---RGHAVDLPDTDPRVQRR-ATGWAPEQI--------------------------	63
TaPAPhy_a3|F6MIW2|/1-539	41	---RGHAVDLPDTDPRVQRR-ATGWAPEQI--------------------------	66
TaPAPhy_a2|C4PKK8|/1-549	42	---RGHAVDLPDTDPRVQRR-VTGWAPEQI--------------------------	67
ScPAPhy_a1|F6MIX2|/1-541	41	---RGHAVDLPDTDPRVQRR-ANGWAPEQI--------------------------	66
TaPAPhy_b3|F6MIW6|/1-536	40	---RGHAVDLPDTDPRVQRR-VTGWAPEQI--------------------------	65
TmPAPhy_b1|F6MIW9|/1-539	43	---RGHAVDLPDTDPRVQRR-VTGWAPEQI--------------------------	68
AtaPAPhy_b1|F6MIX1|/1-538	42	---RGHAVDLPDTDPRVQRR-VTGWAPEQI--------------------------	67
ScPAPhy_b1|F6MIX5|/1-538	42	---RGHAVDLPDTDPRVQRR-VTGWAPEQI--------------------------	67
RcPAP1|B9RWG6|/1-566	69	--FRGHAIDLPDSDPRVQRT-VRDFEPEQI--------------------------	95
VvPAP|A5BGI6|/1-540	42	--LRGKAVDLPDTDPRVRRR-VKGFEPEQI--------------------------	68
PvPAPhy|V7B3Z4|/1-546	49	--LRGNAVDLPPSDPRVRRR-VRGFEPEQI--------------------------	75
VrPAPhy|B5ARZ7|/1-547	51	--LRRGSDDLPMTHPRLRKN-VTLNFPEQI--------------------------	77
AlPAP15|D7L636|/1-532	41	--LRGKAIDLPDTDPRVRRR-VTGFEPEQI--------------------------	67
AtPAP23|Q6TPH1|/1-458	41	--LRRGSDDLPMDHPRLRKRNVSSDFPEQI--------------------------	68
GmPAP4|V9HXG4|/1-442	35	---TLTTI--PWDSISK----AHSSYPQQV--------------------------	55
ZmPAP_c|C4PKL7|/1-566	60	--LRQGSNDVPLTDPRLAPR-VQPPAPEQI--------------------------	86
SbPAP|A0A1Z5R9T8|/1-566	61	--LRQGSDDVPLTDPRLVPR-VQPPAPEQI--------------------------	87
HvPAP_c|C4PKL5|/1-564	58	--LRRGSEDVPLSDPRLAPR-ARPPSPEQI--------------------------	84
PpPAP|A9SPI2|/1-557	50	--LRRGSVDLLPTDPRVAKT-VVGDAPEQI--------------------------	76
OsPAP3|Q6ZCX8|/1-622	55	--LRQGSDDVPLTDPRLAPR-ARPPAPEQI--------------------------	81
OsPAP4|B8B909|/1-622	55	--LRQGSDEVPITEPRLAPC-ARTPAPEQI--------------------------	81
AtPAP5|Q9C927|/1-396	1	---------MSLET--FPPP-AGYNAPEQV--------------------------	18
AtPAP20|Q9LXI7|/1-427	35	----------PTNE-------DDPTFPDQV--------------------------	47
AtPAP22|Q8S340|/1-434	34	----------PIVFVHNDRS---KSDPQQV--------------------------	50
IbPAP3|Q9ZP18|/1-427	1	--------DMPLDSDVFRVP-PGYNVPQQV--------------------------	21
AtPAP21|Q9LXI4|/1-437	38	----------PLFIVSHGRP---KFYPQQV--------------------------	54
LpPAP|Q9MB07|/1-455	30	---QESAVDMPLHADVFRMP-PGYNAPQQV--------------------------	55
RcPAP2|B9SXP8|/1-463	33	---AFPSTDIPLDDPVFASP-AGYNAPHQV--------------------------	58
IbPAP2|Q9SDZ9|/1-465	34	---VEKTVDMPLDSDVFRVP-PGYNAPQQV--------------------------	59
AtPAP11|Q9SI18|/1-441	34	---SEPSEEMSLET--FPPP-AGYNAPEQV--------------------------	57
GmPAP1|Q09131|/1-464	32	---VEKTVDMPLDSDVFAVP-PGYNAPQQV--------------------------	57
AtPAP25|O23244|/1-466	31	---AQPSTEMSLET--FPSP-AGHNAPEQV--------------------------	54
AtPAP12|Q38924|/1-469	38	---SDLPDDMPLDSDVFEVP-PGPNSPQQV--------------------------	63
NtPAP|Q84KZ3|/1-461	34	--VESSE-DMPLDSDVFRVP-HGYNAPQQV--------------------------	59
MtPAP1|Q4KU02|/1-465	33	---VEKTIDMPLDSDVFDVP-SGYNAPQQV--------------------------	58
OsPAP2|Q8S505|/1-476	32	---EYPSTDIPLESEWFAVP-NGYNAPQQV--------------------------	57
LaAP1|Q93VM7|/1-460	31	--LIEKPVDMPLDSDAFAIP-PGYNAPQQV--------------------------	57
PvPAP2|Q764C1|/1-457	38	---EWPAVDIPLDHEAFAVP-KGYNAPQQV--------------------------	63
LlAP2|Q8L6L1|/1-463	33	--LIQNPVDMPLDSDAFAIP-PGYNAPQQV--------------------------	59
AtPAP10|Q9SIV9|/1-468	37	---LEATVDMPLDSDVFRVP-CGYNAPQQV--------------------------	62
PvPAP1|P80366|/1-459	32	---TNKNRDMPLDSDVFRVP-PGYNAPQQV--------------------------	57
TaACP|C4PKL1|/1-477	33	---EFPSTDMPLDSEWFATP-KGYNAPQQV--------------------------	58
AtPAP6|Q9C510|/1-466	30	---ALPSIEMSLDT--FPSP-GGYNTPEQV--------------------------	53
AcPAP|Q93WP4|/1-481	38	---EFPAVDIPIDSKEFAVP-KNQFSPQQV--------------------------	63
AoPAP32|Q9XF09|/1-470	38	---LAAATDMPLNSDVFRVP-PGYNAPQQV--------------------------	63
StPAP3|Q6J5M8|/1-477	31	---QFPSVDIPLENEVLSVP-NGYNAPQQV--------------------------	56
IbPAP1|Q9SE00|/1-473	38	ALPNAEDVDMPWDSDVFAVP-SGYNAPQQV--------------------------	66
AtPAP26|Q949Y3|/1-475	32	---EWPAVDIPLDHHVFKVP-KGYNAPQQV--------------------------	57
RcPAP3|B9SXP6|/1-488	38	---EWPSIDIPLDNEVFAVP-KGYNAPQQV--------------------------	63
LlAP1|Q8L5E1|/1-477	34	---EFPSTDIPLDHEVFAVP-KGYNAPQQV--------------------------	59
GmPAP3|Q6YGT9|/1-512	70	---EWPAVDIPLDHEAFAVP-KGYNAPQQV--------------------------	95
LaAP2|Q9XJ24|/1-638	31	---SERAIDMALDSDVFHVP-RGYNAPQQV--------------------------	56
LlPPD4|Q8VXF4|/1-629	75	--TDWVTVELESPKPSIDDW-VGVFSPAKFDSETCPGTENHVGHIEAPYVCTAPIK	127
LlPPD1|Q8VX11|/1-615	62	HYTEWVTLQYSNNKPSIDDW-IGVFSPANFSASTCPGENKMT---NPPFLCSAPIK	113
LlPPD2|Q8VXF6|/1-612	61	--AEWVTLEYSSPIPSIDDW-IGVFSPSNFSASACPAENRRV---YPPLLCSAPIK	110
TnPAP1|Q4RLR4|/1-378	24	----------TRT------------QPEQV--------------------------	31
HsPAP7|Q6ZNF0|/1-438	22	--------QGSLGAPSA--------APEQV--------------------------	35
CePAP3|Q9NAM9|/1-418	16	----------SD-----------GKAVEQV--------------------------	24
MmPAP7|Q8BX37|/1-438	21	--VQG-----AQEYPHV--------TPEQI--------------------------	35
DmPAP1|Q9VZ56|/1-458	22	----------IRSTPIDQDVDIVHYQPEQV--------------------------	41
DmPAP2|Q9VZ58|/1-450	22	----------IRSTPIDQDVDIVHYQPEQV--------------------------	41
AmPAP|A0A087ZWE4|/1-438	17	----------VCN--------VIYYQPEAV--------------------------	28
CePAP1|O01320|/1-419	5	--------DEKLEKRSSSSS-LDRFLPDLP--------------------------	25
DmPAP3|Q9VZ57|/1-453	22	----------IRSTPIDQDVDIVHYQPEQV--------------------------	41
AgPAP|Q7PUN5|/1-463	23	----------CNGQ-------VFYYQPEQV--------------------------	35

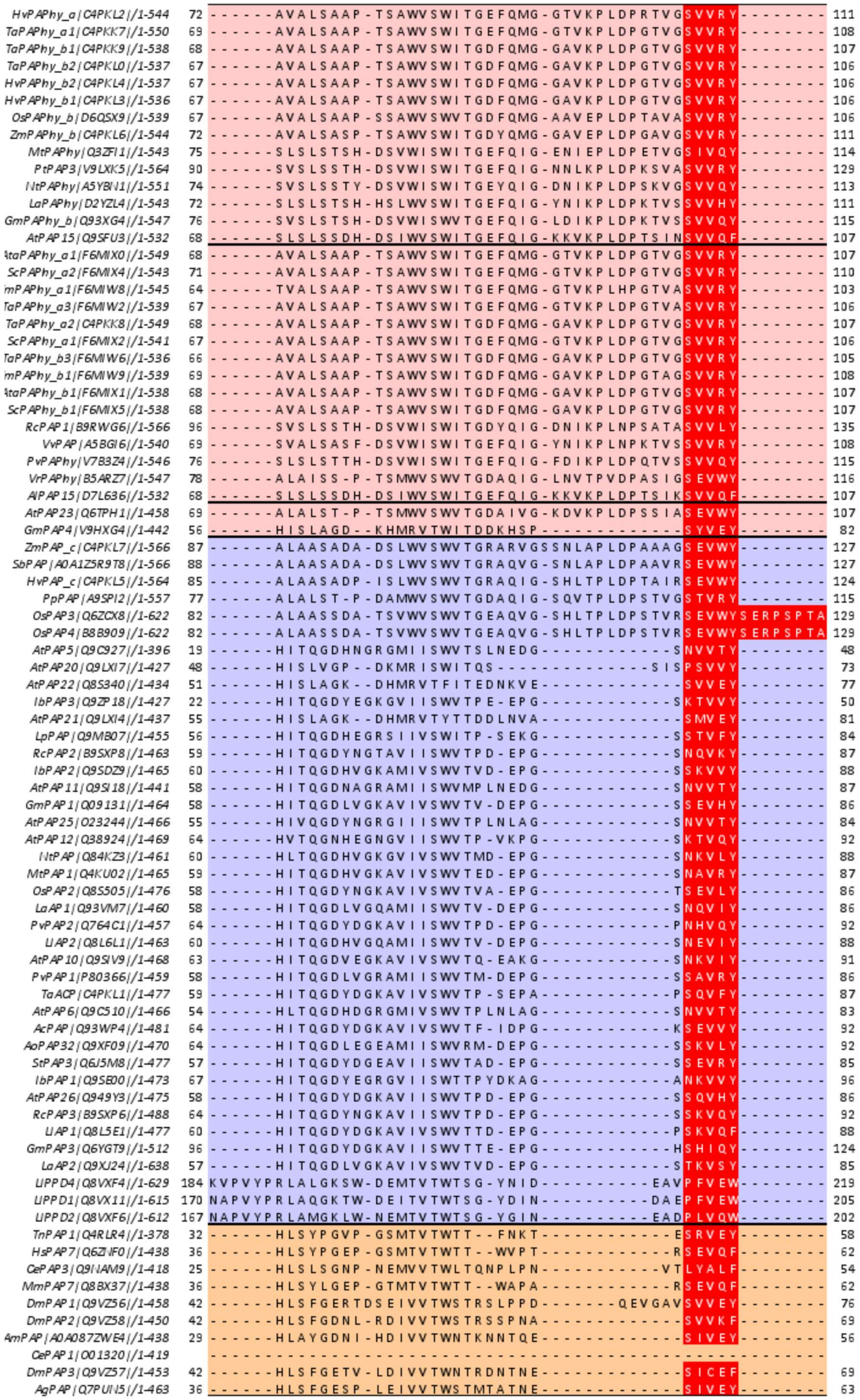

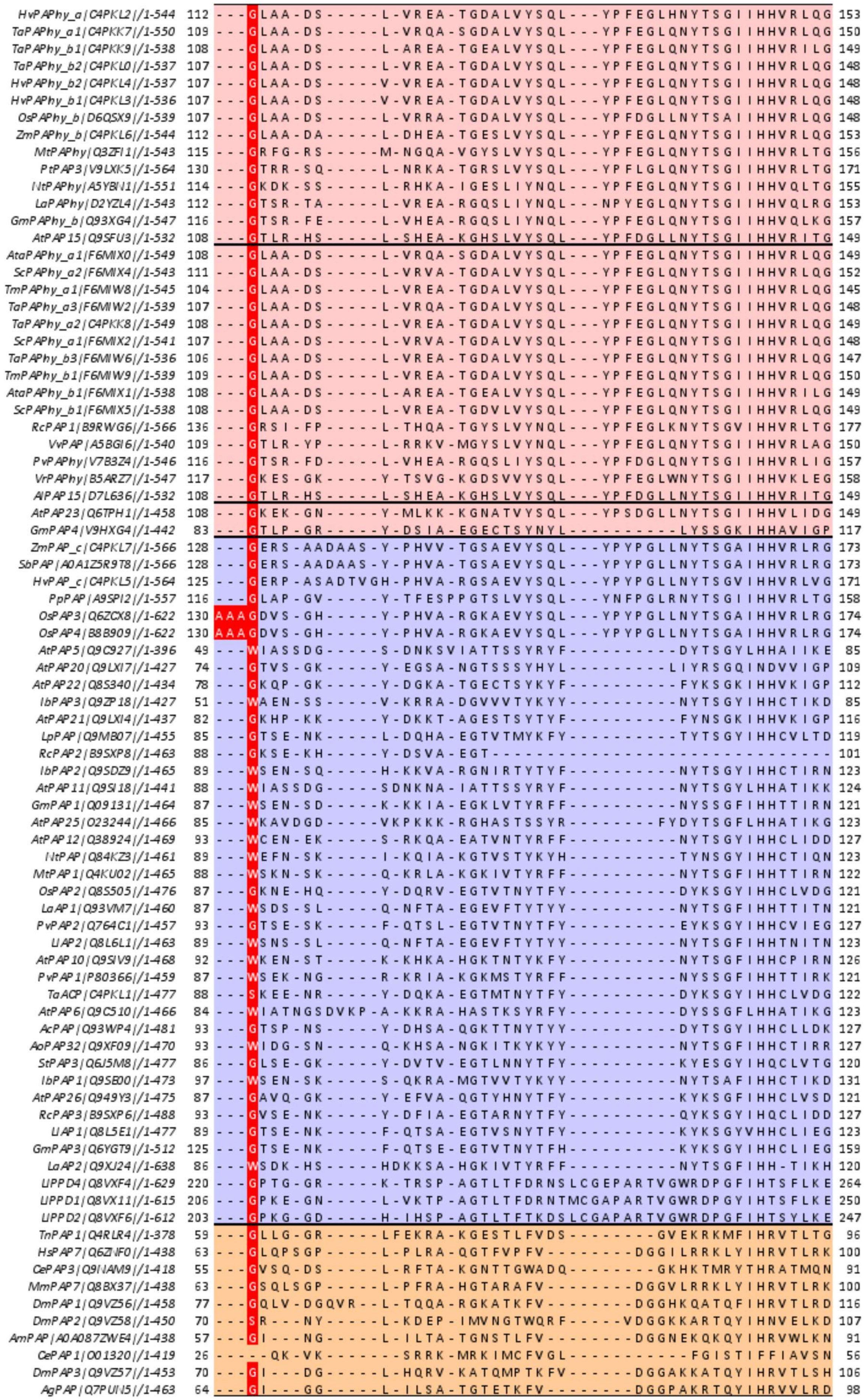

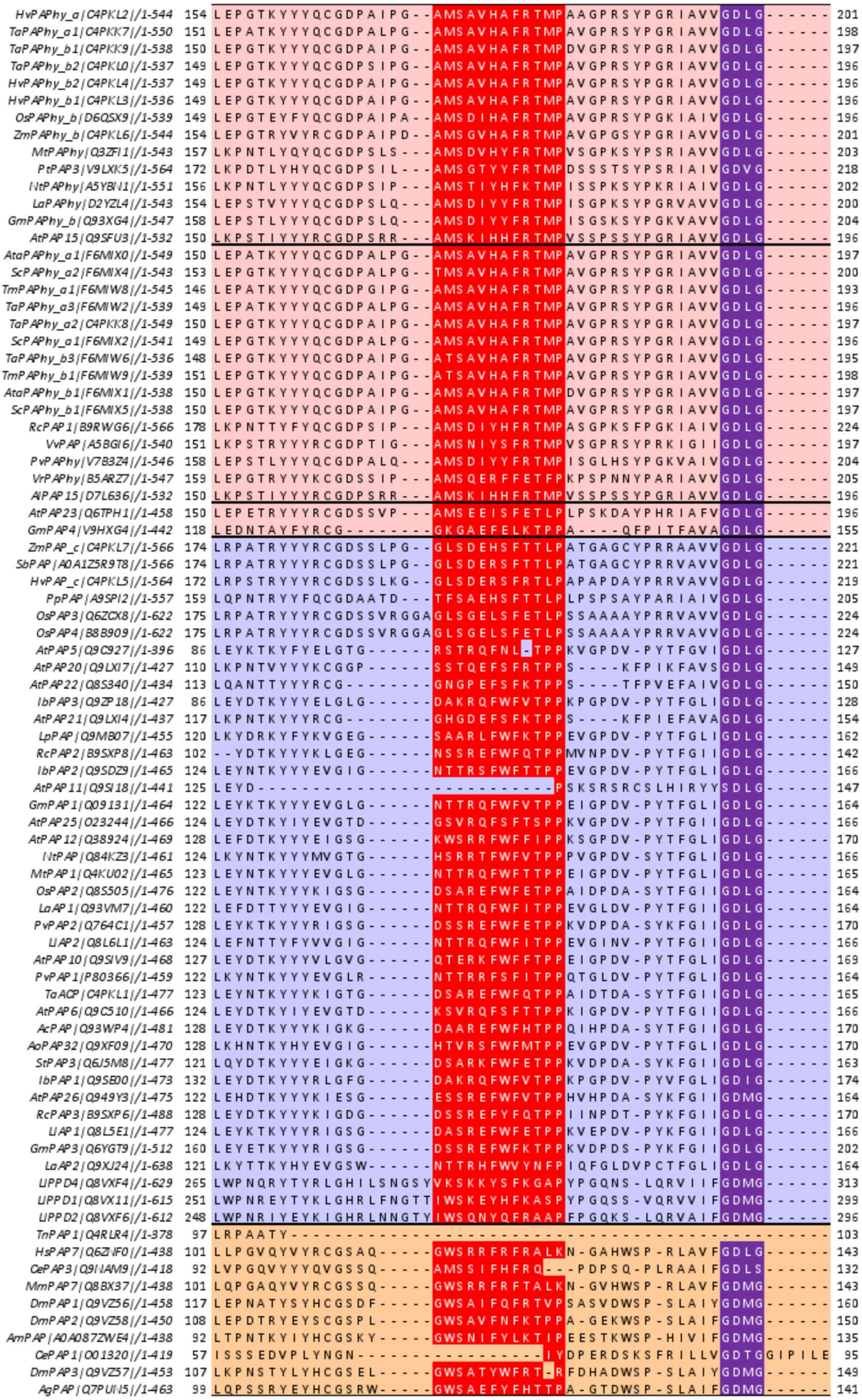

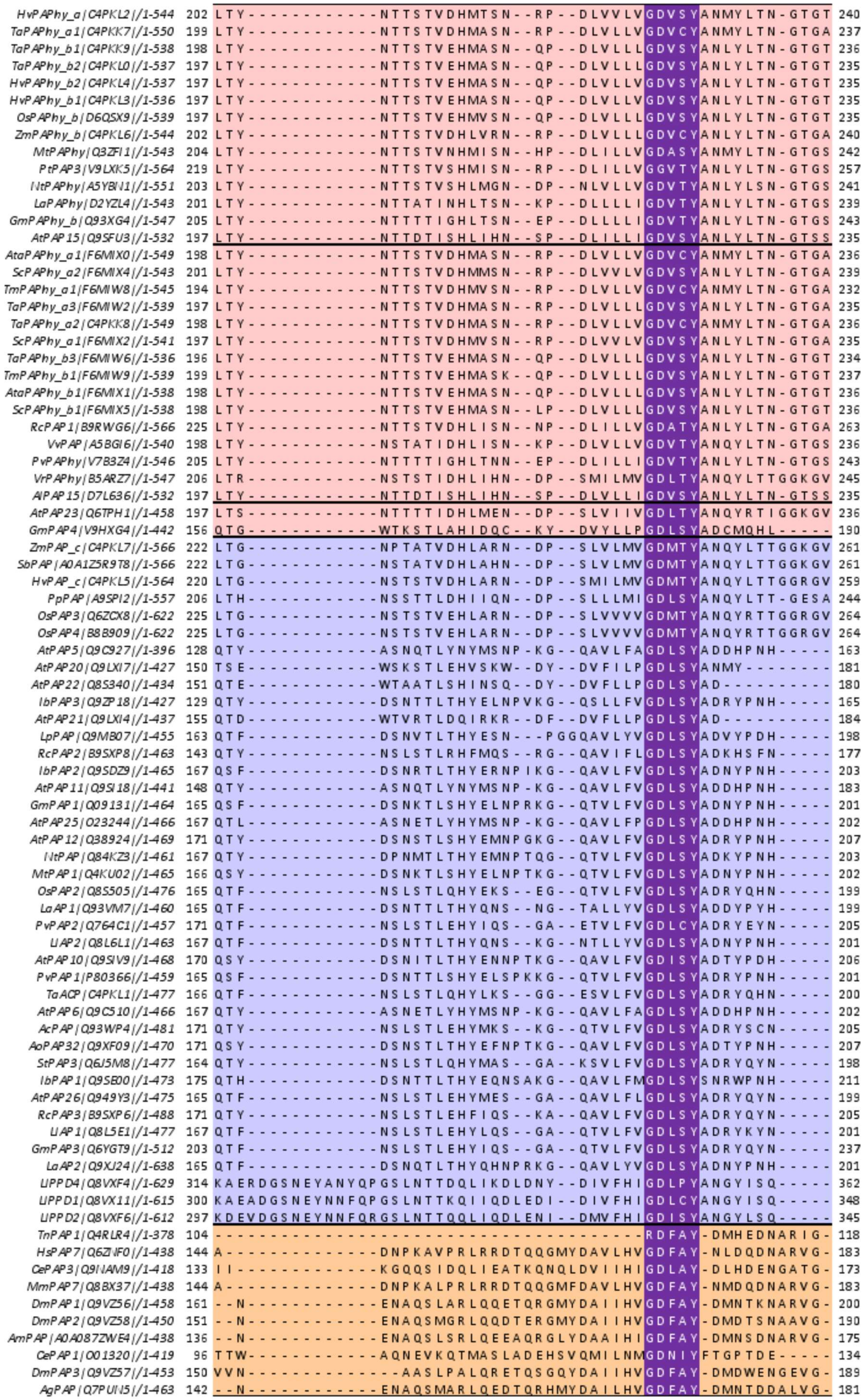
HvPAPhy_a|C4PKL2|/1-544 202 LTY------------NTTSTVDHMTSN--RP--DLVVLVGDVSYANMYLTN-GTGT 240
TaPAPhy_a1|C4PKK7|/1-550 199 LTY------------NTTSTVDHMASN--RP--DLVLLVGDVCYANMYLTN-GTGA 237
TaPAPhy_b1|C4PKK9|/1-538 198 LTY------------NTTSTVEHMASN--QP--DLVLLLGDVSYANLYLTN-GTGT 236
TaPAPhy_b2|C4PKL0|/1-537 197 LTY------------NTTSTVEHMASN--QP--DLVLLLGDVSYANLYLTN-GTGT 235
HvPAPhy_b2|C4PKL4|/1-537 197 LTY------------NTTSTVEHMASN--QP--DLVLLVGDVSYANLYLTN-GTGT 235
HvPAPhy_b1|C4PKL3|/1-536 197 LTY------------NTTSTVEHMASN--QP--DLVLLVGDVSYANLYLTN-GTGT 235
OsPAPhy_b|D6QSX9|/1-539 197 LTY------------NTTSTVEHMVSN--QP--DLVLLLGDVSYANLYLTN-GTGT 235
ZmPAPhy_b|C4PKL6|/1-544 202 LTY------------NTTSTVDHLVRN--RP--DLVLLLGDVCYANLYLTN-GTGA 240
MtPAPhy|Q3ZFI1|/1-543 204 LTY------------NTTSTVNHMISN--HP--DLILLVGDASYANMYLTN-GTGS 242
PtPAP3|V9LXK5|/1-564 219 LTY------------NTTSTVSHMISN--RP--DLILLVGGVTYANLYLTN-GTGS 257
NtPAPhy|A5YBN1|/1-551 203 LTY------------NTTSTVSHLMGN--DP--NLVLLVGDVTYANLYLSN-GTGS 241
LaPAPhy|D2YZL4|/1-543 201 LTY------------NTTATINHLTSN--KP--DLLLLIGDVTYANLYLTN-GTGS 239
GmPAPhy_b|Q93XG4|/1-547 205 LTY------------NTTTTIGHLTSN--EP--DLLLLIGDVTYANLYLTN-GTGS 243
AtPAP15|Q9SFU3|/1-532 197 LTY------------NTTDTISHLIHN--SP--DLILLIGDVSYANLYLTN-GTSS 235
AtaPAPhy_a1|F6MIX0|/1-549 198 LTY------------NTTSTVDHMASN--RP--DLVLLVGDVCYANMYLTN-GTGA 236
ScPAPhy_a2|F6MIX4|/1-543 201 LTY------------NTTSTVDHMMSN--RP--DLVVLVGDVSYANLYLTN-GTGA 239
TmPAPhy_a1|F6MIW8|/1-545 194 LTY------------NTTSTVDHMVSN--RP--DLVLLVGDVCYANMYLTN-GTGA 232
TaPAPhy_a3|F6MIW2|/1-539 197 LTY------------NTTSTVDHMASN--RP--DLVLLLGDVSYANLYLTN-GTGA 235
TaPAPhy_a2|C4PKK8|/1-549 198 LTY------------NTTSTVDHMASN--RP--DLVLLVGDVCYANMYLTN-GTGA 236
ScPAPhy_a1|F6MIX2|/1-541 197 LTY------------NTTSTVDHMVSN--RP--DLVVLVGDVSYANLYLTN-GTGA 235
TaPAPhy_b3|F6MIW6|/1-536 196 LTY------------NTTSTVEHMASN--QP--DLVLLLGDVSYANLYLTN-GTGT 234
TmPAPhy_b1|F6MIW9|/1-539 199 LTY------------NTTSTVEHMASK--QP--DLVLLLGDVSYANLYLTN-GTGT 237
AtaPAPhy_b1|F6MIX1|/1-538 198 LTY------------NTTSTVEHMASN--QP--DLVLLLGDVSYANLYLTN-GTGT 236
ScPAPhy_b1|F6MIX5|/1-538 198 LTY------------NTTSTVEHMASN--LP--DLVLLLGDVSYANLYLTN-GTGT 236
RcPAP1|B9RWG6|/1-566 225 LTY------------NTTSTVDHLISN--NP--DLILLVGDATYANLYLTN-GTGA 263
VvPAP|A5BGI6|/1-540 198 LTY------------NSTATIDHLISN--KP--DLVLLVGDVTYANQYLTN-GTGS 236
PvPAPhy|V7B3Z4|/1-546 205 LTY------------NTTTTIGHLTNN--EP--DLILLIGDVTYANLYLTN-GTGS 243
VrPAPhy|B5ARZ7|/1-547 206 LTR------------NSTSTIDHLIHN--DP--SMILMVGDLTYANQYLTTGGKGV 245
AlPAP15|D7L636|/1-532 197 LTY------------NTTDTISHLIHN--SP--DLVLLIGDVSYANLYLTN-GTSS 235
AtPAP23|Q6TPH1|/1-458 197 LTS------------NTTTTIDHLMEN--DP--SLVIIVGDLTYANQYRTIGGKGV 236
GmPAP4|V9HXG4|/1-442 156 QTG------------WTKSTLAHIDQC--KY--DVYLLPGDLSYADCMQHL----- 190
ZmPAP_c|C4PKL7|/1-566 222 LTG------------NPTATVDHLARN--DP--SLVLMVGDMTYANQYLTTGGKGV 261
SbPAP|A0A1Z5R9T8|/1-566 222 LTG------------NSTATVDHLAHN--DP--SLVLMVGDMTYANQYLTTGGKGV 261
HvPAP_c|C4PKL5|/1-564 220 LTG------------NSTSTVDHLARN--DP--SMILMVGDMTYANQYLTTGGRGV 259
PpPAP|A9SPI2|/1-557 206 LTH------------NSSTTLDHIIQN--DP--SLLLMIGDLSYANQYLTT-GESA 244
OsPAP3|Q6ZCX8|/1-622 225 LTG------------NSTSTVEHLARN--DP--SLVVVVGDMTYANQYRTTGGRGV 264
OsPAP4|B8B909|/1-622 225 LTG------------NSTSTVEHLARN--DP--SLVVVVGDMTYANQYRTTGGRGV 264
AtPAP5|Q9C927|/1-396 128 QTY------------ASNQTLYNYMSNP-KG--QAVLFAGDLSYADDHPNH----- 163
AtPAP20|Q9LXI7|/1-427 150 TSE------------WSKSTLEHVSKW--DY--DVFILPGDLSYANMY-------- 181
AtPAP22|Q8S340|/1-434 151 QTE------------WTAATLSHINSQ--DY--DVFLLPGDLSYAD---------- 180
IbPAP3|Q9ZP18|/1-427 129 QTY------------DSNTTLTHYELNPVKG--QSLLFVGDLSYADRYPNH----- 165
AtPAP21|Q9LXI4|/1-437 155 QTD------------WTVRTLDQIRKR--DF--DVFLLPGDLSYAD---------- 184
LpPAP|Q9MB07|/1-455 163 QTF------------DSNVTLTHYESN---PGGQAVLYVGDLSYADVYPDH----- 198
RcPAP2|B9SXP8|/1-463 143 QTY------------NSLSTLRHFMQS--RG--QAVIFLGDLSYADKHSFN----- 177
IbPAP2|Q9SDZ9|/1-465 167 QSF------------DSNRTLTHYERNPIKG--QAVLFVGDLSYADNYPNH----- 203
AtPAP11|Q9SI18|/1-441 148 QTY------------ASNQTLYNYMSNP-KG--QAVLFVGDLSYADDHPNH----- 183
GmPAP1|Q09131|/1-464 165 QSF------------DSNKTLSHYELNPRKG--QTVLFVGDLSYADNYPNH----- 201
AtPAP25|O23244|/1-466 167 QTL------------ASNETLYHYMSNP-KG--QAVLFPGDLSYADDHPNH----- 202
AtPAP12|Q38924|/1-469 171 QTY------------DSNSTLSHYEMNPGKG--QAVLFVGDLSYADRYPNH----- 207
NtPAP|Q84KZ3|/1-461 167 QTY------------DPNMTLTHYEMNPTQG--QTVLFVGDLSYADKYPNH----- 203
MtPAP1|Q4KU02|/1-465 166 QSY------------DSNKTLSHYELNPTKG--QTVLFVGDLSYADNYPNH----- 202
OsPAP2|Q8S505|/1-476 165 QTF------------NSLSTLQHYEKS--EG--QTVLFVGDLSYADRYQHN----- 199
LaAP1|Q93VM7|/1-460 165 QTF------------DSNTTLTHYQNS--NG--TALLYVGDLSYADDYPYH----- 199
PvPAP2|Q764C1|/1-457 171 QTF------------NSLSTLEHYIQS--GA--ETVLFVGDLCYADRYEYN----- 205
LlAP2|Q8L6L1|/1-463 167 QTF------------DSNTTLTHYQNS--KG--NTLLYVGDLSYADNYPNH----- 201
AtPAP10|Q9SIV9|/1-468 170 QSY------------DSNITLTHYENNPTKG--QAVLFVGDISYADTYPDH----- 206
PvPAP1|P80366|/1-459 165 QSF------------DSNTTLSHYELSPKKG--QTVLFVGDLSYADRYPNH----- 201
TaACP|C4PKL1|/1-477 166 QTF------------NSLSTLQHYLKS--GG--ESVLFVGDLSYADRYQHN----- 200
AtPAP6|Q9C510|/1-466 167 QTY------------ASNETLYHYMSNP-KG--QAVLFAGDLSYADDHPNH----- 202
AcPAP|Q93WP4|/1-481 171 QTY------------NSLSTLEHYMKS--KG--QTVLFVGDLSYADRYSCN----- 205
AoPAP32|Q9XF09|/1-470 171 QSY------------DSNSTLTHYEFNPTKG--QAVLFVGDLSYADTYPNH----- 207
StPAP3|Q6J5M8|/1-477 164 QTY------------NSLSTLQHYMAS--GA--KSVLFVGDLSYADRYQYN----- 198
IbPAP1|Q9SE00|/1-473 175 QTH------------DSNTTLTHYEQNSAKG--QAVLFMGDLSYSNRWPNH----- 211
AtPAP26|Q949Y3|/1-475 165 QTF------------NSLSTLEHYMES--GA--QAVLFLGDLSYADRYQYN----- 199
RcPAP3|B9SXP6|/1-488 171 QTY------------NSLSTLEHFIQS--KA--QAVLFVGDLSYADRYQYN----- 205
LlAP1|Q8L5E1|/1-477 167 QTF------------NSLSTLEHYLQS--GA--QTVLFVGDLSYADRYKYN----- 201
GmPAP3|Q6YGT9|/1-512 203 QTF------------NSLSTLEHYIQS--GA--QTVLFVGDLSYADRYQYN----- 237
LaAP2|Q9XJ24|/1-638 165 QTF------------DSNQTLTHYQHNPRKG--QAVLYVGDLSYADNYPNH----- 201
LlPPD4|Q8VXF4|/1-629 314 KAERDGSNEYANYQPGSLNTTDQLIKDLDNY--DIVFHIGDLPYANGYISQ----- 362
LlPPD1|Q8VX11|/1-615 300 KAEADGSNEYNNFQPGSLNTTKQIIQDLEDI--DIVFHIGDLCYANGYISQ----- 348
LlPPD2|Q8VXF6|/1-612 297 KDEVDGSNEYNNFQRGSLNTTQQLIQDLENI--DMVFHIGDISYANGYLSQ----- 345
TnPAP1|Q4RLR4|/1-378 104 ---------------------------------------RDFAY-DMHEDNARIG- 118
HsPAP7|Q6ZNF0|/1-438 144 A--------------DNPKAVPRLRRDTQQGMYDAVLHVGDFAY-NLDQDNARVG- 183
CePAP3|Q9NAM9|/1-418 133 II-------------KGQQSIDQLIEATKQNQLDVIIHIGDLAY-DLHDENGATG- 173
MmPAP7|Q8BX37|/1-438 144 A--------------DNPKALPRLRRDTQQGMFDAVLHVGDFAY-NMDQDNARVG- 183
DmPAP1|Q9VZ56|/1-458 161 --N------------ENAQSLARLQQETQRGMYDAIIHVGDFAY-DMNTKNARVG- 200
DmPAP2|Q9VZ58|/1-450 151 --N------------ENAQSMGRLQQDTERGMYDAIIHVGDFAY-DMDTSNAAVG- 190
AmPAP|A0A087ZWE4|/1-438 136 --N------------ENAQSLSRLQEEAQRGLYDAAIHIGDFAY-DMNSDNARVG- 175
CePAP1|O01320|/1-419 96 TTW------------AQNEVKQTMASLADEHSVQMILNMGDNIYFTGPTDE----- 134
DmPAP3|Q9VZ57|/1-453 150 VVN--------------AASLPALQRETQSGQYDAIIHVGDFAY-DMDWENGEVG- 189
AgPAP|Q7PUN5|/1-463 142 --N------------ENAQSMARLQEDTQRHMYDAILHVGDFAY-DMNTDDALVG- 181

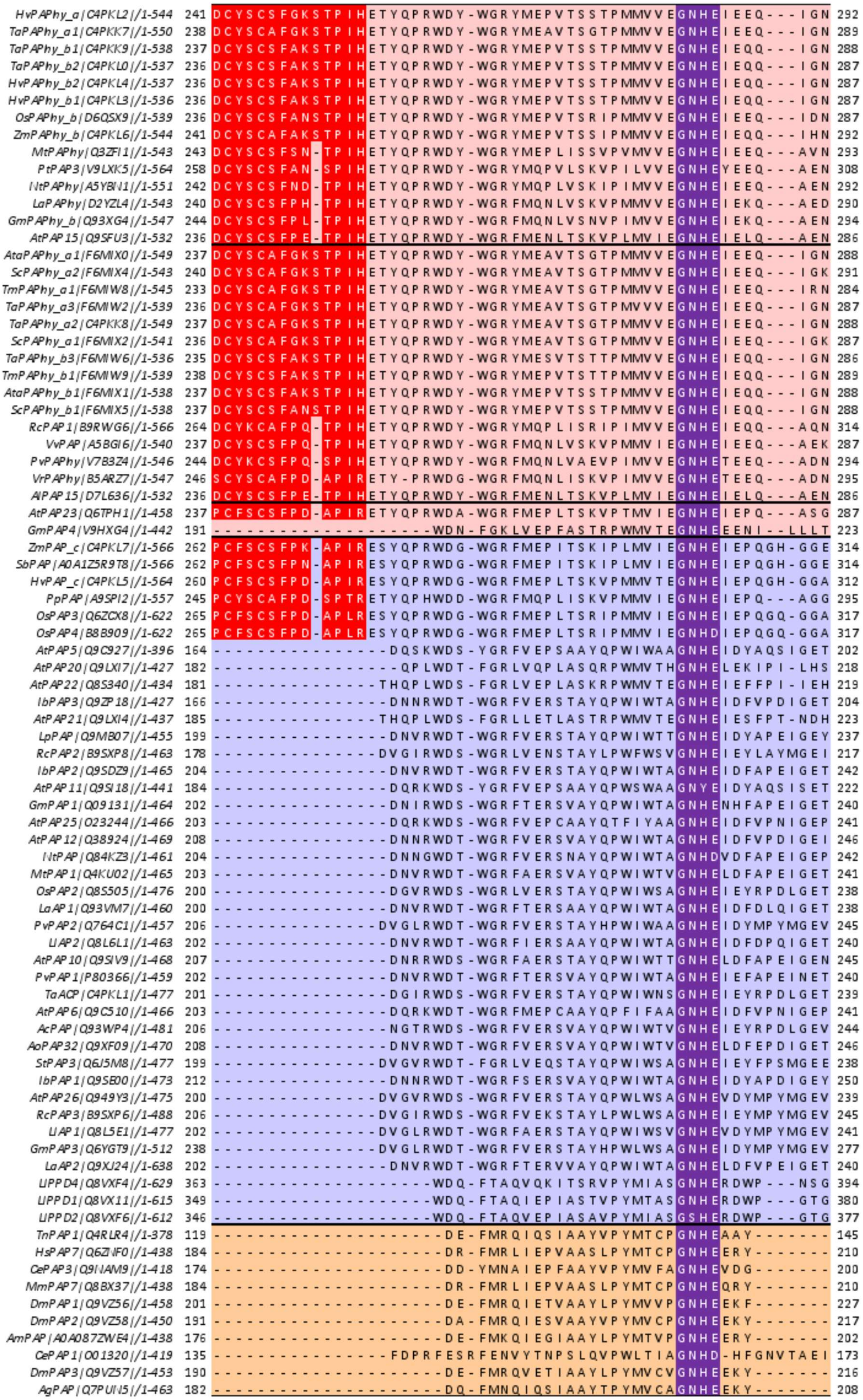
HvPAPhy_a|C4PKL2|/1-544 241 DCYSCSFGKSTPIHETYQPRWDY-WGRYMEPVTSSTPMMVVEGNHEIEEQ---IGN 292
TaPAPhy_a1|C4PKK7|/1-550 238 DCYSCAFGKSTPIHETYQPRWDY-WGRYMEAVTSGTPMMVVEGNHEIEEQ---IGN 289
TaPAPhy_b1|C4PKK9|/1-538 237 DCYSCSFAKSTPIHETYQPRWDY-WGRYMEPVTSSTPMMVVEGNHEIEQQ---IGN 288
TaPAPhy_b2|C4PKL0|/1-537 236 DCYSCSFAKSTPIHETYQPRWDY-WGRYMEPVTSSTPMMVVEGNHEIEQQ---IGN 287
HvPAPhy_b2|C4PKL4|/1-537 236 DCYSCSFAKSTPIHETYQPRWDY-WGRYMEPVTSSTPMMVVEGNHEIEQQ---IGN 287
HvPAPhy_b1|C4PKL3|/1-536 236 DCYSCSFAKSTPIHETYQPRWDY-WGRYMEPVTSSTPMMVVEGNHEIEQQ---IGN 287
OsPAPhy_b|D6QSX9|/1-539 236 DCYSCSFANSTPIHETYQPRWDY-WGRYMEPVTSRIPMMVVEGNHEIEEQ---IDN 287
ZmPAPhy_b|C4PKL6|/1-544 241 DCYSCAFAKSTPIHETYQPRWDY-WGRYMEPVTSSIPMMVVEGNHEIEQQ---IHN 292
MtPAPhy|Q3ZFI1|/1-543 243 DCYSCSFSN-TPIHETYQPRWDY-WGRYMEPLISSVPVMVVEGNHEIEEQ---AVN 293
PtPAP3|V9LXK5|/1-564 258 DCYSCSFAN-SPIHETYQPRWDY-WGRYMQPVLSKVPILVVEGNHEYEEQ---AEN 308
NtPAPhy|A5YBN1|/1-551 242 DCYSCSFND-TPIHETYQPRWDY-WGRYMQPLVSKIPIMVVEGNHEIEEQ---AEN 292
LaPAPhy|D2YZL4|/1-543 240 DCYSCSFPH-TPIHETYQPRWDY-WGRFMQNLVSKVPMMVVEGNHEIEKQ---AED 290
GmPAPhy_b|Q93XG4|/1-547 244 DCYSCSFPL-TPIHETYQPRWDY-WGRFMQNLVSNVPIMVVEGNHEIEKQ---AEN 294
AtPAP15|Q9SFU3|/1-532 236 DCYSCSFPE-TPIHETYQPRWDY-WGRFMENLTSKVPLMVIEGNHEIELQ---AEN 286
AtaPAPhy_a1|F6MIX0|/1-549 237 DCYSCAFGKSTPIHETYQPRWDY-WGRYMEAVTSGTPMMVVEGNHEIEEQ---IGN 288
ScPAPhy_a2|F6MIX4|/1-543 240 DCYSCAFGKSTPIHETYQPRWDY-WGRYMEAVTSGTPMMVVEGNHEIEEQ---IGK 291
TmPAPhy_a1|F6MIW8|/1-545 233 DCYSCAFGKSTPIHETYQPRWDY-WGRYMEAVTSGTPMMVVEGNHEIEEQ---IRN 284
TaPAPhy_a3|F6MIW2|/1-539 236 DCYSCAFGKSTPIHETYQPRWDY-WGRYMEAVTSGTPMVVVEGNHEIEEQ---IGN 287
TaPAPhy_a2|C4PKK8|/1-549 237 DCYSCAFGKSTPIHETYQPRWDY-WGRYMEAVTSGTPMMVVEGNHEIEEQ---IGN 288
ScPAPhy_a1|F6MIX2|/1-541 236 DCYSCAFGKSTPIHETYQPRWDY-WGRYMEAVTSGTPMMVVEGNHEIEEQ---IGK 287
TaPAPhy_b3|F6MIW6|/1-536 235 DCYSCSFAKSTPIHETYQPRWDY-WGRYMESVTSTTPMMVVEGNHEIEQQ---IGN 286
TmPAPhy_b1|F6MIW9|/1-539 238 DCYSCSFAKSTPIHETYQPRWDY-WGRYMEPVTSTTPMMVVEGNHEIEQQ---IGN 289
AtaPAPhy_b1|F6MIX1|/1-538 237 DCYSCSFAKSTPIHETYQPRWDY-WGRYMEPVTSSTPMMVVEGNHEIEQQ---IGN 288
ScPAPhy_b1|F6MIX5|/1-538 237 DCYSCSFANSTPIHETYQPRWDY-WGRYMEPVTSSTPMMVVEGNHEIEQQ---IGN 288
RcPAP1|B9RWG6|/1-566 264 DCYKCAFPQ-TPIHETYQPRWDY-WGRYMQPLISRIPIMVVEGNHEIEQQ---AQN 314
VvPAP|A5BGI6|/1-540 237 DCYSCSFPQ-TPIHETYQPRWDY-WGRFMQNLVSKVPMMVIEGNHEIEEQ---AEK 287
PvPAPhy|V7B3Z4|/1-546 244 DCYKCSFPQ-SPIHETYQPRWDY-WGRFMQNLVAEVPIMVVEGNHETEEQ---ADN 294
VrPAPhy|B5ARZ7|/1-547 246 SCYSCAFPD-APIRETY-PRWDG-WGRFMQNLISKVPIMVVEGNHETEEQ---ADN 295
AlPAP15|D7L636|/1-532 236 DCYSCSFPE-TPIHETYQPRWDY-WGRFMENLTSKVPLMVIEGNHEIELQ---AEN 286
AtPAP23|Q6TPH1|/1-458 237 PCFSCSFPD-APIRETYQPRWDA-WGRFMEPLTSKVPTMVIEGNHEIEPQ---ASG 287
GmPAP4|V9HXG4|/1-442 191 -------------------WDN-FGKLVEPFASTRPWMVTEGNHEEENI--LLLT 223
ZmPAP_c|C4PKL7|/1-566 262 PCFSCSFPK-APIRESYQPRWDG-WGRFMEPITSKIPLMVIEGNHEIEPQGH-GGE 314
SbPAP|A0A1Z5R9T8|/1-566 262 PCFSCSFPN-APIRESYQPRWDG-WGRFMEPITSKIPLMVIEGNHEIEPQGH-GGE 314
HvPAP_c|C4PKL5|/1-564 260 PCFSCSFPD-APIRESYQPRWDG-WGRFMEPLTSKVPMMVTEGNHEIEPQGH-GGA 312
PpPAP|A9SPI2|/1-557 245 PCYSCAFPD-SPTRETYQPHWDD-WGRFMQPLISKVPLMVIEGNHEIEPQ---AGG 295
OsPAP3|Q6ZCX8|/1-622 265 PCFSCSFPD-APLRESYQPRWDG-WGRFMEPLTSRIPMMVIEGNHEIEPQGQ-GGA 317
OsPAP4|B8B909|/1-622 265 PCFSCSFPD-APLRESYQPRWDG-WGRFMEPLTSRIPMMVIEGNHDIEPQGQ-GGA 317
AtPAP5|Q9C927|/1-396 164 ----------------DQSKWDS-YGRFVEPSAAYQPWIWAAGNHEIDYAQSIGET 202
AtPAP20|Q9LXI7|/1-427 182 -----------------QPLWDT-FGRLVQPLASQRPWMVTHGNHELEKIPI-LHS 218
AtPAP22|Q8S340|/1-434 181 ---------------THQPLWDS-FGRLVEPLASKRPWMVTEGNHEIEFFPI-IEH 219
IbPAP3|Q9ZP18|/1-427 166 ----------------DNNRWDT-WGRFVERSTAYQPWIWTAGNHEIDFVPDIGET 204
AtPAP21|Q9LXI4|/1-437 185 ---------------THQPLWDS-FGRLLETLASTRPWMVTEGNHEIESFPT-NDH 223
LpPAP|Q9MB07|/1-455 199 ----------------DNVRWDT-WGRFVERSTAYQPWIWTTGNHEIDYAPEIGEY 237
RcPAP2|B9SXP8|/1-463 178 ---------------DVGIRWDS-WGRLVENSTAYLPWFWSVGNHEIEYLAYMGEI 217
IbPAP2|Q9SDZ9|/1-465 204 ----------------DNVRWDT-WGRFVERSTAYQPWIWTAGNHEIDFAPEIGET 242
AtPAP11|Q9SI18|/1-441 184 ----------------DQRKWDS-YGRFVEPSAAYQPWSWAAGNYEIDYAQSISET 222
GmPAP1|Q09131|/1-464 202 ----------------DNIRWDS-WGRFTERSVAYQPWIWTAGNHENHFAPEIGET 240
AtPAP25|O23244|/1-466 203 ----------------DQRKWDS-WGRFVEPCAAYQTFIYAAGNHEIDFVPNIGEP 241
AtPAP12|Q38924|/1-469 208 ----------------DNNRWDT-WGRFVERSVAYQPWIWTAGNHEIDFVPDIGEI 246
NtPAP|Q84KZ3|/1-461 204 ----------------DNNGWDT-WGRFVERSNAYQPWIWTAGNHDVDFAPEIGEP 242
MtPAP1|Q4KU02|/1-465 203 ----------------DNVRWDT-WGRFAERSVAYQPWIWTVGNHELDFAPEIGET 241
OsPAP2|Q8S505|/1-476 200 ----------------DGVRWDS-WGRLVERSTAYQPWIWSAGNHEIEYRPDLGET 238
LaAP1|Q93VM7|/1-460 200 ----------------DNVRWDT-WGRFTERSAAYQPWIWTAGNHEIDFDLQIGET 238
PvPAP2|Q764C1|/1-457 206 ---------------DVGLRWDT-WGRFVERSTAYHPWIWAAGNHEIDYMPYMGEV 245
LlAP2|Q8L6L1|/1-463 202 ----------------DNVRWDT-WGRFIERSAAYQPWIWTAGNHEIDFDPQIGET 240
AtPAP10|Q9SIV9|/1-468 207 ----------------DNRRWDS-WGRFAERSTAYQPWIWTTGNHELDFAPEIGEN 245
PvPAP1|P80366|/1-459 202 ----------------DNVRWDT-WGRFTERSVAYQPWIWTAGNHEIEFAPEINET 240
TaACP|C4PKL1|/1-477 201 ----------------DGIRWDS-WGRFVERSTAYQPWIWNSGNHEIEYRPDLGET 239
AtPAP6|Q9C510|/1-466 203 ----------------DQRKWDT-WGRFMEPCAAYQPFIFAAGNHEIDFVPNIGEP 241
AcPAP|Q93WP4|/1-481 206 ----------------NGTRWDS-WGRFVERSVAYQPWIWTVGNHEIEYRPDLGEV 244
AoPAP32|Q9XF09|/1-470 208 ----------------DNVRWDT-WGRFVERSVAYQPWIWTVGNHELDFEPDIGET 246
StPAP3|Q6J5M8|/1-477 199 ---------------DVGVRWDT-FGRLVEQSTAYQPWIWSAGNHEIEYFPSMGEE 238
IbPAP1|Q9SE00|/1-473 212 ----------------DNNRWDT-WGRFSERSVAYQPWIWTAGNHEIDYAPDIGEY 250
AtPAP26|Q949Y3|/1-475 200 ---------------DVGVRWDS-WGRFVERSTAYQPWLWSAGNHEVDYMPYMGEV 239
RcPAP3|B9SXP6|/1-488 206 ---------------DVGIRWDS-WGRFVEKSTAYLPWLWSAGNHEIEYMPYMGEV 245
LlAP1|Q8L5E1|/1-477 202 ---------------DVGLRWDT-WGRFAERSTAYQPWIWSVGNHEVDYMPYMGEV 241
GmPAP3|Q6YGT9|/1-512 238 ---------------DVGLRWDT-WGRFVERSTAYHPWLWSAGNHEIDYMPYMGEV 277
LaAP2|Q9XJ24|/1-638 202 ----------------DNVRWDT-WGRFTERVVAYQPWIWTAGNHELDFVPEIGET 240
LlPPD4|Q8VXF4|/1-629 363 --------------------WDQ-FTAQVQKITSRVPYMIASGNHERDWP---NSG 394
LlPPD1|Q8VX11|/1-615 349 --------------------WDQ-FTAQIEPIASTVPYMTASGNHERDWP---GTG 380
LlPPD2|Q8VXF6|/1-612 346 --------------------WDQ-FTAQVEPIASAVPYMIASGSHERDWP---GTG 377
TnPAP1|Q4RLR4|/1-378 119 ---------------------DE-FMRQIQSIAAYVPYMTCPGNHEAAY------- 145
HsPAP7|Q6ZNF0|/1-438 184 ---------------------DR-FMRLIEPVAASLPYMTCPGNHEERY------- 210
CePAP3|Q9NAM9|/1-418 174 ---------------------DD-YMNAIEPFAAYVPYMVFAGNHEVDG------- 200
MmPAP7|Q8BX37|/1-438 184 ---------------------DR-FMRLIEPVAASLPYMTCPGNHEQRY------- 210
DmPAP1|Q9VZ56|/1-458 201 ---------------------DE-FMRQIETVAAYLPYMVVPGNHEEKF------- 227
DmPAP2|Q9VZ58|/1-450 191 ---------------------DA-FMRQIESVAAYVPYMVCPGNHEEKY------- 217
AmPAP|A0A087ZWE4|/1-438 176 ---------------------DE-FMKQIEGIAAYLPYMTVPGNHEERY------- 202
CePAP1|O01320|/1-419 135 ----------------FDPRFESRFENVYTNPSLQVPWLTIAGNHD-HFGNVTAEI 173
DmPAP3|Q9VZ57|/1-453 190 ---------------------DE-FMRQVETIAAYLPYMVCVGNHEEKY------- 216
AgPAP|Q7PUN5|/1-463 182 ---------------------DQ-FMNQIQSIAAYTPYMVCAGNHEEKY------- 208

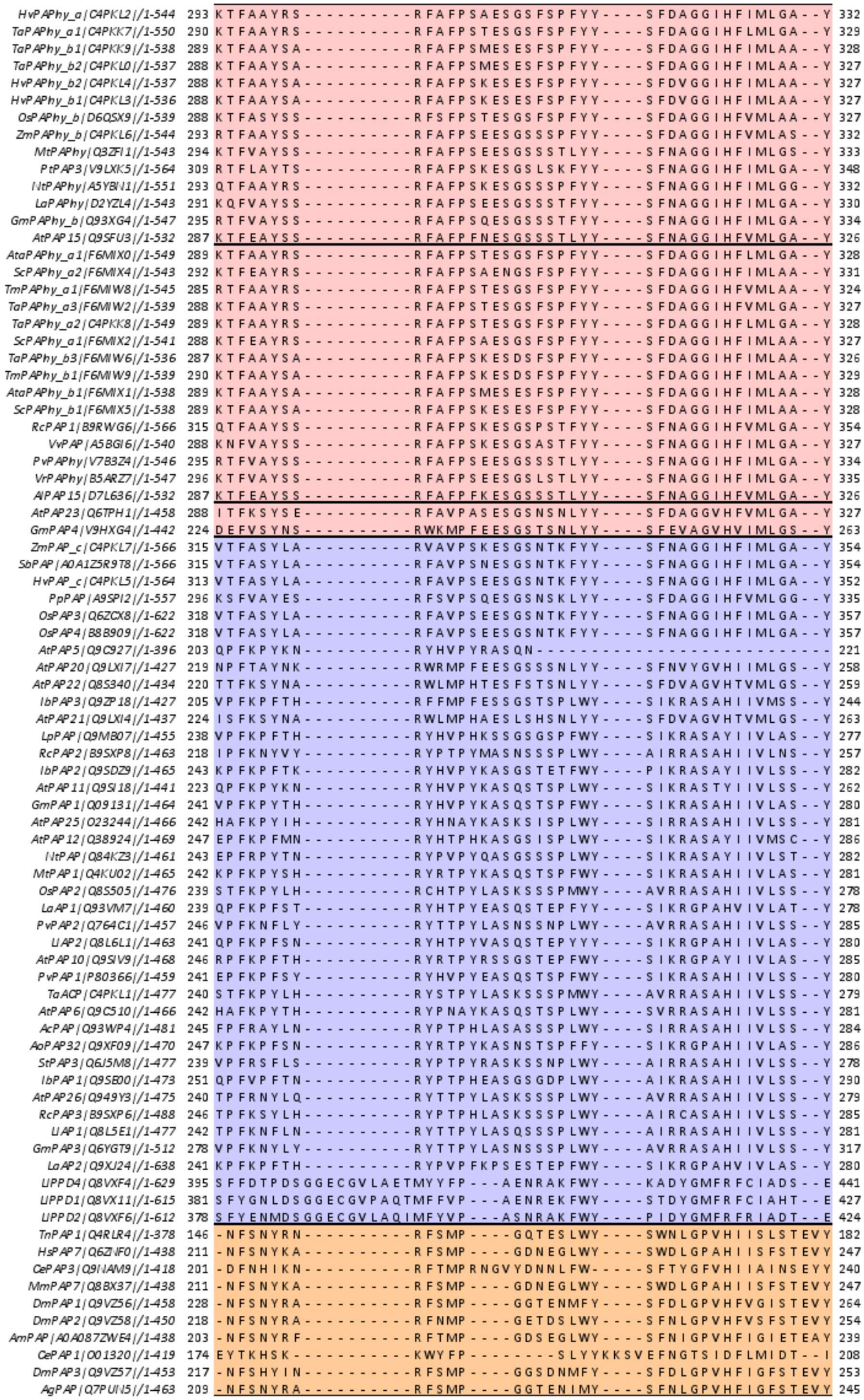
HvPAPhy_a|C4PKL2|/1-544 293 KTFAAYRS----------RFAFPSAESGSFSPFYY----SFDAGGIHFIMLGA--Y 332
TaPAPhy_a1|C4PKK7|/1-550 290 KTFAAYRS----------RFAFPSTESGSFSPFYY----SFDAGGIHFLMLGA--Y 329
TaPAPhy_b1|C4PKK9|/1-538 289 KTFAAYSA----------RFAFPSMESESFSPFYY----SFDAGGIHFIMLAA--Y 328
TaPAPhy_b2|C4PKL0|/1-537 288 KTFAAYSA----------RFAFPSMESESFSPFYY----SFDAGGIHFIMLAA--Y 327
HvPAPhy_b2|C4PKL4|/1-537 288 KTFAAYSA----------RFAFPSKESESFSPFYY----SFDVGGIHFIMLAA--Y 327
HvPAPhy_b1|C4PKL3|/1-536 288 KTFAAYSA----------RFAFPSKESESFSPFYY----SFDVGGIHFIMLAA--Y 327
OsPAPhy_b|D6QSX9|/1-539 288 KTFASYSS----------RFSFPSTESGSFSPFYY----SFDAGGIHFVMLAA--Y 327
ZmPAPhy_b|C4PKL6|/1-544 293 RTFAAYSS----------RFAFPSEESGSSSPFYY----SFDAGGIHFVMLAS--Y 332
MtPAPhy|Q3ZFI1|/1-543 294 KTFVAYSS----------RFAFPSEESGSSSTLYY----SFNAGGIHFIMLGS--Y 333
PtPAP3|V9LXK5|/1-564 309 RTFLAYTS----------RFAFPSKESGSLSKFYY----SFNAGGIHFIMLGA--Y 348
NtPAPhy|A5YBN1|/1-551 293 QTFAAYRS----------RFAFPSKESGSSSPFYY----SFNAGGIHFIMLGG--Y 332
LaPAPhy|D2YZL4|/1-543 291 KQFVAYSS----------RFAFPSEESGSSSTFYY----SFNAGGIHFIMLGA--Y 330
GmPAPhy_b|Q93XG4|/1-547 295 RTFVAYSS----------RFAFPSQESGSSSTFYY----SFNAGGIHFIMLGA--Y 334
AtPAP15|Q9SFU3|/1-532 287 KTFEAYSS----------RFAFPFNESGSSSTLYY----SFNAGGIHFVMLGA--Y 326
AtaPAPhy_a1|F6MIX0|/1-549 289 KTFAAYRS----------RFAFPSTESGSFSPFYY----SFDAGGIHFLMLGA--Y 328
ScPAPhy_a2|F6MIX4|/1-543 292 KTFEAYRS----------RFAFPSAENGSFSPFYY----SFDAGGIHFIMLAA--Y 331
TmPAPhy_a1|F6MIW8|/1-545 285 RTFAAYRS----------RFAFPSTESGSFSPFYY----SFDAGGIHFVMLAA--Y 324
TaPAPhy_a3|F6MIW2|/1-539 288 KTFAAYRS----------RFAFPSTESGSFSPFYY----SFDAGGIHFVMLGA--Y 327
TaPAPhy_a2|C4PKK8|/1-549 289 KTFAAYRS----------RFAFPSTESGSFSPFYY----SFDAGGIHFLMLGA--Y 328
ScPAPhy_a1|F6MIX2|/1-541 288 KTFEAYRS----------RFAFPSAESGSFSPFYY----SFDAGGIHFIMLAA--Y 327
TaPAPhy_b3|F6MIW6|/1-536 287 KTFAAYSA----------RFAFPSKESDSFSPFYY----SFDAGGIHFIMLAA--Y 326
TmPAPhy_b1|F6MIW9|/1-539 290 KTFAAYSA----------RFAFPSKESDSFSPFYY----SFDAGGIHFIMLAA--Y 329
AtaPAPhy_b1|F6MIX1|/1-538 289 KTFAAYSA----------RFAFPSMESESFSPFYY----SFDAGGIHFIMLAA--Y 328
ScPAPhy_b1|F6MIX5|/1-538 289 KTFAAYSA----------RFAFPSKESESFSPFYY----SFDAGGIHFIMLAA--Y 328
RcPAP1|B9RWG6|/1-566 315 QTFAAYSS----------RFAFPSKESGSPSTFYY----SFNAGGIHFVMLGA--Y 354
VvPAP|A5BGI6|/1-540 288 KNFVAYSS----------RFAFPSKESGSASTFYY----SFNAGGIHFIMLGA--Y 327
PvPAPhy|V7B3Z4|/1-546 295 RTFVAYSS----------RFAFPSEESGSSSTLYY----SFNAGGIHFIMLGA--Y 334
VrPAPhy|B5ARZ7|/1-547 296 KTFVAYSS----------RFAFPSEESGSLSTLYY----SFNAGGIHFIMLGA--Y 335
AlPAP15|D7L636|/1-532 287 KTFEAYSS----------RFAFPFKESGSSSTLYY----SFNAGGIHFVMLGA--Y 326
AtPAP23|Q6TPH1|/1-458 288 ITFKSYSE----------RFAVPASESGSNSNLYY----SFDAGGVHFVMLGA--Y 327
GmPAP4|V9HXG4|/1-442 224 DEFVSYNS----------RWKMPFEESGSTSNLYY----SFEVAGVHVIMLGS--Y 263
ZmPAP_c|C4PKL7|/1-566 315 VTFASYLA----------RVAVPSKESGSNTKFYY----SFNAGGIHFIMLGA--Y 354
SbPAP|A0A1Z5R9T8|/1-566 315 VTFASYLA----------RFAVPSNESGSNTKFYY----SFNAGGIHFIMLGA--Y 354
HvPAP_c|C4PKL5|/1-564 313 VTFASYLA----------RFAVPSEESGSNTKFYY----SFNAGGIHFIMLGA--Y 352
PpPAP|A9SPI2|/1-557 296 KSFVAYES----------RFSVPSQESGSNSKLYY----SFDAGGIHFVMLGG--Y 335
OsPAP3|Q6ZCX8|/1-622 318 VTFASYLA----------RFAVPSEESGSNTKFYY----SFNAGGIHFIMLGA--Y 357
OsPAP4|B8B909|/1-622 318 VTFASYLA----------RFAVPSEESGSNTKFYY----SFNAGGIHFIMLGA--Y 357
AtPAP5|Q9C927|/1-396 203 QPFKPYKN----------RYHVPYRASQN--------------------------- 221
AtPAP20|Q9LXI7|/1-427 219 NPFTAYNK----------RWRMPFEESGSSSNLYY----SFNVYGVHIIMLGS--Y 258
AtPAP22|Q8S340|/1-434 220 TTFKSYNA----------RWLMPHTESFSTSNLYY----SFDVAGVHTVMLGS--Y 259
IbPAP3|Q9ZP18|/1-427 205 VPFKPFTH----------RFFMPFESSGSTSPLWY----SIKRASAHIIVMSS--Y 244
AtPAP21|Q9LXI4|/1-437 224 ISFKSYNA----------RWLMPHAESLSHSNLYY----SFDVAGVHTVMLGS--Y 263
LpPAP|Q9MB07|/1-455 238 VPFKPFTH----------RYHVPHKSSGSGSPFWY----SIKRASAYIIVLAS--Y 277
RcPAP2|B9SXP8|/1-463 218 IPFKNYVY----------RYPTPYMASNSSSPLWY----AIRRASAHIIVLNS--Y 257
IbPAP2|Q9SDZ9|/1-465 243 KPFKPFTK----------RYHVPYKASGSTETFWY----PIKRASAYIIVLSS--Y 282
AtPAP11|Q9SI18|/1-441 223 QPFKPYKN----------RYHVPYKASQSTSPLWY----SIKRASTYIIVLSS--Y 262
GmPAP1|Q09131|/1-464 241 VPFKPYTH----------RYHVPYKASQSTSPFWY----SIKRASAHIIVLAS--Y 280
AtPAP25|O23244|/1-466 242 HAFKPYIH----------RYHNAYKASKSISPLWY----SIRRASAHIIVLSS--Y 281
AtPAP12|Q38924|/1-469 247 EPFKPFMN----------RYHTPHKASGSISPLWY----SIKRASAYIIVMSC--Y 286
NtPAP|Q84KZ3|/1-461 243 EPFRPYTN----------RYPVPYQASGSSSPLWY----SIKRASAYIIVLST--Y 282
MtPAP1|Q4KU02|/1-465 242 KPFKPYSH----------RYRTPYKASQSTSPFWY----SIKRASAHIIVLAS--Y 281
OsPAP2|Q8S505|/1-476 239 STFKPYLH----------RCHTPYLASKSSSPMWY----AVRRASAHIIVLSS--Y 278
LaAP1|Q93VM7|/1-460 239 QPFKPFST----------RYHTPYEASQSTEPFWY----SIKRGPAHVIVLAT--Y 278
PvPAP2|Q764C1|/1-457 246 VPFKNFLY----------RYTTPYLASNSSNPLWY----AVRRASAHIIVLSS--Y 285
LlAP2|Q8L6L1|/1-463 241 QPFKPFSN----------RYHTPYVASQSTEPYYY----SIKRGPAHIIVLAS--Y 280
AtPAP10|Q9SIV9|/1-468 246 RPFKPFTH----------RYRTPYRSSGSTEPFWY----SIKRGPAYIIVLAS--Y 285
PvPAP1|P80366|/1-459 241 EPFKPFSY----------RYHVPYEASQSTSPFWY----SIKRASAHIIVLSS--Y 280
TaACP|C4PKL1|/1-477 240 STFKPYLH----------RYSTPYLASKSSSPMWY----AVRRASAHIIVLSS--Y 279
AtPAP6|Q9C510|/1-466 242 HAFKPYTH----------RYPNAYKASQSTSPLWY----SVRRASAHIIVLSS--Y 281
AcPAP|Q93WP4|/1-481 245 FPFRAYLN----------RYPTPHLASASSSPLWY----SIRRASAHIIVLSS--Y 284
AoPAP32|Q9XF09|/1-470 247 KPFKPFSN----------RYRTPYKASNSTSPFFY----SIKRGPAHIIVLAS--Y 286
StPAP3|Q6J5M8|/1-477 239 VPFRSFLS----------RYPTPYRASKSSNPLWY----AIRRASAHIIVLSS--Y 278
IbPAP1|Q9SE00|/1-473 251 QPFVPFTN----------RYPTPHEASGSGDPLWY----AIKRASAHIIVLSS--Y 290
AtPAP26|Q949Y3|/1-475 240 TPFRNYLQ----------RYTTPYLASKSSSPLWY----AVRRASAHIIVLSS--Y 279
RcPAP3|B9SXP6|/1-488 246 TPFKSYLH----------RYPTPHLASKSSSPLWY----AIRCASAHIIVLSS--Y 285
LlAP1|Q8L5E1|/1-477 242 TPFKNFLN----------RYTTPYLASQSSSPLWY----AIRRASAHIIVLSS--Y 281
GmPAP3|Q6YGT9|/1-512 278 VPFKNYLY----------RYTTPYLASNSSSPLWY----AVRRASAHIIVLSS--Y 317
LaAP2|Q9XJ24|/1-638 241 KPFKPFTH----------RYPVPFKPSESTEPFWY----SIKRGPAHVIVLAS--Y 280
LlPPD4|Q8VXF4|/1-629 395 SFFDTPDSGGECGVLAETMYYFP---AENRAKFWY----KADYGMFRFCIADS--E 441
LlPPD1|Q8VX11|/1-615 381 SFYGNLDSGGECGVPAQTMFFVP---AENRKKFWY----STDYGMFRFCIAHT--E 427
LlPPD2|Q8VXF6|/1-612 378 SFYENMDSGGECGVLAQIMFYVP---ASNRAKFWY----PIDYGMFRFRIADT--E 424
TnPAP1|Q4RLR4|/1-378 146 -NFSNYRN----------RFSMP----GQTESLWY----SWNLGPVHIISLSTEVY 182
HsPAP7|Q6ZNF0|/1-438 211 -NFSNYKA----------RFSMP----GDNEGLWY----SWDLGPAHIISFSTEVY 247
CePAP3|Q9NAM9|/1-418 201 -DFNHIKN----------RFTMPRNGVYDNNLFW-----SFTYGFVHIIAINSEYY 240
MmPAP7|Q8BX37|/1-438 211 -NFSNYKA----------RFSMP----GDNEGLWY----SWDLGPAHIISFSTEVY 247
DmPAP1|Q9VZ56|/1-458 228 -NFSNYRA----------RFSMP----GGTENMFY----SFDLGPVHFVGISTEVY 264
DmPAP2|Q9VZ58|/1-450 218 -NFSNYRA----------RFNMP----GETDSLWY----SFNLGPVHFVSFSTEVY 254
AmPAP|A0A087ZWE4|/1-438 203 -NFSNYRF----------RFTMP----GDSEGLWY----SFNIGPVHFIGIETEAY 239
CePAP1|O01320|/1-419 174 EYTKHSK-----------KWYFP--------SLYYKKSVEFNGTSIDFLMIDT--I 208
DmPAP3|Q9VZ57|/1-453 217 -NFSHYIN----------RFSMP----GGSDNMFY----SFDLGPVHFIGFSTEVY 253
AgPAP|Q7PUN5|/1-463 209 -NFSNYRA----------RFSMP----GGTENIMY----SFNLGPVHFIGFSTEVY 245

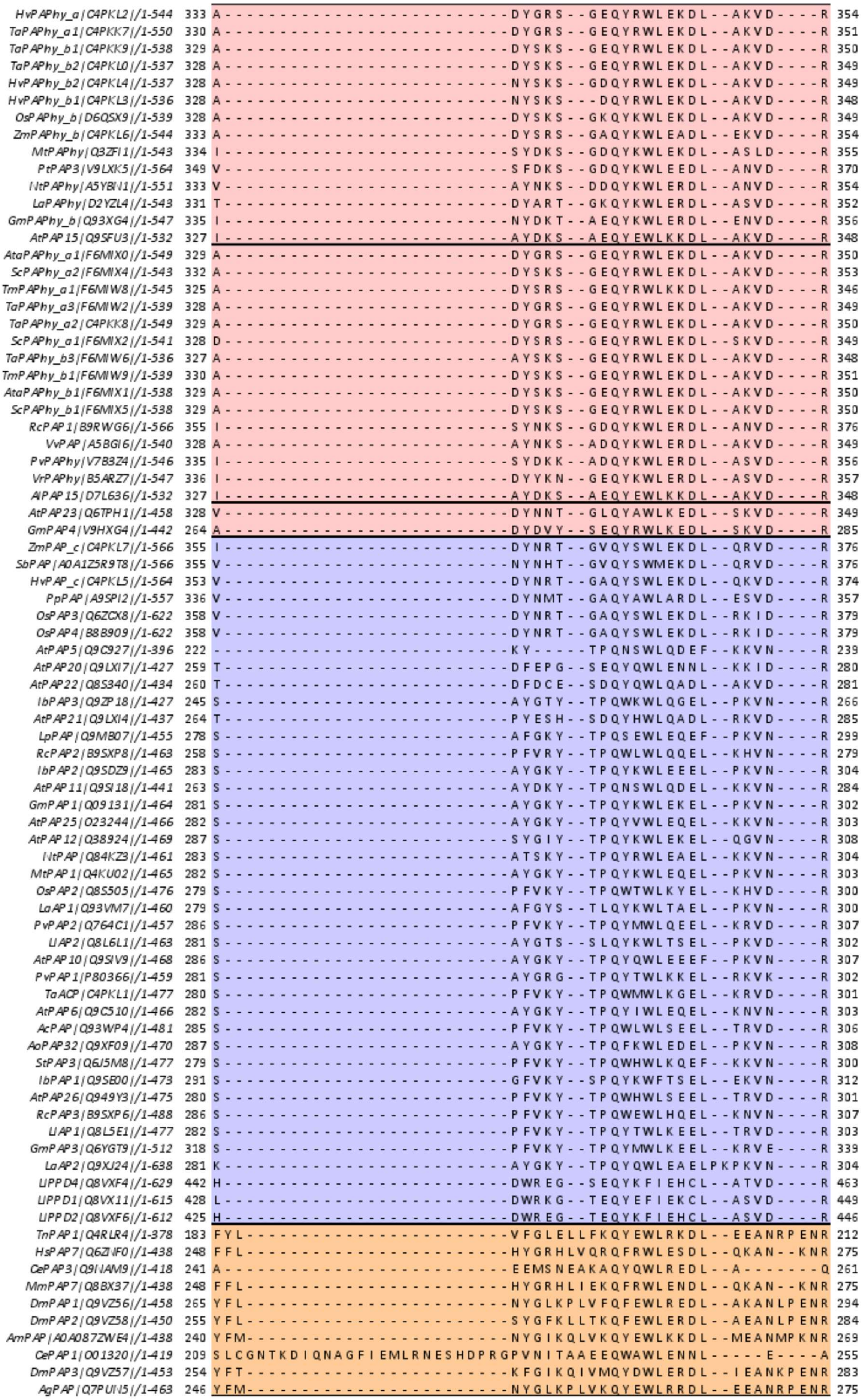

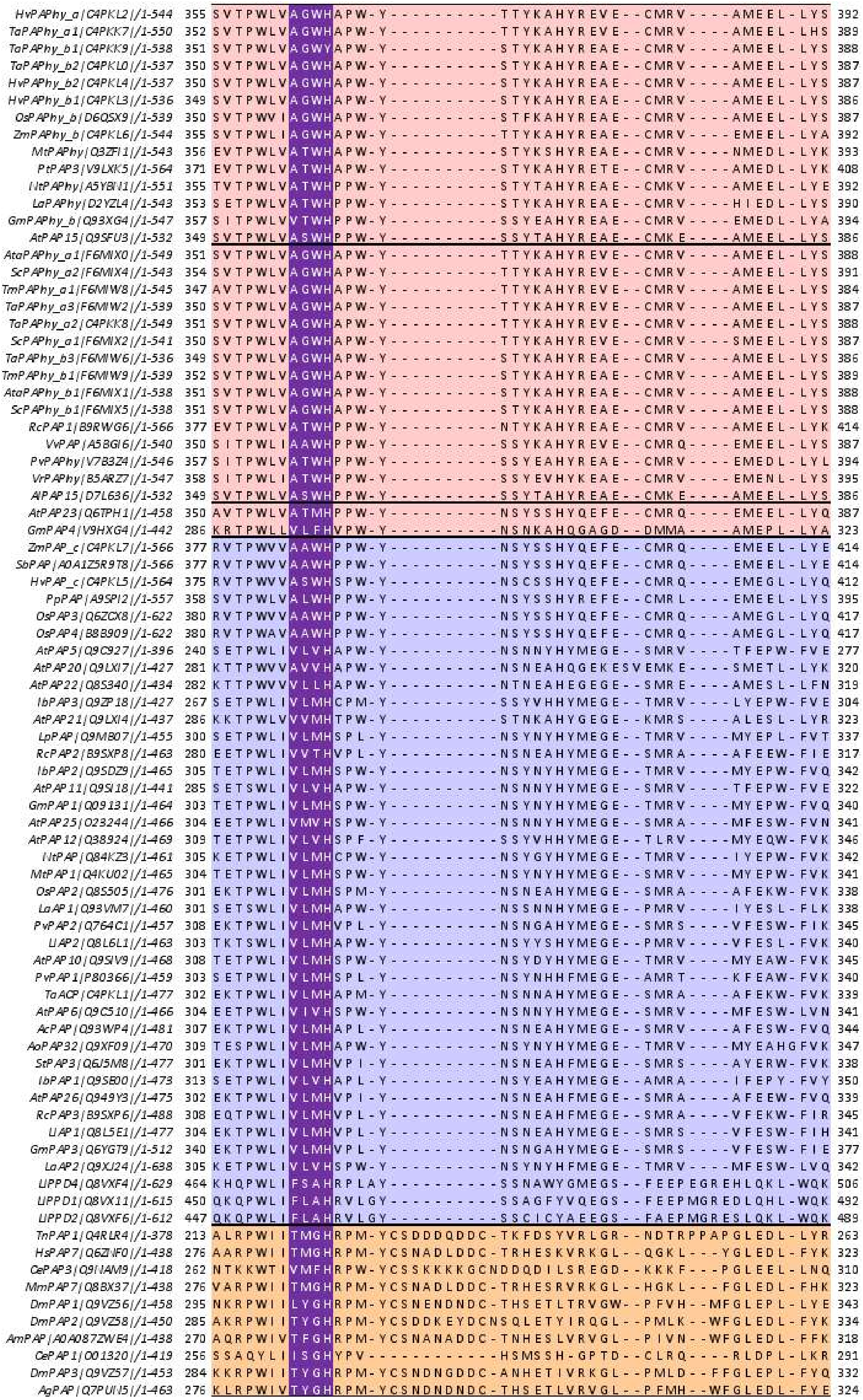
HvPAPhy_a|C4PKL2|/1-544 355 SVTPWLVAGWHAPW-Y----------TTYKAHYREVE--CMRV----AMEEL-LYS 392
TaPAPhy_a1|C4PKK7|/1-550 352 SVTPWLVAGWHAPW-Y----------TTYKAHYREVE--CMRV----AMEEL-LHS 389
TaPAPhy_b1|C4PKK9|/1-538 351 SVTPWLVAGWYAPW-Y----------STYKAHYREAE--CMRV----AMEEL-LYS 388
TaPAPhy_b2|C4PKL0|/1-537 350 SVTPWLVAGWHAPW-Y----------STYKAHYREAE--CMRV----AMEEL-LYS 387
HvPAPhy_b2|C4PKL4|/1-537 350 SVTPWLVAGWHAPW-Y----------STYKAHYREAE--CMRV----AMEEL-LYS 387
HvPAPhy_b1|C4PKL3|/1-536 349 SVTPWLVAGWHAPW-Y----------STYKAHYREAE--CMRV----AMEEL-LYS 386
OsPAPhy_b|D6QSX9|/1-539 350 SVTPWVIAGWHAPW-Y----------STFKAHYREAE--CMRV----AMEEL-LYS 387
ZmPAPhy_b|C4PKL6|/1-544 355 SVTPWLIAGWHAPW-Y----------TTYKAHYREAE--CMRV----EMEEL-LYA 392
MtPAPhy|Q3ZFI1|/1-543 356 EVTPWLVATWHAPW-Y----------STYKSHYREAE--CMRV----NMEDL-LYK 393
PtPAP3|V9LXK5|/1-564 371 EVTPWLVATWHAPW-Y----------STYKAHYRETE--CMRV----AMEDL-LYK 408
NtPAPhy|A5YBN1|/1-551 355 TVTPWLVATWHPPW-Y----------STYTAHYREAE--CMKV----AMEEL-LYE 392
LaPAPhy|D2YZL4|/1-543 353 SETPWLVATWHPPW-Y----------STYKAHYREAE--CMRV----HIEDL-LYS 390
GmPAPhy_b|Q93XG4|/1-547 357 SITPWLVVTWHPPW-Y----------SSYEAHYREAE--CMRV----EMEDL-LYA 394
AtPAP15|Q9SFU3|/1-532 349 SVTPWLVASWHPPW-Y----------SSYTAHYREAE--CMKE----AMEEL-LYS 386
AtaPAPhy_a1|F6MIX0|/1-549 351 SVTPWLVAGWHAPW-Y----------TTYKAHYREVE--CMRV----AMEEL-LYS 388
ScPAPhy_a2|F6MIX4|/1-543 354 SVTPWLVAGWHAPW-Y----------TTYKAHYREVE--CMRV----AMEEL-LYS 391
TmPAPhy_a1|F6MIW8|/1-545 347 AVTPWLVAGWHAPW-Y----------TTYKAHYREVE--CMRV----AMEEL-LYS 384
TaPAPhy_a3|F6MIW2|/1-539 350 SVTPWLVAGWHAPW-Y----------TTYKAHYREVE--CMRV----AMEEL-LYS 387
TaPAPhy_a2|C4PKK8|/1-549 351 SVTPWLVAGWHAPW-Y----------TTYKAHYREVE--CMRV----AMEEL-LYS 388
ScPAPhy_a1|F6MIX2|/1-541 350 SVTPWLVAGWHAPW-Y----------TTYKAHYREVE--CMRV----SMEEL-LYS 387
TaPAPhy_b3|F6MIW6|/1-536 349 SVTPWLVAGWHAPW-Y----------STYKAHYREAE--CMRV----AMEEL-LYS 386
TmPAPhy_b1|F6MIW9|/1-539 352 SVTPWLVAGWHAPW-Y----------STYKAHYREAE--CMRV----AMEEL-LYS 389
AtaPAPhy_b1|F6MIX1|/1-538 351 SVTPWLVAGWHAPW-Y----------STYKAHYREAE--CMRV----AMEEL-LYS 388
ScPAPhy_b1|F6MIX5|/1-538 351 SVTPWLVAGWHAPW-Y----------STYKAHYREAE--CMRV----AMEEL-LYS 388
RcPAP1|B9RWG6|/1-566 377 EVTPWLVATWHPPW-Y----------NTYKAHYREAE--CMRV----AMEEL-LYK 414
VvPAP|A5BGI6|/1-540 350 SITPWLIAAWHPPW-Y----------SSYKAHYREVE--CMRQ----EMEEL-LYS 387
PvPAPhy|V7B3Z4|/1-546 357 SITPWLVATWHPPW-Y----------SSYEAHYREAE--CMRV----EMEDL-LYL 394
VrPAPhy|B5ARZ7|/1-547 358 SITPWLIATWHPPW-Y----------SSYEVHYKEAE--CMRV----EMENL-LYS 395
AlPAP15|D7L636|/1-532 349 SVTPWLVASWHPPW-Y----------SSYTAHYREAE--CMKE----AMEEL-LYS 386
AtPAP23|Q6TPH1|/1-458 350 AVTPWLVATMHPPW-Y----------NSYSSHYQEFE--CMRQ----EMEEL-LYQ 387
GmPAP4|V9HXG4|/1-442 286 KRTPWLLVLFHVPW-Y----------NSNKAHQGAGD--DMMA----AMEPL-LYA 323
ZmPAP_c|C4PKL7|/1-566 377 RVTPWVVAAWHPPW-Y----------NSYSSHYQEFE--CMRQ----EMEEL-LYE 414
SbPAP|A0A1Z5R9T8|/1-566 377 RVTPWVVAAWHPPW-Y----------NSYSSHYQEFE--CMRQ----EMEEL-LYE 414
HvPAP_c|C4PKL5|/1-564 375 RVTPWVVASWHSPW-Y----------NSCSSHYQEFE--CMRQ----EMEGL-LYQ 412
PpPAP|A9SPI2|/1-557 358 SVTPWLVALWHPPW-Y----------NSYSSHYREFE--CMRL----EMEEL-LYS 395
OsPAP3|Q6ZCX8|/1-622 380 RVTPWVVAAWHPPW-Y----------NSYSSHYQEFE--CMRQ----AMEGL-LYQ 417
OsPAP4|B8B909|/1-622 380 RVTPWAVAAWHPPW-Y----------NSYSSHYQEFE--CMRQ----AMEGL-LYQ 417
AtPAP5|Q9C927|/1-396 240 SETPWLIVLVHAPW-Y----------NSNNYHYMEGE--SMRV----TFEPW-FVE 277
AtPAP20|Q9LXI7|/1-427 281 KTTPWVVAVVHAPW-Y----------NSNEAHQGEKESVEMKE----SMETL-LYK 320
AtPAP22|Q8S340|/1-434 282 KTTPWVVVLLHAPW-Y----------NTNEAHEGEGE--SMRE----AMESL-LFN 319
IbPAP3|Q9ZP18|/1-427 267 SETPWLIVLMHCPM-Y----------SSYVHHYMEGE--TMRV----LYEPW-FVE 304
AtPAP21|Q9LXI4|/1-437 286 KKTPWLVVVMHTPW-Y----------STNKAHYGEGE--KMRS----ALESL-LYR 323
LpPAP|Q9MB07|/1-455 300 SETPWLIVLMHSPL-Y----------NSYNYHYMEGE--TMRV----MYEPL-FVT 337
RcPAP2|B9SXP8|/1-463 280 EETPWLIVVTHVPL-Y----------NSNEAHYMEGE--SMRA----AFEEW-FIE 317
IbPAP2|Q9SDZ9|/1-465 305 TETPWLIVLMHSPW-Y----------NSYNYHYMEGE--TMRV----MYEPW-FVQ 342
AtPAP11|Q9SI18|/1-441 285 SETSWLIVLVHAPW-Y----------NSNNYHYMEGE--SMRV----TFEPW-FVE 322
GmPAP1|Q09131|/1-464 303 TETPWLIVLMHSPW-Y----------NSYNYHYMEGE--TMRV----MYEPW-FVQ 340
AtPAP25|O23244|/1-466 304 EETPWLIVMVHSPW-Y----------NSNNYHYMEGE--SMRA----MFESW-FVN 341
AtPAP12|Q38924|/1-469 309 TETPWLIVLVHSPF-Y----------SSYVHHYMEGE--TLRV----MYEQW-FVK 346
NtPAP|Q84KZ3|/1-461 305 KETPWLIVLMHCPW-Y----------NSYGYHYMEGE--TMRV----IYEPW-FVK 342
MtPAP1|Q4KU02|/1-465 304 TETPWLIVLMHSPW-Y----------NSYNYHYMEGE--SMRV----MYEPW-FVK 341
OsPAP2|Q8S505|/1-476 301 EKTPWLIVLMHSPM-Y----------NSNEAHYMEGE--SMRA----AFEKW-FVK 338
LaAP1|Q93VM7|/1-460 301 SETSWLIVLMHAPW-Y----------NSSNNHYMEGE--PMRV----IYESL-FLK 338
PvPAP2|Q764C1|/1-457 308 EKTPWLIVLMHVPL-Y----------NSNGAHYMEGE--SMRS----VFESW-FIK 345
LlAP2|Q8L6L1|/1-463 303 TKTSWLIVLMHAPW-Y----------NSYYSHYMEGE--PMRV----VFESL-FVK 340
AtPAP10|Q9SIV9|/1-468 308 TETPWLIVLMHSPW-Y----------NSYDYHYMEGE--TMRV----MYEAW-FVK 345
PvPAP1|P80366|/1-459 303 SETPWLIVLMHSPL-Y----------NSYNHHFMEGE--AMRT----KFEAW-FVK 340
TaACP|C4PKL1|/1-477 302 EKTPWLIVLMHAPM-Y----------NSNNAHYMEGE--SMRA----AFEKW-FVK 339
AtPAP6|Q9C510|/1-466 304 EETPWLIVIVHSPW-Y----------NSNNYHYMEGE--SMRV----MFESW-LVN 341
AcPAP|Q93WP4|/1-481 307 EKTPWLIVLMHAPL-Y----------NSNEAHYMEGE--SMRV----AFESW-FVQ 344
AoPAP32|Q9XF09|/1-470 309 TESPWLIVLMHAPW-Y----------NSYNYHYMEGE--TMRV----MYEAHGFVK 347
StPAP3|Q6J5M8|/1-477 301 EKTPWLIVLMHVPI-Y----------NSNEAHFMEGE--SMRS----AYERW-FVK 338
IbPAP1|Q9SE00|/1-473 313 SETPWLIVLVHAPL-Y----------NSYEAHYMEGE--AMRA----IFEPY-FVY 350
AtPAP26|Q949Y3|/1-475 302 EKTPWLIVLMHVPI-Y----------NSNEAHFMEGE--SMRA----AFEEW-FVQ 339
RcPAP3|B9SXP6|/1-488 308 EQTPWLIVLMHVPL-Y----------NSNEAHFMEGE--SMRA----VFEKW-FIR 345
LlAP1|Q8L5E1|/1-477 304 EKTPWLIVLMHVPL-Y----------NSNEAHYMEGE--SMRS----VFESW-FIH 341
GmPAP3|Q6YGT9|/1-512 340 EKTPWLIVLMHVPL-Y----------NSNGAHYMEGE--SMRS----VFESW-FIE 377
LaAP2|Q9XJ24|/1-638 305 KETPWLIVLVHSPW-Y----------NSYNYHFMEGE--TMRV----MFESW-LVQ 342
LlPPD4|Q8VXF4|/1-629 464 KHQPWLIFSAHRPLAY----------SSNAWYGMEGS--FEEPEGREHLQKL-WQK 506
LlPPD1|Q8VX11|/1-615 450 QKQPWLIFLAHRVLGY----------SSAGFYVQEGS--FEEPMGREDLQHL-WQK 492
LlPPD2|Q8VXF6|/1-612 447 QKQPWLIFLAHRVLGY----------SSCICYAEEGS--FAEPMGRESLQKL-WQK 489
TnPAP1|Q4RLR4|/1-378 213 ALRPWIITMGHRPM-YCSDDDQDDC-TKFDSYVRLGR--NDTRPPAPGLEDL-LYR 263
HsPAP7|Q6ZNF0|/1-438 276 AARPWIITMGHRPM-YCSNADLDDC-TRHESKVRKGL--QGKL---YGLEDL-FYK 323
CePAP3|Q9NAM9|/1-418 262 NTKKWTIVMFHRPW-YCSSKKKKGCNDDQDILSREGD--KKKF---PGLEEL-LNQ 310
MmPAP7|Q8BX37|/1-438 276 VARPWIITMGHRPM-YCSNADLDDC-TRHESRVRKGL--HGKL---FGLEDL-FHK 323
DmPAP1|Q9VZ56|/1-458 295 NKRPWIILYGHRPM-YCSNENDNDC-THSETLTRVGW--PFVH--MFGLEPL-LYE 343
DmPAP2|Q9VZ58|/1-450 285 AKRPWIITYGHRPM-YCSDDKEYDCNSQLETYIRQGL--PMLK--WFGLEDL-FYK 334
AmPAP|A0A087ZWE4|/1-438 270 AQRPWIVTFGHRPM-YCSNANADDC-TNHESLVRVGL--PIVN--WFGLEDL-FFK 318
CePAP1|O01320|/1-419 256 SSAQYLIISGHYPV------------HSMSSH-GPTD--CLRQ----RLDPL-LKR 291
DmPAP3|Q9VZ57|/1-453 284 KKRPWIITYGHRPM-YCSNDNGDDC-ANHETIVRKGL--PMLD--FFGLEPL-FYQ 332
AgPAP|Q7PUN5|/1-463 276 KLRPWIVTYGHRPM-YCSNDNDNDC-THSETLVRVGL--PFMH--WFGLEDL-FYE 324

```
HvPAPhy_a|C4PKL2|/1-544     393 HGLDIAFTGHVHAYER------SNRVFNYTL-----------DPCGAVYISVGDGG 431
TaPAPhy_a1|C4PKK7|/1-550    390 HGLDIAFTGHVHAYER------SNRVFNYTL-----------DPCGAVHISVGDGG 428
TaPAPhy_b1|C4PKK9|/1-538    389 YGLDIVFTGHVHAYER------SNRVFNYTL-----------DPCGAVHISVGDGG 427
TaPAPhy_b2|C4PKL0|/1-537    388 YGLDIVFTGHVHAYER------SNRVFNYTL-----------DPCGAVHISVGDGG 426
HvPAPhy_b2|C4PKL4|/1-537    388 YGIDIVFTGHVHAYER------SNRVFNYTL-----------DPCGAVHISVGDGG 426
HvPAPhy_b1|C4PKL3|/1-536    387 YGIDIVFTGHVHAYER------SNRVFNYTL-----------DPCGAVHISVGDGG 425
OsPAPhy_b|D6QSX9|/1-539     388 YAVDVVFTGHVHAYER------SNRVFNYTL-----------DPCGPVHISVGDGG 426
ZmPAPhy_b|C4PKL6|/1-544     393 YGVDVVFTGHVHAYER------SNRVFNYTL-----------DACGPVHISVGDGG 431
MtPAPhy|Q3ZFI1|/1-543       394 YGVDIVFNGHVHAYER------SNRVYNYTL-----------DPCGPVYITVGDGG 432
PtPAP3|V9LXK5|/1-564        409 YGVDVVFSGHVHAYER------SNRVYNYTL-----------DPCGPVHITVGDGG 447
NtPAPhy|A5YBN1|/1-551       393 CGVDLVFNGHVHAYER------SNRVYNYTL-----------DPCGPVYITVGDGG 431
LaPAPhy|D2YZL4|/1-543       391 YGVDIVLNGHIHAYER------SNRVYNYNL-----------DPCGPVHITIGDGG 429
GmPAPhy_b|Q93XG4|/1-547     395 YGVDIIFNGHVHAYER------SNRVYNYNL-----------DPCGPVYITVGDGG 433
AtPAP15|Q9SFU3|/1-532       387 YGTDIVFNGHVHAYER------SNRVYNYEL-----------DPCGPVYIVIGDGG 425
AtaPAPhy_a1|F6MIX0|/1-549   389 HGLDIAFTGHVHAYER------SNRVFNYTL-----------DPCGAVHISVGDGG 427
ScPAPhy_a2|F6MIX4|/1-543    392 HGLDIAFTGHVHAYER------SNRVFNYTL-----------DPCGAVHISVGDGG 430
TmPAPhy_a1|F6MIW8|/1-545    385 HGLDIAFTGHVHAYER------SNRVFNYTL-----------DPCGAVHISVGDGG 423
TaPAPhy_a3|F6MIW2|/1-539    388 HGLDIAFTGHVHAYER------SNRVFNYTL-----------DPCGAVHISVGDGG 426
TaPAPhy_a2|C4PKK8|/1-549    389 HGLDIAFTGHVHAYER------SNRVFNYTL-----------DPCGAVHISVGDGG 427
ScPAPhy_a1|F6MIX2|/1-541    388 HGLDIAFTGHVHAYER------SNRVFNYTL-----------DPCGAVHISVGDGG 426
TaPAPhy_b3|F6MIW6|/1-536    387 YGLDIVFTGHVHAYER------SNRVFNYTL-----------DPCGAVHISVGDGG 425
TmPAPhy_b1|F6MIW9|/1-539    390 YGLDIVFTGHVHAYER------SNRVFNYTL-----------DPCGAVHISVGDGG 428
AtaPAPhy_b1|F6MIX1|/1-538   389 YGLDIVFTGHVHAYER------SNRVFNYTL-----------DPCGAVHISVGDGG 427
ScPAPhy_b1|F6MIX5|/1-538    389 YGLDIVFTGHVHAYER------SYRVFNYTL-----------DPCGAVHISVGDGG 427
RcPAP1|B9RWG6|/1-566        415 YGVDMVFNGHVHAYER------SNRVYNYTL-----------DPCGPVHITVGDGG 453
VvPAP|A5BGI6|/1-540         388 YGVDIVFNGHVHAYER------SNRVYNYTL-----------DPCGPVHIMVGDGG 426
PvPAPhy|V7B3Z4|/1-546       395 YGVDIVFNGHVHAYER------SNRVYNYSL-----------DPCGPVHIAVGDGG 433
VrPAPhy|B5ARZ7|/1-547       396 YGVDIVFNGHVHAYER------SNRVYNYSL-----------DPCGPVHIAVGDGG 434
AlPAP15|D7L636|/1-532       387 YGIDIVFNGHVHAYER------SNRVYNYEL-----------DPCGPVYIVVGDGG 425
AtPAP23|Q6TPH1|/1-458       388 YRVDIVFAGHVHAYER------MNRIYNYTL-----------DPCGPVYITIGDGG 426
GmPAP4|V9HXG4|/1-442        324 ASVDLVIAGHVHAYER------SKRLYNGRL-----------DPCGAVHITIGDGG 362
ZmPAP_c|C4PKL7|/1-566       415 YQVDIVFSGHVHAYER------MNRVFNYTL-----------DPCGPIYIGIGDGG 453
SbPAP|A0A1Z5R9T8|/1-566     415 YQVDIVFTGHVHAYER------MNRVFNYTL-----------DPCGPVYIGIGDGG 453
HvPAP_c|C4PKL5|/1-564       413 HGVDIVFSGHVHAYER------MNRVFNYTL-----------DSCGPVYITIGDGG 451
PpPAP|A9SPI2|/1-557         396 YKVNIVFSGHVHAYER------TNQVYNYTL-----------NPCGPVYVTVGDGG 434
OsPAP3|Q6ZCX8|/1-622        418 HGVDIVFSGHVHAYER------MNRVFNYTL-----------DPCGPVYITIGDGG 456
OsPAP4|B8B909|/1-622        418 HGVDIVFSGHVHAYER------MNRVFNYTL-----------DPCGPVYITIGDGG 456
AtPAP5|Q9C927|/1-396        278 NKVDIVFAGHVHAYER------SERVSNIQYNITDGMSTPVKDQNAPVYITIGDGG 327
AtPAP20|Q9LXI7|/1-427       321 ARVDLVFAGHVHAYER------FSRVYQDKF-----------DKCGPVYINIGDGG 359
AtPAP22|Q8S340|/1-434       320 ARVDVVFSGHVHAYER------FKRVYNNKA-----------DPCGPIHITIGDGG 358
IbPAP3|Q9ZP18|/1-427        305 YKVDVVFAGHVHSYER------TERVSNVAYNIVNGLCSPKNDSSAPVYITIGDGG 354
AtPAP21|Q9LXI4|/1-437       324 AQVDVVFAGHVHTYER------FKPIYNKKA-----------DPCGPMYITIGDGG 362
LpPAP|Q9MB07|/1-455         338 YKVDVIFAGHVHAYER------SYRISNVAYNITDGKCTPTSDLSAPVYITVGDGG 387
RcPAP2|B9SXP8|/1-463        318 YKVDVIFSGHVHAYER------SYRFSNVRSSVSSPNCYPVANESAPMYITVGDGG 367
IbPAP2|Q9SDZ9|/1-465        343 HKVDLVFAGHVHAYER------SERVSNVAYDIVNGKCTPVRDQSAPVYITIGDGG 392
AtPAP11|Q9SI18|/1-441       323 NKVDIVFAGHVHAYER------SKRISNIHYNITDGMSTPVKDQNAPIYITIGDGG 372
GmPAP1|Q09131|/1-464        341 YKVDVVFAGHVHAYER------SERVSNVAYNIVNGLCAPVNDKSAPVYITIGDGG 390
AtPAP25|O23244|/1-466       342 SKVDLVLSGHVHSYER------SERVSNIKYNITNGLSYPVKDPSAPIYITIGDGG 391
AtPAP12|Q38924|/1-469       347 YKVDVVFAGHVHAYER------SERVSNIAYNIVNGLCEPISDESAPIYITIGDGG 396
NtPAP|Q84KZ3|/1-461         343 YKVDMVFAGHVHAYER------SKRISNIDYKIVSGECTPASNPSAPVYITVGDGG 392
MtPAP1|Q4KU02|/1-465        342 YKVDVVYAGHVHAYER------SERVSNVAYNVVNGICTPIKDQSAPVYITIGDGG 391
OsPAP2|Q8S505|/1-476        339 YKVDLVFAGHVHAYER------SYRISNINYNITSGNRYPVPDKSAPVYITVGDGG 388
LaAP1|Q93VM7|/1-460         339 YKVDVVFAGHVHAYER------SERVSNNKYNITNGICTPVEDITAPIYITNGDGG 388
PvPAP2|Q764C1|/1-457        346 YKVDVIFAGHVHAYER------SYRFSNIDYNITNGNRYPLPDKSAPVYITVGDGG 395
LlAP2|Q8L6L1|/1-463         341 YKGDVVFAGHVHAYER------PERVSNDKYNITNGICTPVKDISAPVYITNGDGG 390
AtPAP10|Q9SIV9|/1-468       346 YKVDVVFAGHVHAYER------SERVSNIAYNVVNGICTPVKDQSAPVYITIGDGG 395
PvPAP1|P80366|/1-459        341 YKVDVVFAGHVHAYER------SERVSNIAYKITNGLCTPVKDQSAPVYITIGDAG 390
TaACP|C4PKL1|/1-477         340 YKVDLVFAGHVHAYER------SYRISNINYNVTSGNRYPVPDKSAPVYITVGDGG 389
AtPAP6|Q9C510|/1-466        342 SKVDLVLSGHVHAYER------SERISNIKYNITNGLSSPVKDPNAPIYITIGDGG 391
AcPAP|Q93WP4|/1-481         345 YKVDLVFAGHVHAYER------SYRISNIVYNITSGNRYPIPDKSAPVYITVGDGG 394
AoPAP32|Q9XF09|/1-470       348 YKVDLVFAGHVHAYER------TERISNIVYNVVNGICTPVNDSSAPIYITIGDGG 397
StPAP3|Q6J5M8|/1-477        339 YKVDVIFAGHVHAYER------SYRISNIHYNVSGGDAYPVPDKAAPIYITVGDGG 388
IbPAP1|Q9SE00|/1-473        351 YKVDIVFSGHVHSYER------SERVSNVAYNIVNAKCTPVSDESAPVYITIGDGG 400
AtPAP26|Q949Y3|/1-475       340 HKVDVIFAGHVHAYER------SYRISNVRYNVSSGDRYPVPDKSAPVYITVGDGG 389
RcPAP3|B9SXP6|/1-488        346 YKVDIIFAGHVHAYER------SYRISNIQYNVSSGERYPIADKSAPVYITVGDGG 395
LlAP1|Q8L5E1|/1-477         342 YEVDVIFAGHVHAYER------SYRFSNTDYNITSGHRFPIADKSAPVYITVGDGG 391
GmPAP3|Q6YGT9|/1-512        378 YKVDVIFAGHVHAYER------SYRYSNVDYNITGGNRYPLPNKSAPVYITVGDGG 427
LaAP2|Q9XJ24|/1-638         343 YKVDVVFAGHVHAYER------SECVSNVEVRHCKWQVYPCKDQSAPVYITIGDGG 392
LlPPD4|Q8VXF4|/1-629        507 YKVDIAFYGHVHNYER------ICPIYQNQCVNSEKTHYSG-TVNGTIHVVVGGGG 555
LlPPD1|Q8VX11|/1-615        493 YKVDIAMYGHVHNYER------TCPIYQNVCTNKEKHNYKG-NLNGTIHVVVGGGG 541
LlPPD2|Q8VXF6|/1-612        490 YKVDIAIYGHVHNYER------TCPIYQNICTSEEKHHYKG-TLNGTIHIVAGGAG 538
TnPAP1|Q4RLR4|/1-378        264 YGVDLELWAHEHTYER------LWPVYGDKVWNGS-TEQPYVKPRAPVHIITGSAG 312
HsPAP7|Q6ZNF0|/1-438        324 YGVDLQLWAHEHSYER------LWPIYNYQVFNGS-REMPYTNPRGPVHIITGSAG 372
CePAP3|Q9NAM9|/1-418        311 YKVDMVLYGHKHTYER------MWPIYNKNPFKSA-NPGHIKNAPAPVYILTGGAG 359
MmPAP7|Q8BX37|/1-438        324 YGVDLEFWAHEHSYER------LWPIYNYQVFNGS-LESPYTNPRGPVHIITGSAG 372
DmPAP1|Q9VZ56|/1-458        344 FGVDVAIWAHEHSYER------LWPIYDYKVRNGTLKDSPYNDPSAPVHIVTGSAG 393
DmPAP2|Q9VZ58|/1-450        335 HGVDVEIFAHEHFYTR------LWPIYDYKVYNGS-AEAPYTNPKAPIQIITGSAG 383
AmPAP|A0A087ZWE4|/1-438     319 YKVDLLLWAHEHSYER------LWPMYNFKVQNGS-YEKPYKNYKAPVHIVTGSAG 367
CePAP1|O01320|/1-419        292 FNVNAYFSGHDHSLQHFTFPGYGEHIINYVVSGAASRA----DASTK-HI---KEF 339
DmPAP3|Q9VZ57|/1-453        333 YGVDIELWAHEHCYER------MWPMYNYTVFNGS-LAEPYVNPGAPIHIISGAAG 381
AgPAP|Q7PUN5|/1-463         325 HGVDVEIWAHEHSYER------LFPIYDYKVYNGS-YEEPYRNPRAPVHLVTGSAG 373
```

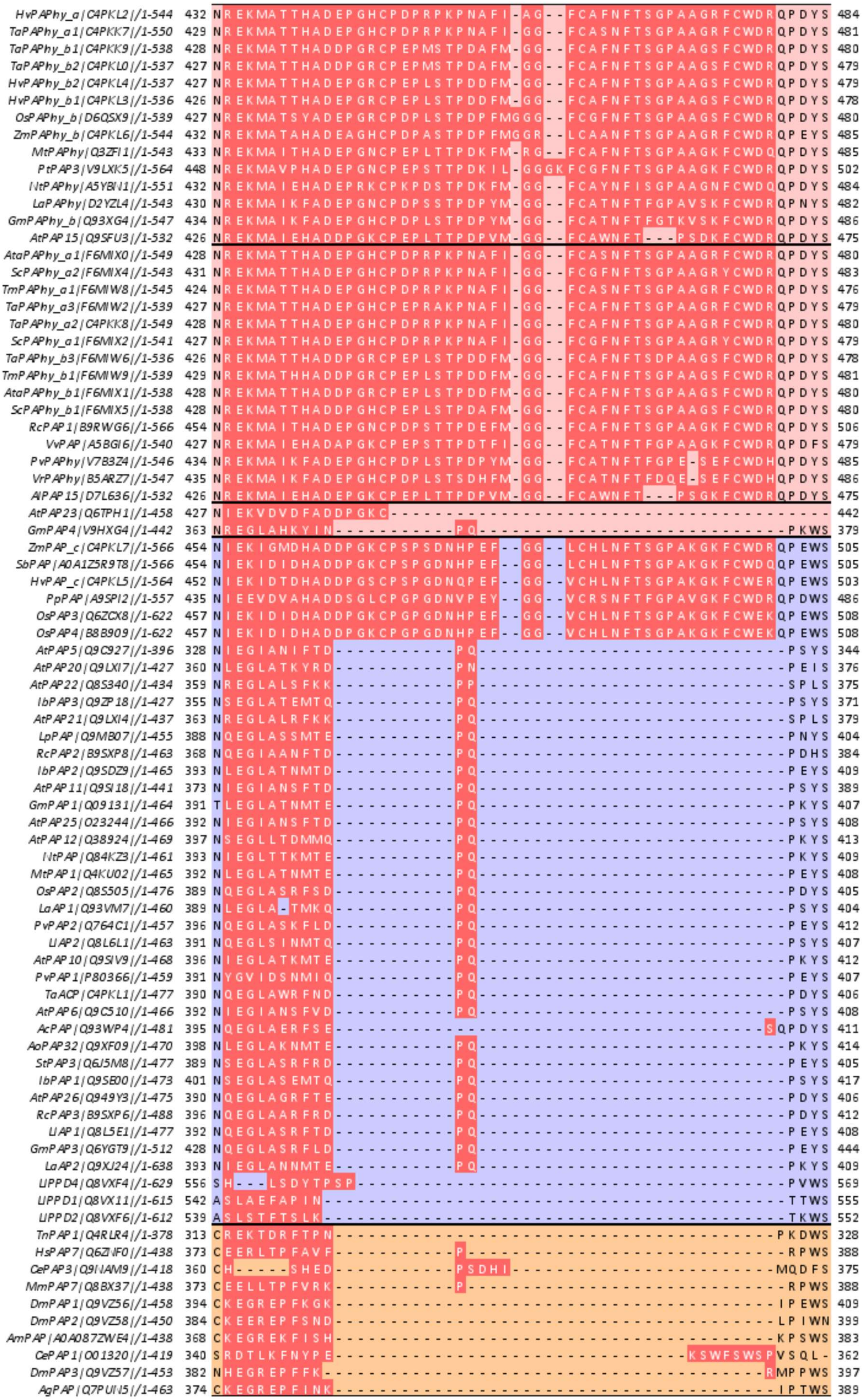

HvPAPhy_a|C4PKL2|/1-544	485	AYRESSFGHGILEVKNETHAL-----WRWHRNQDL---------------------	514
TaPAPhy_a1|C4PKK7|/1-550	482	AYRESSFGHGILEVKNETHAL-----WRWHRNQDH---------------------	511
TaPAPhy_b1|C4PKK9|/1-538	481	AYRESSFGHGILEVKNETYAL-----WKWHRNQDL---------------------	510
TaPAPhy_b2|C4PKL0|/1-537	480	AYRESSFGHGILEVKNETHAL-----WKWHRNQDL---------------------	509
HvPAPhy_b2|C4PKL4|/1-537	480	AYRESSFGHGILEVKNETHAL-----WKWHRNQDL---------------------	509
HvPAPhy_b1|C4PKL3|/1-536	479	AYRESSFGHGILEVKNETHAL-----WKWHRNQDL---------------------	508
OsPAPhy_b|D6QSX9|/1-539	481	AYRESSFGHGILEVKNETHAL-----WRWHRNQDL---------------------	510
ZmPAPhy_b|C4PKL6|/1-544	486	AYRESSFGHGVLEVRNDTHAL-----WRWHRNQDL---------------------	515
MtPAPhy|Q3ZFI1|/1-543	486	AFRESSFGHGILEVKNETHAL-----WSWNRNQDY---------------------	515
PtPAP3|V9LXK5|/1-564	503	AFRESSFGHGILEVKNETHAL-----WTWHRNQDF---------------------	532
NtPAPhy|A5YBN1|/1-551	485	AYRESSFGHGILEVKSETHAL-----WTWHRNQDM---------------------	514
LaPAPhy|D2YZL4|/1-543	483	AFRESSFGYGILEVKNETWAL-----WSWYRNQDS---------------------	512
GmPAPhy_b|Q93XG4|/1-547	487	AFRESSFGYGILEVKNETWAL-----WSWYRNQDS---------------------	516
AtPAP15|Q9SFU3|/1-532	476	ALRESSFGHGILEMKNETWAL-----WTWYRNQDS---------------------	505
AtaPAPhy_a1|F6MIX0|/1-549	481	AYRESSFGHGILEVKNETHAL-----WRWHRNQDH---------------------	510
ScPAPhy_a2|F6MIX4|/1-543	484	AYRESSFGHGILEVKNETHAL-----WRWHRNQDM---------------------	513
TmPAPhy_a1|F6MIW8|/1-545	477	AYRESSFGHGILEVKNETHAL-----WRWHRNQDH---------------------	506
TaPAPhy_a3|F6MIW2|/1-539	480	AYRESSFGHGILEVKNETHAL-----WRWHRNQDM---------------------	509
TaPAPhy_a2|C4PKK8|/1-549	481	AYRESSFGHGILEVKNETHAL-----WRWHRNQDM---------------------	510
ScPAPhy_a1|F6MIX2|/1-541	480	AYRESSFGHGILEVKNETHAL-----WRWHRNQDM---------------------	509
TaPAPhy_b3|F6MIW6|/1-536	479	AYRESSFGHGILEVKNETHAL-----WKWHRNQDL---------------------	508
TmPAPhy_b1|F6MIW9|/1-539	482	AYRESSFGHGILEVKNETHAL-----WKWHRNQDL---------------------	511
AtaPAPhy_b1|F6MIX1|/1-538	481	AYRESSFGHGILEVKNETHAL-----WKWHRNQDL---------------------	510
ScPAPhy_b1|F6MIX5|/1-538	481	AYRESSFGHGILEVKNETHAL-----WKWHRNQDL---------------------	510
RcPAP1|B9RWG6|/1-566	507	AYRESSFGHGILEVKNETHAL-----WTWHRNQDL---------------------	536
VvPAP|A5BGI6|/1-540	480	AFRESSFGHGILEVKNDTWAL-----WTWYRNQDS---------------------	509
PvPAPhy|V7B3Z4|/1-546	486	AFRETSFGYGILEVKNETWAL-----WSWYRNQDS---------------------	515
VrPAPhy|B5ARZ7|/1-547	487	AFRETSFGYGILEVKNETWAL-----WSWYRNQDS---------------------	516
AlPAP15|D7L636|/1-532	476	AMRESSFGHGILEMKNETWAL-----WTWYRNQDS---------------------	505
AtPAP23|Q6TPH1|/1-458	443	---------------HSSYDL-----FFF---------------------------	451
GmPAP4|V9HXG4|/1-442	380	EFREASFGHGELKIVNSTHAF-----WSWHRNDDD---------------------	409
ZmPAP_c|C4PKL7|/1-566	506	AYRESSFGHGILEVLNSTYAL-----WTWHRNQDA---------------------	535
SbPAP|A0A1Z5R9T8|/1-566	506	AYRESSFGHGILEVLNSTYAL-----WTWHRNQDA---------------------	535
HvPAP_c|C4PKL5|/1-564	504	AFRESSFGHGILEVVNSTYAL-----WTWHRNQDT---------------------	533
PpPAP|A9SPI2|/1-557	487	AFRESSFGHGVLEVVNSSHAL-----WTWHRNQDM---------------------	516
OsPAP3|Q6ZCX8|/1-622	509	AFRESSFGHGILEVVNSTYAL-----WTWHRNQDA---------------------	538
OsPAP4|B8B909|/1-622	509	AFRESSFGHGILEVVNSTYAL-----WTWHRNQDA---------------------	538
AtPAP5|Q9C927|/1-396	345	AFREASFGHALLEIKNRTHAH-----YTWHRNKED---------------------	374
AtPAP20|Q9LXI7|/1-427	377	LFREASFGHGQLVVENATHAR-----WEWHRNDDD---------------------	406
AtPAP22|Q8S340|/1-434	376	EFREASFGHGRLKVMDGKRAH-----WSWHRNNDS---------------------	405
IbPAP3|Q9ZP18|/1-427	372	AYREASFGHGIFDIKNRTHAH-----FGWHRNQDG---------------------	401
AtPAP21|Q9LXI4|/1-437	380	EFRESSFGHGRLRIIDHKRAH-----WSWHRNNDE---------------------	409
LpPAP|Q9MB07|/1-455	405	AYREASFGHAIFGIKNRTHAY-----YNWYRNQDG---------------------	434
RcPAP2|B9SXP8|/1-463	385	AFREASYGHSTLEIMNKTHAF-----YYWHRNDDG---------------------	414
IbPAP2|Q9SDZ9|/1-465	410	AFREASFGHATLDIKNRTHAY-----YSWHRNQDG---------------------	439
AtPAP11|Q9SI18|/1-441	390	AFREASFGHALLEIKNRTHAH-----YTWHRNKED---------------------	419
GmPAP1|Q09131|/1-464	408	AFREASFGHAIFDITNRTHAH-----YSWHRNQDG---------------------	437
AtPAP25|O23244|/1-466	409	AYREASFGHAVLEIYNRTHAY-----YTWHRNQDN---------------------	438
AtPAP12|Q38924|/1-469	414	AFREASFGHGLLEIKNRTHAY-----FSWNRNQDG---------------------	443
NtPAP|Q84KZ3|/1-461	410	AYRESSFGHAILEIKNRTHAY-----YSWHRNQDG---------------------	439
MtPAP1|Q4KU02|/1-465	409	AYREASFGHAIFDIKNRTHAH-----YSWHRNQDG---------------------	438
OsPAP2|Q8S505|/1-476	406	AFREASYGHSILQLKNRTHAI-----YQWNRNDDG---------------------	435
LaAP1|Q93VM7|/1-460	405	AYRKASFGHGIFAIKNRTHAH-----YSWNRNQDG---------------------	434
PvPAP2|Q764C1|/1-457	413	AFREASYGHSTLEIKNRTHAI-----YHWNRNDDG---------------------	442
LlAP2|Q8L6L1|/1-463	408	AYREASFGHGTLEIKNRTHAH-----YSWNRNQDG---------------------	437
AtPAP10|Q9SIV9|/1-468	413	AFREASFGHAIFSIKNRTHAH-----YGWHRNHDG---------------------	442
PvPAP1|P80366|/1-459	408	AFREASFGHGMFDIKNRTHAH-----FSWNRNQDG---------------------	437
TaACP|C4PKL1|/1-477	407	AFREASFGHSTLQLVNRTHAV-----YQWNRNDDG---------------------	436
AtPAP6|Q9C510|/1-466	409	AYREASFGHAVLEIMNRTHAQ-----YTWHRNQDN---------------------	438
AcPAP|Q93WP4|/1-481	412	AFRESSYGHSTLELRNRTHAF-----YQWNRNDDG---------------------	441
AoPAP32|Q9XF09|/1-470	415	AFREASFGHATLDIKNRTHAY-----YAWHRNQDG---------------------	444
StPAP3|Q6J5M8|/1-477	406	AFREASYGHSTLDIKNRTHAI-----YHWNRNDDG---------------------	435
IbPAP1|Q9SE00|/1-473	418	AFREASFGHGIFDIKNRTHAH-----FSWHRNQDG---------------------	447
AtPAP26|Q949Y3|/1-475	407	AFREASYGHSTLDIKNRTHAI-----YHWNRNDDG---------------------	436
RcPAP3|B9SXP6|/1-488	413	AFREASFGHSTLEIKNRTHAF-----YQWNRNDDG---------------------	442
LlAP1|Q8L5E1|/1-477	409	AFREASYGHSTLEIKNRTHAI-----YHWNRNDDG---------------------	438
GmPAP3|Q6YGT9|/1-512	445	AFREASYGHSTLEIKNRTHAI-----YHWNRNDDG---------------------	474
LaAP2|Q9XJ24|/1-638	410	AYREASFGHAIFDIKNRTVLGLFSENYRLHTKQEEDEKNLASKGAMVKGVILQQVV	465
LjPPD4|Q8VXF4|/1-629	570	VFRDRDFGFGKLTAFNHSYLL-----FEYKRSSD----------------------	598
LjPPD1|Q8VX11|/1-615	556	IFKDHDFGFVKLTAFDHSNLL-----LEYRKSSD----------------------	584
LjPPD2|Q8VXF6|/1-612	553	IFKDYDHGFVKLTAFDHSNLL-----FEYKKSRD----------------------	581
TnPAP1|Q4RLR4|/1-378	329	AFRSRDYGYTRMQVVNATHLY-----LEQVSDDQY---------------------	358
HsPAP7|Q6ZNF0|/1-438	389	AVRVKEYGYTRLHILNGTHIH-----IQQVSDDQD---------------------	418
CePAP3|Q9NAM9|/1-418	376	VKALGEYGYTYLTVYNSTHIS-----TDYVDTSST---------------------	405
MmPAP7|Q8BX37|/1-438	389	AVRVKEYGYTRMHILNGTHMH-----IQQVSDDQD---------------------	418
DmPAP1|Q9VZ56|/1-458	410	AFHSQDYGYTRLKAHNRTHIH-----FEQV-SDDK---------------------	438
DmPAP2|Q9VZ58|/1-450	400	AYHSNDYGYTRLKAHNGTHLH-----FEQVSDDQN---------------------	429
AmPAP|A0A087ZWE4|/1-438	384	AYRSSDYGYTRMKVYNQTHLY-----LEQV-SDDK---------------------	412
CePAP1|O01320|/1-419	363	GFRKGGLIYAEFGHYNARLDF-----FDKR--------------------------	387
DmPAP3|Q9VZ57|/1-453	398	AFHSQDFGYLRLKAHNGTHLH-----FEQV-SDDK---------------------	426
AgPAP|Q7PUN5|/1-463	390	AIHSRDYGYTRMKAINGSHLY-----FEQI-SVDK---------------------	418

HvPAPhy_a|C4PKL2|/1-544	515	--------Y-GSA-GDE---------------------------IYI--VREPERC	531
TaPAPhy_a1|C4PKK7|/1-550	512	--------Y-GSA-GDE---------------------------IYI--VREPHRC	528
TaPAPhy_b1|C4PKK9|/1-538	511	--------YQGAV-GDE---------------------------IYI--VREPERC	528
TaPAPhy_b2|C4PKL0|/1-537	510	--------YQGAV-GDE---------------------------IYI--VREPERC	527
HvPAPhy_b2|C4PKL4|/1-537	510	--------YQGAV-GDE---------------------------IYI--VREPGRC	527
HvPAPhy_b1|C4PKL3|/1-536	509	--------YQGAV-GDE---------------------------IYI--VREPERC	526
OsPAPhy_b|D6QSX9|/1-539	511	--------Y-GSV-GDE---------------------------IYI--VREPDKC	527
ZmPAPhy_b|C4PKL6|/1-544	516	--------HAANVAADE---------------------------VYI--VREPDKC	534
MtPAPhy|Q3ZFI1|/1-543	516	--------Y-GTA-GDE---------------------------IYI--VRQPDKC	532
PtPAP3|V9LXK5|/1-564	533	--------Y-EAA-GDQ---------------------------IYI--VRQPDLC	549
NtPAPhy|A5YBN1|/1-551	515	--------Y-NKA-GDI---------------------------IYI--VRQPEKC	531
LaPAPhy|D2YZL4|/1-543	513	--------Y-NEV-GDQ---------------------------IYI--VRQPHLC	529
GmPAPhy_b|Q93XG4|/1-547	517	--------Y-KEV-GDQ---------------------------IYI--VRQPDIC	533
AtPAP15|Q9SFU3|/1-532	506	--------S-SEV-GDQ---------------------------IYI--VRQPDRC	522
AtaPAPhy_a1|F6MIX0|/1-549	511	--------Y-GSA-GDE---------------------------IYI--VREPHRC	527
ScPAPhy_a2|F6MIX4|/1-543	514	--------Y-GSA-GDE---------------------------IYI--VREPERC	530
TmPAPhy_a1|F6MIW8|/1-545	507	--------Y-GSA-GDE---------------------------IYI--VREPHRC	523
TaPAPhy_a3|F6MIW2|/1-539	510	--------Y-GSA-GDE---------------------------IYI--VREPHRC	526
TaPAPhy_a2|C4PKK8|/1-549	511	--------Y-GSA-GDE---------------------------IYI--VREPHRC	527
ScPAPhy_a1|F6MIX2|/1-541	510	--------Y-GSA-GDE---------------------------IYI--VREPERC	526
TaPAPhy_b3|F6MIW6|/1-536	509	--------YQGGV-GDE---------------------------IYI--VREPERC	526
TmPAPhy_b1|F6MIW9|/1-539	512	--------YQGVV-ADE---------------------------IYI--VREPERC	529
AtaPAPhy_b1|F6MIX1|/1-538	511	--------YQGAV-GDE---------------------------IYI--VREPERC	528
ScPAPhy_b1|F6MIX5|/1-538	511	--------YQGAV-GDE---------------------------IFI--VREPERC	528
RcPAP1|B9RWG6|/1-566	537	--------Y-SSA-GDQ---------------------------IYI--VRQQERC	553
VvPAP|A5BGI6|/1-540	510	--------R-DNA-GDQ---------------------------IYI--VRTPDMC	526
PvPAPhy|V7B3Z4|/1-546	516	--------Y-KEV-GDQ---------------------------IYI--VRQPDIC	532
VrPAPhy|B5ARZ7|/1-547	517	--------Y-KEV-GDQ---------------------------IYI--VRQPDIC	533
AlPAP15|D7L636|/1-532	506	--------S-SQV-GDQ---------------------------IYI--VRQPDRC	522
AtPAP23|Q6TPH1|/1-458		--	
GmPAP4|V9HXG4|/1-442	410	--------E-PVK-ADD---------------------------IWITSL-VSSRC	427
ZmPAP_c|C4PKL7|/1-566	536	--------YAENSVGDQ---------------------------IYI--VRQPDKC	554
SbPAP|A0A1Z5R9T8|/1-566	536	--------YGENSVGDQ---------------------------IYI--VRQPDKC	554
HvPAP_c|C4PKL5|/1-564	534	--------YGEHSVGDE---------------------------IYI--VREPDKC	552
PpPAP|A9SPI2|/1-557	517	--------YKEAV-GDQ---------------------------IYI--VRQPDGC	534
OsPAP3|Q6ZCX8|/1-622	539	--------YGEDSVGDQ---------------------------IYI--VRQPDKC	557
OsPAP4|B8B909|/1-622	539	--------YGEDSVGDQ---------------------------IYI--VRQPDKC	557
AtPAP5|Q9C927|/1-396	375	--------E-AVI-ADS---------------------------IWL---------	384
AtPAP20|Q9LXI7|/1-427	407	--------V-SVE-KDS---------------------------VWLTSLLADSSC	425
AtPAP22|Q8S340|/1-434	406	--------N-SLL-ADE---------------------------VWLDSLSTSSSC	424
IbPAP3|Q9ZP18|/1-427	402	--------L-AVE-GDS---------------------------LWF---------	411
AtPAP21|Q9LXI4|/1-437	410	--------M-SSI-ADE---------------------------VSFESPRTSSHC	428
LpPAP|Q9MB07|/1-455	435	--------N-AVE-ADS---------------------------LWF---------	444
RcPAP2|B9SXP8|/1-463	415	----------KKVVADK---------------------------LVL---------	424
IbPAP2|Q9SDZ9|/1-465	440	--------Y-AVE-ADS---------------------------MWV---------	449
AtPAP11|Q9SI18|/1-441	420	--------E-AVI-ADS---------------------------IWL---------	429
GmPAP1|Q09131|/1-464	438	--------V-AVE-ADS---------------------------LWS---------	447
AtPAP25|O23244|/1-466	439	--------E-PVA-ADS---------------------------IML---------	448
AtPAP12|Q38924|/1-469	444	--------N-AVA-ADS---------------------------VWL---------	453
NtPAP|Q84KZ3|/1-461	440	--------F-SAK-ADS---------------------------FLF---------	449
MtPAP1|Q4KU02|/1-465	439	--------Y-SVE-ADS---------------------------HWF---------	448
OsPAP2|Q8S505|/1-476	436	--------K-HVP-ADN---------------------------VVF---------	445
LaAP1|Q93VM7|/1-460	435	--------Y-AVE-ADK---------------------------LWL---------	444
PvPAP2|Q764C1|/1-457	443	--------K-KVP-TDS---------------------------FVL---------	452
LlAP2|Q8L6L1|/1-463	438	--------Y-AVE-ADK---------------------------LWL---------	447
AtPAP10|Q9SIV9|/1-468	443	--------Y-AVE-GDR---------------------------MWF---------	452
PvPAP1|P80366|/1-459	438	--------V-AVE-ADS---------------------------VWF---------	447
TaACP|C4PKL1|/1-477	437	--------K-HVP-TDN---------------------------VVF---------	446
AtPAP6|Q9C510|/1-466	439	--------E-PVA-ADS---------------------------IML---------	448
AcPAP|Q93WP4|/1-481	442	----------KHIPVDR---------------------------IIF---------	451
AoPAP32|Q9XF09|/1-470	445	--------Y-AVE-ADT---------------------------LWI---------	454
StPAP3|Q6J5M8|/1-477	436	----------NNITTDS---------------------------FTL---------	445
IbPAP1|Q9SE00|/1-473	448	--------A-SVE-ADS---------------------------LWL---------	457
AtPAP26|Q949Y3|/1-475	437	--------K-KVA-TDE---------------------------FVL---------	446
RcPAP3|B9SXP6|/1-488	443	--------N-KVA-TDA---------------------------FVL---------	452
LlAP1|Q8L5E1|/1-477	439	--------K-KVP-IDS---------------------------FIL---------	448
GmPAP3|Q6YGT9|/1-512	475	--------K-KVP-TDS---------------------------FVL---------	484
LaAP2|Q9XJ24|/1-638	466	QAVVATLLF-AVT-GNDSQDTNQNASLLVSARQFVIAMLVIDTWQYF---------	510
LlPPD4|Q8VXF4|/1-629	599	----------GNV-YDF---------------------------FTI--SRDYRDV	614
LlPPD1|Q8VX11|/1-615	585	----------GQV-YDS---------------------------FTI--SRDYRDI	600
LlPPD2|Q8VXF6|/1-612	582	----------GKV-YDS---------------------------FKI--SRDYRDI	597
TnPAP1|Q4RLR4|/1-378	359	----------GKV-IDS---------------------------IWV--VKEKHG-	373
HsPAP7|Q6ZNF0|/1-438	419	----------GKI-VDD---------------------------VWV--VRPLFG-	433
CePAP3|Q9NAM9|/1-418	406	--------T-GKF-LDP---------------------------FVL---------	415
MmPAP7|Q8BX37|/1-438	419	----------GKI-VDD---------------------------VWV--VRPL---	431
DmPAP1|Q9VZ56|/1-458	439	--------N-GAI-IDD---------------------------FWL--VKSKHGS	455
DmPAP2|Q9VZ58|/1-450	430	----------GAI-VDS---------------------------FWV--IKDKHGA	445
AmPAP|A0A087ZWE4|/1-438	413	--------E-GAV-LDH---------------------------VWL---------	422
CePAP1|O01320|/1-419	388	--------------GKQ---------------------------LYS--TIIPTRV	400
DmPAP3|Q9VZ57|/1-453	427	--------K-GEV-IDS---------------------------FWV--VKDKHGP	443
AgPAP|Q7PUN5|/1-463	419	--------E-GAV-IDS---------------------------FTI--IKDEHLP	435

HvPAPhy_a|C4PKL2|/1-544	532	-L--HK---HNST---	538
TaPAPhy_a1|C4PKK7|/1-550	529	-L--HK---HNSS---	535
TaPAPhy_b1|C4PKK9|/1-538	529	-L-------LKSS---	533
TaPAPhy_b2|C4PKL0|/1-537	528	-L-------LKSS---	532
HvPAPhy_b2|C4PKL4|/1-537	528	-L-------LSSS---	532
HvPAPhy_b1|C4PKL3|/1-536	527	-L-------LKSS---	531
OsPAPhy_b|D6QSX9|/1-539	528	-L--IK---SSRN---	534
ZmPAPhy_b|C4PKL6|/1-544	535	-L-------AKTA---	539
MtPAPhy|Q3ZFI1|/1-543	533	PPVMPEE-AHNT--	543
PtPAP3|V9LXK5|/1-564	550	-PVQPEAYRLNKP---	561
NtPAPhy|A5YBN1|/1-551	532	-PVKPK---VIKP---	540
LaPAPhy|D2YZL4|/1-543	530	-PINQK---VCRE---	538
GmPAPhy_b|Q93XG4|/1-547	534	-PIHQR---VNID---	542
AtPAP15|Q9SFU3|/1-532	523	-PLHHR---LVNH---	531
AtaPAPhy_a1|F6MIX0|/1-549	528	-L--HK---HNSS---	534
ScPAPhy_a2|F6MIX4|/1-543	531	-L--HK---HNST---	537
TmPAPhy_a1|F6MIW8|/1-545	524	-L--HK---HNST---	530
TaPAPhy_a3|F6MIW2|/1-539	527	-L--HK---HNST---	533
TaPAPhy_a2|C4PKK8|/1-549	528	-L--HK---HNST---	534
ScPAPhy_a1|F6MIX2|/1-541	527	-LHKHK---HNST---	535
TaPAPhy_b3|F6MIW6|/1-536	527	-L-------LKSS---	531
TmPAPhy_b1|F6MIW9|/1-539	530	-L-------LKSS---	534
AtaPAPhy_b1|F6MIX1|/1-538	529	-L-------LKSS---	533
ScPAPhy_b1|F6MIX5|/1-538	529	-L-------LKSS---	533
RcPAP1|B9RWG6|/1-566	554	-PVKPK-GAINVL---	564
VvPAP|A5BGI6|/1-540	527	-PTLSA---VTKL---	535
PvPAPhy|V7B3Z4|/1-546	533	-PVPQR---VSGD---	541
VrPAPhy|B5ARZ7|/1-547	534	DVPRK----VCRD---	542
AlPAP15|D7L636|/1-532	523	-PLHHR---LVNH---	531
AtPAP23|Q6TPH1|/1-458	452	---------NSLN---	455
GmPAP4|V9HXG4|/1-442	428	---VDQ---KTHE---	434
ZmPAP_c|C4PKL7|/1-566	555	LLQPASA--SSLN---	565
SbPAP|A0A1Z5R9T8|/1-566	555	LLQPTNA--SSLN---	565
HvPAP_c|C4PKL5|/1-564	553	LL-------QPRG---	558
PpPAP|A9SPI2|/1-557	535	-PYSSMKNYRDRK---	546
OsPAP3|Q6ZCX8|/1-622	558	LLQTTS---ASSE---	567
OsPAP4|B8B909|/1-622	558	LLQTTS---ASSE---	567
AtPAP5|Q9C927|/1-396	385	---------KNRY---	388
AtPAP20|Q9LXI7|/1-427	426	------------K---	426
AtPAP22|Q8S340|/1-434		--	
IbPAP3|Q9ZP18|/1-427	412	---------INRY---	415
AtPAP21|Q9LXI4|/1-437	429	---------HSNR---	432
LpPAP|Q9MB07|/1-455	445	---------FNRV---	448
RcPAP2|B9SXP8|/1-463	425	---------HNQY---	428
IbPAP2|Q9SDZ9|/1-465	450	---------SNRF---	453
AtPAP11|Q9SI18|/1-441	430	---------KKRY---	433
GmPAP1|Q09131|/1-464	448	---------FNRY---	451
AtPAP25|O23244|/1-466	449	---------HNRY---	452
AtPAP12|Q38924|/1-469	454	---------LNRF---	457
NtPAP|Q84KZ3|/1-461	450	---------FNRY---	453
MtPAP1|Q4KU02|/1-465	449	---------FNRF---	452
OsPAP2|Q8S505|/1-476	446	---------HNQY---	449
LaAP1|Q93VM7|/1-460	445	---------FNRY---	448
PvPAP2|Q764C1|/1-457	453	---------HNQY---	456
LlAP2|Q8L6L1|/1-463	448	---------FNRY---	451
AtPAP10|Q9SIV9|/1-468	453	---------YNRF---	456
PvPAP1|P80366|/1-459	448	---------FNRH---	451
TaACP|C4PKL1|/1-477	447	---------HNQY---	450
AtPAP6|Q9C510|/1-466	449	---------HNRH---	452
AcPAP|Q93WP4|/1-481	452	---------RNQY---	455
AoPAP32|Q9XF09|/1-470	455	---------FNRY---	458
StPAP3|Q6J5M8|/1-477	446	---------HNQY---	449
IbPAP1|Q9SE00|/1-473	458	---------LNRY---	461
AtPAP26|Q949Y3|/1-475	447	---------HNQY---	450
RcPAP3|B9SXP6|/1-488	453	---------HNQY---	456
LlAP1|Q8L5E1|/1-477	449	---------YNQY---	452
GmPAP3|Q6YGT9|/1-512	485	---------HNQY---	488
LaAP2|Q9XJ24|/1-638	511	---------MHRYMHHNKFLYKHIHSQHHRLIVPYSFGALYNHPLVGLILDTIGGA	557
LlPPD4|Q8VXF4|/1-629	615	---------LARV---	618
LlPPD1|Q8VX11|/1-615	601	---------LACS---	604
LlPPD2|Q8VXF6|/1-612	598	---------LACT---	601
TnPAP1|Q4RLR4|/1-378		--	
HsPAP7|Q6ZNF0|/1-438		--	
CePAP3|Q9NAM9|/1-418	416	---------EKL--	418
MmPAP7|Q8BX37|/1-438	432	---------LGRM---	435
DmPAP1|Q9VZ56|/1-458	456	---------YRN--	458
DmPAP2|Q9VZ58|/1-450		--	
AmPAP|A0A087ZWE4|/1-438	423	---------IKDD---	426
CePAP1|O01320|/1-419	401	IPTDTS---TRST---	410
DmPAP3|Q9VZ57|/1-453	444	---------YQSD---	447
AgPAP|Q7PUN5|/1-463	436	---------YKQL---	439

HvPAPhy_a|C4PKL2|/1-544	539	-------------------------------RPAHGP-------------------	544
TaPAPhy_a1|C4PKK7|/1-550	536	-------------------------------RPAHGRSNTTRESGG----------	550
TaPAPhy_b1|C4PKK9|/1-538	534	-------------------------------IAAYF--------------------	538
TaPAPhy_b2|C4PKL0|/1-537	533	-------------------------------IAAYF--------------------	537
HvPAPhy_b2|C4PKL4|/1-537	533	-------------------------------IAAYF--------------------	537
HvPAPhy_b1|C4PKL3|/1-536	532	-------------------------------IAAYF--------------------	536
OsPAPhy_b|D6QSX9|/1-539	535	-------------------------------RIAYY--------------------	539
ZmPAPhy_b|C4PKL6|/1-544	540	-------------------------------RLLAY--------------------	544
MtPAPhy|Q3ZFI1|/1-543		--	
PtPAP3|V9LXK5|/1-564	562	-------------------------------KPQ----------------------	564
NtPAPhy|A5YBN1|/1-551	541	-------------------------------WPIGEYQFDWI--------------	551
LaPAPhy|D2YZL4|/1-543	539	-------------------------------YFAAI--------------------	543
GmPAPhy_b|Q93XG4|/1-547	543	-------------------------------CIASI--------------------	547
AtPAP15|Q9SFU3|/1-532	532	-------------------------------C------------------------	532
AtaPAPhy_a1|F6MIX0|/1-549	535	-------------------------------RPAHGRSNTTRESGG----------	549
ScPAPhy_a2|F6MIX4|/1-543	538	-------------------------------RPAHGR-------------------	543
TmPAPhy_a1|F6MIW8|/1-545	531	-------------------------------RPAHGRQNTTRESGG----------	545
TaPAPhy_a3|F6MIW2|/1-539	534	-------------------------------RPTHGR-------------------	539
TaPAPhy_a2|C4PKK8|/1-549	535	-------------------------------RPAHGRQNTTRESGG----------	549
ScPAPhy_a1|F6MIX2|/1-541	536	-------------------------------RPAHGR-------------------	541
TaPAPhy_b3|F6MIW6|/1-536	532	-------------------------------IAAYF--------------------	536
TmPAPhy_b1|F6MIW9|/1-539	535	-------------------------------IAAYF--------------------	539
AtaPAPhy_b1|F6MIX1|/1-538	534	-------------------------------IAAYF--------------------	538
ScPAPhy_b1|F6MIX5|/1-538	534	-------------------------------IAAYF--------------------	538
RcPAP1|B9RWG6|/1-566	565	-------------------------------VA-----------------------	566
VvPAP|A5BGI6|/1-540	536	-------------------------------WSAAR--------------------	540
PvPAPhy|V7B3Z4|/1-546	542	-------------------------------FIASI--------------------	546
VrPAPhy|B5ARZ7|/1-547	543	-------------------------------FTASI--------------------	547
AlPAP15|D7L636|/1-532	532	-------------------------------C------------------------	532
AtPAP23|Q6TPH1|/1-458	456	-------------------------------LSN----------------------	458
GmPAP4|V9HXG4|/1-442	435	-------------------------------LRSTLLTP-----------------	442
ZmPAP_c|C4PKL7|/1-566	566	-------------------------------W------------------------	566
SbPAP|A0A1Z5R9T8|/1-566	566	-------------------------------W------------------------	566
HvPAP_c|C4PKL5|/1-564	559	-------------------------------VISQDS-------------------	564
PpPAP|A9SPI2|/1-557	547	-------------------------------LPVGPEYQQHT--------------	557
OsPAP3|Q6ZCX8|/1-622	568	-NNCPSEGCPSLVSNSGYGAQKDIIRSGHLIWNASLVIWMILISTVFMKGNLCSRF	622
OsPAP4|B8B909|/1-622	568	-NNCPSEGCPSLVSNSGYGAQKDIIRSGHLIWNAFLVIWMILISTVFMKGNLCSRF	622
AtPAP5|Q9C927|/1-396	389	-------------------------------YLPEEETI-----------------	396
AtPAP20|Q9LXI7|/1-427	427	-------------------------------I------------------------	427
AtPAP22|Q8S340|/1-434	425	-------------------------------WPSSRSNDEL---------------	434
IbPAP3|Q9ZP18|/1-427	416	-------------------------------WMSKEEASVSAV-------------	427
AtPAP21|Q9LXI4|/1-437	433	-------------------------------YRGEI--------------------	437
LpPAP|Q9MB07|/1-455	449	-------------------------------WNPRE----------------ES--	455
RcPAP2|B9SXP8|/1-463	429	-------------------------------WASNLRQQNLQKHHRRSLGDETASN	453
IbPAP2|Q9SDZ9|/1-465	454	-------------------------------WHPVDDSTTTKL-------------	465
AtPAP11|Q9SI18|/1-441	434	-------------------------------YLPEE--------------------	438
GmPAP1|Q09131|/1-464	452	-------------------------------WHPVD--------DSTAHVSH----	464
AtPAP25|O23244|/1-466	453	-------------------------------FFPVE--------------------	457
AtPAP12|Q38924|/1-469	458	-------------------------------WRAQK----------------KTWL	466
NtPAP|Q84KZ3|/1-461	454	-------------------------------WHPVDESY-----------------	461
MtPAP1|Q4KU02|/1-465	453	-------------------------------WHPVDDSTTHVSH------------	465
OsPAP2|Q8S505|/1-476	450	-------------------------------WASNTRRRRLKKKHFHLDQIEDLIS	474
LaAP1|Q93VM7|/1-460	449	-------------------------------WNPLNDSTIHIP-------------	460
PvPAP2|Q764C1|/1-457	457	-------------------------------W------------------------	457
LlAP2|Q8L6L1|/1-463	452	-------------------------------WNPRDDSTIHIP-------------	463
AtPAP10|Q9SIV9|/1-468	457	-------------------------------WHPVDDSPSCNS-------------	468
PvPAP1|P80366|/1-459	452	-------------------------------WYPVD--------DST---------	459
TaACP|C4PKL1|/1-477	451	-------------------------------WAGNTRRRRLKKKHLRYESLQSLMS	475
AtPAP6|Q9C510|/1-466	453	-------------------------------FFPVEEIVSSNIRA-----------	466
AcPAP|Q93WP4|/1-481	456	-------------------------------WASNTRRRRLKKTRPSQAVERLISS	480
AoPAP32|Q9XF09|/1-470	459	-------------------------------WNPVDESTSATA-------------	470
StPAP3|Q6J5M8|/1-477	450	-------------------------------WGSGLRRRKLNKNHLNSVISERPFS	474
IbPAP1|Q9SE00|/1-473	462	-------------------------------WASED--------ASSMSAM-----	473
AtPAP26|Q949Y3|/1-475	451	-------------------------------WGKNIRRRKLKKHYIRSVVGGWIAT	475
RcPAP3|B9SXP6|/1-488	457	-------------------------------WASNPRRRKLKKHHLRSVVGWIAST	481
LlAP1|Q8L5E1|/1-477	453	-------------------------------WGSNRRRRKLKKNFLMTLVDEAVSM	477
GmPAP3|Q6YGT9|/1-512	489	-------------------------------WGHNRRRRKL-KHFLLKVIDEVASM	512
LaAP2|Q9XJ24|/1-638	558	LSFLISGMSPRISIFFFSFATIKTVDDHCGLWLPGNLFHIFSTTILLTMMFTISFS	613
LlPPD4|Q8VXF4|/1-629	619	-------------------------------HDGCDKTTLAT--------------	629
LlPPD1|Q8VX11|/1-615	605	-------------------------------VDSCPTTTLAS--------------	615
LlPPD2|Q8VXF6|/1-612	602	-------------------------------VDSCPRTTLAS--------------	612
TnPAP1|Q4RLR4|/1-378	374	-------------------------------YSAWF--------------------	378
HsPAP7|Q6ZNF0|/1-438	434	-------------------------------RRMYL--------------------	438
CePAP3|Q9NAM9|/1-418		--	
MmPAP7|Q8BX37|/1-438	436	-------------------------------MYH----------------------	438
DmPAP1|Q9VZ56|/1-458		--	
DmPAP2|Q9VZ58|/1-450	446	-------------------------------YPSPQ--------------------	450
AmPAP|A0A087ZWE4|/1-438	427	-------------------------------ILPAYNLNLLDK-------------	438
CePAP1|O01320|/1-419	411	-------------------------------ASPFVEIGM----------------	419
DmPAP3|Q9VZ57|/1-453	448	-------------------------------LNSKTL-------------------	453
AgPAP|Q7PUN5|/1-463	440	-------------------------------LERDEQERLRAKSSGSAEAEANLL-	463

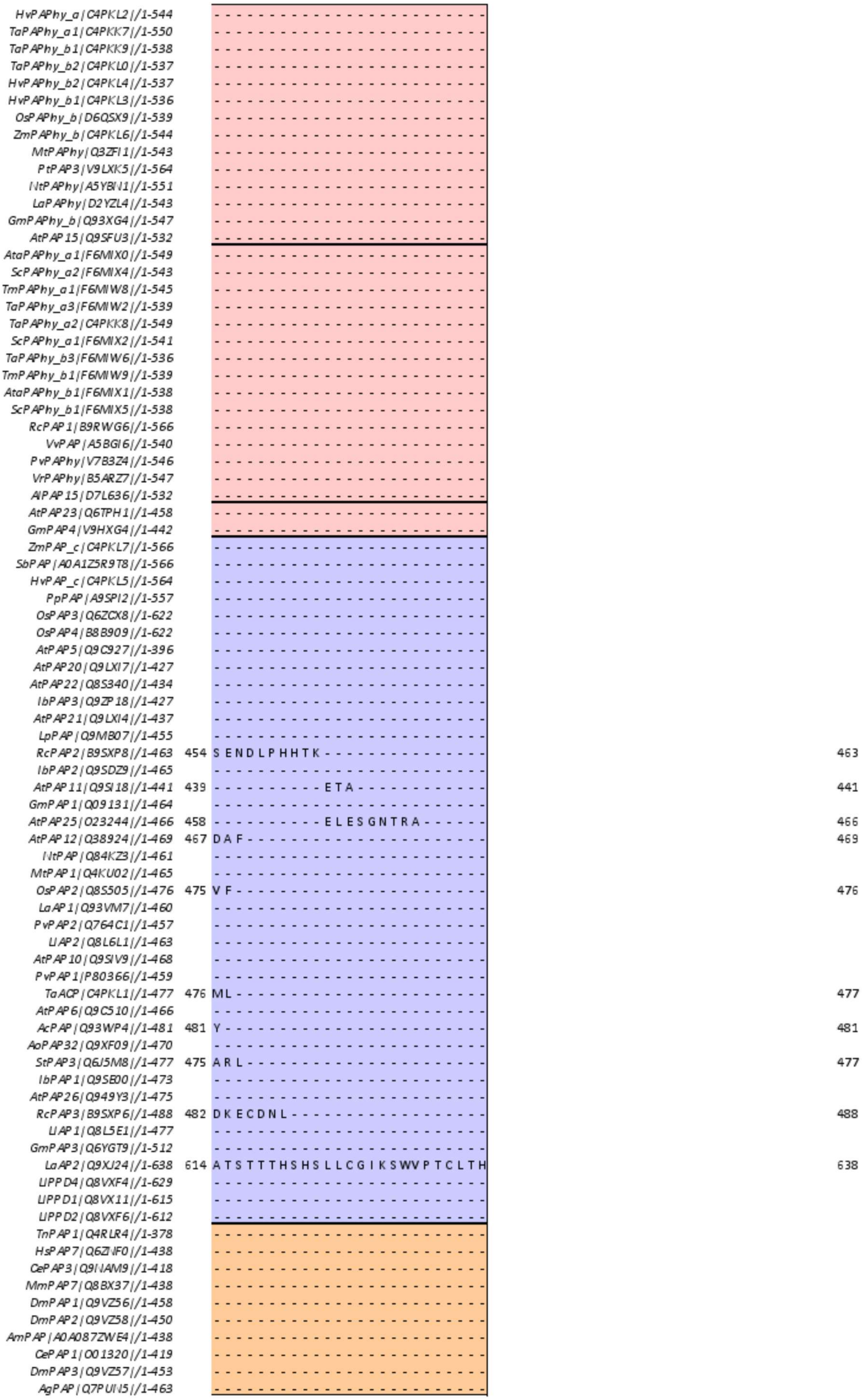
HvPAPhy_a|C4PKL2|/1-544
TaPAPhy_a1|C4PKK7|/1-550
TaPAPhy_b1|C4PKK9|/1-538
TaPAPhy_b2|C4PKL0|/1-537
HvPAPhy_b2|C4PKL4|/1-537
HvPAPhy_b1|C4PKL3|/1-536
OsPAPhy_b|D6QSX9|/1-539
ZmPAPhy_b|C4PKL6|/1-544
MtPAPhy|Q3ZFI1|/1-543
PtPAP3|V9LXK5|/1-564
NtPAPhy|A5YBN1|/1-551
LaPAPhy|D2YZL4|/1-543
GmPAPhy_b|Q93XG4|/1-547
AtPAP15|Q9SFU3|/1-532
AtaPAPhy_a1|F6MIX0|/1-549
ScPAPhy_a2|F6MIX4|/1-543
TmPAPhy_a1|F6MIW8|/1-545
TaPAPhy_a3|F6MIW2|/1-539
TaPAPhy_a2|C4PKK8|/1-549
ScPAPhy_a1|F6MIX2|/1-541
TaPAPhy_b3|F6MIW6|/1-536
TmPAPhy_b1|F6MIW9|/1-539
AtaPAPhy_b1|F6MIX1|/1-538
ScPAPhy_b1|F6MIX5|/1-538
RcPAP1|B9RWG6|/1-566
VvPAP|A5BGI6|/1-540
PvPAPhy|V7B3Z4|/1-546
VrPAPhy|B5ARZ7|/1-547
AlPAP15|D7L636|/1-532
AtPAP23|Q6TPH1|/1-458
GmPAP4|V9HXG4|/1-442
ZmPAP_c|C4PKL7|/1-566
SbPAP|A0A1Z5R9T8|/1-566
HvPAP_c|C4PKL5|/1-564
PpPAP|A9SPI2|/1-557
OsPAP3|Q6ZCX8|/1-622
OsPAP4|B8B909|/1-622
AtPAP5|Q9C927|/1-396
AtPAP20|Q9LXI7|/1-427
AtPAP22|Q8S340|/1-434
IbPAP3|Q9ZP18|/1-427
AtPAP21|Q9LXI4|/1-437
LpPAP|Q9MB07|/1-455
RcPAP2|B9SXP8|/1-463 454 SENDLPHHTK 463
IbPAP2|Q9SDZ9|/1-465
AtPAP11|Q9SI18|/1-441 439 ETA 441
GmPAP1|Q09131|/1-464
AtPAP25|O23244|/1-466 458 ELESGNTRA 466
AtPAP12|Q38924|/1-469 467 DAF 469
NtPAP|Q84KZ3|/1-461
MtPAP1|Q4KU02|/1-465
OsPAP2|Q8S505|/1-476 475 VF 476
LaAP1|Q93VM7|/1-460
PvPAP2|Q764C1|/1-457
LlAP2|Q8L6L1|/1-463
AtPAP10|Q9SIV9|/1-468
PvPAP1|P80366|/1-459
TaACP|C4PKL1|/1-477 476 ML 477
AtPAP6|Q9C510|/1-466
AcPAP|Q93WP4|/1-481 481 Y 481
AoPAP32|Q9XF09|/1-470
StPAP3|Q6J5M8|/1-477 475 ARL 477
IbPAP1|Q9SE00|/1-473
AtPAP26|Q949Y3|/1-475
RcPAP3|B9SXP6|/1-488 482 DKECDNL 488
LlAP1|Q8L5E1|/1-477
GmPAP3|Q6YGT9|/1-512
LaAP2|Q9XJ24|/1-638 614 ATSTTTHSHSLLCGIKSWVPTCLTH 638
LlPPD4|Q8VXF4|/1-629
LlPPD1|Q8VX11|/1-615
LlPPD2|Q8VXF6|/1-612
TnPAP1|Q4RLR4|/1-378
HsPAP7|Q6ZNF0|/1-438
CePAP3|Q9NAM9|/1-418
MmPAP7|Q8BX37|/1-438
DmPAP1|Q9VZ56|/1-458
DmPAP2|Q9VZ58|/1-450
AmPAP|A0A087ZWE4|/1-438
CePAP1|O01320|/1-419
DmPAP3|Q9VZ57|/1-453
AgPAP|Q7PUN5|/1-463

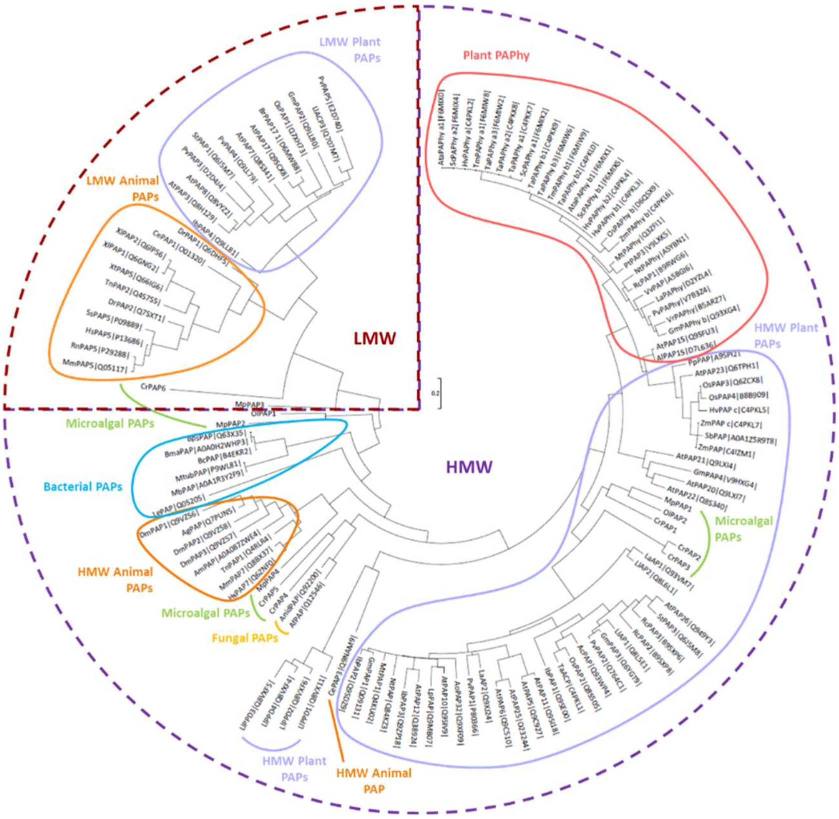

Supplemental Figure 4. Molecular phylogenetic analysis of PAP sequences by the maximum likelihood method. The evolutionary history was inferred by using the Maximum Likelihood method based on the JTT matrix-based model(Jones *et al.*, 1992). The tree with the highest log likelihood (-5950.08) is shown. Initial tree(s) for the heuristic search were obtained automatically by applying Neighbour-Join and BioNJ algorithms to a matrix of pairwise distances estimated using a JTT model, and then selecting the topology with superior log likelihood value. The tree is drawn to scale, with branch lengths measured in the number of substitutions per site. The analysis involved 126 amino acid sequences. All positions containing gaps and missing data were eliminated. There was a total of 59 positions in the final dataset. Evolutionary analyses were conducted in MEGA7 (Kumar *et al.*, 2016).

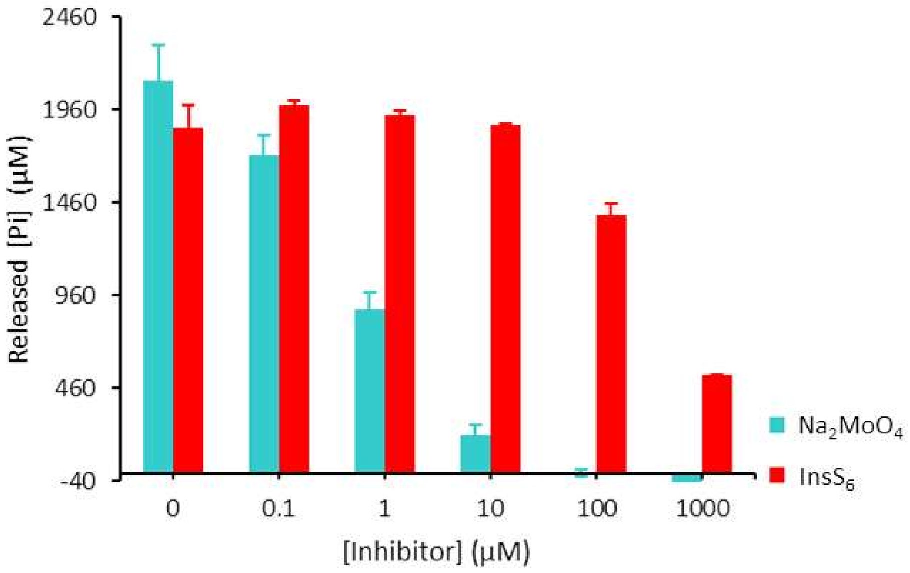

Supplemental Figure 5. Inhibition of phytase activity by *myo*-inositol hexakissulfate ($InsS_6$). Inhibition by sodium molybdate, a known competitive plant PAP inhibitor, is included for reference. Error bars show standard deviations of triplicate measurements.

A

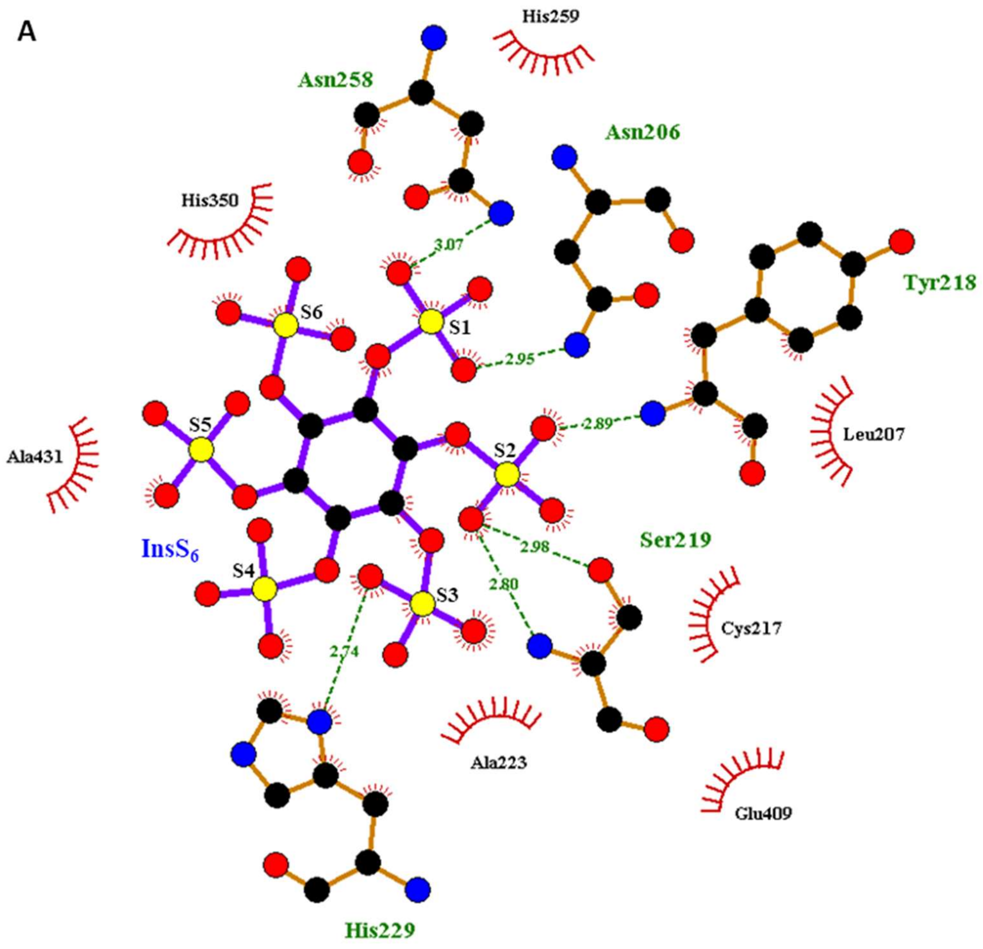

Supplemental Figure 6. Intermolecular interactions in the crystal structure of the TaPAPhy_b2:InsS$_6$ complex. Rendering of interactions in the crystal structure of the complex (PDB entry 6GJ2). Produced using Ligplot+ (Laskowski and Swindells, 2011).

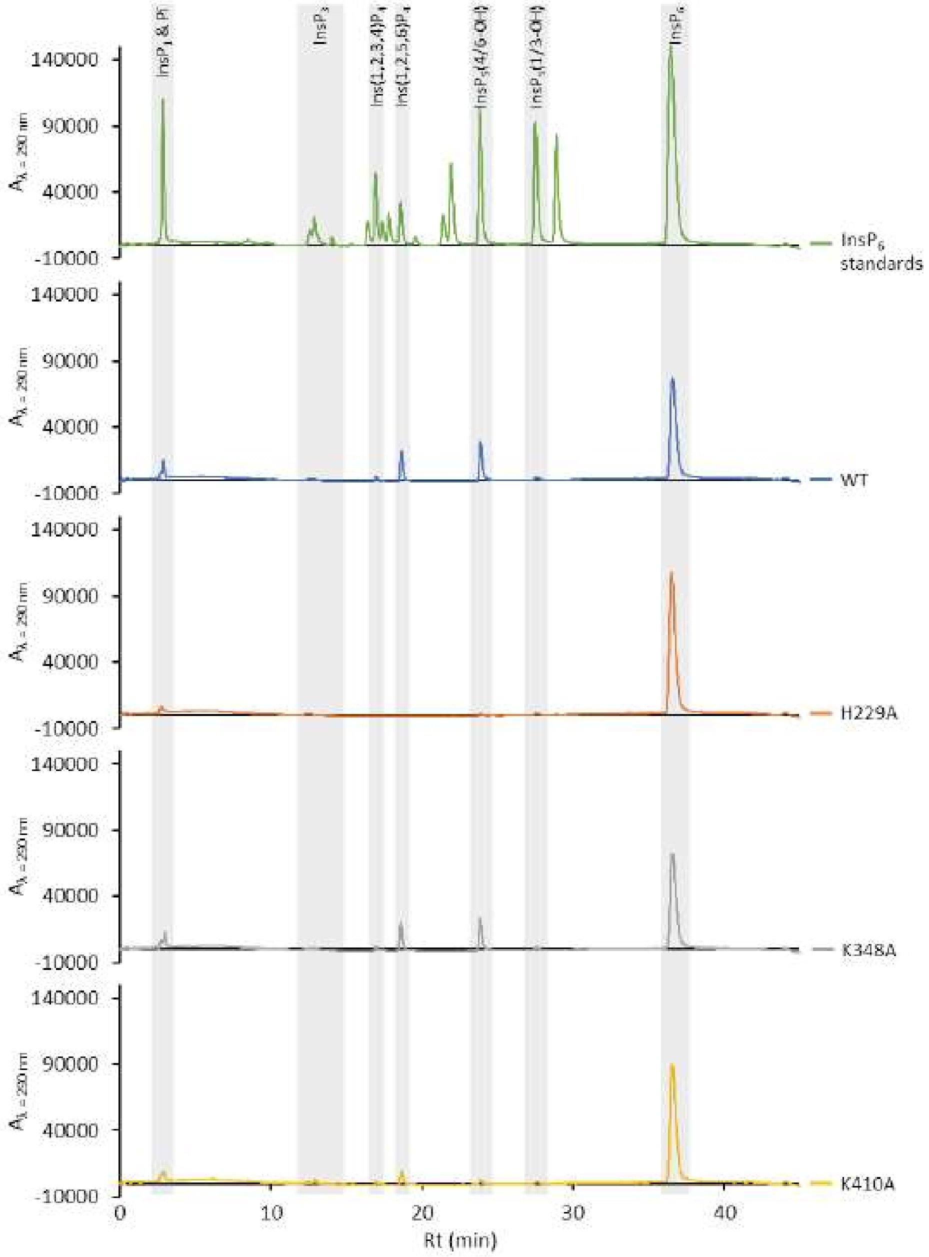

Supplemental Figure 7. HPLC product profiles of recombinant wild type (WT) TaPAPhy_b2 and its active mutants after limited reaction against $InsP_6$. Reactions were performed for 15 min at room temperature with 1 mM $InsP_6$ substrate and 1 µM enzymes in 0.2 M acetate buffer pH 5.5. An acid hydrolysate of $InsP_6$ with relevant peaks labelled for reference is shown ($InsP_5$s are identified by the residual hydroxyl). 'Rt', retention time.

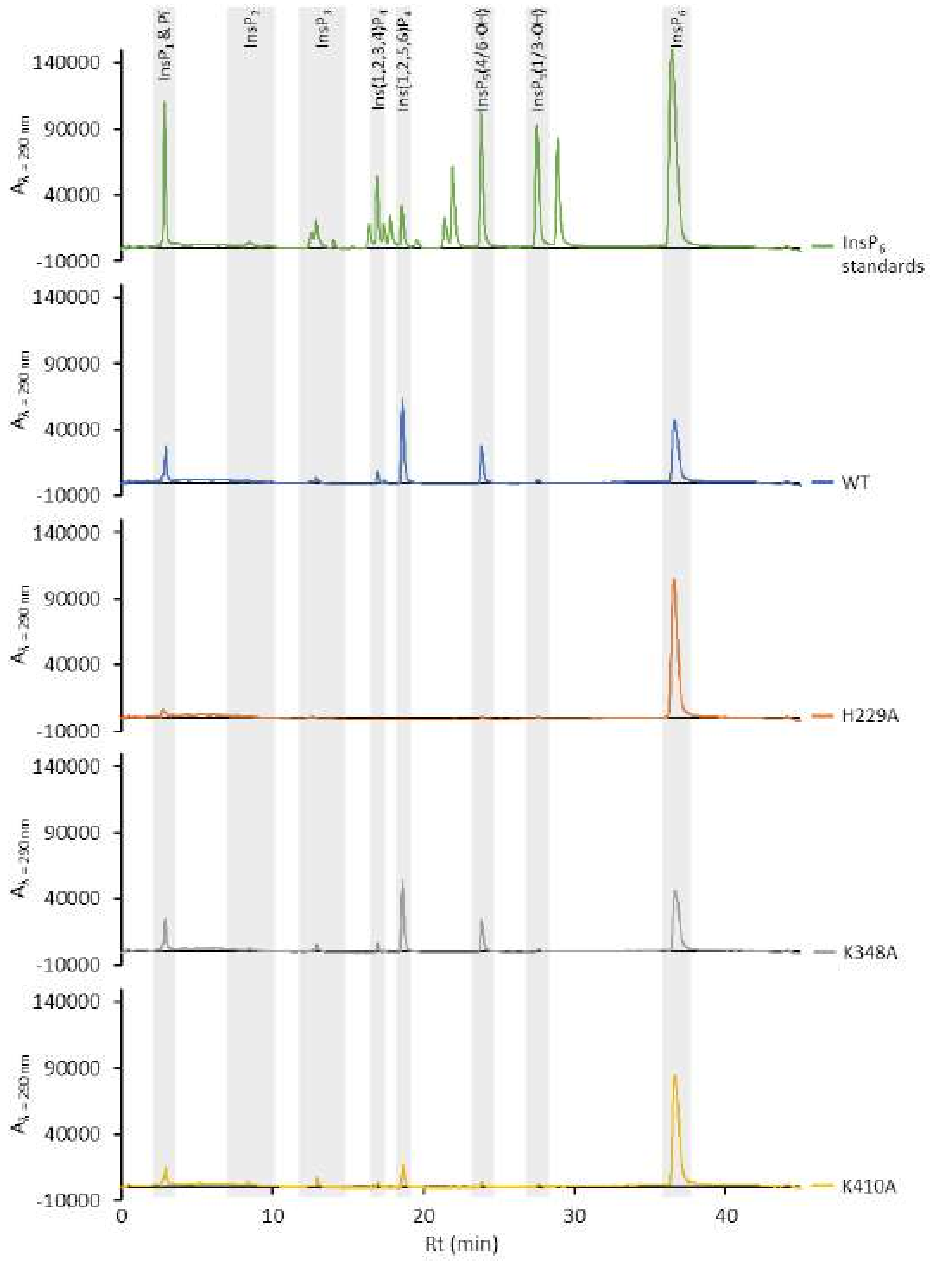

Supplemental Figure 8. HPLC product profiles of recombinant wild type (WT) TaPAPhy_b2 and its active site mutants after progressive reaction against $InsP_6$. Reactions were performed for 30 min at room temperature with 1 mM $InsP_6$ substrate and 1 µM enzymes in 0.2 M acetate buffer pH 5.5. An acid hydrolysate of $InsP_6$ with relevant peaks labelled for reference is shown ($InsP_5$s are identified by the residual hydroxyl). 'Rt', retention time.

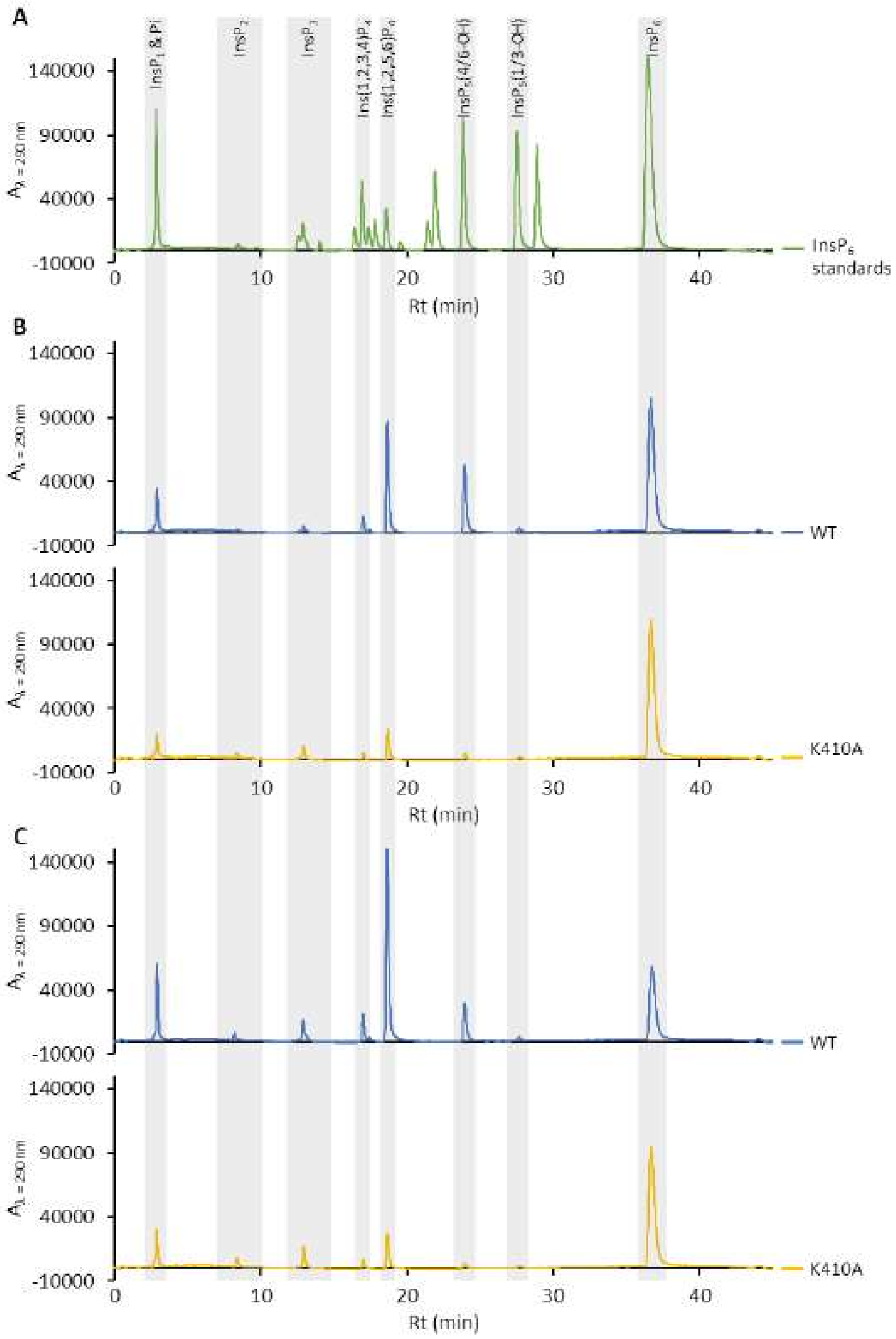

Supplemental Figure 9. HPLC product profiles of recombinant wild type (WT) TaPAPhy_b2 and its K410A mutant after extended reaction against $InsP_6$. Reactions were performed at room temperature with 1 mM $InsP_6$ substrate and 1 µM enzymes in 0.2 M acetate buffer pH 5.5. 'Rt', retention time. (**A**) An acid hydrolysate of $InsP_6$ with relevant peaks labelled for reference is shown ($InsP_5$s are identified by the residual hydroxyl). (**B**) 1 h reaction. (**C**) 2 h reaction.

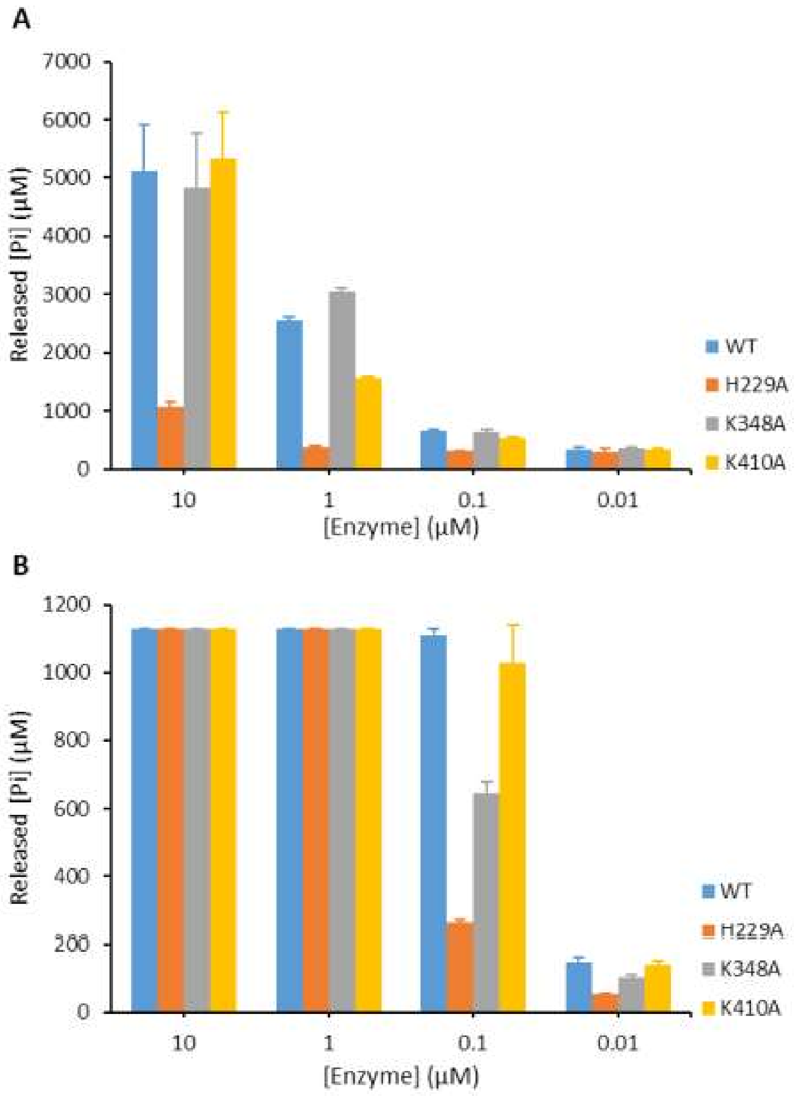

Supplemental Figure 10. Phytase and *p*-nitrophenyl phosphatase activity of WT TaPAPhy_b2 and its mutants. Phosphate release assay with 5 mM $InsP_6$ as substrate in 0.2 M acetate buffer pH 5.5 for 15 min at room temperature. The average phosphate concentration released as a measure of phytase activity of four replicate reactions with decreasing enzyme concentrations is displayed. Error bars represent the standard deviation of the four replicates. (B) Phosphate release assay with 5 mM *p*-nitrophenyl phosphatase (pNPP) as substrate in 0.2M acetate buffer pH 5.5 for 15 min at room temperature. The average phosphate concentration released as a measure of phosphatase activity of four replicate reactions with decreasing enzyme concentrations is displayed. Error bars represent the standard deviation of the four replicates. pNPP background absorbance was subtracted from the measurements. 'Pi', inorganic phosphate.

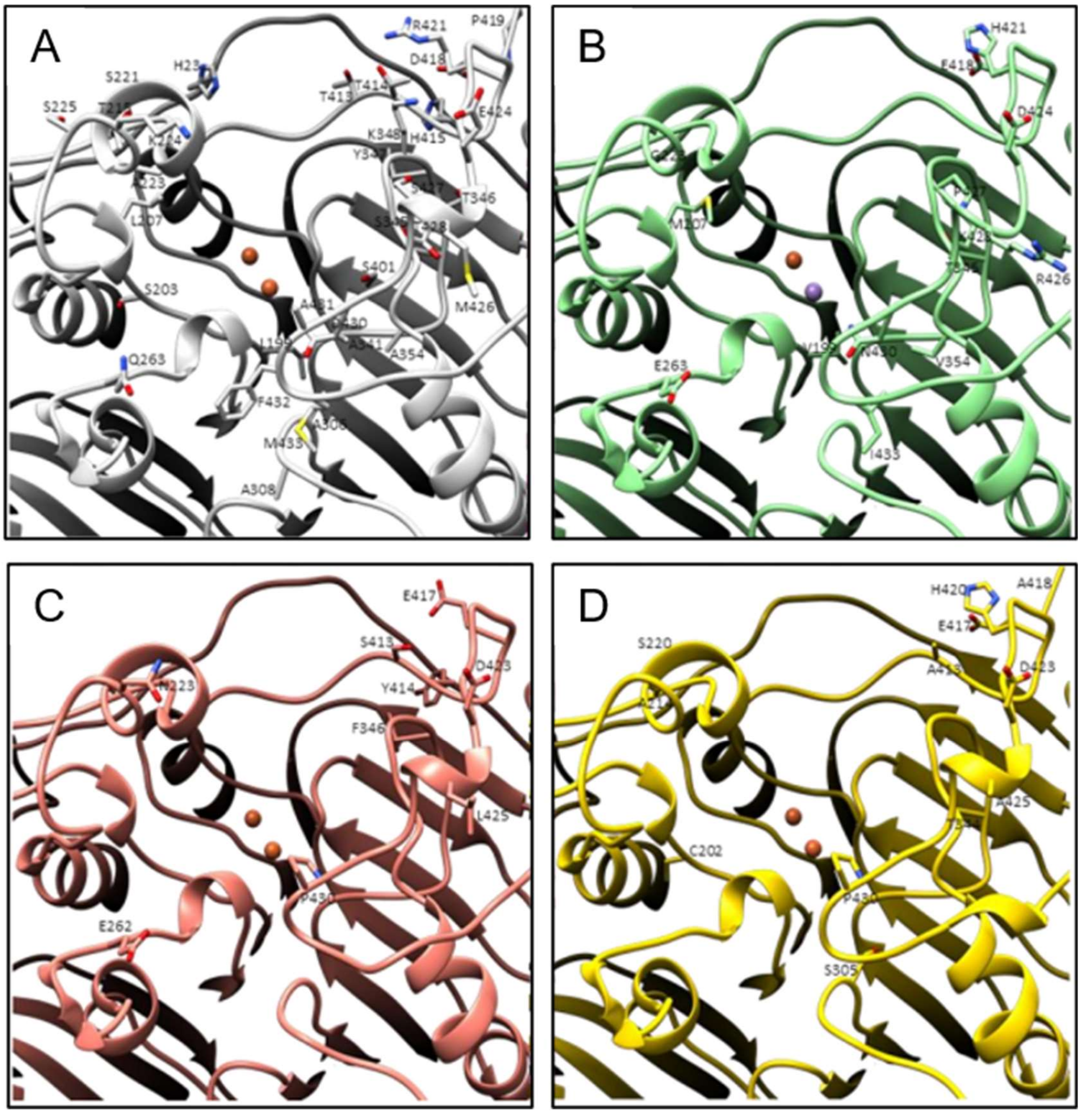

Supplemental Figure 11. Comparison of the active sites of cereal PAPhys. The active sites of the TaPAPhy_b2 crystal structure and plant PAPhy homology models are displayed in cartoon representation with metal ions shown as spheres and coloured by element (i.e. Fe, brown; Mn, lilac). Residues that are not conserved in one or more of the enzymes analysed with respect to TaPAPhy_b2 are shown as sticks, coloured by element (oxygen-red; nitrogen-blue) and labelled. Images created with the UCSF Chimera package (Pettersen *et al.*, 2004). (A) TaPAPhy_b2; (B) HvPAPhy_a; (C) OsPAPhy_b; (D) ZmPAPhy_b.

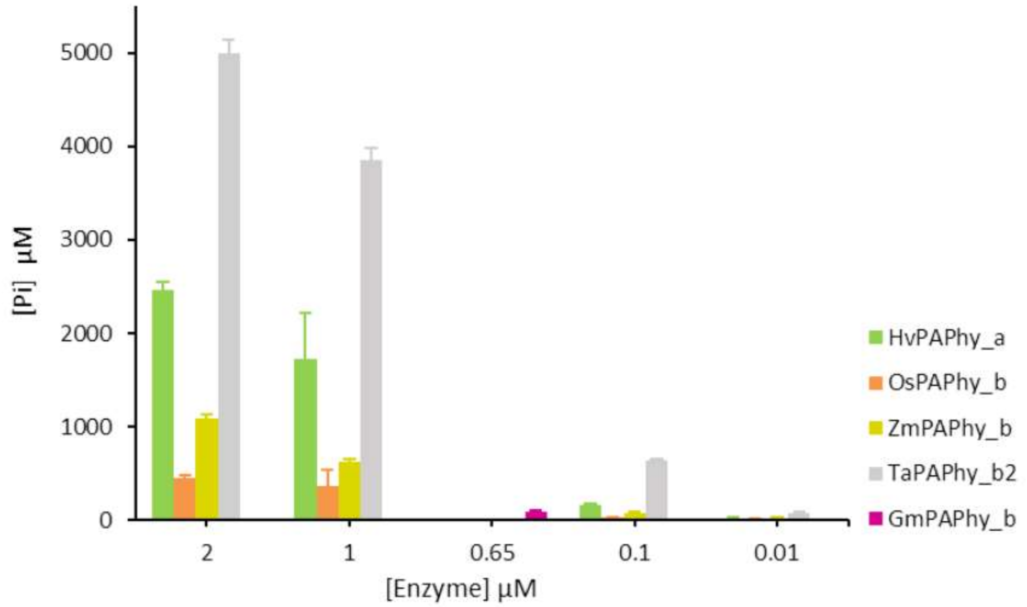

Supplemental Figure 12. Phytase activities of recombinant cereal PAPhy enzymes. Phosphate release assay with 5 mM InsP6 as substrate in 0.2 M acetate buffer pH 5.5 for 15 min at room temperature. The average phosphate concentration released as a measure of phytase activity of four replicate reactions with decreasing enzyme concentrations is displayed. Error bars represent the standard deviation of the four replicates. A single unique concentration with two replicate reactions was assayed for GmPAPhy_b. $InsP_6$ background absorbance was subtracted from the measurements. ‘P_i’, inorganic phosphate.

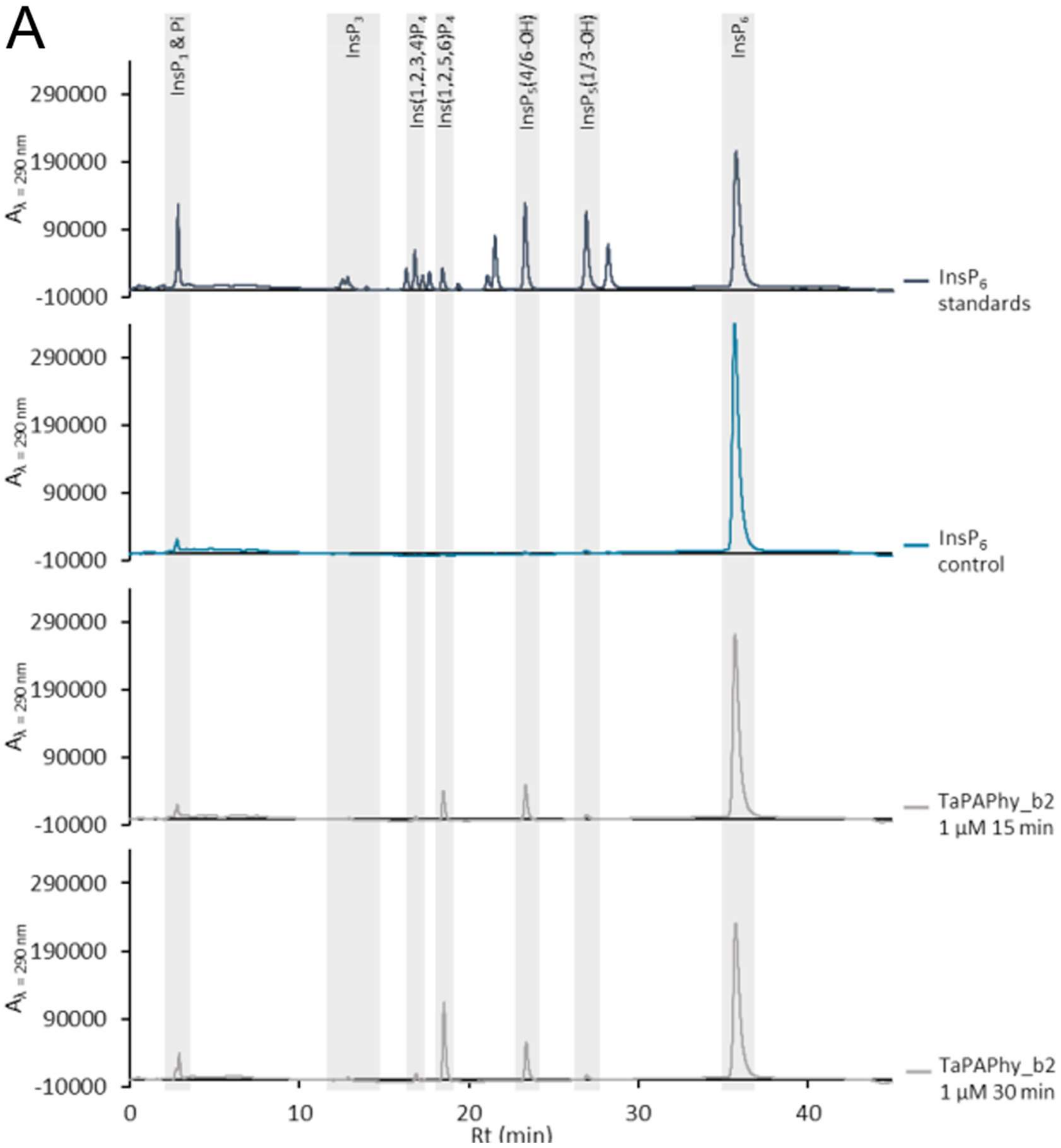

Supplemental Figure 13. Inositol polyphosphate product profiles following hydrolysis of $InsP_6$ by recombinant wheat PAPhy isoform b2 (TaPAPhy_b2). Reactions were performed for varying times (15, 30 mins) at room temperature with 1 mM $InsP_6$ substrate and 1 µM enzyme concentration in 0.2 M acetate buffer pH 5.5. A control reaction in the absence of enzyme was included. An acid hydrolysate of $InsP_6$ with relevant peaks labelled for reference is shown ($InsP_5$ are identified by the residual hydroxyl group). 'Rt', retention time.

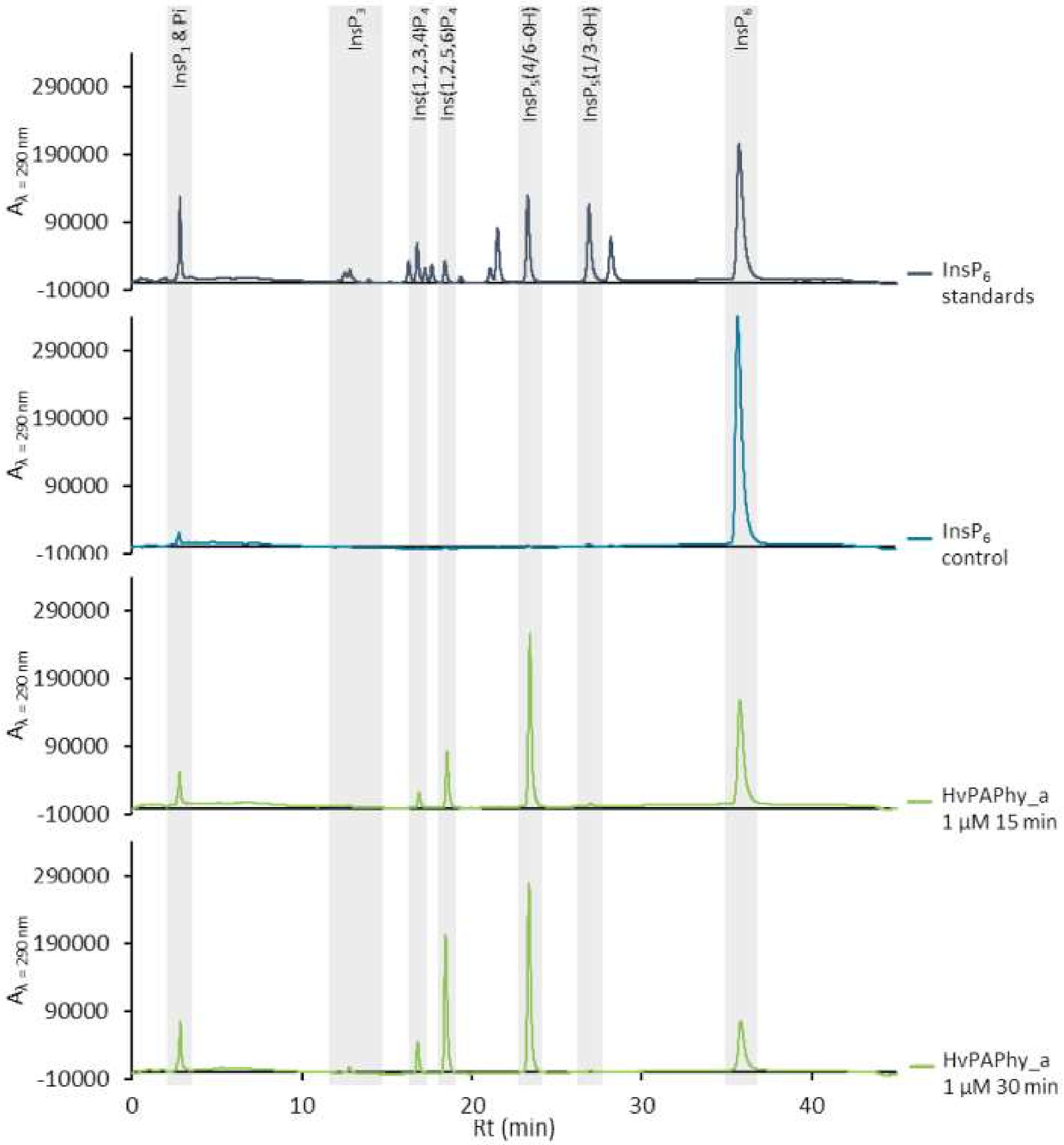

Supplemental Figure 14. Inositol polyphosphate product profiles following hydrolysis of InsP$_6$ by recombinant barley PAPhy isoform a (HvPAPhy_a). Reactions were performed for varying times (15, 30 mins) at room temperature with 1 mM InsP$_6$ substrate and 1 µM enzyme concentration in 0.2 M acetate buffer pH 5.5. A control reaction in the absence of enzyme was included. An acid hydrolysate of InsP$_6$ with relevant peaks labelled for reference is shown (InsP$_5$ are identified by the residual hydroxyl group). 'Rt', retention time.

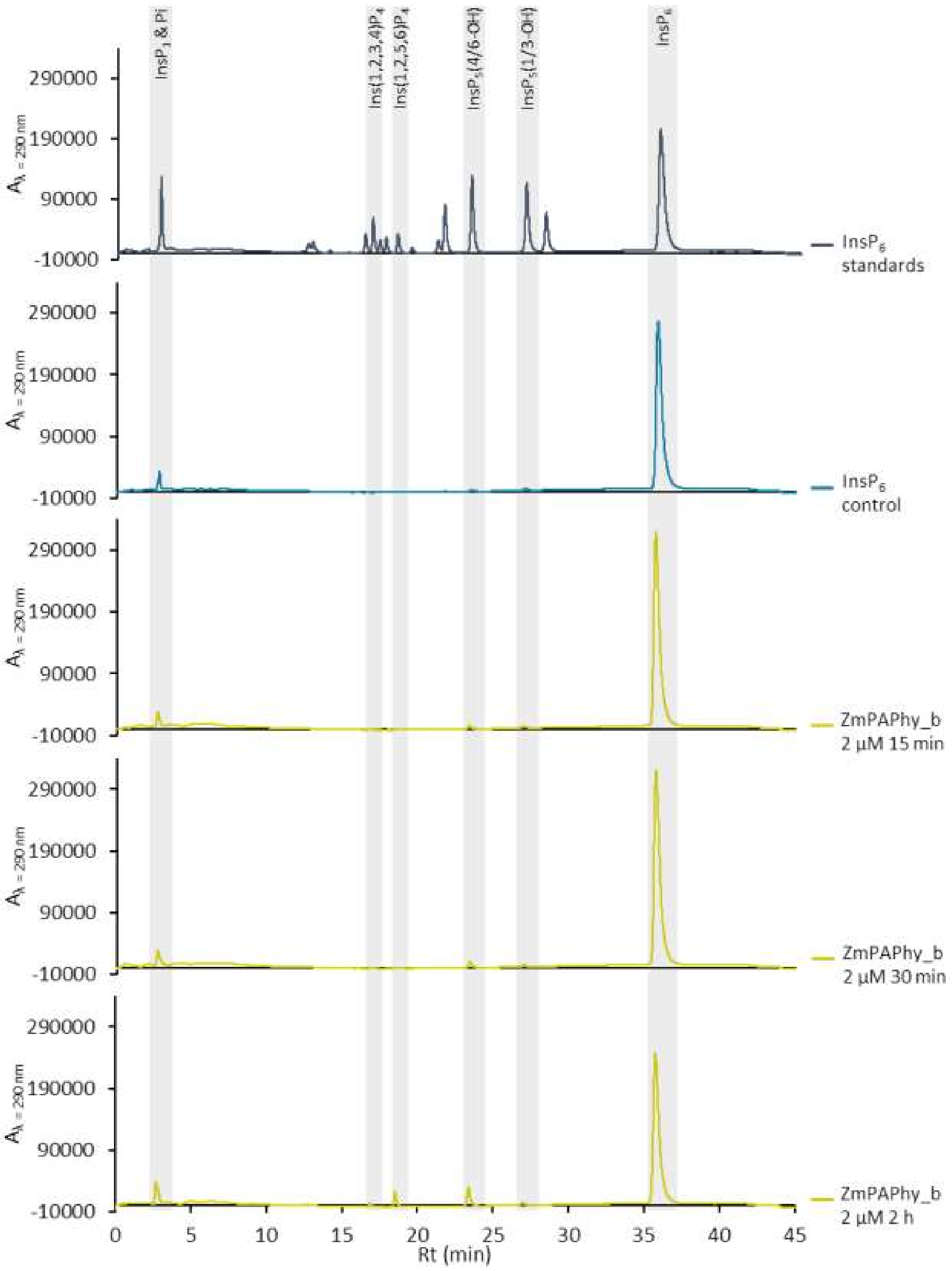

Supplemental Figure 15. Inositol polyphosphate product profiles following hydrolysis of $InsP_6$ by recombinant maize PAPhy isoform b (ZmPAPhy_b). Reactions were performed for varying times (15, 30, 120 mins) at room temperature with 1 mM $InsP_6$ substrate and 2 µM enzyme concentration in 0.2 M acetate buffer pH

5.5. A control reaction in the absence of enzyme was included. An acid hydrolysate of $InsP_6$ with relevant peaks labelled for reference is shown ($InsP_5$ are identified by the residual hydroxyl group). 'Rt', retention time.

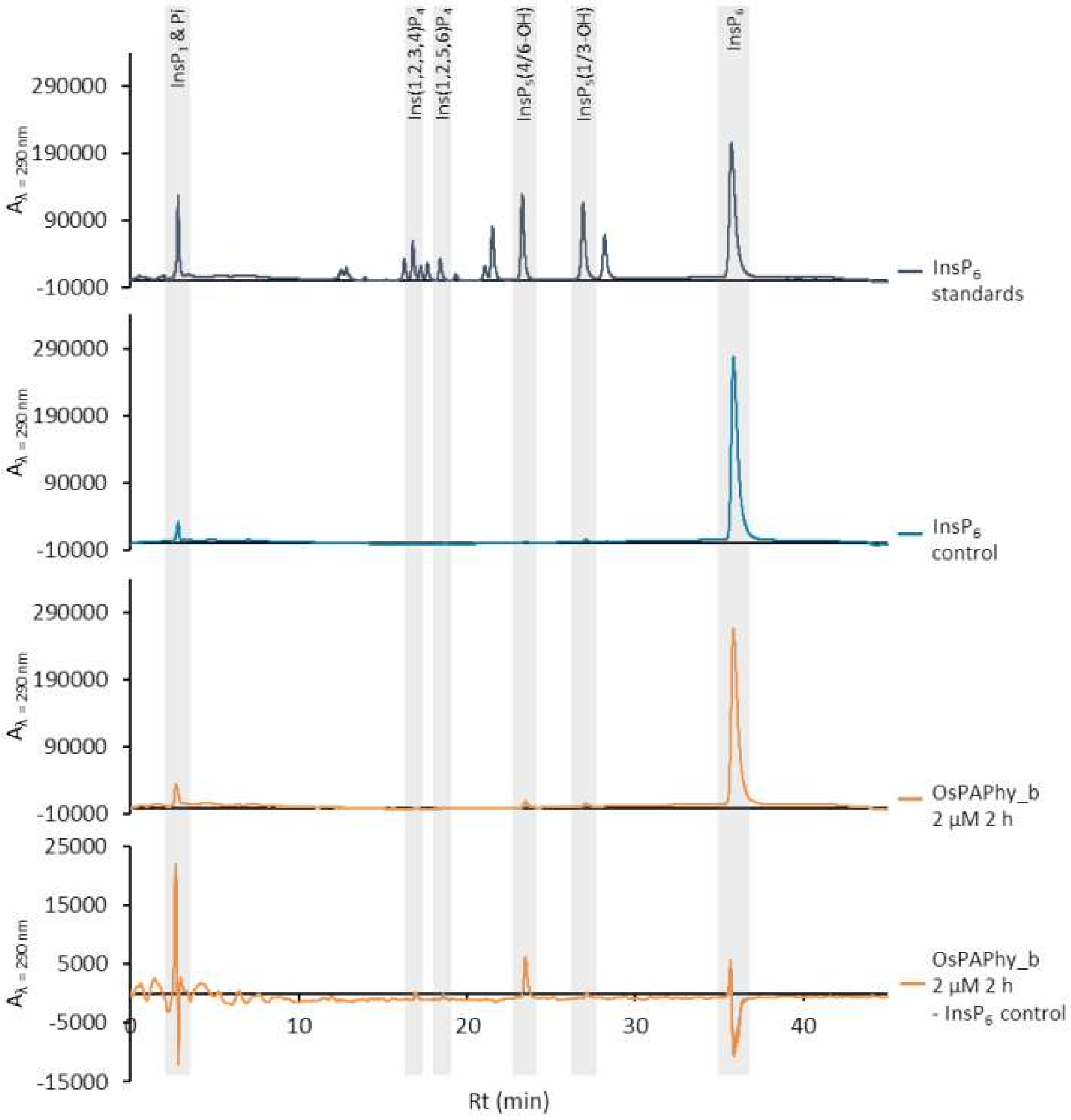

Supplemental Figure 16. Inositol polyphosphate product profiles following hydrolysis of $InsP_6$ by recombinant rice PAPhy isoform b (OsPAPhy_b). Reactions were performed for 120 mins at room temperature with 1 mM $InsP_6$ substrate and 2 µM enzyme concentration in 0.2 M acetate buffer pH 5.5. A control reaction in the absence of enzyme was included. An acid hydrolysate of $InsP_6$ with relevant peaks labelled for reference is shown ($InsP_5$ are identified by the residual

hydroxyl group). ‘Rt’, retention time. Note that for OsPAPhy_b the lower trace shows the profile after subtraction of the substrate-only profile.

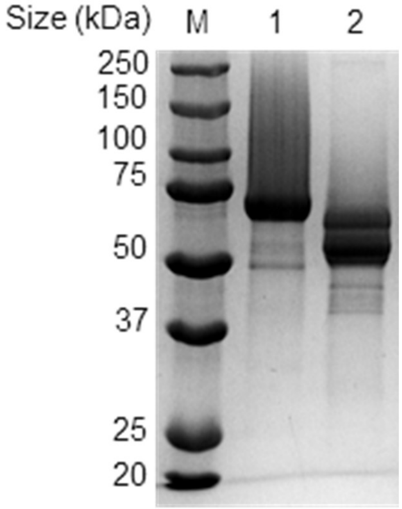

Supplemental Figure 17. Partial enzymatic deglycosylation of TaPAPhy_b2 using recombinant GST-Endo F1. SDS-PAGE (10% v/v acrylamide) gel showing: Lane M, dual colour protein standards (Biorad); Lane 1, glycoengineered TaPAPhy_b2 untreated control; lane 2, TaPAPhy_b2 with GST-Endo F1 (58.7 kDa band).

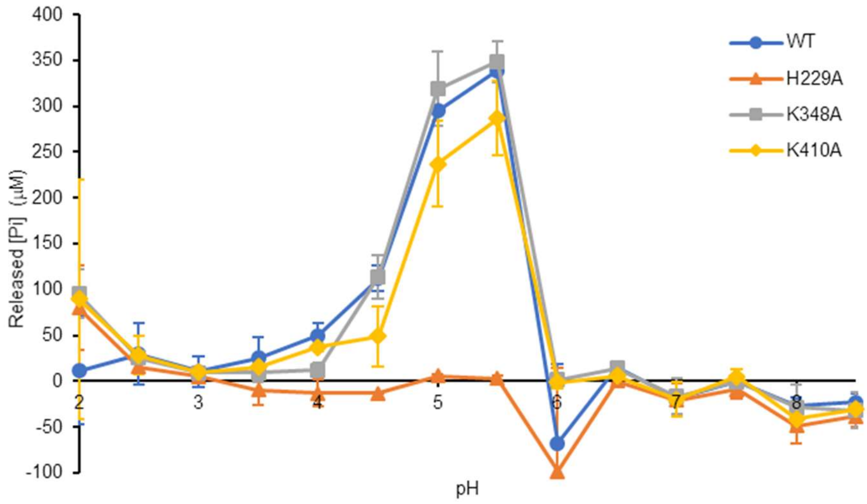

Supplemental Figure 18. pH profile of phytase activities of recombinant wild type (WT) TaPAPhy_b2 and active site mutants.

a

ID	Res.	Metal	*Occupancy*	***B* factor (env.)**[1]	*Ligands*	*Valence*[2]	*nVECSUM*[3]	*Geometry*[1,4]	*gRMSD(°)*[1]	*Vacancy*[1]	Bidentate	Alt. metal
B:1	FE	Fe	0.47	44.8 (35.3)	O_4	**4.2**	**0.32**	Tetrahedral	16.4°	0	0	Cu, Fe
B:2	FE	Fe	0.89	12.8 (18.2)	O_3N_2	2	0.11	Octahedral	9.8°	16%	0	Co, Cu, Zn

Legend: Not applicable | **Outlier** | *Borderline* | Acceptable

b

ID	Res.	Metal	*Occupancy*	***B* factor (env.)**[1]	*Ligands*	*Valence*[2]	*nVECSUM*[3]	*Geometry*[1,4]	*gRMSD(°)*[1]	*Vacancy*[1]	Bidentate	Alt. metal
B:1	FE	Fe	0.62	20.1 (24.8)	O_5N_1	2.4	0.11	Octahedral	15.7°	0	0	Co, Cu, Zn
B:2	FE	Fe	0.9	16.7 (23.1)	O_4N_2	1.8	0.058	Octahedral	14.6°	0	0	Co, Cu

c **d**

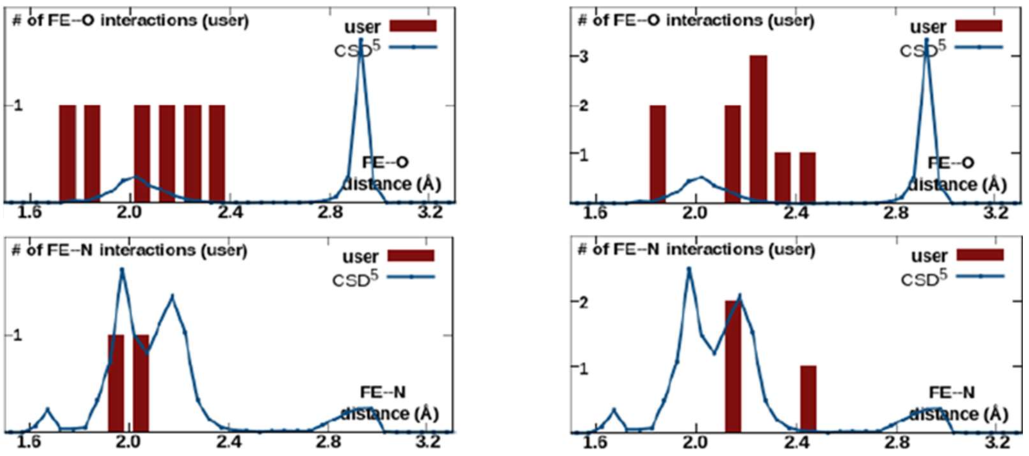

Supplemental Figure 19. Validation of metal-binding sites of TaPAPhy_b2:PO4 structures. Analysis performed using CheckMyMetal (Zheng *et al.*, 2014). Evaluation of the metal-binding sites in **a**, product-bound and **b**, substrate-bound structures. Metal-ligand distance distributions for **c**, the product-bound and **d**, the substrate-bound structures in comparison with data from the Cambridge Structural Database (CSD).

SUPPLEMENTAL REFERENCES

Adams, P. D. *et al.* (2010) 'PHENIX: A comprehensive Python-based system for macromolecular structure solution', *Acta Crystallographica Section D: Biological Crystallography*, 66(2), pp. 213–221. doi: 10.1107/S0907444909052925.

Altschul, S. F. and Gish, W. (1996) 'Local alignment statistics', *Methods in enzymology*, 266, pp. 460–480. doi: 10.1016/S0076-6879(96)66029-7.

Baker, N. A. *et al.* (2001) 'Electrostatics of nanosystems: Application to microtubules and the ribosome', 98, pp. 10037–10041. Available at: www.pnas.orgcgidoi10.1073pnas.181342398 (Accessed: 28 July 2021).

Bateman, A. *et al.* (2017) 'UniProt: The universal protein knowledgebase', *Nucleic Acids Research*. Oxford University Press, 45(D1), pp. D158–D169. doi: 10.1093/nar/gkw1099.

Bretthauer, R. K. and Castellino, F. J. (1999) 'Glycosylation of Pichia pastoris-derived proteins.', *Biotechnology and applied biochemistry*, 30(3), pp. 193–200. doi: 10.1111/j.1470-8744.1999.tb00770.x.

Bunkóczi, G. and Read, R. J. (2011) 'Improvement of molecular-replacement models with Sculptor', *Acta Crystallographica Section D: Biological Crystallography*. International Union of Crystallography, 67(4), pp. 303–312. doi: 10.1107/S0907444910051218.

Daura, X. *et al.* (1999) 'Peptide folding: When simulation meets experiment', *Angewandte Chemie - International Edition*, 38(1/2), pp. 236–240. doi: 10.1002/(sici)1521-3773(19990115)38:1/2<236::aid-anie236>3.0.co;2-m.

Dionisio, G. *et al.* (2011) 'Cloning and characterization of purple acid phosphatase phytases from wheat, barley, maize, and rice', *Plant Physiology*. American Society of Plant Biologists, 156(3), pp. 1087–1100. doi: 10.1104/pp.110.164756.

Dionisio, G. *et al.* (2012) 'Glycosylations and truncations of functional cereal phytases expressed and secreted by Pichia pastoris documented by mass spectrometry.', *Protein expression and purification*. Protein Expr Purif, 82(1), pp. 179–85. doi: 10.1016/j.pep.2011.12.003.

Edgar, R. C. (2004) 'MUSCLE: Multiple sequence alignment with high accuracy and high throughput', *Nucleic Acids Research*. Oxford University Press, 32(5), pp. 1792–1797. doi: 10.1093/nar/gkh340.

Emsley, P. *et al.* (2010) 'Features and development of Coot', *Acta Crystallographica Section D: Biological Crystallography*. International Union of Crystallography, 66(4), pp. 486–501. doi: 10.1107/S0907444910007493.

Fiser, A. and Sali, A. (2003) 'ModLoop: automated modeling of loops in protein structures', *BIOINFORMATICS APPLICATIONS NOTE*, 19(18), pp. 2500–2501. doi: 10.1093/bioinformatics/btg362.

Goodstein, D. M. *et al.* (2012) 'Phytozome: a comparative platform for green plant genomics', *Nucleic Acids Research*. Oxford University Press, 40(D1), pp. D1178-86. doi: 10.1093/nar/gkr944.

Gordon, J. C. *et al.* (2005) 'H++: A server for estimating pKas and adding missing hydrogens to macromolecules', *Nucleic Acids Research*, 33(SUPPL. 2), pp. 368–371. doi: 10.1093/nar/gki464.

Greiner, R., Jany, K. D. and Larsson Alminger, M. (2000) 'Identification and Properties of myo -Inositol Hexakisphosphate Phosphohydrolases (Phytases) from Barley (Hordeum vulgare)', *Journal of Cereal Science*. Academic Press, 31(2), pp. 127–139. doi: 10.1006/JCRS.1999.0254.

Greiner, R., Konietzny, U. and Jany, K. D. (1998) 'Purification and properties of a phytase from rye', *Journal of Food Biochemistry*. Blackwell Publishing Ltd, 22(2), pp. 143–161. doi: 10.1111/J.1745-4514.1998.TB00236.X.

Grueninger-Leitch, F. *et al.* (1996) ‘Deglycosylation of proteins for crystallization using recombinant fusion protein glycosidases’, *Protein Science*, 5(12), pp. 2617–2622. doi: 10.1002/pro.5560051224.

Hayakawa, T., Toma, Y. and Igaue, I. ‘Purification and Characterization of Acid Phosphatases with or without Phytase Activity from Rice Bran’, *Agr. Biol. Chem.*, 53(6), pp. 1475–1483. doi: 10.1080/00021369.1989.10869506.

Hegeman, C. E. and Grabau, E. A. (2001) ‘A novel phytase with sequence similarity to purple acid phosphatases is expressed in cotyledons of germinating soybean seedlings’, *Plant Physiology*. Plant Physiol, 126(4), pp. 1598–1608. doi: 10.1104/pp.126.4.1598.

Hess, B. *et al.* (2008) ‘GROMACS 4: Algorithms for highly efficient, load-balanced, and scalable molecular simulation’, *Journal of Chemical Theory and Computation*, 4(3), pp. 435–447. doi: 10.1021/ct700301q.

Jacobs, P. P. *et al.* (2009) ‘Engineering complex-type N-glycosylation in Pichia pastoris using GlycoSwitch technology’, *Nature Protocols*, 4(1), pp. 58–70. doi: 10.1038/nprot.2008.213.

Jones, D. T. *et al.* (1992) ‘The rapid generation of mutation data matrices from protein sequences’, *CABIOS*, 8(3), pp. 275–282. Available at: https://pubmed.ncbi.nlm.nih.gov/1633570/ (Accessed: 1 August 2021).

Kong, Y. *et al.* (2014) ‘GmPAP4, a novel purple acid phosphatase gene isolated from soybean (Glycine max), enhanced extracellular phytate utilization in Arabidopsis thaliana’, *Plant Cell Reports*. Springer Verlag, 33(4), pp. 655–667. doi: 10.1007/s00299-014-1588-5.

Koziara, K. B. *et al.* (2014) ‘Testing and validation of the Automated Topology Builder (ATB) version 2.0: Prediction of hydration free enthalpies’, *Journal of Computer-Aided Molecular Design*, 28(3), pp. 221–233. doi: 10.1007/s10822-014-9713-7.

Kuang, R. *et al.* (2009) 'Molecular and biochemical characterization of AtPAP15, a purple acid phosphatase with phytase activity, in Arabidopsis', *Plant Physiology*. American Society of Plant Biologists, 151(1), pp. 199–209. doi: 10.1104/pp.109.143180.

Kumar, S. *et al.* (2016) 'MEGA7: Molecular Evolutionary Genetics Analysis Version 7.0 for Bigger Datasets', *Mol. Biol. Evol*, 33(7), pp. 1870–1874. doi: 10.1093/molbev/msw054.

Laskowski, R. A. and Swindells, M. B. (2011) 'LigPlot+: Multiple ligand-protein interaction diagrams for drug discovery', *Journal of Chemical Information and Modeling*. J Chem Inf Model, 51(10), pp. 2778–2786. doi: 10.1021/ci200227u.

Lazali, M. *et al.* (2013) 'A phytase gene is overexpressed in root nodules cortex of Phaseolus vulgaris-rhizobia symbiosis under phosphorus deficiency', *Planta*. Planta, 238(2), pp. 317–324. doi: 10.1007/s00425-013-1893-1.

Lazali, M. *et al.* (2014) 'Localization of phytase transcripts in germinating seeds of the common bean (Phaseolus vulgaris L.)', *Planta*. Springer Verlag, 240(3), pp. 471–478. doi: 10.1007/s00425-014-2101-7.

Lin-Cereghino, J. *et al.* (2005) 'Condensed protocol for competent cell preparation and transformation of the methylotrophic yeast Pichia pastoris', *Biotechniques*, 38(1), pp. 44–48.

Lin-Cereghino, J. *et al.* (2008) 'Direct selection of Pichia pastoris expression strains using new G418 resistance vectors', *Yeast*, 25(4), pp. 293–299.

Liu, H. and Naismith, J. H. (2008) 'An efficient one-step site-directed deletion, insertion, single and multiple-site plasmid mutagenesis protocol', *BMC biotechnology*. BMC Biotechnol, 8, p. 91. doi: 10.1186/1472-6750-8-91.

Lung, S. C. *et al.* (2008) 'Phytase activity in tobacco (Nicotiana tabacum) root exudates is exhibited by a purple acid phosphatase', *Phytochemistry*.

Phytochemistry, 69(2), pp. 365–373. doi: 10.1016/j.phytochem.2007.06.036.

Madsen, C. K. *et al.* (2013) ‘High mature grain phytase activity in the Triticeae has evolved by duplication followed by neofunctionalization of the purple acid phosphatase phytase (PAPhy) gene’, *Journal of Experimental Botany*. Oxford Academic, 64(11), pp. 3111–3123. doi: 10.1093/jxb/ert116.

Maruyama, H. *et al.* (2012) ‘Effect of exogenous phosphatase and phytase activities on organic phosphate mobilization in soils with different phosphate adsorption capacities’, *J. Soil Sci. Plant Nutr.* Taylor & Francis Group, 58(1), pp. 41–51. doi: 10.1080/00380768.2012.656298.

McCoy, A. J. *et al.* (2007) ‘Phaser crystallographic software’, *Journal of Applied Crystallography*, 40(4), pp. 658–674. doi: 10.1107/S0021889807021206.

Nagul, E. A. *et al.* (2015) ‘The molybdenum blue reaction for the determination of orthophosphate revisited: Opening the black box’, *Analytica Chimica Acta*. Elsevier B.V., pp. 60–82. doi: 10.1016/j.aca.2015.07.030.

Nakano, T. *et al.* (1999) ‘Purification and Characterization of Phytase from Bran of Triticum aestivum L.cv. Nourin #61.’, *Food Science and Technology Research*. S. Karger AG, 5(1), pp. 18–23. doi: 10.3136/fstr.5.18.

Oostenbrink, C. *et al.* (2004) ‘A biomolecular force field based on the free enthalpy of hydration and solvation: The GROMOS force-field parameter sets 53A5 and 53A6’, *Journal of Computational Chemistry*, 25(13), pp. 1656–1676. doi: 10.1002/jcc.20090.

Pettersen, E. F. *et al.* (2004) ‘UCSF Chimera - A visualization system for exploratory research and analysis’, *Journal of Computational Chemistry*, 25(13), pp. 1605–1612. doi: 10.1002/jcc.20084.

Phillippy, B. Q. and Bland, J. M. (1988) ‘Gradient ion chromatography of inositol phosphates’, *Analytical Biochemistry*, 175(1), pp. 162–166. doi: 10.1016/0003-2697(88)90374-0.

Rivera-Solís, R. A. *et al.* (2014) ‘Chlamydomonas reinhardtii has a small family of purple acid phosphatase homologue genes that are differentially expressed in response to phytate’, *Annals of Microbiology*, 64(2), pp. 551–559. doi: 10.1007/s13213-013-0688-8.

Schenk, G. *et al.* (2008) ‘Crystal structures of a purple acid phosphatase, representing different steps of this enzyme’s catalytic cycle’, *BMC Struct Biol*, 8, p. 6. doi: 10.1186/1472-6807-8-6.

Schrodinger LLC (2015) ‘The PyMOL Molecular Graphics System, Version 1.3’.

Shu, B., Wang, P. and Xia, R. X. (2015) ‘Characterisation of the phytase gene in trifoliate orange (Poncirus trifoliata (L.) Raf.) seedlings’, *Scientia Horticulturae*. Elsevier, 194, pp. 222–229. doi: 10.1016/J.SCIENTA.2015.08.028.

Singh, P. *et al.* (2013) ‘Characterization and expression of codon optimized soybean phytase gene in E. coli’, *Indian Journal of Biochemistry and Biophysics*, 50(6), pp. 537–547. Available at: http://www.ncbi.nlm.nih.gov/pubmed/24772979 (Accessed: 31 March 2020).

Veiga, N. *et al.* (2014) ‘Coordination, microprotonation equilibria and conformational changes of *myo*-inositol hexakisphosphate with pertinence to its biological function’, *Dalton Trans.* The Royal Society of Chemistry, 43(43), pp. 16238–16251. doi: 10.1039/C4DT01350F.

Wang, X. *et al.* (2009) ‘Overexpressing AtPAP15 enhances phosphorus efficiency in soybean’, *Plant Physiology*. American Society of Plant Biologists, 151(1), pp. 233–240. doi: 10.1104/pp.109.138891.

Waterhouse, A. M. *et al.* (2009) ‘Sequence analysis Jalview Version 2-a multiple sequence alignment editor and analysis workbench’, *BIOINFORMATICS APPLICATIONS NOTE*, 25(9), pp. 1189–1191. doi: 10.1093/bioinformatics/btp033.

Wilkins, M. R. *et al.* (1999) ‘Protein identification and analysis tools in the ExPASy

server.', *Methods in molecular biology (Clifton, N.J.)*. Humana Press, pp. 531–552. doi: 10.1385/1-59259-584-7:531.

Winter, G., Lobley, C. M. C. and Prince, S. M. (2013) 'Decision making in xia2', *Acta Crystallogr D Biol Crystallogr.*, 69(7), pp. 1260–1273. doi: 10.1107/S0907444913015308.

Wongkaew, A., Srinives, P. and Nakasathien, S. (2013) 'Isolation and characterization of purple acid phosphatase gene during seedling development in mungbean', *Biologia Plantarum*, 57(2), pp. 267–273. doi: 10.1007/s10535-012-0292-y.

Xiao, K. *et al.* (2006) 'Ectopic Expression of a Phytase Gene from Medicago truncatula Barrel Medic Enhances Phosphorus Absorption in Plants', *Journal of Integrative Plant Biology*. John Wiley & Sons, Ltd, 48(1), pp. 35–43. doi: 10.1111/J.1744-7909.2006.00189.X.

Xiao, K., Harrison, M. J. and Wang, Z. Y. (2005) 'Transgenic expression of a novel M. truncatula phytase gene results in improved acquisition of organic phosphorus by Arabidopsis', *Planta*. Planta, 222(1), pp. 27–36. doi: 10.1007/s00425-005-1511-y.

Zhang, W. *et al.* (2008) 'An Arabidopsis Purple Acid Phosphatase with Phytase Activity Increases Foliar Ascorbate', *Plant Physiology*, 146(2), pp. 431–440. doi: 10.1104/pp.107.109934.

Zheng, H. *et al.* (2014) 'Validation of metal-binding sites in macromolecular structures with the CheckMyMetal web server', *Nat. Protoc.*, 9(1), pp. 156–170. doi: 10.1038/nprot.2013.172.

Zhu, H. *et al.* (2005) 'Expression patterns of purple acid phosphatase genes in Arabidopsis organs and functional analysis of AtPAP23 predominantly transcribed in flower', *Plant Molecular Biology*. Springer, 59(4), pp. 581–594. doi: 10.1007/s11103-005-0183-0.

VALIDATION REPORTS FOR PDB ENTRIES

1. 6GIT. PURPLE ACID PHYTASE FROM WHEAT ISOFORM B2 - PRODUCT COMPLEX
2. 6GIZ. PURPLE ACID PHYTASE FROM WHEAT ISOFORM B2 - SUBSTRATE COMPLEX
3. 6GJ2. PURPLE ACID PHYTASE FROM WHEAT ISOFORM B2 - COMPLEX WITH INOSITOL HEXASULPHATE
4. 6GJA. PURPLE ACID PHYTASE FROM WHEAT ISOFORM B2 - H229A MUTANT

Full wwPDB X-ray Structure Validation Report (i)

Aug 10, 2020 – 11:15 AM BST

PDB ID	:	6GIT
Title	:	PURPLE ACID PHYTASE FROM WHEAT ISOFORM B2 - PRODUCT COMPLEX
Authors	:	Faba-Rodriguez, R.; Brearley, C.A.; Hemmings, A.M.
Deposited on	:	2018-05-15
Resolution	:	1.42 Å(reported)

This is a Full wwPDB X-ray Structure Validation Report for a publicly released PDB entry.

We welcome your comments at *validation@mail.wwpdb.org*
A user guide is available at
https://www.wwpdb.org/validation/2017/XrayValidationReportHelp
with specific help available everywhere you see the (i) symbol.

The following versions of software and data (see references (i)) were used in the production of this report:

MolProbity	:	4.02b-467
Mogul	:	1.8.5 (274361), CSD as541be (2020)
Xtriage (Phenix)	:	1.13
EDS	:	2.13.1
Percentile statistics	:	20191225.v01 (using entries in the PDB archive December 25th 2019)
Refmac	:	5.8.0158
CCP4	:	7.0.044 (Gargrove)
Ideal geometry (proteins)	:	Engh & Huber (2001)
Ideal geometry (DNA, RNA)	:	Parkinson et al. (1996)
Validation Pipeline (wwPDB-VP)	:	2.13.1

1 Overall quality at a glance (i)

The following experimental techniques were used to determine the structure:
X-RAY DIFFRACTION

The reported resolution of this entry is 1.42 Å.

Percentile scores (ranging between 0-100) for global validation metrics of the entry are shown in the following graphic. The table shows the number of entries on which the scores are based.

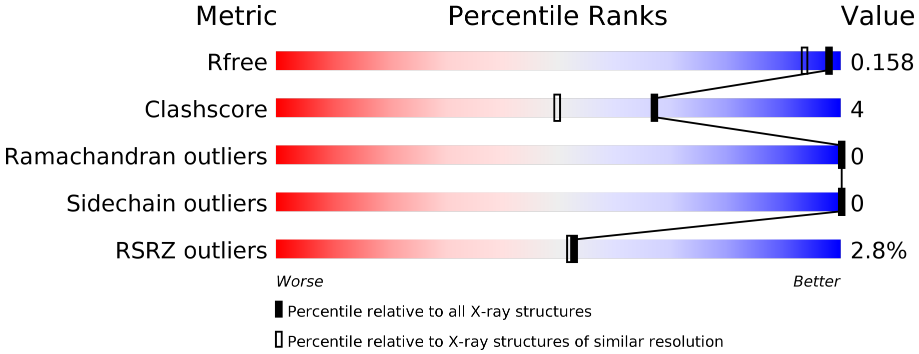

Metric	Whole archive (#Entries)	Similar resolution (#Entries, resolution range(Å))
R_{free}	130704	2579 (1.44-1.40)
Clashscore	141614	2696 (1.44-1.40)
Ramachandran outliers	138981	2632 (1.44-1.40)
Sidechain outliers	138945	2631 (1.44-1.40)
RSRZ outliers	127900	2528 (1.44-1.40)

The table below summarises the geometric issues observed across the polymeric chains and their fit to the electron density. The red, orange, yellow and green segments on the lower bar indicate the fraction of residues that contain outliers for >=3, 2, 1 and 0 types of geometric quality criteria respectively. A grey segment represents the fraction of residues that are not modelled. The numeric value for each fraction is indicated below the corresponding segment, with a dot representing fractions <=5% The upper red bar (where present) indicates the fraction of residues that have poor fit to the electron density. The numeric value is given above the bar.

Mol	Chain	Length	Quality of chain
1	A	516	3% / 92% 6% •
2	B	2	100%

2 Entry composition (i)

There are 10 unique types of molecules in this entry. The entry contains 5093 atoms, of which 283 are hydrogens and 0 are deuteriums.

In the tables below, the ZeroOcc column contains the number of atoms modelled with zero occupancy, the AltConf column contains the number of residues with at least one atom in alternate conformation and the Trace column contains the number of residues modelled with at most 2 atoms.

- Molecule 1 is a protein called Purple acid phosphatase.

Mol	Chain	Residues	Atoms					ZeroOcc	AltConf	Trace
			Total	C	N	O	S			
1	A	507	4078	2597	684	772	25	0	24	0

There are 6 discrepancies between the modelled and reference sequences:

Chain	Residue	Modelled	Actual	Comment	Reference
A	511	HIS	-	expression tag	UNP C4PKL0
A	512	HIS	-	expression tag	UNP C4PKL0
A	513	HIS	-	expression tag	UNP C4PKL0
A	514	HIS	-	expression tag	UNP C4PKL0
A	515	HIS	-	expression tag	UNP C4PKL0
A	516	HIS	-	expression tag	UNP C4PKL0

- Molecule 2 is an oligosaccharide called 2-acetamido-2-deoxy-beta-D-glucopyranose-(1-4)-2-acetamido-2-deoxy-beta-D-glucopyranose.

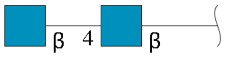

Mol	Chain	Residues	Atoms					ZeroOcc	AltConf	Trace
			Total	C	H	N	O			
2	B	2	55	16	27	2	10	0	0	0

- Molecule 3 is FE (III) ION (three-letter code: FE) (formula: Fe).

Mol	Chain	Residues	Atoms		ZeroOcc	AltConf
			Total	Fe		
3	A	2	2	2	0	0

- Molecule 4 is 2-acetamido-2-deoxy-beta-D-glucopyranose (three-letter code: NAG) (formula: $C_8H_{15}NO_6$).

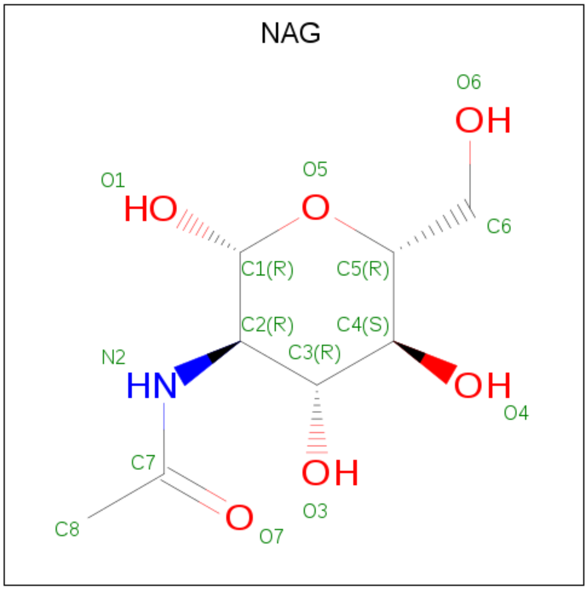

Mol	Chain	Residues	Atoms	ZeroOcc	AltConf
4	A	1	Total C H N O<br>28 8 14 1 5	0	0
4	A	1	Total C H N O<br>28 8 14 1 5	0	0
4	A	1	Total C H N O<br>28 8 14 1 5	0	0
4	A	1	Total C H N O<br>28 8 14 1 5	0	0
4	A	1	Total C H N O<br>28 8 14 1 5	0	0
4	A	1	Total C H N O<br>28 8 14 1 5	0	0

- Molecule 5 is PHOSPHATE ION (three-letter code: PO4) (formula: O_4P).

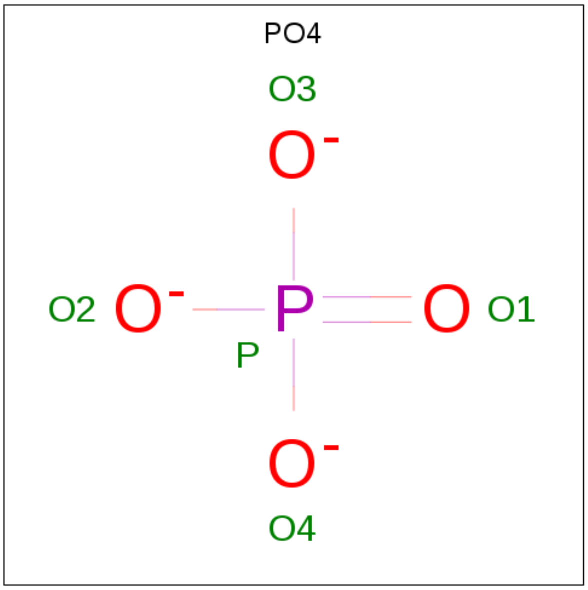

Mol	Chain	Residues	Atoms	ZeroOcc	AltConf
5	A	1	Total O P<br>5 4 1	0	0
5	A	1	Total O P<br>5 4 1	0	0

- Molecule 6 is DI(HYDROXYETHYL)ETHER (three-letter code: PEG) (formula: $C_4H_{10}O_3$).

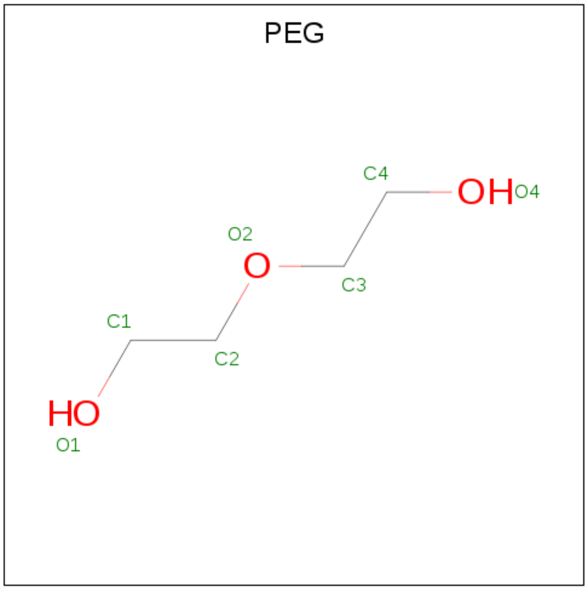

Mol	Chain	Residues	Atoms	ZeroOcc	AltConf
6	A	1	Total C H O<br>17 4 10 3	0	0
6	A	1	Total C H O<br>17 4 10 3	0	0

Continued on next page...

Continued from previous page...

Mol	Chain	Residues	Atoms	ZeroOcc	AltConf
6	A	1	Total C H O<br>17 4 10 3	0	0
6	A	1	Total C H O<br>17 4 10 3	0	0
6	A	1	Total C H O<br>17 4 10 3	0	0

- Molecule 7 is TRIETHYLENE GLYCOL (three-letter code: PGE) (formula: $C_6H_{14}O_4$).

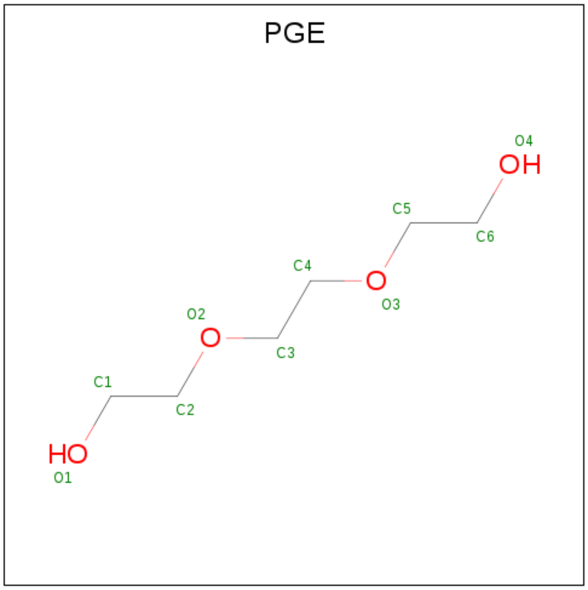

Mol	Chain	Residues	Atoms	ZeroOcc	AltConf
7	A	1	Total C H O<br>24 6 14 4	0	0
7	A	1	Total C H O<br>24 6 14 4	0	0

- Molecule 8 is PENTAETHYLENE GLYCOL (three-letter code: 1PE) (formula: $C_{10}H_{22}O_6$).

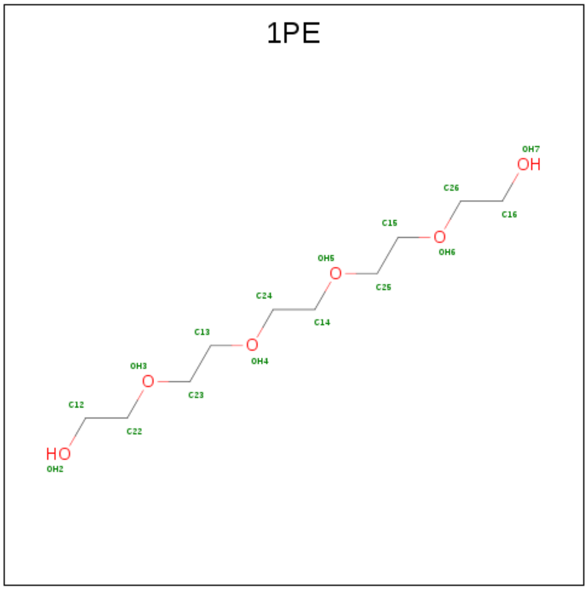

Mol	Chain	Residues	Atoms				ZeroOcc	AltConf
			Total	C	H	O		
8	A	1	38	10	22	6	0	0

- Molecule 9 is 1,2-ETHANEDIOL (three-letter code: EDO) (formula: $C_2H_6O_2$).

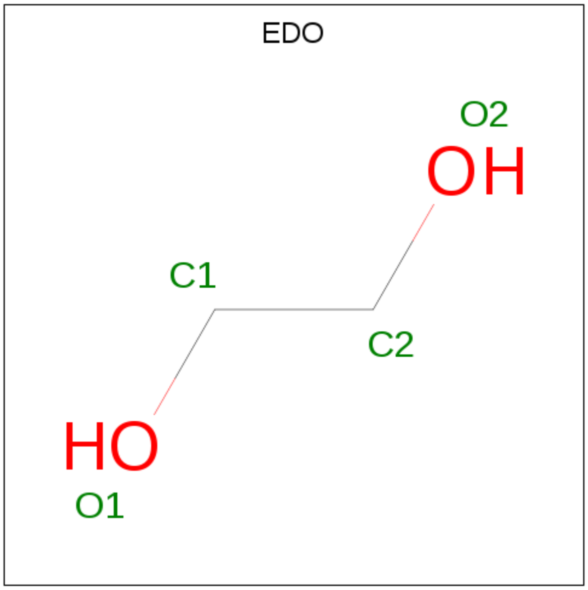

Mol	Chain	Residues	Atoms				ZeroOcc	AltConf
			Total	C	H	O		
9	A	1	10	2	6	2	0	0
9	A	1	10	2	6	2	0	0
9	A	1	10	2	6	2	0	0

Continued on next page...

Continued from previous page...

Mol	Chain	Residues	Atoms	ZeroOcc	AltConf
9	A	1	Total C H O<br>10 2 6 2	0	0
9	A	1	Total C H O<br>10 2 6 2	0	0
9	A	1	Total C H O<br>10 2 6 2	0	0
9	A	1	Total C H O<br>10 2 6 2	0	0
9	A	1	Total C H O<br>10 2 6 2	0	0
9	A	1	Total C H O<br>10 2 6 2	0	0
9	A	1	Total C H O<br>10 2 6 2	0	0
9	A	1	Total C H O<br>10 2 6 2	0	0
9	A	1	Total C H O<br>10 2 6 2	0	0

- Molecule 10 is water.

Mol	Chain	Residues	Atoms	ZeroOcc	AltConf
10	A	489	Total O<br>489 489	0	0

3 Residue-property plots (i)

These plots are drawn for all protein, RNA, DNA and oligosaccharide chains in the entry. The first graphic for a chain summarises the proportions of the various outlier classes displayed in the second graphic. The second graphic shows the sequence view annotated by issues in geometry and electron density. Residues are color-coded according to the number of geometric quality criteria for which they contain at least one outlier: green = 0, yellow = 1, orange = 2 and red = 3 or more. A red dot above a residue indicates a poor fit to the electron density (RSRZ > 2). Stretches of 2 or more consecutive residues without any outlier are shown as a green connector. Residues present in the sample, but not in the model, are shown in grey.

- Molecule 1: Purple acid phosphatase

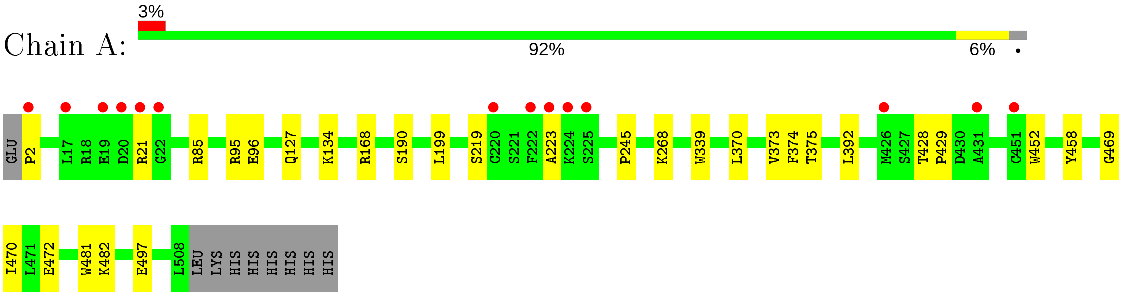

- Molecule 2: 2-acetamido-2-deoxy-beta-D-glucopyranose-(1-4)-2-acetamido-2-deoxy-beta-D-glucopyranose

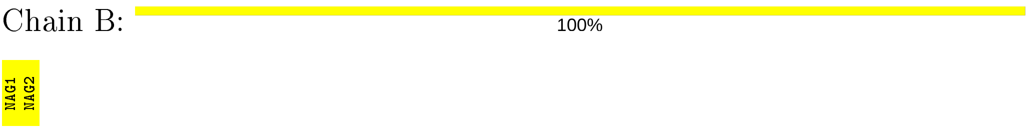

4 Data and refinement statistics (i)

Property	Value	Source
Space group	H 3	Depositor
Cell constants<br>a, b, c, α, β, γ	126.48Å 126.48Å 106.80Å<br>90.00° 90.00° 120.00°	Depositor
Resolution (Å)	63.24 – 1.42<br>63.24 – 1.42	Depositor<br>EDS
% Data completeness<br>(in resolution range)	92.6 (63.24-1.42)<br>92.6 (63.24-1.42)	Depositor<br>EDS
R_{merge}	0.05	Depositor
R_{sym}	(Not available)	Depositor
$< I/\sigma(I) >$ [1]	2.11 (at 1.42Å)	Xtriage
Refinement program	PHENIX (1.11.1_2575: ???)	Depositor
R, R_{free}	0.132 , 0.158<br>0.132 , 0.158	Depositor<br>DCC
R_{free} test set	5746 reflections (5.14%)	wwPDB-VP
Wilson B-factor (Å^2)	14.5	Xtriage
Anisotropy	0.135	Xtriage
Bulk solvent k_{sol}(e/Å^3), B_{sol}(Å^2)	0.38 , 47.0	EDS
L-test for twinning[2]	$< \|L\| > = 0.49$, $< L^2 > = 0.33$	Xtriage
Estimated twinning fraction	0.018 for h,-h-k,-l	Xtriage
F_o,F_c correlation	0.98	EDS
Total number of atoms	5093	wwPDB-VP
Average B, all atoms (Å^2)	23.0	wwPDB-VP

Xtriage's analysis on translational NCS is as follows: *The largest off-origin peak in the Patterson function is 3.87% of the height of the origin peak. No significant pseudotranslation is detected.*

[1]Intensities estimated from amplitudes.

[2]Theoretical values of $< |L| >$, $< L^2 >$ for acentric reflections are 0.5, 0.333 respectively for untwinned datasets, and 0.375, 0.2 for perfectly twinned datasets.

5 Model quality (i)

5.1 Standard geometry (i)

Bond lengths and bond angles in the following residue types are not validated in this section: PGE, NAG, PO4, EDO, 1PE, FE, PEG

The Z score for a bond length (or angle) is the number of standard deviations the observed value is removed from the expected value. A bond length (or angle) with $|Z| > 5$ is considered an outlier worth inspection. RMSZ is the root-mean-square of all Z scores of the bond lengths (or angles).

Mol	Chain	Bond lengths		Bond angles	
		RMSZ	#|Z| >5	RMSZ	#|Z| >5
1	A	0.32	0/4282	0.54	0/5836

There are no bond length outliers.

There are no bond angle outliers.

There are no chirality outliers.

There are no planarity outliers.

5.2 Too-close contacts (i)

In the following table, the Non-H and H(model) columns list the number of non-hydrogen atoms and hydrogen atoms in the chain respectively. The H(added) column lists the number of hydrogen atoms added and optimized by MolProbity. The Clashes column lists the number of clashes within the asymmetric unit, whereas Symm-Clashes lists symmetry related clashes.

Mol	Chain	Non-H	H(model)	H(added)	Clashes	Symm-Clashes
1	A	4078	0	3879	32	0
2	B	28	27	25	0	0
3	A	2	0	0	1	0
4	A	84	84	78	0	0
5	A	10	0	0	1	0
6	A	35	50	50	3	0
7	A	20	28	28	1	0
8	A	16	22	22	0	0
9	A	48	72	72	11	0
10	A	489	0	0	5	0
All	All	4810	283	4154	33	0

The all-atom clashscore is defined as the number of clashes found per 1000 atoms (including hydrogen atoms). The all-atom clashscore for this structure is 4.

All (33) close contacts within the same asymmetric unit are listed below, sorted by their clash magnitude.

Atom-1	Atom-2	Interatomic distance (Å)	Clash overlap (Å)
1:A:190[B]:SER:OG	10:A:701:HOH:O	2.09	0.70
1:A:134:LYS:NZ	7:A:619:PGE:O4	2.15	0.70
1:A:219:SER:HA	1:A:223:ALA:HB2	1.75	0.67
1:A:2:PRO:HG3	1:A:497[A]:GLU:OE2	1.96	0.65
3:A:601:FE:FE	5:A:611:PO4:O1	1.49	0.65
1:A:85:ARG:NH1	10:A:702:HOH:O	2.21	0.65
1:A:470:ILE:HG13	9:A:623:EDO:H12	1.78	0.64
1:A:21:ARG:HE	9:A:624:EDO:C2	2.10	0.64
1:A:21:ARG:HE	9:A:624:EDO:H22	1.61	0.64
1:A:469:GLY:HA3	1:A:481:TRP:CH2	2.40	0.57
1:A:95:ARG:HE	9:A:621:EDO:H22	1.69	0.56
1:A:245:PRO:HB2	6:A:614:PEG:H12	1.88	0.56
1:A:2:PRO:HB3	1:A:497[A]:GLU:HG3	1.88	0.55
1:A:127[A]:GLN:NE2	10:A:714:HOH:O	2.39	0.55
1:A:472:GLU:OE1	9:A:623:EDO:O2	2.26	0.53
1:A:168[A]:ARG:HH12	9:A:627:EDO:C1	2.21	0.53
1:A:96:GLU:O	9:A:631:EDO:H22	2.08	0.53
1:A:127[A]:GLN:HG2	10:A:709:HOH:O	2.09	0.52
1:A:482:LYS:HE3	9:A:623:EDO:O1	2.09	0.52
1:A:168[A]:ARG:HH12	9:A:627:EDO:H12	1.75	0.51
1:A:452:TRP:HA	6:A:617:PEG:H21	1.92	0.50
1:A:199:LEU:HD11	1:A:374:PHE:HB3	1.94	0.50
1:A:452:TRP:HA	6:A:617:PEG:H41	1.93	0.49
1:A:428:THR:N	1:A:429:PRO:CD	2.77	0.48
1:A:428:THR:N	1:A:429:PRO:HD2	2.29	0.48
1:A:370:LEU:HD21	1:A:373:VAL:HG22	1.99	0.45
1:A:268[B]:LYS:HE2	1:A:268[B]:LYS:HB3	1.75	0.43
1:A:482:LYS:HG2	1:A:497[B]:GLU:HG3	2.00	0.43
1:A:21:ARG:HE	9:A:624:EDO:H21	1.82	0.42
1:A:339:TRP:O	1:A:375:THR:HA	2.20	0.42
1:A:392:LEU:HD21	1:A:458:TYR:HA	2.02	0.42
1:A:127[A]:GLN:NE2	10:A:725:HOH:O	2.50	0.41
1:A:96:GLU:O	9:A:631:EDO:C2	2.69	0.41

There are no symmetry-related clashes.

5.3 Torsion angles (i)

5.3.1 Protein backbone (i)

In the following table, the Percentiles column shows the percent Ramachandran outliers of the chain as a percentile score with respect to all X-ray entries followed by that with respect to entries of similar resolution.

The Analysed column shows the number of residues for which the backbone conformation was analysed, and the total number of residues.

Mol	Chain	Analysed	Favoured	Allowed	Outliers	Percentiles	
1	A	531/516 (103%)	516 (97%)	15 (3%)	0	100	100

There are no Ramachandran outliers to report.

5.3.2 Protein sidechains (i)

In the following table, the Percentiles column shows the percent sidechain outliers of the chain as a percentile score with respect to all X-ray entries followed by that with respect to entries of similar resolution.

The Analysed column shows the number of residues for which the sidechain conformation was analysed, and the total number of residues.

Mol	Chain	Analysed	Rotameric	Outliers	Percentiles	
1	A	439/425 (103%)	439 (100%)	0	100	100

There are no protein residues with a non-rotameric sidechain to report.

Some sidechains can be flipped to improve hydrogen bonding and reduce clashes. There are no such sidechains identified.

5.3.3 RNA (i)

There are no RNA molecules in this entry.

5.4 Non-standard residues in protein, DNA, RNA chains (i)

There are no non-standard protein/DNA/RNA residues in this entry.

5.5 Carbohydrates (i)

2 monosaccharides are modelled in this entry.

In the following table, the Counts columns list the number of bonds (or angles) for which Mogul statistics could be retrieved, the number of bonds (or angles) that are observed in the model and the number of bonds (or angles) that are defined in the Chemical Component Dictionary. The Link column lists molecule types, if any, to which the group is linked. The Z score for a bond length (or angle) is the number of standard deviations the observed value is removed from the expected value. A bond length (or angle) with $|Z| > 2$ is considered an outlier worth inspection. RMSZ is the root-mean-square of all Z scores of the bond lengths (or angles).

Mol	Type	Chain	Res	Link	Bond lengths			Bond angles		
					Counts	RMSZ	$\#\|Z\| > 2$	Counts	RMSZ	$\#\|Z\| > 2$
2	NAG	B	1	1,2	14,14,15	1.89	3 (21%)	17,19,21	1.15	1 (5%)
2	NAG	B	2	2	14,14,15	1.98	4 (28%)	17,19,21	1.23	2 (11%)

In the following table, the Chirals column lists the number of chiral outliers, the number of chiral centers analysed, the number of these observed in the model and the number defined in the Chemical Component Dictionary. Similar counts are reported in the Torsion and Rings columns. '-' means no outliers of that kind were identified.

Mol	Type	Chain	Res	Link	Chirals	Torsions	Rings
2	NAG	B	1	1,2	-	2/6/23/26	0/1/1/1
2	NAG	B	2	2	-	0/6/23/26	0/1/1/1

All (7) bond length outliers are listed below:

Mol	Chain	Res	Type	Atoms	Z	Observed(Å)	Ideal(Å)
2	B	2	NAG	O5-C1	4.47	1.50	1.43
2	B	1	NAG	O5-C1	4.28	1.50	1.43
2	B	2	NAG	C7-N2	3.68	1.47	1.34
2	B	1	NAG	C7-N2	3.60	1.46	1.34
2	B	2	NAG	C2-N2	2.42	1.50	1.46
2	B	1	NAG	C2-N2	2.41	1.50	1.46
2	B	2	NAG	O5-C5	2.09	1.47	1.43

All (3) bond angle outliers are listed below:

Mol	Chain	Res	Type	Atoms	Z	Observed(^{o})	Ideal(^{o})
2	B	2	NAG	C8-C7-N2	2.85	120.93	116.10
2	B	1	NAG	C8-C7-N2	2.51	120.35	116.10
2	B	2	NAG	C2-N2-C7	-2.39	119.50	122.90

There are no chirality outliers.

All (2) torsion outliers are listed below:

Mol	Chain	Res	Type	Atoms
2	B	1	NAG	C8-C7-N2-C2
2	B	1	NAG	O7-C7-N2-C2

There are no ring outliers.

No monomer is involved in short contacts.

The following is a two-dimensional graphical depiction of Mogul quality analysis of bond lengths, bond angles, torsion angles, and ring geometry for oligosaccharide.

Oligosaccharide Chain B

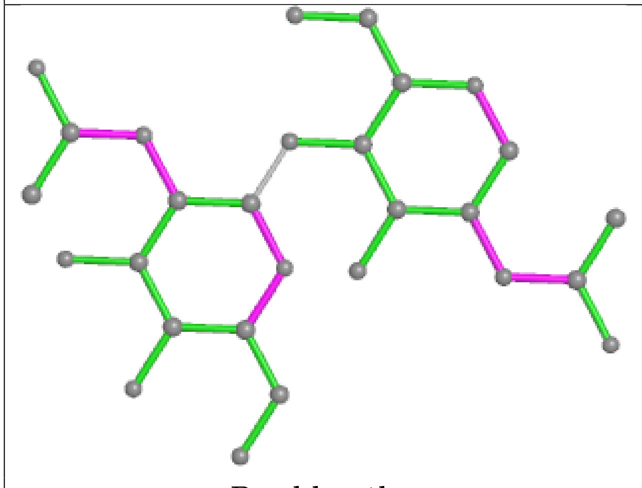

Bond lengths

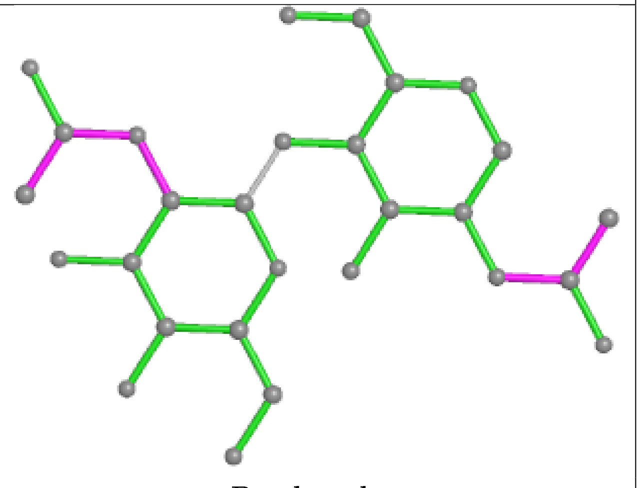

Bond angles

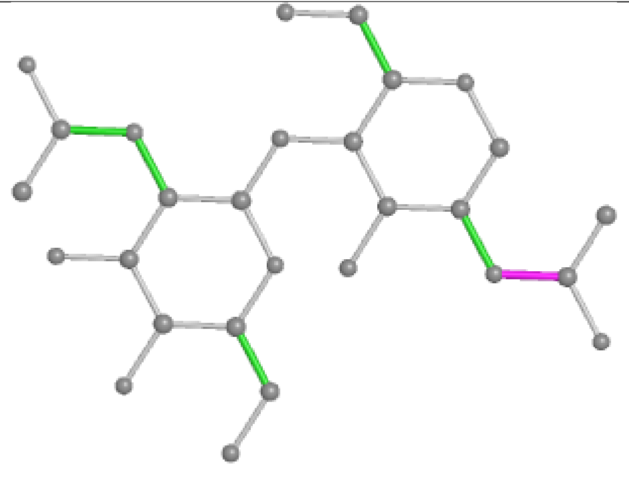

Torsions

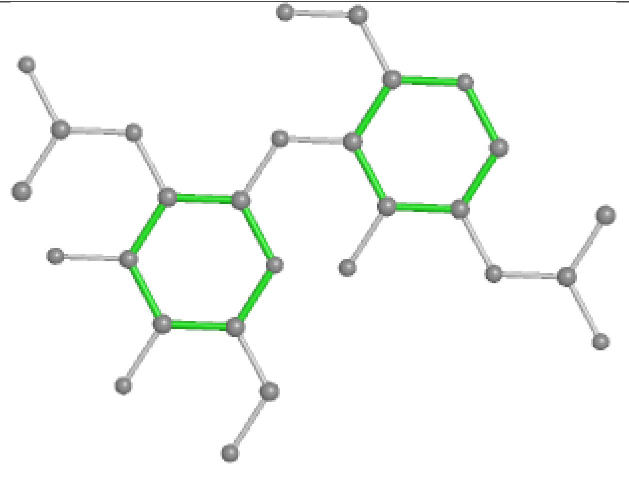

Rings

5.6 Ligand geometry (i)

Of 30 ligands modelled in this entry, 2 are monoatomic - leaving 28 for Mogul analysis.

In the following table, the Counts columns list the number of bonds (or angles) for which Mogul statistics could be retrieved, the number of bonds (or angles) that are observed in the model and the number of bonds (or angles) that are defined in the Chemical Component Dictionary. The Link column lists molecule types, if any, to which the group is linked. The Z score for a bond length (or angle) is the number of standard deviations the observed value is removed from the expected value. A bond length (or angle) with $|Z| > 2$ is considered an outlier worth inspection. RMSZ is the root-mean-square of all Z scores of the bond lengths (or angles).

Mol	Type	Chain	Res	Link	Bond lengths			Bond angles		
					Counts	RMSZ	$\#\|Z\| > 2$	Counts	RMSZ	$\#\|Z\| > 2$
9	EDO	A	632	-	3,3,3	0.42	0	2,2,2	0.15	0
7	PGE	A	618	-	9,9,9	0.51	0	8,8,8	0.34	0
5	PO4	A	611	3	4,4,4	0.87	0	6,6,6	0.44	0
8	1PE	A	620	-	15,15,15	0.53	0	14,14,14	0.40	0
9	EDO	A	628	-	3,3,3	0.47	0	2,2,2	0.26	0
5	PO4	A	612	-	4,4,4	0.93	0	6,6,6	0.50	0
6	PEG	A	614	-	6,6,6	0.47	0	5,5,5	0.88	0
6	PEG	A	616	-	6,6,6	0.47	0	5,5,5	0.32	0
6	PEG	A	617	-	6,6,6	0.50	0	5,5,5	0.56	0
4	NAG	A	604	1	14,14,15	1.56	2 (14%)	17,19,21	0.97	1 (5%)
4	NAG	A	605	1	14,14,15	1.99	4 (28%)	17,19,21	1.25	2 (11%)
9	EDO	A	631	-	3,3,3	0.45	0	2,2,2	0.22	0
9	EDO	A	630	-	3,3,3	0.47	0	2,2,2	0.29	0
4	NAG	A	603	1	14,14,15	1.76	3 (21%)	17,19,21	1.02	2 (11%)
4	NAG	A	607	1	14,14,15	1.92	3 (21%)	17,19,21	1.05	2 (11%)
7	PGE	A	619	-	9,9,9	0.51	0	8,8,8	0.24	0
9	EDO	A	621	-	3,3,3	0.47	0	2,2,2	0.39	0
9	EDO	A	625	-	3,3,3	0.48	0	2,2,2	0.28	0
4	NAG	A	608	1	14,14,15	1.79	3 (21%)	17,19,21	1.10	2 (11%)
9	EDO	A	624	-	3,3,3	0.45	0	2,2,2	0.27	0
9	EDO	A	629	-	3,3,3	0.47	0	2,2,2	0.33	0
9	EDO	A	626	-	3,3,3	0.45	0	2,2,2	0.26	0
9	EDO	A	627	-	3,3,3	0.47	0	2,2,2	0.31	0
9	EDO	A	622	-	3,3,3	0.47	0	2,2,2	0.34	0
9	EDO	A	623	-	3,3,3	0.49	0	2,2,2	0.36	0
4	NAG	A	606	1	14,14,15	1.98	5 (35%)	17,19,21	1.67	3 (17%)
6	PEG	A	615	-	6,6,6	0.49	0	5,5,5	0.21	0
6	PEG	A	613	-	6,6,6	0.48	0	5,5,5	0.28	0

In the following table, the Chirals column lists the number of chiral outliers, the number of chiral centers analysed, the number of these observed in the model and the number defined in the Chemical Component Dictionary. Similar counts are reported in the Torsion and Rings columns. '-' means no outliers of that kind were identified.

Mol	Type	Chain	Res	Link	Chirals	Torsions	Rings
9	EDO	A	632	-	-	1/1/1/1	-
7	PGE	A	618	-	-	1/7/7/7	-
8	1PE	A	620	-	-	1/13/13/13	-
9	EDO	A	628	-	-	0/1/1/1	-
6	PEG	A	615	-	-	0/4/4/4	-
6	PEG	A	614	-	-	3/4/4/4	-
6	PEG	A	616	-	-	1/4/4/4	-
6	PEG	A	617	-	-	1/4/4/4	-
4	NAG	A	604	1	-	0/6/23/26	0/1/1/1
4	NAG	A	605	1	-	1/6/23/26	0/1/1/1
9	EDO	A	631	-	-	1/1/1/1	-
9	EDO	A	630	-	-	0/1/1/1	-
4	NAG	A	603	1	-	0/6/23/26	0/1/1/1
4	NAG	A	607	1	-	0/6/23/26	0/1/1/1
7	PGE	A	619	-	-	0/7/7/7	-
9	EDO	A	621	-	-	0/1/1/1	-
9	EDO	A	625	-	-	0/1/1/1	-
4	NAG	A	608	1	-	0/6/23/26	0/1/1/1
9	EDO	A	624	-	-	1/1/1/1	-
9	EDO	A	629	-	-	0/1/1/1	-
9	EDO	A	626	-	-	0/1/1/1	-
9	EDO	A	627	-	-	0/1/1/1	-
9	EDO	A	622	-	-	1/1/1/1	-
9	EDO	A	623	-	-	1/1/1/1	-
4	NAG	A	606	1	-	0/6/23/26	0/1/1/1
6	PEG	A	613	-	-	0/4/4/4	-

All (20) bond length outliers are listed below:

Mol	Chain	Res	Type	Atoms	Z	Observed(Å)	Ideal(Å)
4	A	605	NAG	O5-C1	4.67	1.51	1.43
4	A	606	NAG	O5-C1	4.39	1.50	1.43
4	A	607	NAG	O5-C1	4.37	1.50	1.43
4	A	603	NAG	O5-C1	3.89	1.49	1.43
4	A	608	NAG	O5-C1	3.86	1.49	1.43
4	A	604	NAG	O5-C1	3.56	1.49	1.43
4	A	606	NAG	C7-N2	3.51	1.46	1.34
4	A	603	NAG	C7-N2	3.47	1.46	1.34
4	A	607	NAG	C7-N2	3.47	1.46	1.34
4	A	605	NAG	C7-N2	3.46	1.46	1.34
4	A	608	NAG	C7-N2	3.45	1.46	1.34
4	A	604	NAG	C7-N2	3.00	1.44	1.34
4	A	606	NAG	O5-C5	2.35	1.48	1.43

Continued on next page...

Continued from previous page...

Mol	Chain	Res	Type	Atoms	Z	Observed(Å)	Ideal(Å)
4	A	605	NAG	O5-C5	2.26	1.48	1.43
4	A	607	NAG	C2-N2	2.24	1.50	1.46
4	A	605	NAG	C2-N2	2.17	1.50	1.46
4	A	606	NAG	C2-N2	2.14	1.50	1.46
4	A	603	NAG	C2-N2	2.11	1.49	1.46
4	A	608	NAG	C2-N2	2.10	1.49	1.46
4	A	606	NAG	C3-C2	-2.07	1.48	1.52

All (12) bond angle outliers are listed below:

Mol	Chain	Res	Type	Atoms	Z	Observed(^{o})	Ideal(^{o})
4	A	606	NAG	C1-C2-N2	-3.87	103.89	110.49
4	A	606	NAG	O5-C1-C2	3.48	116.79	111.29
4	A	608	NAG	C2-N2-C7	-2.88	118.81	122.90
4	A	605	NAG	C2-N2-C7	-2.55	119.28	122.90
4	A	605	NAG	C8-C7-N2	2.52	120.37	116.10
4	A	608	NAG	C8-C7-N2	2.43	120.21	116.10
4	A	606	NAG	C8-C7-N2	2.31	120.01	116.10
4	A	607	NAG	C8-C7-N2	2.20	119.83	116.10
4	A	607	NAG	C2-N2-C7	-2.19	119.79	122.90
4	A	603	NAG	C2-N2-C7	-2.19	119.79	122.90
4	A	603	NAG	C8-C7-N2	2.14	119.72	116.10
4	A	604	NAG	O5-C1-C2	-2.04	108.07	111.29

There are no chirality outliers.

All (13) torsion outliers are listed below:

Mol	Chain	Res	Type	Atoms
6	A	614	PEG	C4-C3-O2-C2
6	A	614	PEG	O1-C1-C2-O2
9	A	632	EDO	O1-C1-C2-O2
9	A	631	EDO	O1-C1-C2-O2
7	A	618	PGE	C1-C2-O2-C3
6	A	614	PEG	C1-C2-O2-C3
9	A	624	EDO	O1-C1-C2-O2
8	A	620	1PE	C23-C13-OH4-C24
4	A	605	NAG	O5-C5-C6-O6
6	A	617	PEG	C4-C3-O2-C2
9	A	622	EDO	O1-C1-C2-O2
6	A	616	PEG	C4-C3-O2-C2
9	A	623	EDO	O1-C1-C2-O2

There are no ring outliers.

9 monomers are involved in 16 short contacts:

Mol	Chain	Res	Type	Clashes	Symm-Clashes
5	A	611	PO4	1	0
6	A	614	PEG	1	0
6	A	617	PEG	2	0
9	A	631	EDO	2	0
7	A	619	PGE	1	0
9	A	621	EDO	1	0
9	A	624	EDO	3	0
9	A	627	EDO	2	0
9	A	623	EDO	3	0

5.7 Other polymers (i)

There are no such residues in this entry.

5.8 Polymer linkage issues (i)

There are no chain breaks in this entry.

6 Fit of model and data (i)

6.1 Protein, DNA and RNA chains (i)

In the following table, the column labelled '#RSRZ> 2' contains the number (and percentage) of RSRZ outliers, followed by percent RSRZ outliers for the chain as percentile scores relative to all X-ray entries and entries of similar resolution. The OWAB column contains the minimum, median, 95th percentile and maximum values of the occupancy-weighted average B-factor per residue. The column labelled 'Q< 0.9' lists the number of (and percentage) of residues with an average occupancy less than 0.9.

Mol	Chain	Analysed	<RSRZ>	#RSRZ>2			OWAB(Å^2)	Q<0.9
1	A	507/516 (98%)	-0.17	14 (2%)	53	52	11, 16, 33, 70	0

All (14) RSRZ outliers are listed below:

Mol	Chain	Res	Type	RSRZ
1	A	223	ALA	5.2
1	A	220	CYS	4.9
1	A	20	ASP	3.7
1	A	21	ARG	3.3
1	A	224	LYS	3.1
1	A	222	PHE	2.8
1	A	426	MET	2.6
1	A	22	GLY	2.6
1	A	2	PRO	2.5
1	A	451	CYS	2.4
1	A	225	SER	2.3
1	A	431	ALA	2.3
1	A	17	LEU	2.2
1	A	19	GLU	2.2

6.2 Non-standard residues in protein, DNA, RNA chains (i)

There are no non-standard protein/DNA/RNA residues in this entry.

6.3 Carbohydrates (i)

In the following table, the Atoms column lists the number of modelled atoms in the group and the number defined in the chemical component dictionary. The B-factors column lists the minimum, median, 95th percentile and maximum values of B factors of atoms in the group. The column labelled 'Q< 0.9' lists the number of atoms with occupancy less than 0.9.

Mol	Type	Chain	Res	Atoms	RSCC	RSR	B-factors(Å^2)	Q<0.9
2	NAG	B	2	14/15	0.81	0.18	43,50,60,61	28
2	NAG	B	1	14/15	0.92	0.13	27,40,51,51	0

The following is a graphical depiction of the model fit to experimental electron density for oligosaccharide. Each fit is shown from different orientation to approximate a three-dimensional view.

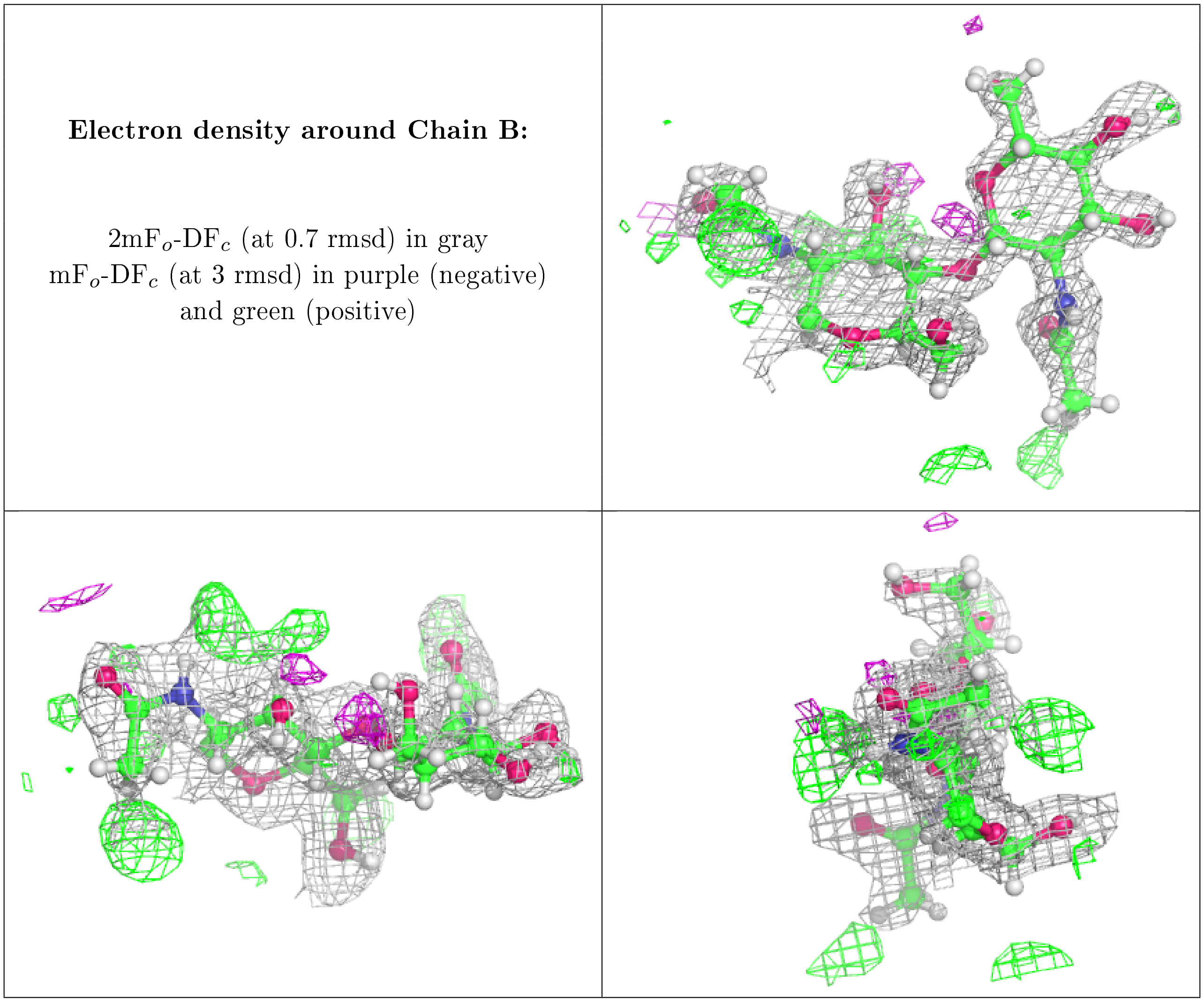

6.4 Ligands (i)

In the following table, the Atoms column lists the number of modelled atoms in the group and the number defined in the chemical component dictionary. The B-factors column lists the minimum, median, 95th percentile and maximum values of B factors of atoms in the group. The column labelled 'Q< 0.9' lists the number of atoms with occupancy less than 0.9.

Mol	Type	Chain	Res	Atoms	RSCC	RSR	B-factors(Å^2)	Q<0.9
4	NAG	A	606	14/15	0.75	0.23	49,61,73,73	28

Continued on next page...

Continued from previous page...

Mol	Type	Chain	Res	Atoms	RSCC	RSR	B-factors(Å^2)	Q<0.9
9	EDO	A	624	4/4	0.76	0.19	47,57,59,60	0
6	PEG	A	617	7/7	0.77	0.28	47,58,69,70	0
9	EDO	A	627	4/4	0.78	0.14	56,67,67,68	0
6	PEG	A	613	7/7	0.78	0.16	53,64,67,68	0
9	EDO	A	625	4/4	0.80	0.14	60,72,74,75	0
4	NAG	A	607	14/15	0.82	0.20	35,44,53,55	28
7	PGE	A	619	10/10	0.83	0.15	55,67,73,74	0
6	PEG	A	615	7/7	0.83	0.12	56,67,70,70	0
6	PEG	A	614	7/7	0.83	0.16	45,54,63,63	0
9	EDO	A	628	4/4	0.84	0.15	57,68,69,71	0
9	EDO	A	626	4/4	0.84	0.14	44,53,56,58	0
9	EDO	A	630	4/4	0.85	0.12	52,62,63,63	0
6	PEG	A	616	7/7	0.85	0.13	58,69,70,70	0
9	EDO	A	632	4/4	0.86	0.20	30,37,42,44	0
9	EDO	A	629	4/4	0.87	0.23	47,57,59,60	0
9	EDO	A	621	4/4	0.89	0.09	40,48,49,50	0
9	EDO	A	623	4/4	0.91	0.15	40,48,48,51	0
4	NAG	A	605	14/15	0.91	0.14	36,45,56,58	0
7	PGE	A	618	10/10	0.92	0.11	40,48,58,60	0
8	1PE	A	620	16/16	0.92	0.16	33,43,63,65	0
9	EDO	A	631	4/4	0.94	0.20	31,37,41,41	0
4	NAG	A	603	14/15	0.95	0.12	20,28,34,36	0
4	NAG	A	608	14/15	0.95	0.17	26,33,47,47	0
5	PO4	A	612	5/5	0.95	0.21	56,57,58,59	0
9	EDO	A	622	4/4	0.95	0.06	51,62,63,64	0
3	FE	A	601	1/1	0.96	0.09	44,44,44,44	1
4	NAG	A	604	14/15	0.98	0.08	14,18,23,26	0
5	PO4	A	611	5/5	0.98	0.07	22,27,29,40	0
3	FE	A	602	1/1	1.00	0.06	12,12,12,12	1

6.5 Other polymers ⓘ

There are no such residues in this entry.

Full wwPDB X-ray Structure Validation Report (i)

Aug 7, 2020 – 12:14 PM BST

PDB ID	:	6GIZ
Title	:	PURPLE ACID PHYTASE FROM WHEAT ISOFORM B2 - SUBSTRATE COMPLEX
Authors	:	Faba-Rodriguez, R.; Brearley, C.A.; Hemmings, A.M.
Deposited on	:	2018-05-15
Resolution	:	1.54 Å(reported)

This is a Full wwPDB X-ray Structure Validation Report for a publicly released PDB entry.

We welcome your comments at *validation@mail.wwpdb.org*
A user guide is available at
https://www.wwpdb.org/validation/2017/XrayValidationReportHelp
with specific help available everywhere you see the (i) symbol.

The following versions of software and data (see references (i)) were used in the production of this report:

MolProbity	:	4.02b-467
Mogul	:	1.8.5 (274361), CSD as541be (2020)
Xtriage (Phenix)	:	1.13
EDS	:	2.13.1
Percentile statistics	:	20191225.v01 (using entries in the PDB archive December 25th 2019)
Refmac	:	5.8.0158
CCP4	:	7.0.044 (Gargrove)
Ideal geometry (proteins)	:	Engh & Huber (2001)
Ideal geometry (DNA, RNA)	:	Parkinson et al. (1996)
Validation Pipeline (wwPDB-VP)	:	2.13.1

1 Overall quality at a glance (i)

The following experimental techniques were used to determine the structure:
X-RAY DIFFRACTION

The reported resolution of this entry is 1.54 Å.

Percentile scores (ranging between 0-100) for global validation metrics of the entry are shown in the following graphic. The table shows the number of entries on which the scores are based.

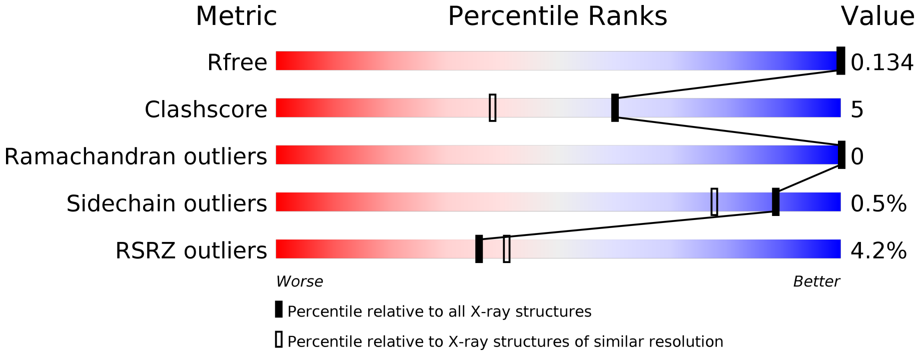

Metric	Whole archive (#Entries)	Similar resolution (#Entries, resolution range(Å))
R_{free}	130704	2556 (1.56-1.52)
Clashscore	141614	2634 (1.56-1.52)
Ramachandran outliers	138981	2580 (1.56-1.52)
Sidechain outliers	138945	2577 (1.56-1.52)
RSRZ outliers	127900	2524 (1.56-1.52)

The table below summarises the geometric issues observed across the polymeric chains and their fit to the electron density. The red, orange, yellow and green segments on the lower bar indicate the fraction of residues that contain outliers for >=3, 2, 1 and 0 types of geometric quality criteria respectively. A grey segment represents the fraction of residues that are not modelled. The numeric value for each fraction is indicated below the corresponding segment, with a dot representing fractions <=5% The upper red bar (where present) indicates the fraction of residues that have poor fit to the electron density. The numeric value is given above the bar.

Mol	Chain	Length	Quality of chain
1	A	516	4%<br>91% 7% •
2	B	2	100%
2	C	2	100%

The following table lists non-polymeric compounds, carbohydrate monomers and non-standard residues in protein, DNA, RNA chains that are outliers for geometric or electron-density-fit criteria:

Mol	Type	Chain	Res	Chirality	Geometry	Clashes	Electron density
8	EDO	A	624	-	-	X	-

2 Entry composition

There are 9 unique types of molecules in this entry. The entry contains 4915 atoms, of which 254 are hydrogens and 0 are deuteriums.

In the tables below, the ZeroOcc column contains the number of atoms modelled with zero occupancy, the AltConf column contains the number of residues with at least one atom in alternate conformation and the Trace column contains the number of residues modelled with at most 2 atoms.

- Molecule 1 is a protein called Purple acid phosphatase.

Mol	Chain	Residues	Atoms					ZeroOcc	AltConf	Trace
			Total	C	N	O	S			
1	A	504	3995	2541	668	762	24	5	14	0

There are 6 discrepancies between the modelled and reference sequences:

Chain	Residue	Modelled	Actual	Comment	Reference
A	511	HIS	-	expression tag	UNP C4PKL0
A	512	HIS	-	expression tag	UNP C4PKL0
A	513	HIS	-	expression tag	UNP C4PKL0
A	514	HIS	-	expression tag	UNP C4PKL0
A	515	HIS	-	expression tag	UNP C4PKL0
A	516	HIS	-	expression tag	UNP C4PKL0

- Molecule 2 is an oligosaccharide called 2-acetamido-2-deoxy-beta-D-glucopyranose-(1-4)-2-acetamido-2-deoxy-beta-D-glucopyranose.

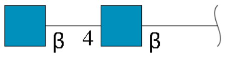

Mol	Chain	Residues	Atoms					ZeroOcc	AltConf	Trace
			Total	C	H	N	O			
2	B	2	55	16	27	2	10	0	0	0
2	C	2	55	16	27	2	10	0	0	0

- Molecule 3 is FE (III) ION (three-letter code: FE) (formula: Fe).

Mol	Chain	Residues	Atoms		ZeroOcc	AltConf
			Total	Fe		
3	A	2	2	2	0	0

- Molecule 4 is 2-acetamido-2-deoxy-beta-D-glucopyranose (three-letter code: NAG) (formula: $C_8H_{15}NO_6$).

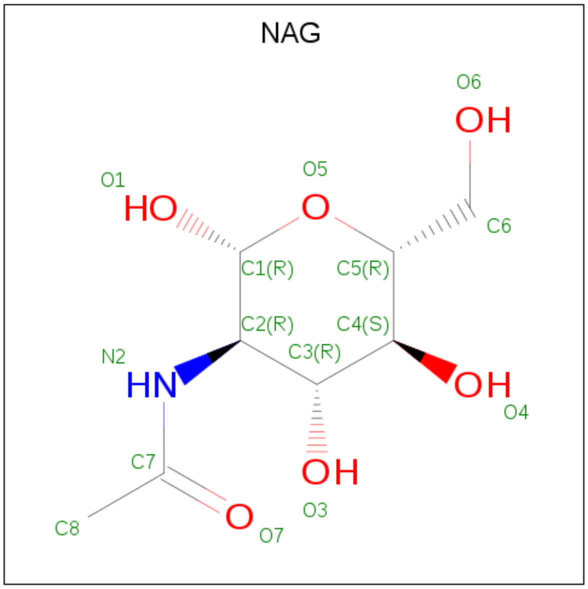

Mol	Chain	Residues	Atoms					ZeroOcc	AltConf
			Total	C	H	N	O		
4	A	1	28	8	14	1	5	0	0
			Total	C	H	N	O		
4	A	1	28	8	14	1	5	0	0
			Total	C	H	N	O		
4	A	1	28	8	14	1	5	0	0
			Total	C	H	N	O		
4	A	1	28	8	14	1	5	0	0
			Total	C	H	N	O		
4	A	1	28	8	14	1	5	0	0

- Molecule 5 is PHOSPHATE ION (three-letter code: PO4) (formula: O_4P).

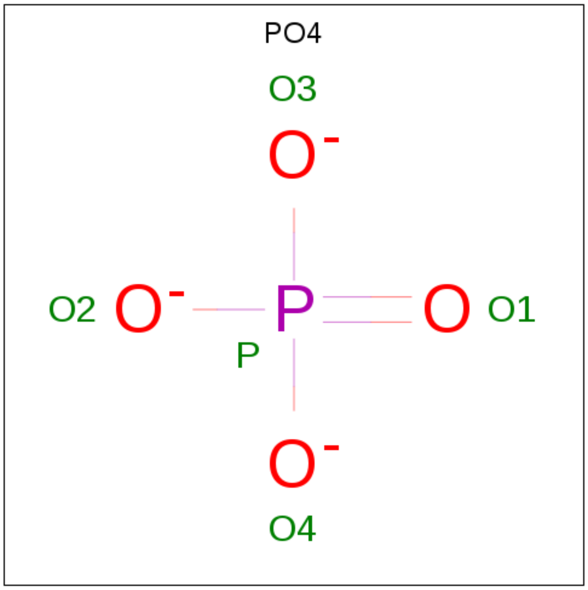

Mol	Chain	Residues	Atoms	ZeroOcc	AltConf
5	A	1	Total O P<br>5 4 1	0	0
5	A	1	Total O P<br>5 4 1	0	0
5	A	1	Total O P<br>5 4 1	0	0

- Molecule 6 is TRIETHYLENE GLYCOL (three-letter code: PGE) (formula: $C_6H_{14}O_4$).

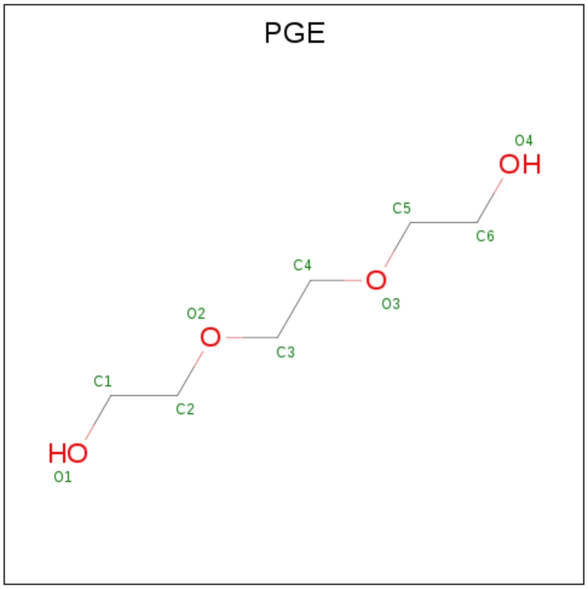

Mol	Chain	Residues	Atoms	ZeroOcc	AltConf
6	A	1	Total C H O<br>24 6 14 4	0	0

Continued on next page...

Continued from previous page...

Mol	Chain	Residues	Atoms	ZeroOcc	AltConf
6	A	1	Total C H O<br>24 6 14 4	0	0
6	A	1	Total C H O<br>24 6 14 4	0	0

- Molecule 7 is DI(HYDROXYETHYL)ETHER (three-letter code: PEG) (formula: $C_4H_{10}O_3$).

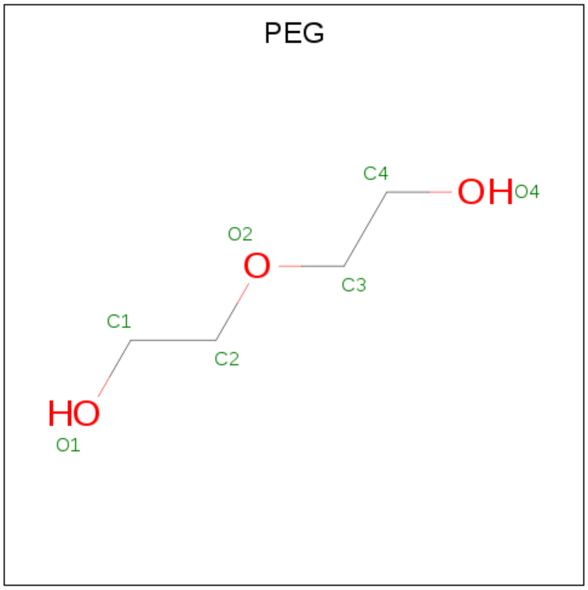

Mol	Chain	Residues	Atoms	ZeroOcc	AltConf
7	A	1	Total C H O<br>17 4 10 3	0	0
7	A	1	Total C H O<br>17 4 10 3	0	0
7	A	1	Total C H O<br>17 4 10 3	0	0
7	A	1	Total C H O<br>17 4 10 3	0	0

- Molecule 8 is 1,2-ETHANEDIOL (three-letter code: EDO) (formula: $C_2H_6O_2$).

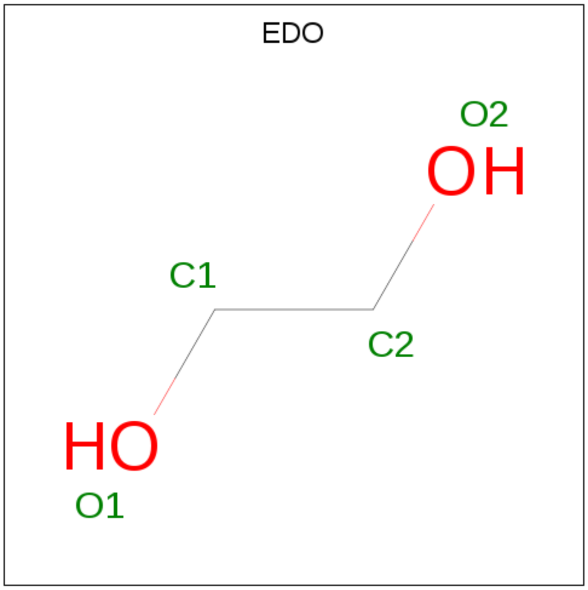

Mol	Chain	Residues	Atoms	ZeroOcc	AltConf
8	A	1	Total C H O<br>10 2 6 2	0	0
8	A	1	Total C H O<br>10 2 6 2	0	0
8	A	1	Total C H O<br>10 2 6 2	0	0
8	A	1	Total C H O<br>10 2 6 2	0	0
8	A	1	Total C H O<br>10 2 6 2	0	0
8	A	1	Total C H O<br>10 2 6 2	0	0
8	A	1	Total C H O<br>10 2 6 2	0	0
8	A	1	Total C H O<br>10 2 6 2	0	0

- Molecule 9 is water.

Mol	Chain	Residues	Atoms	ZeroOcc	AltConf
9	A	433	Total O<br>433 433	0	0

3 Residue-property plots (i)

These plots are drawn for all protein, RNA, DNA and oligosaccharide chains in the entry. The first graphic for a chain summarises the proportions of the various outlier classes displayed in the second graphic. The second graphic shows the sequence view annotated by issues in geometry and electron density. Residues are color-coded according to the number of geometric quality criteria for which they contain at least one outlier: green = 0, yellow = 1, orange = 2 and red = 3 or more. A red dot above a residue indicates a poor fit to the electron density (RSRZ > 2). Stretches of 2 or more consecutive residues without any outlier are shown as a green connector. Residues present in the sample, but not in the model, are shown in grey.

- Molecule 1: Purple acid phosphatase

Chain A: 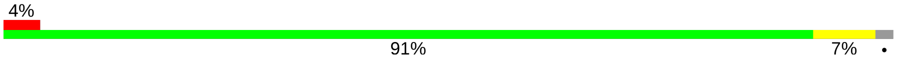

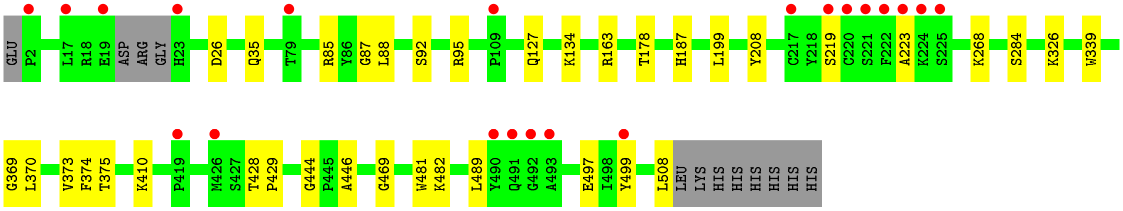

- Molecule 2: 2-acetamido-2-deoxy-beta-D-glucopyranose-(1-4)-2-acetamido-2-deoxy-beta-D-glucopyranose

Chain B: 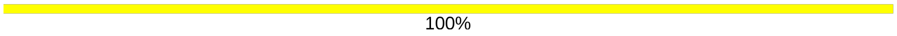

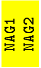

- Molecule 2: 2-acetamido-2-deoxy-beta-D-glucopyranose-(1-4)-2-acetamido-2-deoxy-beta-D-glucopyranose

Chain C: 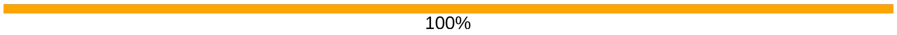

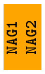

4 Data and refinement statistics

Property	Value	Source
Space group	H 3	Depositor
Cell constants<br>a, b, c, α, β, γ	126.73Å 126.73Å 107.04Å<br>90.00° 90.00° 120.00°	Depositor
Resolution (Å)	48.10 – 1.54<br>48.11 – 1.54	Depositor<br>EDS
% Data completeness<br>(in resolution range)	99.8 (48.10-1.54)<br>99.8 (48.11-1.54)	Depositor<br>EDS
R_{merge}	0.06	Depositor
R_{sym}	(Not available)	Depositor
$< I/\sigma(I) >$ [1]	1.66 (at 1.54Å)	Xtriage
Refinement program	PHENIX	Depositor
R, R_{free}	0.136 , 0.167<br>0.138 , 0.134	Depositor<br>DCC
R_{free} test set	4709 reflections (4.97%)	wwPDB-VP
Wilson B-factor (Å^2)	18.8	Xtriage
Anisotropy	0.131	Xtriage
Bulk solvent k_{sol}(e/Å^3), B_{sol}(Å^2)	0.38 , 53.1	EDS
L-test for twinning[2]	$< \|L\| > = 0.48$, $< L^2 > = 0.31$	Xtriage
Estimated twinning fraction	0.024 for h,-h-k,-l	Xtriage
F_o,F_c correlation	0.98	EDS
Total number of atoms	4915	wwPDB-VP
Average B, all atoms (Å^2)	28.0	wwPDB-VP

Xtriage's analysis on translational NCS is as follows: *The largest off-origin peak in the Patterson function is 3.82% of the height of the origin peak. No significant pseudotranslation is detected.*

[1]Intensities estimated from amplitudes.

[2]Theoretical values of $< |L| >$, $< L^2 >$ for acentric reflections are 0.5, 0.333 respectively for untwinned datasets, and 0.375, 0.2 for perfectly twinned datasets.

5 Model quality (i)

5.1 Standard geometry (i)

Bond lengths and bond angles in the following residue types are not validated in this section: PGE, NAG, PO4, EDO, FE, PEG

The Z score for a bond length (or angle) is the number of standard deviations the observed value is removed from the expected value. A bond length (or angle) with $|Z| > 5$ is considered an outlier worth inspection. RMSZ is the root-mean-square of all Z scores of the bond lengths (or angles).

Mol	Chain	Bond lengths		Bond angles	
		RMSZ	#|Z| >5	RMSZ	#|Z| >5
1	A	0.34	0/4161	0.52	0/5678

There are no bond length outliers.

There are no bond angle outliers.

There are no chirality outliers.

There are no planarity outliers.

5.2 Too-close contacts (i)

In the following table, the Non-H and H(model) columns list the number of non-hydrogen atoms and hydrogen atoms in the chain respectively. The H(added) column lists the number of hydrogen atoms added and optimized by MolProbity. The Clashes column lists the number of clashes within the asymmetric unit, whereas Symm-Clashes lists symmetry related clashes.

Mol	Chain	Non-H	H(model)	H(added)	Clashes	Symm-Clashes
1	A	3995	0	3749	34	0
2	B	28	27	25	0	0
2	C	28	27	25	2	0
3	A	2	0	0	0	0
4	A	70	70	65	1	0
5	A	15	0	0	2	0
6	A	30	42	42	4	0
7	A	28	40	40	3	0
8	A	32	48	47	8	0
9	A	433	0	0	7	0
All	All	4661	254	3993	38	0

The all-atom clashscore is defined as the number of clashes found per 1000 atoms (including hydrogen atoms). The all-atom clashscore for this structure is 5.

All (38) close contacts within the same asymmetric unit are listed below, sorted by their clash magnitude.

Atom-1	Atom-2	Interatomic distance (Å)	Clash overlap (Å)
1:A:219:SER:HA	1:A:223:ALA:HB2	1.56	0.87
1:A:88:LEU:HG	8:A:624:EDO:H11	1.61	0.82
1:A:444:GLY:HA3	6:A:617:PGE:H12	1.73	0.71
1:A:134:LYS:NZ	7:A:620:PEG:O1	2.16	0.71
1:A:127[B]:GLN:NE2	9:A:707:HOH:O	2.26	0.69
1:A:482:LYS:HG2	1:A:497:GLU:HG3	1.75	0.68
1:A:26[B]:ASP:OD2	1:A:208:TYR:OH	2.11	0.67
1:A:95:ARG:HD2	8:A:624:EDO:O1	1.97	0.63
1:A:26[A]:ASP:OD2	9:A:702:HOH:O	2.15	0.62
1:A:369:GLY:HA3	1:A:508:LEU:HD21	1.83	0.60
1:A:127[B]:GLN:HG2	9:A:704:HOH:O	2.02	0.59
1:A:326:LYS:HZ2	6:A:616:PGE:H3	1.67	0.59
1:A:469:GLY:HA3	1:A:481:TRP:CH2	2.37	0.58
1:A:87:GLY:HA2	8:A:624:EDO:H12	1.86	0.56
5:A:612:PO4:P	9:A:709:HOH:O	2.63	0.56
1:A:163:ARG:NH1	8:A:627:EDO:O2	2.36	0.51
1:A:428:THR:N	1:A:429:PRO:HD2	2.27	0.49
1:A:88:LEU:CG	8:A:624:EDO:H11	2.39	0.49
1:A:410:LYS:HE3	5:A:614:PO4:O1	2.13	0.48
1:A:428:THR:N	1:A:429:PRO:CD	2.79	0.46
2:C:1:NAG:O3	2:C:2:NAG:O5	2.31	0.46
1:A:26[B]:ASP:OD1	1:A:178:THR:HG22	2.16	0.46
1:A:199:LEU:HD11	1:A:374:PHE:HB3	1.99	0.45
1:A:187:HIS:NE2	1:A:489:LEU:HD23	2.31	0.45
1:A:85:ARG:NH1	9:A:701:HOH:O	2.09	0.44
1:A:284:SER:O	6:A:616:PGE:H4	2.16	0.44
1:A:446:ALA:HA	8:A:625:EDO:H21	2.00	0.44
1:A:163:ARG:H	8:A:627:EDO:H12	1.84	0.43
1:A:92:SER:HB3	9:A:711:HOH:O	2.18	0.43
4:A:611:NAG:C8	4:A:611:NAG:H3	2.48	0.43
1:A:339:TRP:O	1:A:375:THR:HA	2.18	0.42
1:A:370:LEU:HD21	1:A:373:VAL:CG2	2.50	0.42
1:A:268:LYS:HE2	9:A:1017:HOH:O	2.20	0.41
1:A:326:LYS:HZ2	6:A:616:PGE:H6	1.85	0.41
1:A:370:LEU:HD21	1:A:373:VAL:HG22	2.03	0.41
7:A:621:PEG:C1	2:C:2:NAG:O4	2.69	0.41
1:A:95:ARG:HE	7:A:618:PEG:H12	1.86	0.40
1:A:446:ALA:HA	8:A:625:EDO:C2	2.50	0.40

There are no symmetry-related clashes.

5.3 Torsion angles (i)

5.3.1 Protein backbone (i)

In the following table, the Percentiles column shows the percent Ramachandran outliers of the chain as a percentile score with respect to all X-ray entries followed by that with respect to entries of similar resolution.

The Analysed column shows the number of residues for which the backbone conformation was analysed, and the total number of residues.

Mol	Chain	Analysed	Favoured	Allowed	Outliers	Percentiles	
1	A	514/516 (100%)	498 (97%)	16 (3%)	0	100	100

There are no Ramachandran outliers to report.

5.3.2 Protein sidechains (i)

In the following table, the Percentiles column shows the percent sidechain outliers of the chain as a percentile score with respect to all X-ray entries followed by that with respect to entries of similar resolution.

The Analysed column shows the number of residues for which the sidechain conformation was analysed, and the total number of residues.

Mol	Chain	Analysed	Rotameric	Outliers	Percentiles	
1	A	424/425 (100%)	422 (100%)	2 (0%)	88	77

All (2) residues with a non-rotameric sidechain are listed below:

Mol	Chain	Res	Type
1	A	35	GLN
1	A	499	TYR

Some sidechains can be flipped to improve hydrogen bonding and reduce clashes. All (1) such sidechains are listed below:

Mol	Chain	Res	Type
1	A	191	ASN

5.3.3 RNA (i)

There are no RNA molecules in this entry.

5.4 Non-standard residues in protein, DNA, RNA chains (i)

There are no non-standard protein/DNA/RNA residues in this entry.

5.5 Carbohydrates (i)

4 monosaccharides are modelled in this entry.

In the following table, the Counts columns list the number of bonds (or angles) for which Mogul statistics could be retrieved, the number of bonds (or angles) that are observed in the model and the number of bonds (or angles) that are defined in the Chemical Component Dictionary. The Link column lists molecule types, if any, to which the group is linked. The Z score for a bond length (or angle) is the number of standard deviations the observed value is removed from the expected value. A bond length (or angle) with $|Z| > 2$ is considered an outlier worth inspection. RMSZ is the root-mean-square of all Z scores of the bond lengths (or angles).

Mol	Type	Chain	Res	Link	Bond lengths			Bond angles		
					Counts	RMSZ	$\#\|Z\| > 2$	Counts	RMSZ	$\#\|Z\| > 2$
2	NAG	B	1	1,2	14,14,15	1.66	2 (14%)	17,19,21	1.59	4 (23%)
2	NAG	B	2	2	14,14,15	1.94	4 (28%)	17,19,21	1.25	2 (11%)
2	NAG	C	1	1,2	14,14,15	1.62	2 (14%)	17,19,21	1.03	0
2	NAG	C	2	2	14,14,15	1.94	4 (28%)	17,19,21	1.32	2 (11%)

In the following table, the Chirals column lists the number of chiral outliers, the number of chiral centers analysed, the number of these observed in the model and the number defined in the Chemical Component Dictionary. Similar counts are reported in the Torsion and Rings columns. '-' means no outliers of that kind were identified.

Mol	Type	Chain	Res	Link	Chirals	Torsions	Rings
2	NAG	B	1	1,2	-	2/6/23/26	0/1/1/1
2	NAG	B	2	2	-	0/6/23/26	0/1/1/1
2	NAG	C	1	1,2	-	2/6/23/26	0/1/1/1
2	NAG	C	2	2	-	0/6/23/26	0/1/1/1

All (12) bond length outliers are listed below:

Mol	Chain	Res	Type	Atoms	Z	Observed(Å)	Ideal(Å)
2	C	2	NAG	O5-C1	4.48	1.50	1.43
2	B	2	NAG	O5-C1	4.39	1.50	1.43
2	B	2	NAG	C7-N2	3.51	1.46	1.34
2	C	2	NAG	C7-N2	3.47	1.46	1.34
2	B	1	NAG	O5-C1	3.42	1.49	1.43
2	C	1	NAG	O5-C1	3.34	1.49	1.43

Continued on next page...

Continued from previous page...

Mol	Chain	Res	Type	Atoms	Z	Observed(Å)	Ideal(Å)
2	B	1	NAG	C7-N2	3.28	1.45	1.34
2	C	1	NAG	C7-N2	3.12	1.45	1.34
2	C	2	NAG	C2-N2	2.33	1.50	1.46
2	B	2	NAG	C2-N2	2.30	1.50	1.46
2	B	2	NAG	O5-C5	2.11	1.47	1.43
2	C	2	NAG	O5-C5	2.03	1.47	1.43

All (8) bond angle outliers are listed below:

Mol	Chain	Res	Type	Atoms	Z	Observed(^{o})	Ideal(^{o})
2	B	2	NAG	C1-C2-N2	-3.59	104.35	110.49
2	B	1	NAG	C4-C3-C2	2.91	115.29	111.02
2	B	1	NAG	O4-C4-C5	-2.88	102.15	109.30
2	B	1	NAG	O5-C1-C2	-2.41	107.48	111.29
2	C	2	NAG	O5-C5-C6	2.36	110.90	107.20
2	B	2	NAG	C8-C7-N2	2.23	119.88	116.10
2	B	1	NAG	C2-N2-C7	-2.16	119.83	122.90
2	C	2	NAG	C8-C7-N2	2.03	119.53	116.10

There are no chirality outliers.

All (4) torsion outliers are listed below:

Mol	Chain	Res	Type	Atoms
2	B	1	NAG	C4-C5-C6-O6
2	C	1	NAG	C4-C5-C6-O6
2	C	1	NAG	O5-C5-C6-O6
2	B	1	NAG	O5-C5-C6-O6

There are no ring outliers.

2 monomers are involved in 2 short contacts:

Mol	Chain	Res	Type	Clashes	Symm-Clashes
2	C	1	NAG	1	0
2	C	2	NAG	2	0

The following is a two-dimensional graphical depiction of Mogul quality analysis of bond lengths, bond angles, torsion angles, and ring geometry for oligosaccharide.

Oligosaccharide Chain B

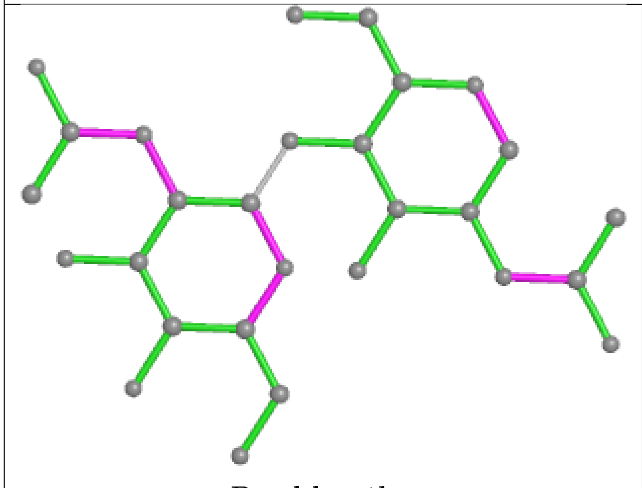

Bond lengths

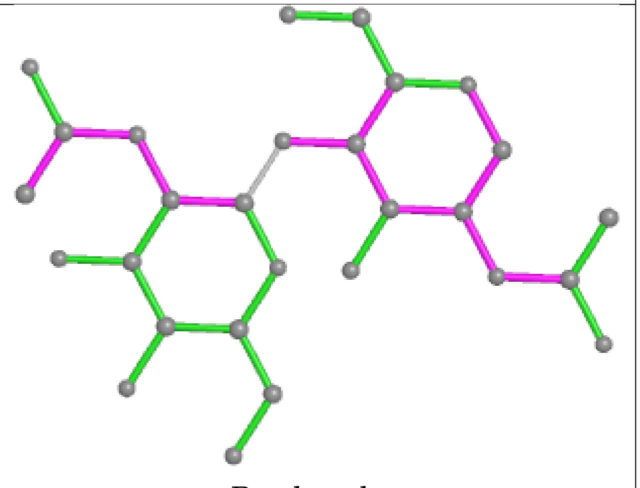

Bond angles

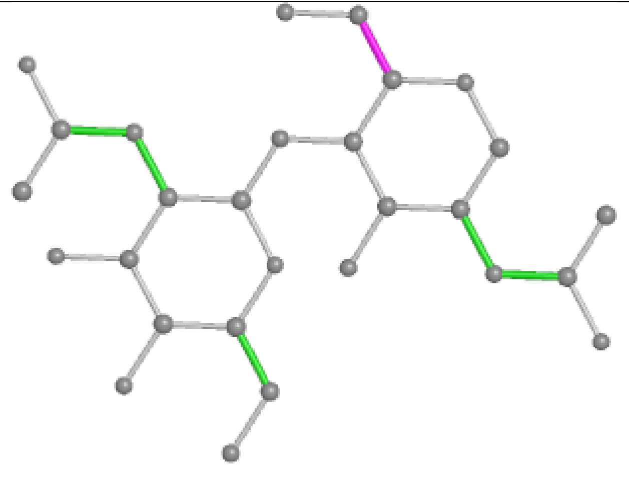

Torsions

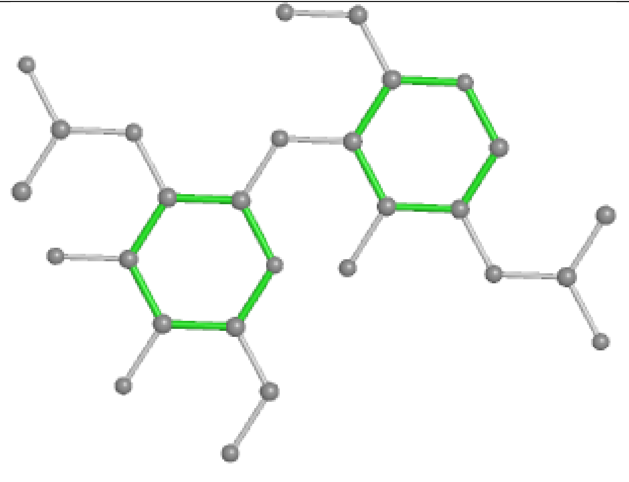

Rings

Oligosaccharide Chain C

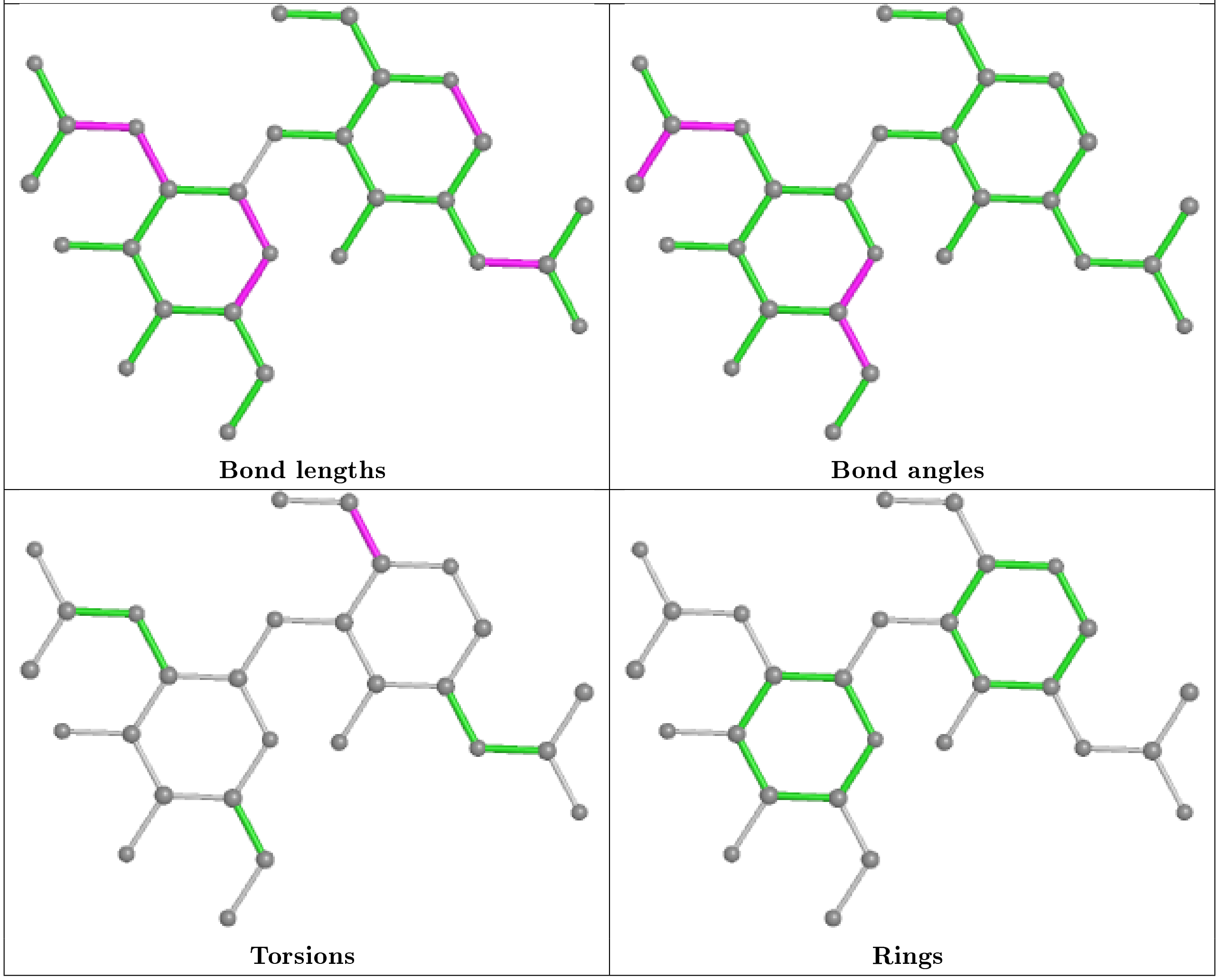

5.6 Ligand geometry (i)

Of 25 ligands modelled in this entry, 2 are monoatomic - leaving 23 for Mogul analysis.

In the following table, the Counts columns list the number of bonds (or angles) for which Mogul statistics could be retrieved, the number of bonds (or angles) that are observed in the model and the number of bonds (or angles) that are defined in the Chemical Component Dictionary. The Link column lists molecule types, if any, to which the group is linked. The Z score for a bond length (or angle) is the number of standard deviations the observed value is removed from the expected value. A bond length (or angle) with $|Z| > 2$ is considered an outlier worth inspection. RMSZ is the root-mean-square of all Z scores of the bond lengths (or angles).

Mol	Type	Chain	Res	Link	Bond lengths			Bond angles		
					Counts	RMSZ	$\#\|Z\| > 2$	Counts	RMSZ	$\#\|Z\| > 2$
4	NAG	A	608	1	14,14,15	2.10	5 (35%)	17,19,21	1.70	3 (17%)
8	EDO	A	628	-	3,3,3	0.47	0	2,2,2	0.26	0
4	NAG	A	611	1	14,14,15	1.90	3 (21%)	17,19,21	2.12	5 (29%)

Mol	Type	Chain	Res	Link	Bond lengths			Bond angles		
					Counts	RMSZ	#|Z| > 2	Counts	RMSZ	#|Z| > 2
7	PEG	A	620	-	6,6,6	0.50	0	5,5,5	0.36	0
4	NAG	A	607	1	14,14,15	1.86	2 (14%)	17,19,21	1.35	4 (23%)
8	EDO	A	622	-	3,3,3	0.49	0	2,2,2	0.34	0
8	EDO	A	623	-	3,3,3	0.47	0	2,2,2	0.33	0
5	PO4	A	612	3	4,4,4	0.86	0	6,6,6	0.76	0
4	NAG	A	609	1	14,14,15	1.91	3 (21%)	17,19,21	1.07	1 (5%)
7	PEG	A	621	-	6,6,6	0.48	0	5,5,5	0.39	0
8	EDO	A	625	-	3,3,3	0.47	0	2,2,2	0.24	0
4	NAG	A	610	1	14,14,15	1.86	3 (21%)	17,19,21	1.08	2 (11%)
8	EDO	A	627	-	3,3,3	0.87	0	2,2,2	0.68	0
7	PEG	A	619	-	6,6,6	0.47	0	5,5,5	0.43	0
5	PO4	A	613	-	4,4,4	0.87	0	6,6,6	0.45	0
5	PO4	A	614	-	4,4,4	0.97	0	6,6,6	0.62	0
6	PGE	A	615	-	9,9,9	0.51	0	8,8,8	0.31	0
8	EDO	A	626	-	3,3,3	0.47	0	2,2,2	0.28	0
8	EDO	A	629	-	3,3,3	0.45	0	2,2,2	0.34	0
6	PGE	A	617	-	9,9,9	0.50	0	8,8,8	0.56	0
7	PEG	A	618	-	6,6,6	0.46	0	5,5,5	0.53	0
8	EDO	A	624	-	3,3,3	0.47	0	2,2,2	0.40	0
6	PGE	A	616	-	9,9,9	0.50	0	8,8,8	0.70	0

In the following table, the Chirals column lists the number of chiral outliers, the number of chiral centers analysed, the number of these observed in the model and the number defined in the Chemical Component Dictionary. Similar counts are reported in the Torsion and Rings columns. '-' means no outliers of that kind were identified.

Mol	Type	Chain	Res	Link	Chirals	Torsions	Rings
4	NAG	A	607	1	-	2/6/23/26	0/1/1/1
6	PGE	A	615	-	-	0/7/7/7	-
8	EDO	A	622	-	-	1/1/1/1	-
4	NAG	A	608	1	-	0/6/23/26	0/1/1/1
4	NAG	A	610	1	-	0/6/23/26	0/1/1/1
7	PEG	A	619	-	-	1/4/4/4	-
8	EDO	A	628	-	-	0/1/1/1	-
8	EDO	A	623	-	-	0/1/1/1	-
7	PEG	A	620	-	-	0/4/4/4	-
8	EDO	A	629	-	-	1/1/1/1	-
4	NAG	A	609	1	-	0/6/23/26	0/1/1/1
7	PEG	A	621	-	-	1/4/4/4	-
6	PGE	A	617	-	-	7/7/7/7	-
7	PEG	A	618	-	-	4/4/4/4	-

Continued on next page...

Continued from previous page...

Mol	Type	Chain	Res	Link	Chirals	Torsions	Rings
4	NAG	A	611	1	-	3/6/23/26	0/1/1/1
8	EDO	A	625	-	-	1/1/1/1	-
8	EDO	A	624	-	-	0/1/1/1	-
8	EDO	A	626	-	-	0/1/1/1	-
8	EDO	A	627	-	-	0/1/1/1	-
6	PGE	A	616	-	-	2/7/7/7	-

All (16) bond length outliers are listed below:

Mol	Chain	Res	Type	Atoms	Z	Observed(Å)	Ideal(Å)
4	A	608	NAG	O5-C1	4.89	1.51	1.43
4	A	607	NAG	O5-C1	4.36	1.50	1.43
4	A	609	NAG	O5-C1	4.25	1.50	1.43
4	A	610	NAG	O5-C1	4.12	1.50	1.43
4	A	611	NAG	O5-C1	4.02	1.50	1.43
4	A	609	NAG	C7-N2	3.57	1.46	1.34
4	A	611	NAG	C7-N2	3.52	1.46	1.34
4	A	610	NAG	C7-N2	3.48	1.46	1.34
4	A	608	NAG	C7-N2	3.45	1.46	1.34
4	A	607	NAG	C7-N2	3.32	1.45	1.34
4	A	608	NAG	O5-C5	2.78	1.49	1.43
4	A	611	NAG	C2-N2	2.59	1.50	1.46
4	A	608	NAG	C3-C2	-2.30	1.47	1.52
4	A	609	NAG	C2-N2	2.26	1.50	1.46
4	A	608	NAG	C2-N2	2.06	1.49	1.46
4	A	610	NAG	C2-N2	2.05	1.49	1.46

All (15) bond angle outliers are listed below:

Mol	Chain	Res	Type	Atoms	Z	Observed(°)	Ideal(°)
4	A	611	NAG	C8-C7-N2	5.17	124.85	116.10
4	A	611	NAG	C1-C2-N2	-4.94	102.06	110.49
4	A	608	NAG	C1-C2-N2	-4.16	103.38	110.49
4	A	608	NAG	O5-C1-C2	3.62	117.00	111.29
4	A	611	NAG	C2-N2-C7	3.27	127.56	122.90
4	A	611	NAG	O7-C7-N2	-2.77	116.86	121.95
4	A	608	NAG	O5-C5-C6	2.77	111.54	107.20
4	A	610	NAG	C2-N2-C7	-2.66	119.11	122.90
4	A	607	NAG	C1-O5-C5	-2.59	108.68	112.19
4	A	609	NAG	C8-C7-N2	2.48	120.29	116.10
4	A	607	NAG	C8-C7-N2	2.40	120.16	116.10
4	A	607	NAG	C1-C2-N2	-2.39	106.40	110.49

Continued on next page...

Continued from previous page...

Mol	Chain	Res	Type	Atoms	Z	Observed(^{o})	Ideal(^{o})
4	A	610	NAG	C8-C7-N2	2.35	120.08	116.10
4	A	607	NAG	C2-N2-C7	-2.29	119.64	122.90
4	A	611	NAG	O7-C7-C8	-2.04	118.28	122.06

There are no chirality outliers.

All (23) torsion outliers are listed below:

Mol	Chain	Res	Type	Atoms
4	A	611	NAG	C3-C2-N2-C7
6	A	616	PGE	C1-C2-O2-C3
4	A	607	NAG	O5-C5-C6-O6
4	A	607	NAG	C4-C5-C6-O6
4	A	611	NAG	C8-C7-N2-C2
4	A	611	NAG	O7-C7-N2-C2
6	A	617	PGE	O2-C3-C4-O3
7	A	621	PEG	O1-C1-C2-O2
6	A	617	PGE	O1-C1-C2-O2
6	A	616	PGE	O2-C3-C4-O3
6	A	617	PGE	O3-C5-C6-O4
7	A	618	PEG	O1-C1-C2-O2
7	A	618	PEG	O2-C3-C4-O4
8	A	625	EDO	O1-C1-C2-O2
6	A	617	PGE	C3-C4-O3-C5
6	A	617	PGE	C4-C3-O2-C2
7	A	618	PEG	C1-C2-O2-C3
6	A	617	PGE	C1-C2-O2-C3
6	A	617	PGE	C6-C5-O3-C4
8	A	622	EDO	O1-C1-C2-O2
7	A	619	PEG	C1-C2-O2-C3
8	A	629	EDO	O1-C1-C2-O2
7	A	618	PEG	C4-C3-O2-C2

There are no ring outliers.

11 monomers are involved in 18 short contacts:

Mol	Chain	Res	Type	Clashes	Symm-Clashes
4	A	611	NAG	1	0
7	A	620	PEG	1	0
5	A	612	PO4	1	0
7	A	621	PEG	1	0
8	A	625	EDO	2	0

Continued on next page...

Continued from previous page...

Mol	Chain	Res	Type	Clashes	Symm-Clashes
8	A	627	EDO	2	0
5	A	614	PO4	1	0
6	A	617	PGE	1	0
7	A	618	PEG	1	0
8	A	624	EDO	4	0
6	A	616	PGE	3	0

5.7 Other polymers (i)

There are no such residues in this entry.

5.8 Polymer linkage issues (i)

There are no chain breaks in this entry.

6 Fit of model and data (i)

6.1 Protein, DNA and RNA chains (i)

In the following table, the column labelled '#RSRZ> 2' contains the number (and percentage) of RSRZ outliers, followed by percent RSRZ outliers for the chain as percentile scores relative to all X-ray entries and entries of similar resolution. The OWAB column contains the minimum, median, 95th percentile and maximum values of the occupancy-weighted average B-factor per residue. The column labelled 'Q< 0.9' lists the number of (and percentage) of residues with an average occupancy less than 0.9.

Mol	Chain	Analysed	<RSRZ>	#RSRZ>2			OWAB(Å^2)	Q<0.9
1	A	504/516 (97%)	0.07	21 (4%)	36	41	13, 21, 43, 68	0

All (21) RSRZ outliers are listed below:

Mol	Chain	Res	Type	RSRZ
1	A	223	ALA	7.1
1	A	220	CYS	6.5
1	A	224	LYS	5.8
1	A	222	PHE	5.4
1	A	493	ALA	5.3
1	A	2	PRO	5.2
1	A	490	TYR	4.9
1	A	17	LEU	4.2
1	A	492	GLY	4.0
1	A	225	SER	3.6
1	A	19	GLU	3.5
1	A	217	CYS	3.4
1	A	491	GLN	3.3
1	A	221	SER	3.1
1	A	79	THR	2.5
1	A	426	MET	2.5
1	A	23	HIS	2.2
1	A	219	SER	2.1
1	A	109	PRO	2.1
1	A	499	TYR	2.0
1	A	419	PRO	2.0

6.2 Non-standard residues in protein, DNA, RNA chains (i)

There are no non-standard protein/DNA/RNA residues in this entry.

6.3 Carbohydrates (i)

In the following table, the Atoms column lists the number of modelled atoms in the group and the number defined in the chemical component dictionary. The B-factors column lists the minimum, median, 95th percentile and maximum values of B factors of atoms in the group. The column labelled 'Q< 0.9' lists the number of atoms with occupancy less than 0.9.

Mol	Type	Chain	Res	Atoms	RSCC	RSR	B-factors(Å^2)	Q<0.9
2	NAG	B	2	14/15	0.71	0.36	53,63,72,75	28
2	NAG	C	2	14/15	0.76	0.17	42,51,62,65	28
2	NAG	B	1	14/15	0.93	0.16	26,36,45,46	0
2	NAG	C	1	14/15	0.96	0.09	22,28,36,39	0

The following is a graphical depiction of the model fit to experimental electron density for oligosaccharide. Each fit is shown from different orientation to approximate a three-dimensional view.

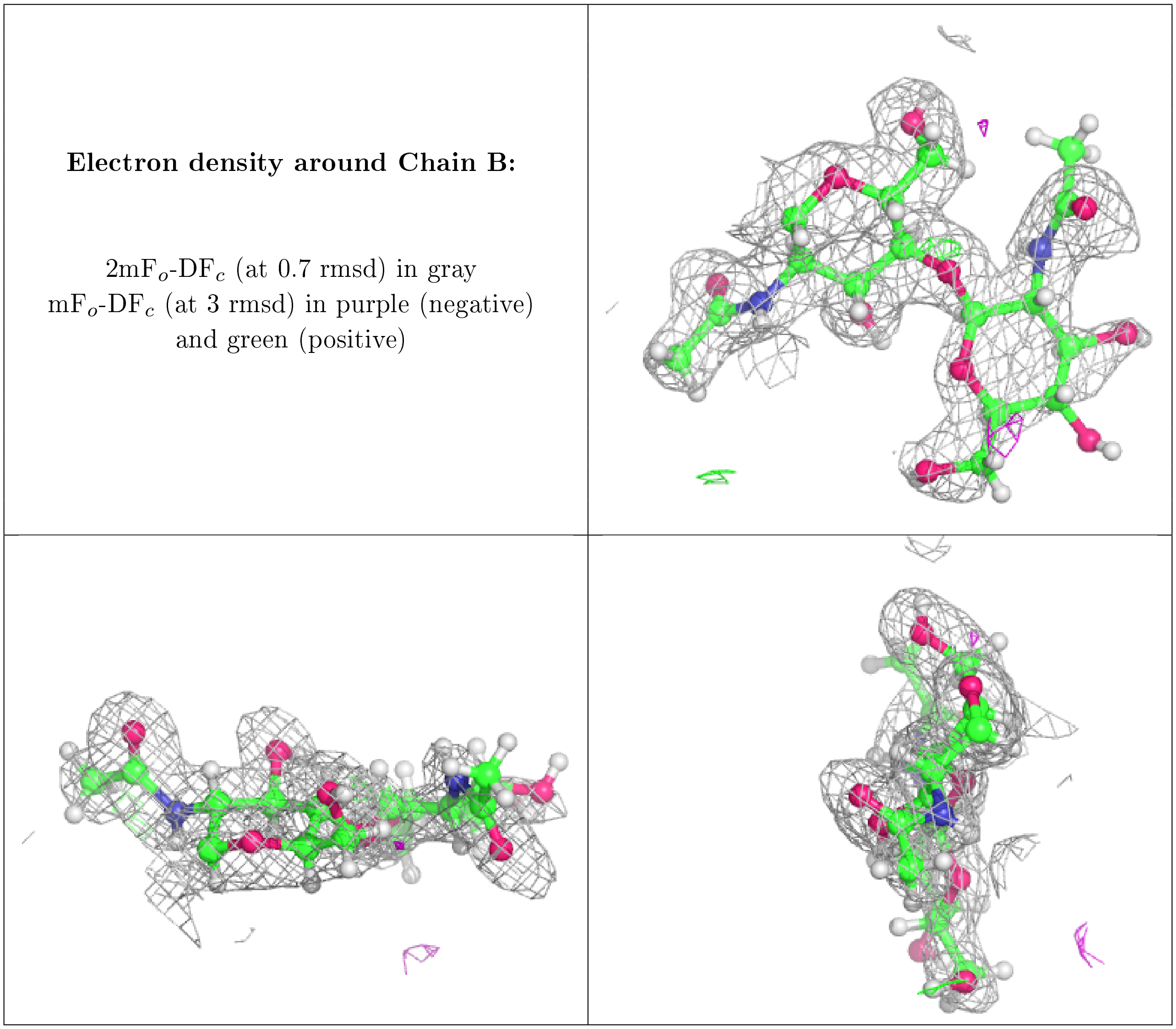

Electron density around Chain C:

$2mF_o$-DF_c (at 0.7 rmsd) in gray
mF_o-DF_c (at 3 rmsd) in purple (negative)
and green (positive)

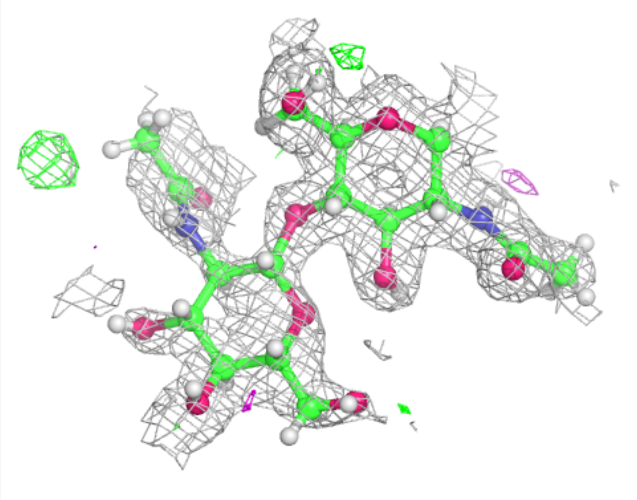

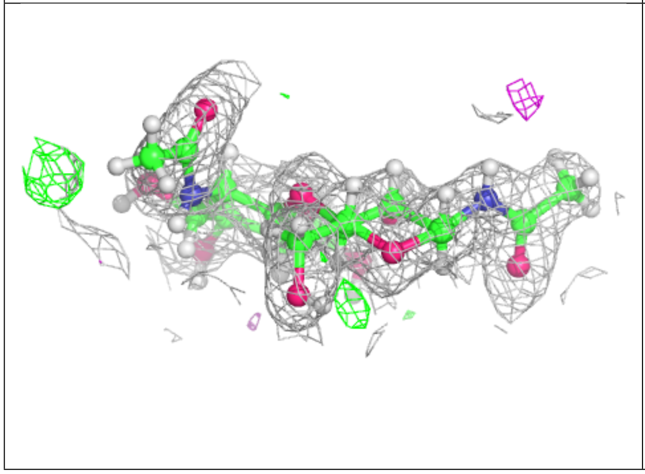

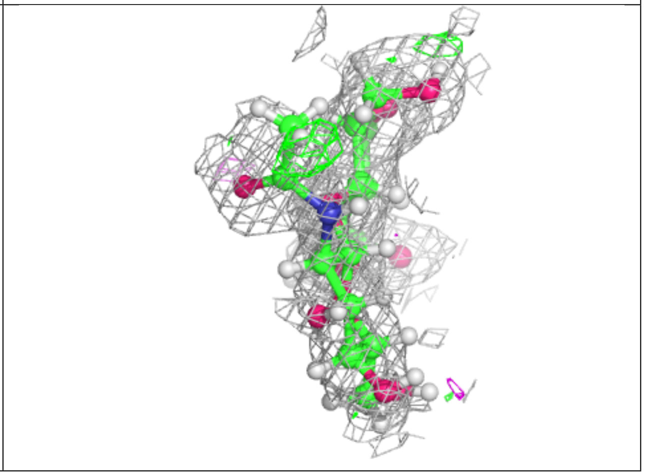

6.4 Ligands (i)

In the following table, the Atoms column lists the number of modelled atoms in the group and the number defined in the chemical component dictionary. The B-factors column lists the minimum, median, 95th percentile and maximum values of B factors of atoms in the group. The column labelled 'Q< 0.9' lists the number of atoms with occupancy less than 0.9.

Mol	Type	Chain	Res	Atoms	RSCC	RSR	B-factors(Å^2)	Q<0.9
8	EDO	A	625	4/4	0.51	0.22	52,63,70,72	0
4	NAG	A	609	14/15	0.60	0.34	48,63,75,77	28
7	PEG	A	619	7/7	0.61	0.23	55,66,74,75	0
4	NAG	A	608	14/15	0.65	0.24	53,66,80,80	28
7	PEG	A	621	7/7	0.65	0.28	60,72,75,76	0
8	EDO	A	626	4/4	0.67	0.18	66,79,83,83	0
6	PGE	A	616	10/10	0.71	0.14	61,73,76,77	0
7	PEG	A	620	7/7	0.74	0.15	66,79,79,80	0
8	EDO	A	623	4/4	0.77	0.14	65,78,79,80	0
6	PGE	A	617	10/10	0.81	0.20	57,70,72,74	0
4	NAG	A	611	14/15	0.82	0.21	35,51,62,62	28

Continued on next page...

Continued from previous page...

Mol	Type	Chain	Res	Atoms	RSCC	RSR	B-factors(Å^2)	Q<0.9
4	NAG	A	607	14/15	0.85	0.14	41,51,62,65	28
7	PEG	A	618	7/7	0.87	0.14	54,65,75,77	0
8	EDO	A	628	4/4	0.87	0.09	35,42,46,51	0
8	EDO	A	629	4/4	0.88	0.28	53,64,64,65	0
8	EDO	A	622	4/4	0.89	0.15	59,71,72,73	0
4	NAG	A	610	14/15	0.89	0.25	39,48,63,63	0
6	PGE	A	615	10/10	0.91	0.10	40,50,63,65	0
5	PO4	A	613	5/5	0.93	0.14	66,66,67,67	5
8	EDO	A	624	4/4	0.95	0.16	48,58,61,66	0
5	PO4	A	612	5/5	0.95	0.13	31,32,35,36	5
8	EDO	A	627	4/4	0.97	0.12	14,32,39,46	0
5	PO4	A	614	5/5	0.98	0.10	36,36,40,41	0
3	FE	A	601	1/1	1.00	0.05	20,20,20,20	1
3	FE	A	602	1/1	1.00	0.07	16,16,16,16	0

6.5 Other polymers (i)

There are no such residues in this entry.

Full wwPDB X-ray Structure Validation Report (i)

Aug 8, 2020 – 06:40 AM BST

PDB ID	:	6GJ2
Title	:	PURPLE ACID PHYTASE FROM WHEAT ISOFORM B2 - COMPLEX WITH INOSITOL HEXASULPHATE
Authors	:	Faba-Rodriguez, R.; Brearley, C.A.; Hemmings, A.M.
Deposited on	:	2018-05-15
Resolution	:	1.68 Å(reported)

This is a Full wwPDB X-ray Structure Validation Report for a publicly released PDB entry.

We welcome your comments at *validation@mail.wwpdb.org*
A user guide is available at
https://www.wwpdb.org/validation/2017/XrayValidationReportHelp
with specific help available everywhere you see the (i) symbol.

The following versions of software and data (see references (i)) were used in the production of this report:

MolProbity	:	4.02b-467
Mogul	:	1.8.5 (274361), CSD as541be (2020)
Xtriage (Phenix)	:	1.13
EDS	:	2.13.1
buster-report	:	1.1.7 (2018)
Percentile statistics	:	20191225.v01 (using entries in the PDB archive December 25th 2019)
Refmac	:	5.8.0158
CCP4	:	7.0.044 (Gargrove)
Ideal geometry (proteins)	:	Engh & Huber (2001)
Ideal geometry (DNA, RNA)	:	Parkinson et al. (1996)
Validation Pipeline (wwPDB-VP)	:	2.13.1

1 Overall quality at a glance (i)

The following experimental techniques were used to determine the structure:
X-RAY DIFFRACTION

The reported resolution of this entry is 1.68 Å.

Percentile scores (ranging between 0-100) for global validation metrics of the entry are shown in the following graphic. The table shows the number of entries on which the scores are based.

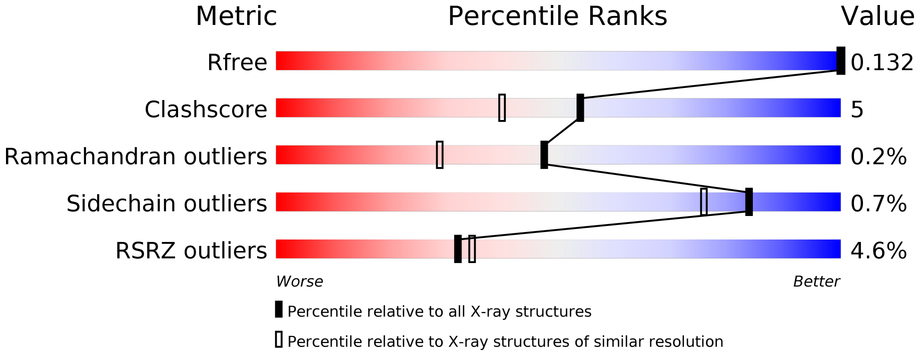

Metric	Whole archive (#Entries)	Similar resolution (#Entries, resolution range(Å))
R_{free}	130704	6780 (1.70-1.66)
Clashscore	141614	7310 (1.70-1.66)
Ramachandran outliers	138981	7173 (1.70-1.66)
Sidechain outliers	138945	7172 (1.70-1.66)
RSRZ outliers	127900	6661 (1.70-1.66)

The table below summarises the geometric issues observed across the polymeric chains and their fit to the electron density. The red, orange, yellow and green segments on the lower bar indicate the fraction of residues that contain outliers for >=3, 2, 1 and 0 types of geometric quality criteria respectively. A grey segment represents the fraction of residues that are not modelled. The numeric value for each fraction is indicated below the corresponding segment, with a dot representing fractions <=5% The upper red bar (where present) indicates the fraction of residues that have poor fit to the electron density. The numeric value is given above the bar.

Mol	Chain	Length	Quality of chain
1	A	516	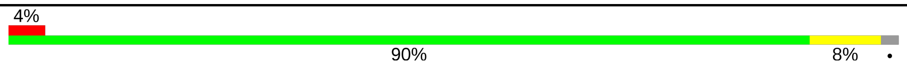
2	B	2	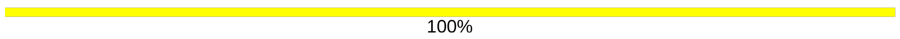

The following table lists non-polymeric compounds, carbohydrate monomers and non-standard residues in protein, DNA, RNA chains that are outliers for geometric or electron-density-fit criteria:

Mol	Type	Chain	Res	Chirality	Geometry	Clashes	Electron density
9	EDO	A	622	-	-	X	-

2 Entry composition (i)

There are 11 unique types of molecules in this entry. The entry contains 4748 atoms, of which 251 are hydrogens and 0 are deuteriums.

In the tables below, the ZeroOcc column contains the number of atoms modelled with zero occupancy, the AltConf column contains the number of residues with at least one atom in alternate conformation and the Trace column contains the number of residues modelled with at most 2 atoms.

- Molecule 1 is a protein called Purple acid phosphatase.

Mol	Chain	Residues	Atoms					ZeroOcc	AltConf	Trace
			Total	C	N	O	S			
1	A	504	3966	2522	670	750	24	0	7	0

There are 6 discrepancies between the modelled and reference sequences:

Chain	Residue	Modelled	Actual	Comment	Reference
A	511	HIS	-	expression tag	UNP C4PKL0
A	512	HIS	-	expression tag	UNP C4PKL0
A	513	HIS	-	expression tag	UNP C4PKL0
A	514	HIS	-	expression tag	UNP C4PKL0
A	515	HIS	-	expression tag	UNP C4PKL0
A	516	HIS	-	expression tag	UNP C4PKL0

- Molecule 2 is an oligosaccharide called 2-acetamido-2-deoxy-beta-D-glucopyranose-(1-4)-2-acetamido-2-deoxy-beta-D-glucopyranose.

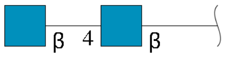

Mol	Chain	Residues	Atoms					ZeroOcc	AltConf	Trace
			Total	C	H	N	O			
2	B	2	55	16	27	2	10	0	0	0

- Molecule 3 is FE (III) ION (three-letter code: FE) (formula: Fe).

Mol	Chain	Residues	Atoms		ZeroOcc	AltConf
			Total	Fe		
3	A	2	2	2	0	0

- Molecule 4 is 2-acetamido-2-deoxy-beta-D-glucopyranose (three-letter code: NAG) (formula: $C_8H_{15}NO_6$).

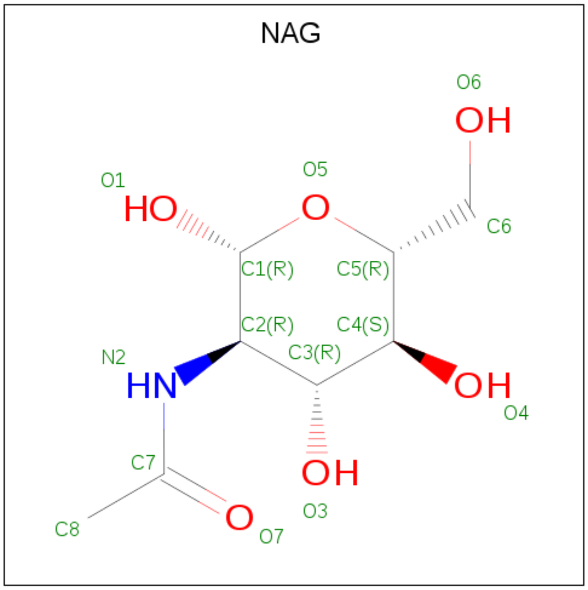

Mol	Chain	Residues	Atoms	ZeroOcc	AltConf
4	A	1	Total C H N O<br>28 8 14 1 5	0	0
4	A	1	Total C H N O<br>28 8 14 1 5	0	0
4	A	1	Total C H N O<br>28 8 14 1 5	0	0
4	A	1	Total C H N O<br>28 8 14 1 5	0	0
4	A	1	Total C H N O<br>28 8 14 1 5	0	0

- Molecule 5 is DI(HYDROXYETHYL)ETHER (three-letter code: PEG) (formula: $C_4H_{10}O_3$).

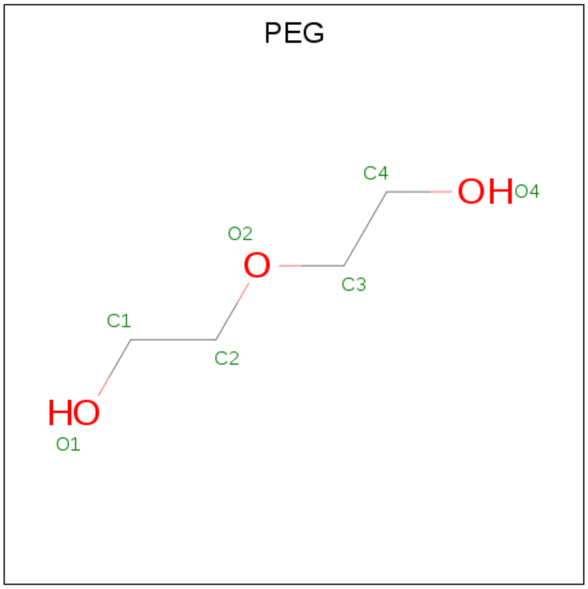

Mol	Chain	Residues	Atoms				ZeroOcc	AltConf
5	A	1	Total 17	C 4	H 10	O 3	0	0
5	A	1	Total 17	C 4	H 10	O 3	0	0
5	A	1	Total 17	C 4	H 10	O 3	0	0
5	A	1	Total 17	C 4	H 10	O 3	0	0
5	A	1	Total 17	C 4	H 10	O 3	0	0

- Molecule 6 is TRIETHYLENE GLYCOL (three-letter code: PGE) (formula: $C_6H_{14}O_4$).

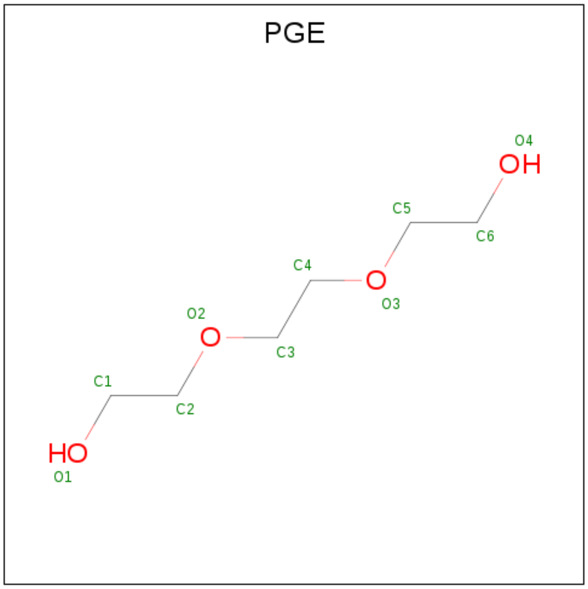

Mol	Chain	Residues	Atoms				ZeroOcc	AltConf
6	A	1	Total 24	C 6	H 14	O 4	0	0
6	A	1	Total 24	C 6	H 14	O 4	0	0
6	A	1	Total 24	C 6	H 14	O 4	0	0
6	A	1	Total 24	C 6	H 14	O 4	0	0

- Molecule 7 is PHOSPHATE ION (three-letter code: PO4) (formula: O_4P).

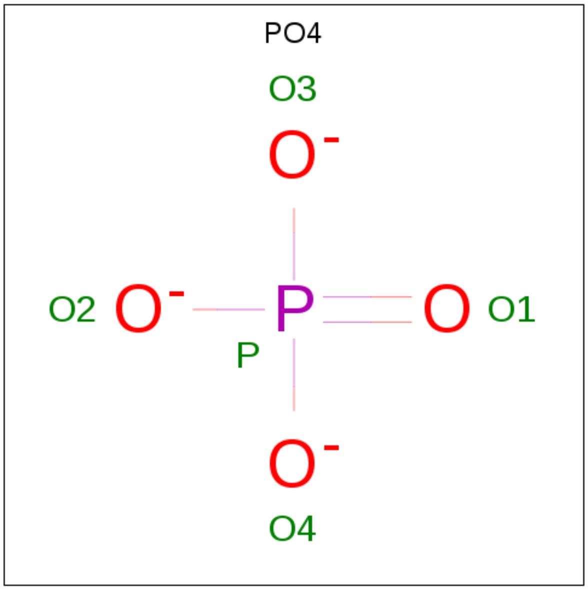

Mol	Chain	Residues	Atoms			ZeroOcc	AltConf
			Total	O	P		
7	A	1	5	4	1	0	0

- Molecule 8 is TETRAETHYLENE GLYCOL (three-letter code: PG4) (formula: $C_8H_{18}O_5$).

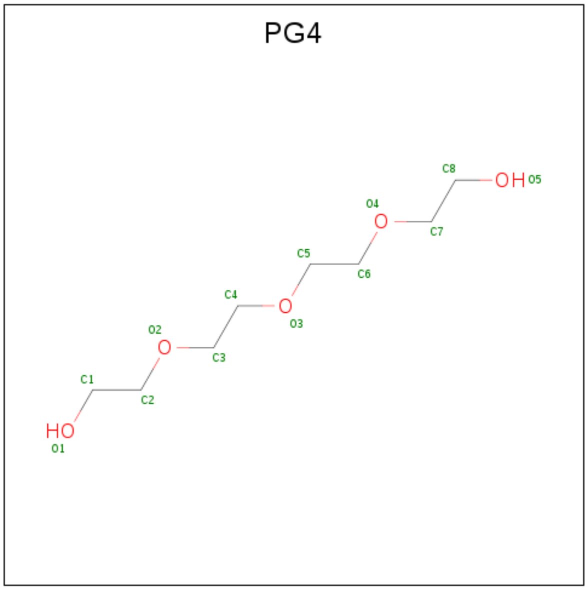

Mol	Chain	Residues	Atoms				ZeroOcc	AltConf
			Total	C	H	O		
8	A	1	31	8	18	5	0	0

- Molecule 9 is 1,2-ETHANEDIOL (three-letter code: EDO) (formula: $C_2H_6O_2$).

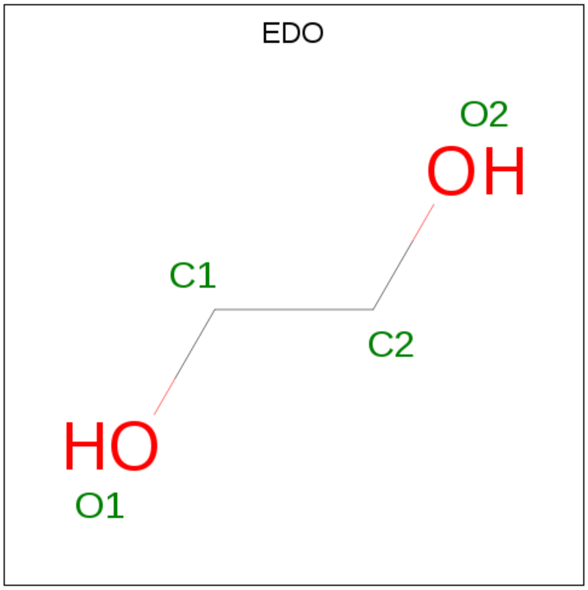

Mol	Chain	Residues	Atoms	ZeroOcc	AltConf
9	A	1	Total C H O<br>10 2 6 2	0	0
9	A	1	Total C H O<br>10 2 6 2	0	0
9	A	1	Total C H O<br>10 2 6 2	0	0
9	A	1	Total C H O<br>10 2 6 2	0	0

- Molecule 10 is D-MYO-INOSITOL-HEXASULPHATE (three-letter code: IHS) (formula: $C_6H_{12}O_{24}S_6$).

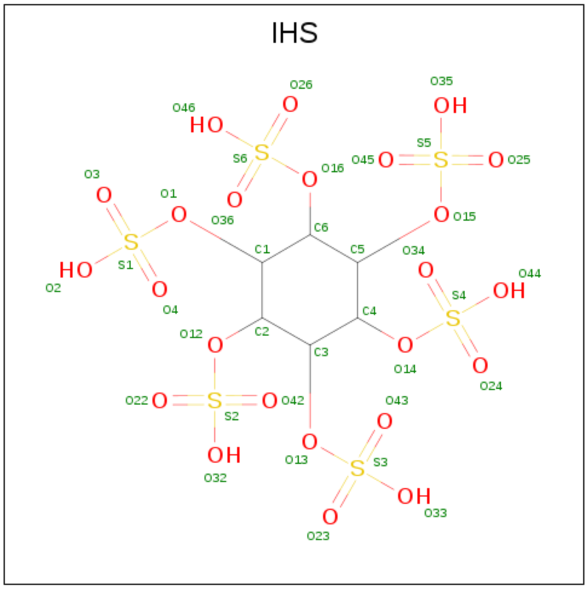

Mol	Chain	Residues	Atoms					ZeroOcc	AltConf
			Total	C	H	O	S		
10	A	1	42	6	6	24	6	0	0

- Molecule 11 is water.

Mol	Chain	Residues	Atoms		ZeroOcc	AltConf
			Total	O		
11	A	286	286	286	0	0

3 Residue-property plots (i)

These plots are drawn for all protein, RNA, DNA and oligosaccharide chains in the entry. The first graphic for a chain summarises the proportions of the various outlier classes displayed in the second graphic. The second graphic shows the sequence view annotated by issues in geometry and electron density. Residues are color-coded according to the number of geometric quality criteria for which they contain at least one outlier: green = 0, yellow = 1, orange = 2 and red = 3 or more. A red dot above a residue indicates a poor fit to the electron density (RSRZ > 2). Stretches of 2 or more consecutive residues without any outlier are shown as a green connector. Residues present in the sample, but not in the model, are shown in grey.

- Molecule 1: Purple acid phosphatase

Chain A:

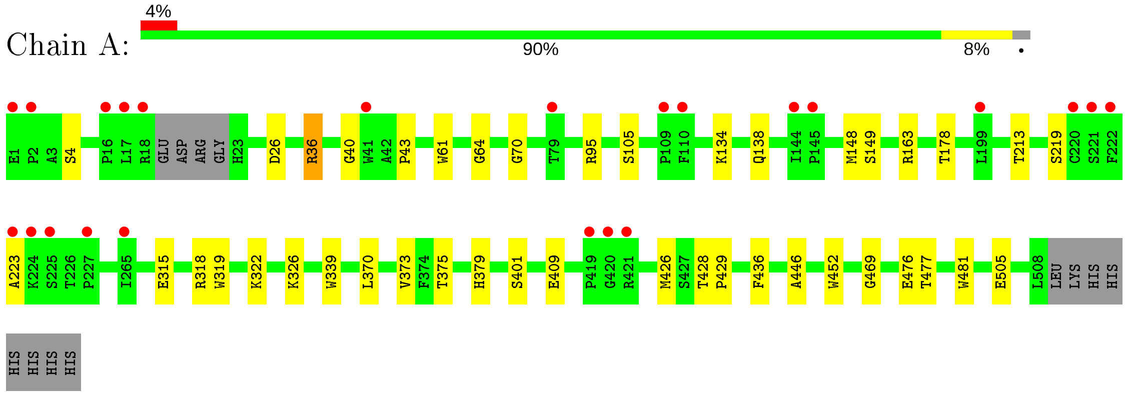

- Molecule 2: 2-acetamido-2-deoxy-beta-D-glucopyranose-(1-4)-2-acetamido-2-deoxy-beta-D-glucopyranose

Chain B:

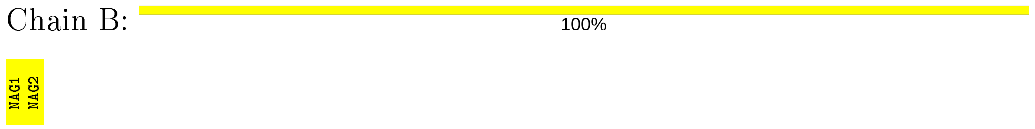

4 Data and refinement statistics (i)

Property	Value	Source
Space group	H 3	Depositor
Cell constants<br>a, b, c, α, β, γ	126.02Å 126.02Å 105.91Å<br>90.00° 90.00° 120.00°	Depositor
Resolution (Å)	38.44 – 1.68<br>38.44 – 1.68	Depositor<br>EDS
% Data completeness<br>(in resolution range)	99.9 (38.44-1.68)<br>99.9 (38.44-1.68)	Depositor<br>EDS
R_{merge}	0.06	Depositor
R_{sym}	(Not available)	Depositor
$< I/\sigma(I) >$ [1]	1.56 (at 1.68Å)	Xtriage
Refinement program	PHENIX	Depositor
R, R_{free}	0.134 , 0.176<br>0.136 , 0.132	Depositor<br>DCC
R_{free} test set	3791 reflections (5.31%)	wwPDB-VP
Wilson B-factor (Å^2)	26.2	Xtriage
Anisotropy	0.240	Xtriage
Bulk solvent k_{sol}(e/Å^3), B_{sol}(Å^2)	0.41 , 54.7	EDS
L-test for twinning[2]	$< \|L\| > = 0.50$, $< L^2 > = 0.34$	Xtriage
Estimated twinning fraction	0.020 for h,-h-k,-l	Xtriage
F_o,F_c correlation	0.98	EDS
Total number of atoms	4748	wwPDB-VP
Average B, all atoms (Å^2)	37.0	wwPDB-VP

Xtriage's analysis on translational NCS is as follows: *The largest off-origin peak in the Patterson function is 4.15% of the height of the origin peak. No significant pseudotranslation is detected.*

[1] Intensities estimated from amplitudes.

[2] Theoretical values of $< |L| >$, $< L^2 >$ for acentric reflections are 0.5, 0.333 respectively for untwinned datasets, and 0.375, 0.2 for perfectly twinned datasets.

5 Model quality (i)

5.1 Standard geometry (i)

Bond lengths and bond angles in the following residue types are not validated in this section: PGE, NAG, IHS, PO4, EDO, PG4, FE, PEG

The Z score for a bond length (or angle) is the number of standard deviations the observed value is removed from the expected value. A bond length (or angle) with $|Z| > 5$ is considered an outlier worth inspection. RMSZ is the root-mean-square of all Z scores of the bond lengths (or angles).

Mol	Chain	Bond lengths		Bond angles	
		RMSZ	#$\|Z\|$ >5	RMSZ	#$\|Z\|$ >5
1	A	0.34	0/4111	0.52	0/5609

There are no bond length outliers.

There are no bond angle outliers.

There are no chirality outliers.

There are no planarity outliers.

5.2 Too-close contacts (i)

In the following table, the Non-H and H(model) columns list the number of non-hydrogen atoms and hydrogen atoms in the chain respectively. The H(added) column lists the number of hydrogen atoms added and optimized by MolProbity. The Clashes column lists the number of clashes within the asymmetric unit, whereas Symm-Clashes lists symmetry related clashes.

Mol	Chain	Non-H	H(model)	H(added)	Clashes	Symm-Clashes
1	A	3966	0	3722	42	0
2	B	28	27	25	0	0
3	A	2	0	0	0	0
4	A	70	70	65	2	0
5	A	35	50	50	8	0
6	A	40	56	56	7	0
7	A	5	0	0	0	0
8	A	13	18	18	0	0
9	A	16	24	24	4	0
10	A	36	6	12	1	0
11	A	286	0	0	1	0
All	All	4497	251	3972	43	0

The all-atom clashscore is defined as the number of clashes found per 1000 atoms (including

hydrogen atoms). The all-atom clashscore for this structure is 5.

All (43) close contacts within the same asymmetric unit are listed below, sorted by their clash magnitude.

Atom-1	Atom-2	Interatomic distance (Å)	Clash overlap (Å)
1:A:213:THR:OG1	4:A:605:NAG:N2	2.18	0.74
1:A:134:LYS:HE2	6:A:616:PGE:H62	1.73	0.70
1:A:446:ALA:HA	5:A:613:PEG:H22	1.76	0.68
1:A:315:GLU:HG3	9:A:622:EDO:H11	1.78	0.65
1:A:64:GLY:HA2	6:A:617:PGE:H3	1.78	0.64
1:A:219:SER:HB3	10:A:625:IHS:H3	1.81	0.62
1:A:319:TRP:HB2	9:A:622:EDO:H12	1.80	0.62
1:A:315:GLU:HG3	9:A:622:EDO:C1	2.31	0.60
1:A:95:ARG:HH21	5:A:614:PEG:H22	1.66	0.60
1:A:213:THR:HG1	4:A:605:NAG:HN2	1.46	0.59
1:A:429:PRO:CG	1:A:436:PHE:HD1	2.16	0.58
1:A:163:ARG:NH2	5:A:612:PEG:H31	2.20	0.56
1:A:219:SER:HA	1:A:223:ALA:HB2	1.88	0.56
1:A:105:SER:H	5:A:611:PEG:H11	1.71	0.54
1:A:138[B]:GLN:HG3	1:A:148:MET:CE	2.37	0.54
1:A:134:LYS:HE2	6:A:616:PGE:C6	2.37	0.53
1:A:476[A]:GLU:HG3	1:A:477:THR:HG23	1.91	0.53
1:A:429:PRO:HG3	1:A:436:PHE:HD1	1.72	0.52
1:A:138[A]:GLN:HG2	1:A:149:SER:O	2.10	0.51
1:A:95:ARG:HB3	5:A:614:PEG:H32	1.92	0.51
1:A:318:ARG:HG2	9:A:622:EDO:H22	1.92	0.51
1:A:370:LEU:HD21	1:A:373:VAL:HG22	1.93	0.51
1:A:469:GLY:HA3	1:A:481:TRP:CH2	2.45	0.51
1:A:318:ARG:HE	1:A:322:LYS:HZ1	1.60	0.49
1:A:452:TRP:HA	5:A:613:PEG:H21	1.94	0.48
5:A:610:PEG:H12	6:A:616:PGE:H12	1.96	0.46
1:A:43:PRO:HB3	1:A:61:TRP:CD1	2.51	0.46
1:A:326:LYS:HD2	6:A:615:PGE:H52	1.99	0.45
1:A:138[B]:GLN:HG3	1:A:148:MET:HE2	1.98	0.45
1:A:379:HIS:HE1	1:A:409:GLU:OE1	2.00	0.45
1:A:36[B]:ARG:NH2	1:A:40:GLY:O	2.49	0.45
1:A:105:SER:H	5:A:611:PEG:C1	2.31	0.44
1:A:134:LYS:NZ	6:A:616:PGE:H6	2.32	0.44
1:A:429:PRO:HG3	1:A:436:PHE:CD1	2.53	0.43
1:A:339:TRP:O	1:A:375:THR:HA	2.19	0.43
1:A:138[B]:GLN:HG3	1:A:148:MET:HE3	2.01	0.42
1:A:326:LYS:NZ	6:A:615:PGE:H52	2.35	0.41
1:A:428:THR:N	1:A:429:PRO:CD	2.83	0.41

Continued on next page...

Continued from previous page...

Atom-1	Atom-2	Interatomic distance (Å)	Clash overlap (Å)
1:A:426:MET:CE	1:A:426:MET:HA	2.50	0.41
1:A:505:GLU:HG3	11:A:797:HOH:O	2.20	0.41
1:A:318:ARG:NE	1:A:322:LYS:HZ1	2.19	0.41
1:A:429:PRO:HG2	1:A:436:PHE:HD1	1.85	0.40
1:A:26:ASP:OD1	1:A:178:THR:HG22	2.21	0.40

There are no symmetry-related clashes.

5.3 Torsion angles (i)

5.3.1 Protein backbone (i)

In the following table, the Percentiles column shows the percent Ramachandran outliers of the chain as a percentile score with respect to all X-ray entries followed by that with respect to entries of similar resolution.

The Analysed column shows the number of residues for which the backbone conformation was analysed, and the total number of residues.

Mol	Chain	Analysed	Favoured	Allowed	Outliers	Percentiles	
1	A	507/516 (98%)	488 (96%)	18 (4%)	1 (0%)	47	29

All (1) Ramachandran outliers are listed below:

Mol	Chain	Res	Type
1	A	70	GLY

5.3.2 Protein sidechains (i)

In the following table, the Percentiles column shows the percent sidechain outliers of the chain as a percentile score with respect to all X-ray entries followed by that with respect to entries of similar resolution.

The Analysed column shows the number of residues for which the sidechain conformation was analysed, and the total number of residues.

Mol	Chain	Analysed	Rotameric	Outliers	Percentiles	
1	A	417/425 (98%)	413 (99%)	4 (1%)	76	65

All (4) residues with a non-rotameric sidechain are listed below:

Mol	Chain	Res	Type
1	A	4	SER
1	A	36[A]	ARG
1	A	36[B]	ARG
1	A	401	SER

Some sidechains can be flipped to improve hydrogen bonding and reduce clashes. There are no such sidechains identified.

5.3.3 RNA (i)

There are no RNA molecules in this entry.

5.4 Non-standard residues in protein, DNA, RNA chains (i)

There are no non-standard protein/DNA/RNA residues in this entry.

5.5 Carbohydrates (i)

2 monosaccharides are modelled in this entry.

In the following table, the Counts columns list the number of bonds (or angles) for which Mogul statistics could be retrieved, the number of bonds (or angles) that are observed in the model and the number of bonds (or angles) that are defined in the Chemical Component Dictionary. The Link column lists molecule types, if any, to which the group is linked. The Z score for a bond length (or angle) is the number of standard deviations the observed value is removed from the expected value. A bond length (or angle) with $|Z| > 2$ is considered an outlier worth inspection. RMSZ is the root-mean-square of all Z scores of the bond lengths (or angles).

Mol	Type	Chain	Res	Link	Bond lengths			Bond angles		
					Counts	RMSZ	$\#\|Z\| > 2$	Counts	RMSZ	$\#\|Z\| > 2$
2	NAG	B	1	1,2	14,14,15	1.77	2 (14%)	17,19,21	1.02	1 (5%)
2	NAG	B	2	2	14,14,15	1.97	4 (28%)	17,19,21	1.12	2 (11%)

In the following table, the Chirals column lists the number of chiral outliers, the number of chiral centers analysed, the number of these observed in the model and the number defined in the Chemical Component Dictionary. Similar counts are reported in the Torsion and Rings columns. '-' means no outliers of that kind were identified.

Mol	Type	Chain	Res	Link	Chirals	Torsions	Rings
2	NAG	B	1	1,2	-	0/6/23/26	0/1/1/1
2	NAG	B	2	2	-	0/6/23/26	0/1/1/1

All (6) bond length outliers are listed below:

Mol	Chain	Res	Type	Atoms	Z	Observed(Å)	Ideal(Å)
2	B	2	NAG	O5-C1	4.29	1.50	1.43
2	B	1	NAG	O5-C1	4.15	1.50	1.43
2	B	2	NAG	C7-N2	3.64	1.46	1.34
2	B	1	NAG	C7-N2	3.38	1.46	1.34
2	B	2	NAG	C2-N2	2.57	1.50	1.46
2	B	2	NAG	O5-C5	2.00	1.47	1.43

All (3) bond angle outliers are listed below:

Mol	Chain	Res	Type	Atoms	Z	Observed(^{o})	Ideal(^{o})
2	B	2	NAG	C8-C7-N2	3.01	121.19	116.10
2	B	1	NAG	C1-C2-N2	-2.66	105.95	110.49
2	B	2	NAG	C2-N2-C7	-2.00	120.05	122.90

There are no chirality outliers.

There are no torsion outliers.

There are no ring outliers.

No monomer is involved in short contacts.

The following is a two-dimensional graphical depiction of Mogul quality analysis of bond lengths, bond angles, torsion angles, and ring geometry for oligosaccharide.

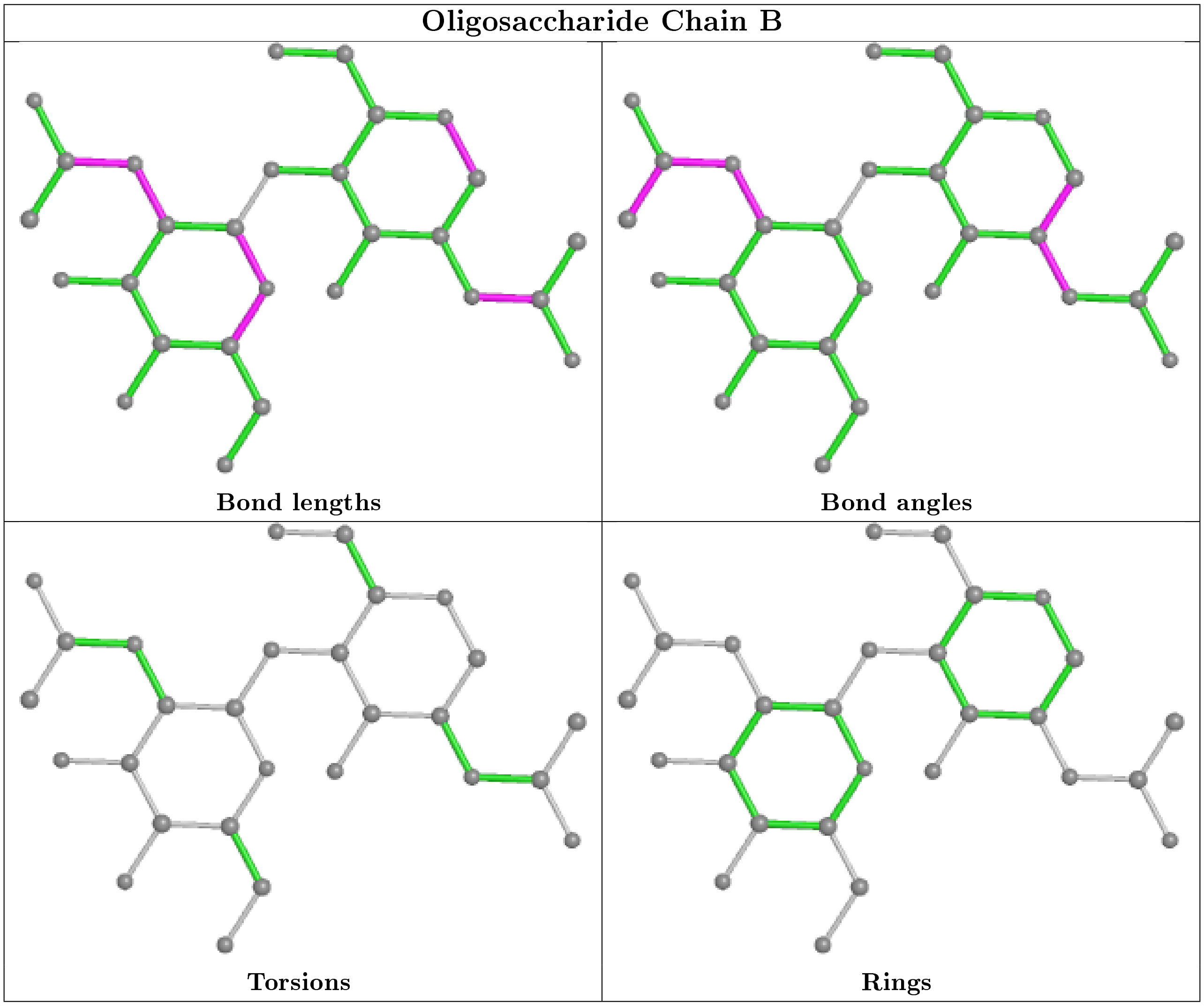

5.6 Ligand geometry ⓘ

Of 23 ligands modelled in this entry, 2 are monoatomic - leaving 21 for Mogul analysis.

In the following table, the Counts columns list the number of bonds (or angles) for which Mogul statistics could be retrieved, the number of bonds (or angles) that are observed in the model and the number of bonds (or angles) that are defined in the Chemical Component Dictionary. The Link column lists molecule types, if any, to which the group is linked. The Z score for a bond length (or angle) is the number of standard deviations the observed value is removed from the expected value. A bond length (or angle) with $|Z| > 2$ is considered an outlier worth inspection. RMSZ is the root-mean-square of all Z scores of the bond lengths (or angles).

Mol	Type	Chain	Res	Link	Bond lengths			Bond angles		
					Counts	RMSZ	$\#\|Z\| > 2$	Counts	RMSZ	$\#\|Z\| > 2$
6	PGE	A	616	-	9,9,9	0.53	0	8,8,8	0.88	0
10	IHS	A	625	-	36,36,36	1.84	12 (33%)	42,60,60	1.31	6 (14%)
5	PEG	A	613	-	6,6,6	0.46	0	5,5,5	0.34	0

Mol	Type	Chain	Res	Link	Bond lengths			Bond angles		
					Counts	RMSZ	#|Z| > 2	Counts	RMSZ	#|Z| > 2
9	EDO	A	621	-	3,3,3	0.47	0	2,2,2	0.31	0
5	PEG	A	614	-	6,6,6	0.49	0	5,5,5	0.24	0
4	NAG	A	604	1	14,14,15	1.66	2 (14%)	17,19,21	0.93	1 (5%)
4	NAG	A	607	1	14,14,15	1.84	2 (14%)	17,19,21	1.18	2 (11%)
5	PEG	A	611	-	6,6,6	0.47	0	5,5,5	0.76	0
5	PEG	A	610	-	6,6,6	0.50	0	5,5,5	0.33	0
9	EDO	A	624	-	3,3,3	0.47	0	2,2,2	0.28	0
4	NAG	A	603	1	14,14,15	1.77	2 (14%)	17,19,21	1.09	1 (5%)
9	EDO	A	623	-	3,3,3	0.48	0	2,2,2	0.29	0
6	PGE	A	615	-	9,9,9	0.52	0	8,8,8	0.35	0
8	PG4	A	620	-	12,12,12	0.52	0	11,11,11	0.35	0
7	PO4	A	619	3	4,4,4	0.90	0	6,6,6	0.59	0
4	NAG	A	605	1	14,14,15	1.88	3 (21%)	17,19,21	1.27	3 (17%)
9	EDO	A	622	-	3,3,3	0.45	0	2,2,2	0.27	0
6	PGE	A	617	-	9,9,9	0.51	0	8,8,8	0.87	0
4	NAG	A	606	1	14,14,15	1.94	3 (21%)	17,19,21	1.07	1 (5%)
5	PEG	A	612	-	6,6,6	0.51	0	5,5,5	0.48	0
6	PGE	A	618	-	9,9,9	0.51	0	8,8,8	0.50	0

In the following table, the Chirals column lists the number of chiral outliers, the number of chiral centers analysed, the number of these observed in the model and the number defined in the Chemical Component Dictionary. Similar counts are reported in the Torsion and Rings columns. '-' means no outliers of that kind were identified.

Mol	Type	Chain	Res	Link	Chirals	Torsions	Rings
6	PGE	A	616	-	-	0/7/7/7	-
10	IHS	A	625	-	-	2/30/54/54	0/1/1/1
5	PEG	A	613	-	-	0/4/4/4	-
9	EDO	A	621	-	-	1/1/1/1	-
5	PEG	A	614	-	-	1/4/4/4	-
4	NAG	A	604	1	-	0/6/23/26	0/1/1/1
4	NAG	A	607	1	-	0/6/23/26	0/1/1/1
5	PEG	A	611	-	-	1/4/4/4	-
5	PEG	A	610	-	-	0/4/4/4	-
9	EDO	A	624	-	-	1/1/1/1	-
4	NAG	A	603	1	-	0/6/23/26	0/1/1/1
9	EDO	A	623	-	-	0/1/1/1	-
6	PGE	A	615	-	-	3/7/7/7	-
8	PG4	A	620	-	-	1/10/10/10	-
4	NAG	A	605	1	-	0/6/23/26	0/1/1/1
9	EDO	A	622	-	-	0/1/1/1	-

Continued on next page...

Continued from previous page...

Mol	Type	Chain	Res	Link	Chirals	Torsions	Rings
6	PGE	A	617	-	-	4/7/7/7	-
4	NAG	A	606	1	-	0/6/23/26	0/1/1/1
5	PEG	A	612	-	-	0/4/4/4	-
6	PGE	A	618	-	-	4/7/7/7	-

All (24) bond length outliers are listed below:

Mol	Chain	Res	Type	Atoms	Z	Observed(Å)	Ideal(Å)
4	A	606	NAG	O5-C1	4.62	1.51	1.43
4	A	607	NAG	O5-C1	4.24	1.50	1.43
4	A	603	NAG	O5-C1	4.11	1.50	1.43
4	A	605	NAG	O5-C1	3.99	1.50	1.43
4	A	604	NAG	O5-C1	3.75	1.49	1.43
4	A	605	NAG	C7-N2	3.64	1.46	1.34
4	A	606	NAG	C7-N2	3.43	1.46	1.34
4	A	607	NAG	C7-N2	3.43	1.46	1.34
4	A	603	NAG	C7-N2	3.34	1.45	1.34
4	A	604	NAG	C7-N2	3.26	1.45	1.34
10	A	625	IHS	O1-S1	3.16	1.66	1.57
10	A	625	IHS	O15-S5	3.11	1.66	1.57
10	A	625	IHS	O12-S2	2.98	1.66	1.57
10	A	625	IHS	O16-S6	2.91	1.65	1.57
10	A	625	IHS	O13-S3	2.87	1.65	1.57
10	A	625	IHS	O14-S4	2.78	1.65	1.57
4	A	605	NAG	C2-N2	2.67	1.50	1.46
10	A	625	IHS	O14-C4	-2.63	1.41	1.46
10	A	625	IHS	O13-C3	-2.62	1.41	1.46
10	A	625	IHS	O12-C2	-2.58	1.41	1.46
10	A	625	IHS	O16-C6	-2.48	1.41	1.46
10	A	625	IHS	O15-C5	-2.46	1.41	1.46
10	A	625	IHS	O1-C1	-2.30	1.41	1.46
4	A	606	NAG	C2-N2	2.12	1.49	1.46

All (14) bond angle outliers are listed below:

Mol	Chain	Res	Type	Atoms	Z	Observed(°)	Ideal(°)
4	A	607	NAG	C2-N2-C7	-2.95	118.70	122.90
10	A	625	IHS	O4-S1-O3	-2.91	100.55	112.22
10	A	625	IHS	O36-S6-O26	-2.86	100.76	112.22
10	A	625	IHS	O42-S2-O22	-2.84	100.81	112.22
10	A	625	IHS	O43-S3-O23	-2.77	101.08	112.22
10	A	625	IHS	O34-S4-O24	-2.71	101.36	112.22

Continued on next page...

Continued from previous page...

Mol	Chain	Res	Type	Atoms	Z	Observed(°)	Ideal(°)
4	A	605	NAG	C8-C7-N2	2.65	120.58	116.10
4	A	603	NAG	C2-N2-C7	-2.55	119.28	122.90
4	A	607	NAG	C8-C7-N2	2.44	120.23	116.10
4	A	605	NAG	C1-C2-N2	-2.17	106.78	110.49
4	A	604	NAG	C2-N2-C7	-2.16	119.83	122.90
10	A	625	IHS	O35-S5-O25	-2.15	101.02	108.49
4	A	606	NAG	C1-O5-C5	-2.14	109.30	112.19
4	A	605	NAG	O5-C1-C2	2.04	114.50	111.29

There are no chirality outliers.

All (18) torsion outliers are listed below:

Mol	Chain	Res	Type	Atoms
6	A	617	PGE	C4-C3-O2-C2
6	A	615	PGE	O2-C3-C4-O3
6	A	617	PGE	O2-C3-C4-O3
6	A	618	PGE	C4-C3-O2-C2
6	A	615	PGE	O3-C5-C6-O4
5	A	611	PEG	C1-C2-O2-C3
5	A	614	PEG	C4-C3-O2-C2
6	A	617	PGE	C6-C5-O3-C4
6	A	615	PGE	C3-C4-O3-C5
6	A	618	PGE	C3-C4-O3-C5
10	A	625	IHS	C1-C6-O16-S6
6	A	617	PGE	C3-C4-O3-C5
9	A	624	EDO	O1-C1-C2-O2
9	A	621	EDO	O1-C1-C2-O2
10	A	625	IHS	C6-O16-S6-O46
8	A	620	PG4	O3-C5-C6-O4
6	A	618	PGE	C6-C5-O3-C4
6	A	618	PGE	O2-C3-C4-O3

There are no ring outliers.

11 monomers are involved in 21 short contacts:

Mol	Chain	Res	Type	Clashes	Symm-Clashes
6	A	616	PGE	4	0
10	A	625	IHS	1	0
5	A	613	PEG	2	0
5	A	614	PEG	2	0
5	A	611	PEG	2	0

Continued on next page...

Continued from previous page...

Mol	Chain	Res	Type	Clashes	Symm-Clashes
5	A	610	PEG	1	0
6	A	615	PGE	2	0
4	A	605	NAG	2	0
9	A	622	EDO	4	0
6	A	617	PGE	1	0
5	A	612	PEG	1	0

The following is a two-dimensional graphical depiction of Mogul quality analysis of bond lengths, bond angles, torsion angles, and ring geometry for all instances of the Ligand of Interest. In addition, ligands with molecular weight > 250 and outliers as shown on the validation Tables will also be included. For torsion angles, if less then 5% of the Mogul distribution of torsion angles is within 10 degrees of the torsion angle in question, then that torsion angle is considered an outlier. Any bond that is central to one or more torsion angles identified as an outlier by Mogul will be highlighted in the graph. For rings, the root-mean-square deviation (RMSD) between the ring in question and similar rings identified by Mogul is calculated over all ring torsion angles. If the average RMSD is greater than 60 degrees and the minimal RMSD between the ring in question and any Mogul-identified rings is also greater than 60 degrees, then that ring is considered an outlier. The outliers are highlighted in purple. The color gray indicates Mogul did not find sufficient equivalents in the CSD to analyse the geometry.

Ligand IHS A 625

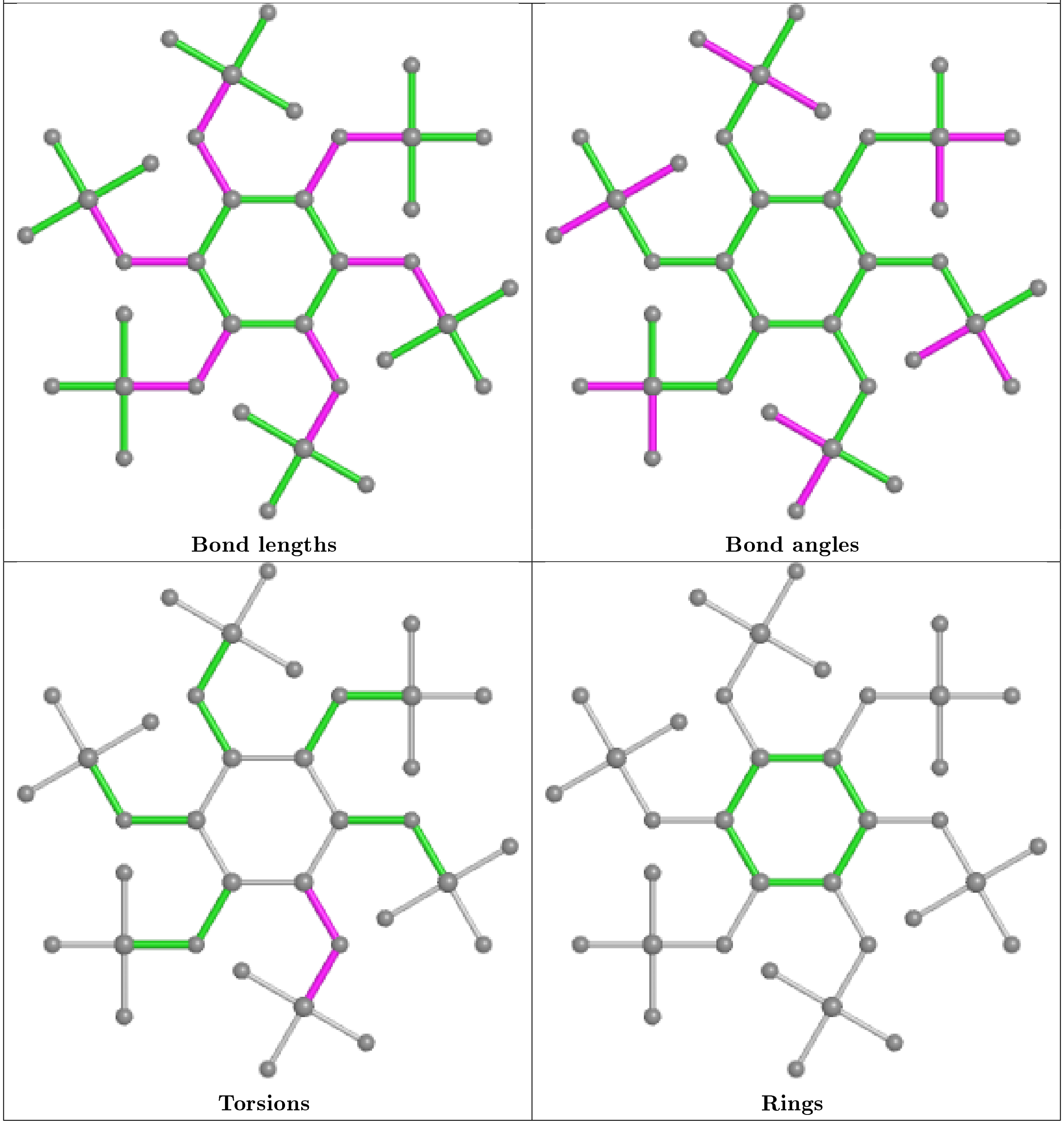

5.7 Other polymers (i)

There are no such residues in this entry.

5.8 Polymer linkage issues (i)

There are no chain breaks in this entry.

6 Fit of model and data

6.1 Protein, DNA and RNA chains

In the following table, the column labelled '#RSRZ> 2' contains the number (and percentage) of RSRZ outliers, followed by percent RSRZ outliers for the chain as percentile scores relative to all X-ray entries and entries of similar resolution. The OWAB column contains the minimum, median, 95th percentile and maximum values of the occupancy-weighted average B-factor per residue. The column labelled 'Q< 0.9' lists the number of (and percentage) of residues with an average occupancy less than 0.9.

Mol	Chain	Analysed	<RSRZ>	#RSRZ>2			OWAB(Å^2)	Q<0.9
1	A	504/516 (97%)	-0.12	23 (4%)	32	35	20, 29, 52, 76	0

All (23) RSRZ outliers are listed below:

Mol	Chain	Res	Type	RSRZ
1	A	17	LEU	7.2
1	A	220	CYS	6.0
1	A	223	ALA	4.9
1	A	222	PHE	4.8
1	A	1	GLU	4.4
1	A	2	PRO	3.6
1	A	419	PRO	3.3
1	A	18	ARG	3.1
1	A	145	PRO	3.1
1	A	221	SER	3.0
1	A	225	SER	3.0
1	A	109	PRO	2.9
1	A	110	PHE	2.8
1	A	224	LYS	2.6
1	A	421	ARG	2.6
1	A	144	ILE	2.4
1	A	16	PRO	2.4
1	A	265	ILE	2.3
1	A	41	TRP	2.2
1	A	420	GLY	2.2
1	A	79	THR	2.1
1	A	199	LEU	2.1
1	A	227	PRO	2.0

6.2 Non-standard residues in protein, DNA, RNA chains (i)

There are no non-standard protein/DNA/RNA residues in this entry.

6.3 Carbohydrates (i)

In the following table, the Atoms column lists the number of modelled atoms in the group and the number defined in the chemical component dictionary. The B-factors column lists the minimum, median, 95th percentile and maximum values of B factors of atoms in the group. The column labelled 'Q< 0.9' lists the number of atoms with occupancy less than 0.9.

Mol	Type	Chain	Res	Atoms	RSCC	RSR	B-factors(Å^2)	Q<0.9
2	NAG	B	2	14/15	0.77	0.27	57,69,87,90	0
2	NAG	B	1	14/15	0.95	0.13	37,53,65,65	0

The following is a graphical depiction of the model fit to experimental electron density for oligosaccharide. Each fit is shown from different orientation to approximate a three-dimensional view.

Electron density around Chain B:

2mF$_o$-DF$_c$ (at 0.7 rmsd) in gray
mF$_o$-DF$_c$ (at 3 rmsd) in purple (negative)
and green (positive)

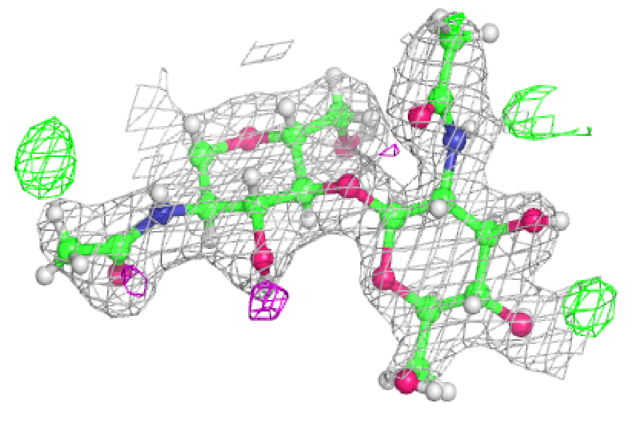

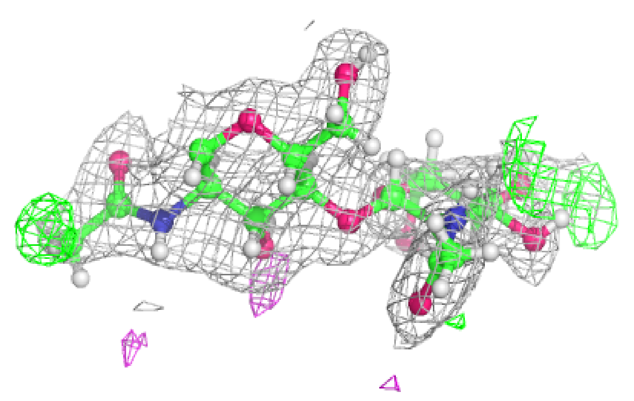

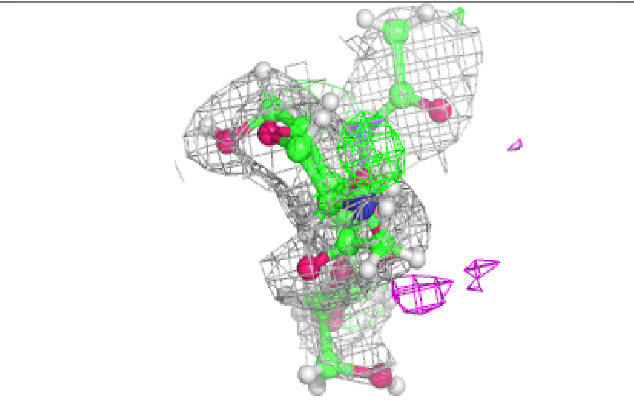

6.4 Ligands (i)

In the following table, the Atoms column lists the number of modelled atoms in the group and the number defined in the chemical component dictionary. The B-factors column lists the minimum,

median, 95th percentile and maximum values of B factors of atoms in the group. The column labelled 'Q< 0.9' lists the number of atoms with occupancy less than 0.9.

Mol	Type	Chain	Res	Atoms	RSCC	RSR	B-factors($Å^2$)	Q<0.9
9	EDO	A	623	4/4	0.73	0.16	53,65,73,78	0
5	PEG	A	613	7/7	0.78	0.26	70,84,89,90	0
4	NAG	A	605	14/15	0.78	0.18	66,79,92,94	0
5	PEG	A	614	7/7	0.79	0.13	59,71,76,78	0
5	PEG	A	612	7/7	0.81	0.20	59,70,84,86	0
4	NAG	A	606	14/15	0.81	0.23	54,67,80,83	28
10	IHS	A	625	36/36	0.82	0.15	95,116,139,146	0
6	PGE	A	617	10/10	0.83	0.27	60,75,90,92	0
9	EDO	A	624	4/4	0.83	0.12	71,85,90,91	0
6	PGE	A	618	10/10	0.84	0.13	66,81,92,92	0
6	PGE	A	616	10/10	0.86	0.14	67,80,88,90	0
8	PG4	A	620	13/13	0.86	0.11	58,72,87,88	0
5	PEG	A	610	7/7	0.87	0.15	62,74,86,89	0
9	EDO	A	621	4/4	0.88	0.18	69,83,84,85	0
5	PEG	A	611	7/7	0.89	0.21	66,79,80,80	0
9	EDO	A	622	4/4	0.89	0.16	66,80,80,81	0
6	PGE	A	615	10/10	0.90	0.12	51,64,78,78	0
3	FE	A	601	1/1	0.95	0.09	56,56,56,56	1
4	NAG	A	603	14/15	0.96	0.18	38,48,57,61	0
4	NAG	A	607	14/15	0.96	0.20	46,58,72,72	0
7	PO4	A	619	5/5	0.98	0.10	38,46,52,63	0
4	NAG	A	604	14/15	0.98	0.06	29,37,46,48	0
3	FE	A	602	1/1	1.00	0.13	20,20,20,20	1

The following is a graphical depiction of the model fit to experimental electron density of all instances of the Ligand of Interest. In addition, ligands with molecular weight > 250 and outliers as shown on the geometry validation Tables will also be included. Each fit is shown from different orientation to approximate a three-dimensional view.

Electron density around IHS A 625:

2mF$_o$-DF$_c$ (at 0.7 rmsd) in gray
mF$_o$-DF$_c$ (at 3 rmsd) in purple (negative)
and green (positive)

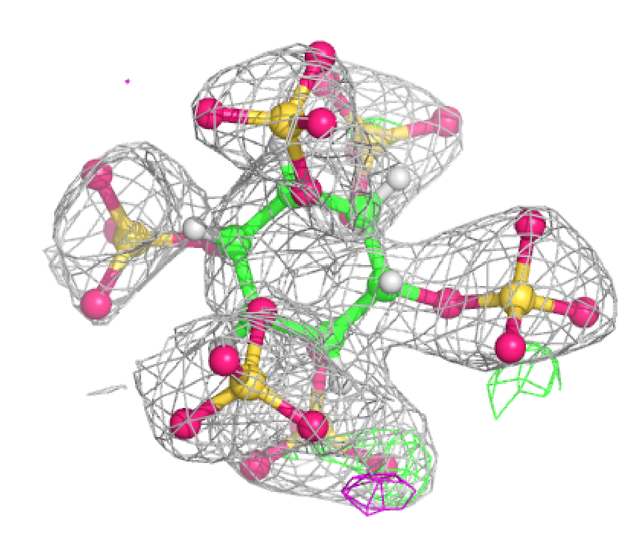

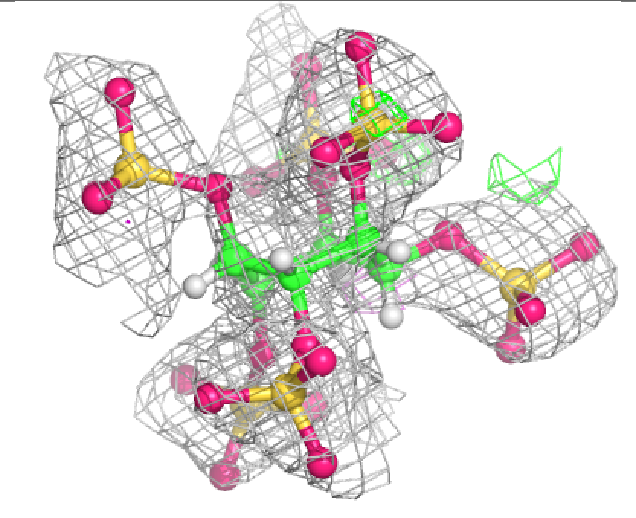

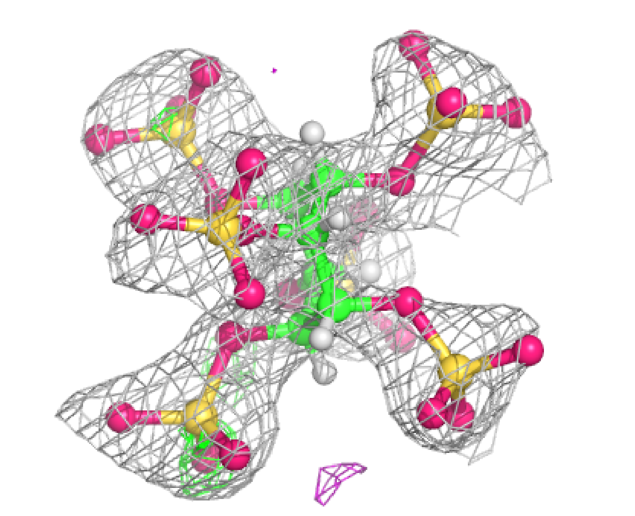

6.5 Other polymers ⓘ

There are no such residues in this entry.

Full wwPDB X-ray Structure Validation Report (i)

Aug 7, 2020 – 11:09 PM BST

PDB ID	:	6GJA
Title	:	PURPLE ACID PHYTASE FROM WHEAT ISOFORM B2 - H229A MUTANT
Authors	:	Faba-Rodriguez, R.; Brearley, C.A.; Hemmings, A.M.
Deposited on	:	2018-05-16
Resolution	:	1.50 Å(reported)

This is a Full wwPDB X-ray Structure Validation Report for a publicly released PDB entry.

We welcome your comments at *validation@mail.wwpdb.org*
A user guide is available at
https://www.wwpdb.org/validation/2017/XrayValidationReportHelp
with specific help available everywhere you see the (i) symbol.

The following versions of software and data (see references (i)) were used in the production of this report:

MolProbity	:	4.02b-467
Mogul	:	1.8.5 (274361), CSD as541be (2020)
Xtriage (Phenix)	:	1.13
EDS	:	2.13.1
buster-report	:	1.1.7 (2018)
Percentile statistics	:	20191225.v01 (using entries in the PDB archive December 25th 2019)
Refmac	:	5.8.0158
CCP4	:	7.0.044 (Gargrove)
Ideal geometry (proteins)	:	Engh & Huber (2001)
Ideal geometry (DNA, RNA)	:	Parkinson et al. (1996)
Validation Pipeline (wwPDB-VP)	:	2.13.1

1 Overall quality at a glance (i)

The following experimental techniques were used to determine the structure:
X-RAY DIFFRACTION

The reported resolution of this entry is 1.50 Å.

Percentile scores (ranging between 0-100) for global validation metrics of the entry are shown in the following graphic. The table shows the number of entries on which the scores are based.

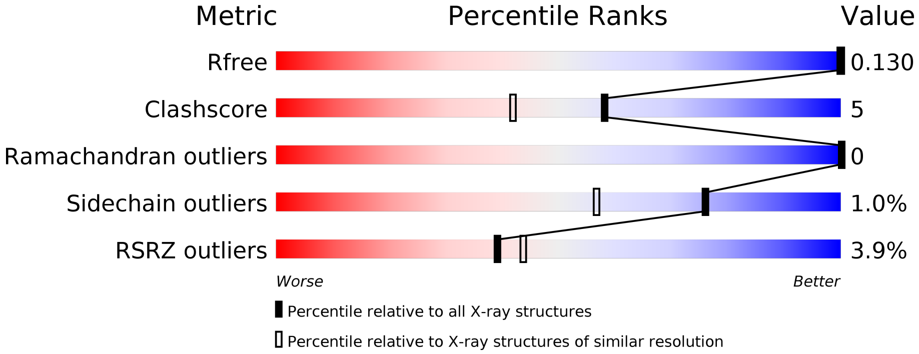

Metric	Whole archive (#Entries)	Similar resolution (#Entries, resolution range(Å))
R_{free}	130704	2936 (1.50-1.50)
Clashscore	141614	3144 (1.50-1.50)
Ramachandran outliers	138981	3066 (1.50-1.50)
Sidechain outliers	138945	3064 (1.50-1.50)
RSRZ outliers	127900	2884 (1.50-1.50)

The table below summarises the geometric issues observed across the polymeric chains and their fit to the electron density. The red, orange, yellow and green segments on the lower bar indicate the fraction of residues that contain outliers for >=3, 2, 1 and 0 types of geometric quality criteria respectively. A grey segment represents the fraction of residues that are not modelled. The numeric value for each fraction is indicated below the corresponding segment, with a dot representing fractions <=5% The upper red bar (where present) indicates the fraction of residues that have poor fit to the electron density. The numeric value is given above the bar.

Mol	Chain	Length	Quality of chain
1	A	516	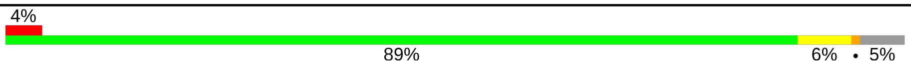
2	B	3	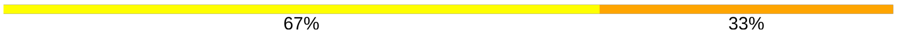

The following table lists non-polymeric compounds, carbohydrate monomers and non-standard residues in protein, DNA, RNA chains that are outliers for geometric or electron-density-fit criteria:

Mol	Type	Chain	Res	Chirality	Geometry	Clashes	Electron density
7	EDO	A	627	-	-	X	-

2 Entry composition (i)

There are 10 unique types of molecules in this entry. The entry contains 4940 atoms, of which 285 are hydrogens and 0 are deuteriums.

In the tables below, the ZeroOcc column contains the number of atoms modelled with zero occupancy, the AltConf column contains the number of residues with at least one atom in alternate conformation and the Trace column contains the number of residues modelled with at most 2 atoms.

- Molecule 1 is a protein called Purple acid phosphatase.

Mol	Chain	Residues	Atoms					ZeroOcc	AltConf	Trace
1	A	492	Total<br>3957	C<br>2528	N<br>659	O<br>746	S<br>24	0	26	0

There are 7 discrepancies between the modelled and reference sequences:

Chain	Residue	Modelled	Actual	Comment	Reference
A	229	ALA	HIS	conflict	UNP C4PKL0
A	511	HIS	-	expression tag	UNP C4PKL0
A	512	HIS	-	expression tag	UNP C4PKL0
A	513	HIS	-	expression tag	UNP C4PKL0
A	514	HIS	-	expression tag	UNP C4PKL0
A	515	HIS	-	expression tag	UNP C4PKL0
A	516	HIS	-	expression tag	UNP C4PKL0

- Molecule 2 is an oligosaccharide called beta-D-mannopyranose-(1-4)-2-acetamido-2-deoxy-beta-D-glucopyranose-(1-4)-2-acetamido-2-deoxy-beta-D-glucopyranose.

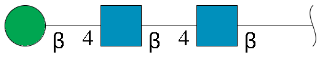

Mol	Chain	Residues	Atoms					ZeroOcc	AltConf	Trace
2	B	3	Total<br>74	C<br>22	H<br>35	N<br>2	O<br>15	0	0	0

- Molecule 3 is FE (III) ION (three-letter code: FE) (formula: Fe).

Mol	Chain	Residues	Atoms		ZeroOcc	AltConf
3	A	2	Total<br>2	Fe<br>2	0	0

- Molecule 4 is 2-acetamido-2-deoxy-beta-D-glucopyranose (three-letter code: NAG) (formula:

$C_8H_{15}NO_6$).

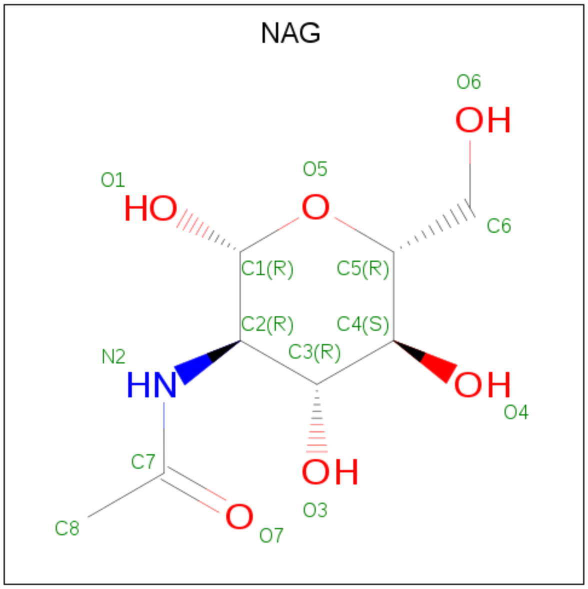

Mol	Chain	Residues	Atoms	ZeroOcc	AltConf
4	A	1	Total C H N O<br>28 8 14 1 5	0	0
4	A	1	Total C H N O<br>28 8 14 1 5	0	0
4	A	1	Total C H N O<br>28 8 14 1 5	0	0
4	A	1	Total C H N O<br>28 8 14 1 5	0	0
4	A	1	Total C H N O<br>28 8 14 1 5	0	0
4	A	1	Total C H N O<br>28 8 14 1 5	0	0

- Molecule 5 is PHOSPHATE ION (three-letter code: PO4) (formula: O_4P).

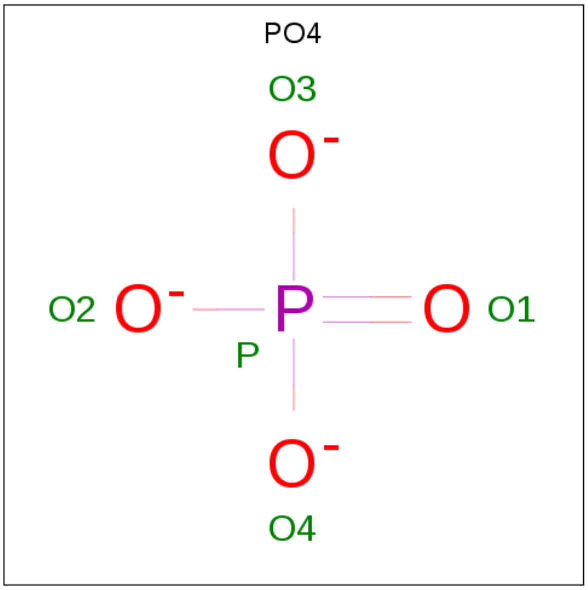

Mol	Chain	Residues	Atoms	ZeroOcc	AltConf
5	A	1	Total O P<br>5 4 1	0	0
5	A	1	Total O P<br>5 4 1	0	0
5	A	1	Total O P<br>5 4 1	0	0

- Molecule 6 is DI(HYDROXYETHYL)ETHER (three-letter code: PEG) (formula: $C_4H_{10}O_3$).

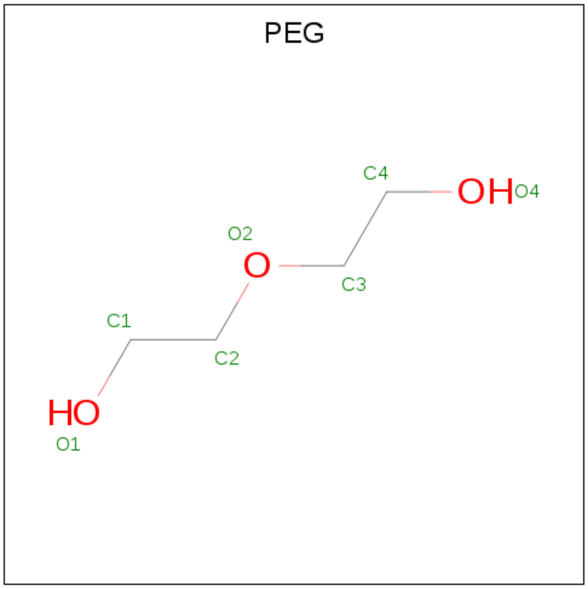

Mol	Chain	Residues	Atoms	ZeroOcc	AltConf
6	A	1	Total C H O<br>17 4 10 3	0	0

Continued on next page...

Continued from previous page...

Mol	Chain	Residues	Atoms				ZeroOcc	AltConf
6	A	1	Total 17	C 4	H 10	O 3	0	0
6	A	1	Total 17	C 4	H 10	O 3	0	0
6	A	1	Total 17	C 4	H 10	O 3	0	0
6	A	1	Total 17	C 4	H 10	O 3	0	0

- Molecule 7 is 1,2-ETHANEDIOL (three-letter code: EDO) (formula: $C_2H_6O_2$).

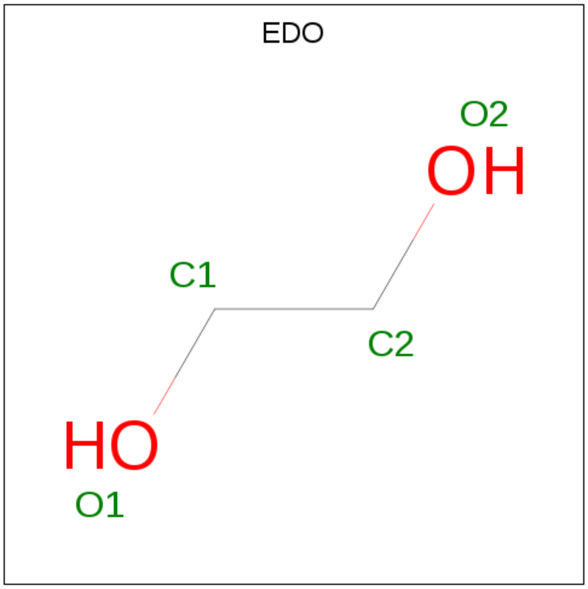

Mol	Chain	Residues	Atoms				ZeroOcc	AltConf
7	A	1	Total 10	C 2	H 6	O 2	0	0
7	A	1	Total 10	C 2	H 6	O 2	0	0
7	A	1	Total 10	C 2	H 6	O 2	0	0
7	A	1	Total 10	C 2	H 6	O 2	0	0
7	A	1	Total 10	C 2	H 6	O 2	0	0
7	A	1	Total 10	C 2	H 6	O 2	0	0
7	A	1	Total 10	C 2	H 6	O 2	0	0

Continued on next page...

Continued from previous page...

Mol	Chain	Residues	Atoms	ZeroOcc	AltConf
7	A	1	Total C H O<br>10 2 6 2	0	0

- Molecule 8 is TRIETHYLENE GLYCOL (three-letter code: PGE) (formula: $C_6H_{14}O_4$).

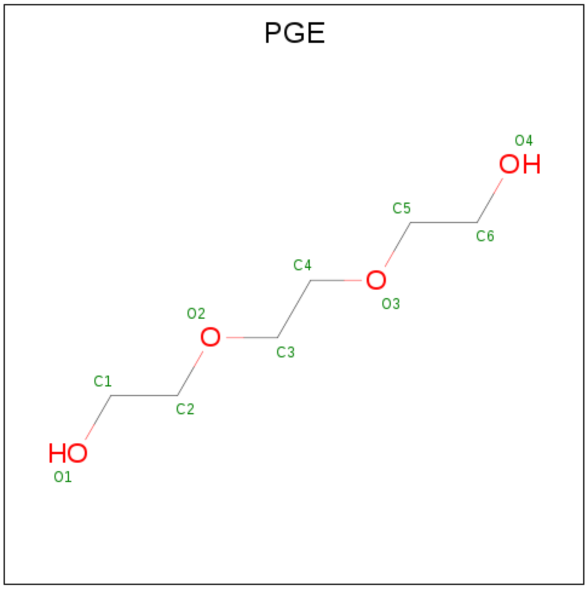

Mol	Chain	Residues	Atoms	ZeroOcc	AltConf
8	A	1	Total C H O<br>24 6 14 4	0	0
8	A	1	Total C H O<br>24 6 14 4	0	0
8	A	1	Total C H O<br>24 6 14 4	0	0

- Molecule 9 is 1-(2-METHOXY-ETHOXY)-2-{2-[2-(2-METHOXY-ETHOXY]-ETHOXY}-ETHANE (three-letter code: PG6) (formula: $C_{12}H_{26}O_6$).

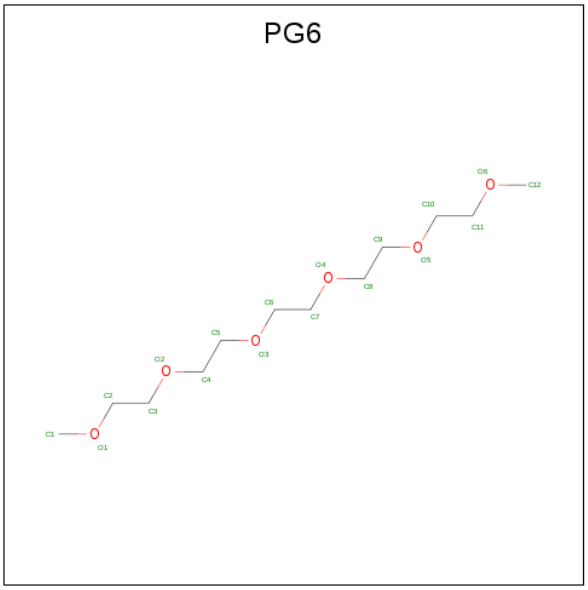

Mol	Chain	Residues	Atoms	ZeroOcc	AltConf
9	A	1	Total C H O<br>44 12 26 6	0	0

- Molecule 10 is water.

Mol	Chain	Residues	Atoms	ZeroOcc	AltConf
10	A	443	Total O<br>443 443	0	0

3 Residue-property plots (i)

These plots are drawn for all protein, RNA, DNA and oligosaccharide chains in the entry. The first graphic for a chain summarises the proportions of the various outlier classes displayed in the second graphic. The second graphic shows the sequence view annotated by issues in geometry and electron density. Residues are color-coded according to the number of geometric quality criteria for which they contain at least one outlier: green = 0, yellow = 1, orange = 2 and red = 3 or more. A red dot above a residue indicates a poor fit to the electron density (RSRZ > 2). Stretches of 2 or more consecutive residues without any outlier are shown as a green connector. Residues present in the sample, but not in the model, are shown in grey.

- Molecule 1: Purple acid phosphatase

Chain A:

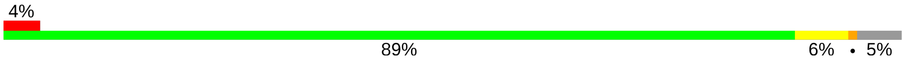

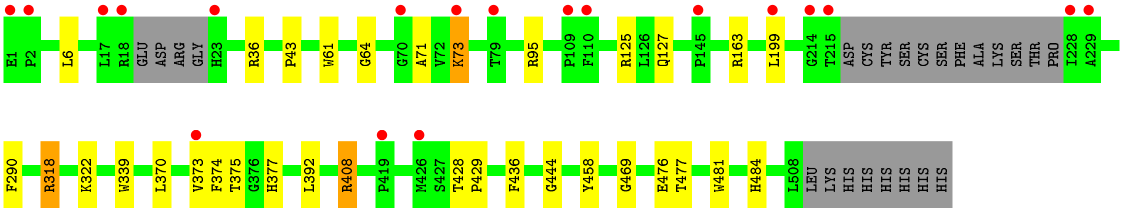

- Molecule 2: beta-D-mannopyranose-(1-4)-2-acetamido-2-deoxy-beta-D-glucopyranose-(1-4)-2-acetamido-2-deoxy-beta-D-glucopyranose

Chain B:

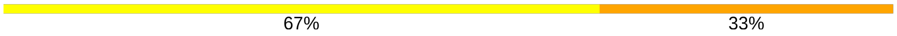

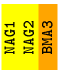

4 Data and refinement statistics (i)

Property	Value	Source
Space group	H 3	Depositor
Cell constants<br>a, b, c, α, β, γ	125.98Å 125.98Å 106.55Å<br>90.00° 90.00° 120.00°	Depositor
Resolution (Å)	36.37 – 1.50<br>36.37 – 1.50	Depositor<br>EDS
% Data completeness<br>(in resolution range)	96.5 (36.37-1.50)<br>96.5 (36.37-1.50)	Depositor<br>EDS
R_{merge}	0.06	Depositor
R_{sym}	(Not available)	Depositor
$< I/\sigma(I) >$ [1]	1.78 (at 1.50Å)	Xtriage
Refinement program	PHENIX	Depositor
R, R_{free}	0.128 , 0.152<br>0.130 , 0.130	Depositor<br>DCC
R_{free} test set	4921 reflections (5.05%)	wwPDB-VP
Wilson B-factor (Å^2)	16.0	Xtriage
Anisotropy	0.062	Xtriage
Bulk solvent k_{sol}(e/Å^3), B_{sol}(Å^2)	0.41 , 58.2	EDS
L-test for twinning[2]	$< \|L\| > = 0.51$, $< L^2 > = 0.34$	Xtriage
Estimated twinning fraction	0.015 for h,-h-k,-l	Xtriage
F_o,F_c correlation	0.98	EDS
Total number of atoms	4940	wwPDB-VP
Average B, all atoms (Å^2)	24.0	wwPDB-VP

Xtriage's analysis on translational NCS is as follows: *The largest off-origin peak in the Patterson function is 4.30% of the height of the origin peak. No significant pseudotranslation is detected.*

[1] Intensities estimated from amplitudes.

[2] Theoretical values of $< |L| >$, $< L^2 >$ for acentric reflections are 0.5, 0.333 respectively for untwinned datasets, and 0.375, 0.2 for perfectly twinned datasets.

5 Model quality (i)

5.1 Standard geometry (i)

Bond lengths and bond angles in the following residue types are not validated in this section: PGE, NAG, PO4, EDO, PG6, BMA, FE, PEG

The Z score for a bond length (or angle) is the number of standard deviations the observed value is removed from the expected value. A bond length (or angle) with $|Z| > 5$ is considered an outlier worth inspection. RMSZ is the root-mean-square of all Z scores of the bond lengths (or angles).

Mol	Chain	Bond lengths		Bond angles	
		RMSZ	#|Z| >5	RMSZ	#|Z| >5
1	A	0.33	0/4157	0.53	0/5668

There are no bond length outliers.

There are no bond angle outliers.

There are no chirality outliers.

There are no planarity outliers.

5.2 Too-close contacts (i)

In the following table, the Non-H and H(model) columns list the number of non-hydrogen atoms and hydrogen atoms in the chain respectively. The H(added) column lists the number of hydrogen atoms added and optimized by MolProbity. The Clashes column lists the number of clashes within the asymmetric unit, whereas Symm-Clashes lists symmetry related clashes.

Mol	Chain	Non-H	H(model)	H(added)	Clashes	Symm-Clashes
1	A	3957	0	3780	39	0
2	B	39	35	34	1	0
3	A	2	0	0	0	0
4	A	84	84	78	0	0
5	A	15	0	0	0	0
6	A	35	50	50	2	0
7	A	32	48	48	8	0
8	A	30	42	42	6	0
9	A	18	26	26	2	0
10	A	443	0	0	6	0
All	All	4655	285	4058	40	0

The all-atom clashscore is defined as the number of clashes found per 1000 atoms (including hydrogen atoms). The all-atom clashscore for this structure is 5.

All (40) close contacts within the same asymmetric unit are listed below, sorted by their clash magnitude.

Atom-1	Atom-2	Interatomic distance (Å)	Clash overlap (Å)
1:A:163:ARG:HH22	8:A:629:PGE:H32	1.47	0.79
1:A:444:GLY:H	9:A:631:PG6:H13	1.50	0.75
1:A:318:ARG:HD2	7:A:621:EDO:H22	1.70	0.73
1:A:322:LYS:HE3	7:A:627:EDO:O2	1.89	0.71
1:A:163:ARG:HH12	8:A:629:PGE:H3	1.58	0.69
1:A:6:LEU:O	10:A:702:HOH:O	2.13	0.67
1:A:64:GLY:HA2	6:A:617:PEG:H31	1.78	0.66
1:A:408[A]:ARG:HD3	10:A:741:HOH:O	1.98	0.63
1:A:322:LYS:HG2	7:A:627:EDO:H21	1.80	0.62
1:A:444:GLY:N	9:A:631:PG6:H32	2.13	0.62
1:A:199[B]:LEU:HD23	1:A:375:THR:CA	2.30	0.61
1:A:95:ARG:HE	7:A:622:EDO:H22	1.65	0.61
1:A:322:LYS:HE3	7:A:627:EDO:C2	2.32	0.60
1:A:199[B]:LEU:HD21	1:A:374:PHE:HB3	1.82	0.60
1:A:429:PRO:HG3	1:A:436:PHE:HD1	1.66	0.59
1:A:199[B]:LEU:HD23	1:A:375:THR:C	2.23	0.59
1:A:322:LYS:HG2	7:A:627:EDO:C2	2.36	0.55
1:A:125[A]:ARG:HH22	8:A:630:PGE:C1	2.18	0.54
1:A:163:ARG:HH22	8:A:629:PGE:C3	2.21	0.53
1:A:484:HIS:HE1	7:A:625:EDO:H12	1.76	0.51
1:A:429:PRO:CG	1:A:436:PHE:HD1	2.23	0.51
1:A:377:HIS:HA	10:A:704:HOH:O	2.11	0.50
1:A:71:ALA:O	1:A:73:LYS:HD2	2.12	0.50
1:A:127[A]:GLN:HG2	10:A:815:HOH:O	2.14	0.48
1:A:125[A]:ARG:HH22	8:A:630:PGE:H1	1.78	0.47
1:A:469:GLY:HA3	1:A:481:TRP:CH2	2.49	0.47
1:A:290:PHE:HZ	6:A:618:PEG:H32	1.79	0.47
1:A:428:THR:N	1:A:429:PRO:CD	2.79	0.46
1:A:476:GLU:HG2	1:A:477:THR:HG23	1.98	0.46
1:A:370:LEU:HD21	1:A:373:VAL:HG22	1.99	0.45
1:A:429:PRO:HG3	1:A:436:PHE:CD1	2.50	0.44
1:A:125[A]:ARG:HH22	8:A:630:PGE:H12	1.80	0.44
1:A:318:ARG:HD3	10:A:707:HOH:O	2.17	0.44
1:A:339:TRP:O	1:A:375:THR:HA	2.18	0.43
10:A:703:HOH:O	2:B:3:BMA:O3	2.20	0.43
1:A:322:LYS:HE3	7:A:627:EDO:H22	2.01	0.42
1:A:199[A]:LEU:HD11	1:A:374:PHE:HB3	2.00	0.42
1:A:392:LEU:HD21	1:A:458:TYR:HA	2.02	0.42
1:A:428:THR:N	1:A:429:PRO:HD3	2.35	0.41
1:A:43:PRO:HB3	1:A:61:TRP:CD1	2.57	0.40

There are no symmetry-related clashes.

5.3 Torsion angles (i)

5.3.1 Protein backbone (i)

In the following table, the Percentiles column shows the percent Ramachandran outliers of the chain as a percentile score with respect to all X-ray entries followed by that with respect to entries of similar resolution.

The Analysed column shows the number of residues for which the backbone conformation was analysed, and the total number of residues.

Mol	Chain	Analysed	Favoured	Allowed	Outliers	Percentiles	
1	A	513/516 (99%)	497 (97%)	16 (3%)	0	100	100

There are no Ramachandran outliers to report.

5.3.2 Protein sidechains (i)

In the following table, the Percentiles column shows the percent sidechain outliers of the chain as a percentile score with respect to all X-ray entries followed by that with respect to entries of similar resolution.

The Analysed column shows the number of residues for which the sidechain conformation was analysed, and the total number of residues.

Mol	Chain	Analysed	Rotameric	Outliers	Percentiles	
1	A	424/424 (100%)	419 (99%)	5 (1%)	71	48

All (5) residues with a non-rotameric sidechain are listed below:

Mol	Chain	Res	Type
1	A	36	ARG
1	A	73	LYS
1	A	318	ARG
1	A	408[A]	ARG
1	A	408[B]	ARG

Some sidechains can be flipped to improve hydrogen bonding and reduce clashes. There are no such sidechains identified.

5.3.3 RNA (i)

There are no RNA molecules in this entry.

5.4 Non-standard residues in protein, DNA, RNA chains (i)

There are no non-standard protein/DNA/RNA residues in this entry.

5.5 Carbohydrates (i)

3 monosaccharides are modelled in this entry.

In the following table, the Counts columns list the number of bonds (or angles) for which Mogul statistics could be retrieved, the number of bonds (or angles) that are observed in the model and the number of bonds (or angles) that are defined in the Chemical Component Dictionary. The Link column lists molecule types, if any, to which the group is linked. The Z score for a bond length (or angle) is the number of standard deviations the observed value is removed from the expected value. A bond length (or angle) with $|Z| > 2$ is considered an outlier worth inspection. RMSZ is the root-mean-square of all Z scores of the bond lengths (or angles).

Mol	Type	Chain	Res	Link	Bond lengths			Bond angles		
					Counts	RMSZ	$\#\|Z\| > 2$	Counts	RMSZ	$\#\|Z\| > 2$
2	NAG	B	1	1,2	14,14,15	1.78	3 (21%)	17,19,21	0.98	1 (5%)
2	NAG	B	2	2	14,14,15	1.88	3 (21%)	17,19,21	1.02	1 (5%)
2	BMA	B	3	2	11,11,12	1.75	2 (18%)	15,15,17	0.77	1 (6%)

In the following table, the Chirals column lists the number of chiral outliers, the number of chiral centers analysed, the number of these observed in the model and the number defined in the Chemical Component Dictionary. Similar counts are reported in the Torsion and Rings columns. '-' means no outliers of that kind were identified.

Mol	Type	Chain	Res	Link	Chirals	Torsions	Rings
2	NAG	B	1	1,2	-	0/6/23/26	0/1/1/1
2	NAG	B	2	2	-	0/6/23/26	0/1/1/1
2	BMA	B	3	2	-	0/2/19/22	0/1/1/1

All (8) bond length outliers are listed below:

Mol	Chain	Res	Type	Atoms	Z	Observed(Å)	Ideal(Å)
2	B	3	BMA	O5-C1	4.61	1.51	1.43
2	B	2	NAG	O5-C1	4.31	1.50	1.43
2	B	1	NAG	O5-C1	3.93	1.50	1.43
2	B	2	NAG	C7-N2	3.48	1.46	1.34

Continued on next page...

Continued from previous page...

Mol	Chain	Res	Type	Atoms	Z	Observed(Å)	Ideal(Å)
2	B	1	NAG	C7-N2	3.45	1.46	1.34
2	B	3	BMA	C2-C3	-2.37	1.49	1.52
2	B	2	NAG	C2-N2	2.28	1.50	1.46
2	B	1	NAG	C2-N2	2.26	1.50	1.46

All (3) bond angle outliers are listed below:

Mol	Chain	Res	Type	Atoms	Z	Observed(^{o})	Ideal(^{o})
2	B	1	NAG	C8-C7-N2	2.22	119.86	116.10
2	B	2	NAG	C8-C7-N2	2.15	119.74	116.10
2	B	3	BMA	C1-C2-C3	2.09	112.24	109.67

There are no chirality outliers.

There are no torsion outliers.

There are no ring outliers.

1 monomer is involved in 1 short contact:

Mol	Chain	Res	Type	Clashes	Symm-Clashes
2	B	3	BMA	1	0

The following is a two-dimensional graphical depiction of Mogul quality analysis of bond lengths, bond angles, torsion angles, and ring geometry for oligosaccharide.

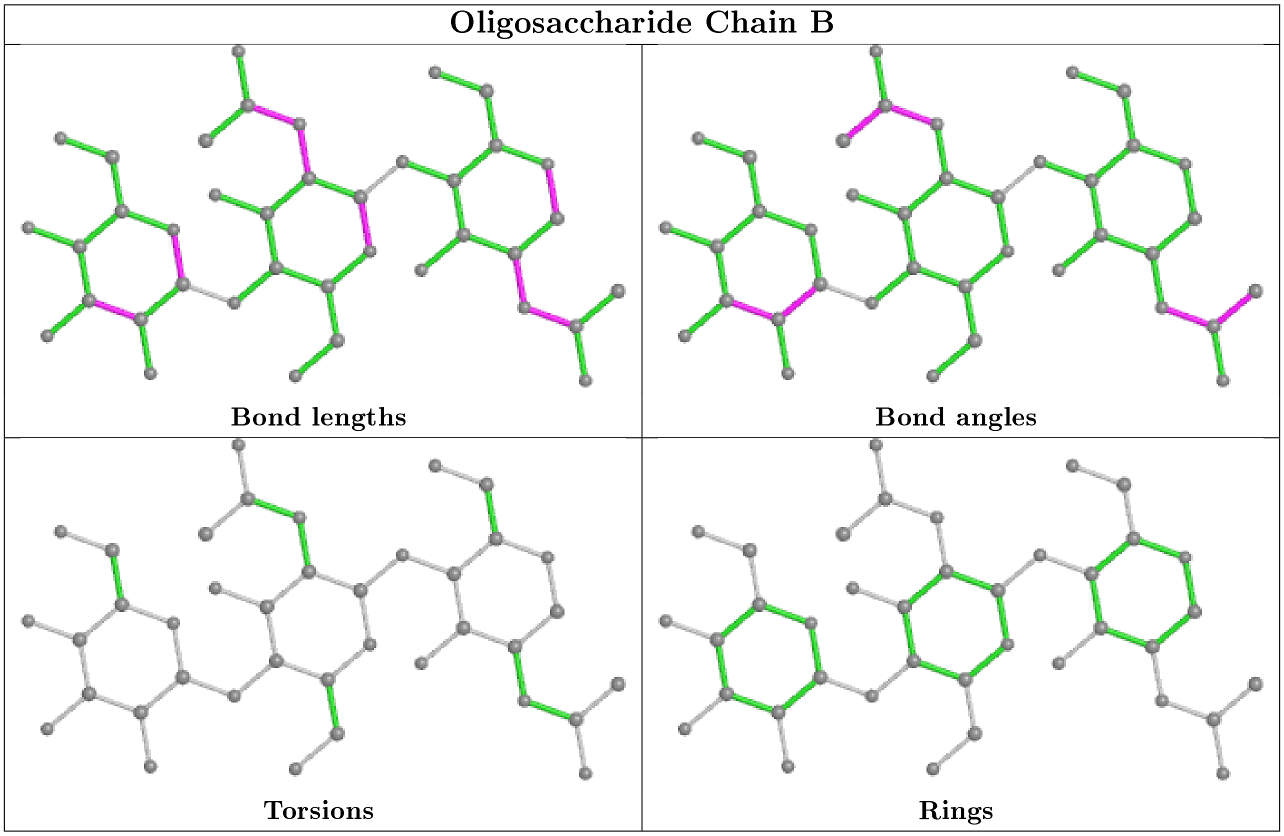

5.6 Ligand geometry (i)

Of 28 ligands modelled in this entry, 2 are monoatomic - leaving 26 for Mogul analysis.

In the following table, the Counts columns list the number of bonds (or angles) for which Mogul statistics could be retrieved, the number of bonds (or angles) that are observed in the model and the number of bonds (or angles) that are defined in the Chemical Component Dictionary. The Link column lists molecule types, if any, to which the group is linked. The Z score for a bond length (or angle) is the number of standard deviations the observed value is removed from the expected value. A bond length (or angle) with $|Z| > 2$ is considered an outlier worth inspection. RMSZ is the root-mean-square of all Z scores of the bond lengths (or angles).

Mol	Type	Chain	Res	Link	Bond lengths			Bond angles		
					Counts	RMSZ	$\#\|Z\| > 2$	Counts	RMSZ	$\#\|Z\| > 2$
6	PEG	A	619	-	6,6,6	0.48	0	5,5,5	0.61	0
4	NAG	A	606	-	14,14,15	1.87	3 (21%)	17,19,21	1.05	1 (5%)
8	PGE	A	629	-	9,9,9	0.50	0	8,8,8	0.58	0
5	PO4	A	612	3	4,4,4	0.98	0	6,6,6	0.29	0
7	EDO	A	626	-	3,3,3	0.46	0	2,2,2	0.30	0
4	NAG	A	603	1	14,14,15	1.76	2 (14%)	17,19,21	1.12	1 (5%)
7	EDO	A	622	-	3,3,3	0.47	0	2,2,2	0.34	0
8	PGE	A	630	-	9,9,9	0.52	0	8,8,8	0.35	0
4	NAG	A	604	1	14,14,15	1.59	2 (14%)	17,19,21	1.00	1 (5%)

Mol	Type	Chain	Res	Link	Bond lengths			Bond angles		
					Counts	RMSZ	#|Z| > 2	Counts	RMSZ	#|Z| > 2
4	NAG	A	605	1	14,14,15	1.91	3 (21%)	17,19,21	1.17	2 (11%)
7	EDO	A	620	-	3,3,3	0.47	0	2,2,2	0.35	0
7	EDO	A	621	-	3,3,3	0.49	0	2,2,2	0.37	0
4	NAG	A	608	1	14,14,15	1.77	3 (21%)	17,19,21	1.09	2 (11%)
9	PG6	A	631	-	17,17,17	0.52	0	16,16,16	0.47	0
4	NAG	A	607	1	14,14,15	1.91	3 (21%)	17,19,21	1.13	1 (5%)
7	EDO	A	623	-	3,3,3	0.47	0	2,2,2	0.26	0
6	PEG	A	617	-	6,6,6	0.51	0	5,5,5	0.53	0
8	PGE	A	628	-	9,9,9	0.51	0	8,8,8	0.24	0
6	PEG	A	618	-	6,6,6	0.47	0	5,5,5	0.60	0
7	EDO	A	625	-	3,3,3	0.47	0	2,2,2	0.37	0
6	PEG	A	615	-	6,6,6	0.49	0	5,5,5	0.40	0
5	PO4	A	613	-	4,4,4	0.95	0	6,6,6	0.41	0
7	EDO	A	627	-	3,3,3	0.46	0	2,2,2	0.23	0
6	PEG	A	616	-	6,6,6	0.50	0	5,5,5	0.25	0
5	PO4	A	614	-	4,4,4	0.91	0	6,6,6	0.41	0
7	EDO	A	624	-	3,3,3	0.48	0	2,2,2	0.24	0

In the following table, the Chirals column lists the number of chiral outliers, the number of chiral centers analysed, the number of these observed in the model and the number defined in the Chemical Component Dictionary. Similar counts are reported in the Torsion and Rings columns. '-' means no outliers of that kind were identified.

Mol	Type	Chain	Res	Link	Chirals	Torsions	Rings
6	PEG	A	619	-	-	1/4/4/4	-
4	NAG	A	606	-	-	0/6/23/26	0/1/1/1
8	PGE	A	629	-	-	1/7/7/7	-
7	EDO	A	626	-	-	0/1/1/1	-
4	NAG	A	603	1	-	0/6/23/26	0/1/1/1
7	EDO	A	622	-	-	0/1/1/1	-
8	PGE	A	630	-	-	1/7/7/7	-
4	NAG	A	604	1	-	0/6/23/26	0/1/1/1
4	NAG	A	605	1	-	0/6/23/26	0/1/1/1
7	EDO	A	620	-	-	0/1/1/1	-
7	EDO	A	621	-	-	0/1/1/1	-
4	NAG	A	608	1	-	0/6/23/26	0/1/1/1
9	PG6	A	631	-	-	8/15/15/15	-
4	NAG	A	607	1	-	4/6/23/26	0/1/1/1
7	EDO	A	623	-	-	0/1/1/1	-
6	PEG	A	617	-	-	2/4/4/4	-
8	PGE	A	628	-	-	0/7/7/7	-

Continued on next page...

Continued from previous page...

Mol	Type	Chain	Res	Link	Chirals	Torsions	Rings
6	PEG	A	618	-	-	2/4/4/4	-
7	EDO	A	625	-	-	1/1/1/1	-
6	PEG	A	615	-	-	1/4/4/4	-
7	EDO	A	627	-	-	1/1/1/1	-
6	PEG	A	616	-	-	0/4/4/4	-
7	EDO	A	624	-	-	0/1/1/1	-

All (16) bond length outliers are listed below:

Mol	Chain	Res	Type	Atoms	Z	Observed(Å)	Ideal(Å)
4	A	605	NAG	O5-C1	4.44	1.50	1.43
4	A	607	NAG	O5-C1	4.27	1.50	1.43
4	A	606	NAG	O5-C1	4.21	1.50	1.43
4	A	603	NAG	O5-C1	3.89	1.49	1.43
4	A	608	NAG	O5-C1	3.79	1.49	1.43
4	A	607	NAG	C7-N2	3.60	1.46	1.34
4	A	606	NAG	C7-N2	3.52	1.46	1.34
4	A	605	NAG	C7-N2	3.46	1.46	1.34
4	A	604	NAG	O5-C1	3.45	1.49	1.43
4	A	608	NAG	C7-N2	3.45	1.46	1.34
4	A	603	NAG	C7-N2	3.37	1.46	1.34
4	A	604	NAG	C7-N2	3.12	1.45	1.34
4	A	607	NAG	C2-N2	2.50	1.50	1.46
4	A	606	NAG	C2-N2	2.17	1.50	1.46
4	A	605	NAG	C2-N2	2.17	1.50	1.46
4	A	608	NAG	C2-N2	2.09	1.49	1.46

All (8) bond angle outliers are listed below:

Mol	Chain	Res	Type	Atoms	Z	Observed(^{o})	Ideal(^{o})
4	A	607	NAG	C8-C7-N2	2.92	121.05	116.10
4	A	608	NAG	C2-N2-C7	-2.66	119.12	122.90
4	A	604	NAG	O5-C1-C2	-2.63	107.14	111.29
4	A	605	NAG	C2-N2-C7	-2.57	119.24	122.90
4	A	606	NAG	C1-C2-N2	-2.42	106.36	110.49
4	A	608	NAG	C8-C7-N2	2.39	120.14	116.10
4	A	605	NAG	C8-C7-N2	2.12	119.69	116.10
4	A	603	NAG	C2-N2-C7	-2.08	119.94	122.90

There are no chirality outliers.

All (22) torsion outliers are listed below:

Mol	Chain	Res	Type	Atoms
4	A	607	NAG	C8-C7-N2-C2
4	A	607	NAG	O7-C7-N2-C2
9	A	631	PG6	C9-C8-O4-C7
6	A	617	PEG	O1-C1-C2-O2
6	A	617	PEG	C4-C3-O2-C2
8	A	629	PGE	O1-C1-C2-O2
4	A	607	NAG	C4-C5-C6-O6
4	A	607	NAG	O5-C5-C6-O6
6	A	615	PEG	O1-C1-C2-O2
7	A	625	EDO	O1-C1-C2-O2
9	A	631	PG6	C5-C4-O2-C3
6	A	618	PEG	C4-C3-O2-C2
6	A	618	PEG	C1-C2-O2-C3
9	A	631	PG6	C10-C11-O6-C12
6	A	619	PEG	C4-C3-O2-C2
7	A	627	EDO	O1-C1-C2-O2
8	A	630	PGE	C4-C3-O2-C2
9	A	631	PG6	C11-C10-O5-C9
9	A	631	PG6	O1-C2-C3-O2
9	A	631	PG6	C3-C2-O1-C1
9	A	631	PG6	O5-C10-C11-O6
9	A	631	PG6	O2-C4-C5-O3

There are no ring outliers.

9 monomers are involved in 18 short contacts:

Mol	Chain	Res	Type	Clashes	Symm-Clashes
8	A	629	PGE	3	0
7	A	622	EDO	1	0
8	A	630	PGE	3	0
7	A	621	EDO	1	0
9	A	631	PG6	2	0
6	A	617	PEG	1	0
6	A	618	PEG	1	0
7	A	625	EDO	1	0
7	A	627	EDO	5	0

The following is a two-dimensional graphical depiction of Mogul quality analysis of bond lengths, bond angles, torsion angles, and ring geometry for all instances of the Ligand of Interest. In addition, ligands with molecular weight > 250 and outliers as shown on the validation Tables will also be included. For torsion angles, if less then 5% of the Mogul distribution of torsion angles is within 10 degrees of the torsion angle in question, then that torsion angle is considered an outlier. Any bond that is central to one or more torsion angles identified as an outlier by Mogul will be

highlighted in the graph. For rings, the root-mean-square deviation (RMSD) between the ring in question and similar rings identified by Mogul is calculated over all ring torsion angles. If the average RMSD is greater than 60 degrees and the minimal RMSD between the ring in question and any Mogul-identified rings is also greater than 60 degrees, then that ring is considered an outlier. The outliers are highlighted in purple. The color gray indicates Mogul did not find sufficient equivalents in the CSD to analyse the geometry.

Ligand PG6 A 631

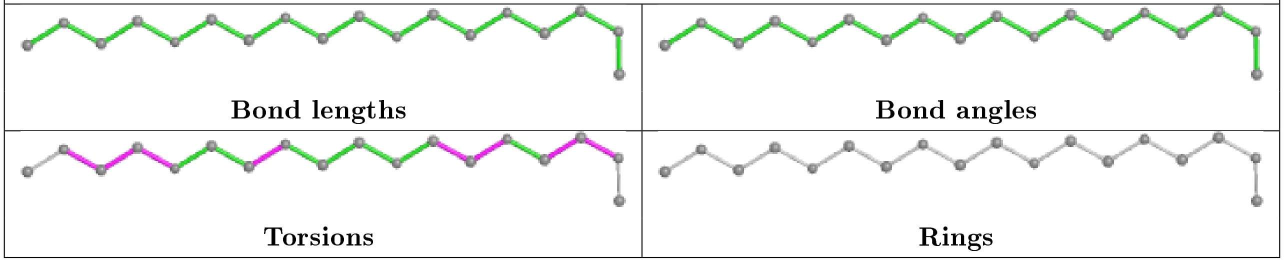

5.7 Other polymers (i)

There are no such residues in this entry.

5.8 Polymer linkage issues (i)

There are no chain breaks in this entry.

6 Fit of model and data (i)

6.1 Protein, DNA and RNA chains (i)

In the following table, the column labelled '#RSRZ> 2' contains the number (and percentage) of RSRZ outliers, followed by percent RSRZ outliers for the chain as percentile scores relative to all X-ray entries and entries of similar resolution. The OWAB column contains the minimum, median, 95th percentile and maximum values of the occupancy-weighted average B-factor per residue. The column labelled 'Q< 0.9' lists the number of (and percentage) of residues with an average occupancy less than 0.9.

Mol	Chain	Analysed	<RSRZ>	#RSRZ>2			OWAB(Å^2)	Q<0.9
1	A	492/516 (95%)	0.09	19 (3%)	39	44	12, 17, 34, 59	0

All (19) RSRZ outliers are listed below:

Mol	Chain	Res	Type	RSRZ
1	A	17	LEU	6.3
1	A	215	THR	5.0
1	A	2	PRO	5.0
1	A	228	ILE	4.8
1	A	110	PHE	3.9
1	A	229	ALA	3.5
1	A	109	PRO	3.3
1	A	214	GLY	2.7
1	A	1	GLU	2.7
1	A	70	GLY	2.5
1	A	79	THR	2.5
1	A	23	HIS	2.4
1	A	145	PRO	2.4
1	A	18	ARG	2.3
1	A	199[A]	LEU	2.3
1	A	73	LYS	2.3
1	A	419	PRO	2.2
1	A	373	VAL	2.1
1	A	426	MET	2.1

6.2 Non-standard residues in protein, DNA, RNA chains (i)

There are no non-standard protein/DNA/RNA residues in this entry.

6.3 Carbohydrates (i)

In the following table, the Atoms column lists the number of modelled atoms in the group and the number defined in the chemical component dictionary. The B-factors column lists the minimum, median, 95th percentile and maximum values of B factors of atoms in the group. The column labelled 'Q< 0.9' lists the number of atoms with occupancy less than 0.9.

Mol	Type	Chain	Res	Atoms	RSCC	RSR	B-factors(Å^2)	Q<0.9
2	BMA	B	3	11/12	0.91	0.30	43,49,58,59	0
2	NAG	B	2	14/15	0.92	0.22	27,34,45,48	0
2	NAG	B	1	14/15	0.97	0.11	19,28,39,39	0

The following is a graphical depiction of the model fit to experimental electron density for oligosaccharide. Each fit is shown from different orientation to approximate a three-dimensional view.

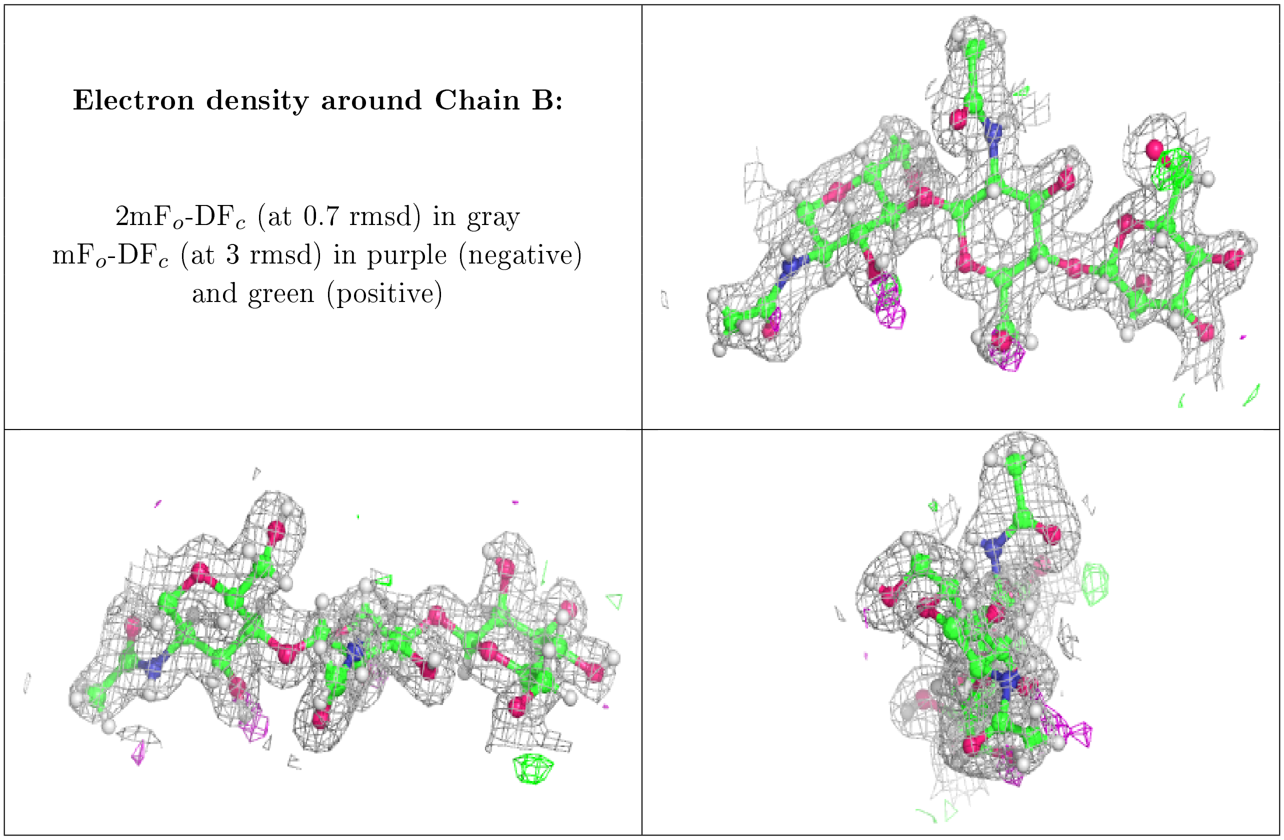

6.4 Ligands (i)

In the following table, the Atoms column lists the number of modelled atoms in the group and the number defined in the chemical component dictionary. The B-factors column lists the minimum, median, 95th percentile and maximum values of B factors of atoms in the group. The column labelled 'Q< 0.9' lists the number of atoms with occupancy less than 0.9.

Mol	Type	Chain	Res	Atoms	RSCC	RSR	B-factors(Å^2)	Q<0.9
4	NAG	A	606	14/15	0.72	0.21	50,56,66,67	28
7	EDO	A	624	4/4	0.75	0.27	62,75,75,76	0
5	PO4	A	614	5/5	0.77	0.25	77,77,77,78	5
8	PGE	A	629	10/10	0.78	0.19	51,62,73,73	0
6	PEG	A	617	7/7	0.81	0.15	49,59,64,64	0
6	PEG	A	619	7/7	0.82	0.22	47,57,63,63	0
7	EDO	A	627	4/4	0.82	0.27	39,49,58,58	0
4	NAG	A	607	14/15	0.83	0.17	30,38,44,46	28
6	PEG	A	618	7/7	0.85	0.17	53,64,69,70	0
9	PG6	A	631	18/18	0.85	0.17	36,48,60,60	0
7	EDO	A	622	4/4	0.86	0.14	55,66,67,68	0
7	EDO	A	625	4/4	0.86	0.20	58,70,70,71	0
5	PO4	A	613	5/5	0.86	0.23	63,65,66,67	5
8	PGE	A	630	10/10	0.87	0.15	53,64,69,71	0
8	PGE	A	628	10/10	0.89	0.21	56,68,74,75	0
6	PEG	A	615	7/7	0.89	0.12	49,59,60,61	0
4	NAG	A	605	14/15	0.89	0.24	45,56,67,70	0
7	EDO	A	623	4/4	0.92	0.22	54,65,66,68	0
6	PEG	A	616	7/7	0.92	0.19	40,51,64,66	0
7	EDO	A	621	4/4	0.94	0.22	43,51,55,57	0
7	EDO	A	620	4/4	0.94	0.10	33,47,54,58	0
7	EDO	A	626	4/4	0.95	0.13	42,51,52,53	0
4	NAG	A	603	14/15	0.96	0.20	26,35,41,42	0
4	NAG	A	608	14/15	0.97	0.17	27,35,50,50	0
5	PO4	A	612	5/5	0.97	0.08	22,22,25,25	5
4	NAG	A	604	14/15	0.98	0.08	16,21,26,30	0
3	FE	A	601	1/1	1.00	0.05	20,20,20,20	1
3	FE	A	602	1/1	1.00	0.07	14,14,14,14	0

The following is a graphical depiction of the model fit to experimental electron density of all instances of the Ligand of Interest. In addition, ligands with molecular weight > 250 and outliers as shown on the geometry validation Tables will also be included. Each fit is shown from different orientation to approximate a three-dimensional view.

Electron density around PG6 A 631:

2mF$_o$-DF$_c$ (at 0.7 rmsd) in gray
mF$_o$-DF$_c$ (at 3 rmsd) in purple (negative)
and green (positive)

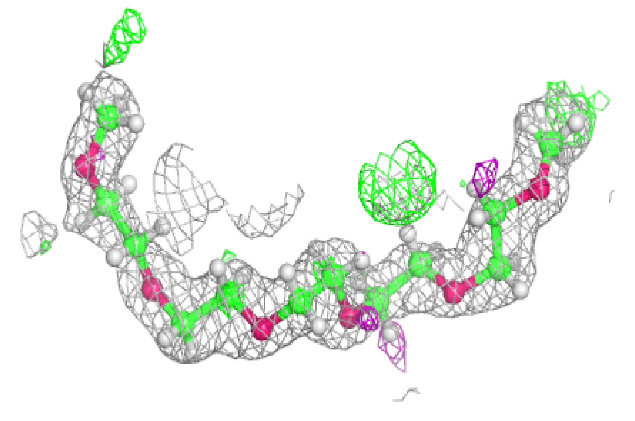

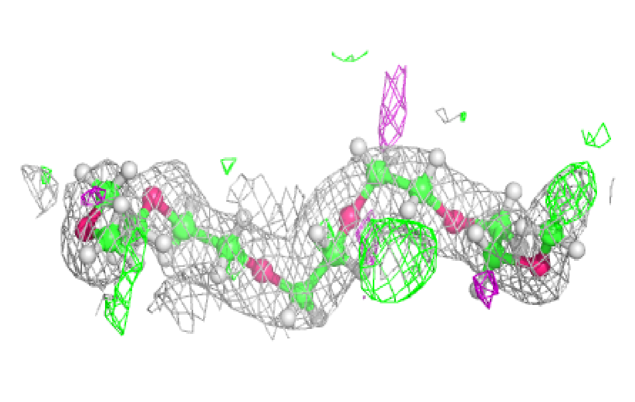

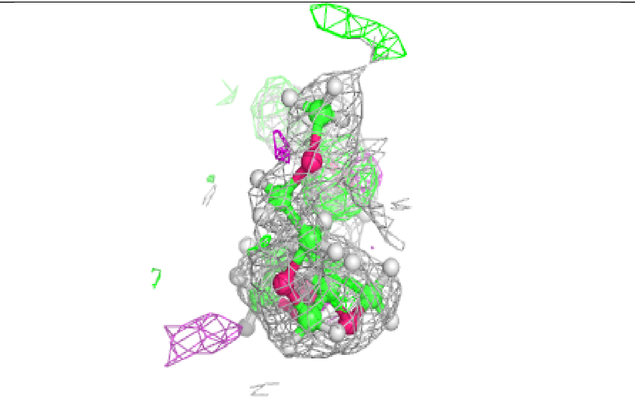

6.5 Other polymers (i)

There are no such residues in this entry.
